# The Transcriptome of *Trichuris suis* – First Molecular Insights into a Parasite with Curative Properties for Key Immune Diseases of Humans

**DOI:** 10.1371/journal.pone.0023590

**Published:** 2011-08-24

**Authors:** Cinzia Cantacessi, Neil D. Young, Peter Nejsum, Aaron R. Jex, Bronwyn E. Campbell, Ross S. Hall, Stig M. Thamsborg, Jean-Pierre Scheerlinck, Robin B. Gasser

**Affiliations:** 1 Department of Veterinary Science, The University of Melbourne, Parkville, Victoria, Australia; 2 Departments of Veterinary Disease Biology and Basic Animal and Veterinary Science, University of Copenhagen, Frederiksberg, Denmark; 3 Centre for Animal Biotechnology, The University of Melbourne, Parkville, Australia; Griffith University, Australia

## Abstract

**Background:**

Iatrogenic infection of humans with *Trichuris suis* (a parasitic nematode of swine) is being evaluated or promoted as a biological, curative treatment of immune diseases, such as inflammatory bowel disease (IBD) and ulcerative colitis, in humans. Although it is understood that short-term *T. suis* infection in people with such diseases usually induces a modified Th2-immune response, nothing is known about the molecules in the parasite that induce this response.

**Methodology/Principal Findings:**

As a first step toward filling the gaps in our knowledge of the molecular biology of *T. suis*, we characterised the transcriptome of the adult stage of this nematode employing next-generation sequencing and bioinformatic techniques. A total of ∼65,000,000 reads were generated and assembled into ∼20,000 contiguous sequences ( = contigs); ∼17,000 peptides were predicted and classified based on homology searches, protein motifs and gene ontology and biological pathway mapping.

**Conclusions:**

These analyses provided interesting insights into a number of molecular groups, particularly predicted excreted/secreted molecules (n = 1,288), likely to be involved in the parasite-host interactions, and also various molecules (n = 120) linked to chemokine, T-cell receptor and TGF-β signalling as well as leukocyte transendothelial migration and natural killer cell-mediated cytotoxicity, which are likely to be immuno-regulatory or -modulatory in the infected host. This information provides a conceptual framework within which to test the immunobiological basis for the curative effect of *T. suis* infection in humans against some immune diseases. Importantly, the *T. suis* transcriptome characterised herein provides a curated resource for detailed studies of the immuno-molecular biology of this parasite, and will underpin future genomic and proteomic explorations.

## Introduction

Parasitic nematodes that infect the gastrointestinal tracts of humans are of major socioeconomic significance worldwide [1], [2]. Amongst these nematodes are the soil-transmitted helminths (STHs), including *Ancylostoma duodenale*, *Necator americanus*, *Ascaris* spp. and *Trichuris* spp., which are estimated to infect one-sixth of all humans [3], [4]. Infections by these parasites cause symptoms that range from malabsorption and malnutrition (e.g., for *Ascaris* spp. and *Trichuris* spp.) to iron-deficiency anaemia, physical and mental retardation and adverse maternal-foetal outcomes (e.g., for *A. duodenale* and *N. americanus*), which severely impact on the social and economic development of the affected communities [2]. In contrast to hookworms, *Trichuris* is usually mildly pathogenic in humans, and only pathogenic in people infected with large numbers of adult worms [4]–[6].

There is an increasing body of evidence suggesting that, although STHs can a have major adverse impact on human health, people in endemic countries tend to suffer significantly less from (chronic) immunopathological diseases [7]. Interestingly, this situation contrasts published evidence [8]–[11] for developed countries, where people who are not exposed to STHs (and/or other parasites; cf. [12]) suffer significantly more from these diseases, such as inflammatory bowel diseases (IBD; including Crohn's disease and ulcerative colitis [9]) and asthma [8], [10], [11]. The apparent increase in both incidence and severity of these immune diseases in developed countries has been linked, at least in part, to a modern lifestyle, in which there is a lack of exposure to parasites throughout life (cf. “hygiene hypothesis” [12]–[15]). Interestingly, various studies [16]–[20] have indicated that iatrogenic infections of human patients suffering from immunopathological diseases, such as IBD, with selected intestinal nematodes, such as *Trichuris suis*, can significantly suppress clinical symptoms associated with these persistent and complex diseases. Although the mechanisms by which *T. suis* modulates the host's immune system are still unclear [14], [17], [21], studies have suggested that a modified CD4+ T helper 2 (Th2)-immune response and the production of anti-inflammatory cytokines, including the interleukins (IL-) IL-4 and IL-10, contribute to the inhibition of effector mechanisms [19], [22], [23]. The advent of advanced proteomic and genomic tools provides enormous scope for investigations of the molecular mechanisms that take place between *T. suis* and humans who are affected by autoimmune or other immune diseases. A starting point to underpin such investigations could be to characterise and catalogue molecules in the parasite and to construct a conceptual framework to subsequently test hypotheses regarding the parasite-host interplay at the molecular level. Therefore, we explore, for the first time on a large scale, the transcriptome of the adult stage of *T. suis*, in order to establish a curated resource for future investigations of the immuno-molecular biology of this parasite in humans and pigs. For this purpose, we employ Illumina technology [24] and an advanced bioinformatic platform [25].

## Materials and Methods

### Production and procurement of parasite material

Adult specimens of *T. suis* were collected from pigs with naturally acquired infection from an organic farm in Denmark. Pigs were killed using a captive bolt and exsanguination, according to animal ethics approval number 2005/561-1060 (University of Copenhagen). The colon and caecum were cut open, and worms removed and washed extensively in physiological saline (37°C). The worms were then washed four times (15 min each) in Hank's solution (Sigma-Aldrich) and incubated in RPMI 1640 medium (Gibco), containing glucose (1% w/v) and penicillin (500 IU/ml), streptomycin (0.5 mg/ml) and fungizone (1.25 µg/ml) for 20 min at 37°C [26]. During the last washing step, live worms were transferred in RNAse/DNAse-free cryo-tubes, snap-frozen in liquid nitrogen and then stored at −80°C until RNA isolation.

### RNA isolation and Illumina sequencing

The method of paired-end RNA-seq [24] was used to sequence the transcriptome of *T. suis*. In brief, total RNA was extracted from adult *T. suis* (n = 40; both sexes) using the TriPure reagent (Roche) and *DN*ase I-treated [27]. RNA amounts were estimated spectrophotometrically (NanoDrop Technologies), and RNA integrity was verified using a 2100 BioAnalyser (Agilent). Polyadenylated (polyA+) RNA was purified from 10 µg of total RNA using Sera-mag oligo(dT) beads, fragmented to a length of 100–500 bases, reverse transcribed using random hexamers, end-repaired and adaptor-ligated, according to the manufacturer's protocol (Illumina). Ligated products of ∼300 (average: 336) base pairs (bp) were excised from agarose and PCR-amplified (15 cycles) [27]. Products were cleaned using a MinElute column (Qiagen) and paired-end sequenced on a Genome Analyzer II (Illumina), according to manufacturer's instructions.

### Bioinformatic analyses

The 100 bp single-read sequences generated from the non-normalized cDNA library representing the adult stage of *T. suis* were assembled using the program Velvet v1.0.19 (http://www.ebi.ac.uk/~zerbino/velvet/; [28]), followed by Oases v0.1.18 software (http://www.ebi.ac.uk/~zerbino/oases/). Adapter sequences and sequences with suboptimal read quality (i.e., PHRED score of <32.0) were eliminated. The remaining sequences (99%) were used to construct a de Bruijn-graph using a *k*-mer value of 61 bp. The raw sequence reads from the cDNA library were then mapped to the non-redundant sequence data using SOAPAligner [27]–[29]. In brief, raw reads were aligned to the assembled, non-redundant transcriptomic data, such that each read was mapped to a unique transcript. Reads that mapped to more than one transcript (called “multi-reads”) were randomly allocated to a unique transcript, such that they were recorded only once. To provide a relative assessment of transcript-abundance, the numbers of raw reads that mapped to individual contigs were normalized for sequence length (i.e., reads per kilobase per million reads, RPKM; [30]).

The non-redundant transcriptomic dataset for *T. suis* was then analysed using an established approach [25]. Briefly, assembled contigs were compared (using BLASTn and BLASTx algorithms; [31]) with sequences available in public databases, including NCBI (www.ncbi.nlm.nih.gov), ENSEMBL (http://www.ensembl.org/) and the EMBL-EBI Parasite Genome Blast Server (www.ebi.ac.uk) to identify putative homologues in other nematodes and organisms other than nematodes, including *Homo sapiens* (human) and *Sus scrofa* (swine) (March 2011; e-value cut-off: <10^−5^). Proteins were conceptually translated from the open reading frames (ORFs) of individual sequences using ESTScan [32] and compared with protein data available for *Caenorhabditis elegans* (free-living nematode) (release WS223; www.wormbase.org; [33], [34]) as well as *Pristionchus pacificus* [35], the enoplid nematode *Trichinella spiralis* [36] and the ‘root-knot’ parasitic nematode of plants, *Meloidogyne hapla* [37], using the program JackHmmer (http://hmmer.janelia.org/; [38]).

Protein-coding sequences were classified functionally using InterProScan [39], employing the default search parameters. Based on their homology to conserved domains and protein families, proteins predicted for *T. suis* were assigned parental (i.e., level 2) Gene Ontology (GO) terms (i.e., ‘biological process’, ‘cellular component’ and ‘molecular function’) (http://www.geneontology.org/; [40]) and displayed using the WEGO tool (http://wego.genomics.org.cn/cgi-bin/wego/index.pl; [41]). Inferred proteins with homologues in other organisms were mapped to conserved biological pathways utilizing the Kyoto Encyclopedia of Genes and Genomes (KEGG) Orthology-Based Annotation System ( = KOBAS) [42]. Signal peptides were also predicted using the program SignalP 3.0, employing the neural network and hidden Markov models [43]. Excreted/secreted (ES) peptides were predicted based on the presence of a signal peptide and sequence homology to one or more known ES proteins listed in the Secreted Protein (http://spd.cbi.pku.edu.cn/; [44]) and the Signal Peptide (http://proline.bic.nus.edu.sg/spdb/index.html; [45]) databases.

## Results

**Figure 1:**
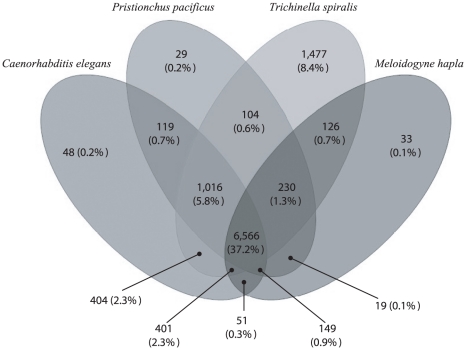
Similarity searches. Venn diagram illustrating the overlap in amino acid sequence homology between and among *Trichuris suis* and selected species of the class Enoplea (i.e., *Trichinella spiralis*) and Chromadorea (i.e., *Caenorhabditis elegans*, *Pristionchus pacificus* and *Meloidogyne hapla*).

A total number of 64,496,406 Illumina reads were produced for the adult stage of *T. suis* (Table 1). The assembly of the raw reads yielded 19,823 contiguous sequences ( = contigs) (1,480±1577 bases in length; range: 101–20,908) with a GC content of 44.22±5.3% (Table 1). A total of 39,659,580 reads (∼61.5% of all raw reads) could be re-mapped to the assembled contigs, with a mean depth of coverage of 108.3±288 reads per sequence. A total of 9,661 and 13,822 of 19,823 assembled contigs (49% and 70%, respectively) matched known *C. elegans* and *T. spiralis* homologues (e-value cut-off: <10^−5^), respectively, whereas 9,749 (49.1%) and 8,161 (46.2%) had homologues in *H. sapiens* and *S. scrofa*, respectively (Table 1); 15,553 (78%) and 9,214 (46%) nucleotide sequences of *T. suis* had homologues in other parasitic nematodes and other eukaryotic organisms, respectively (see Table 1). In total, 17,646 peptides were predicted from the transcriptome of *T. suis* (Table 1); 10,324 of them (58.5%) had highest homology to proteins predicted for *T. spiralis* (cf. [46]), followed by those from *C. elegans* (n = 8,754; 49.6%), *M. hapla* (n = 7,575; 49.9%) and *P. pacificus* (n = 8,232; 46.6%) (Figure 1).

**Table 1:** Summary of the nucleotide sequence data for adult *Trichuris suis* prior and following assembly, as well as detailed bioinformatic annotation and analyses.

*Raw reads (paired-end)*	64,496,406
Contigs (average length ± SD; min–max length)	19,823 (1480±1577.22; 101–20,908)
GC content (%)	44.22
Raw reads mapped to contigs (%)	39,659,580 (61.5)
Containing an Open Reading frame (%)	17,646 (90)
With homologues in *Caenorhabditis elegans* (%)	8,353 (47)
*Trichinella spiralis*	10,324 (58.5)
*Homo sapiens*	9,749 (49.1)
*Sus scrofa*	8,161 (46.2)
other parasitic nematodes (%)	15,533 (78)
other organisms (%)	9,214 (46)
Returning InterProScan results (%)	12,113 (68.6)
*Number of InterPro terms*	3,534
Gene Ontology (%)	10,248 (58)
*Number of Biological process terms (level 5)*	316
*Cellular component*	104
*Molecular function*	457
Returning a KOBAS result (%)	4,588 (26)
Number of predicted biological pathways (KEGG)	262
Predicted proteins with signal peptides (%)	1,992 (11.2)
with transmembrane domains (%)	3,004 (17)
Homologous to proteins in the SPD database (%)	5,548 (31.4)
Predicted excretory/secretory proteins*	1,288 (7.3)

*Inferred based on the presence of a signal peptide and homology to known proteins in the SPD database.

**Figure 2:**
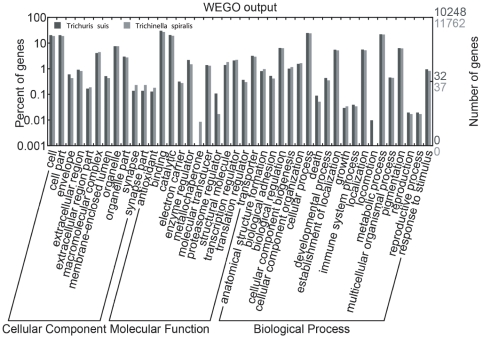
Gene Ontology. Bar graph illustrating similarities and differences between Gene Ontology (GO) terms (according to the categories ‘cellular component’ and ‘molecular function’ and ‘biological process’) assigned to peptides from *Trichuris suis* and *Trichinella spiralis* inferred from transcriptomic and genomic data, respectively, plotted using a web-based tool, WEGO [41].

Proteins inferred from the *T. suis* transcriptome were then categorized according to the presence of conserved (Pfam and/or InterPro) domains/signatures; 12,113 (68.6%) sequences could be mapped to known proteins characterised by 3,534 different conserved domains (Table 1). Predicted proteins were also classified according to their GO (based on their inferred molecular function, cellular localization and association with biological pathways) and compared with those encoded in the genome of the related nematode, *T. spiralis*. Of 10,248 proteins predicted for *T. suis*, 58% could be assigned to 316 ‘biological process’, 104 ‘cellular component’ and 457 ‘molecular function’ parental (level 2) terms (Table 1). The predominant terms were ‘metabolic process’ and ‘cellular process’ for ‘biological process’ (17.1% and 16.8%, respectively), ‘cell’ and ‘cell part’ for ‘cellular component’ (15.3% and 13.7%, respectively) and ‘binding activity’ and ‘catalytic activity’ for ‘molecular function’ (22.4% and 16.8%, respectively) (Figure 2). The GO profiles were similar between *T. suis* and *T. spiralis*, with only one ‘biological process’ term (namely ‘locomotion’) and one ‘molecular function’ term (i.e., ‘metallo-chaperone ativity’) being uniquely assigned to the *T. suis* and *T. spiralis* datasets, respectively (Figure 2). From the proteome predicted for *T. suis*, 4,588 (26%) proteins mapped to homologous proteins in the KEGG database, which were assigned to 262 biological pathway terms (Table 1), including ‘oxidative phosphorylation’ (ko00190; n = 76 predicted proteins), ‘ribosome’ (ko03010; n = 73) and ‘Huntington disease’ (ko05016; n = 36) (Figure S1). Interestingly, 120 proteins could be mapped to pathways associated with the immune system, including ‘chemokine signalling pathway’ (ko04062; n = 26), ‘leukocyte transendothelial migration’ (ko04670; n = 34), ‘T-cell receptor signalling pathway’ (ko04560; n = 22), ‘TGF-β signalling pathway’ (ko04350; n = 26) and ‘natural killer cell mediated cytotoxicity’ (ko04650; n = 12) (Figure S1). Putative proteins involved in reproductive processes (i.e., vitellogenins and chitin-binding proteins), structural proteins (collagens) and molecules involved in the metabolism of sugars (lectins) were most abundant (Table S1), in accordance with previous studies of the transcriptomes of other gastrointestinal nematodes, including the ‘barber's pole worm’ of small ruminants, *Haemonchus contortus* [47], the ascaridoid of swine, *Ascaris suum* [48], and the bovine lungworm, *Dictyocaulus viviparus* [49].

A total of 1,288 (7.3%) ES proteins were inferred from the transcriptome of *T. suis* (Tables 1 and 2), 22% of which were inferred to be proteases (n = 281), including serine proteases (n = 5; 0.4%) with identity (92%) to known ‘secretory leukocyte protease inhibitors’ (SLPIs) from *Mus musculus* (Table 2), uncharacterised proteins (n = 200; 16%) and hypothetical proteins (n = 143; 11%) (Table 2). Eighteen (∼1%) predicted ES proteins matched known pore-forming proteins from the human whipworm, *T. trichiura* [50] (see Table 2).

**Table 2:** Composition of putative excreted/secreted proteins inferred from the transcriptome of adult *Trichuris suis* and corresponding (conserved) signature protein motifs.

Predicted proteins	Number (%)	Pfam domain(s) (code)/number of putative peptides
*Proteases*	**281 (21.8)**	
Serine proteases*	42	Serine carboxypeptidase (PF05577.5);
	29	Rhomboid serine protease (PF12595.1);
	27	Serine proteinase (PF01972.9)
Metalloproteases	38	Zinc metalloprotease (PF01421.12);
	22	Metallopeptidase family (PF00557.17);
	18	Aminopeptidase I zinc metalloprotease (PF02127.8)
Cysteine proteinase	32	Cysteine protease (PF00548.13)
	12	Calpain family cystein protease (PF00648.14)
Aspartyl proteases	6	Eukaryotic aspartyl protease (PF00026.16);
	4	Aspartyl protease (PF09668.3);
	2	Retroviral aspartyl protease (PF00077.13)
Other peptidases	41	Peptidase family (PF03416.12; PF01431.14; PF01435.11);
	8	Trypsin (PF00089.19)
*Uncharacterised proteins*	**200 (15.5)**	
	161	Uncharacterised protein families (PF03665.6; PF03650.6)/161;
	39	Uncharacterised, conserved proteins (PF09758.2; PF10103.2; PF10171.2; PF10217.2; PF10225.2; PF10229.2; PF10164.2; PF10226.2)
*Protein kinases*	**143 (11.1)**	
Hexokinases	27	Hexokinase (PF00349.14)
Serine/threonine kinases	17	Serine/threonine protein kinase (PF05445.4)
Acylglycerol-kinases	9	Dyacylglycerol kinase (PF00609.12; PF00781.17)
Other kinases	24	Adenylate kinase (PF00406.15);
	19	Protein kinase domain (PF00069.18);
	19	Phosphofructokinase (PF00365.13);
	16	Pyruvate kinase (PF02887.9);
	5	Calcium-calmodulin dependent protein kinase (PF08332.3);
	5	Fructosamine kinase (PF03881.7);
	2	Deoxynucleoside kinase (PF01712.12)
*Receptor and channels*	**139 (10.8)**	
Lipid receptors	7	Low-density lipoprotein receptor (PF00057.11; PF00058.10)
Other receptors	41	A-macroglobulin receptor (PF07677.7);
	30	B cell receptor associated (PF05529.5);
	22	Transient receptor ion channel (PF08344.4);
	13	Ephrin receptor ligand binding (PF01404.12);
	11	Inositol triphosphate/ryanodine receptor (PF08709.4);
	8	G protein-coupled receptor (PF12205.1);
	3	Natural killer receptor (PF11465.1)
*Hypothetical proteins*	**143 (11.1)**	
	133	Conserved hypothetical protein (PF03602.8);
	8	Conserved hypothetical ATP-binding protein (PF03029.10);
	2	Hypothetical methyltransferase (PF05148.8)
*Transcription factors*	**132 (10.2)**	
	60	Transcription elongation factor (PF05129.6);
	51	Transcription factor (PF01096.11; PF00382.12; PF00352.14)
	21	CP2 transcription factor (PF04516.8)
*Protease inhibitors*	**86 (6.7)**	
Kunitz-type protease inhibitors	45	Kunitz-type protease inhibitor domain (PF00014.16)
Serine-protease inhibitors	11	Serine protease inhibitor domain (PF00050.14);
	2	Serpin (PF00079.13)
Other protease inhibitors	28	Cathepsin inhibitor domain (PF08246.5)
*Pore-forming proteins*	**18 (1.4)**	Eukaryotic porin (PF01459.15)
*Other enzymes*	**146 (11.3)**	
	102	Helicase conserved domain (PF00271.24);
	39	ATPase family (PF00004.22);
	5	Macrophage migration inhibitory factor (PF01187.11)

*Including n = 5 serine proteases with identity (92%) to known ‘secretory leukocyte protease inhibitors’ from *Mus musculus*.

## Discussion

The *T. suis* transcriptome characterised here represents the first and largest molecular dataset for any intestinal enoplid nematode. Previously, ∼7,000 unannotated expressed sequence tags (ESTs) from *T. muris* were available in public databases (GenBank accession nos. AW288312.1-FF146308.1). For *T. suis*, ∼47% of predicted proteins had homologues in *C. elegans*, which is in accordance with percentages (38–52%) for strongylid nematodes, including *D. viviparus*, *H. contortus*, *Trichostrongylus colubriformis* and *Oesophagostomum dentatum*) [25], [49], [51], [52], and refutes the hypothesis that *Trichuris* spp. have significantly less homologues/orthologues in *C. elegans* than strongylid nematodes [53]. As expected based on relationships inferred by phylogenetic analysis of nuclear ribosomal DNA sequence data [54], *T. suis* shares more homologues (58.5%) with *T. spiralis* than the other nematodes considered herein. Although *T. suis* and *T. spiralis* appear to encode a similar number of proteins (∼16,000–17,000; Table 1; [36]), all orphan molecules (i.e., with no homologues in any current database and representing 42 and 45% of all proteins encoded in *T. suis* and *T. spiralis*, respectively) were always exclusive to each of these two enoplids, reflecting their biological uniqueness [55].

Approximately one third of peptides inferred from the transcriptome of the adult stage of *T. suis* mapped to biological pathways linked to oxidative phosphorylation and ribosome biogenesis, and Huntington disease (HD) in humans, the latter of which was particularly interesting. HD is an age-dependent, progressive genetic disorder that affects the neuro-musculature system, leading to cognitive decline and dementia [56]. This disease is caused by an expansion of a poly-glutamine repeat in the protein huntingtin, resulting in the degeneration of motoneurons [57]. Although the cellular and molecular processes that lead to this neurodegenerative disorder during ageing are still unclear [58], the fork-head transcription factor *daf-16* has been suggested to play a central role [59], [60]. In *C. elegans*, ageing relates to the insulin-signalling pathway *via* the activity of *daf-16* [56], [61], [62]. A *daf-16* homologue/orthologue is also encoded in *T. suis* (see Figure S1). Although not yet studied for most parasitic nematodes, *daf-16* appears to regulate the transition to parasitism in other gastrointestinal nematodes, such as *Strongyloides stercoralis* (i.e., *Ss-daf-16*; [63]), *Ancylostoma caninum* [64], [65] and *H. contortus* (i.e., *Hc-daf-16.1* and *Hc-daf-16.2*; [66]). As the biology and development of this enoplid nematode (*T. suis*) [67], [68] is very different from other intestinal nematodes of animals studied to date, there is major merit in undertaking comparative functional genomic, transcriptomic and proteomic investigations to establish whether *daf-16* plays a similar role in the development of *T. suis* and its transition to parasitism. The approach of genetic complementation in *C. elegans*, as conducted recently by Hu and co-workers [66], could be used to assist this research endeavour.

A particularly important group of molecules inferred for *T. suis* and recognised as integral in the parasite-host interplay [69]–[73] is the ES proteins (see Table 2). Proteases were particularly well represented in *T. suis*, and are likely to facilitate host invasion and establishment, and contribute to the pathogenesis of disease [74], [75]. Indeed, chronic dysentery caused by heavy infections with some species of *Trichuris* has been linked to the disruption of the integrity of epithelial cell membranes caused by proteases excreted/secreted by adult worms and, consequently, to the leakage of plasma proteins across the mucosal surface of the intestinal epithelium [76], [77]. In addition, proteases secreted by cells (stichocytes) of the anterior end ( = stichosome) of *Trichuris* are likely to be involved in the establishment of the worms in the caecum of mammalian hosts [77], [78], for example, by inducing the formation of a syncytial tunnel derived from the host's caecal epithelium embedding the stichosome [50]. Interestingly, a proteomic analysis of *T. muris* ES products led to the identification of two major proteases (designated M_r_ 85 and M_r_ 105; [77]), which were shown to degrade type I collagen (but not casein, haemoglobin, fibrinogen or albumin) [77]. This finding, together with the observation that adult *T. muris* (murine whipworm) degrade basement membrane proteins from the endothelial cells of rat [77], suggest that ES proteases are actively involved in the formation and maintenance of the syncytium (cf. [77]). Furthermore, the ES proteins inferred for *T. suis* include pore-forming protein homologues. Experimental evidence [50] shows that ‘pore-forming proteins’ from both *T. trichiura* and *T. muris* induce ion-conducting pores in planar lipid bilayers, indicating that these proteins are involved in the formation of the syncytial structure [50]. In murine models, IgG serum antibodies raised against a pore-forming protein (called TM43) from *T. muris* were shown to inhibit the activity of this protein, leading to the rapid expulsion of the parasite from the host [79]. This information, together with the observation that TM43 shares a high degree of structural similarity with a pore-forming protein (TT47) from *T. trichiura* [50], suggests that this and similar molecules might represent effective immunogens. Clearly, future work should focus on investigating the stichosome and the parasite-host interface at the molecular level using advanced genomic and immuno-proteomic tools.

Other ES proteins inferred for *T. suis* are likely to play key roles in host immune responses and/or the modulation thereof. Of particular note are homologues of the secretory leukocyte protease inhibitors (SLPIs). These molecules are naturally occurring serine proteases, which are constitutively expressed by immune cells, including monocytes, macrophages, dendritic cells and neutrophils, in the mucosal tissues of the airways and secretions, such as bronchial and seminal fluids, saliva and breast milk [80]–[82], exhibit anti-inflammatory, anti-fungal [83] and anti-microbial activities [84]. Originally, an SLPI was found on mucosal surfaces, where it has a key role in protecting the epithelium from damage during inflammation processes (e.g., by degrading proteolytic enzymes released by neutrophils at the site of inflammation and by reducing the pro-inflammatory effect of bacterial lipopolysaccharides [85], [86]. High expression levels of SLPIs have been observed in patients affected by allergic asthma [87], [88]. More recently, the role of SLPIs in the pathogenesis of asthma has been elucidated in a study, in which sensitized, SLPI-deficient mice were treated with resiquimod, an immune response-modifier from the family of imidazoquinolinamines, known to inhibit allergen-induced Th2 responses [89], [90]. The results showed that SLPI-deficient mice exhibited airway eosinophilia, hyperplasia of goblet cells and high plasmatic IgE levels and decreased resistance of the lungs to allergens, when compared with wild-type mice [90]. In addition, the treatment with the compound resiquimod was effective in reducing the inflammatory hyper-responsiveness in the airways of SLPI-deficient mice, thus supporting the hypothesis that the treatment effect was independent of SLPI expression [90]. Recombinant SLPI administered by aerosol for 4 days (3 mg/day) has been shown experimentally to reduce airway hyper-responsiveness in sheep sensitized to *A. suum* antigens and “challenged” with carbacol [91]. Based on the results of these studies [90], [91], the administration of recombinant SLPI was proposed to represent a promising strategy to treat asthma. The relationship between SLPIs and the ability of *T. suis* to suppress the symptoms of allergic diseases remains to be explored. Future studies could investigate, for instance, the effect/s of the inoculation of recombinant *T. suis* SLPI homologues in non-*I*mmortalized *P*orcine *E*ndothelial *C*ell ( = IPEC) lines and/or in SLPI-deficient mice.

**Figure 3:**
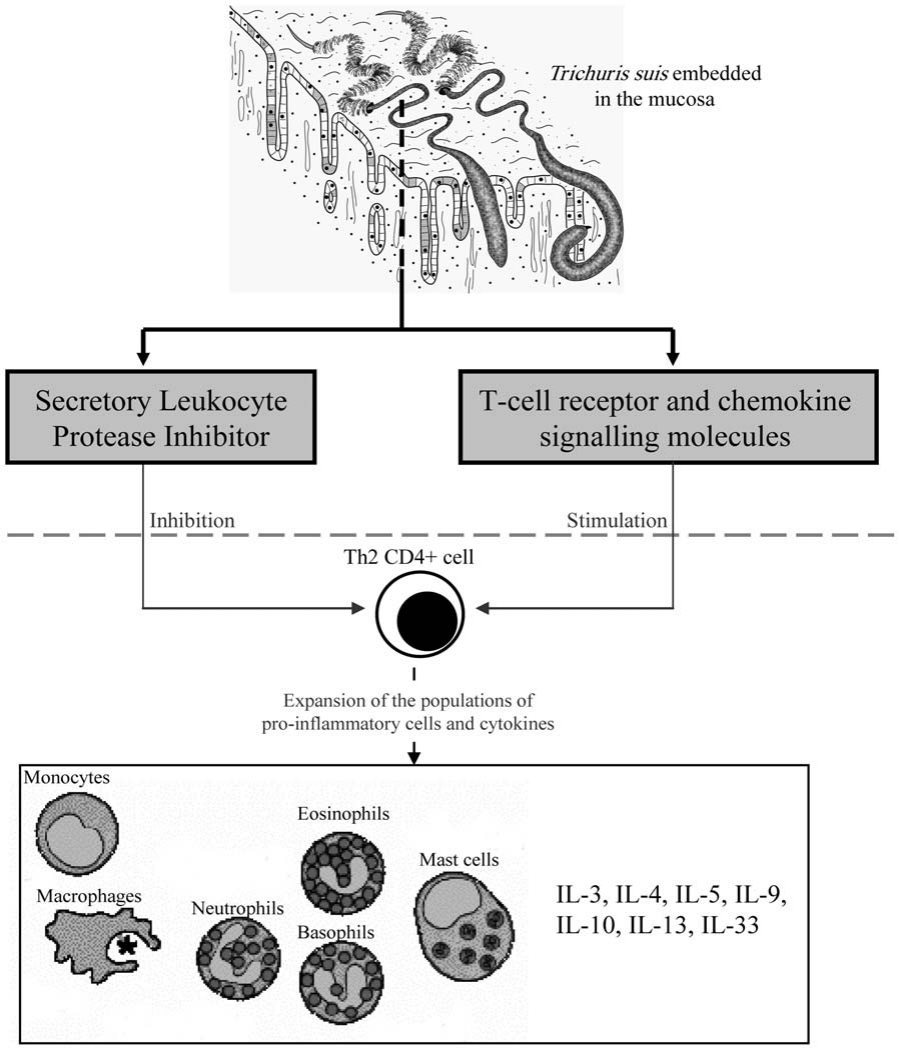
Immuno-modulatory mechanisms. Cascade of immuno-molecular events hypothesized to lead to the modulation of immune response in *Trichuris suis*-infected humans. Molecules linked to the T-cell receptor and chemokine signalling pathways could be involved in the stimulation of the Th2-type immune response, which, in turn, stimulates the expansion of populations of pro-inflammatory cells and cytokines. *T. suis* homologues of secreted leukocyte protease inhibitors (SLPIs) could inhibit hyper-responsiveness of the immune system by down-regulating the activity of Th2-type cells.

A range of other *T. suis* molecules (n = 120) were inferred to be associated with the chemokine, T-cell receptor and TGF-β signalling pathways as well as with leukocyte transendothelial migration and/or natural killer cell mediated cytotoxicity (Figure S1). Although their significance or roles remain to be established, they are likely to be involved in regulating/modulating the Th2-biased immune response, characteristic of *T. suis* infection in its host/s [92], [93]. A detailed understanding of these molecules might assist in designing new treatment strategies for immunological disorders in humans. Interestingly, there is evidence that iatrogenic *T. suis* infection is safe and effective for the treatment of immune-mediated diseases, including IBD [14], [22], [94], Crohn's Disease (CD; [17], [21]), allergic rhinitis [20] and, more recently, multiple sclerosis [95]. Indeed multiple studies have demonstrated that the administration of live *T. suis* ova is consistently associated with an increase in the levels of IL-4 and IL-10 and other Th2-cytokines; the release of these cytokines has been reported to result in a remission of clinical symptoms of allergic diseases [17], [19], [95]. However, despite these observations, the molecular mechanisms by which *T. suis* modulates the host's immune response remain to be explored. One hypothesis is that the *T. suis* molecules associated with the T-cell receptor and chemokine signalling pathways might be involved in the stimulation of the adaptive Th2-type immune response, typical of a vast range of helminth infections [73], [96]. This type of immune response involves the release of the IL-3, IL-4, IL-5, IL-9, IL-10, IL-13 and IL-33 cytokines, and the expansion of populations of eosinophils, basophils, mast cells and macrophages [73], [97]–[99]. The *T. suis* molecules associated with the TGF-β signalling pathway might induce the release of Th2-type cytokines with anti-inflammatory and wound-healing functions [100], whereas the SLPI homologues could contribute to the modulation of the immune system of the mammalian host by inhibiting hyper-responsiveness of the Th2 cell population [90] (Figure 3). This cascade of events might indirectly lead to the reduction of symptoms linked to allergic disorders. Clearly, elucidating the mechanisms responsible for the remission of the clinical symptoms linked to immunopathological diseases in patients infected with *T. suis* should ultimately provide a sound foundation for the development of novel approaches for the treatment of such diseases.

In conclusion, the transcriptome (and inferred proteome) characterised in the present study will assist in future efforts to decode the entire genome of *T. suis*. The availability of this genome will underpin proteomic studies, aimed at elucidating the molecular biology of the parasite as well as host-parasite interactions, with a view toward improving our knowledge and understanding of the mechanisms by which *T. suis* modulates the host's immune response(s) and suppresses clinical symptoms in IBD patients. Profound insights into these areas could facilitate the identification and development of entirely novel strategies for the treatment of key immune diseases of humans.

## Supporting Information

Figure S1 **Biological pathways.** Biological pathway mapping of proteins (red boxes) inferred from the transcriptome of the adult stage of *Trichuris suis* mapped to pathways based on homology (BLASTp, *E*-value≤1e^−15^) to KEGG orthology terms. Grey boxes represent proteins for which no *T. suis* homologues could be mapped.
(PDF)

Table S1 The twenty most abundant proteins encoded in the transcriptome of *Trichuris suis*, following conceptual translation of individual contigs.
(DOC)

## References

[1] de Silva NR, Brooker S, Hotez PJ, Montresor A, Engels D (2003). Soil-transmitted helminth infections: updating the global picture.. Trends Parasitol.

[2] Harhay MO, Horton J, Olliaro PL (2010). Epidemiology and control of human gastrointestinal parasites in children.. Expert Rev Anti Infect Ther.

[3] Hotez PJ, Fenwick A, Savioli L, Molyneux DH (2009). Rescuing the bottom billion through control of neglected tropical diseases.. Lancet.

[4] Chanco PP, Vidad JY (1978). A review of trichuriasis, its incidence, pathogenicity and treatment.. Drugs.

[5] Bundy DA, Cooper ES (1989). *Trichuris* and trichuriasis in humans.. Adv Parasitol.

[6] Stephenson LS, Holland CV, Cooper ES (2000). The public health significance of *Trichuris trichiura*.. Parasitology.

[7] Okada H, Kuhn C, Feillet H, Bach JF (2010). The ‘hygiene hypothesis’ for autoimmune and allergic diseases: an update.. Clin Exp Immunol.

[8] ISAAC (1998). Worldwide variation in prevalence of symptoms of asthma, allergic rhinoconjunctivitis, and atopic eczema: ISAAC. The International Study of Asthma and Allergies in Childhood (ISAAC) Steering Committee.. Lancet.

[9] Bach JF (2002). The effect of infections on susceptibility to autoimmune and allergic diseases.. N Engl J Med.

[10] Braman SS (2006). The global burden of asthma.. Chest.

[11] Eder W, Ege MJ, von Mutius E (2008). The asthma epidemic.. N Engl J Med.

[12] Yazdanbakhsh M, Matricardi PM (2004). Parasites and the hygiene hypothesis. Regulating the immune system?. Clin Rev Allergy Immunol.

[13] McGeady SJ (2004). Immunocompetence and allergy.. Pediatrics.

[14] Erb KJ (2008). Can helminths or helminth-derived products be used in humans to prevent or treat allergic diseases?. Trends Immunol.

[15] Yang X, Gao X (2011). Role of dendritic cells: a step forward for the hygiene hypothesis.. Cell Mol Immunol.

[16] Summers RW, Elliott DE, Weinstock JV (2005). Is there a role for helminths in the therapy of inflammatory bowel disease?. Nat Clin Pract Gastroenterol Hepatol.

[17] Summers RW, Elliott DE, Qadir K, Urban JF, Thompson R (2005). *Trichuris suis* therapy in Crohn's disease.. Gut.

[18] Summers RW, Elliott DE, Weinstock JV (2005). Why *Trichuris suis* should prove safe for use in inflammatory bowel disease.. Inflamm Bowel Dis.

[19] Bager P, Arnved J, Ronborg S, Wohlfahrt J, Poulsen LK (2010). *Trichuris suis* ova therapy for allergic rhinitis: a randomized, double-blind, placebo-controlled clinical trial.. J Allergy Clin Immunol.

[20] Hepworth MR, Hamelmann E, Lucius R, Hartmann S (2010). Looking into the future of *Trichuris suis* therapy.. J Allergy Clin Immunol.

[21] Reddy A, Fried B (2007). The use of *Trichuris suis* and other helminth therapies to treat Crohn's disease.. Parasitol Res.

[22] Hunter MM, McKay DM (2004). Helminths as therapeutic agents for inflammatory bowel disease.. Aliment Pharmacol Ther.

[23] Figuereido CA, Barreto ML, Rodrigues LC, Cooper PJ, Silva NB (2010). Chronic intestinal helminth infections are associated with immune hyporesponsiveness and induction of a regulatory network.. Infect Immun.

[24] Bentley DR, Balasubramanian S, Swerdlow HP, Bentley DR, Swerdlow HP (2008). Accurate whole human genome sequencing using reversible terminator chemistry.. Nature.

[25] Cantacessi C, Jex AR, Hall RS, Young ND, Campbell BE (2010). A practical, bioinformatic workflow system for large data sets generated by next-generation sequencing.. Nucleic Acids Res.

[26] Hill DE, Gamble HR, Rhoads ML, Fetterer RH, Urban JF (1993). *Trichuris suis*: a zinc metalloprotease from culture fluids of adult parasites.. Exp Parasitol.

[27] Young ND, Jex AR, Cantacessi C, Hall RS, Campbell BE (2011). A portrait of the transcriptome of the neglected trematode, *Fasciola gigantica*—Biological and biotechnological implications.. PLoS Negl Trop Dis.

[28] Zerbino DR, Birney E (2008). Velvet: algorithms for de novo short read assembly using de Bruijn graphs.. Genome Res.

[29] Li R, Yu C, Li Y, Lam TW, Yiu SM (2009). SOAP2: an improved ultrafast tool for short read alignment.. Bioinformatics.

[30] Mortazavi A, Williams BA, McCue K, Schaeffer L, Wold B (2008). Mapping and quantifying mammalian transcriptomes by RNA-seq.. Nat Methods.

[31] Altschul SF, Madden TL, Schäffer AA, Zhang J, Zhang Z (1997). Gapped BLAST and PSI-BLAST: a new generation of protein database search programs.. Nucleic Acids Res.

[32] Iseli C, Jongeneel CV, Bucher P (1999). ESTScan: a program for detecting, evaluating, and reconstructing potential coding regions in EST sequences.. Proc Int Conf Intell Syst Mol Biol.

[33] Harris TW, Stein LD (2006). WormBase: methods for data mining and comparative genomics.. Methods Mol Biol.

[34] Harris TW, Antoshechkin I, Bieri T, Blasiar D, Chan J (2010). WormBase: a comprehensive resource for nematode research.. Nucleic Acids Res.

[35] Dieterich C, Clifton SW, Schuster LN, Chinwalla A, Delehaunty I (2008). The *Pristionchus pacificus* genome provides a unique perspective on nematode lifestyle and parasitism.. Nat Genet.

[36] Mitreva M, Jasmer DP, Zarlenga DS, Wang Z, Abubucker S (2011). The draft genome of the parasitic nematode *Trichinella spiralis*.. Nat Genet.

[37] Mbeunkui S, Scholl EH, Opperman CH, Goshe MB, Bird DM (2010). Proteomic and bioinformatic analysis of the root-knot nematode *Meloidogyne hapla*: the basis for plant parasitism.. J Proteome Res.

[38] Johnson LS, Eddy SR, Portugaly E (2010). Hidden Markov model speed heuristic and iterative HMM procedure.. BMC Bioinformatics.

[39] Hunter S, Apweiler R, Attwood TK, Bairoch A, Bateman A (2009). InterPro: the integrative protein signature database.. Nucleic Acids Res.

[40] Ashburner M, Ball CA, Blake JA, Botstein D, Butler H (2000). Gene ontology: tool for the unification of biology. The Gene Ontology Consortium.. Nat Genet.

[41] Ye J, Fang L, Zheng H, Zhang Y, Chen J (2006). WEGO: a web tool for plotting GO annotations.. Nucleic Acids Res.

[42] Wu J, Mao X, Cai T, Luo J, Wei L (2006). KOBAS server: a web-based platform for automated annotation and pathway identification.. Nucleic Acids Res.

[43] Bendtsen JD, Nielsen H, von Heijne G, Brunak S (2004). Improved prediction of signal peptides: SignalP 3.0.. J Mol Biol.

[44] Chen Y, Zhang Y, Yin Y, Gao G, Li S (2005). SPD—a web based secreted protein database.. Nucleic Acids Res.

[45] Choo KH, Tan TW, Ranganathan S (2005). SPdb – a signal peptide database.. BMC Bioinformatics.

[46] Blaxter ML, De Ley P, Garey JR, Liu LX, Scheldeman P (1998). A molecular evolutionary framework for the phylum Nematoda.. Nature.

[47] Campbell BE, Nagaraj SH, Hu M, Zhong W, Sternberg PW (2010). Gender-enriched transcripts in *Haemonchus contortus* – predicted functions and genetic interactions based on comparative analyses with *Caenorhabditis elegans*.. Int J Parasitol.

[48] Cantacessi C, Zou FC, Hall RS, Zhong W, Jex AR (2009). Bioinformatic analysis of abundant, gender-enriched transcripts of *Ascaris suum* (Nematoda) using a semi-automated workflow platform.. Mol Cell Probes.

[49] Cantacessi C, Gasser RB, Strube C, Schnieder T, Jex AR (2011). Deep insights into *Dictyocaulus viviparus* transcriptomes provides unique prospects for new drug targets and disease intervention.. Biotechnol Adv.

[50] Drake L, Korchev Y, Bashford L, Djamgoz M, Wakelin D (1994). The major secreted product of the whipworm, *Trichuris*, is a pore-forming protein.. Proc R Soc Lond B.

[51] Cantacessi C, Mitreva M, Campbell BE, Hall RS, Young ND (2010). First transcriptomic analysis of the economically important parasitic nematode, *Trichostrongylus colubriformis*, using a next-generation sequencing approach.. Infect Genetic Evol.

[52] Cantacessi C, Campbell BE, Young ND, Jex AR, Hall RS (2010). Differences in transcription between free-living and CO_2_-activated third-stage larvae of *Haemonchus contortus*.. BMC Genomics.

[53] Blaxter M (1998). *Caenorhabditis elegans* is a nematode.. Science.

[54] Van Megen H, Van Den Elsen S, Holterman M, Karssen G, Mooyman P (2009). A phylogenetic tree of nematodes based on about 1200 full-length small subunit ribosomal DNA sequences.. Nematology.

[55] Leder K, Schwartz E (2010). Chapter 31. Intestinal Helminths: *Strongyloides stercoralis*, *Ascaris lumbricoides*, Hookworm, *Trichuris trichiura*, *Enterobius vermicularis*, *Trichinella*, Intestinal Tapeworms, and Liver Flukes.. Tropical Diseases in Travelers.

[56] Ross CA, Tabrizi SJ (2011). Huntington's disease: from molecular pathogenesis to clinical treatment.. Lancet Neurol.

[57] HD Collaborative Group (1993). A novel gene containing a trinucleotide repeat that is expanded and unstable on Huntington's disease chromosomes.. Cell.

[58] Coppede F, Migliore L (2010). DNA repair in premature aging disorders and neurodegenration.. Curr Aging Sci.

[59] Lin K, Dorman JB, Rodan A, Kenyon C (1997). DAF-16: an HNF-3/forkhead family member that can function to double the life-span of *Caenorhabditis elegans*.. Science.

[60] Ogg S, Paradis S, Gottlieb S, Patterson GI, Lee L (1997). The Fork head transcription factor DAF-16 transduces insulin-like metabolic and longevity signals in *C. elegans*.. Nature.

[61] Libina N, Berman JR, Kenyon C (2003). Tissue-specific activities of *C. elegans* DAF-16 in the regulation of lifespan.. Cell.

[62] Voisine C, Varma H, Walker N, Bates EA, Stockwell BR (2007). Identification of potential therapeutic drugs for Huntington's disease using *Caenorhabditis elegans*.. PLoS One.

[63] Massey HC, Nishi M, Chaudhary K, Pakpour N, Lok JB (2003). Structure and development expression of *Strongyloides starcoralis fktf-1*, a proposed ortholog of *daf-16* in *Caenorhabditis elegans*.. Int J Parasitol.

[64] Datu BJD, Loukas A, Cantacessi C, O'Donoghue P, Gasser RB (2009). Investigation of the regulation of transcriptional changes in *Ancylostoma caninum* larvae following serum-activation, with a focus on the insulin-like signalling pathway.. Vet Parasitol.

[65] Gao X, Wang Z, Martin J, Abubucker S, Zhang X (2010). Identification of hookworm DAF-16/FOXO response elements and direct gene targets.. PLoS One.

[66] Hu M, Lok JB, Ranjit N, Massey HC, Sternberg PW (2010). Structural and functional characterisation of the fork-head transcription factor-encoding gene, *Hc-daf-16*, from the parasitic nematode *Haemonchus contortus*.. Int J Parasitol.

[67] Beer RJS (1973). Studies on the biology of the life-cycle of *Trichuris suis* Schrank, 1788.. Parasitology.

[68] Panesar TS, Croll NA (1980). The location of parasites within their hosts: site selection by *Trichuris muris* in the laboratory mouse.. Int J Parasitol.

[69] Grencis RK, Bancroft AJ (2004). Interleukin-13: a key mediator in resistance to gastrointestinal-dwelling nematode parasites.. Clin Rev Allergy Immunol.

[70] Hewitson JP, Grainger JR, Maizels RM (2009). Helminth immunoregulation: the role of parasite secreted proteins in modulating host immunity.. Mol Biochem Parasitol.

[71] Maizels RM (2009). Parasite immunomodulation and polymorphisms of the immune system.. J Biol.

[72] Maizels RM (2009). Exploring the immunology of parasitism – from surface antigens to the hygiene hypothesis.. Parasitology.

[73] Allen JE, Maizels RM (2011). Diversity and dialogue in immunity to helminths.. Nat Rev Immunol.

[74] Ramsey FC (1962). *Trichuris* dysentery syndrome.. West Indian Med J.

[75] Cooper ES, Bundy DAP (1988). *Trichuris* is not trivial.. Parasitol Today.

[76] Cooper ES, Whyte-Alleng CAM, Finzi-Smith JS, McDonald TT (1992). Intestinal nematode infections in children: the pathophysiological price paid.. Parasitology.

[77] Drake LJ, Bianco AE, Bundy DAP, Ashall F (1994). Characterization of peptidases of adult *Trichuris suis*.. Parasitology.

[78] Nimmo-Smith RH, Keeling JED (1960). Some hydrolytic enzymes of the parasitic nematode *Trichuris muris*.. Exp Parasitol.

[79] Wakelin D (1967). Acquired immunity to *Trichuris muris* in the albino laboratory mice.. Parasitology.

[80] Eisenberg SP, Hale KK, Heimdal P, Thompson RC (1990). Location of the protease-inhibitory region of secretory leukocyte protease inhibitor.. J Biol Chem.

[81] Odaka C, Mizuochi T, Yang J, Ding A (2003). Murine macrophages produce secretory leukocyte protease inhibitor during clearance of apoptotic cells: implications for resolution of the inflammatory response.. J Immunol.

[82] Fakioglu E, Wilson SS, Mesquita PM, Hazrati E, Cheshenko N (2008). Herpes simplex virus downregulates secretory leukocyte protease inhibitor: a novel immune evasion mechanism.. J Virol.

[83] Tomee JF, Hiemstra PS, Heinzel-Wieland R, Kauffman HF (1997). Antileukoprotease: an endogenous protein in the innate mucosal defense against fungi.. J Infect Dis.

[84] Sagel SD, Sontag MK, Accurso FJ (2009). Relationship between antimicrobial proteins and airway inflammation and infection in cystic fibrosis.. Pediatr Pulmonol.

[85] Fryksmark U, Ohlsson K, Rosengren M, Tegner H (1983). Studies on the interaction between leukocyte elastase, antileukoproteinase and plasma proteinase inhibitors alpha 1-proteinase inhibitor and alpha 2-macroglobulin.. Hoppe Seylers Z Physiol Chem.

[86] Ashcroft GS, Lei K, Jin W, Longenecker G, Lulkarni AB (2000). Secretory leukocyte protease inhibitor mediates non-redundant functions necessary for normal wound healing.. Nat Med.

[87] Wemzel SE, Fowler AA, Schwartz LB (1988). Activation of pulmonary mast cells by bronchoalveolar allergen challenge. *In vitro* release of histamine and tryptase in atopic subjects with and without asthma.. Am Rev Respir Dis.

[88] Fahy JV, Kim KW, Liu J, Boushey HA (1995). Prominent neutrophilic inflammation in sputum from patients with asthma exacerbation.. J Allergy Clin Immunol.

[89] Quarcoo D, Weixler S, Joachim RA, Stock P, Kallinich T (2004). Resiquimod, a new immune response modifier from the family of imidazoquinolinamines, inhibits allergen-induced Th2 responses, airway inflammation and airway hyper-reactivity in mice.. Clin Exp Allergy.

[90] Marino R, Thuraisingam T, Camateros P, Kanagaratham C, Xu YZ (2011). Secretory leukocyte protease inhibitor plays an important role in the regulation of allergic asthma in mice.. J Immunol.

[91] Wright CD, Havill AM, Middleton SC, Kashem MA, Lee PA (1999). Secretory leukocyte protease inhibitor prevents allergen-induced pulmonary responses in animal models of asthma.. J Pharmacol Exp Ther.

[92] Parthasarathy G, Mansfield LS (2005). *Trichuris suis* excretory secretory products (ESP) elicit interleukin-6 (IL-6) and IL-10 secretion from intestinal epithelial cells (IPEC-1).. Vet Parasitol.

[93] Kringel H, Iburg T, Dawson H, Aasted B, Roepstorff A (2006). A time course study of immunological responses in *Trichuris suis* infected pigs demonstrates induction of a local type 2 response associated with worm burden.. Int J Parasitol.

[94] Elliott DE, Urban JF, Argo CK, Weinstock JV (2000). Does the failure to acquire helminthic parasites predispose to Crohn's disease?. FASEB J.

[95] Fleming JO, Isaak A, Lee JE, Luzzio CC, Carrithers MD (2011). Probiotic helminth administration in relapsing-remitting multiple sclerosis: a phase I study.. Mult Scler.

[96] Koyasu S, Moro K (2011). Type 2 innate immune responses and the natural helper cell.. Immunology.

[97] Finkelman FD, Shea-Donohue T, Morris SC, Gildea L, Strait R (2004). Interleukin-4 and interleukin-13- mediated host protection against intestinal nematode parasites.. Immunol Rev.

[98] Anthony RM, Rutitzky LI, Urban JF, Stadecker MJ, Gause WC (2007). Protective immune mechanisms in helminth infection.. Nature Rev Immunol.

[99] Jenkins SJ, Allen JE (2010). Similarity and diversity in macrophage activation by nematodes, trematodes, and cestodes.. J Biomed Biotechnol.

[100] Eming SA, Krieg T, Davidson JM (2007). Inflammation in wound repair: molecular and cellular mechanisms.. J Invest Dermatol.

