# Supplementary material for: The Transcriptome of Trichuris suis – First Molecular Insights into a Parasite with Curative Properties for Key Immune Diseases of Humans
Source: PLoS One. 2011 Aug 24;6(8):e23590. doi: 10.1371/journal.pone.0023590 (PMC3160910; doi:10.1371/journal.pone.0023590)
Supplement: Figure S1 — Biological pathways. Biological pathway mapping of proteins (red boxes) inferred from the transcriptome of the adult stage of Trichuris suis mapped to pathways based on homology (BLASTp, E-value≤1e−15) to KEGG orthology terms. Grey boxes represent proteins for which no T. suis homologues could be mapped. (PDF) [file pone.0023590.s001.pdf]

KEGG pathways for *Trichuris suis*

March 24, 2011

List of Figures

1 ko01110 Biosynthesis of secondary metabolites . . . 2
2 ko05200 Pathways in cancer . . . 3
3 ko01120 Microbial metabolism in diverse environments . . . 4
4 ko05016 Huntington's disease . . . 5
5 ko00190 Oxidative phosphorylation . . . 6
6 ko03010 Ribosome . . . 7
7 ko05010 Alzheimer's disease . . . 8
8 ko05012 Parkinson's disease . . . 9
9 ko04010 MAPK signaling pathway . . . 10
10 ko00230 Purine metabolism . . . 11
11 ko04141 Protein processing in endoplasmic reticulum . . . 12
12 ko04110 Cell cycle . . . 13
13 ko00240 Pyrimidine metabolism . . . 14
14 ko04810 Regulation of actin cytoskeleton . . . 15
15 ko04120 Ubiquitin mediated proteolysis . . . 16
16 ko04310 Wnt signaling pathway . . . 17
17 ko04910 Insulin signaling pathway . . . 18
18 ko04510 Focal adhesion . . . 19
19 ko04111 Cell cycle - yeast . . . 20

20 ko04530 Tight junction . . . 21
21 ko03013 RNA transport . . . 22
22 ko04114 Oocyte meiosis . . . 23
23 ko04145 Phagosome . . . 24
24 ko04722 Neurotrophin signaling pathway . . . 25
25 ko04914 Progesterone-mediated oocyte maturation . . . 26
26 ko03050 Proteasome . . . 27
27 ko04113 Meiosis - yeast . . . 28
28 ko04916 Melanogenesis . . . 29
29 ko04520 Adherens junction . . . 30
30 ko04062 Chemokine signaling pathway . . . 31
31 ko04144 Endocytosis . . . 32
32 ko04020 Calcium signaling pathway . . . 33
33 ko05211 Renal cell carcinoma . . . 34
34 ko04360 Axon guidance . . . 35
35 ko04670 Leukocyte transendothelial migration . . . 36
36 ko00510 N-Glycan biosynthesis . . . 37
37 ko04666 Fc gamma R-mediated phagocytosis . . . 38
38 ko05215 Prostate cancer . . . 39
39 ko04012 ErbB signaling pathway . . . 40
40 ko05210 Colorectal cancer . . . 41
41 ko04080 Neuroactive ligand-receptor interaction . . . 42
42 ko04660 T cell receptor signaling pathway . . . 43
43 ko05100 Bacterial invasion of epithelial cells . . . 44
44 ko04350 TGF-beta signaling pathway . . . 45
45 ko05160 Hepatitis C . . . 46
46 ko04150 mTOR signaling pathway . . . 47
47 ko05120 Epithelial cell signaling in Helicobacter pylori infection . . . 48
48 ko05212 Pancreatic cancer . . . 49
49 ko00010 Glycolysis / Gluconeogenesis . . . 50
50 ko03030 DNA replication . . . 51

51 ko04070 Phosphatidylinositol signaling system . . . 52
52 ko00970 Aminoacyl-tRNA biosynthesis . . . 53
53 ko05110 Vibrio cholerae infection . . . 54
54 ko05220 Chronic myeloid leukemia . . . 55
55 ko00520 Amino sugar and nucleotide sugar metabolism . . . 56
56 ko00280 Valine, leucine and isoleucine degradation . . . 57
57 ko04912 GnRH signaling pathway . . . 58
58 ko05131 Shigellosis . . . 59
59 ko04142 Lysosome . . . 60
60 ko00020 Citrate cycle (TCA cycle) . . . 61
61 ko05213 Endometrial cancer . . . 62
62 ko03020 RNA polymerase . . . 63
63 ko05145 Toxoplasmosis . . . 64
64 ko04340 Hedgehog signaling pathway . . . 65
65 ko04270 Vascular smooth muscle contraction . . . 66
66 ko04920 Adipocytokine signaling pathway . . . 67
67 ko05142 Chagas disease (American trypanosomiasis) . . . 68
68 ko03420 Nucleotide excision repair . . . 69
69 ko05223 Non-small cell lung cancer . . . 70
70 ko00562 Inositol phosphate metabolism . . . 71
71 ko05130 Pathogenic Escherichia coli infection . . . 72
72 ko05217 Basal cell carcinoma . . . 73
73 ko00500 Starch and sucrose metabolism . . . 74
74 ko03022 Basal transcription factors . . . 75
75 ko04260 Cardiac muscle contraction . . . 76
76 ko05214 Glioma . . . 77
77 ko05146 Amoebiasis . . . 78
78 ko04380 Osteoclast differentiation . . . 79
79 ko05222 Small cell lung cancer . . . 80
80 ko04972 Pancreatic secretion . . . 81
81 ko00620 Pyruvate metabolism . . . 82

82 ko00564 Glycerophospholipid metabolism . . . 83
83 ko04664 Fc epsilon RI signaling pathway . . . 84
84 ko04330 Notch signaling pathway . . . 85
85 ko04370 VEGF signaling pathway . . . 86
86 ko04662 B cell receptor signaling pathway . . . 87
87 ko04720 Long-term potentiation . . . 88
88 ko04974 Protein digestion and absorption . . . 89
89 ko00640 Propanoate metabolism . . . 90
90 ko04540 Gap junction . . . 91
91 ko04730 Long-term depression . . . 92
92 ko00250 Alanine, aspartate and glutamate metabolism . . . 93
93 ko04962 Vasopressin-regulated water reabsorption . . . 94
94 ko00030 Pentose phosphate pathway . . . 95
95 ko00513 Various types of N-glycan biosynthesis . . . 96
96 ko00380 Tryptophan metabolism . . . 97
97 ko04612 Antigen processing and presentation . . . 98
98 ko00480 Glutathione metabolism . . . 99
99 ko00650 Butanoate metabolism . . . 100
100 ko03320 PPAR signaling pathway . . . 101
101 ko05323 Rheumatoid arthritis . . . 102
102 ko02010 ABC transporters . . . 103
103 ko00051 Fructose and mannose metabolism . . . 104
104 ko04630 Jak-STAT signaling pathway . . . 105
105 ko05221 Acute myeloid leukemia . . . 106
106 ko04976 Bile secretion . . . 107
107 ko03060 Protein export . . . 108
108 ko04650 Natural killer cell mediated cytotoxicity . . . 109
109 ko04115 p53 signaling pathway . . . 110
110 ko00563 Glycosylphosphatidylinositol(GPI)-anchor biosynthesis . . . 111
111 ko00071 Fatty acid metabolism . . . 112
112 ko00330 Arginine and proline metabolism . . . 113

113 ko00680 Methane metabolism . . . 114
114 ko05410 Hypertrophic cardiomyopathy (HCM) . . . 115
115 ko04620 Toll-like receptor signaling pathway . . . 116
116 ko05218 Melanoma . . . 117
117 ko04130 SNARE interactions in vesicular transport . . . 118
118 ko03410 Base excision repair . . . 119
119 ko04971 Gastric acid secretion . . . 120
120 ko00710 Carbon fixation in photosynthetic organisms . . . 121
121 ko05412 Arrhythmogenic right ventricular cardiomyopathy (ARVC) . . . 122
122 ko00270 Cysteine and methionine metabolism . . . 123
123 ko04210 Apoptosis . . . 124
124 ko03008 Ribosome biogenesis in eukaryotes . . . 125
125 ko00627 Aminobenzoate degradation . . . 126
126 ko00350 Tyrosine metabolism . . . 127
127 ko04320 Dorso-ventral axis formation . . . 128
128 ko05414 Dilated cardiomyopathy . . . 129
129 ko05014 Amyotrophic lateral sclerosis (ALS) . . . 130
130 ko03430 Mismatch repair . . . 131
131 ko00052 Galactose metabolism . . . 132
132 ko04140 Regulation of autophagy . . . 133
133 ko04930 Type II diabetes mellitus . . . 134
134 ko05216 Thyroid cancer . . . 135
135 ko04512 ECM-receptor interaction . . . 136
136 ko04970 Salivary secretion . . . 137
137 ko00720 Carbon fixation pathways in prokaryotes . . . 138
138 ko00260 Glycine, serine and threonine metabolism . . . 139
139 ko03018 RNA degradation . . . 140
140 ko00670 One carbon pool by folate . . . 141
141 ko00534 Glycosaminoglycan biosynthesis - heparan sulfate . . . 142
142 ko05416 Viral myocarditis . . . 143
143 ko05219 Bladder cancer . . . 144

144 ko00910 Nitrogen metabolism . . . 145
145 ko00561 Glycerolipid metabolism . . . 146
146 ko04966 Collecting duct acid secretion . . . 147
147 ko04745 Phototransduction - fly . . . 148
148 ko04960 Aldosterone-regulated sodium reabsorption . . . 149
149 ko04973 Carbohydrate digestion and absorption . . . 150
150 ko03015 mRNA surveillance pathway . . . 151
151 ko00040 Pentose and glucuronate interconversions . . . 152
152 ko00310 Lysine degradation . . . 153
153 ko04013 MAPK signaling pathway - fly . . . 154
154 ko00983 Drug metabolism - other enzymes . . . 155
155 ko03440 Homologous recombination . . . 156
156 ko00600 Sphingolipid metabolism . . . 157
157 ko05020 Prion diseases . . . 158
158 ko04514 Cell adhesion molecules (CAMs) . . . 159
159 ko04710 Circadian rhythm - mammal . . . 160
160 ko04060 Cytokine-cytokine receptor interaction . . . 161
161 ko00630 Glyoxylate and dicarboxylate metabolism . . . 162
162 ko00410 beta-Alanine metabolism . . . 163
163 ko00790 Folate biosynthesis . . . 164
164 ko00514 Other types of O-glycan biosynthesis . . . 165
165 ko04610 Complement and coagulation cascades . . . 166
166 ko00360 Phenylalanine metabolism . . . 167
167 ko04011 MAPK signaling pathway - yeast . . . 168
168 ko00900 Terpenoid backbone biosynthesis . . . 169
169 ko04622 RIG-I-like receptor signaling pathway . . . 170
170 ko00290 Valine, leucine and isoleucine biosynthesis . . . 171
171 ko00860 Porphyrin and chlorophyll metabolism . . . 172
172 ko04626 Plant-pathogen interaction . . . 173
173 ko00980 Metabolism of xenobiotics by cytochrome P450 . . . 174
174 ko00760 Nicotinate and nicotinamide metabolism . . . 175

175 ko00450 Selenocompound metabolism . . . 176
176 ko04623 Cytosolic DNA-sensing pathway . . . 177
177 ko00362 Benzoate degradation . . . 178
178 ko00062 Fatty acid elongation in mitochondria . . . 179
179 ko00903 Limonene and pinene degradation . . . 180
180 ko04146 Peroxisome . . . 181
181 ko04621 NOD-like receptor signaling pathway . . . 182
182 ko00982 Drug metabolism - cytochrome P450 . . . 183
183 ko02020 Two-component system . . . 184
184 ko00130 Ubiquinone and other terpenoid-quinone biosynthesis . . . 185
185 ko00565 Ether lipid metabolism . . . 186
186 ko04740 Olfactory transduction . . . 187
187 ko05143 African trypanosomiasis . . . 188
188 ko03040 Spliceosome . . . 189
189 ko05140 Leishmaniasis . . . 190
190 ko04964 Proximal tubule bicarbonate reclamation . . . 191
191 ko04614 Renin-angiotensin system . . . 192
192 ko03450 Non-homologous end-joining . . . 193
193 ko03070 Bacterial secretion system . . . 194
194 ko00521 Streptomycin biosynthesis . . . 195
195 ko04940 Type I diabetes mellitus . . . 196
196 ko00830 Retinol metabolism . . . 197
197 ko04950 Maturity onset diabetes of the young . . . 198
198 ko01040 Biosynthesis of unsaturated fatty acids . . . 199
199 ko00460 Cyanoamino acid metabolism . . . 200
200 ko00625 Chloroalkane and chloroalkene degradation . . . 201
201 ko00363 Bisphenol degradation . . . 202
202 ko00950 Isoquinoline alkaloid biosynthesis . . . 203
203 ko04122 Sulfur relay system . . . 204
204 ko00590 Arachidonic acid metabolism . . . 205
205 ko00511 Other glycan degradation . . . 206

206 ko00072 Synthesis and degradation of ketone bodies . . . 207
207 ko00340 Histidine metabolism . . . 208
208 ko00532 Glycosaminoglycan biosynthesis - chondroitin sulfate . . . 209
209 ko00930 Caprolactam degradation . . . 210
210 ko00281 Geraniol degradation . . . 211
211 ko04640 Hematopoietic cell lineage . . . 212
212 ko00960 Tropane, piperidine and pyridine alkaloid biosynthesis . . . 213
213 ko00740 Riboflavin metabolism . . . 214
214 ko00626 Naphthalene degradation . . . 215
215 ko00920 Sulfur metabolism . . . 216
216 ko00053 Ascorbate and aldarate metabolism . . . 217
217 ko00140 Steroid hormone biosynthesis . . . 218
218 ko00624 Polycyclic aromatic hydrocarbon degradation . . . 219
219 ko00061 Fatty acid biosynthesis . . . 220
220 ko05322 Systemic lupus erythematosus . . . 221
221 ko04975 Fat digestion and absorption . . . 222
222 ko04742 Taste transduction . . . 223
223 ko00750 Vitamin B6 metabolism . . . 224
224 ko00601 Glycosphingolipid biosynthesis - lacto and neolacto series . . . 225
225 ko00430 Taurine and hypotaurine metabolism . . . 226
226 ko04711 Circadian rhythm - fly . . . 227
227 ko00785 Lipoic acid metabolism . . . 228
228 ko00603 Glycosphingolipid biosynthesis - globo series . . . 229
229 ko00643 Styrene degradation . . . 230
230 ko04712 Circadian rhythm - plant . . . 231
231 ko05340 Primary immunodeficiency . . . 232
232 ko00440 Phosphonate and phosphinate metabolism . . . 233
233 ko00730 Thiamine metabolism . . . 234
234 ko00780 Biotin metabolism . . . 235
235 ko00591 Linoleic acid metabolism . . . 236
236 ko00400 Phenylalanine, tyrosine and tryptophan biosynthesis . . . 237

237 ko00965 Betalain biosynthesis . . . 238
238 ko00940 Phenylpropanoid biosynthesis . . . 239
239 ko00512 Mucin type O-Glycan biosynthesis . . . 240
240 ko05150 Staphylococcus aureus infection . . . 241
241 ko05144 Malaria . . . 242
242 ko00770 Pantothenate and CoA biosynthesis . . . 243
243 ko00471 D-Glutamine and D-glutamate metabolism . . . 244
244 ko00604 Glycosphingolipid biosynthesis - ganglio series . . . 245
245 ko00901 Indole alkaloid biosynthesis . . . 246
246 ko00945 Stilbenoid, diarylheptanoid and gingerol biosynthesis . . . 247
247 ko01055 Biosynthesis of vancomycin group antibiotics . . . 248
248 ko04112 Cell cycle - Caulobacter . . . 249
249 ko04744 Phototransduction . . . 250
250 ko00533 Glycosaminoglycan biosynthesis - keratan sulfate . . . 251
251 ko00642 Ethylbenzene degradation . . . 252
252 ko00100 Steroid biosynthesis . . . 253
253 ko01051 Biosynthesis of ansamycins . . . 254
254 ko00660 C5-Branched dibasic acid metabolism . . . 255
255 ko00524 Butirosin and neomycin biosynthesis . . . 256
256 ko00944 Flavone and flavonol biosynthesis . . . 257
257 ko00592 alpha-Linolenic acid metabolism . . . 258
258 ko00401 Novobiocin biosynthesis . . . 259
259 ko00531 Glycosaminoglycan degradation . . . 260
260 ko00908 Zeatin biosynthesis . . . 261
261 ko00523 Polyketide sugar unit biosynthesis . . . 262
262 ko04977 Vitamin digestion and absorption . . . 263

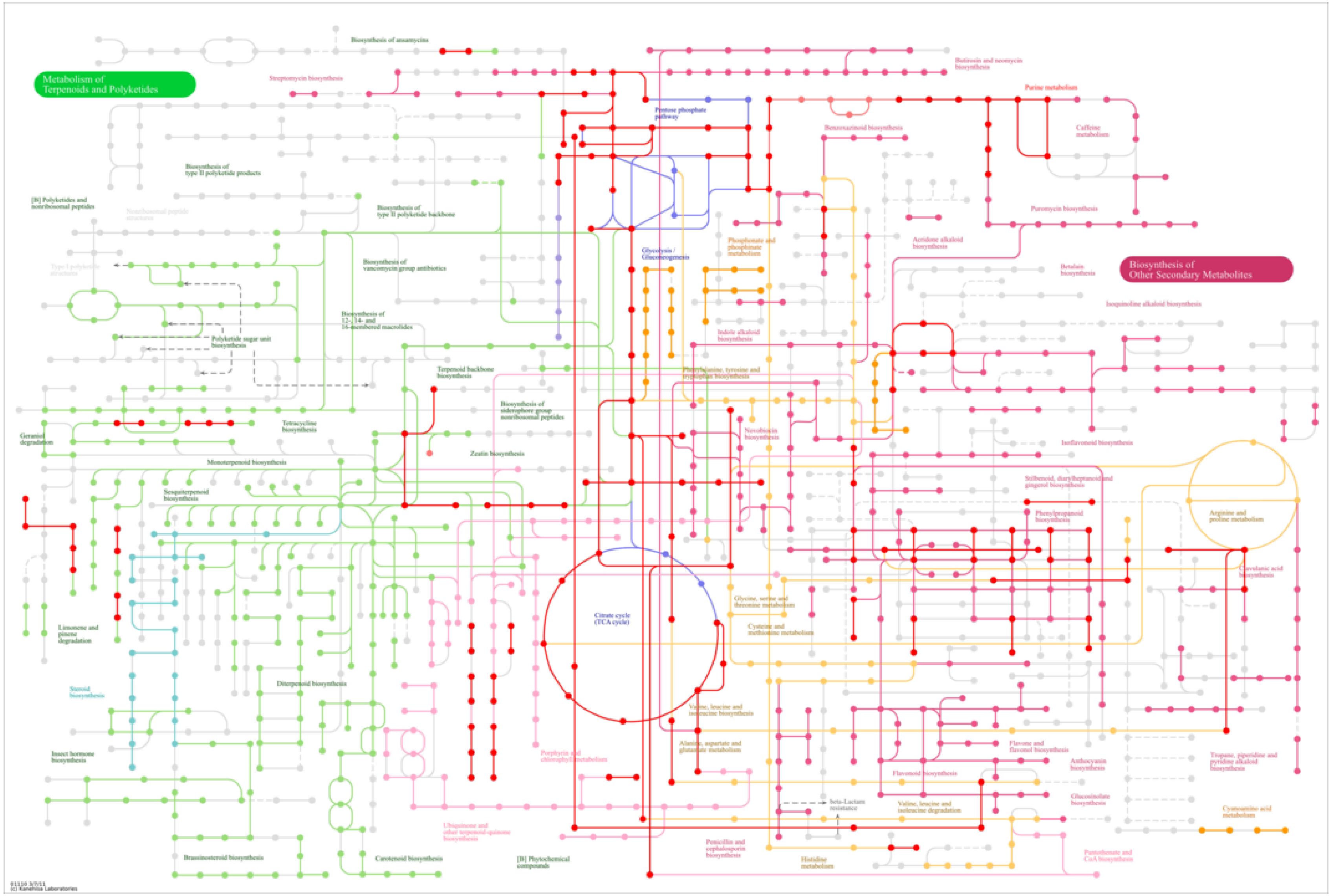

Figure 1: ko01110 Biosynthesis of secondary metabolites

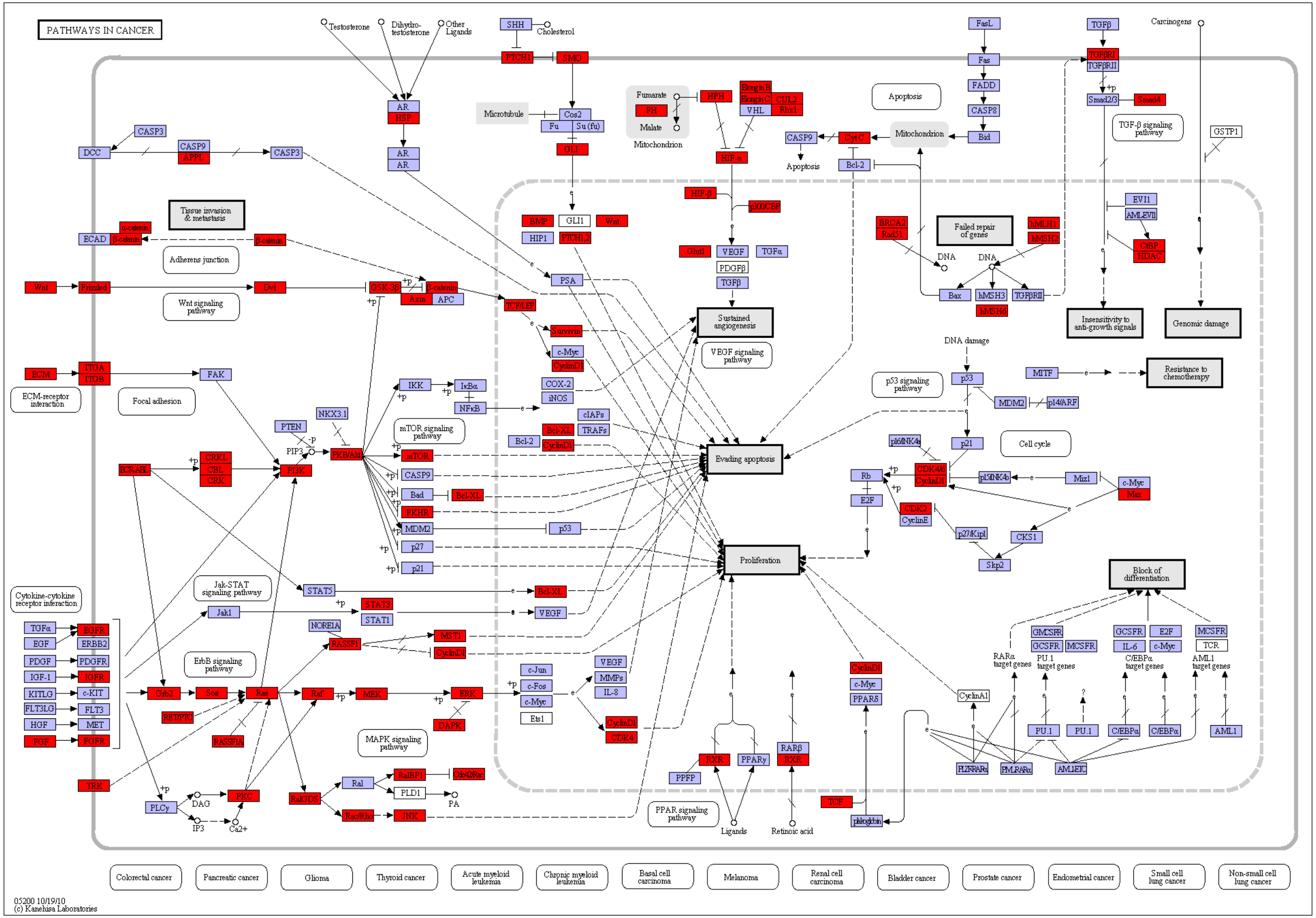

Figure 2: ko05200 Pathways in cancer

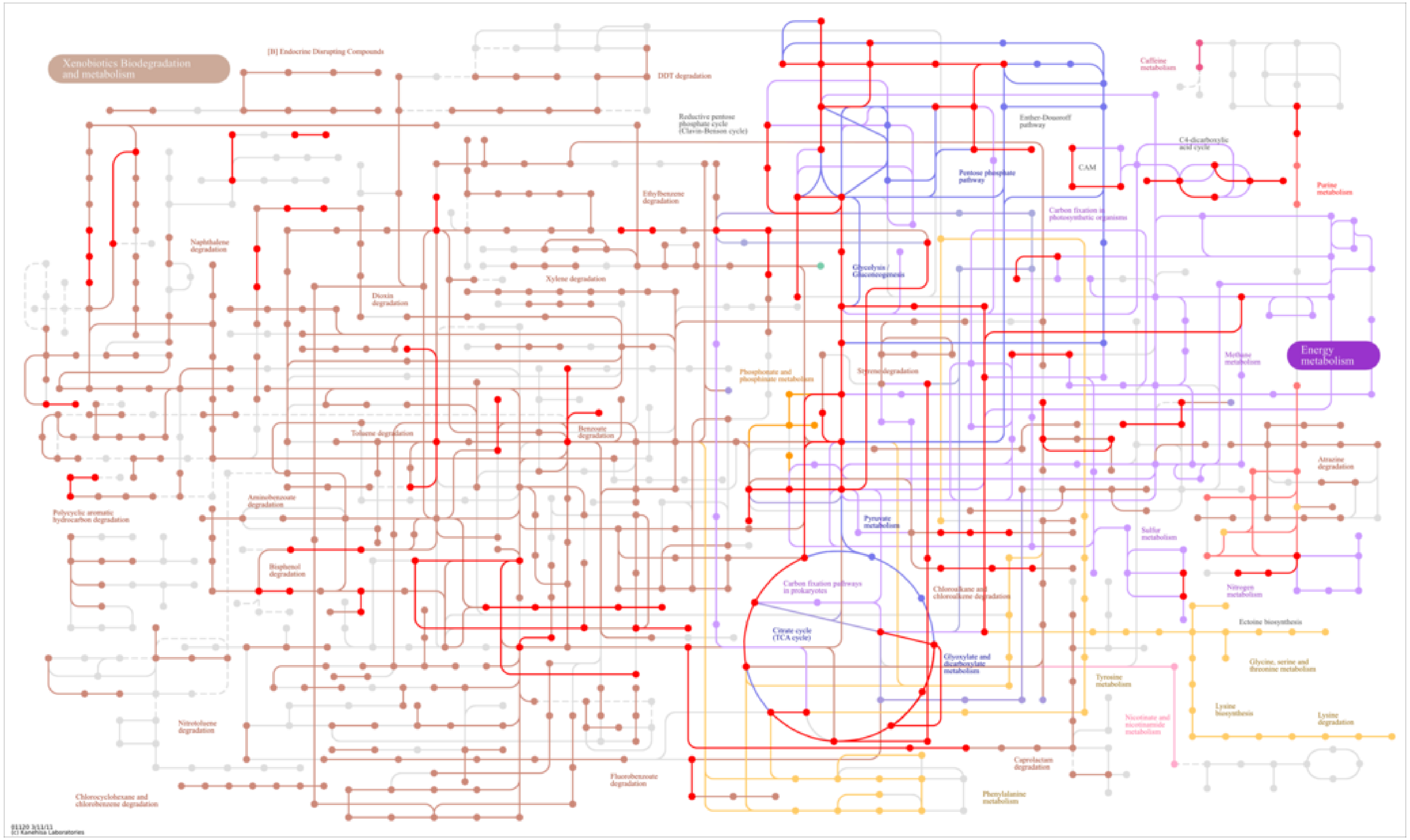

Figure 3: ko01120 Microbial metabolism in diverse environments

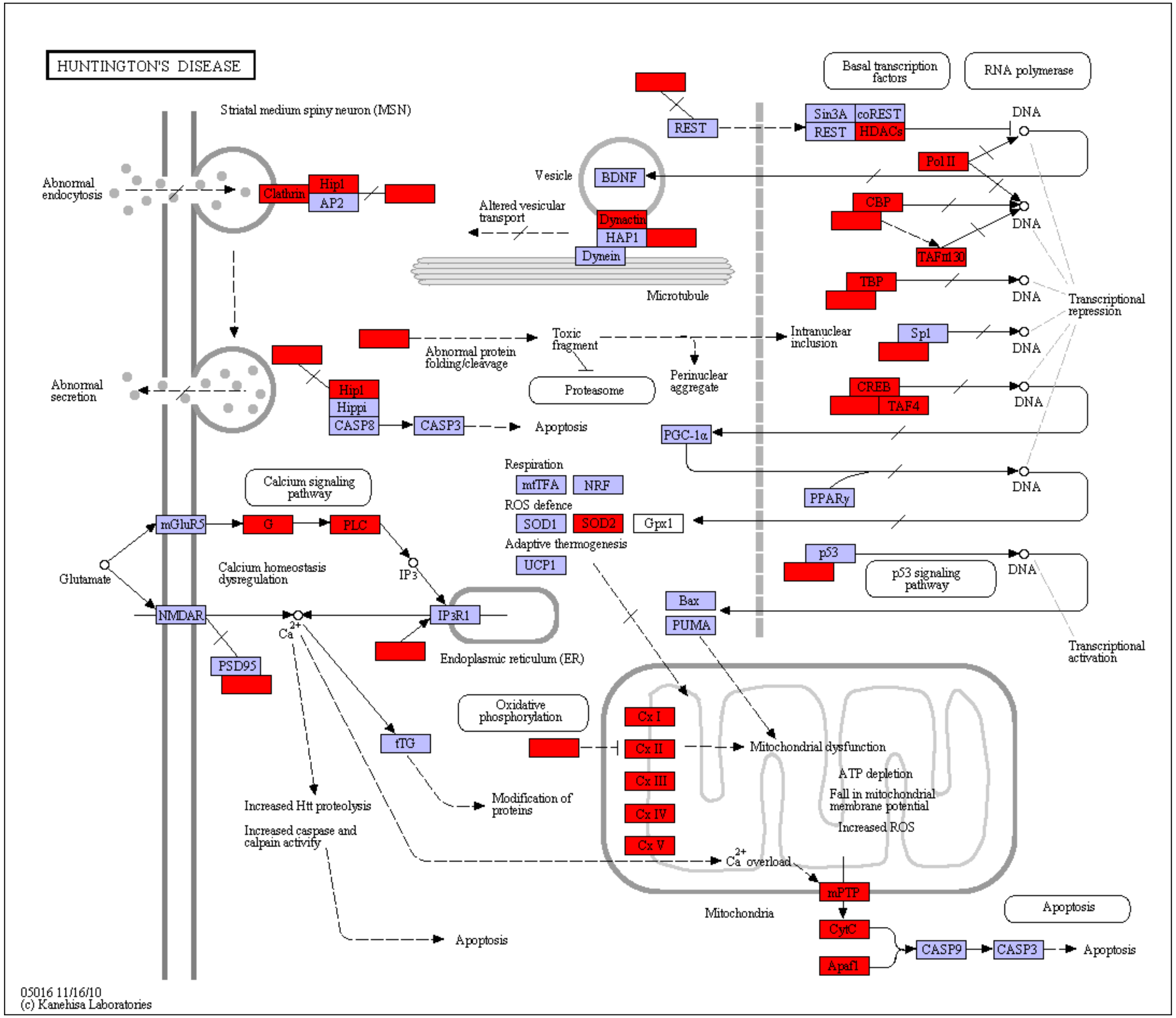

Figure 4: ko05016 Huntington's disease

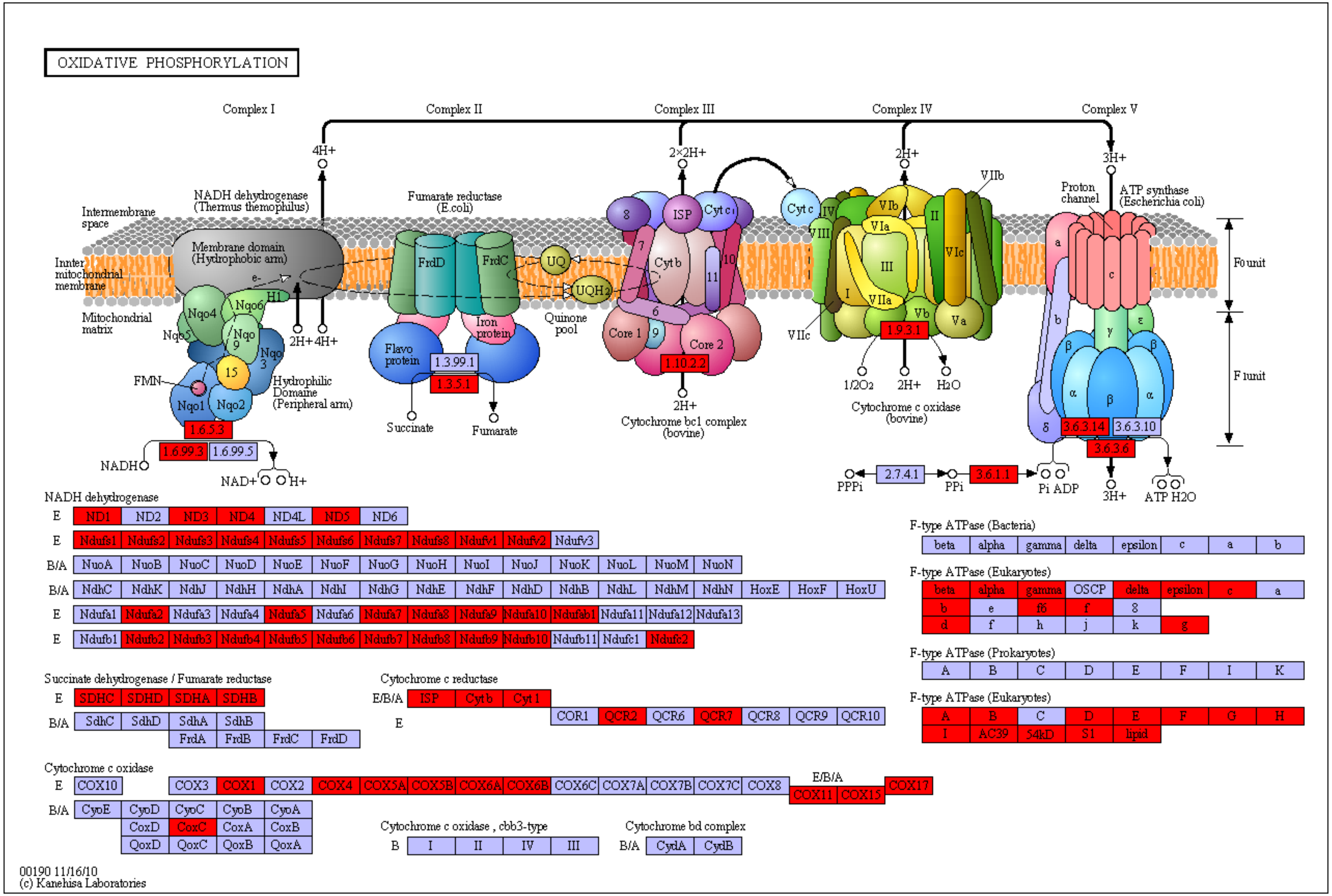

Figure 5: ko00190 Oxidative phosphorylation

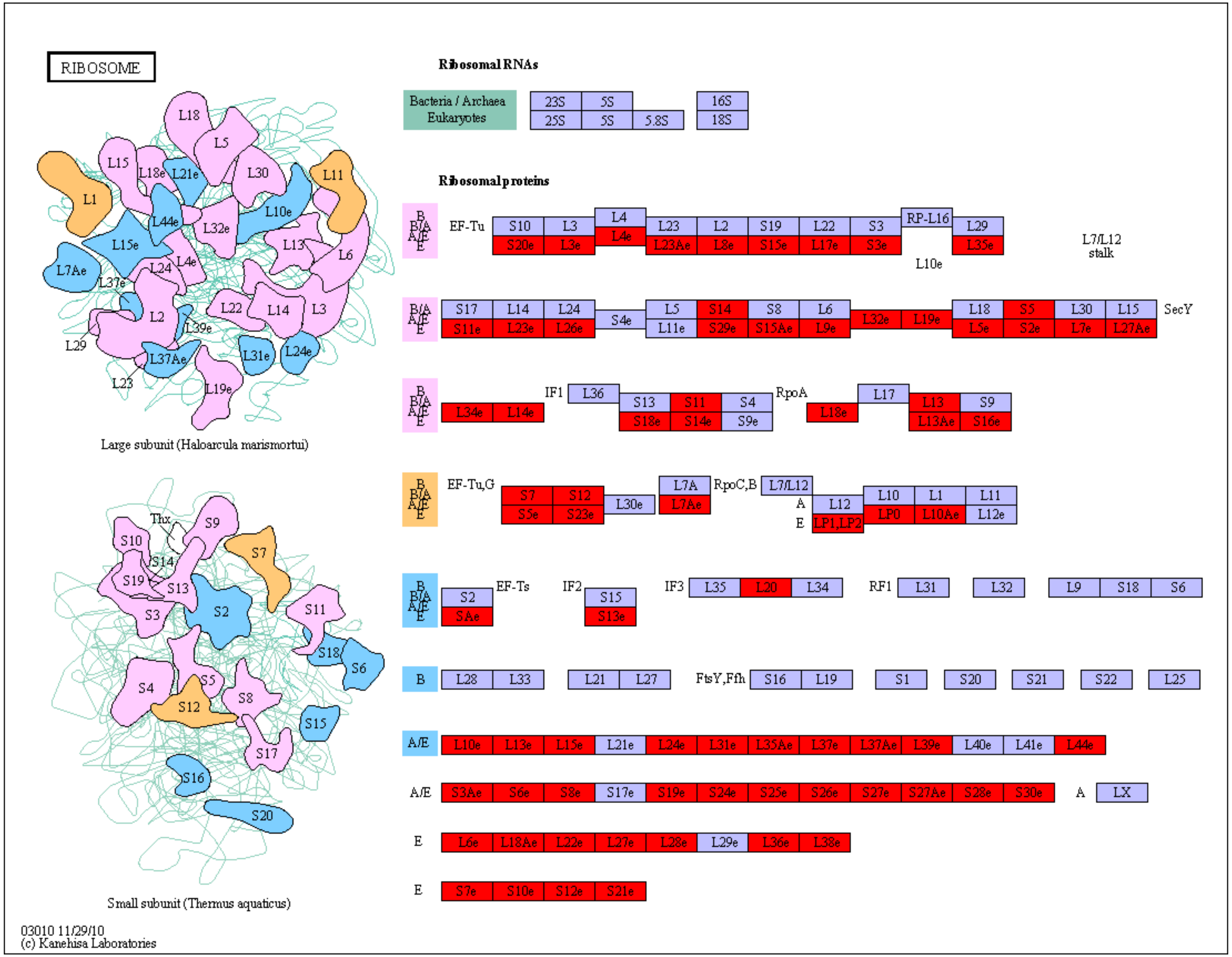

Figure 6: ko03010 Ribosome

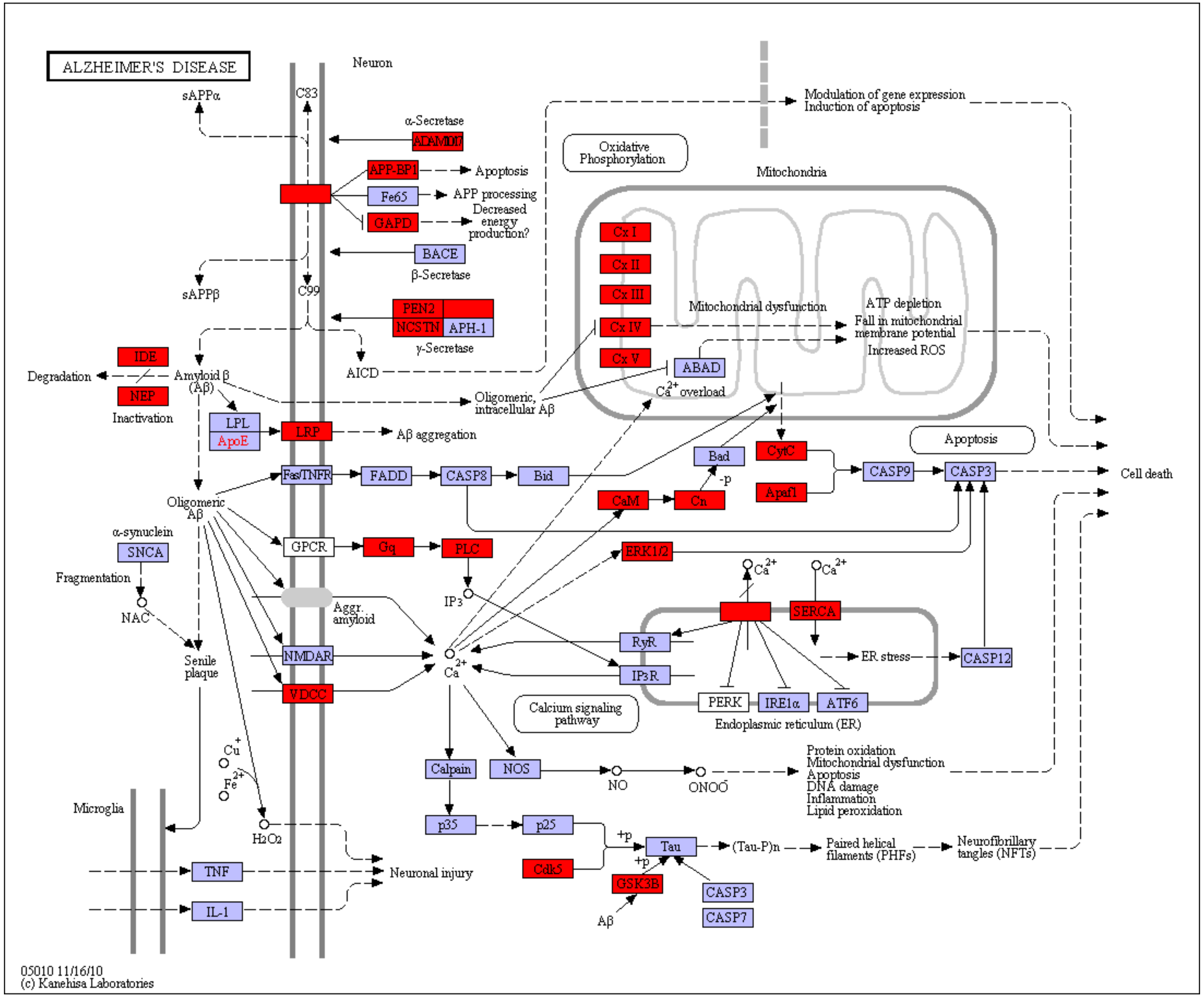

Figure 7: ko05010 Alzheimer's disease

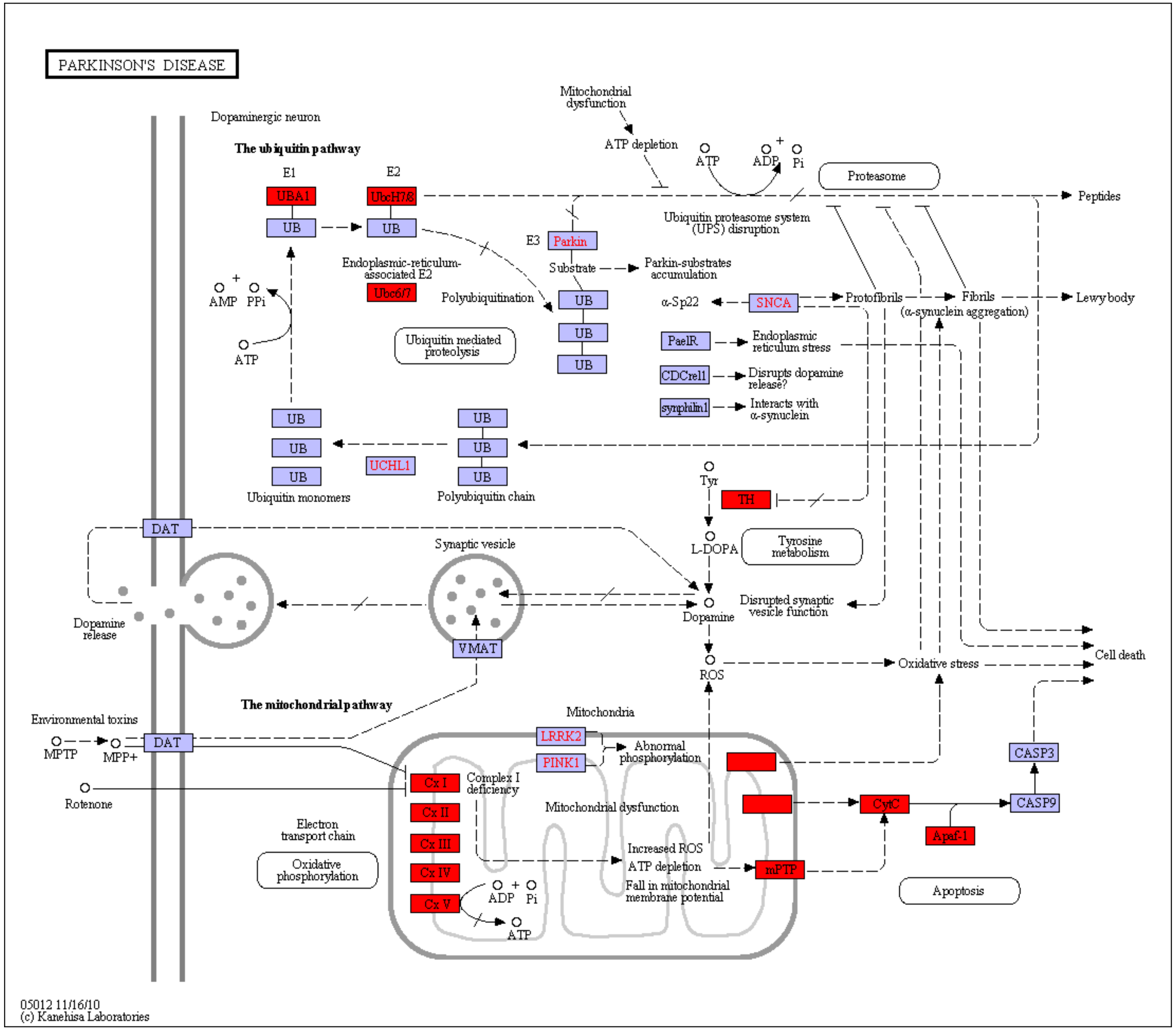

Figure 8: ko05012 Parkinson's disease

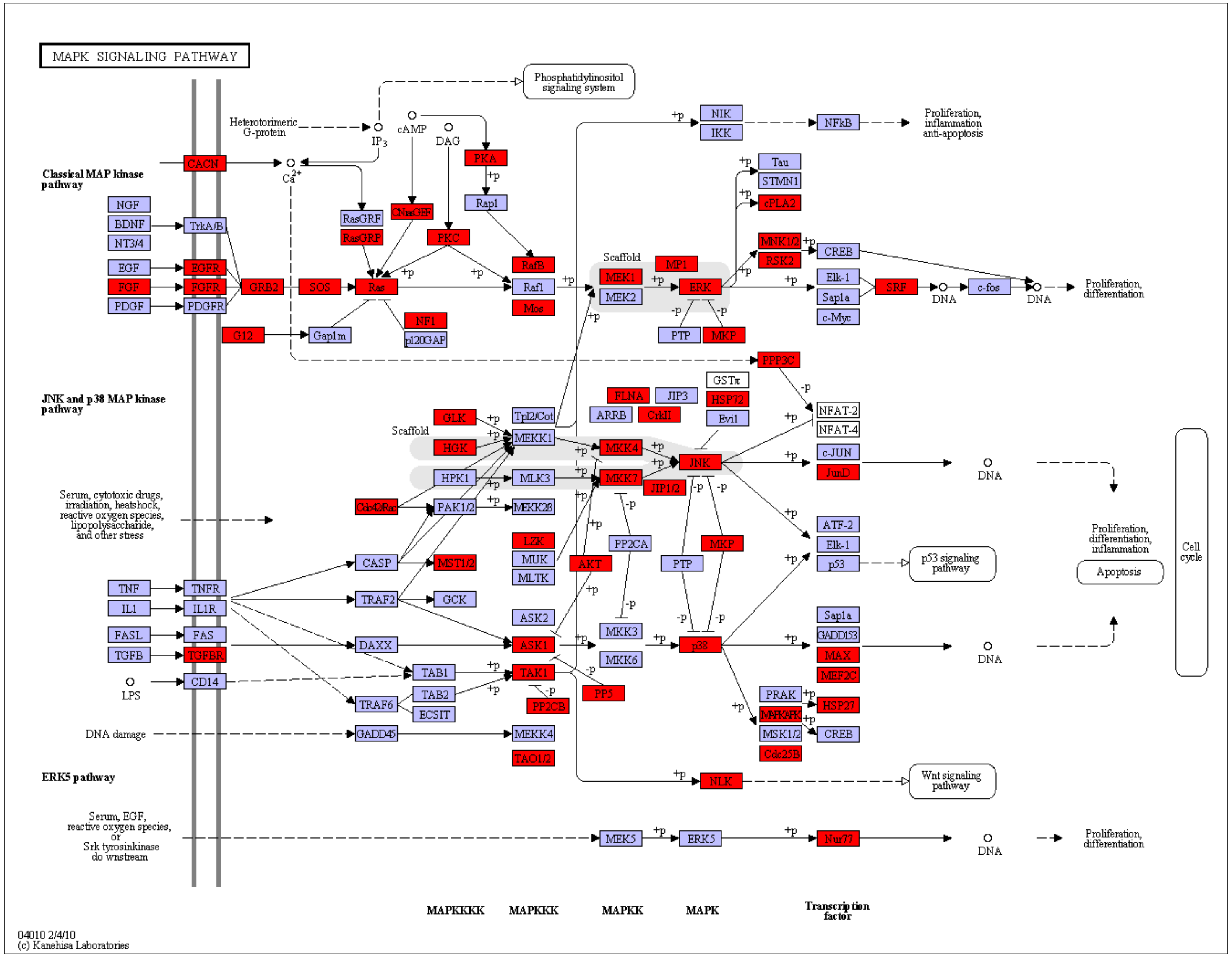

Figure 9: ko04010 MAPK signaling pathway

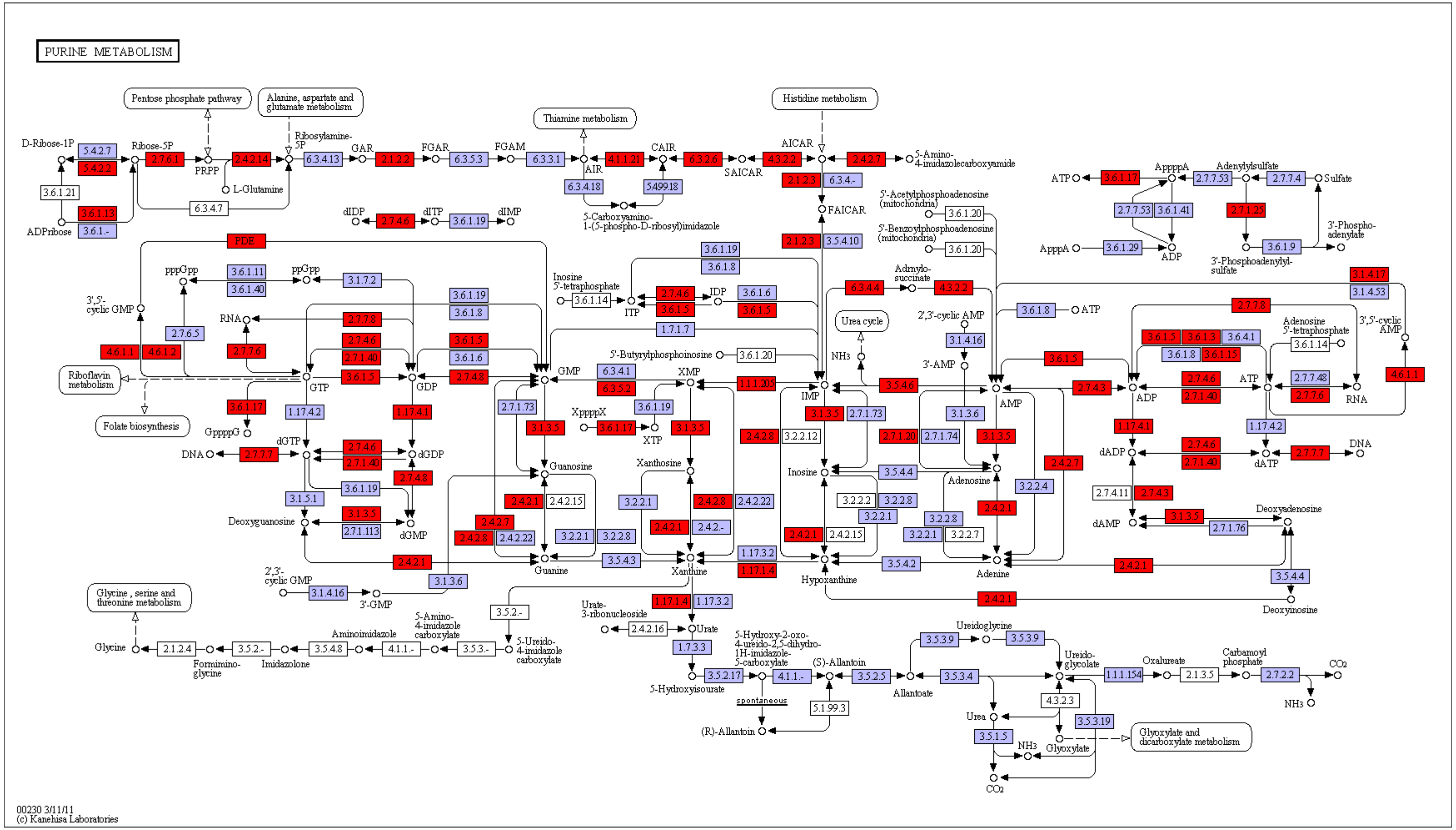

Figure 10: ko00230 Purine metabolism

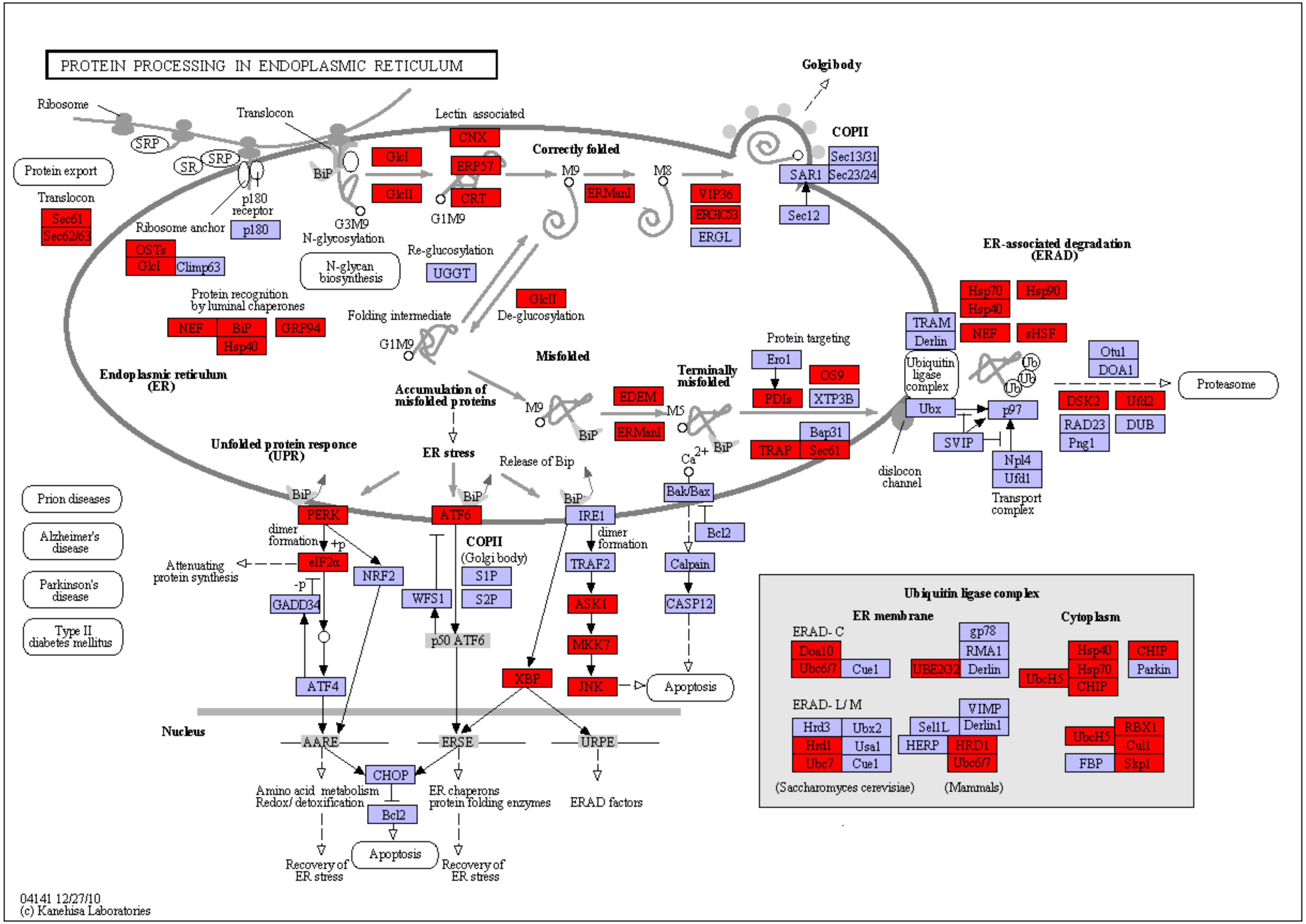

Figure 11: ko04141 Protein processing in endoplasmic reticulum

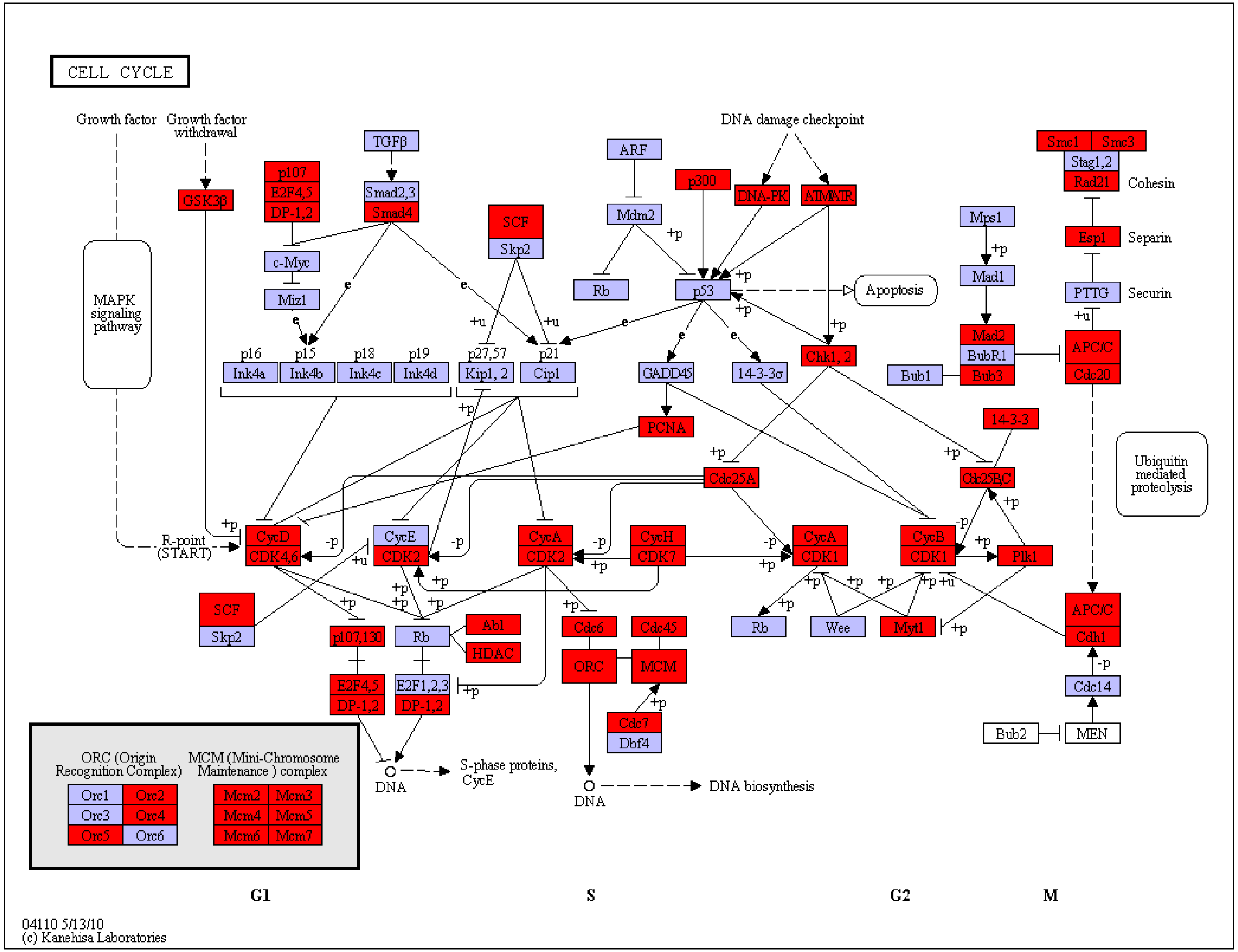

Figure 12: ko04110 Cell cycle

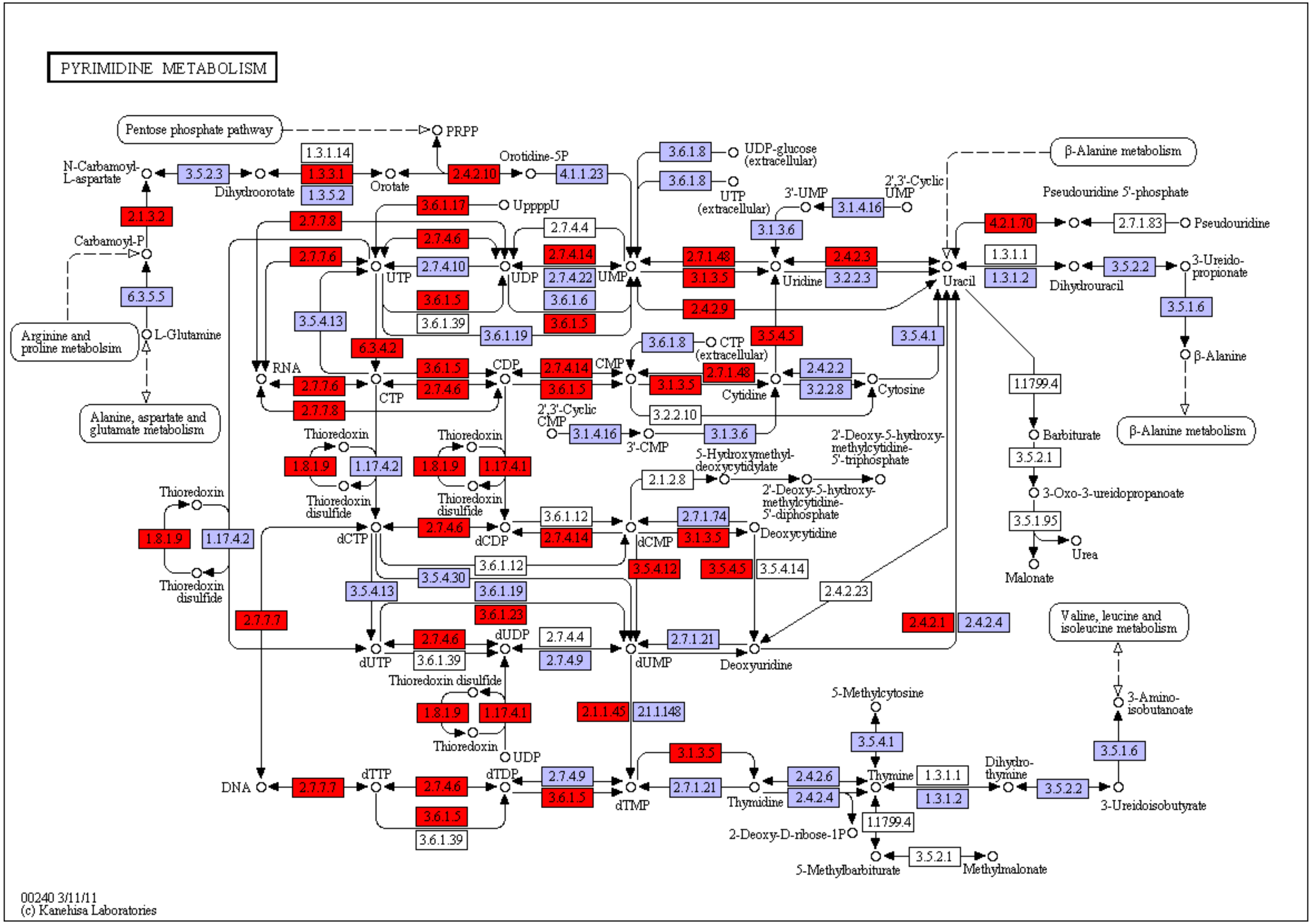

Figure 13: ko00240 Pyrimidine metabolism

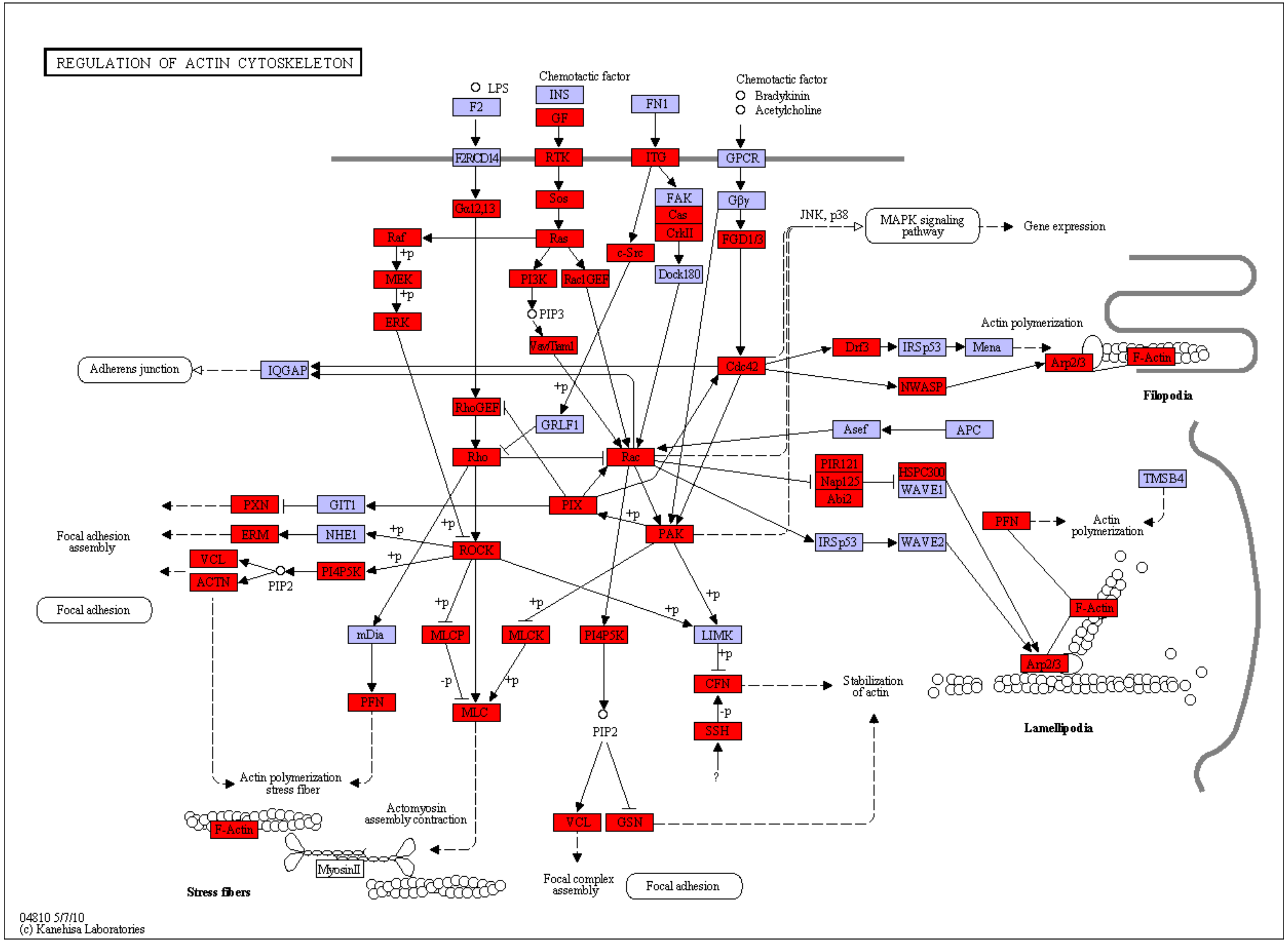

Figure 14: ko04810 Regulation of actin cytoskeleton

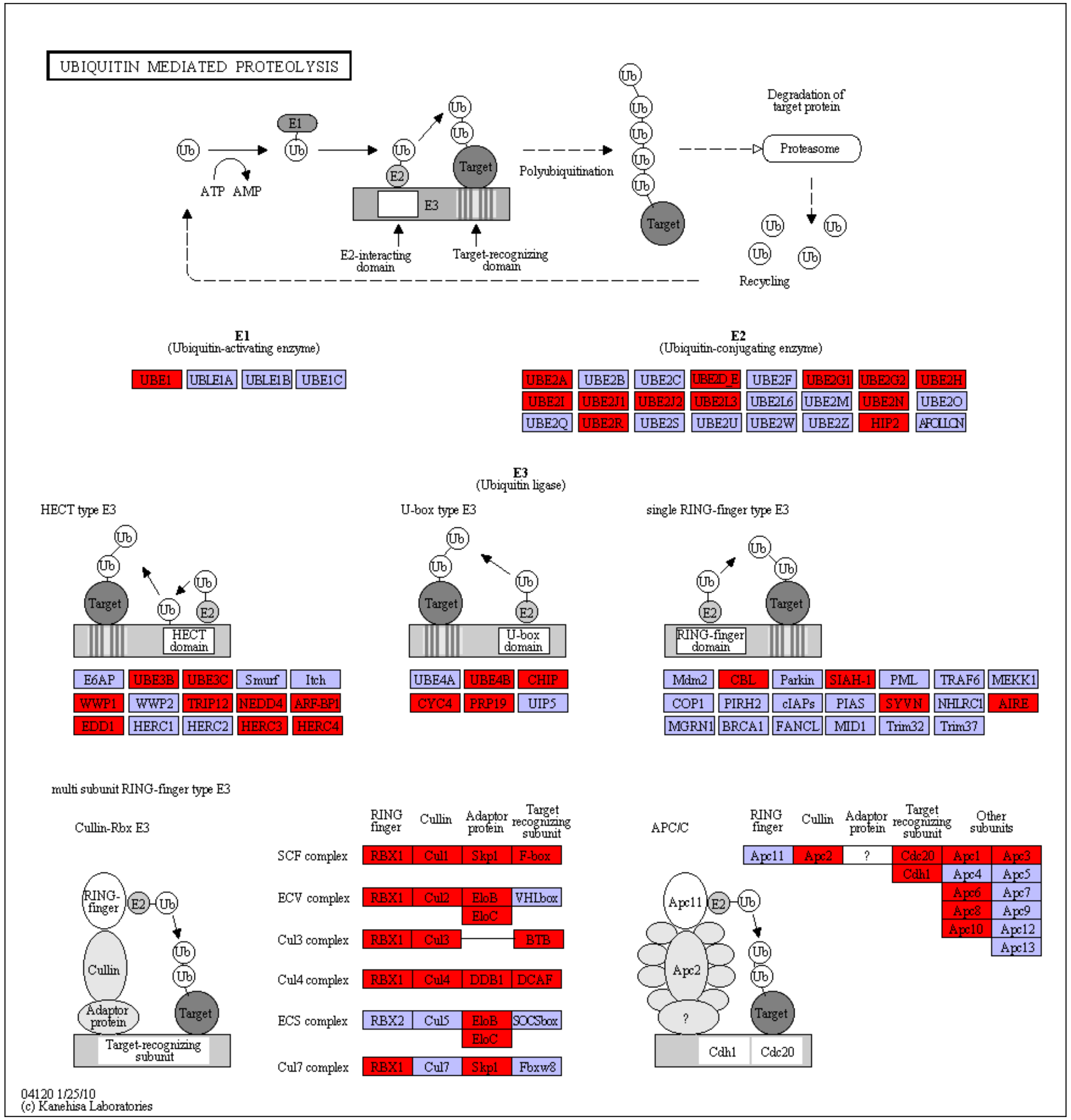

Figure 15: ko04120 Ubiquitin mediated proteolysis

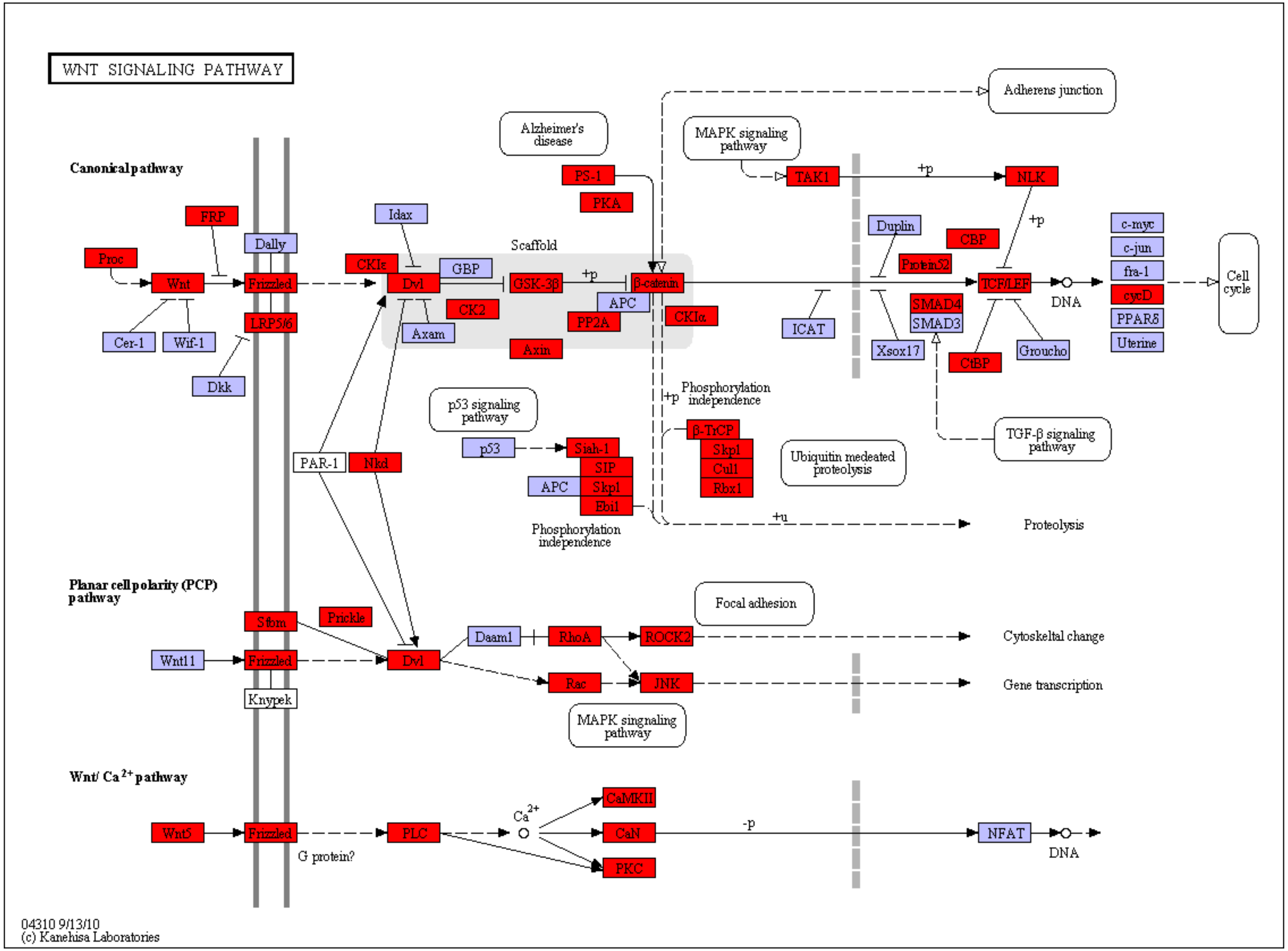

Figure 16: ko04310 Wnt signaling pathway

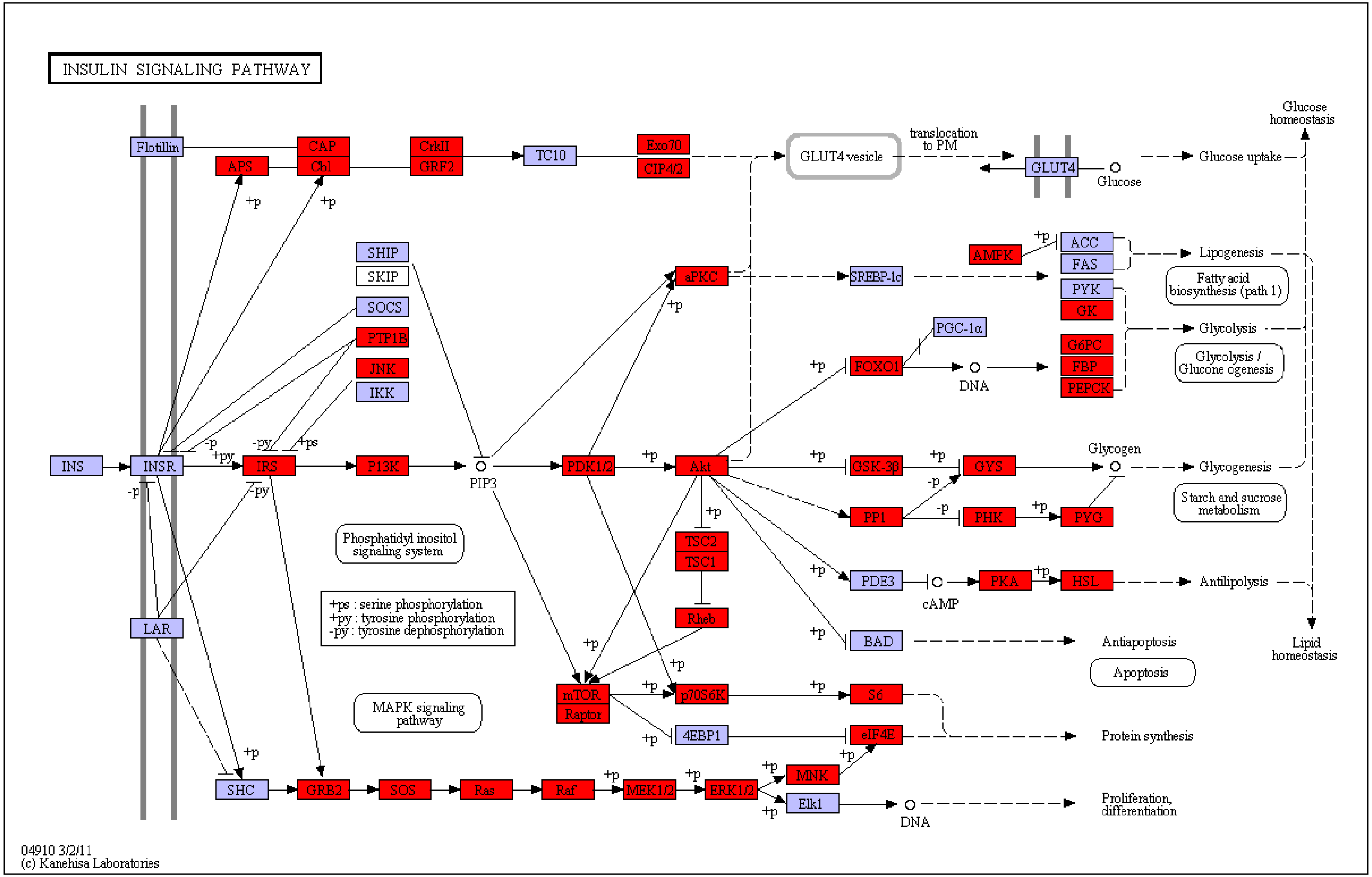

Figure 17: ko04910 Insulin signaling pathway

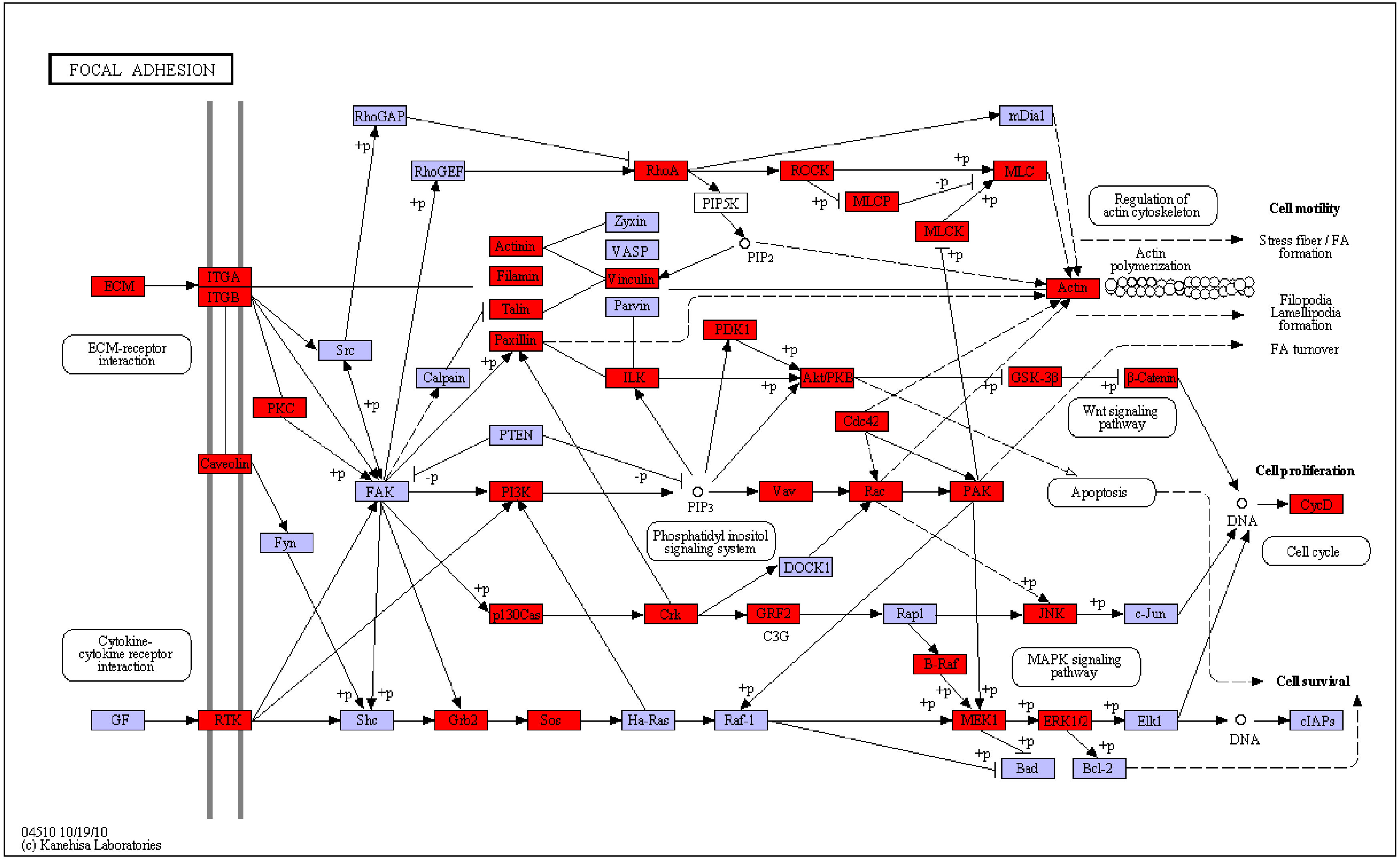

Figure 18: ko04510 Focal adhesion

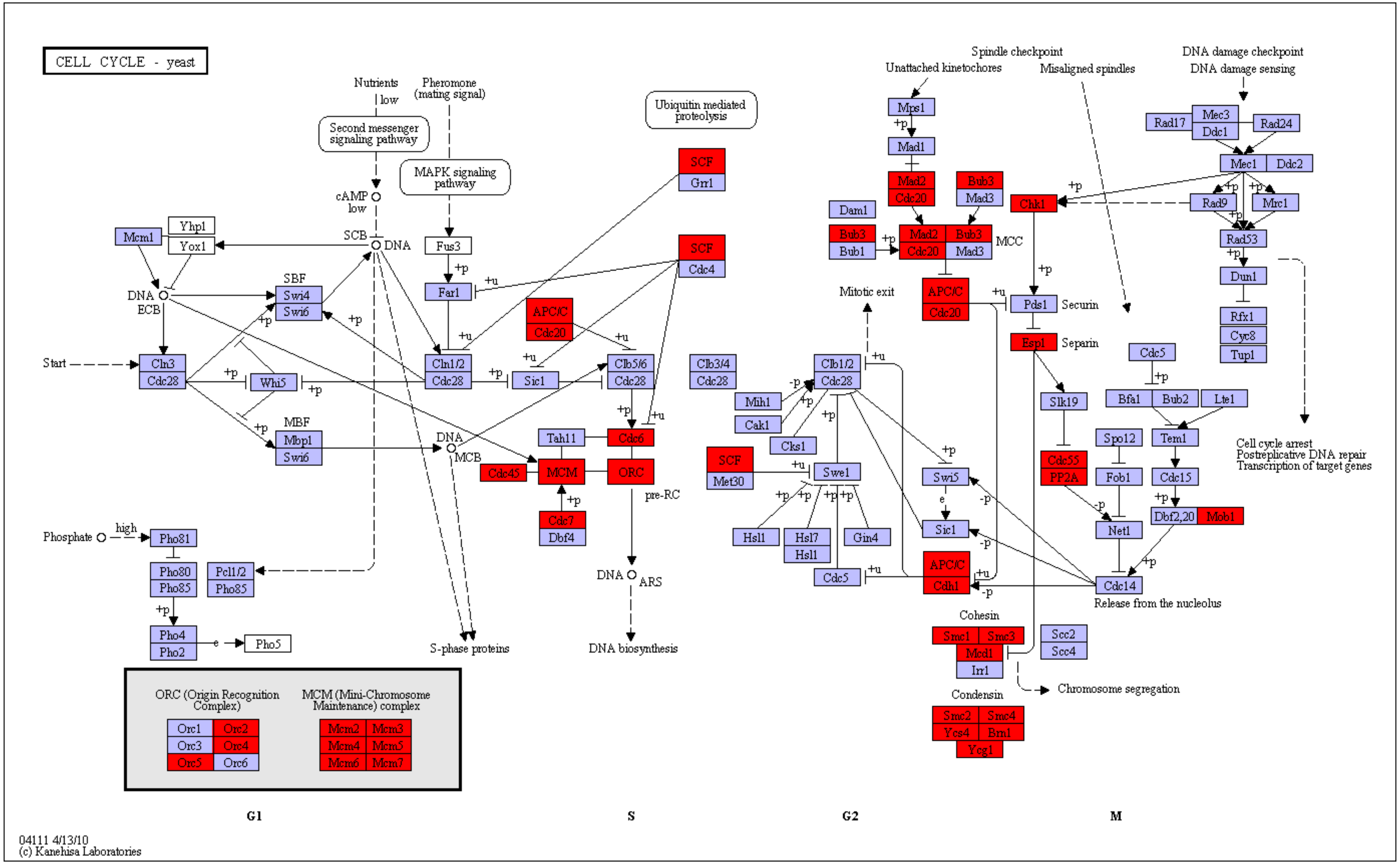

Figure 19: ko04111 Cell cycle - yeast

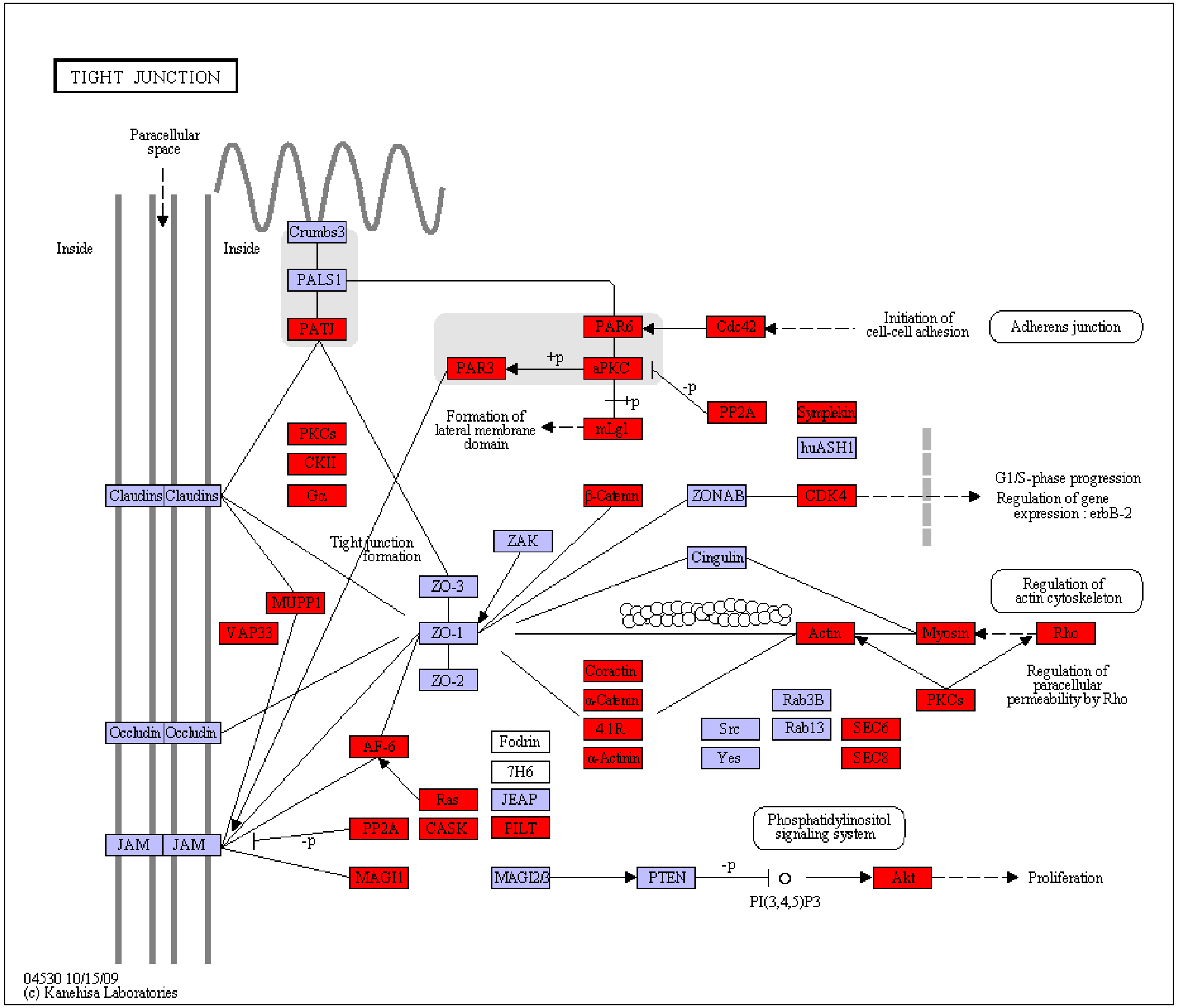

Figure 20: ko04530 Tight junction

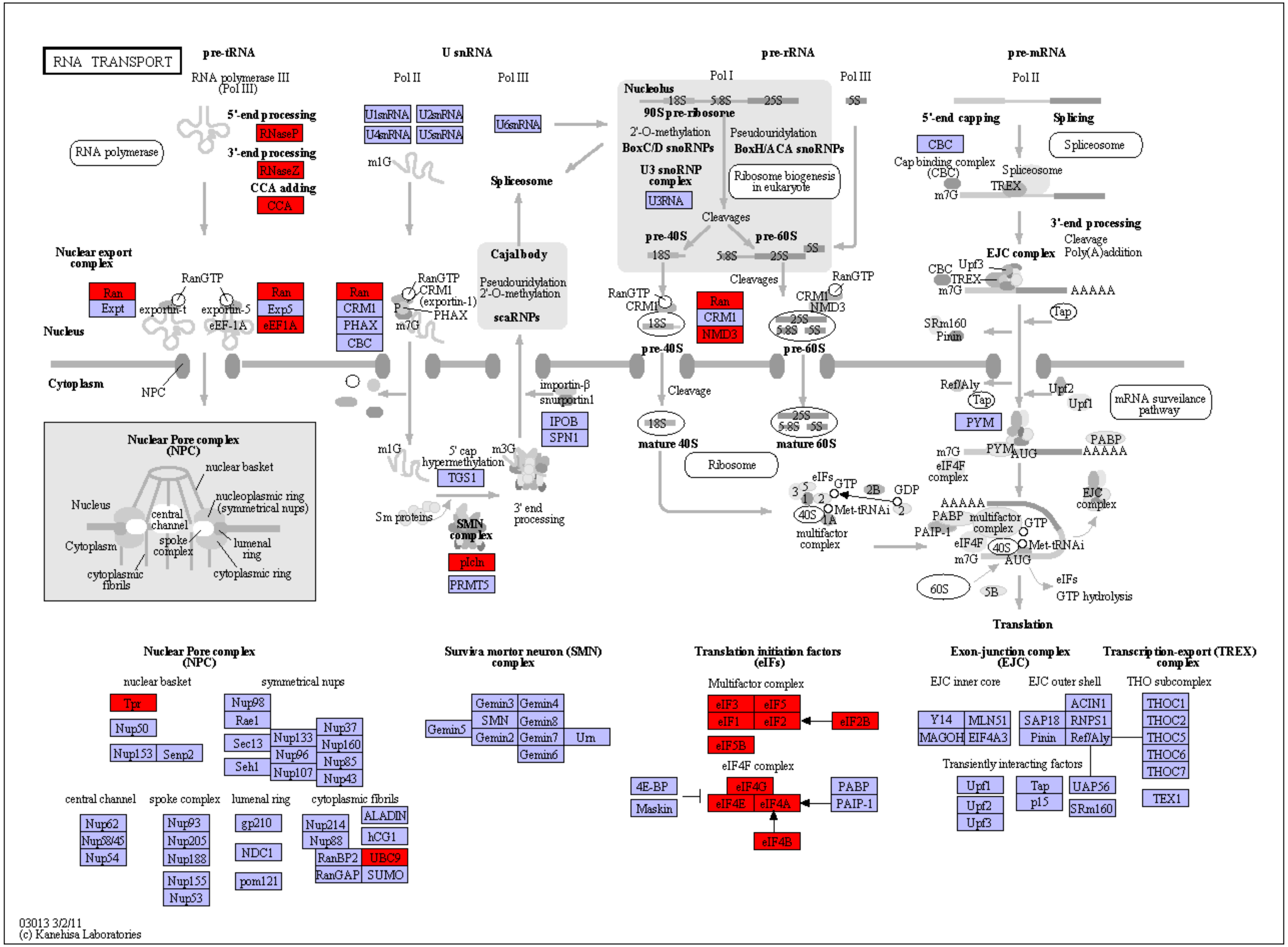

Figure 21: ko03013 RNA transport

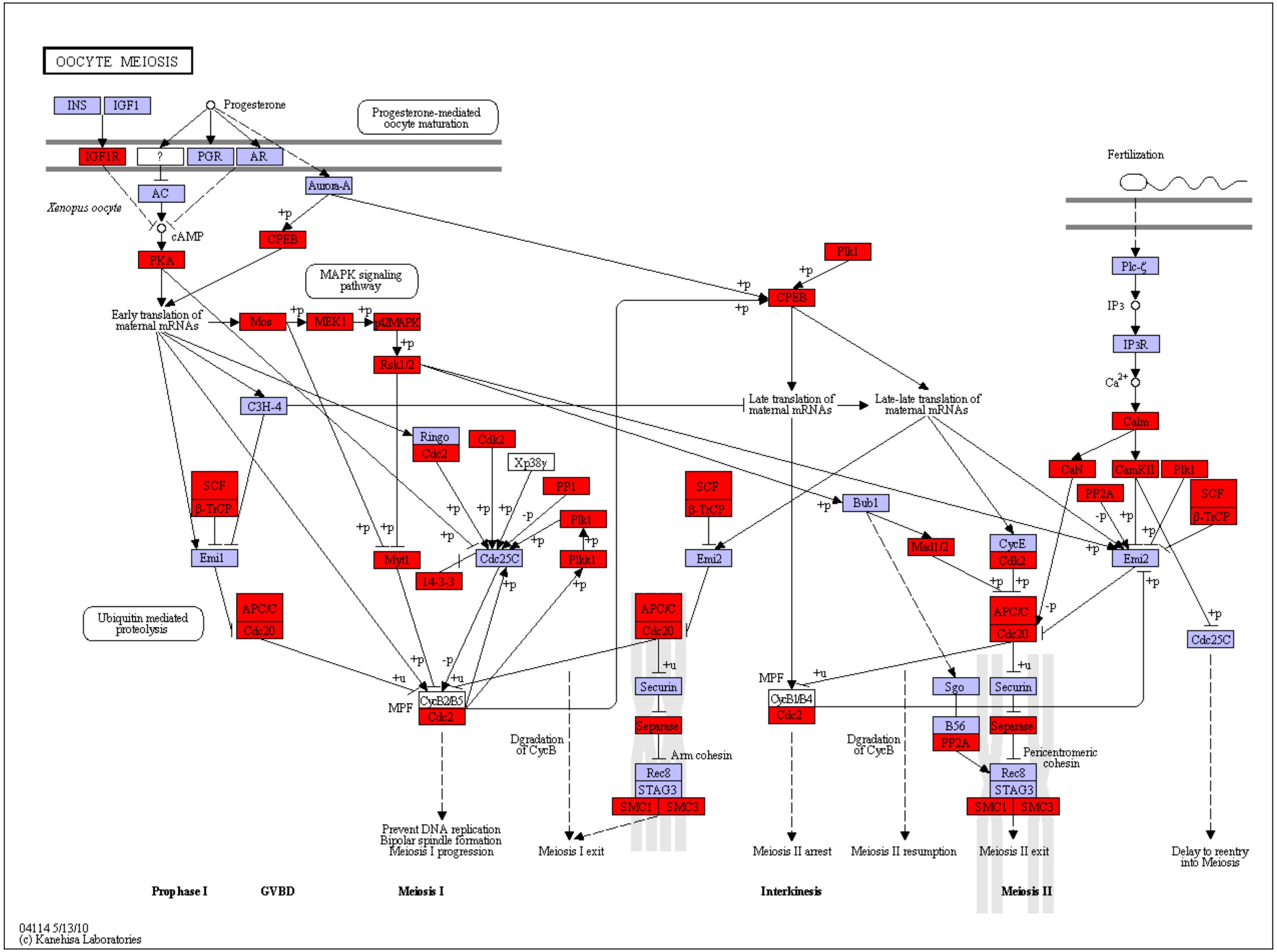

Figure 22: ko04114 Oocyte meiosis

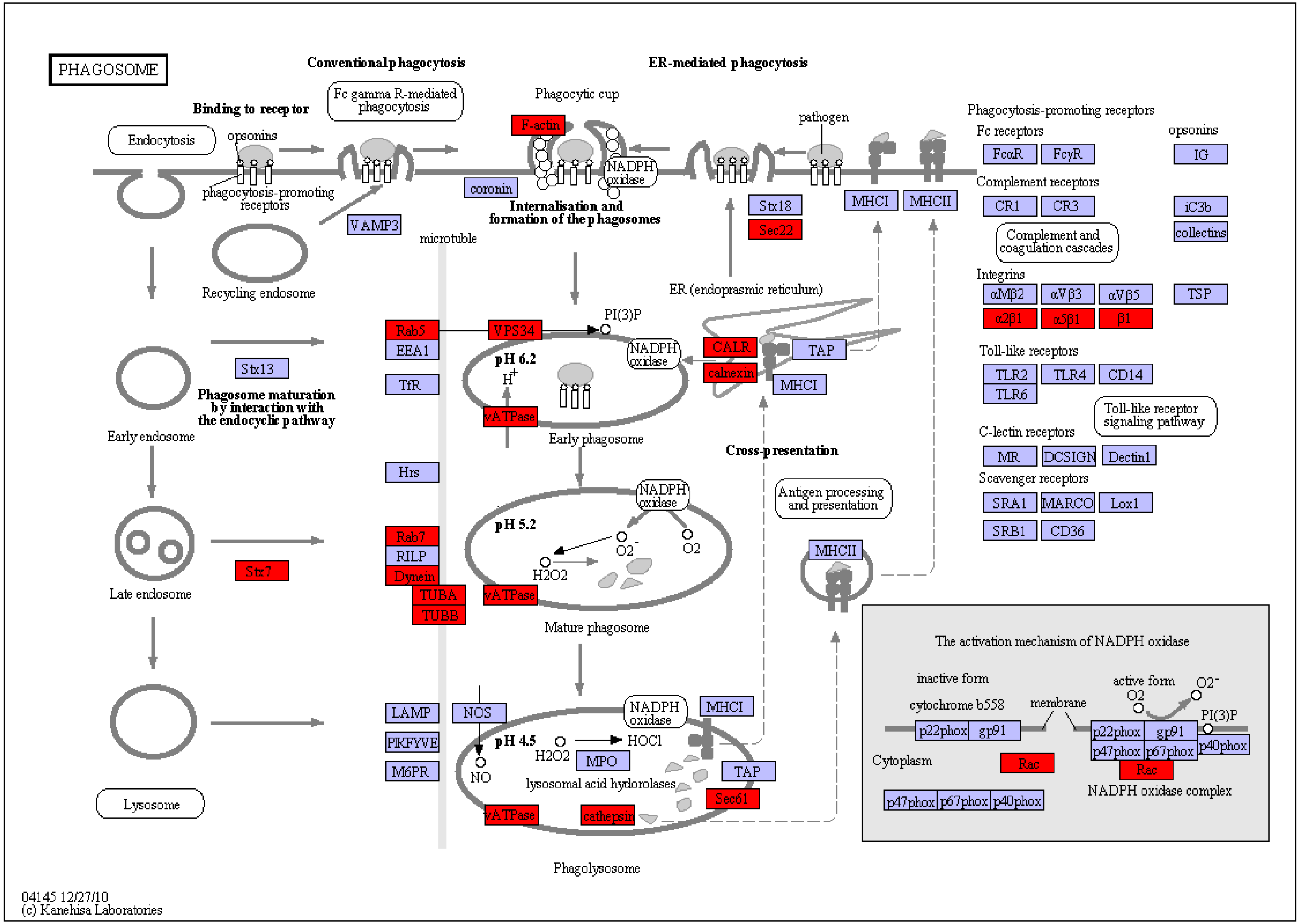

Figure 23: ko04145 Phagosome

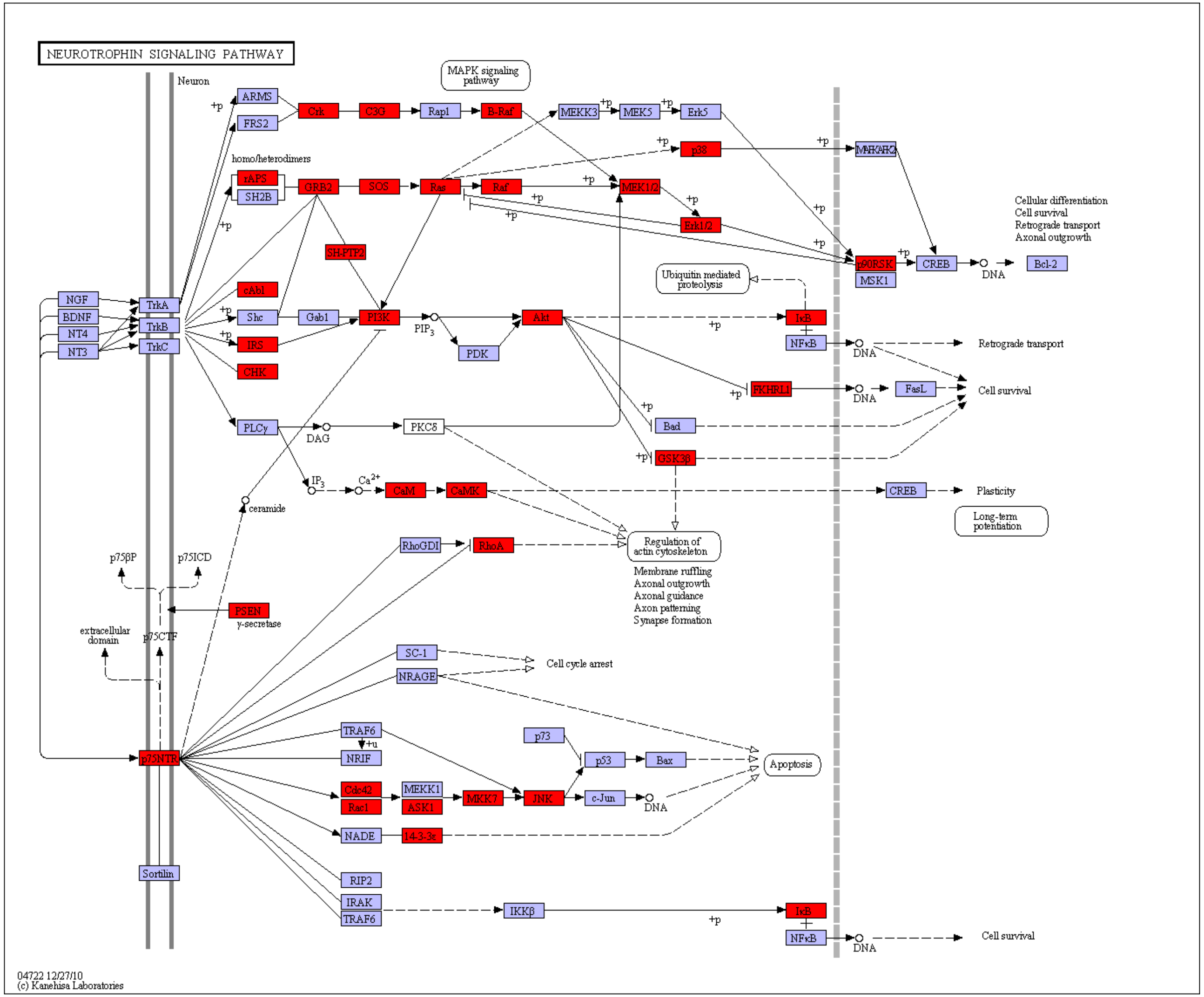

Figure 24: ko04722 Neurotrophin signaling pathway

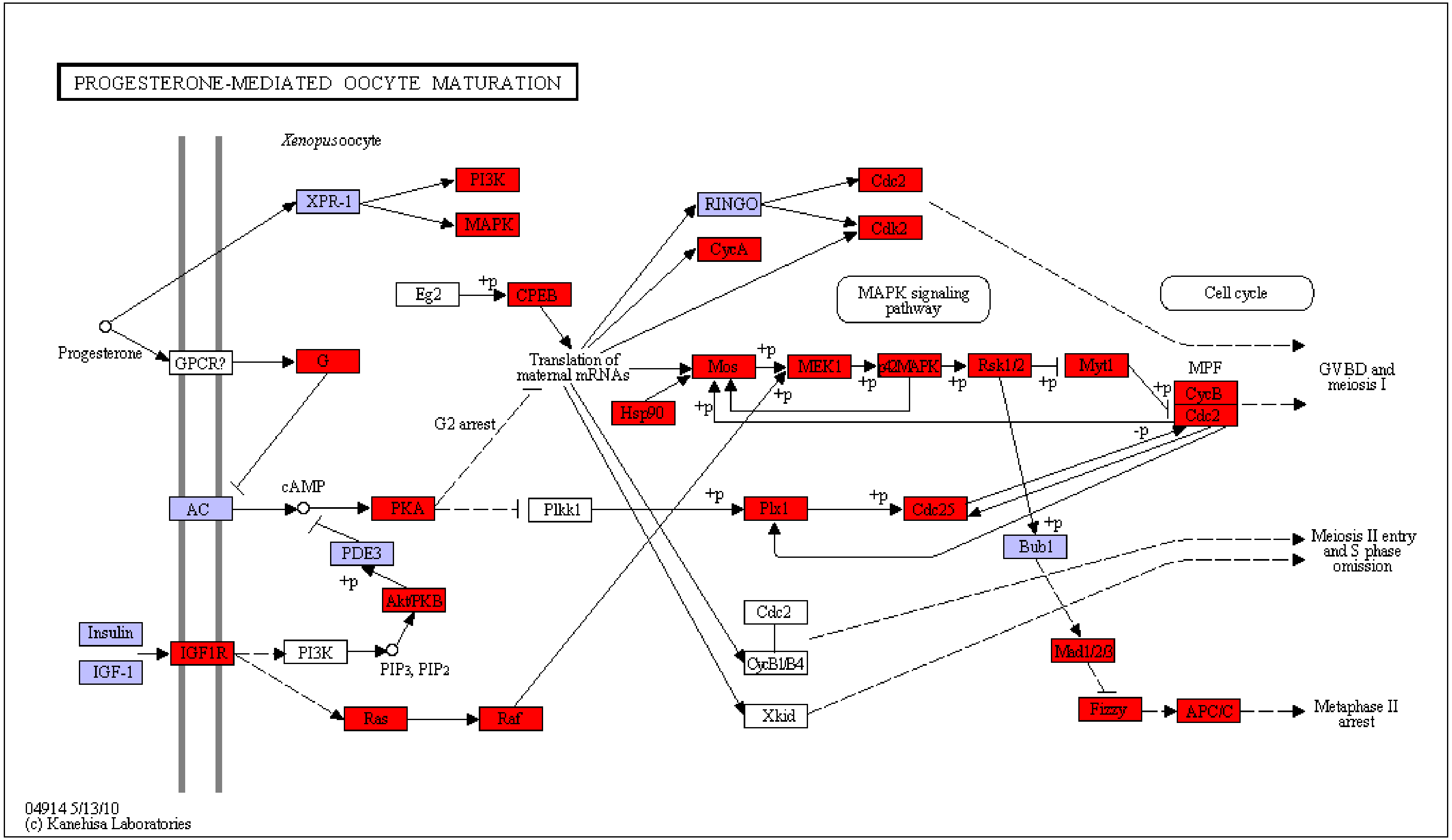

Figure 25: ko04914 Progesterone-mediated oocyte maturation

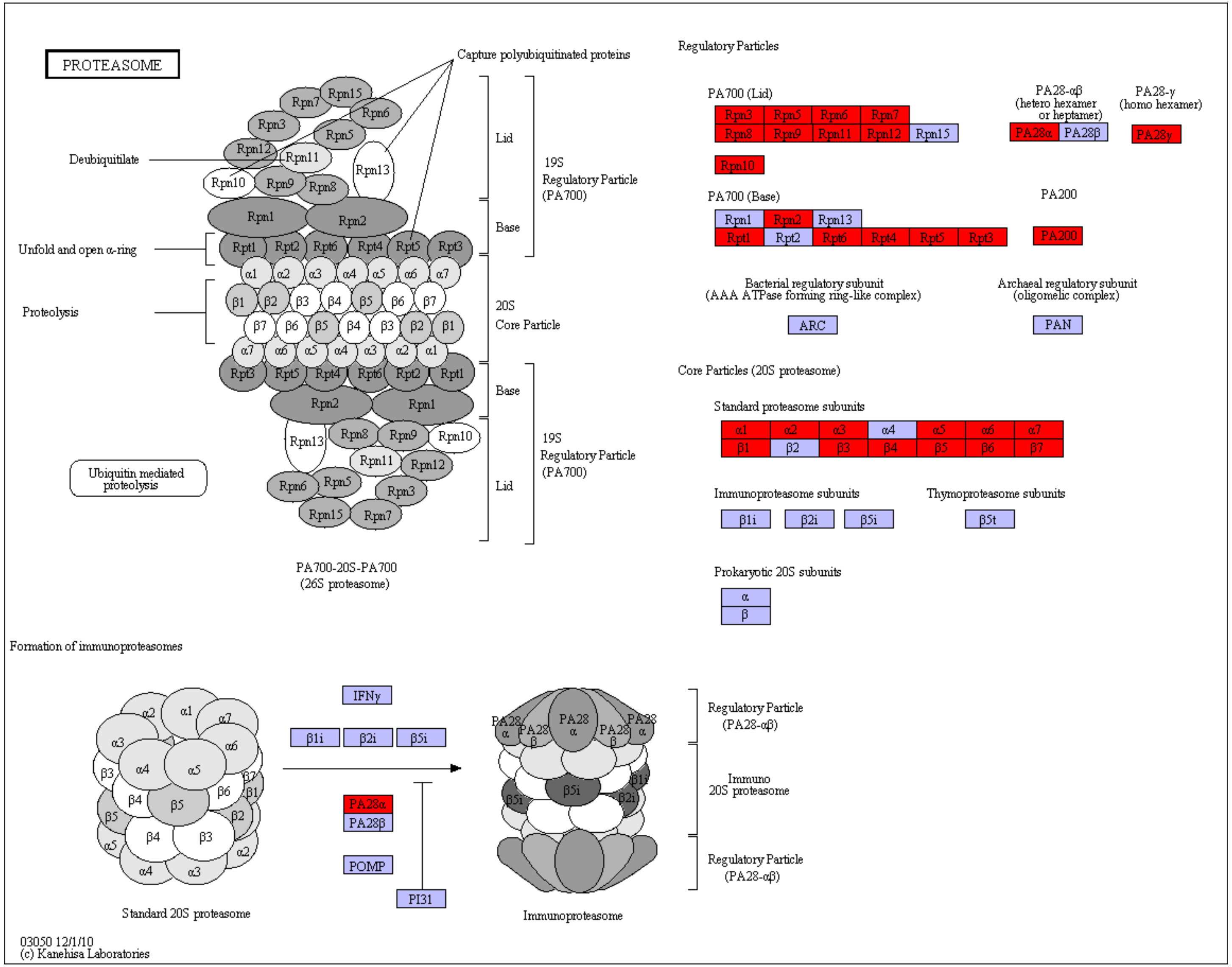

Figure 26: ko03050 Proteasome

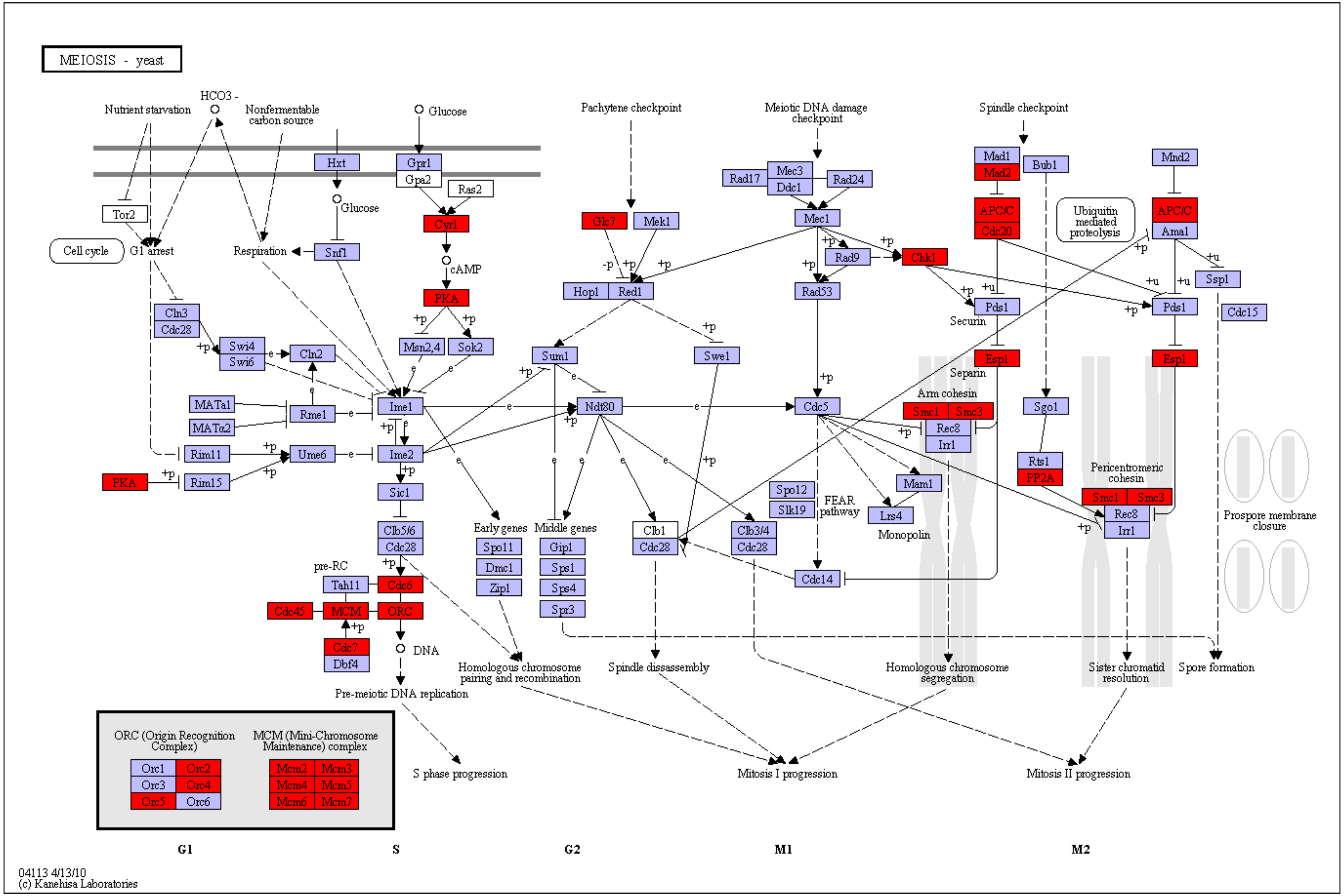

Figure 27: ko04113 Meiosis - yeast

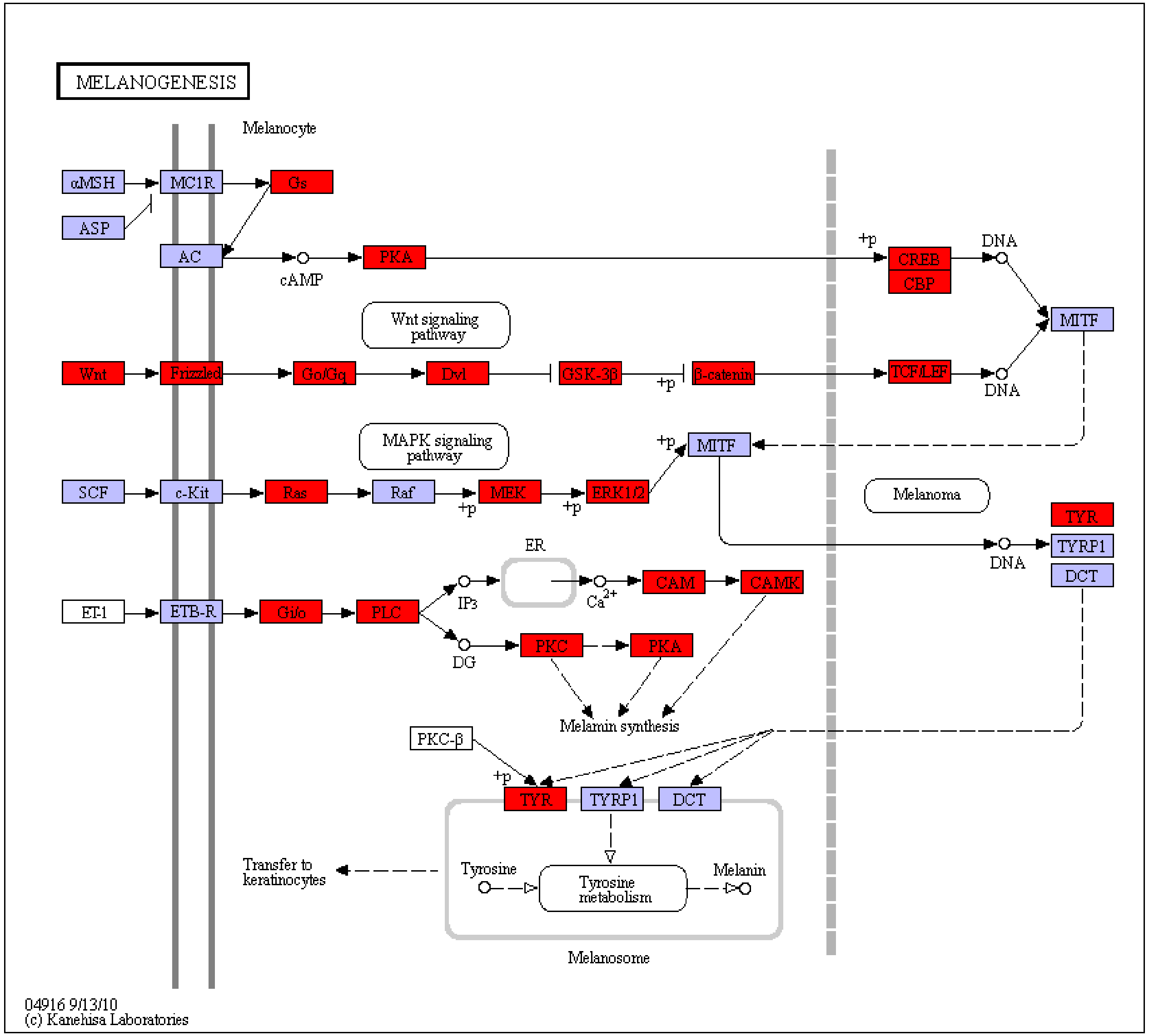

Figure 28: ko04916 Melanogenesis

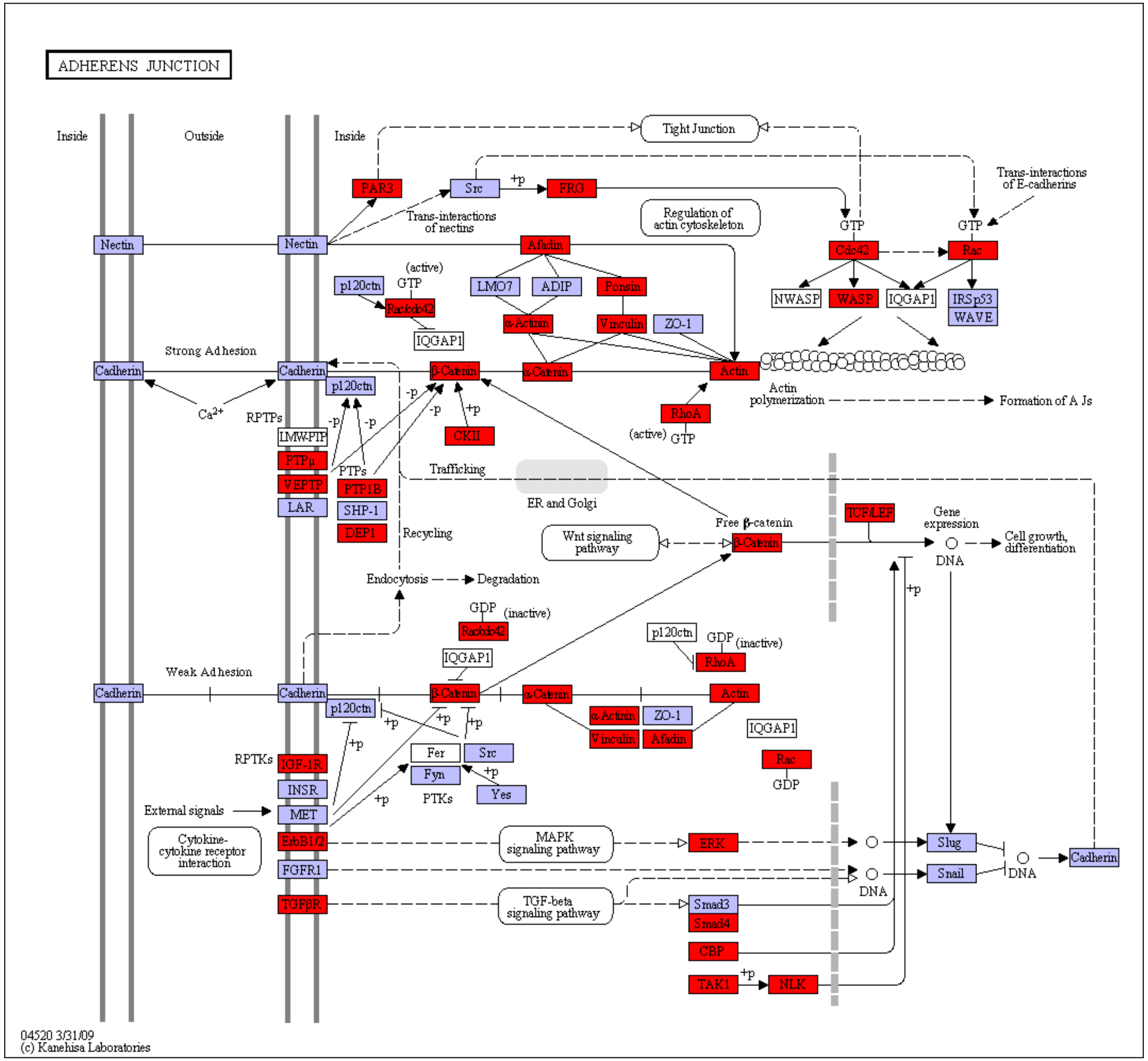

Figure 29: ko04520 Adherens junction

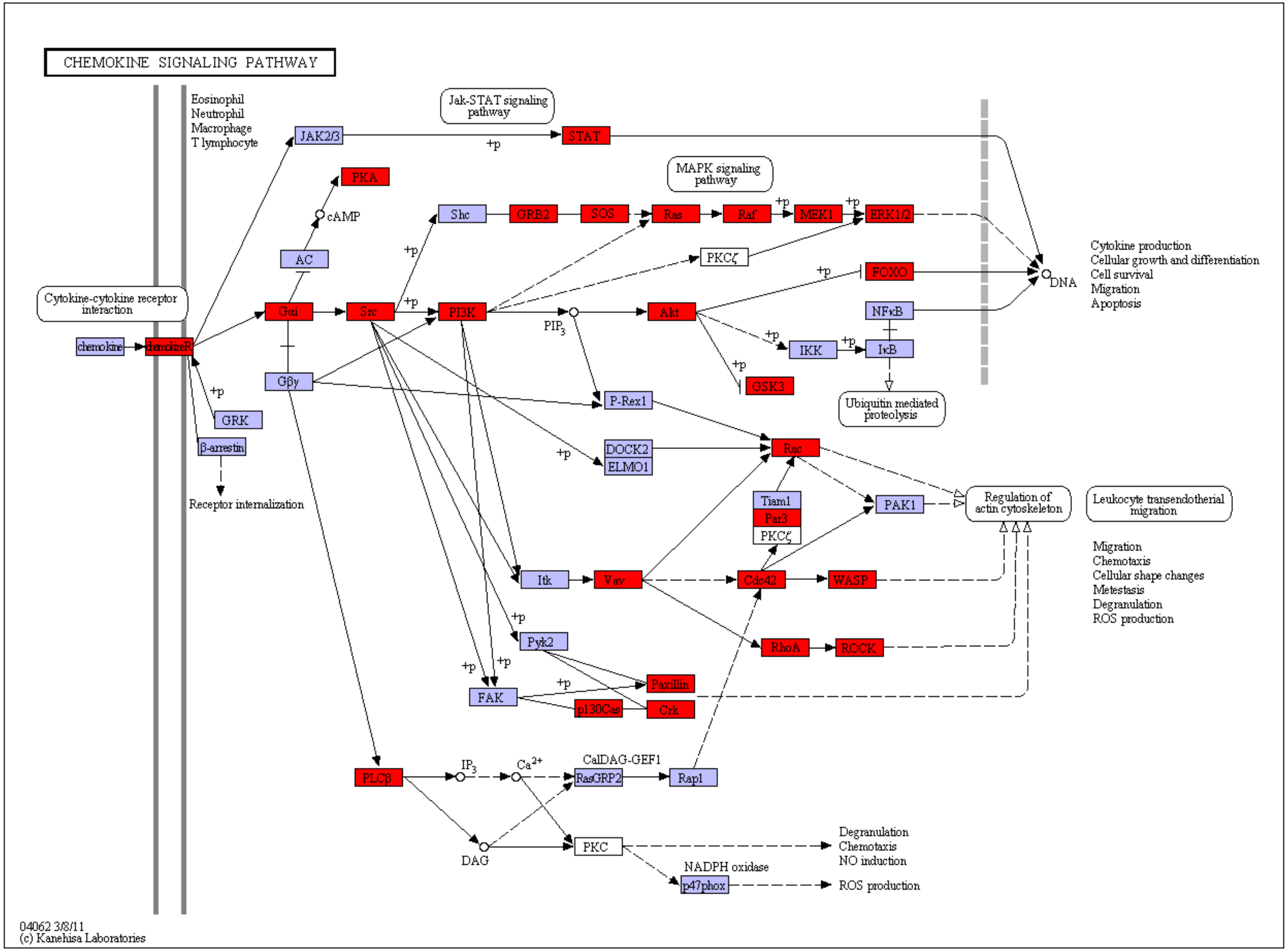

Figure 30: ko04062 Chemokine signaling pathway

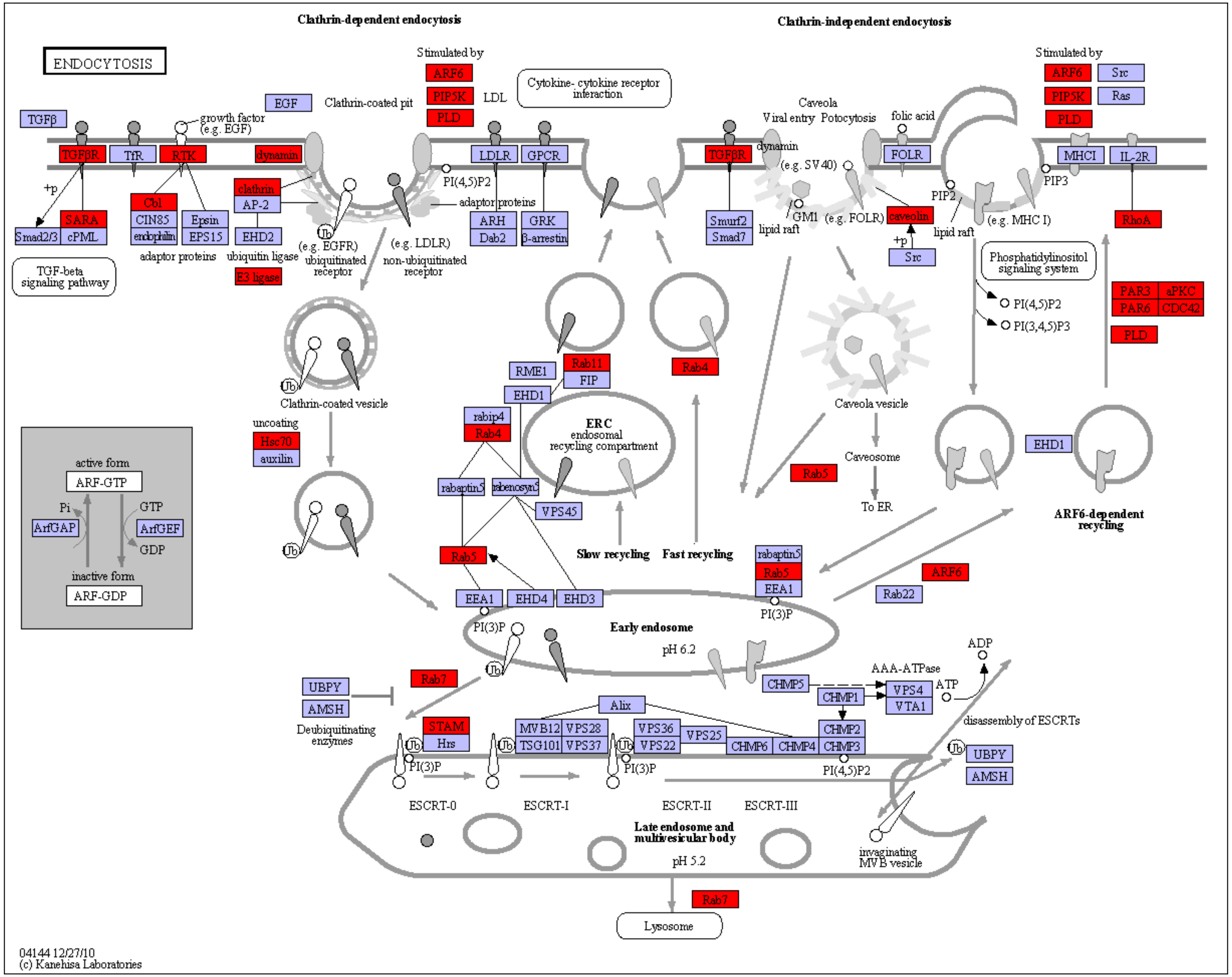

Figure 31: ko04144 Endocytosis

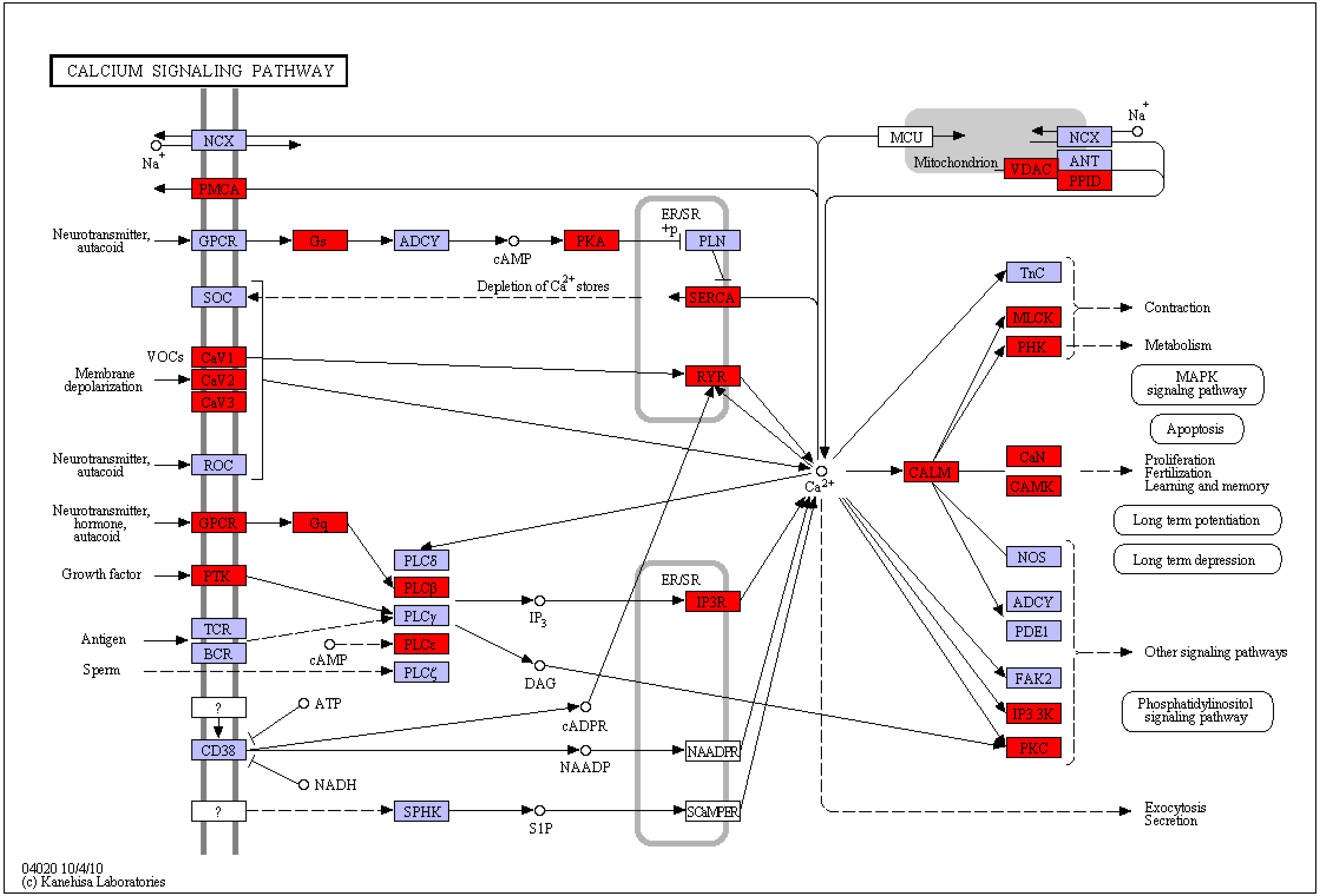

Figure 32: ko04020 Calcium signaling pathway

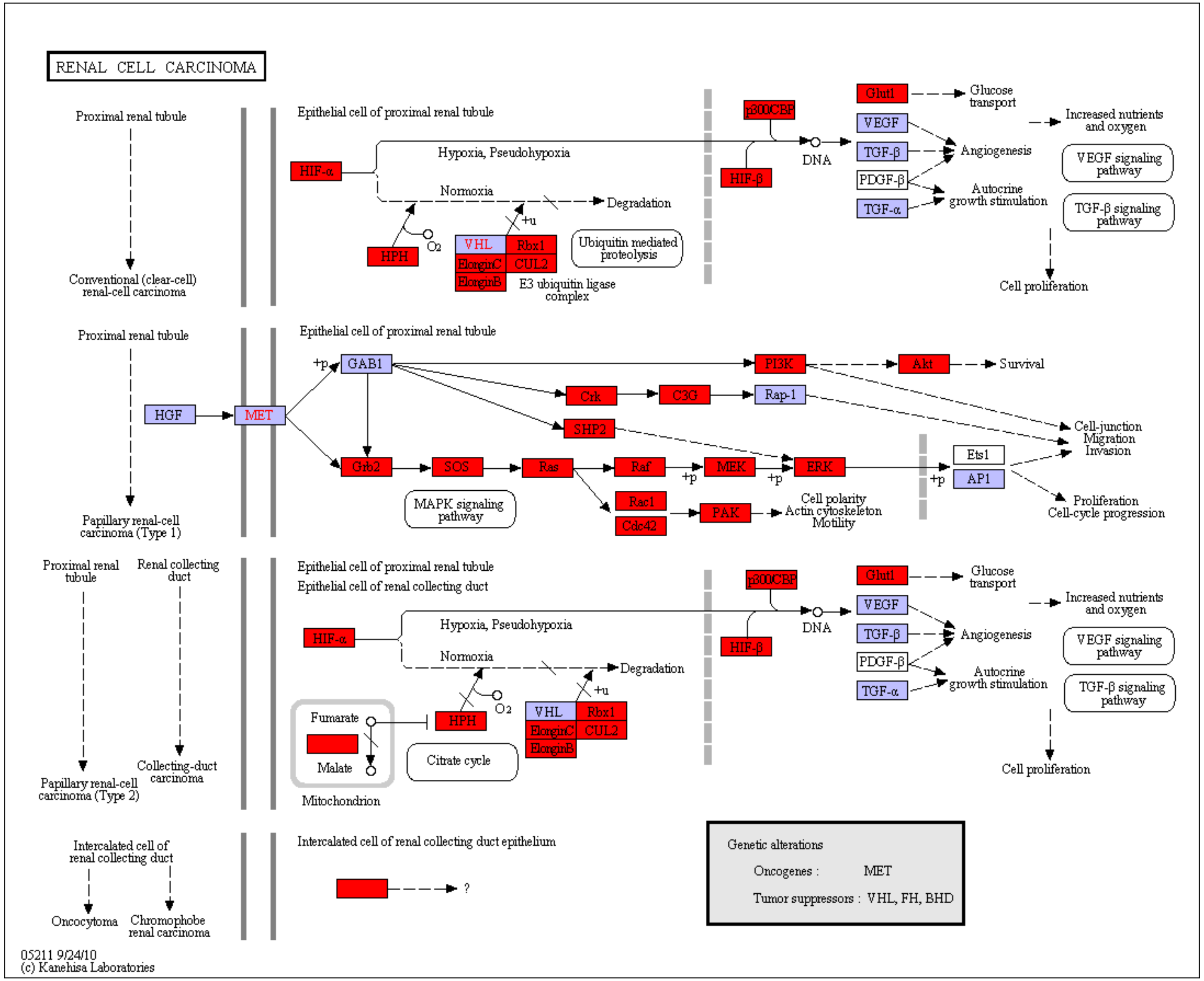

Figure 33: ko05211 Renal cell carcinoma

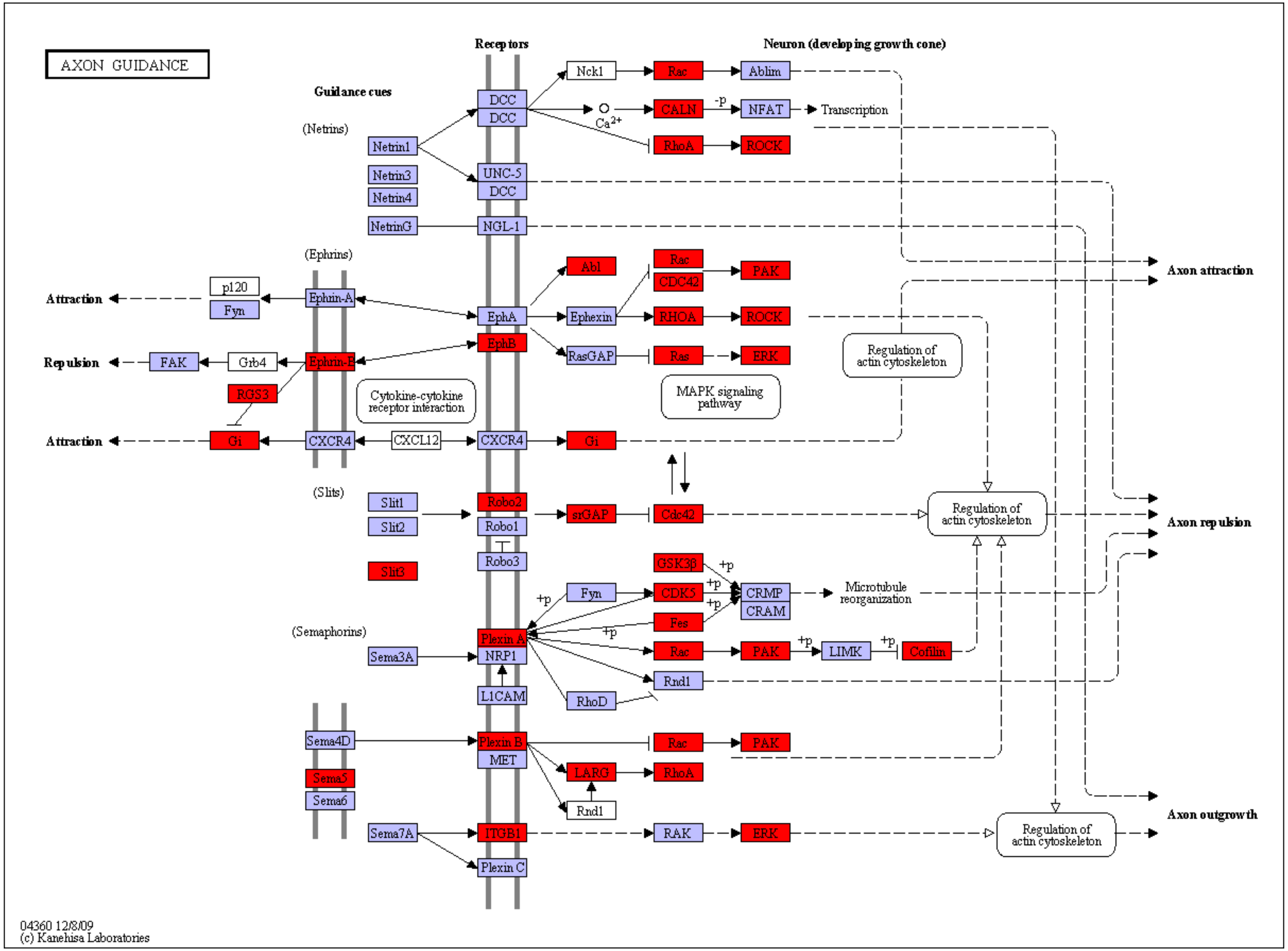

Figure 34: ko04360 Axon guidance

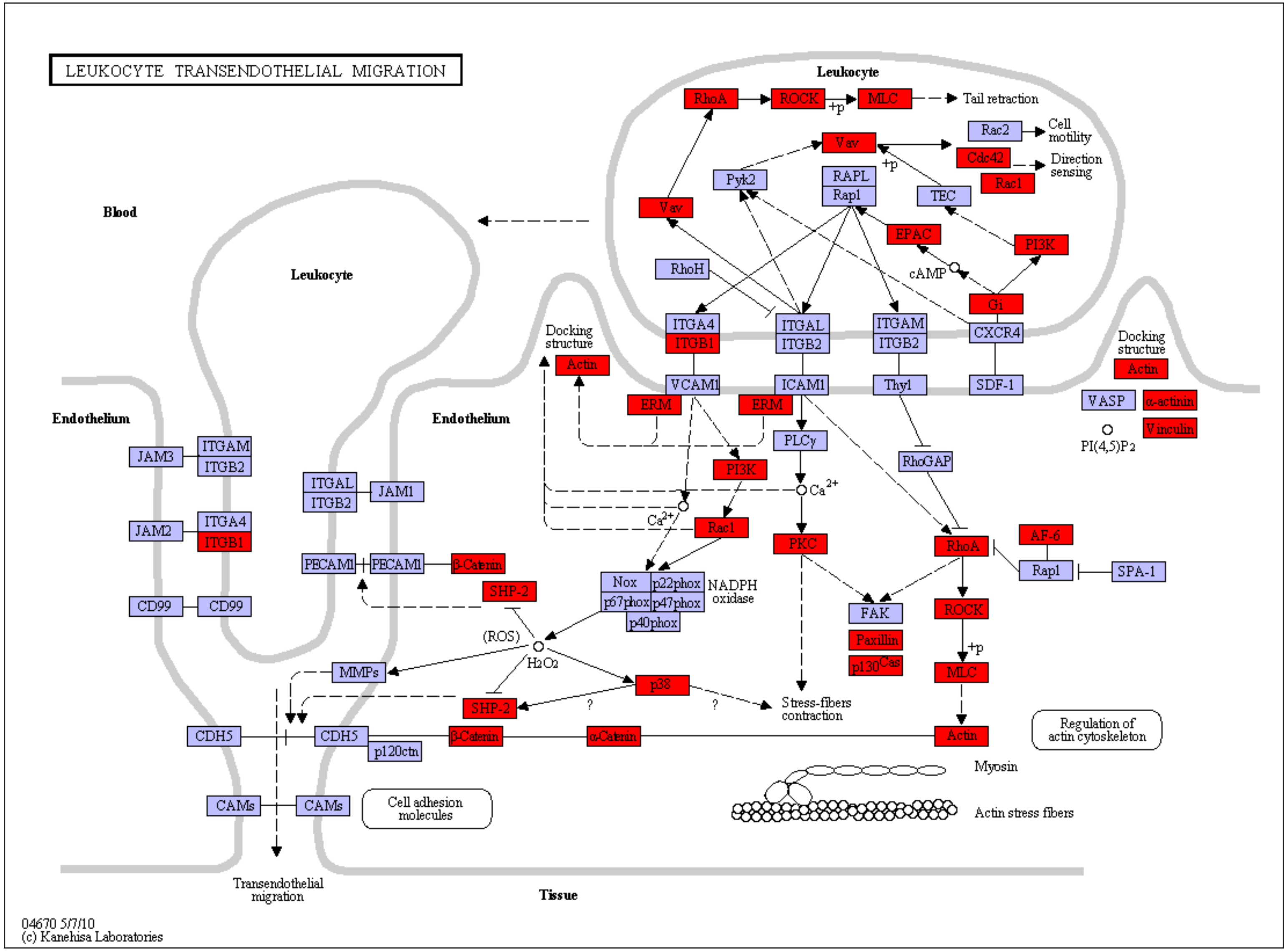

Figure 35: ko04670 Leukocyte transendothelial migration

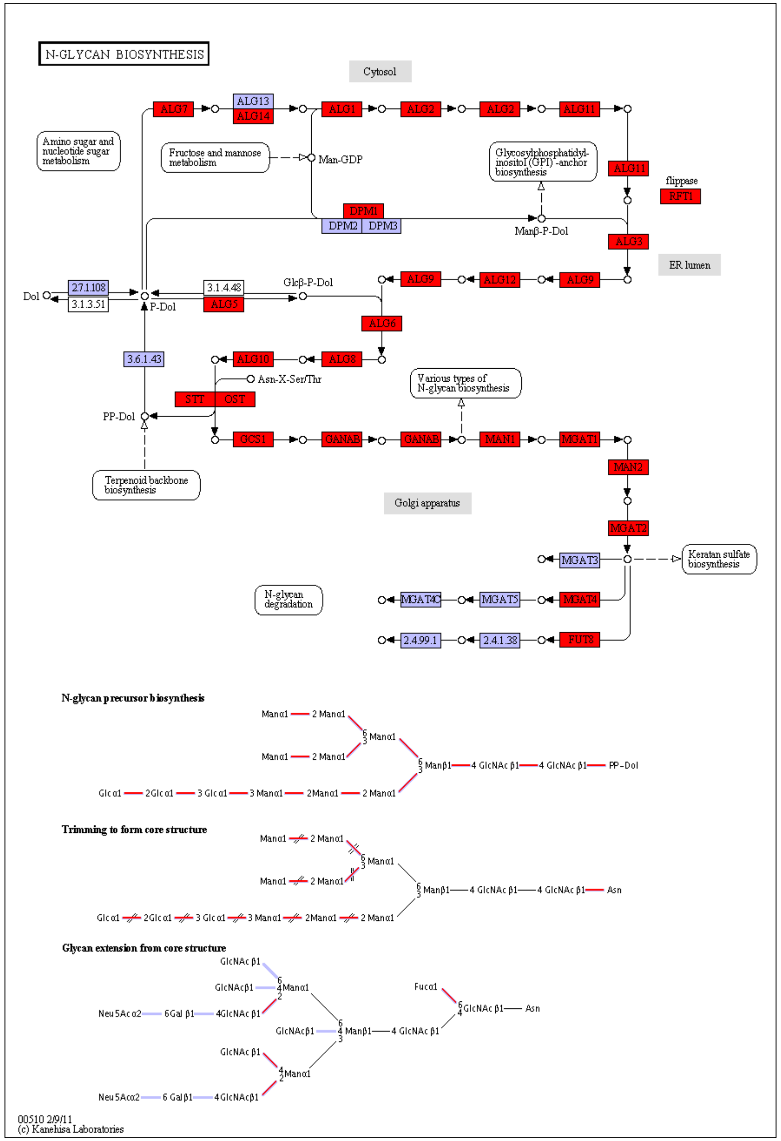

Figure 36: ko00510 N-Glycan biosynthesis

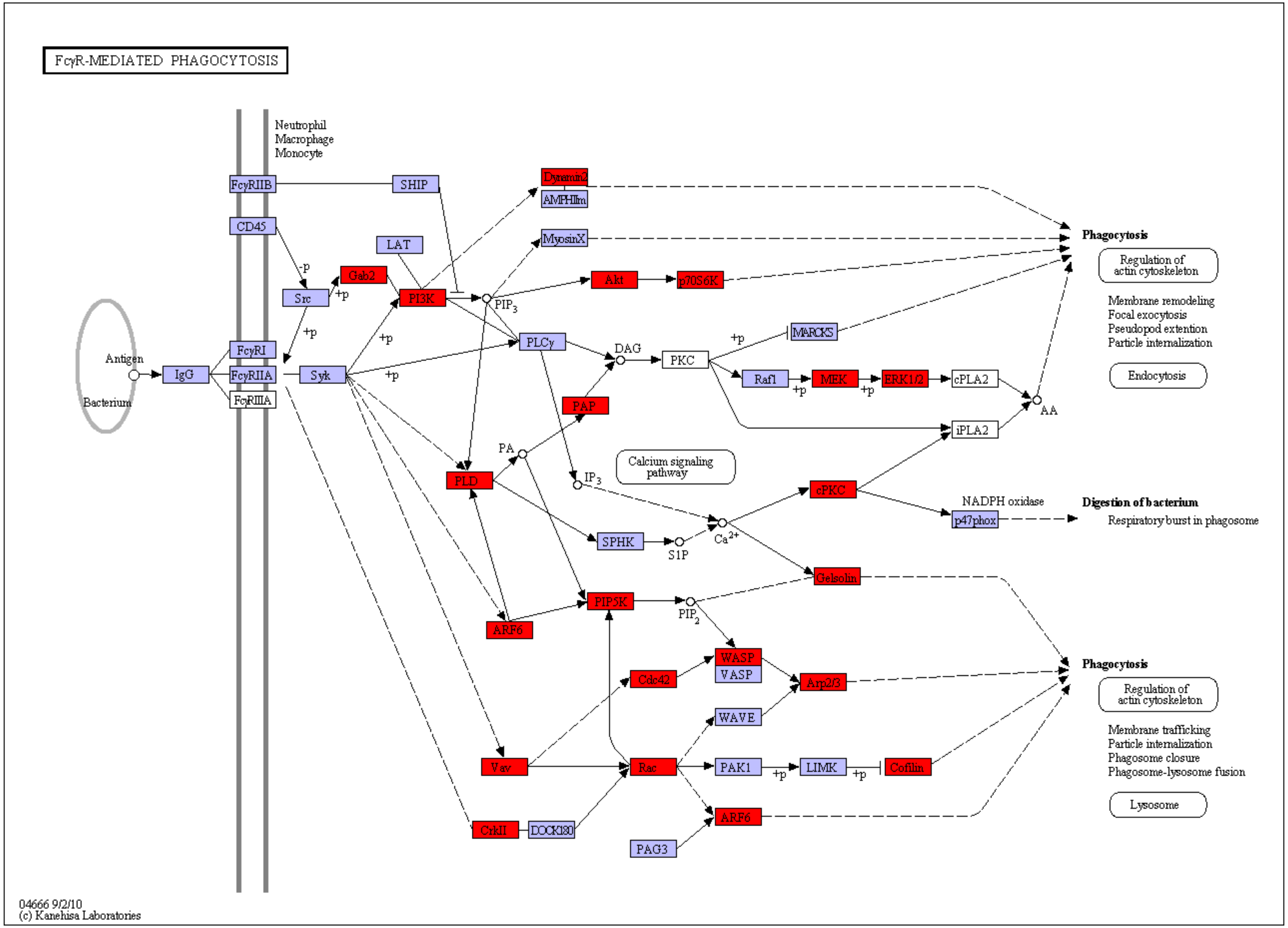

Figure 37: ko04666 Fc gamma R-mediated phagocytosis

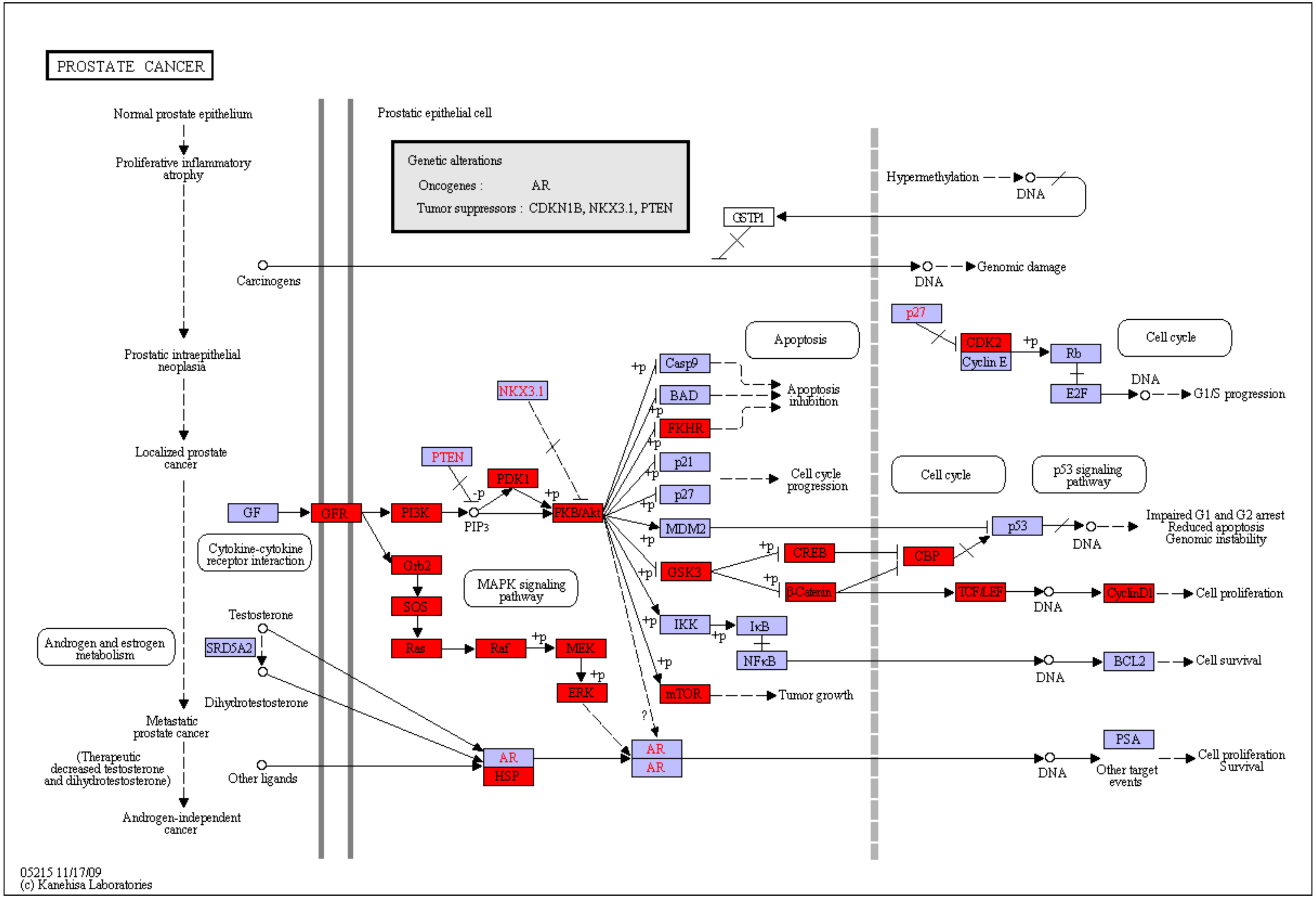

Figure 38: ko05215 Prostate cancer

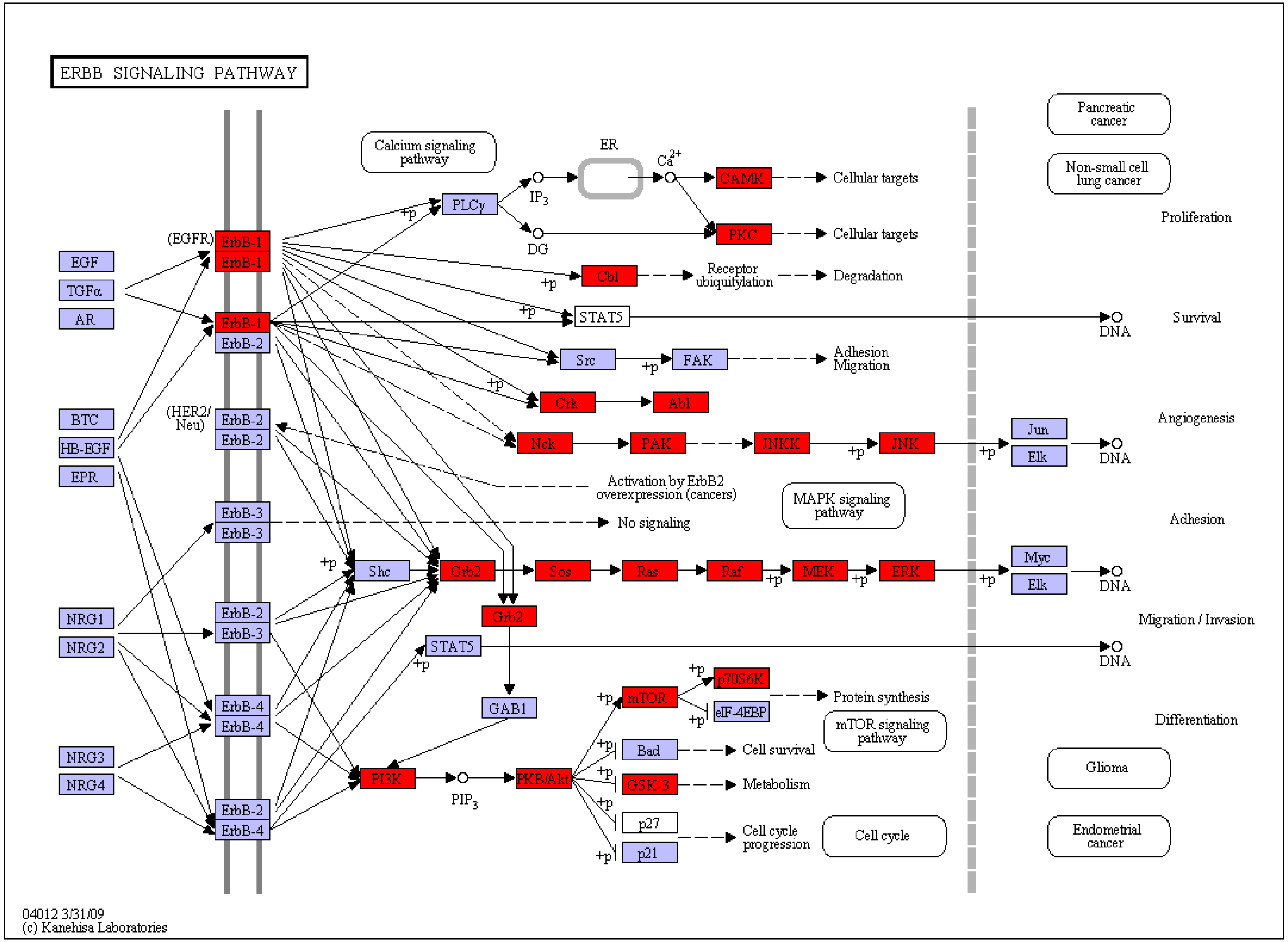

Figure 39: ko04012 ErbB signaling pathway

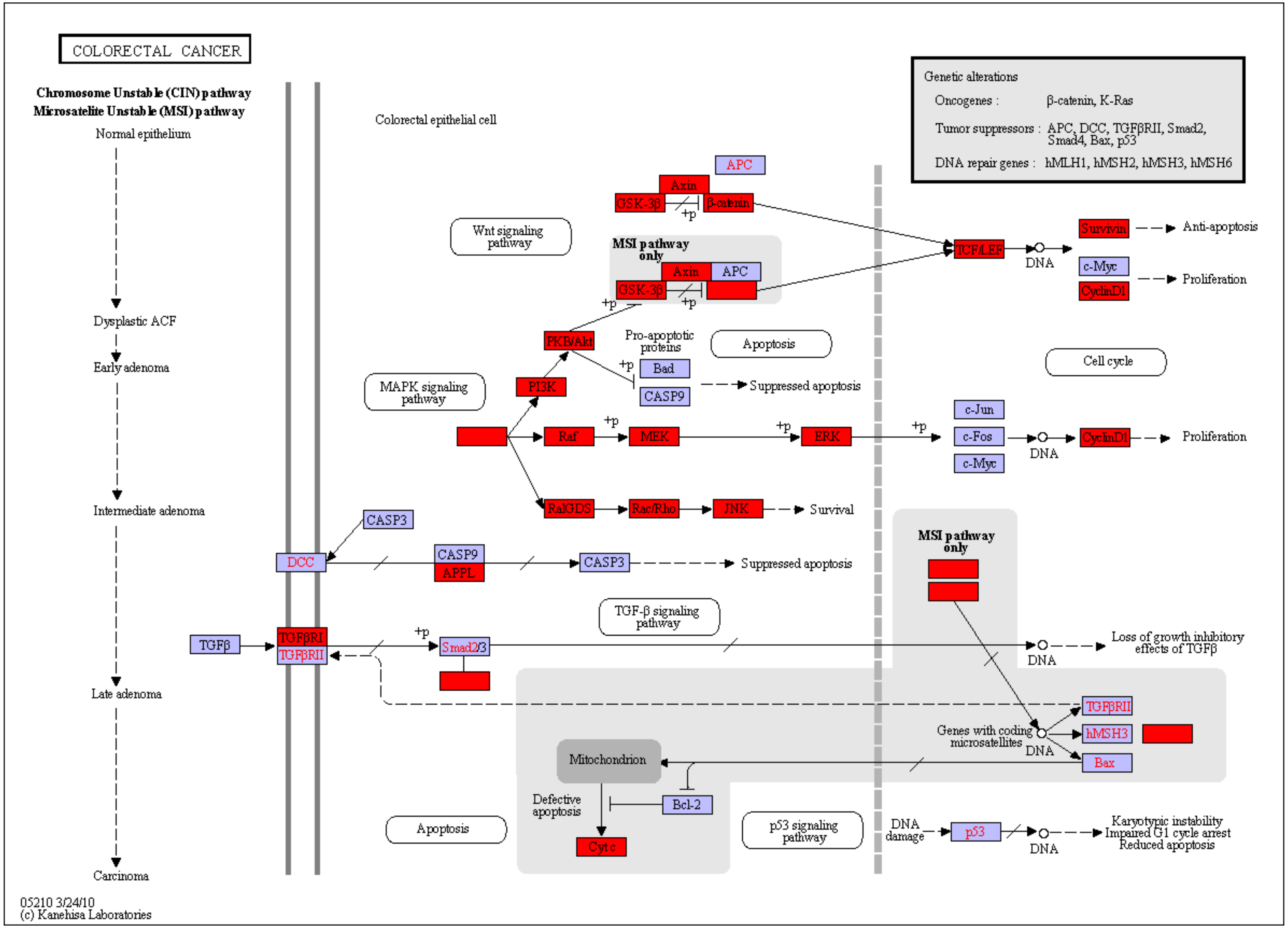

Figure 40: ko05210 Colorectal cancer

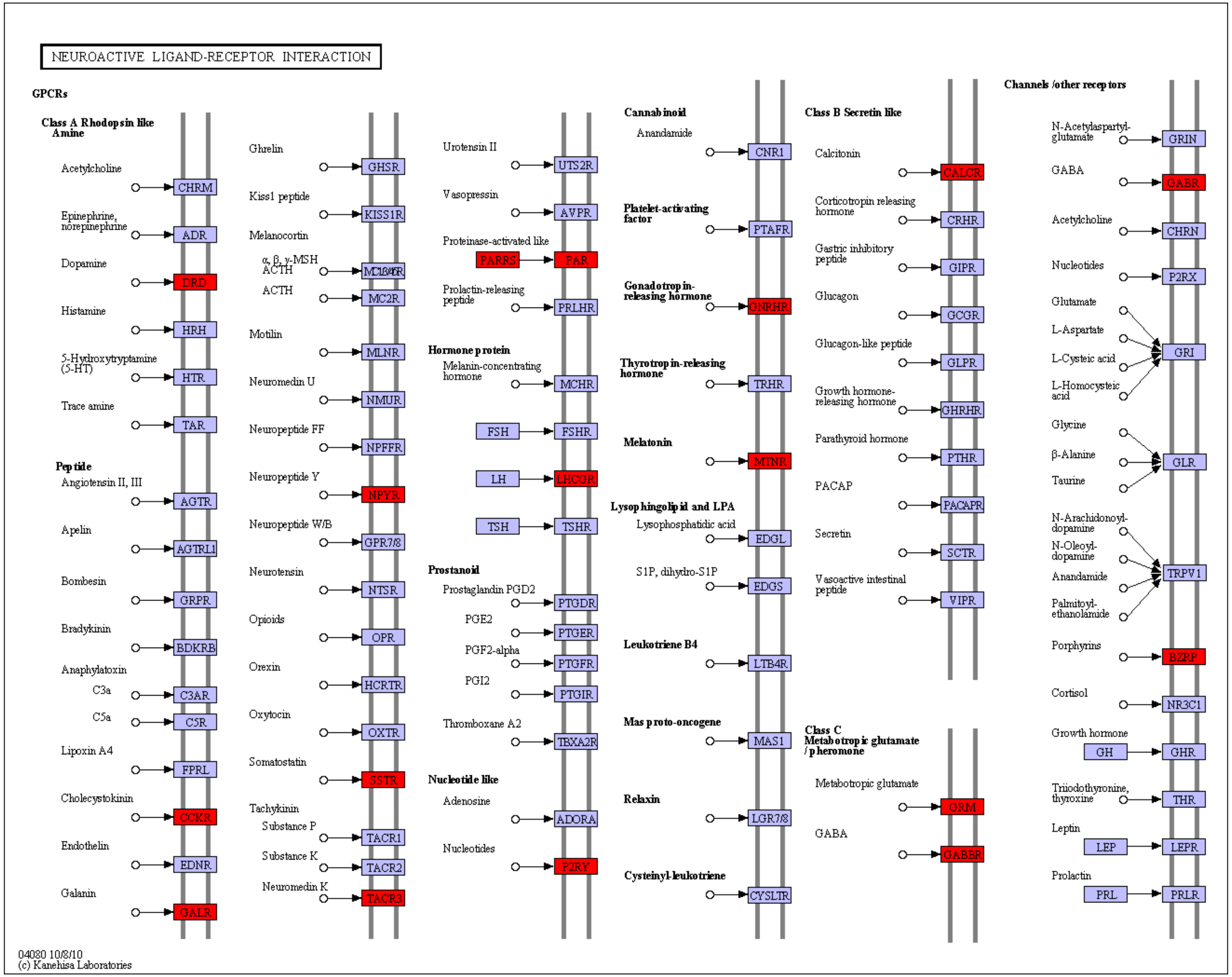

Figure 41: ko04080 Neuroactive ligand-receptor interaction

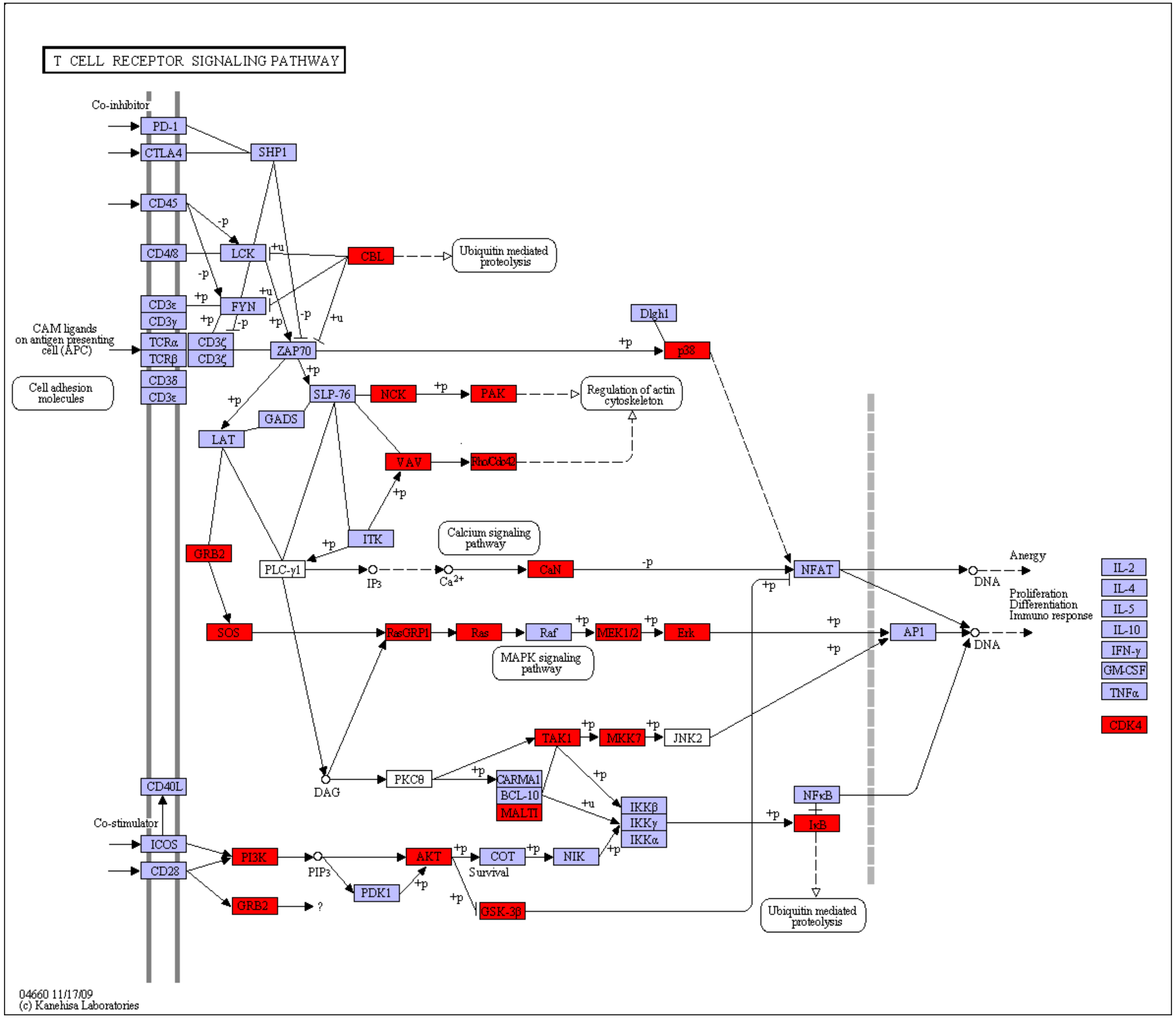

Figure 42: ko04660 T cell receptor signaling pathway

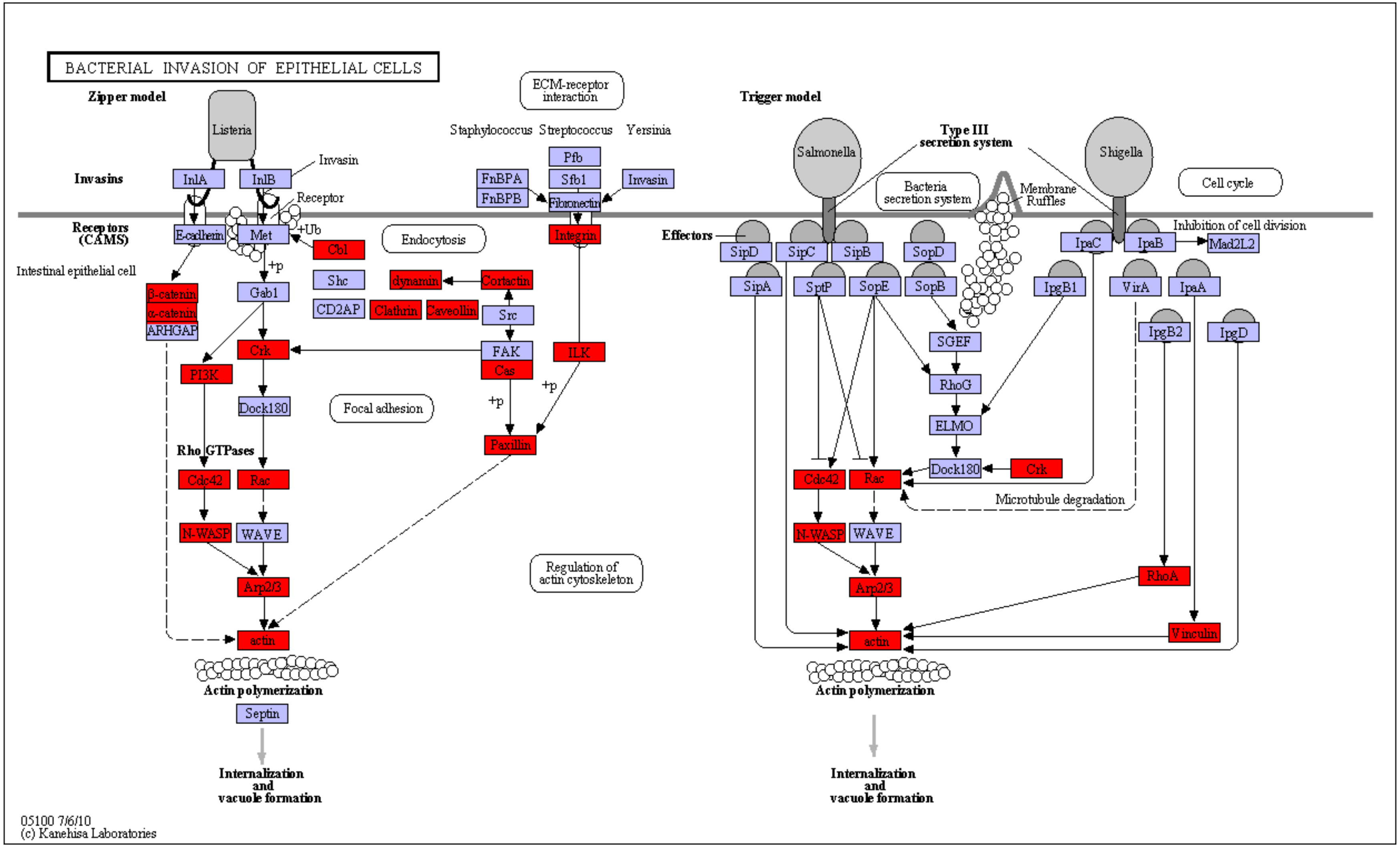

Figure 43: ko05100 Bacterial invasion of epithelial cells

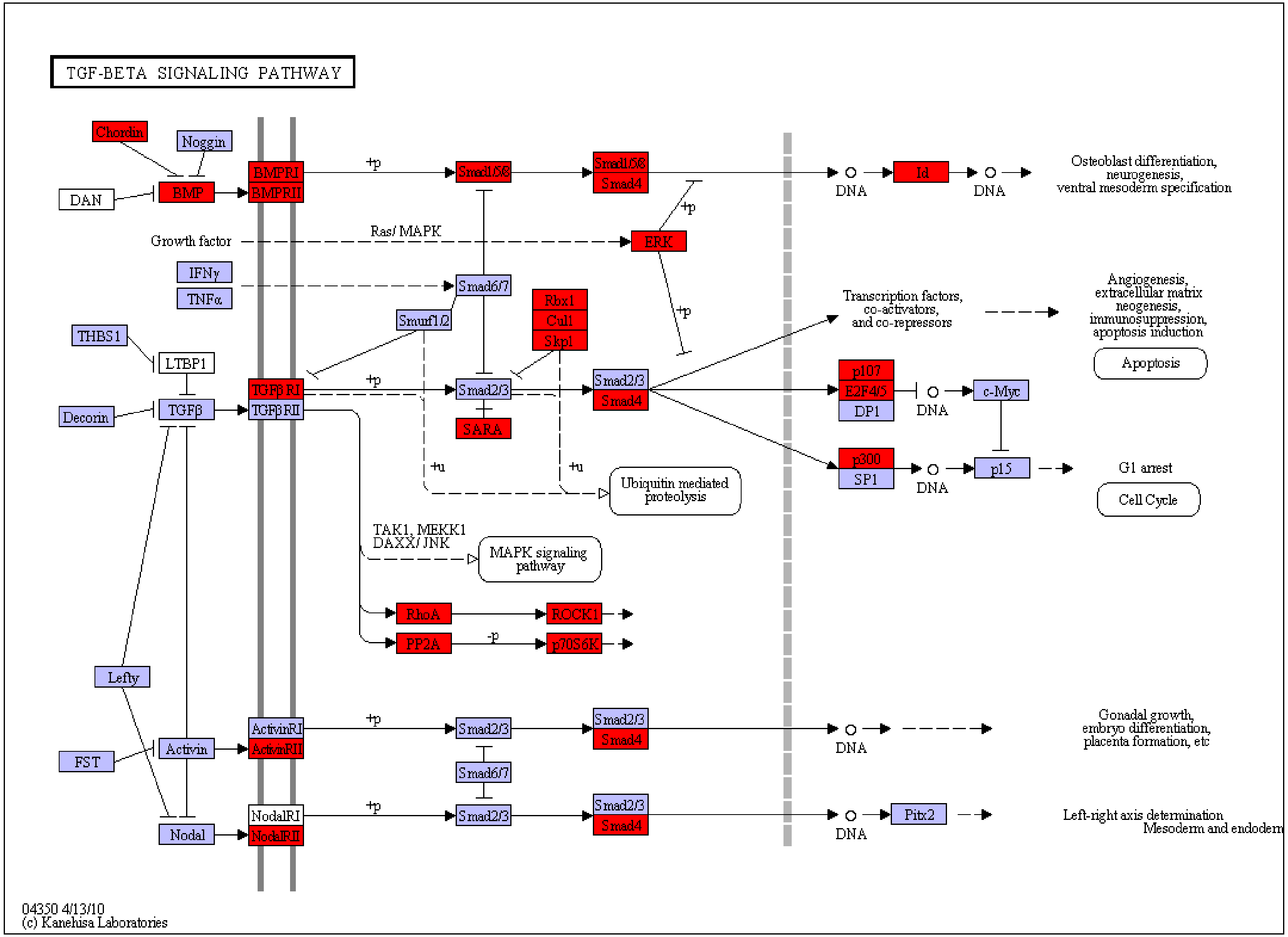

Figure 44: ko04350 TGF-beta signaling pathway

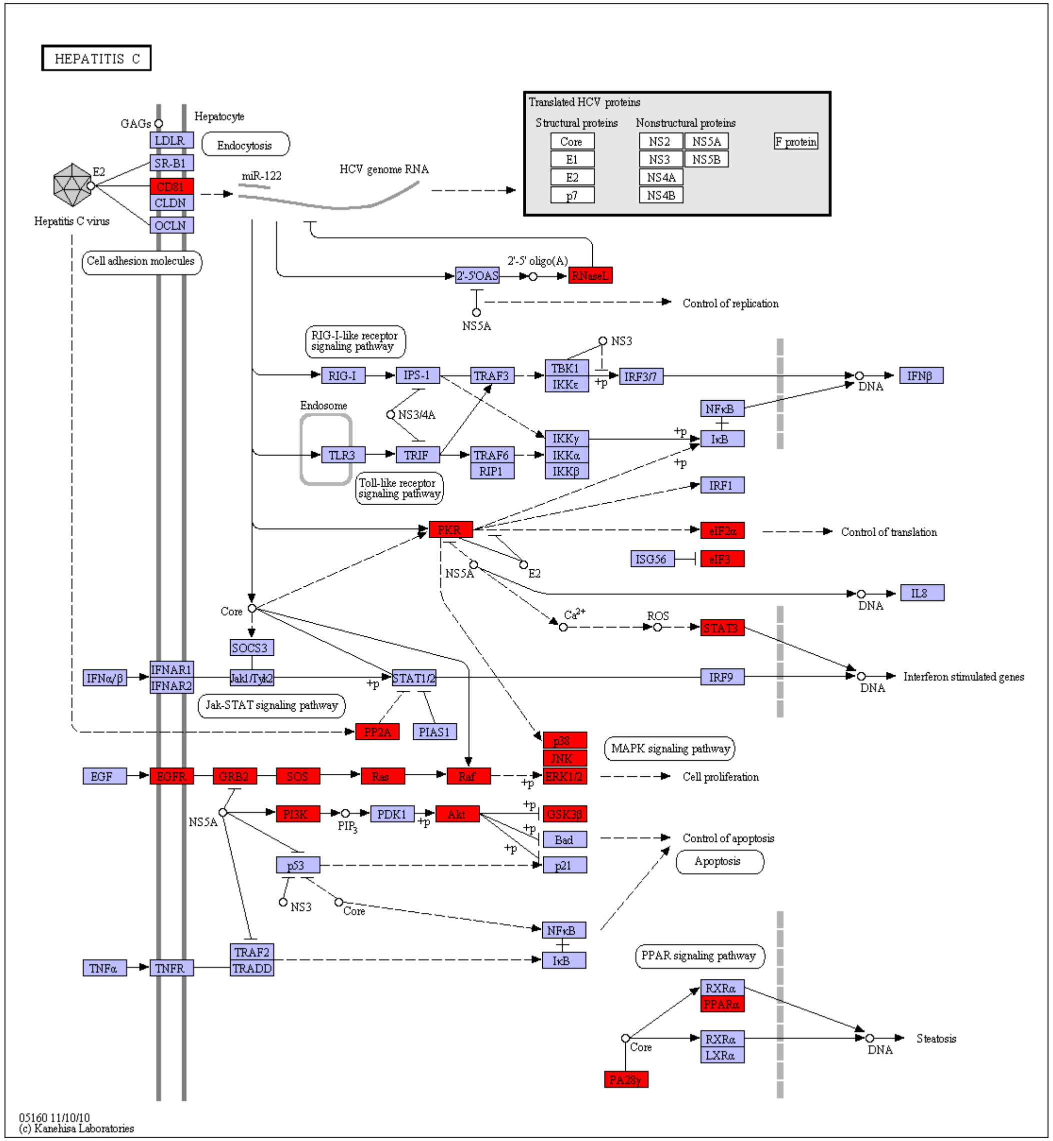

Figure 45: ko05160 Hepatitis C

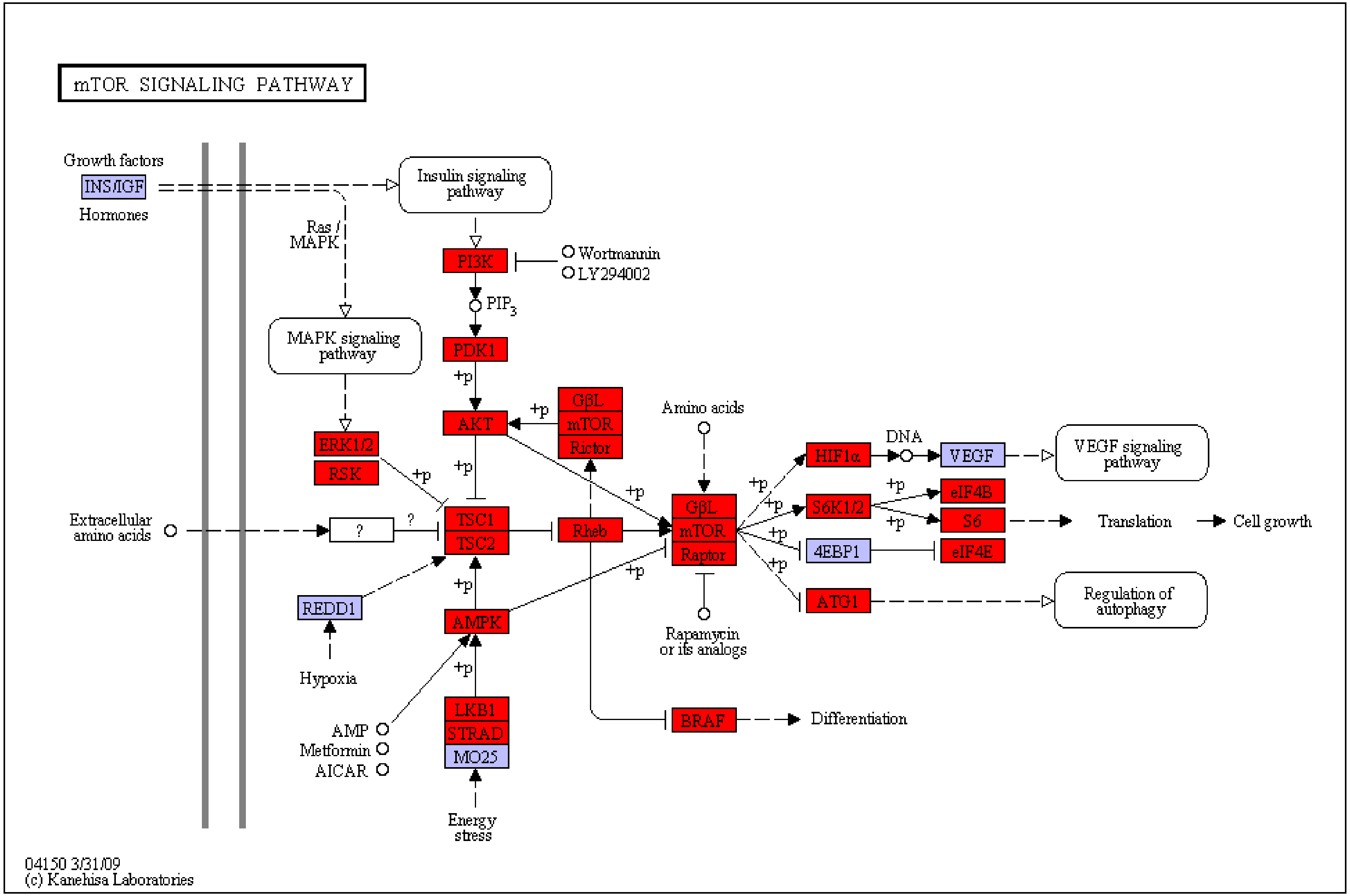

Figure 46: ko04150 mTOR signaling pathway

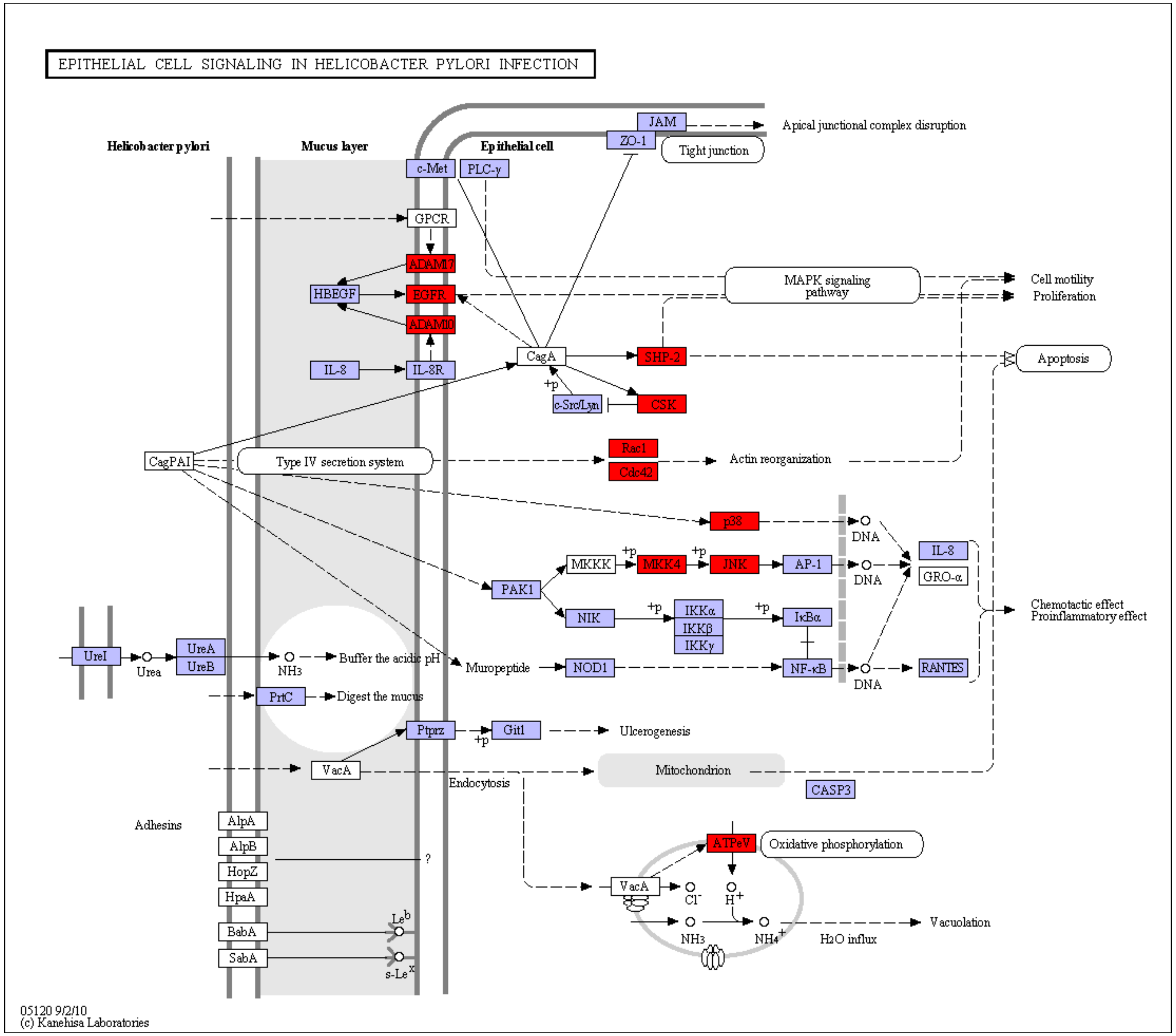

Figure 47: ko05120 Epithelial cell signaling in Helicobacter pylori infection

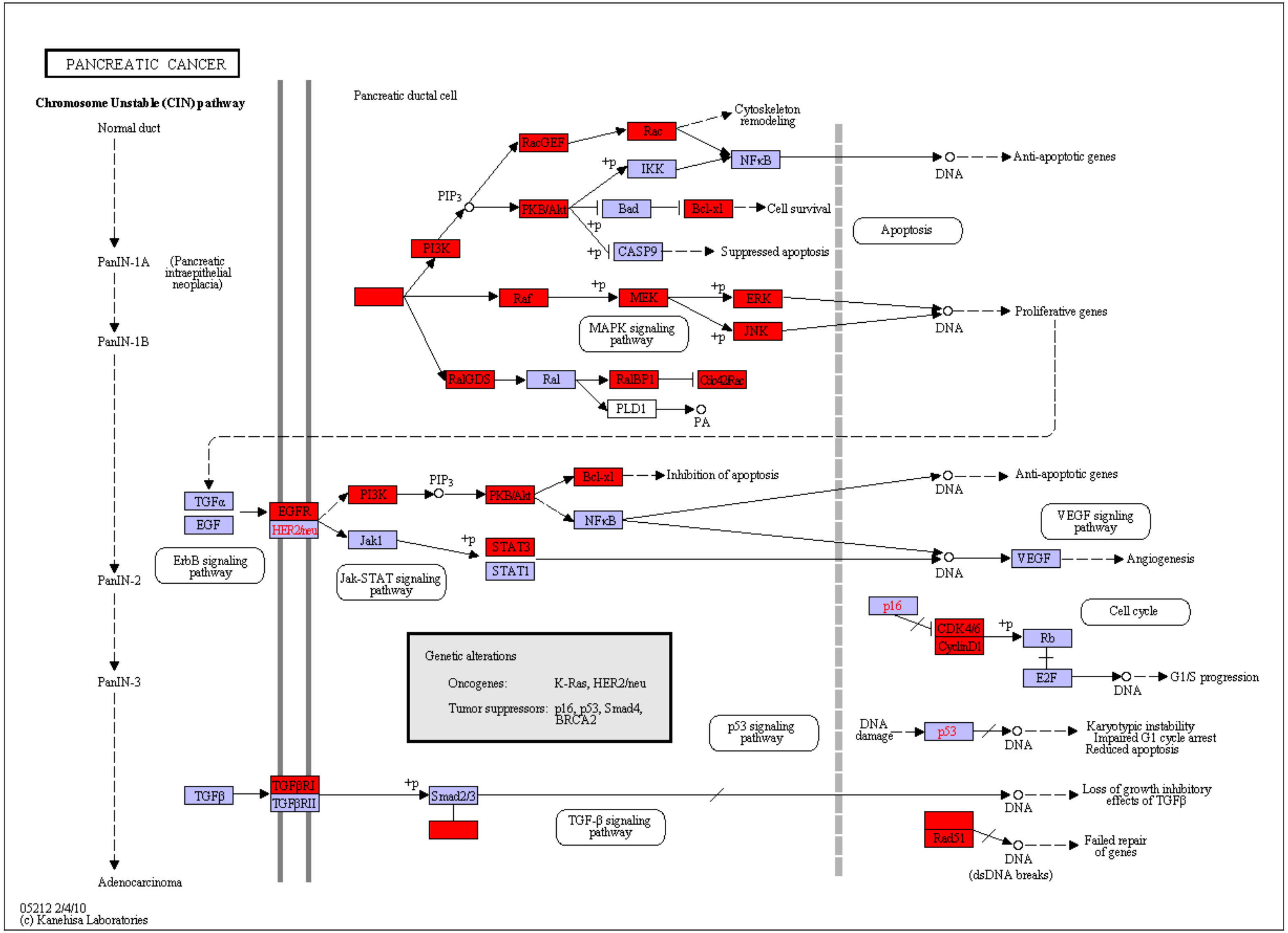

Figure 48: ko05212 Pancreatic cancer

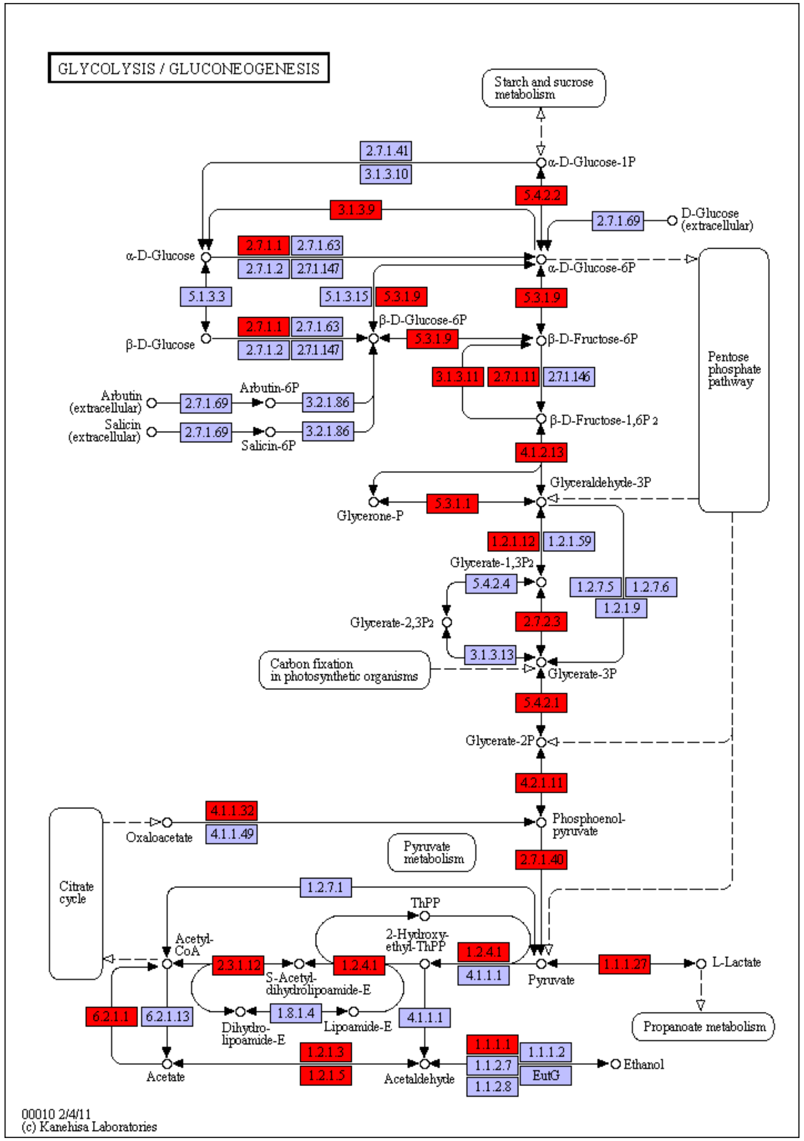

Figure 49: ko00010 Glycolysis / Gluconeogenesis

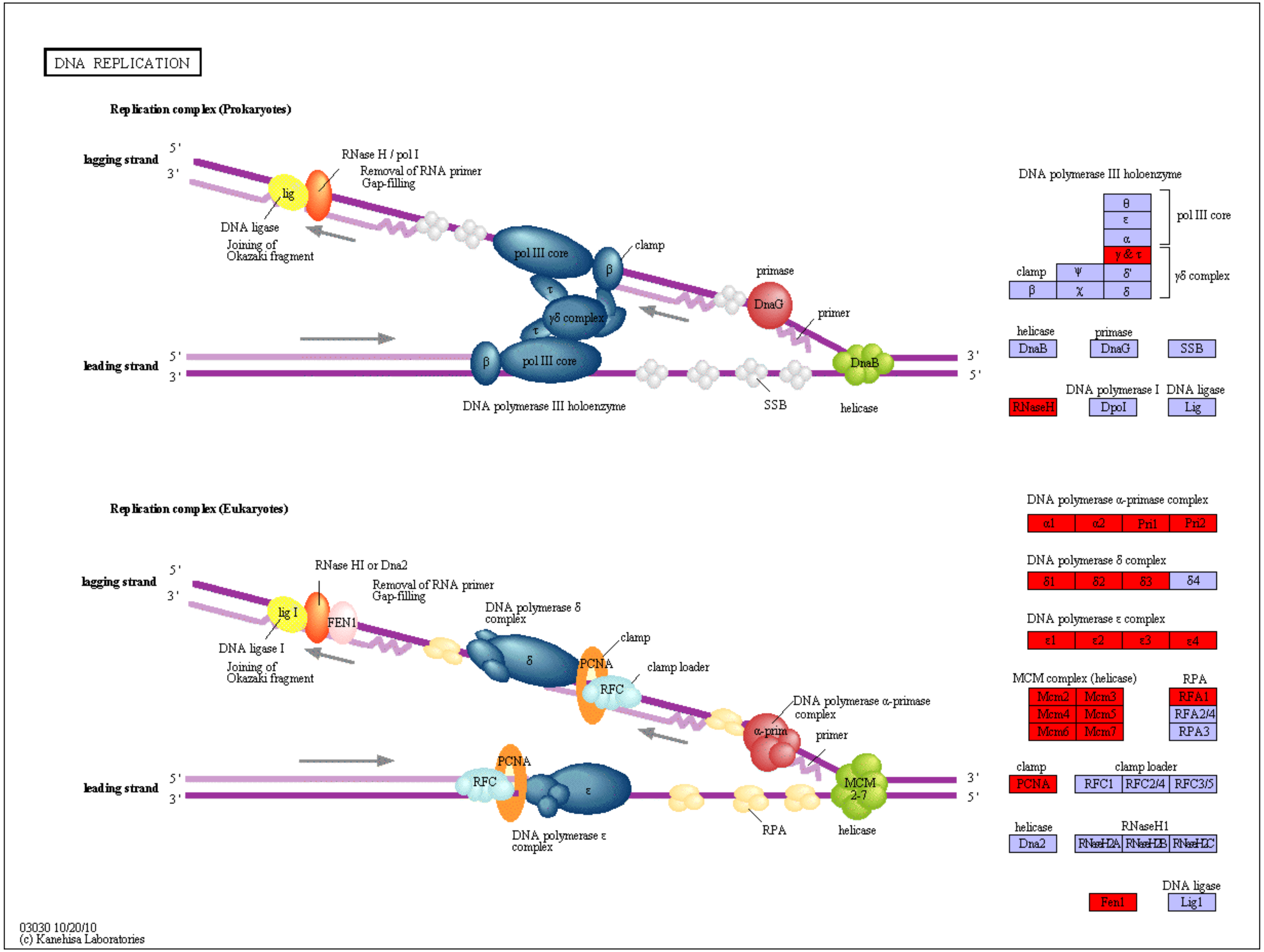

Figure 50: ko03030 DNA replication

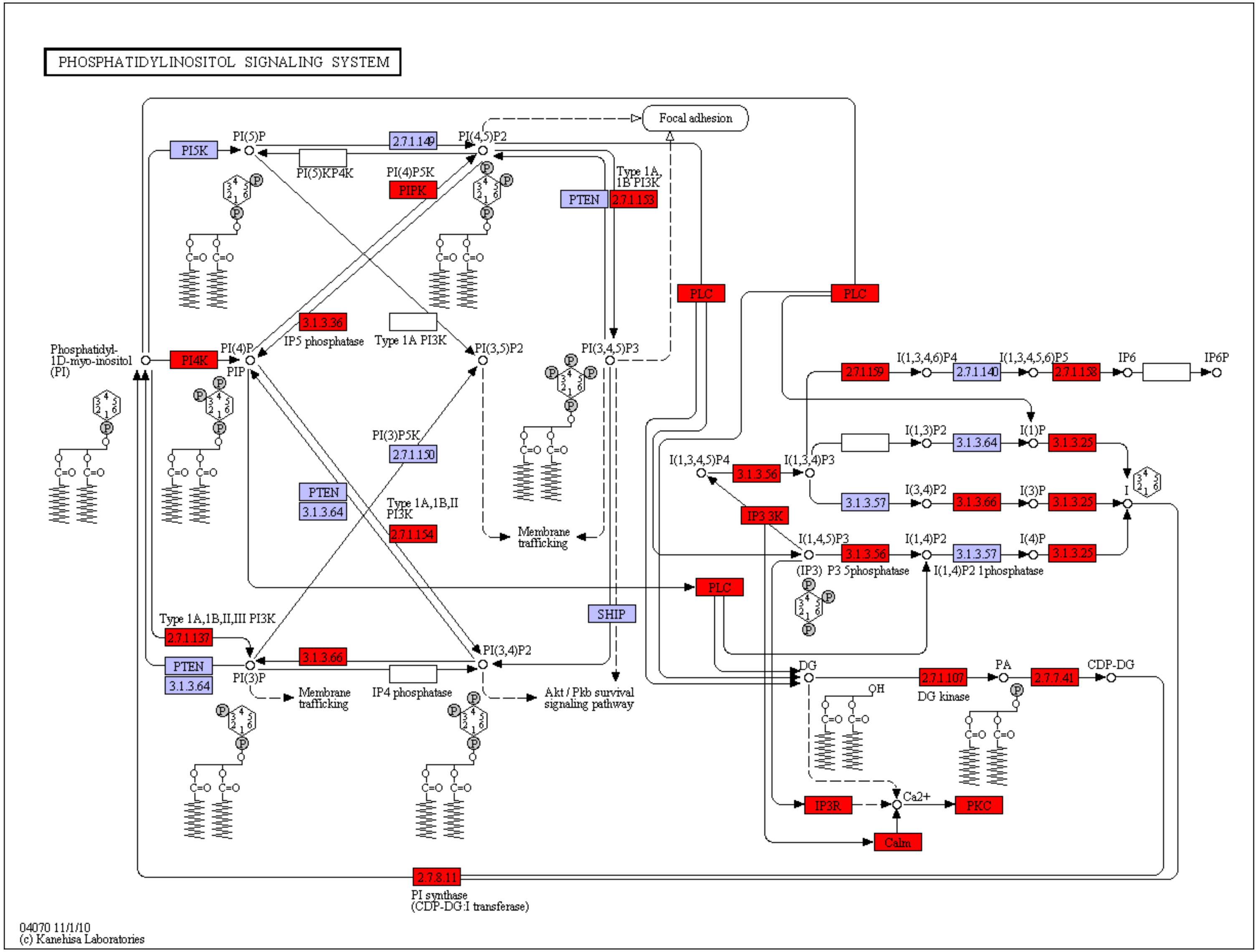

Figure 51: ko04070 Phosphatidylinositol signaling system

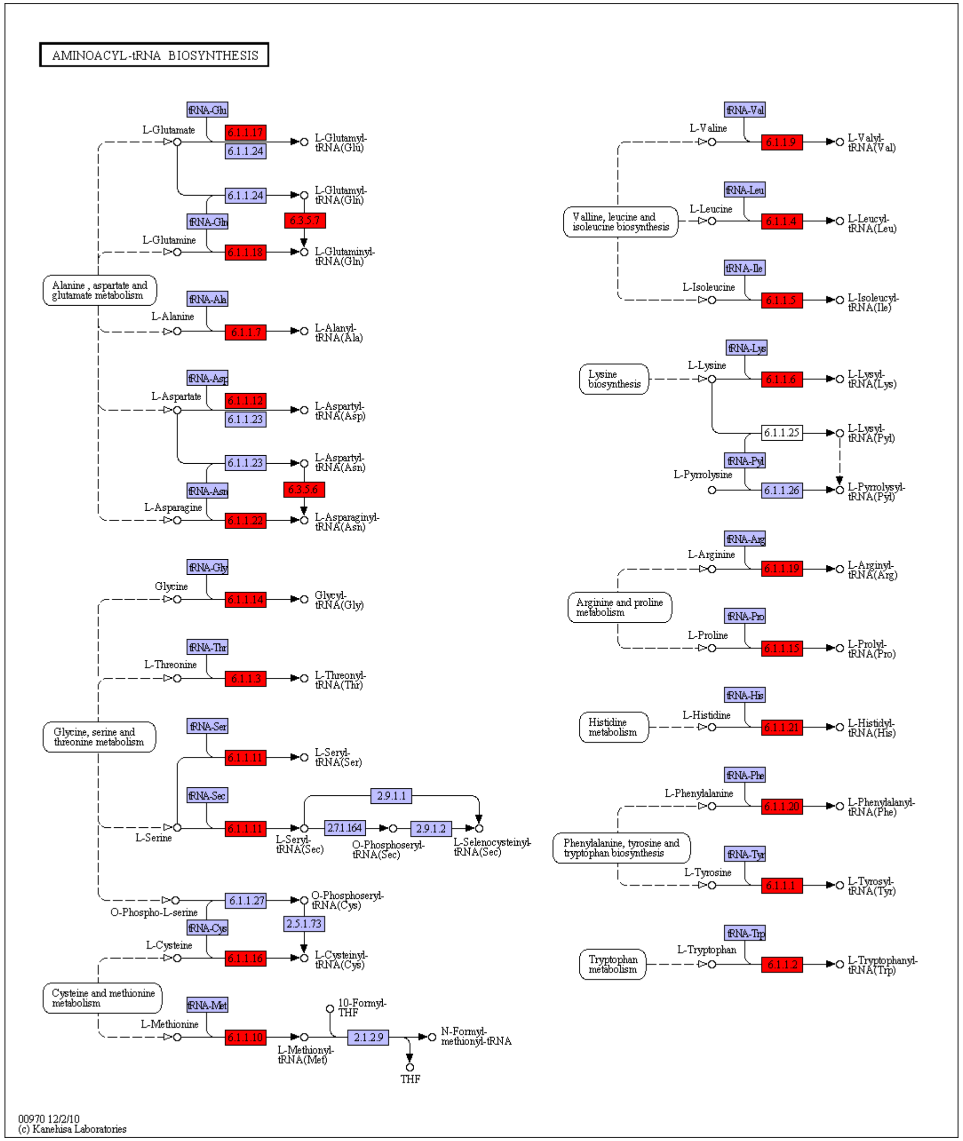

Figure 52: ko00970 Aminoacyl-tRNA biosynthesis

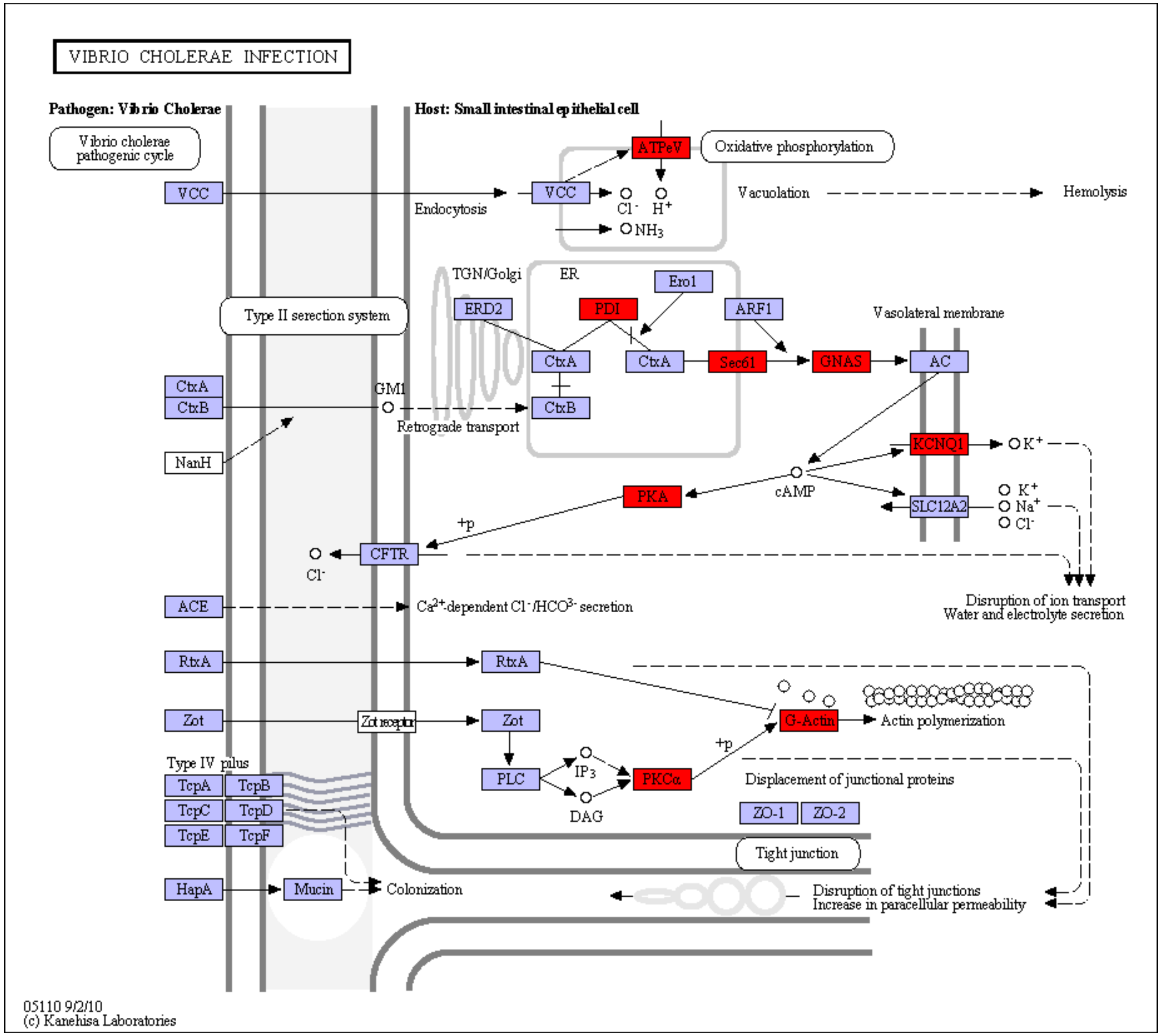

Figure 53: ko05110 Vibrio cholerae infection

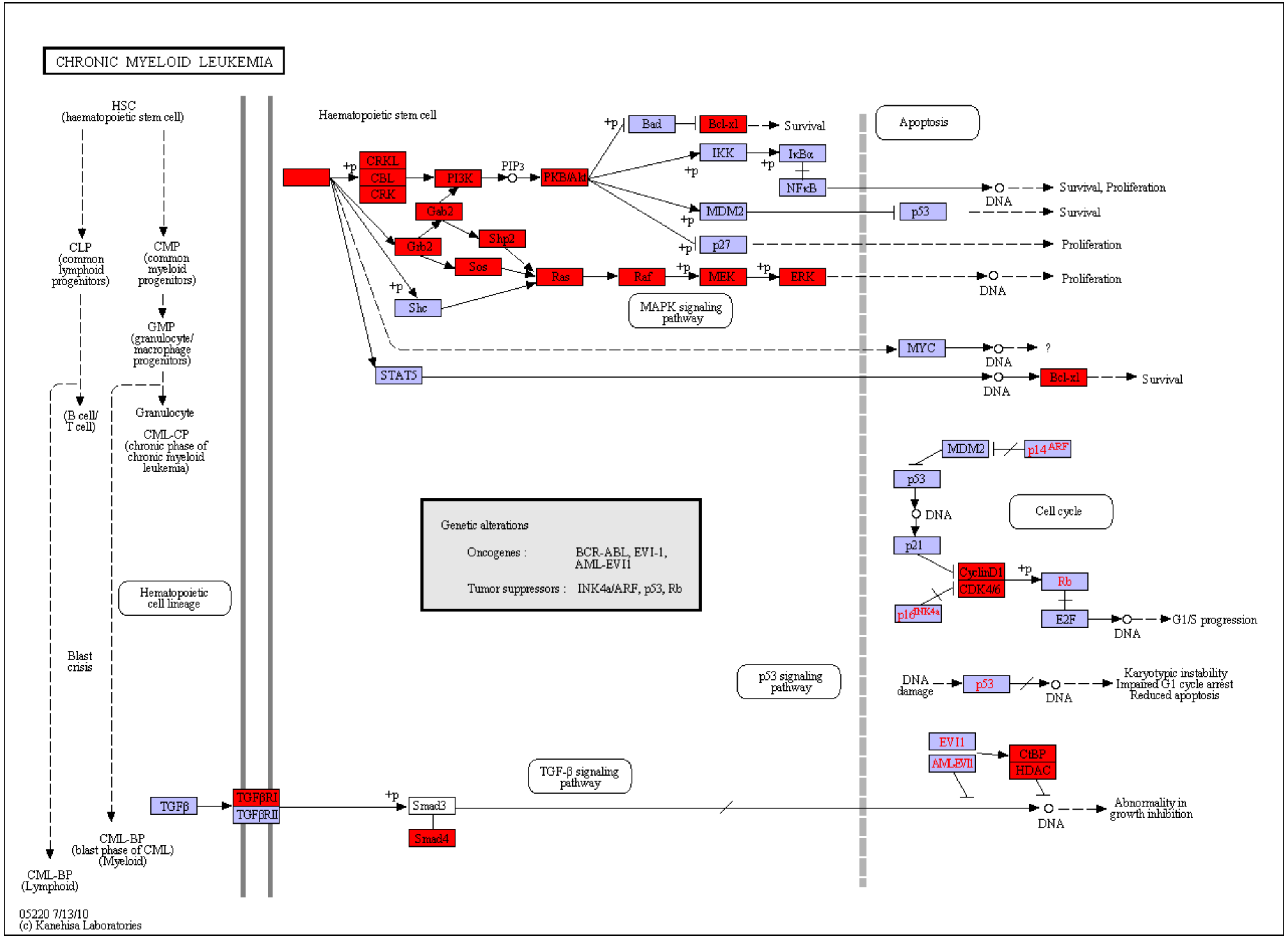

Figure 54: ko05220 Chronic myeloid leukemia

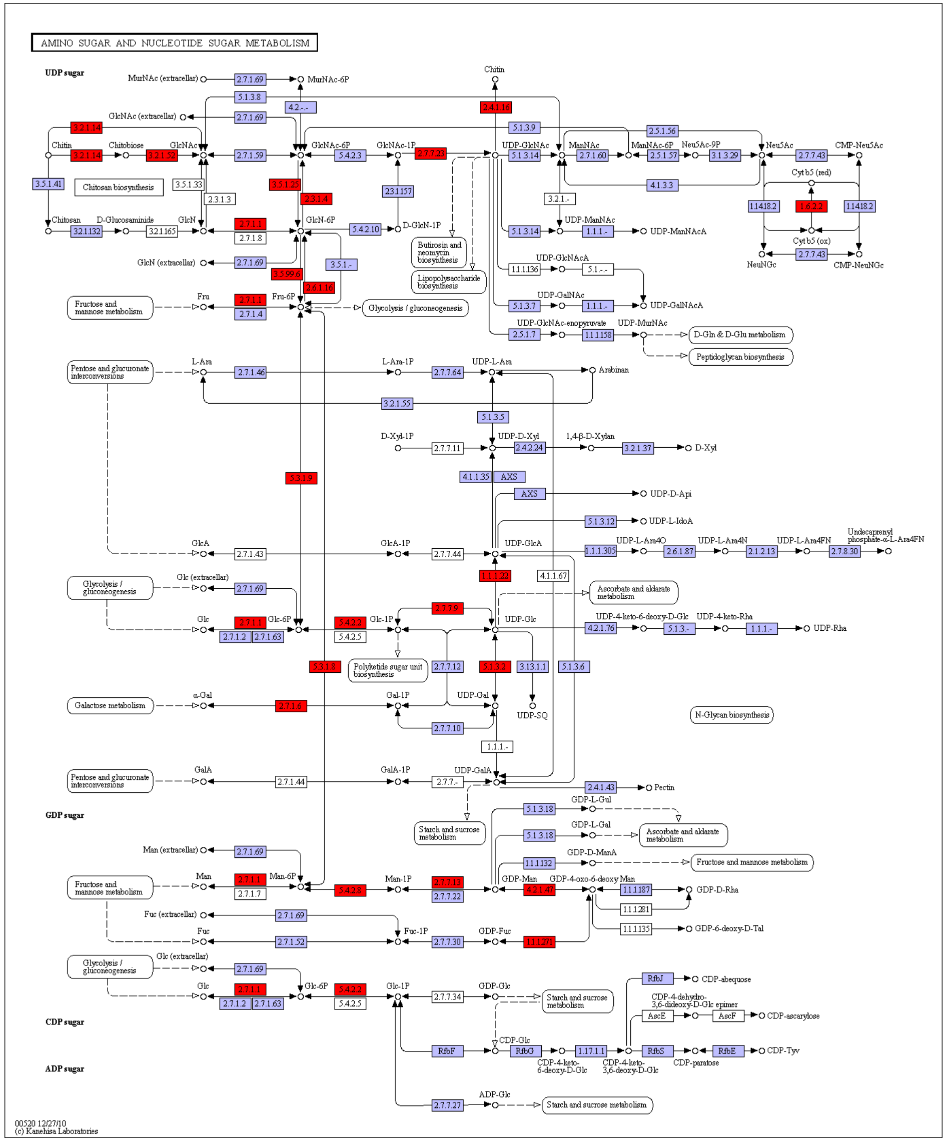

Figure 55: ko00520 Amino sugar and nucleotide sugar metabolism

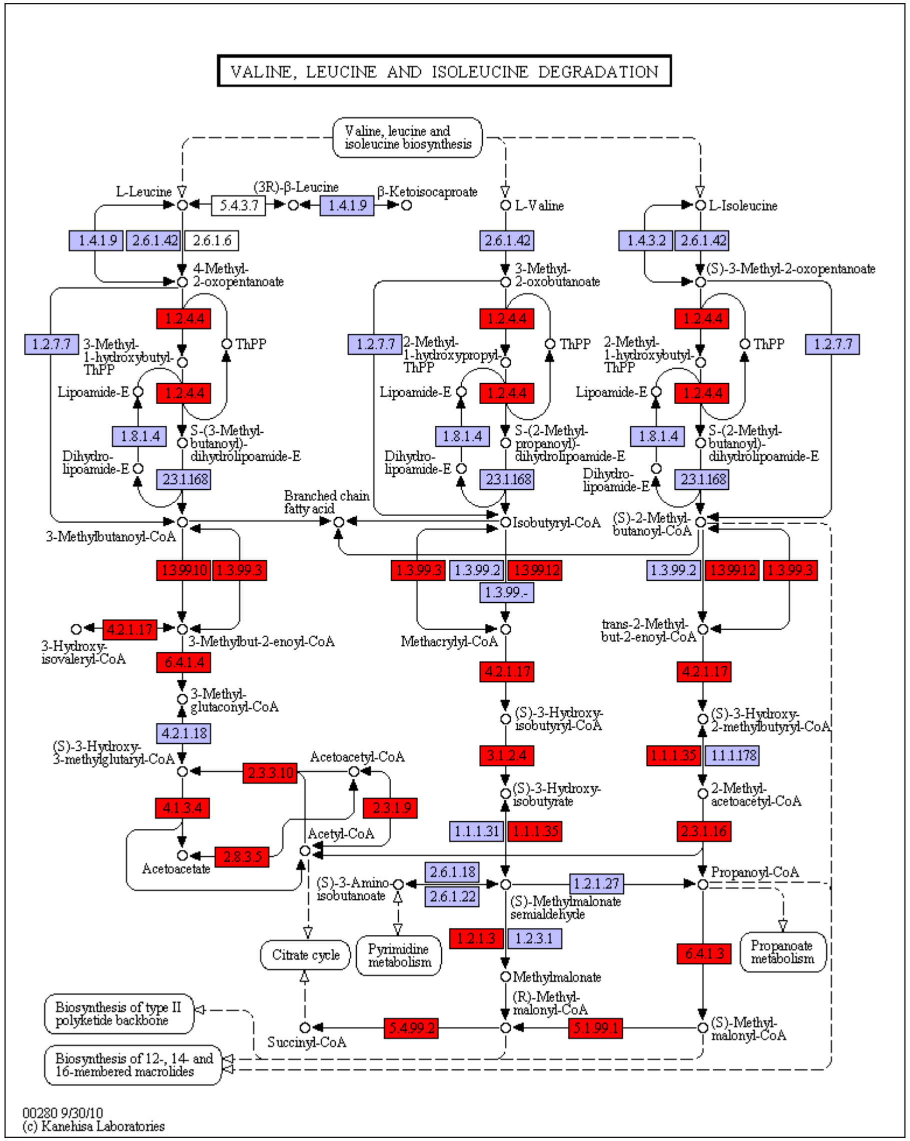

Figure 56: ko00280 Valine, leucine and isoleucine degradation

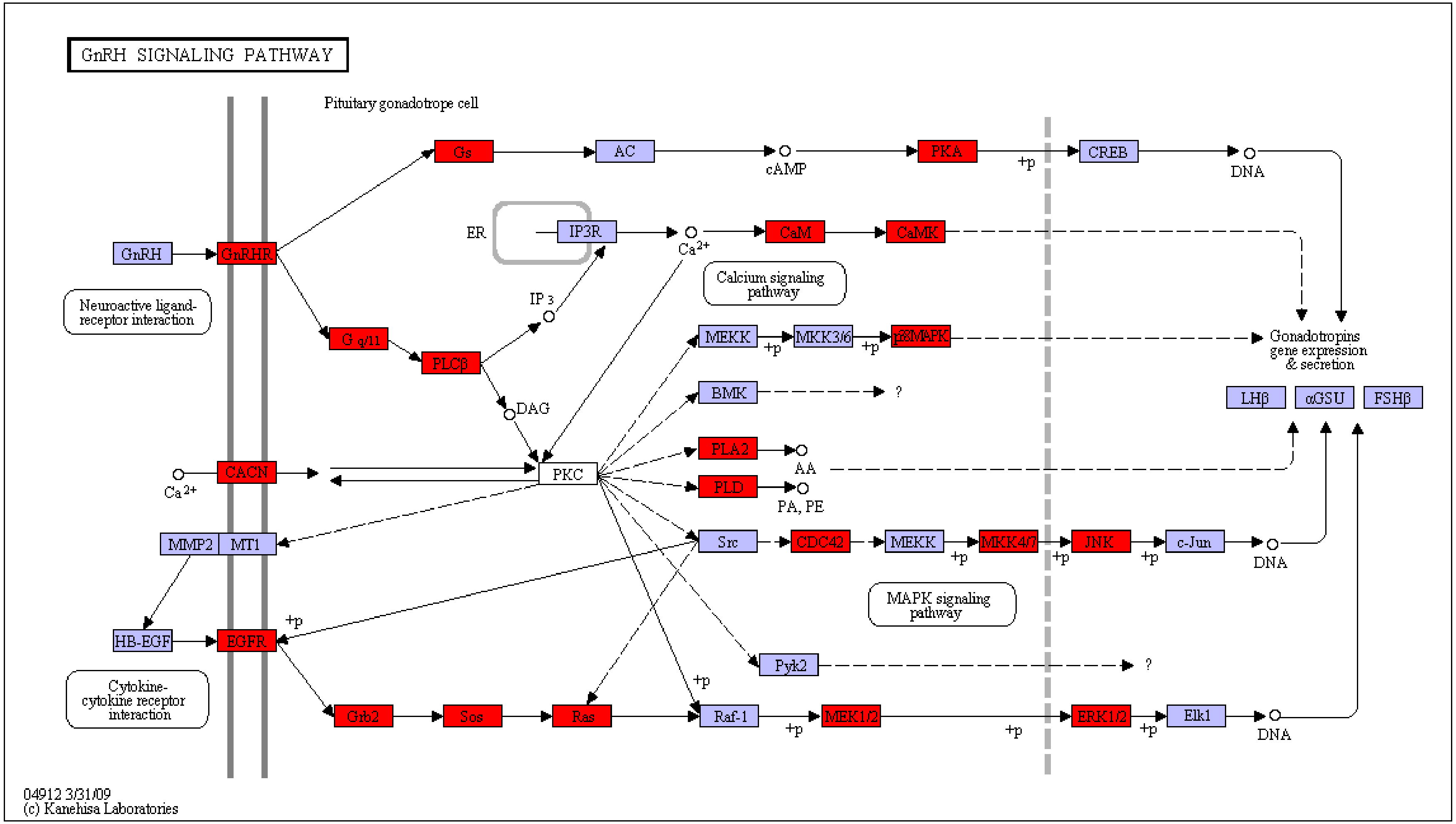

Figure 57: ko04912 GnRH signaling pathway

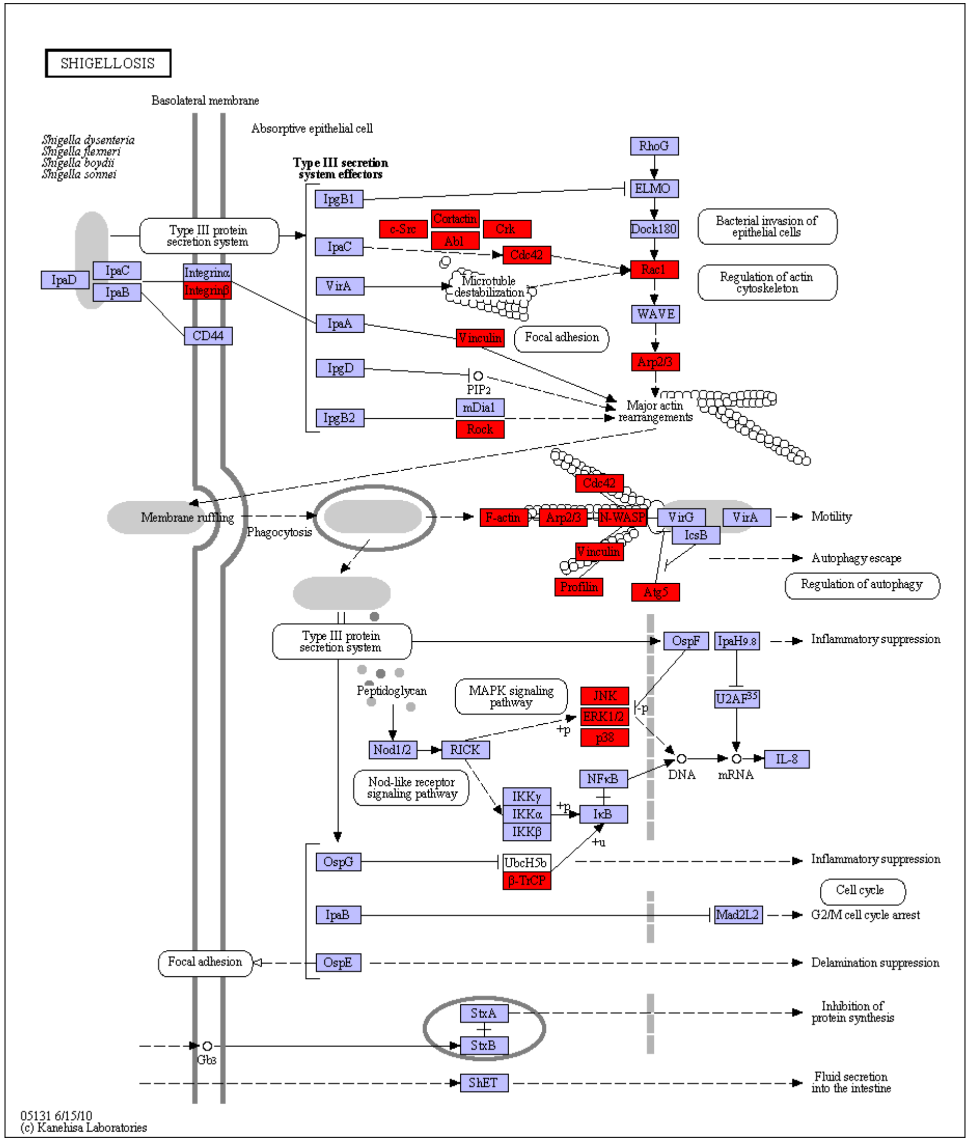

Figure 58: ko05131 Shigellosis

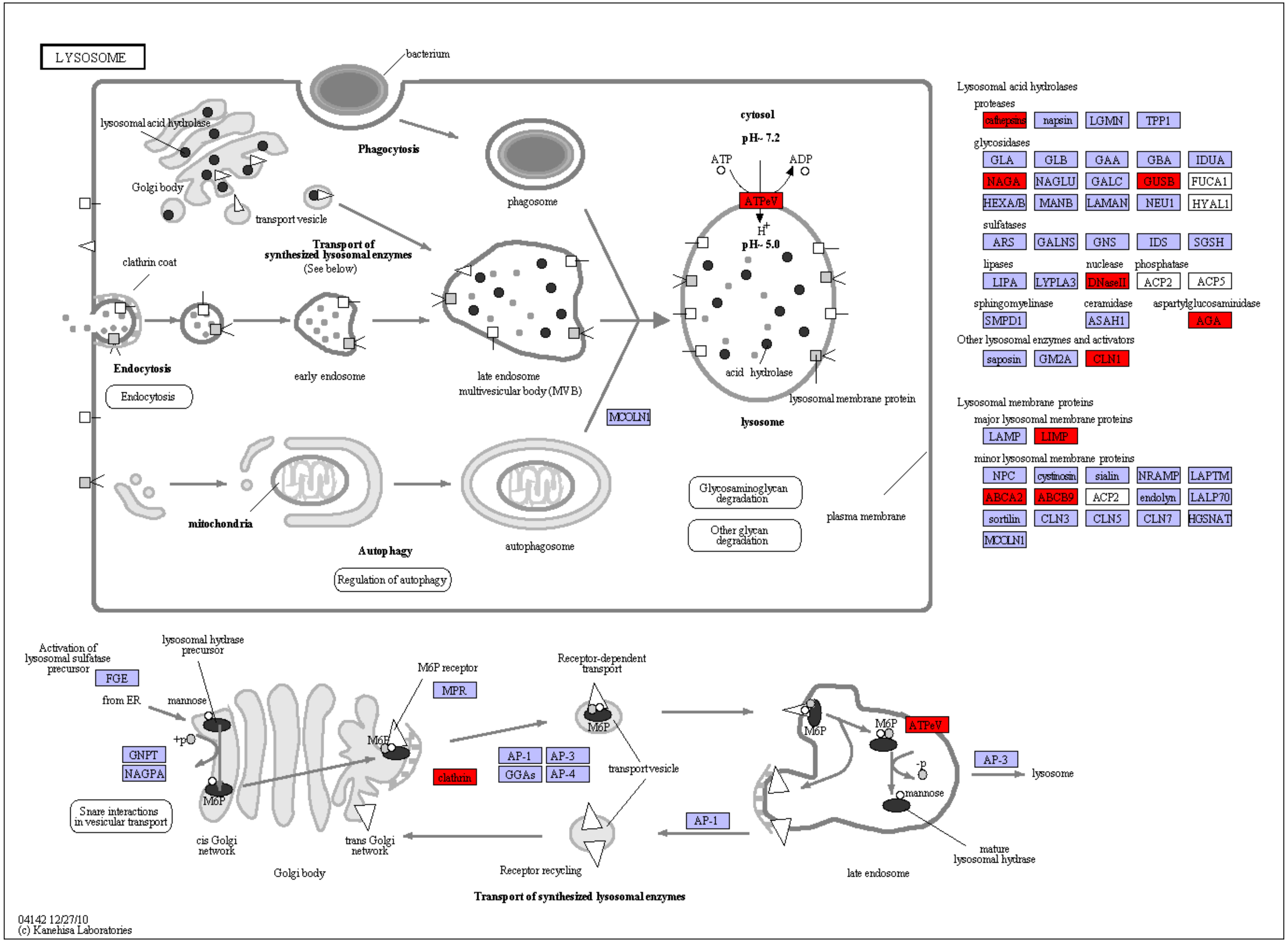

Figure 59: ko04142 Lysosome

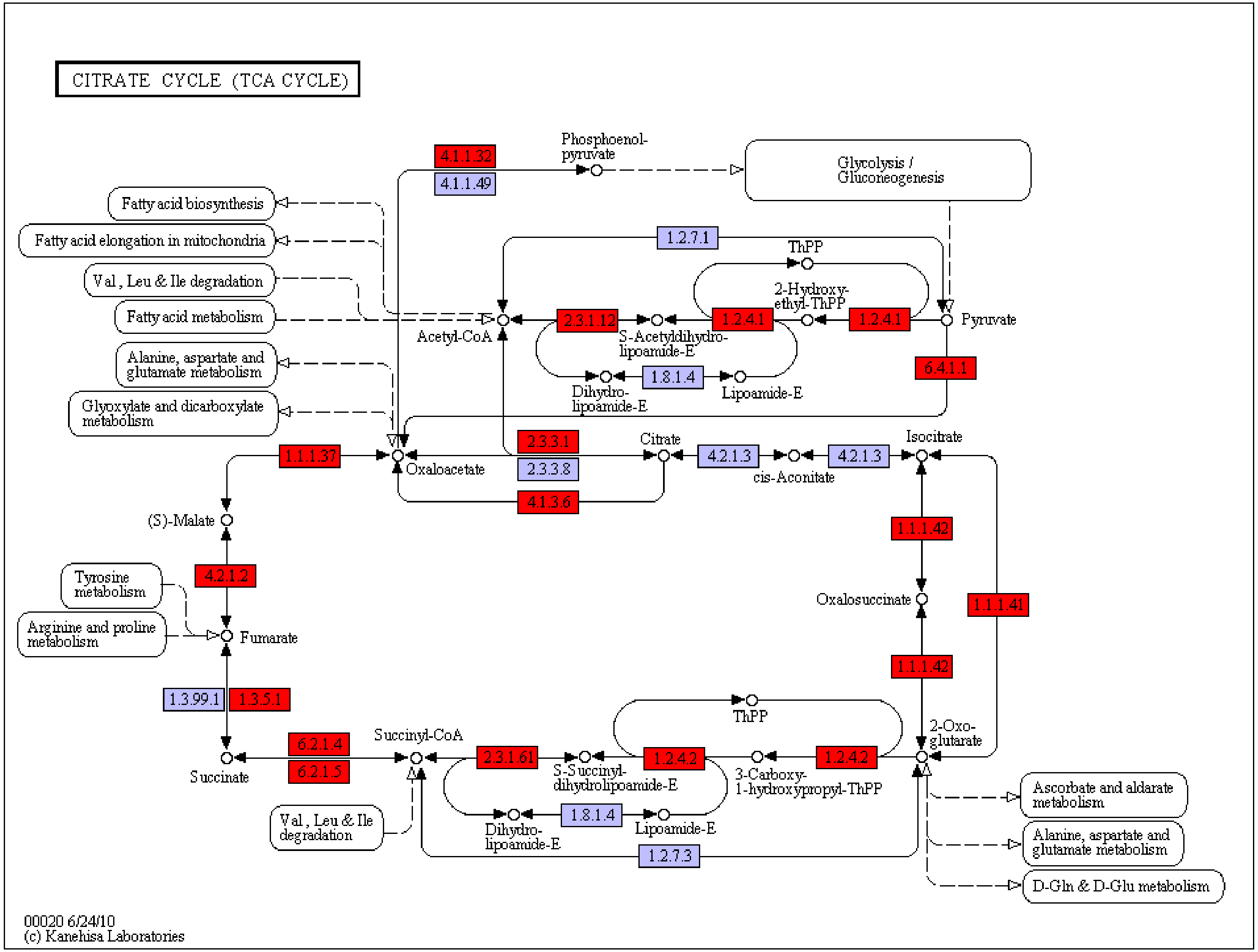

Figure 60: ko00020 Citrate cycle (TCA cycle)

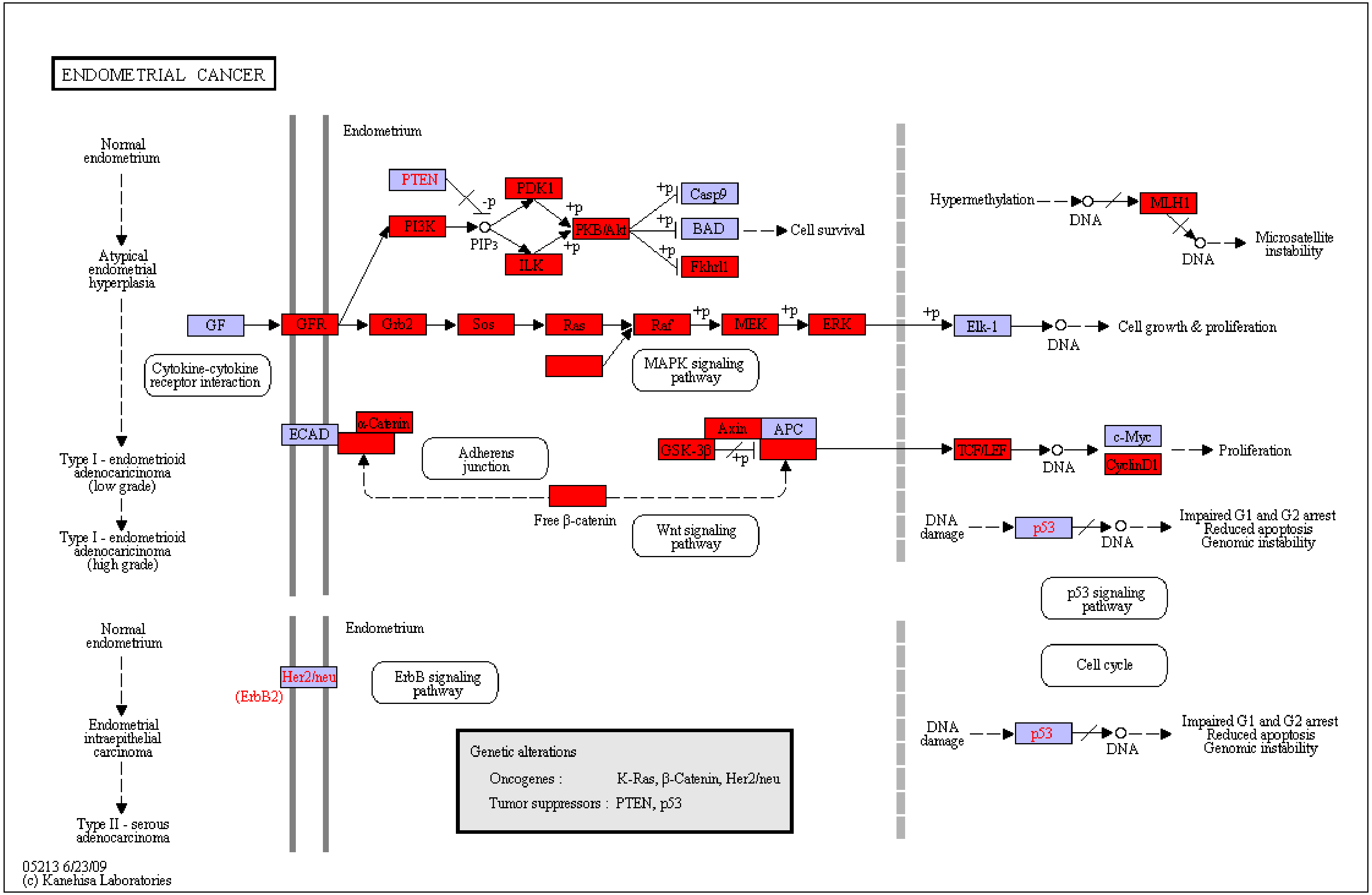

Figure 61: ko05213 Endometrial cancer

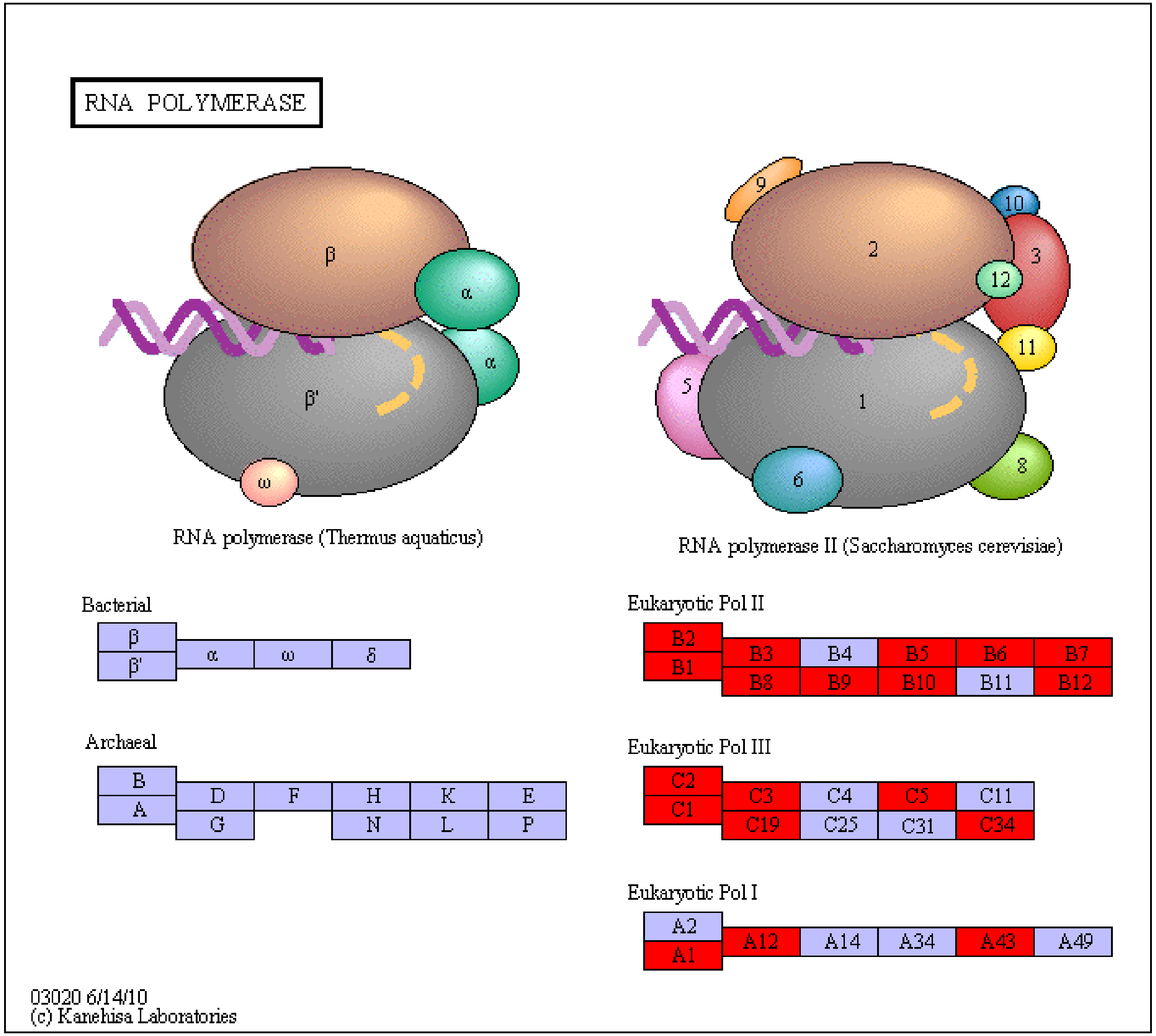

Figure 62: ko03020 RNA polymerase

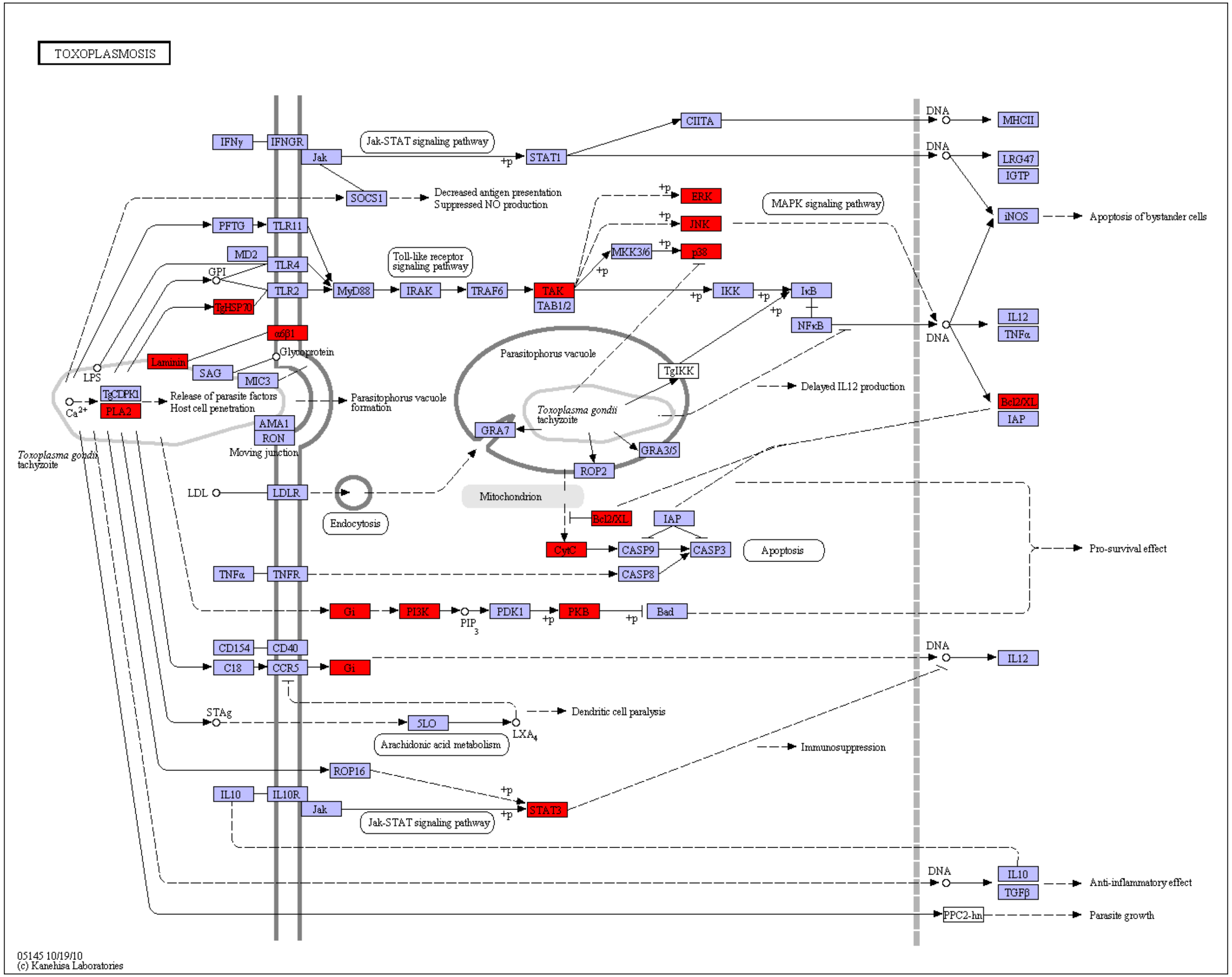

Figure 63: ko05145 Toxoplasmosis

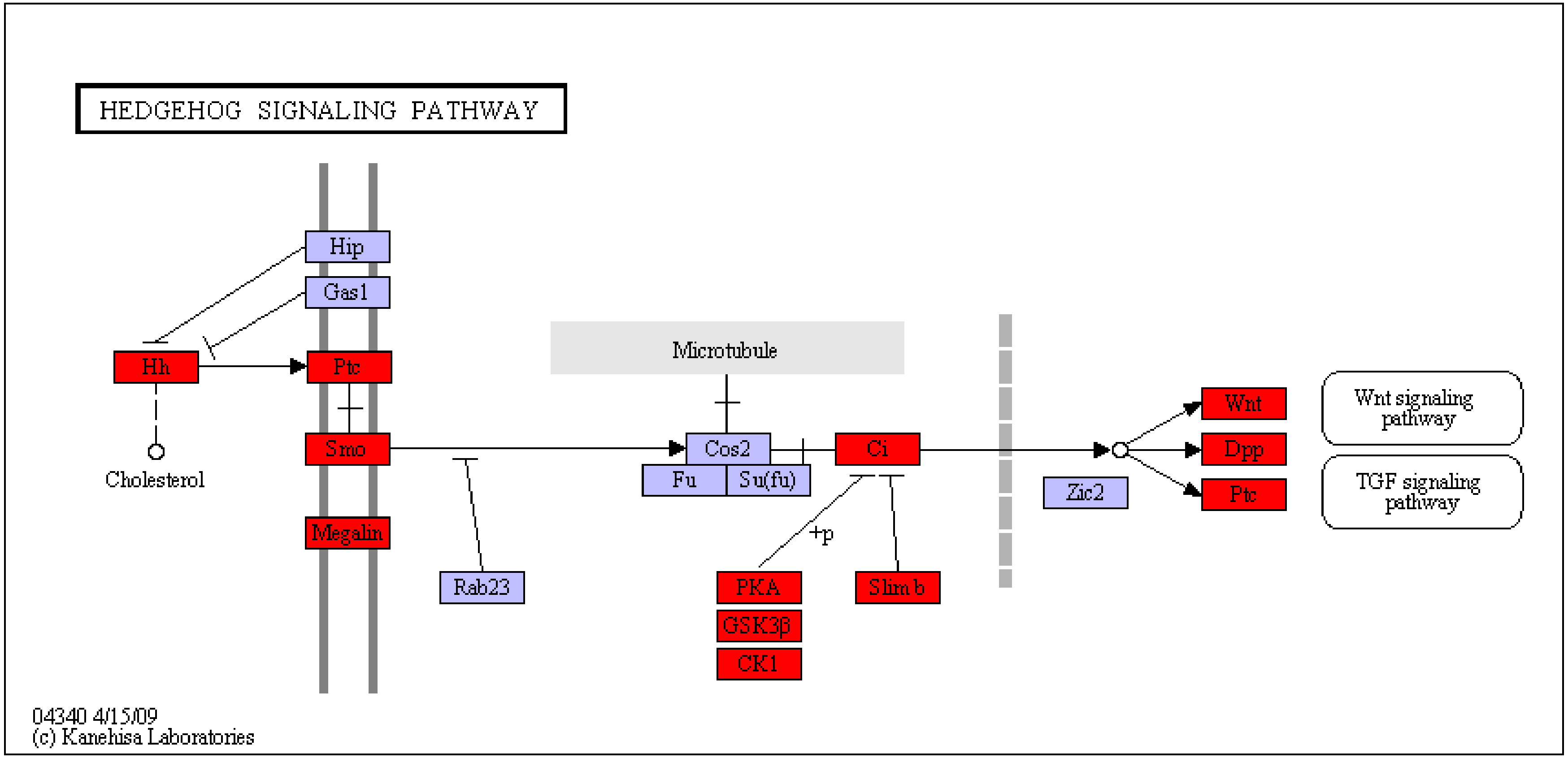

Figure 64: ko04340 Hedgehog signaling pathway

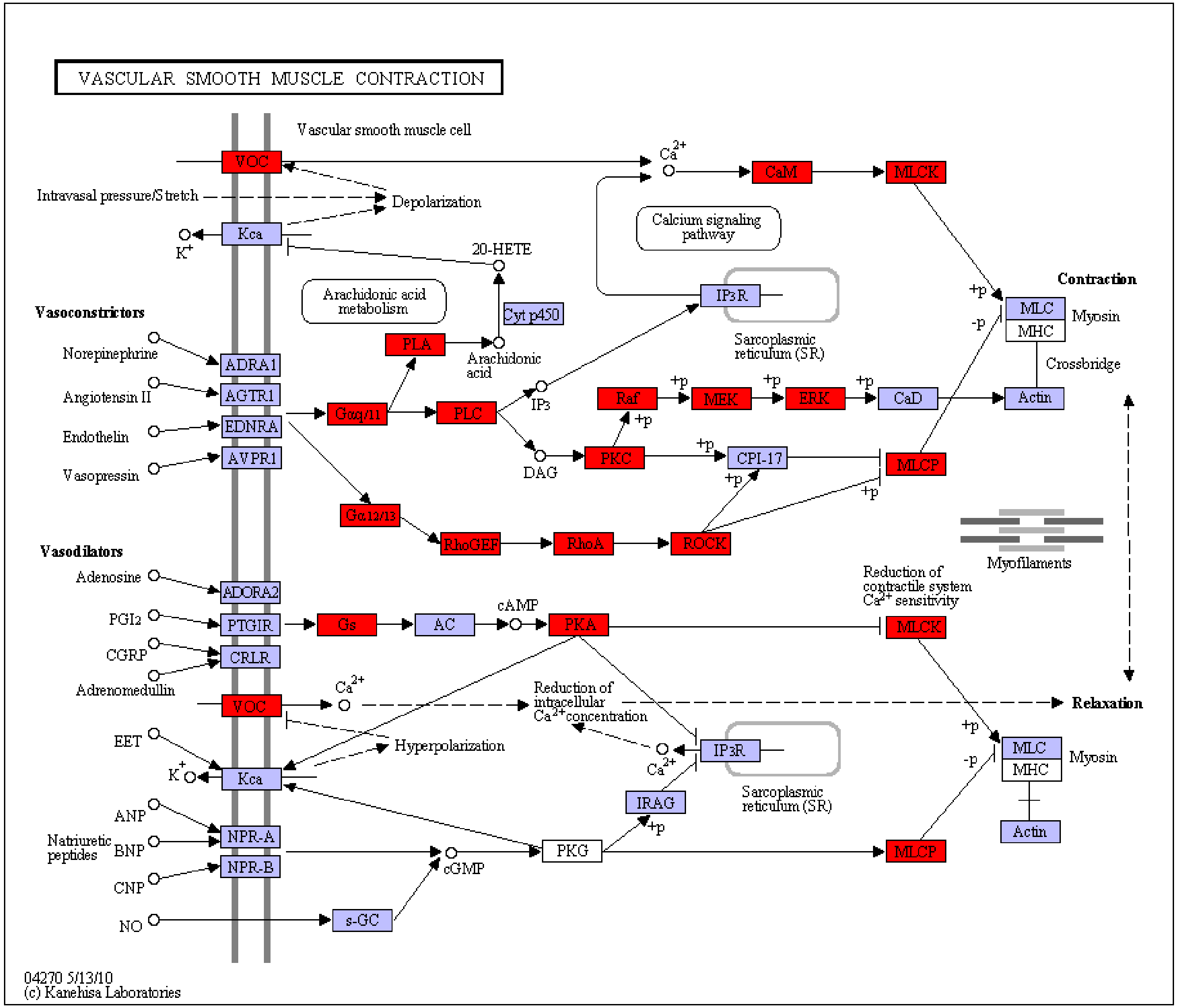

Figure 65: ko04270 Vascular smooth muscle contraction

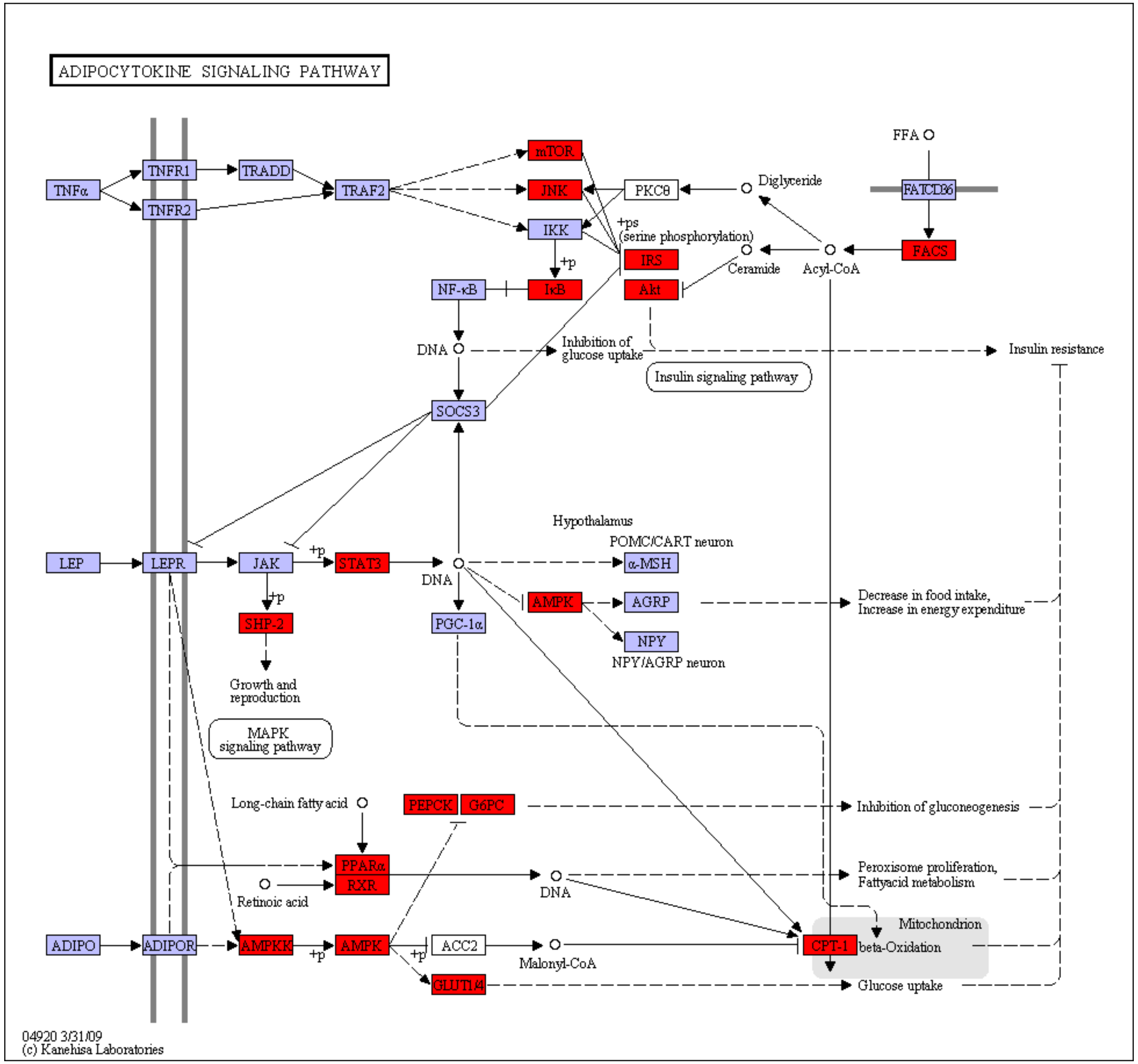

Figure 66: ko04920 Adipocytokine signaling pathway

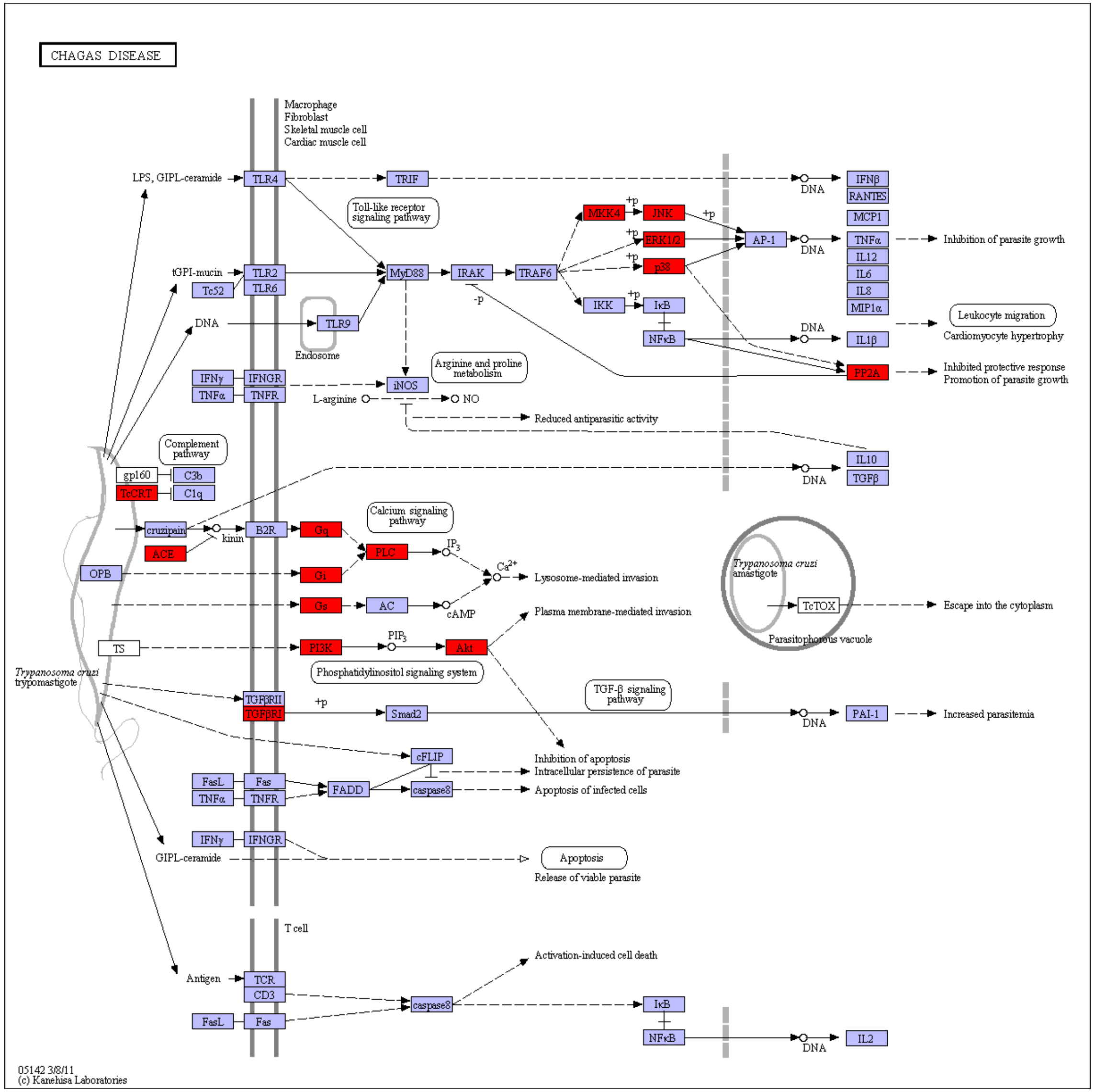

Figure 67: ko05142 Chagas disease (American trypanosomiasis)

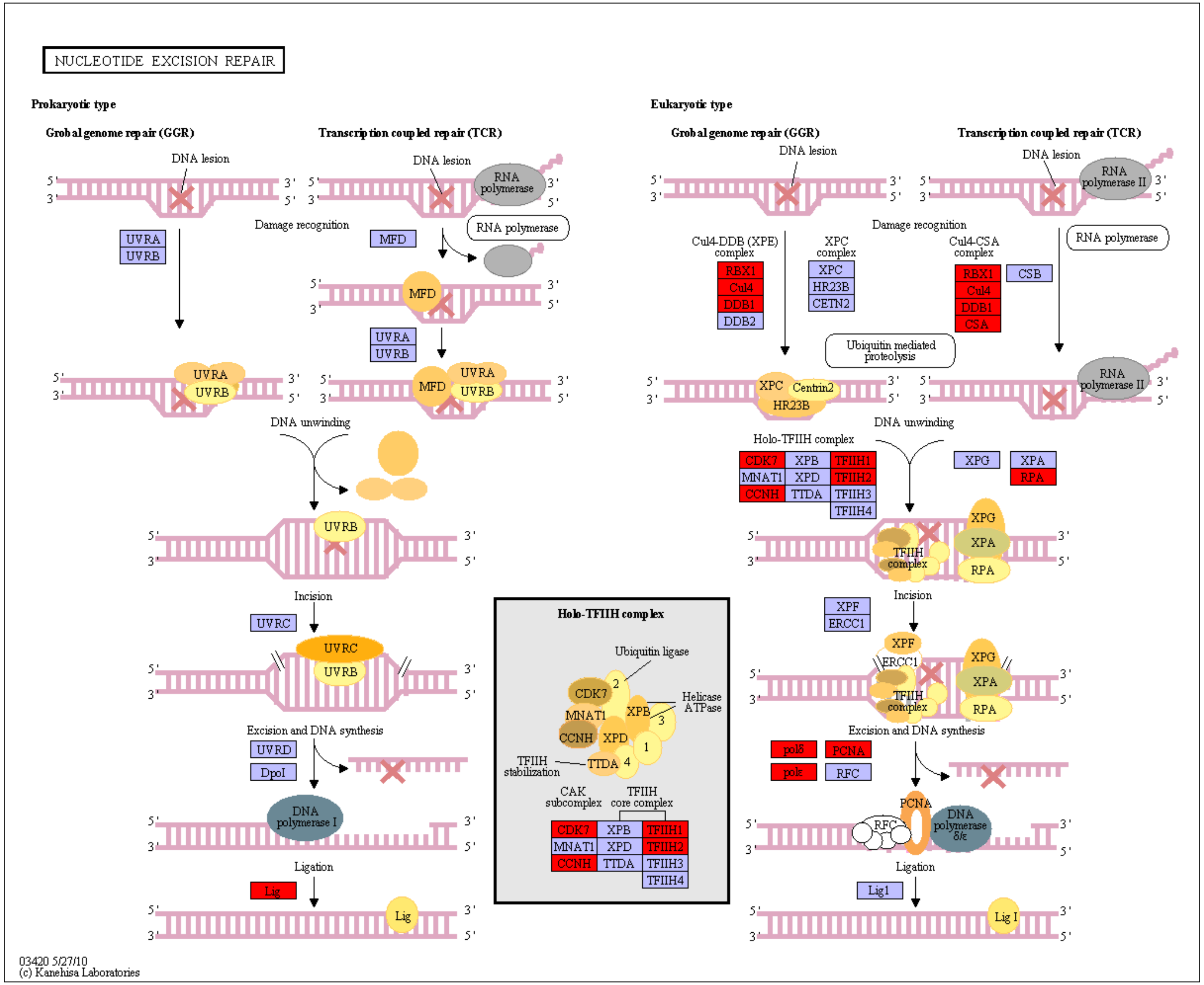

Figure 68: ko03420 Nucleotide excision repair

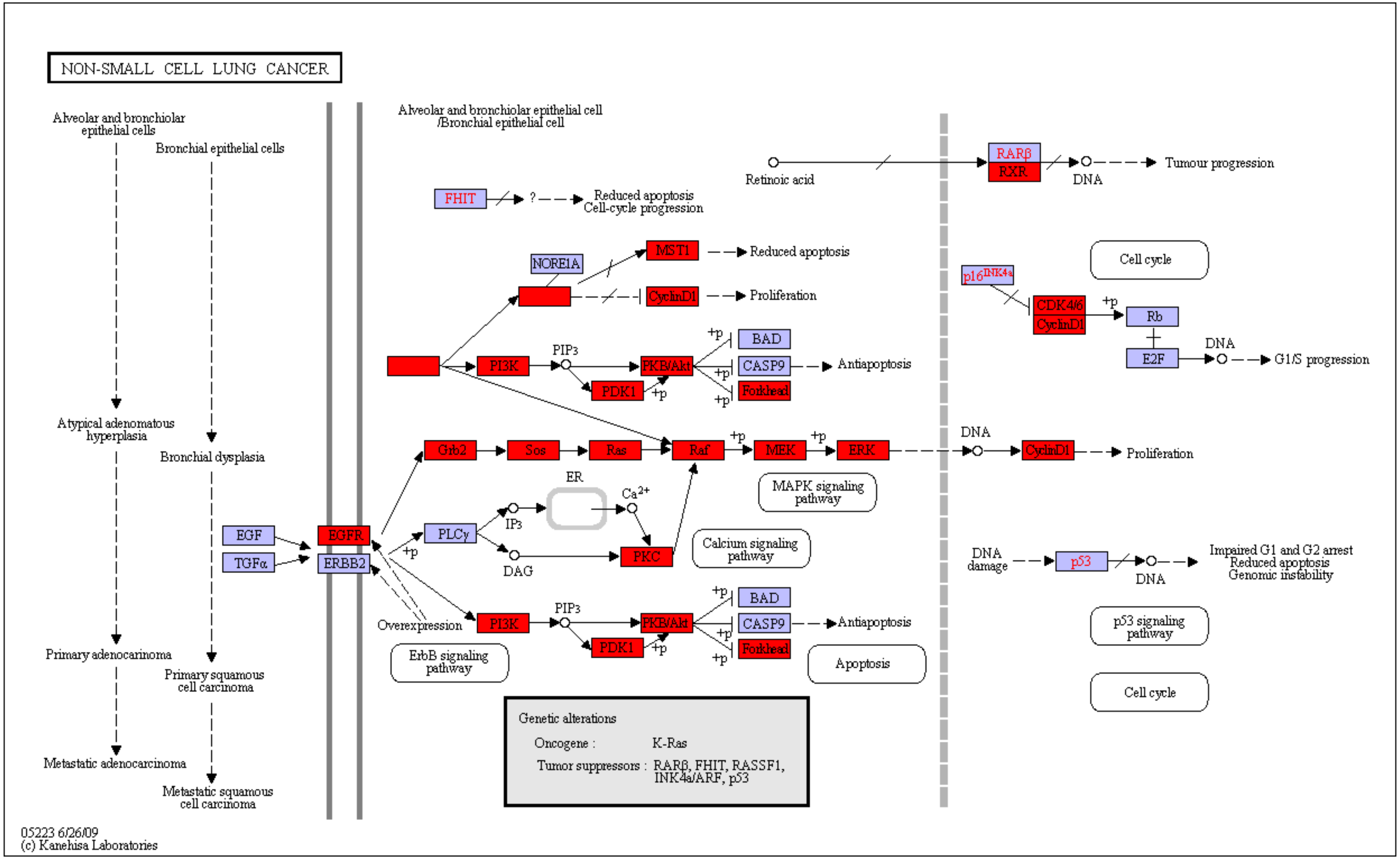

Figure 69: ko05223 Non-small cell lung cancer

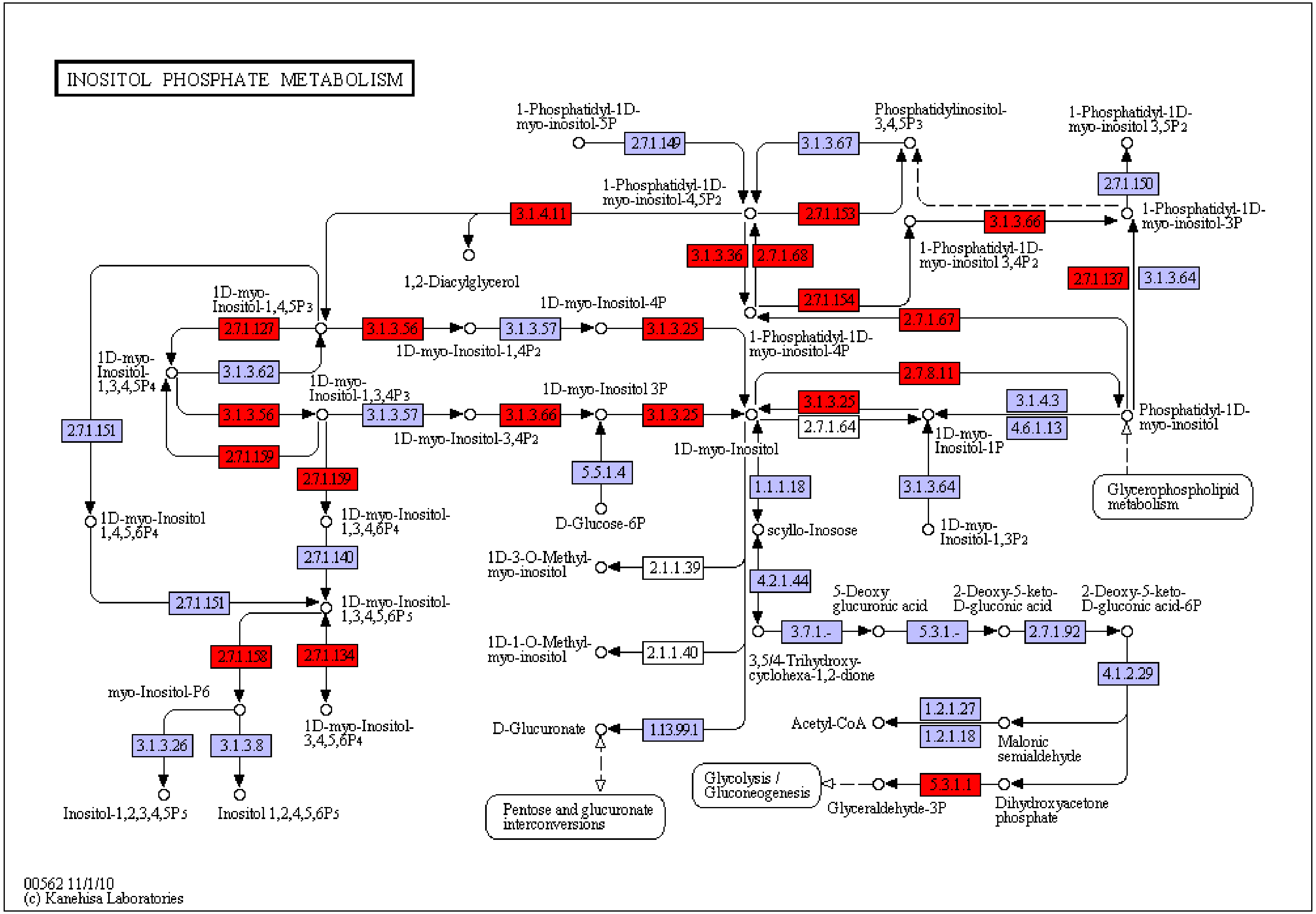

Figure 70: ko00562 Inositol phosphate metabolism

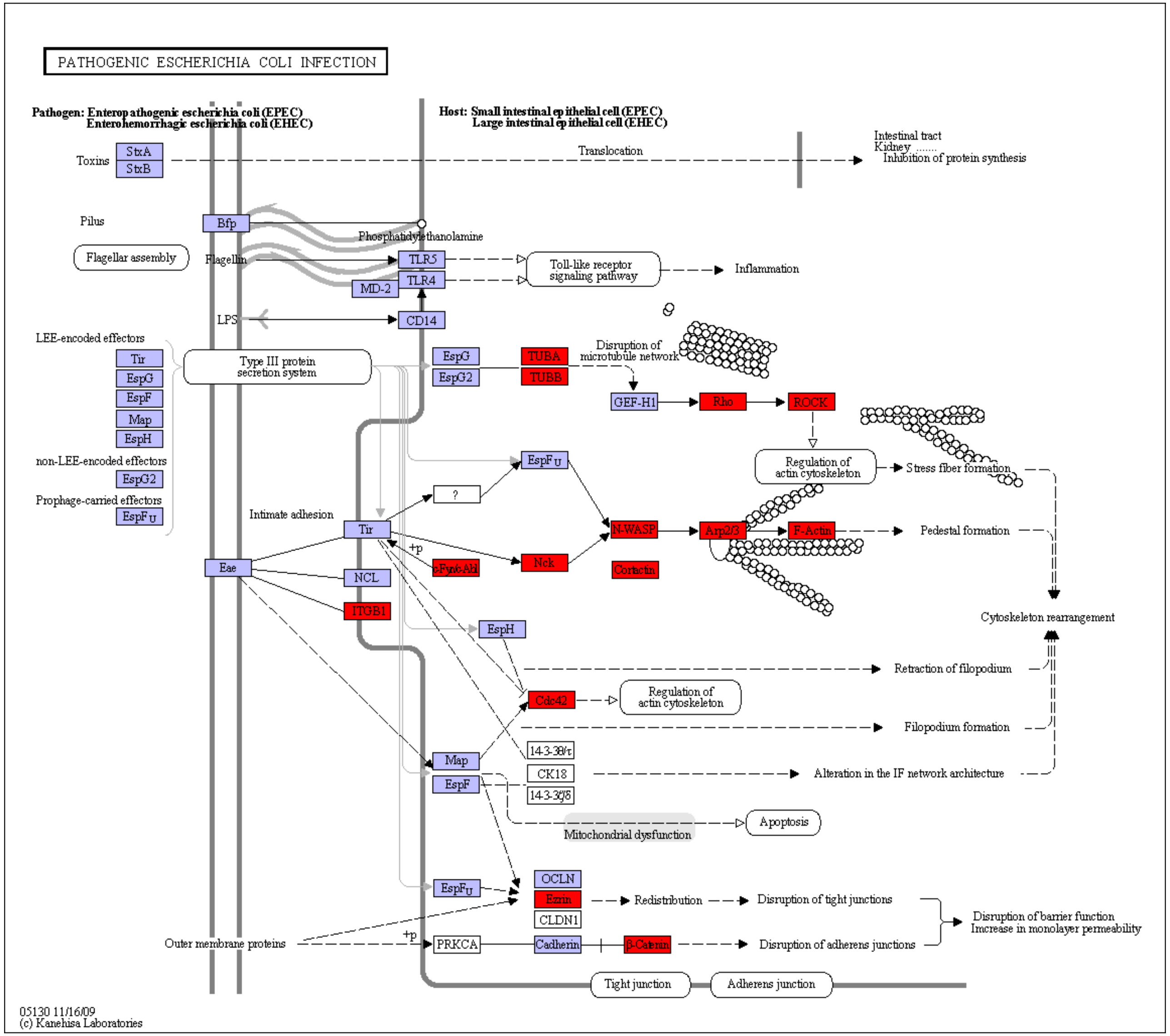

Figure 71: ko05130 Pathogenic Escherichia coli infection

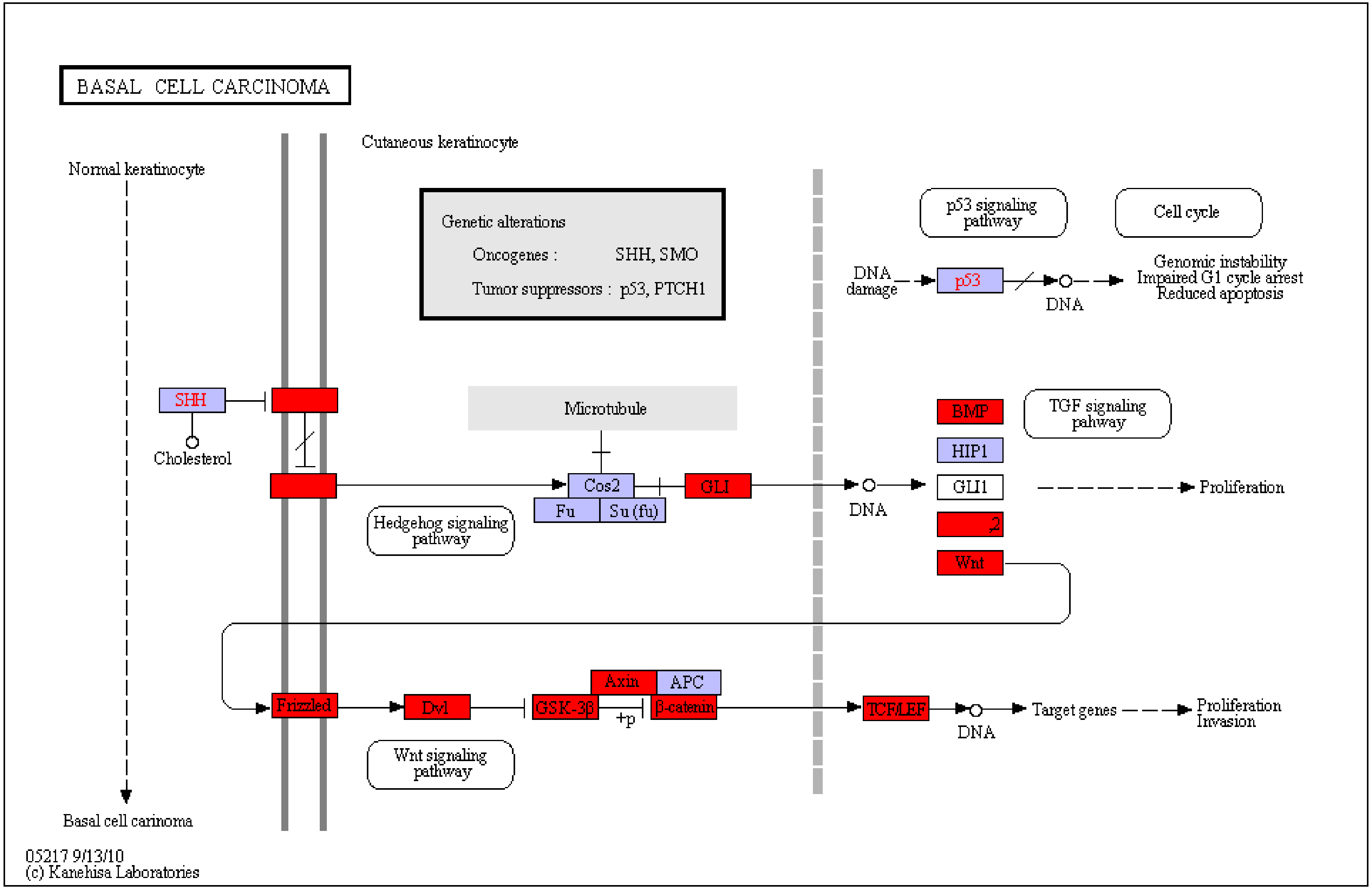

Figure 72: ko05217 Basal cell carcinoma

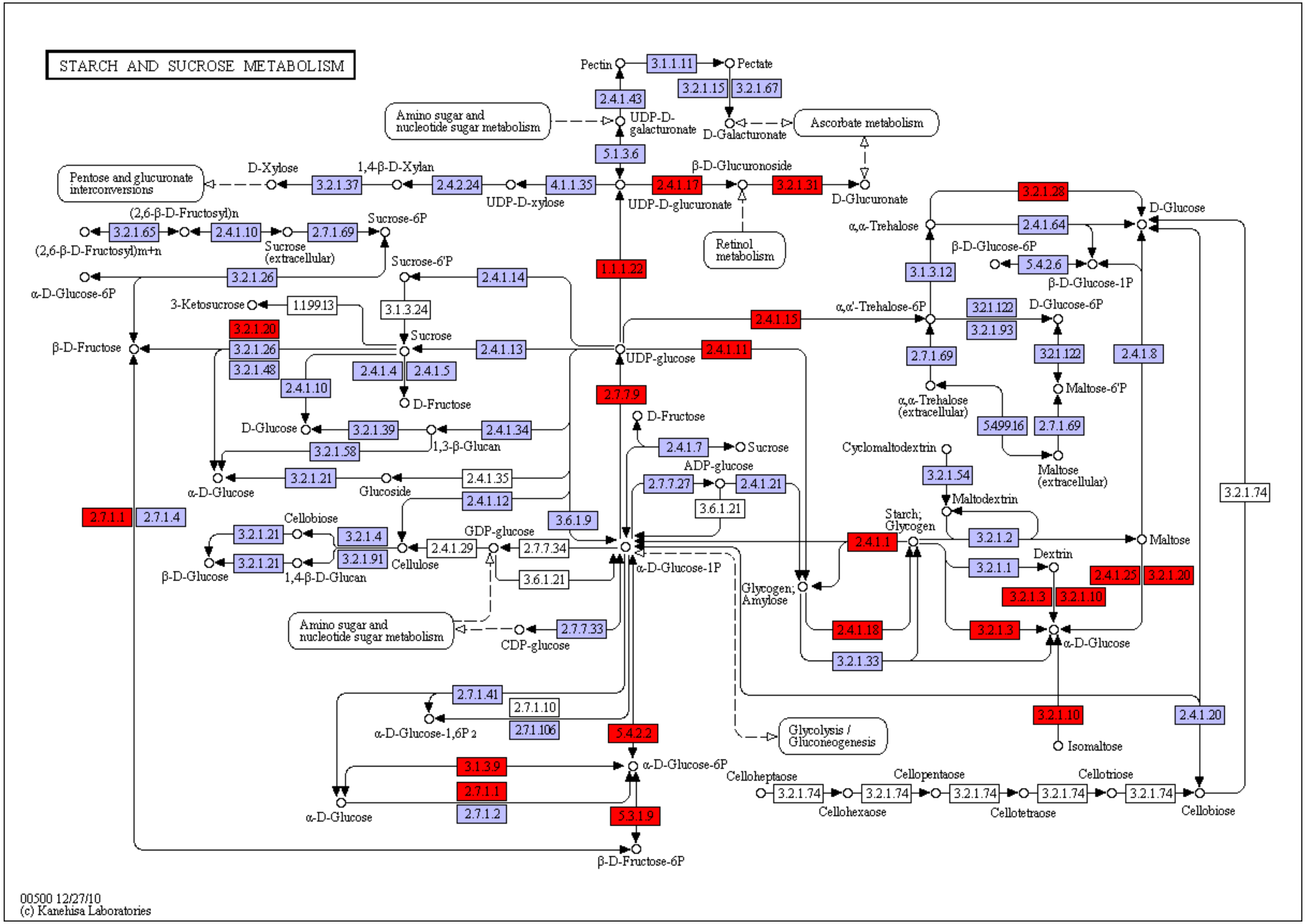

Figure 73: ko00500 Starch and sucrose metabolism

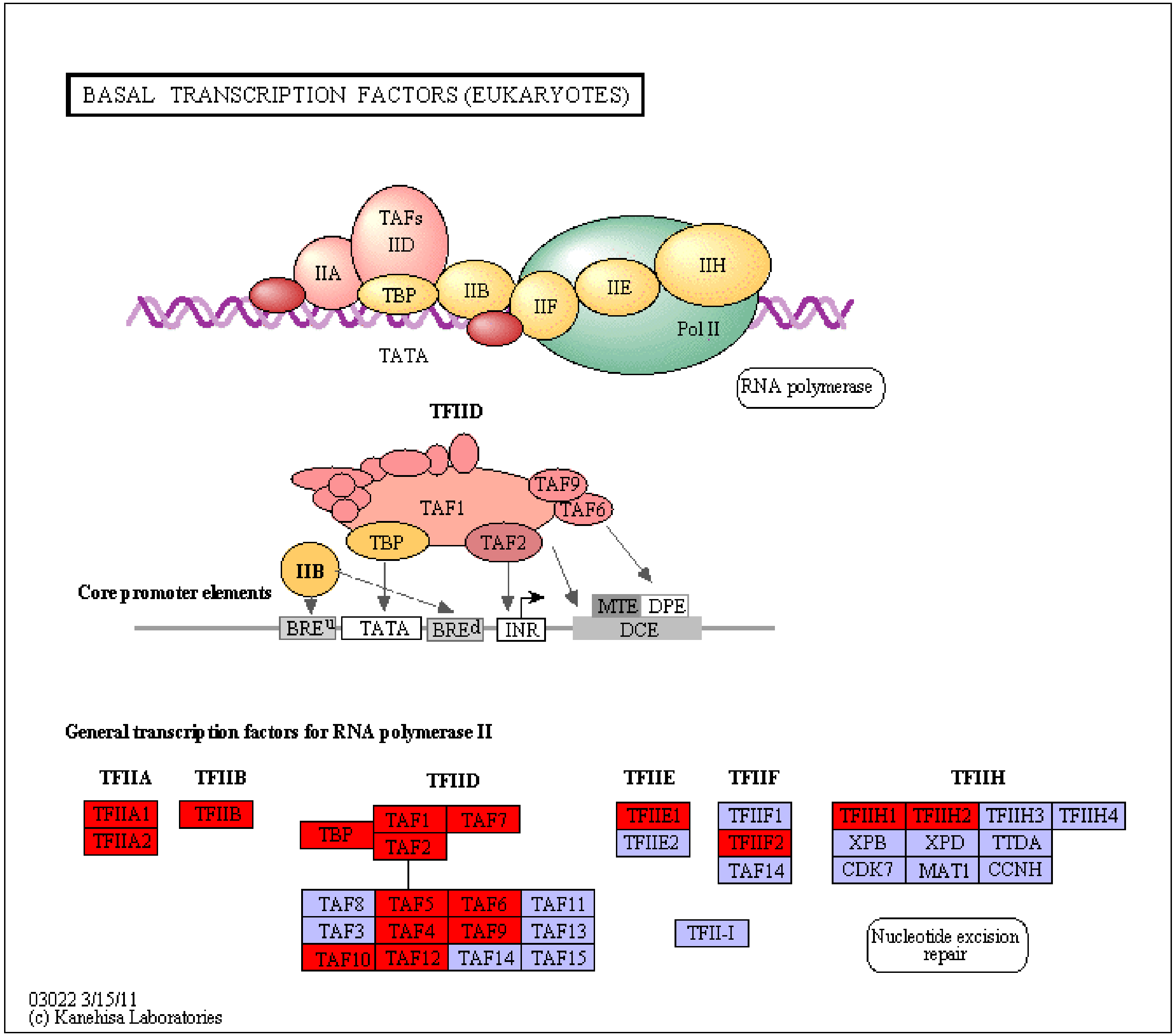

Figure 74: ko03022 Basal transcription factors

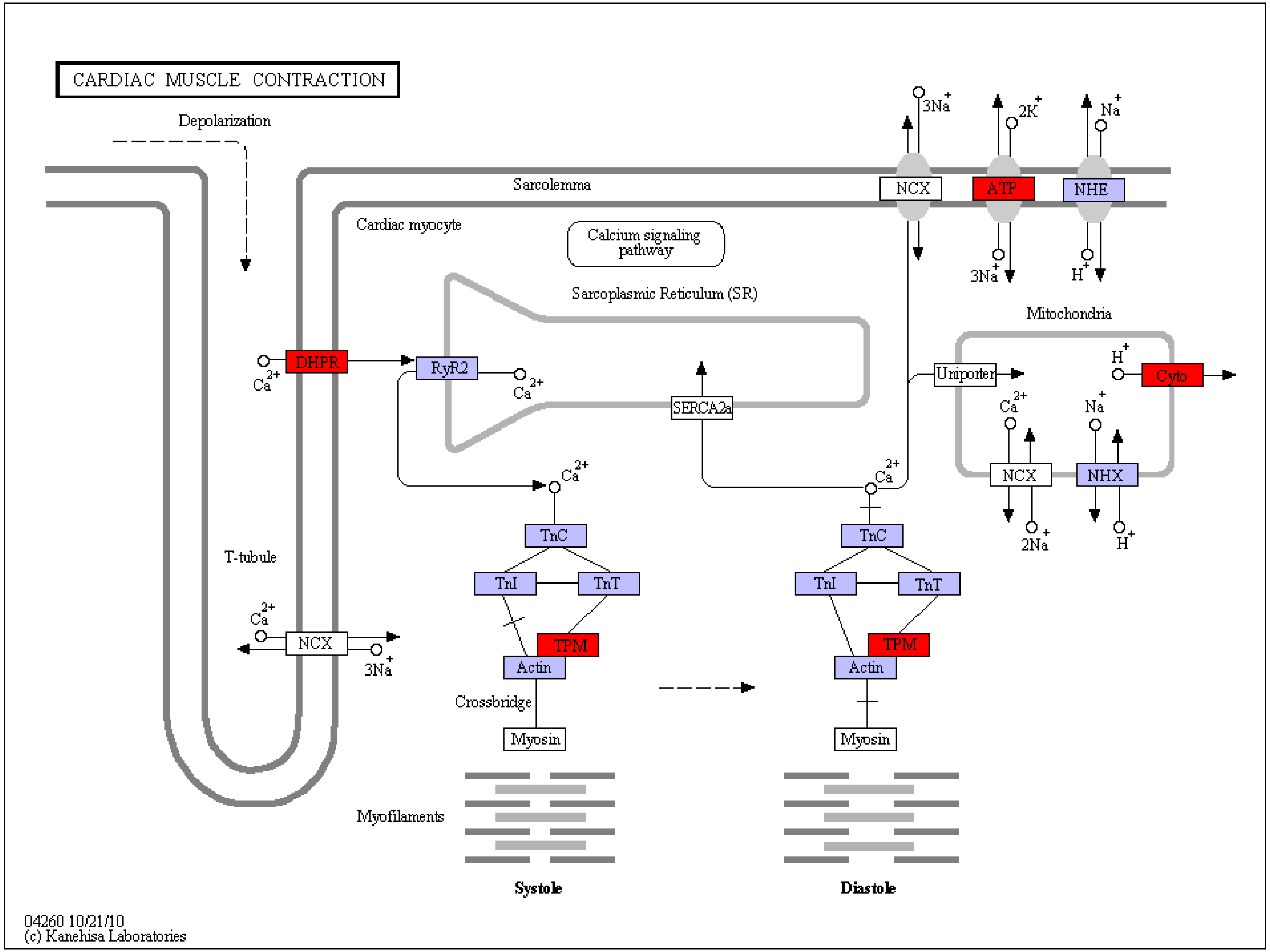

Figure 75: ko04260 Cardiac muscle contraction

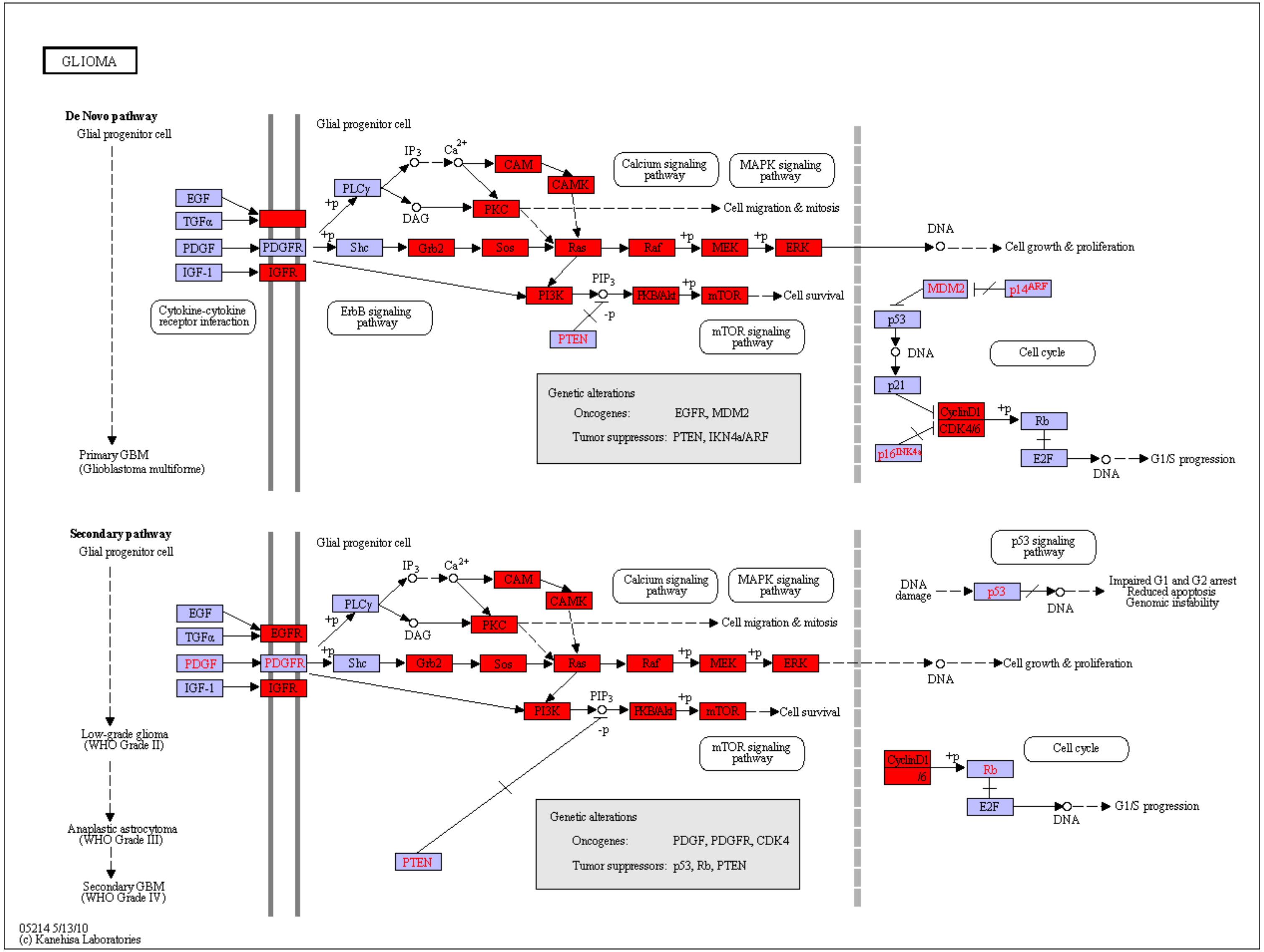

Figure 76: ko05214 Glioma

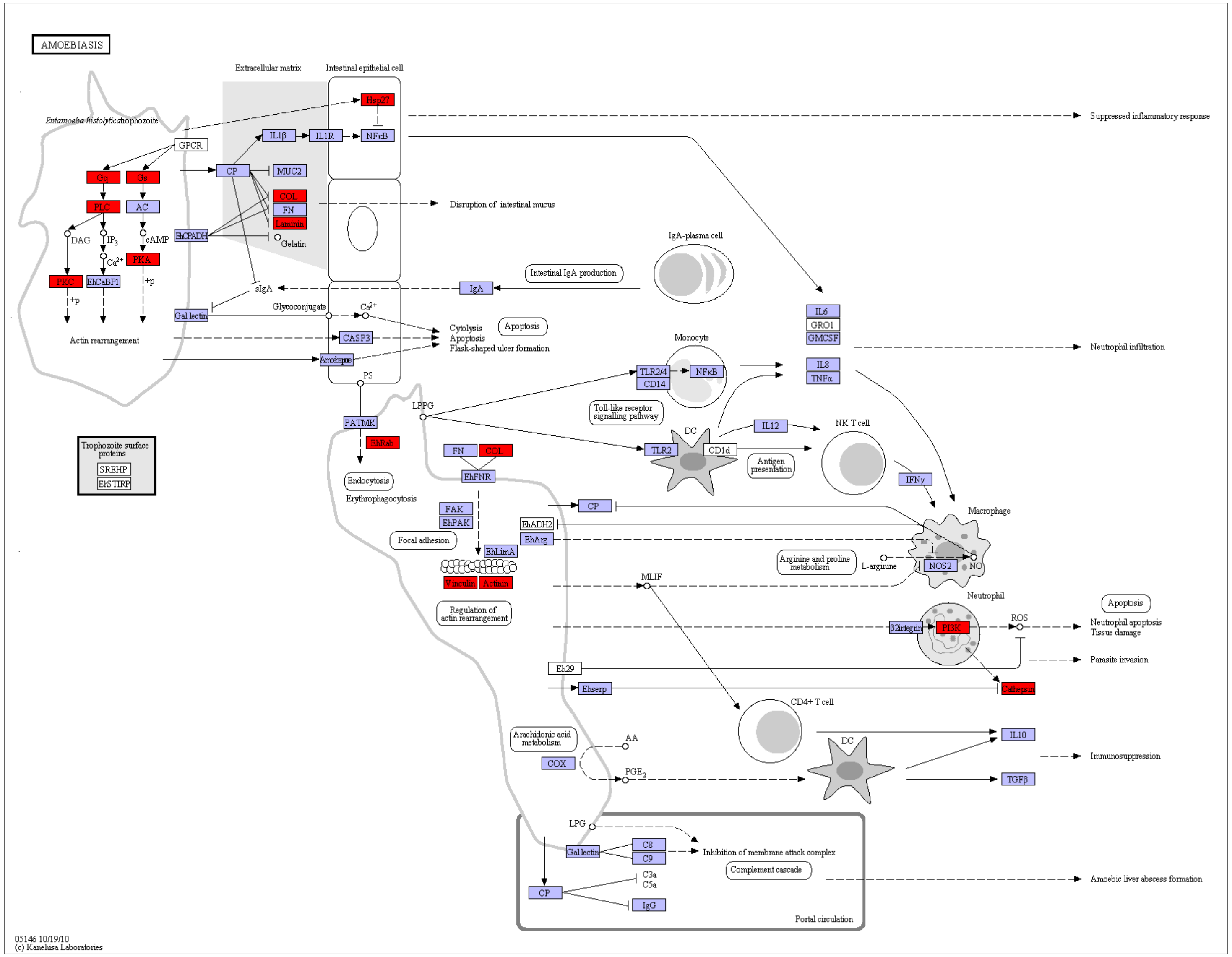

Figure 77: ko05146 Amoebiasis

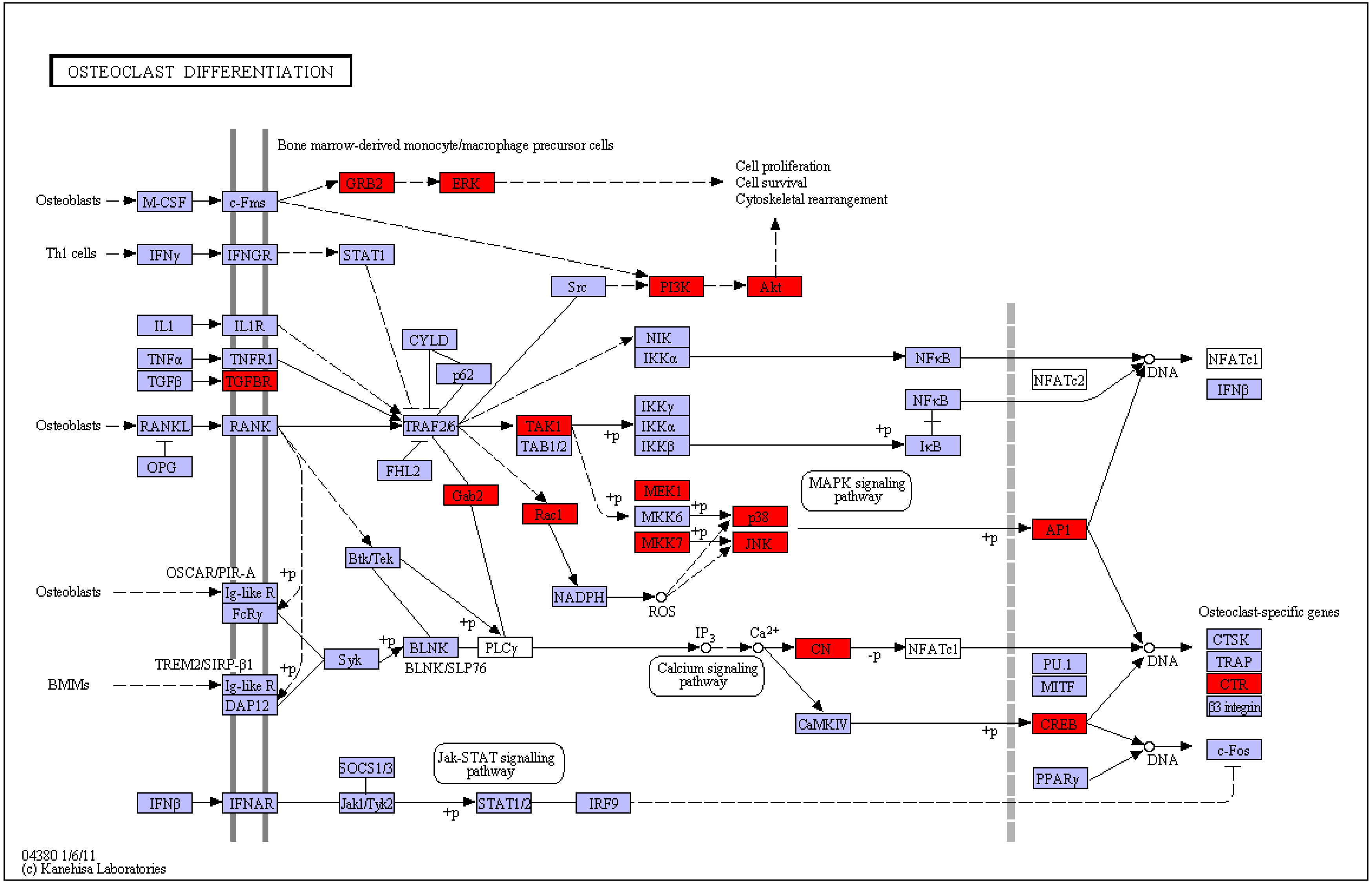

Figure 78: ko04380 Osteoclast differentiation

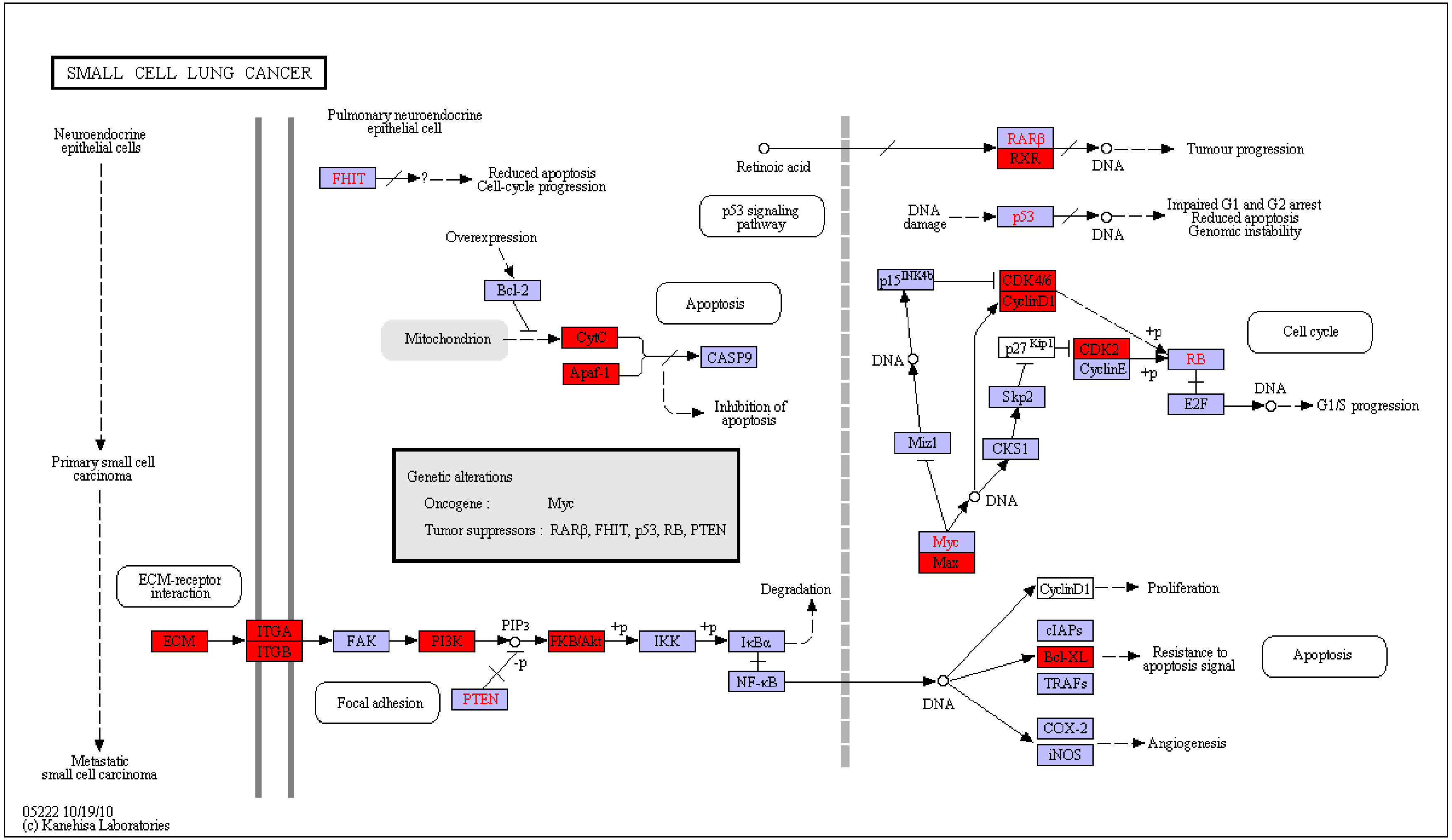

Figure 79: ko05222 Small cell lung cancer

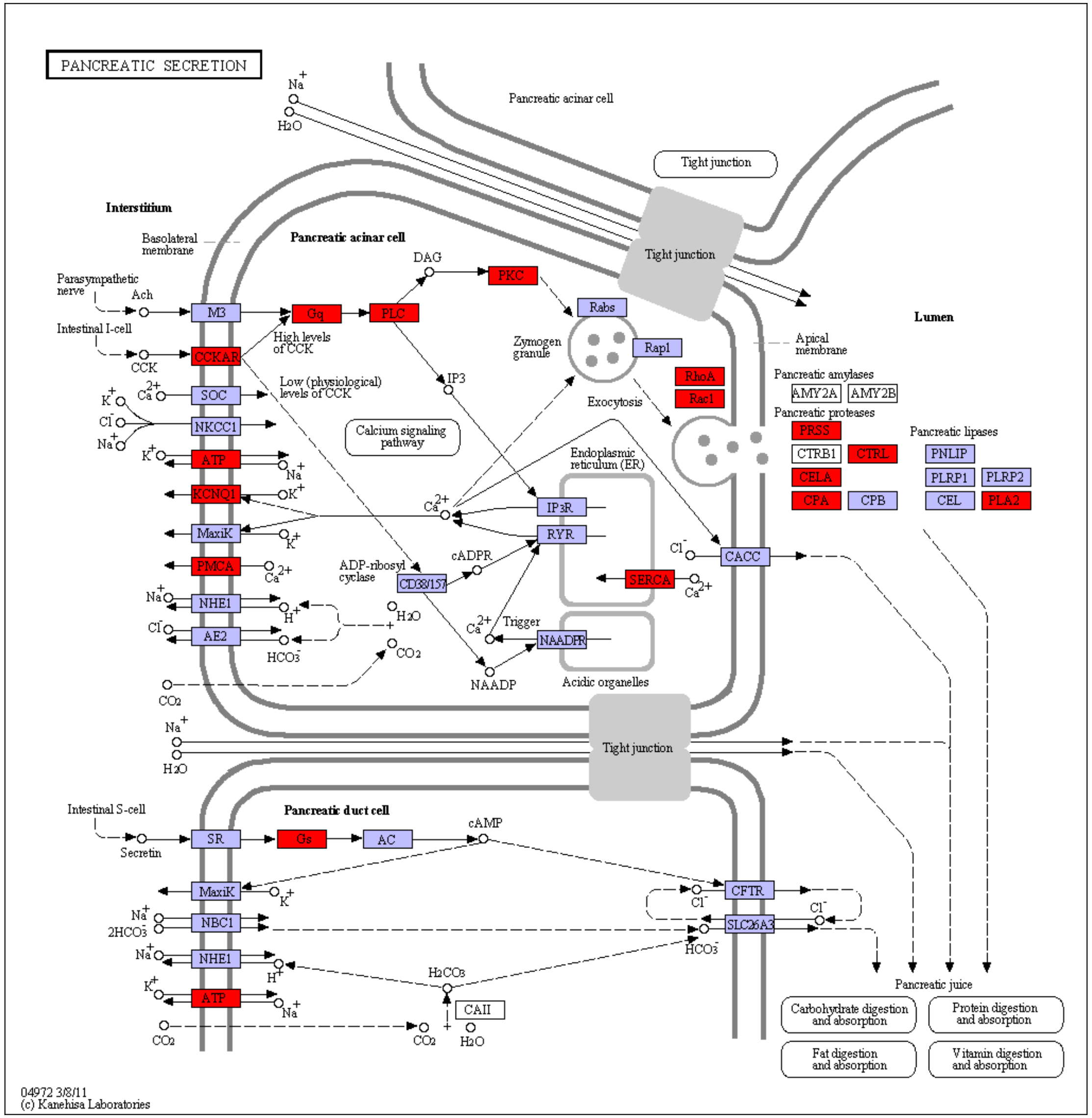

Figure 80: ko04972 Pancreatic secretion

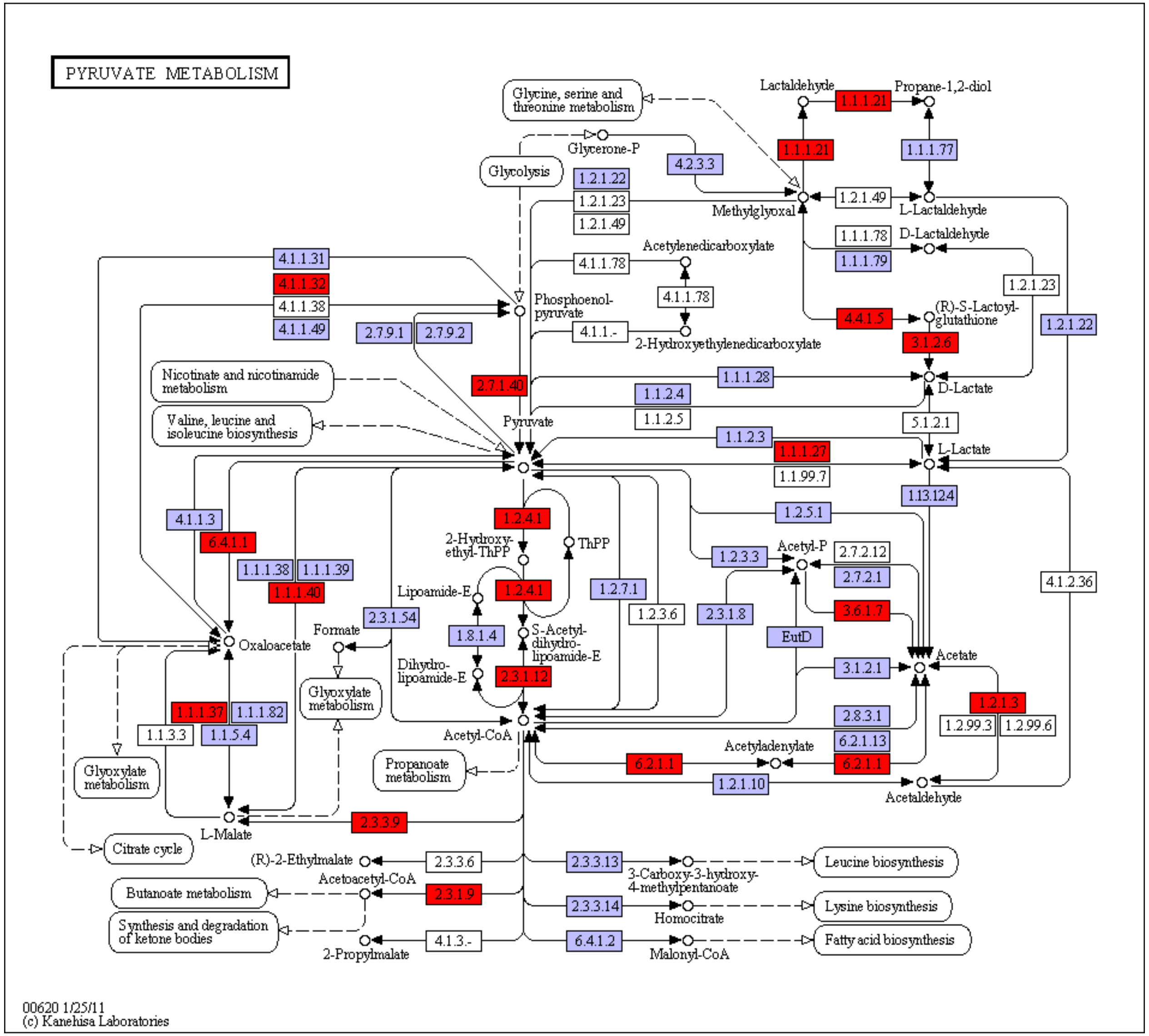

Figure 81: ko00620 Pyruvate metabolism

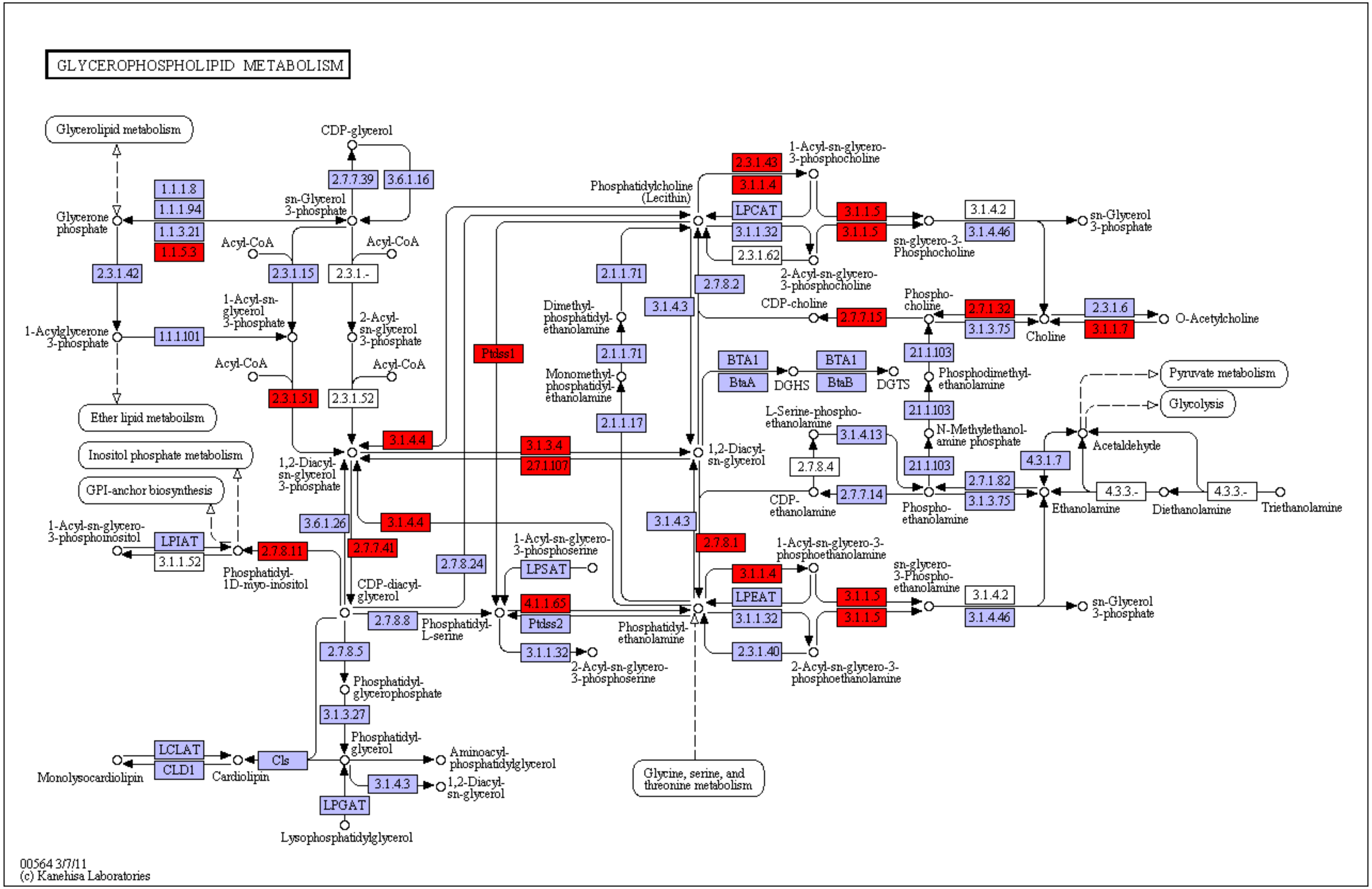

Figure 82: ko00564 Glycerophospholipid metabolism

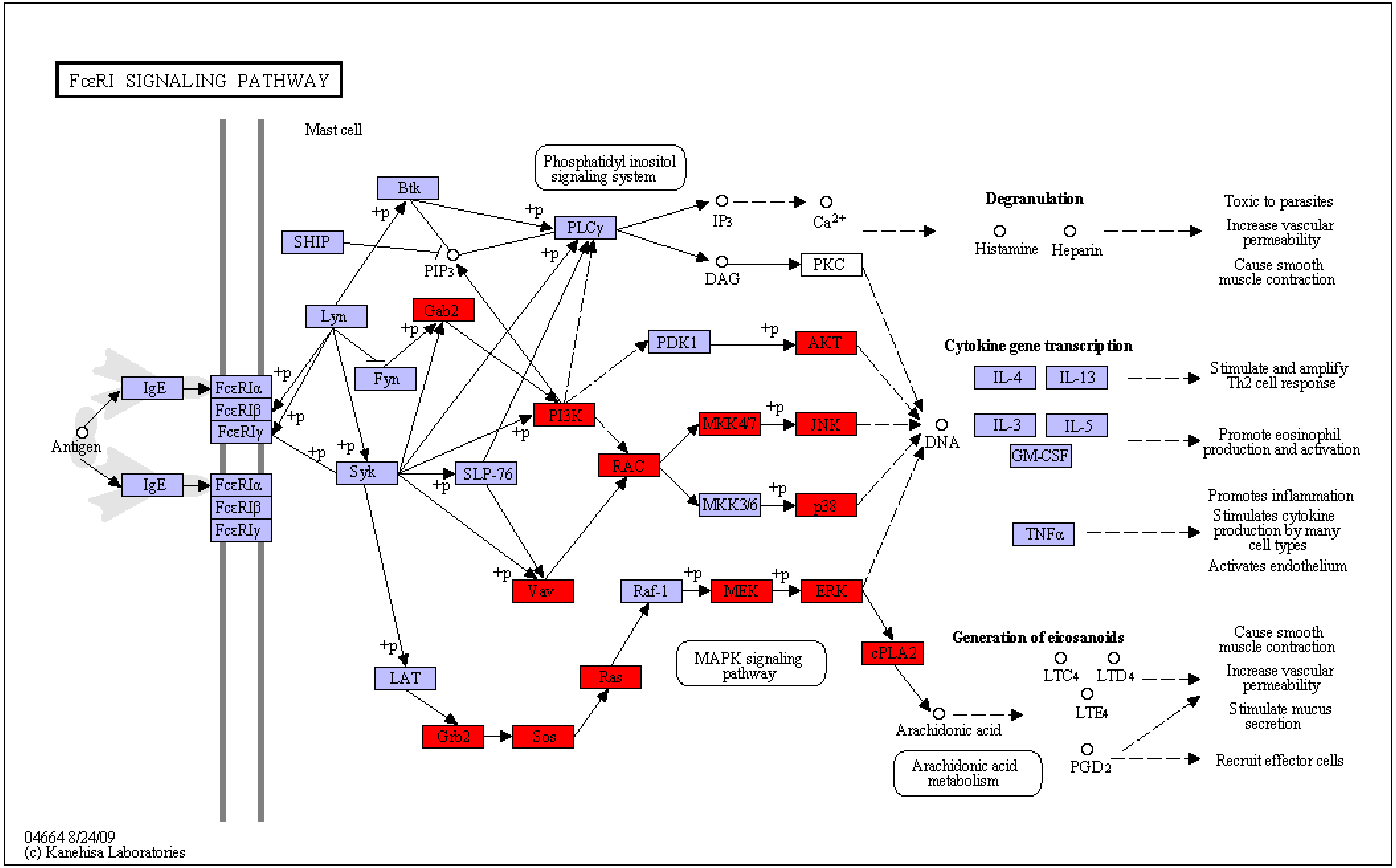

Figure 83: ko04664 Fc epsilon RI signaling pathway

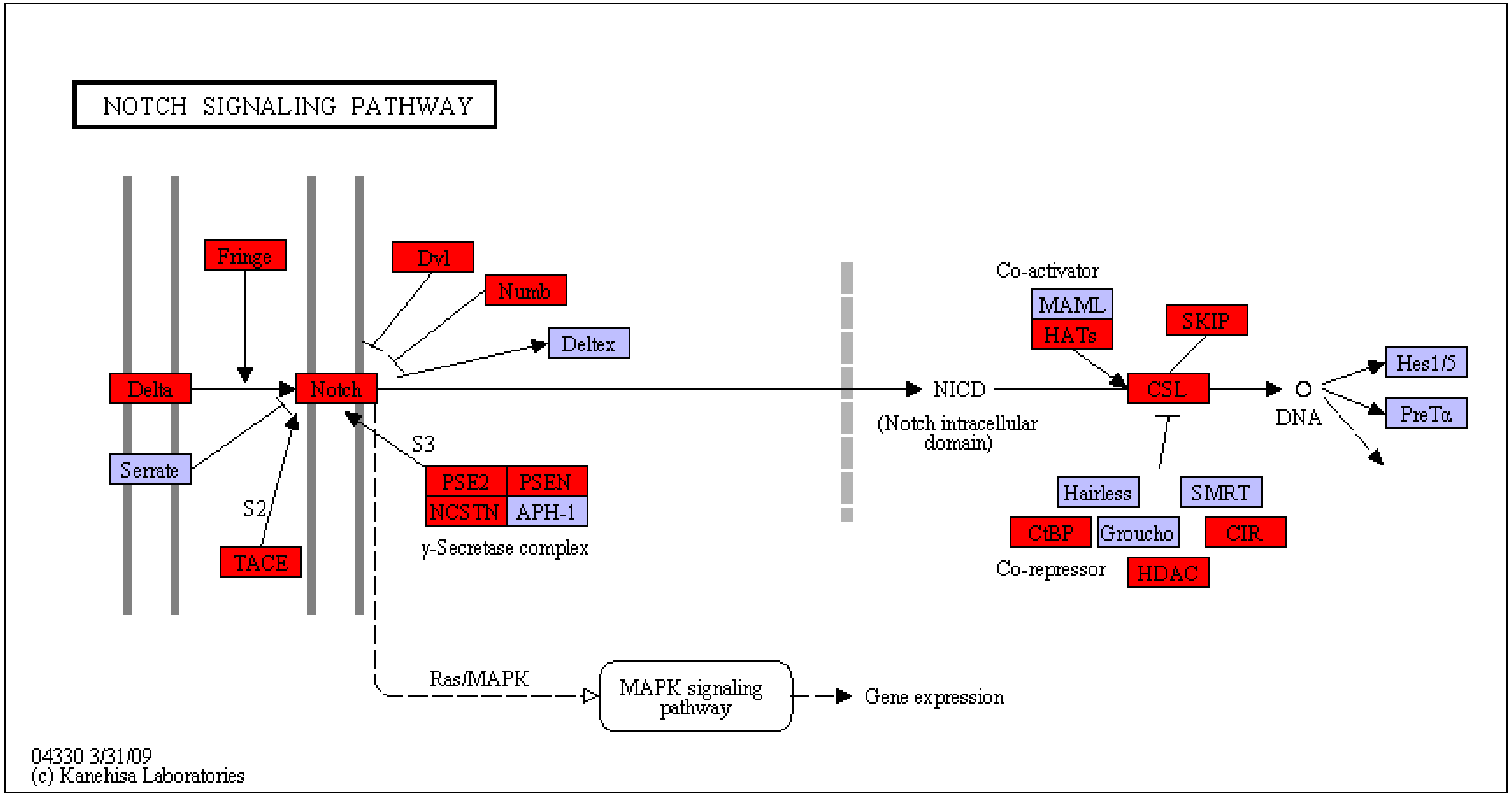

Figure 84: ko04330 Notch signaling pathway

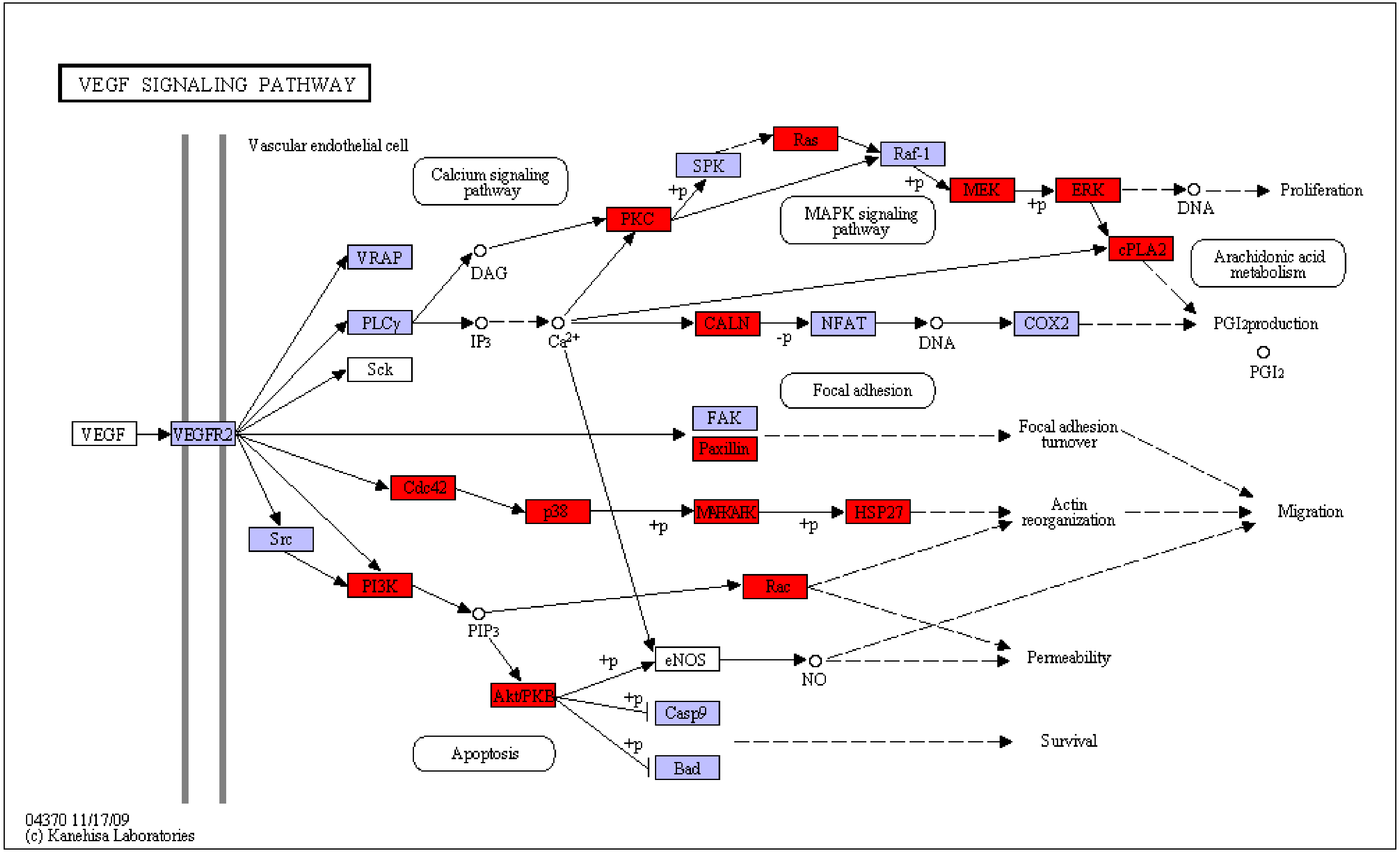

Figure 85: ko04370 VEGF signaling pathway

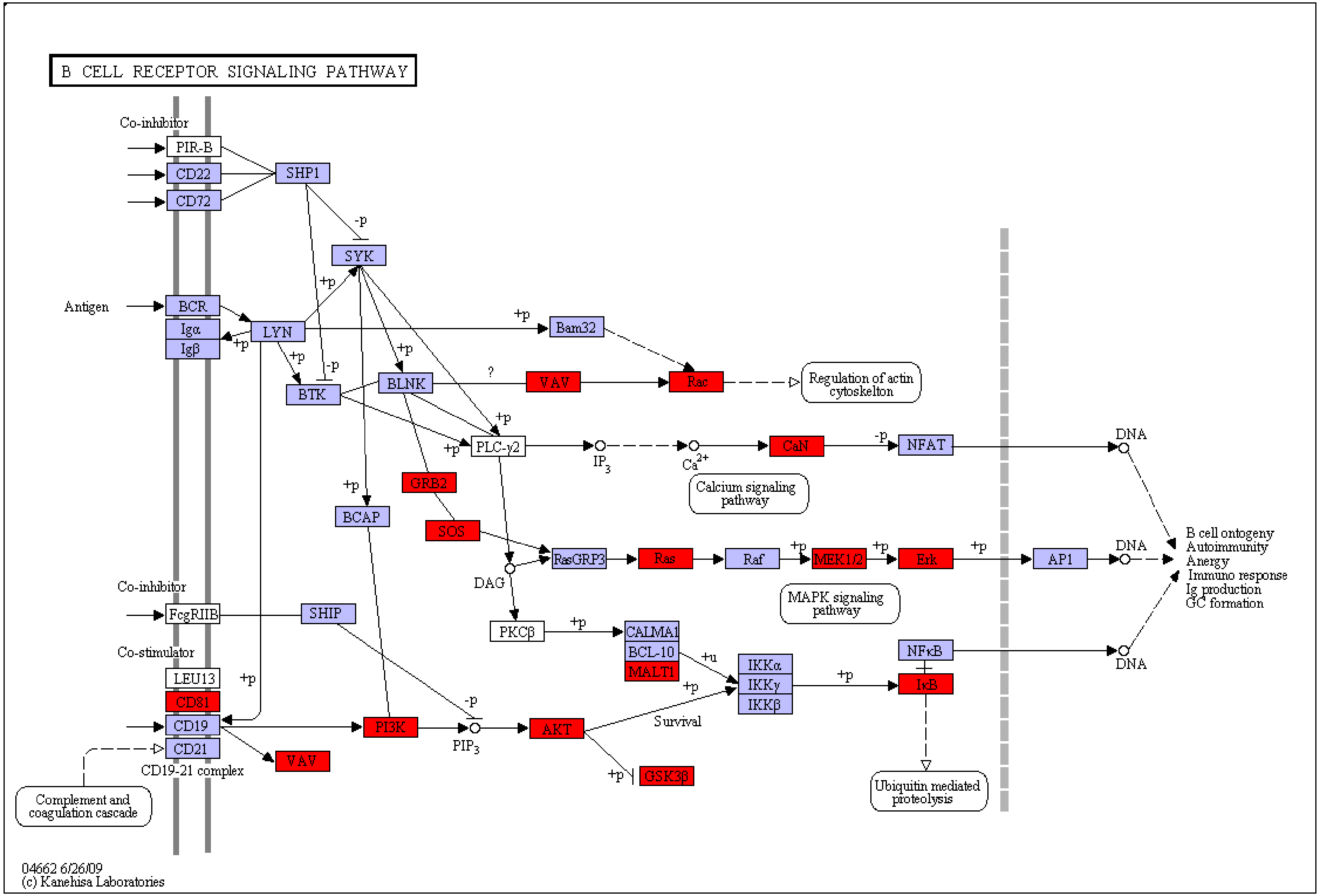

Figure 86: ko04662 B cell receptor signaling pathway

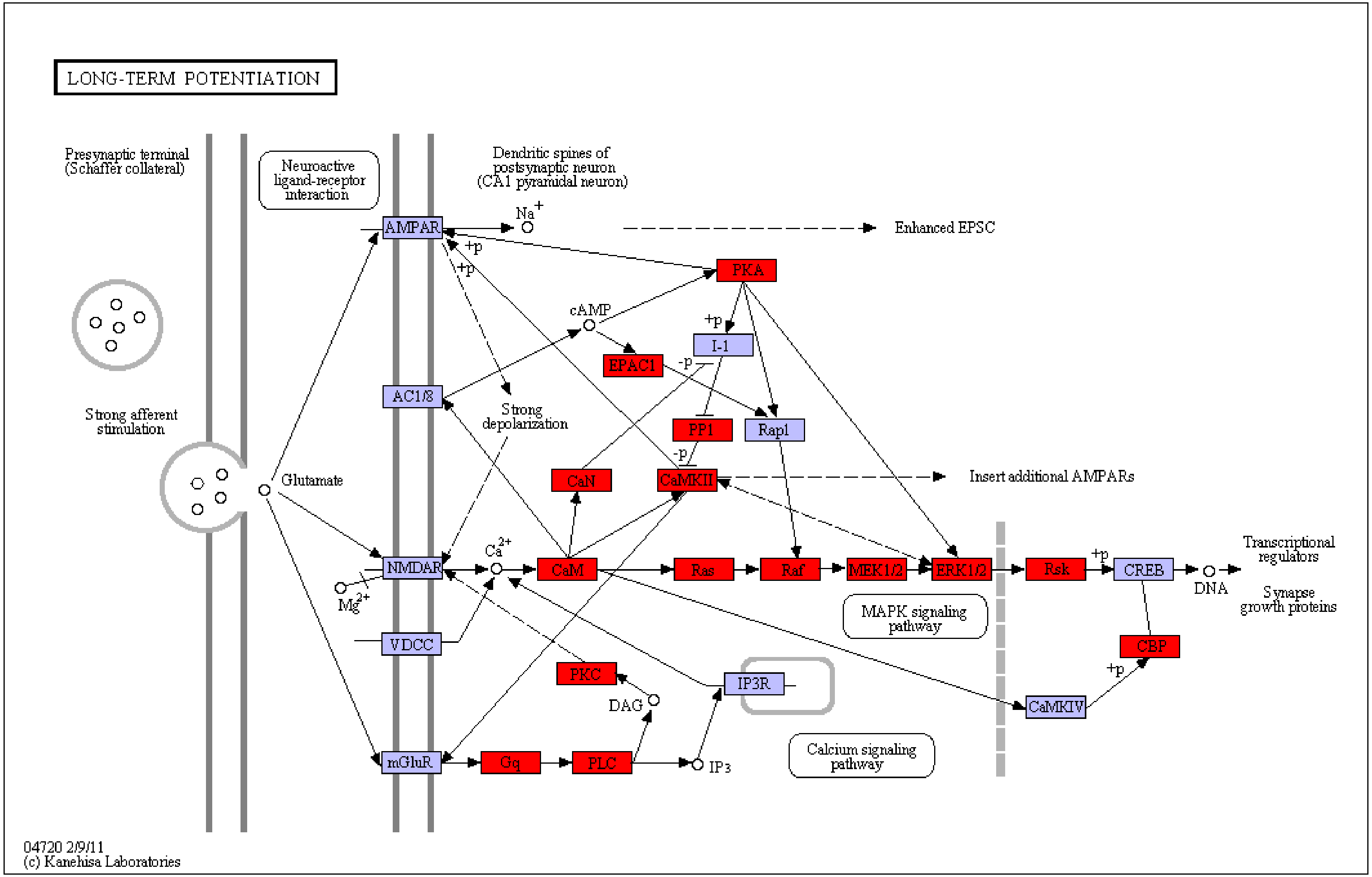

Figure 87: ko04720 Long-term potentiation

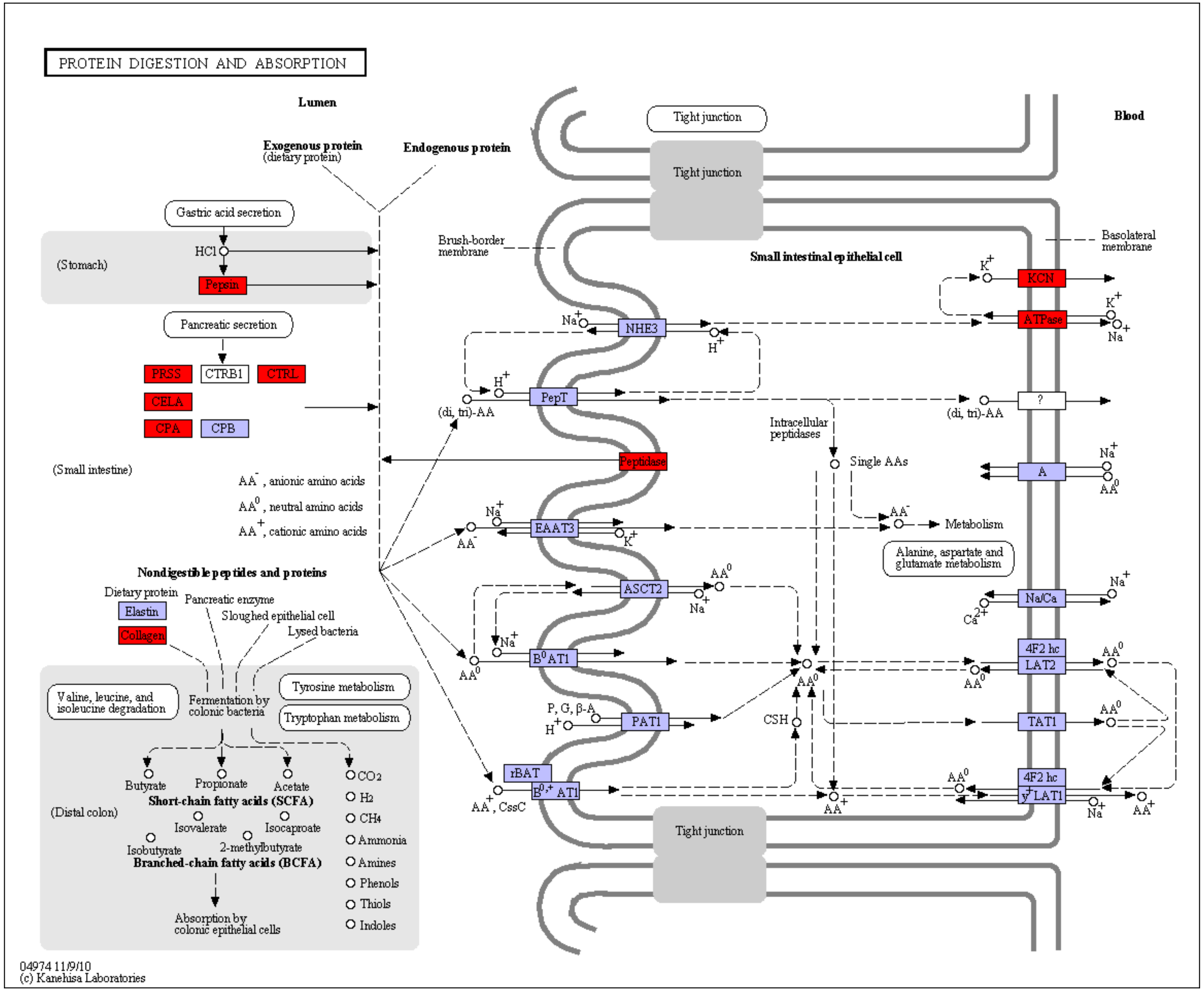

Figure 88: ko04974 Protein digestion and absorption

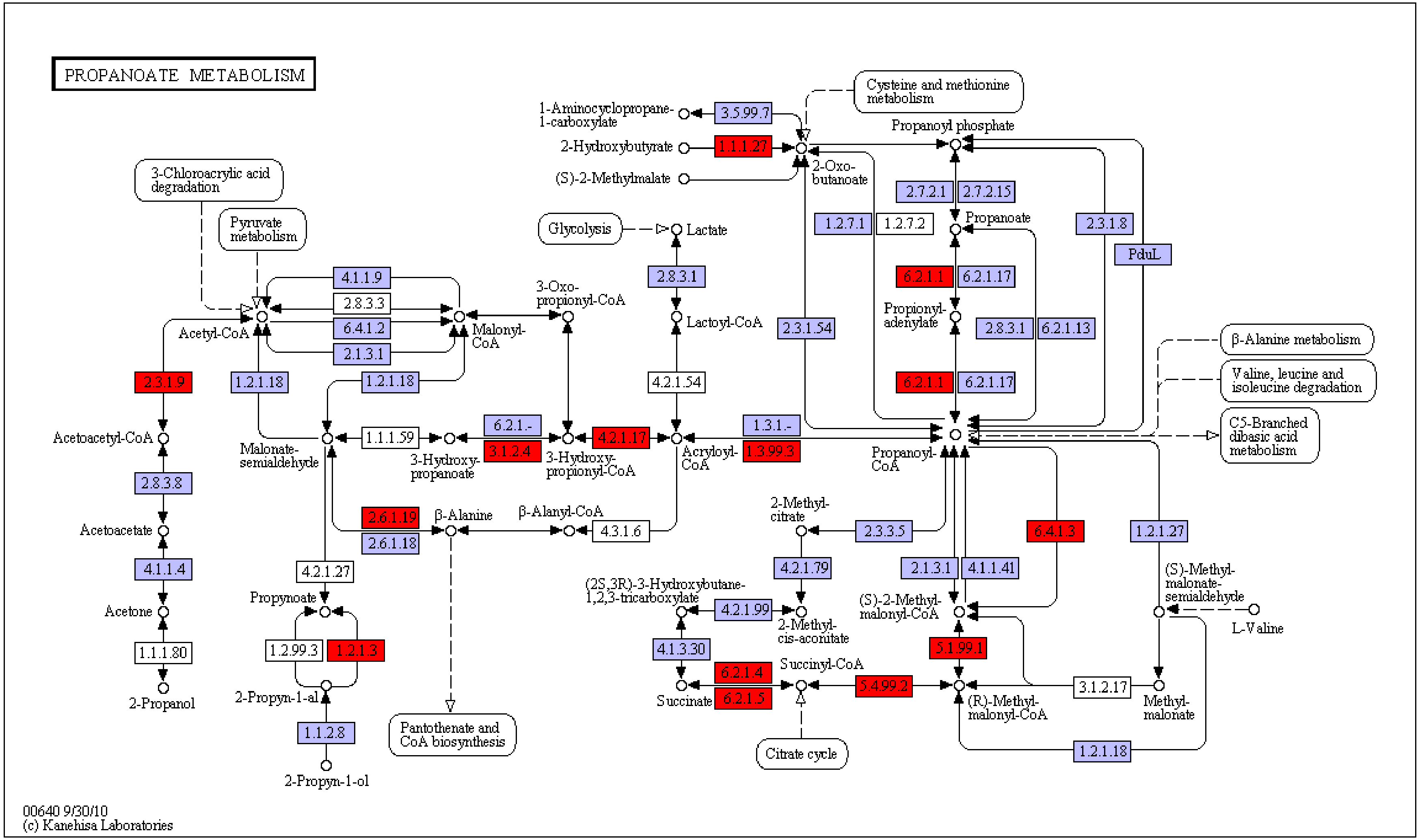

Figure 89: ko00640 Propanoate metabolism

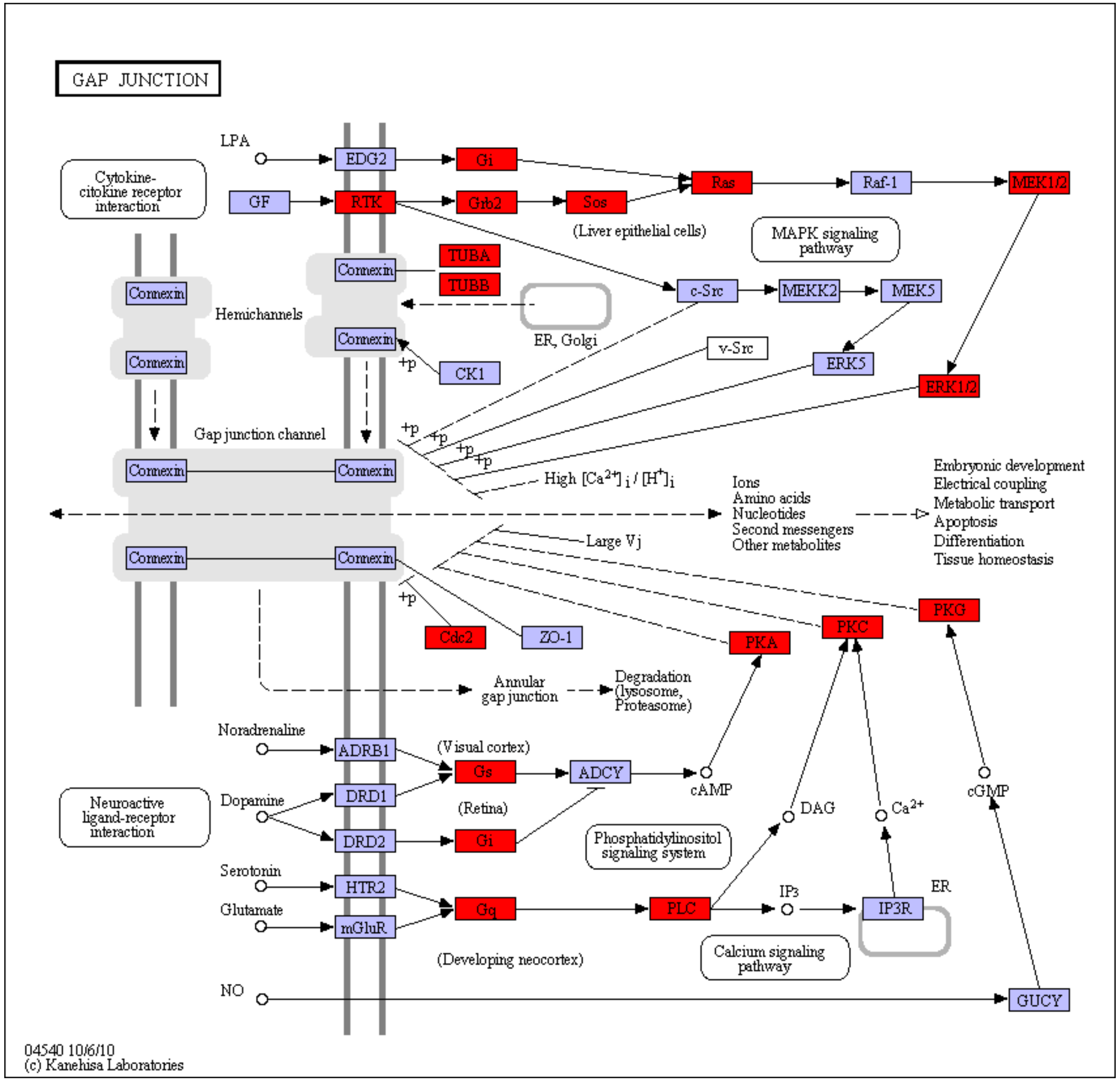

Figure 90: ko04540 Gap junction

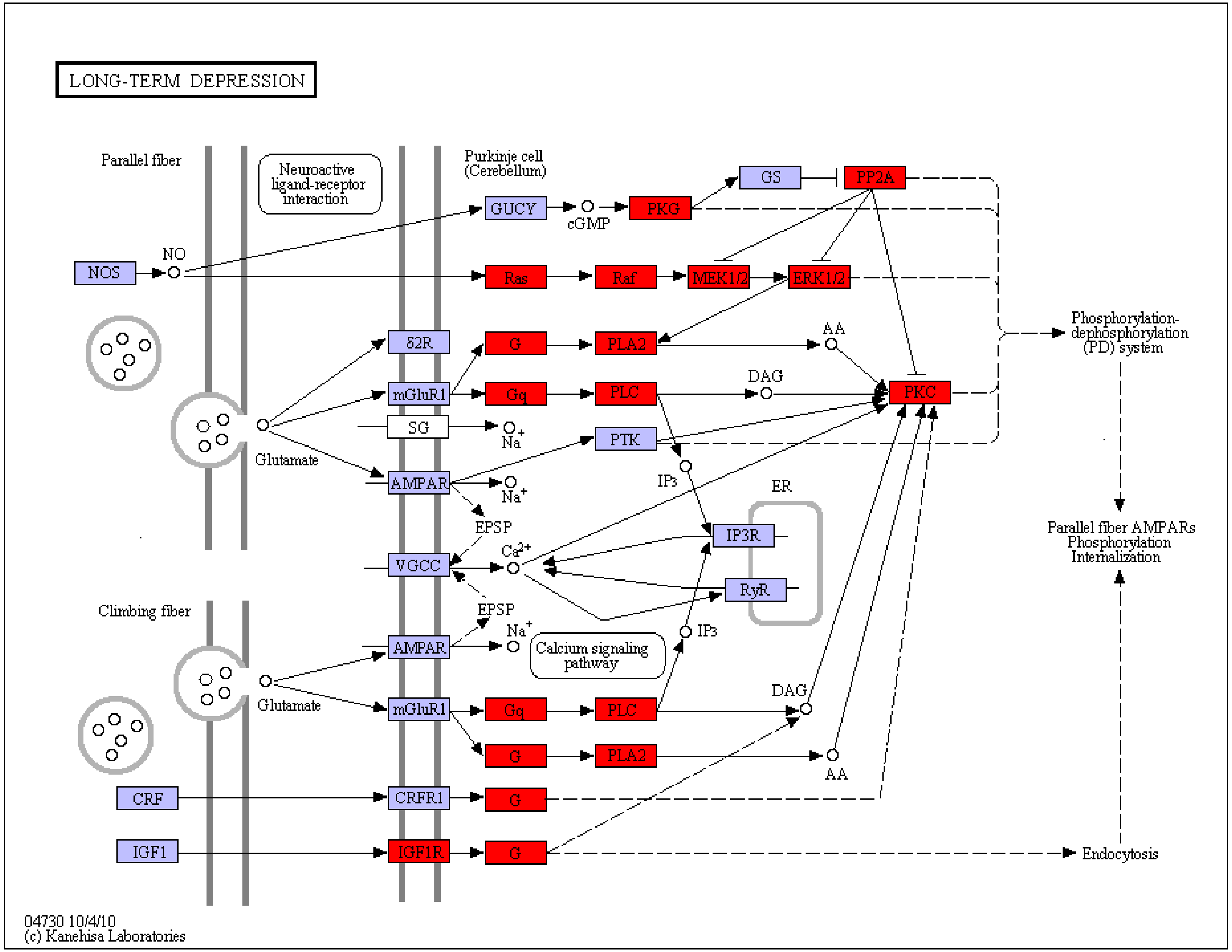

Figure 91: ko04730 Long-term depression

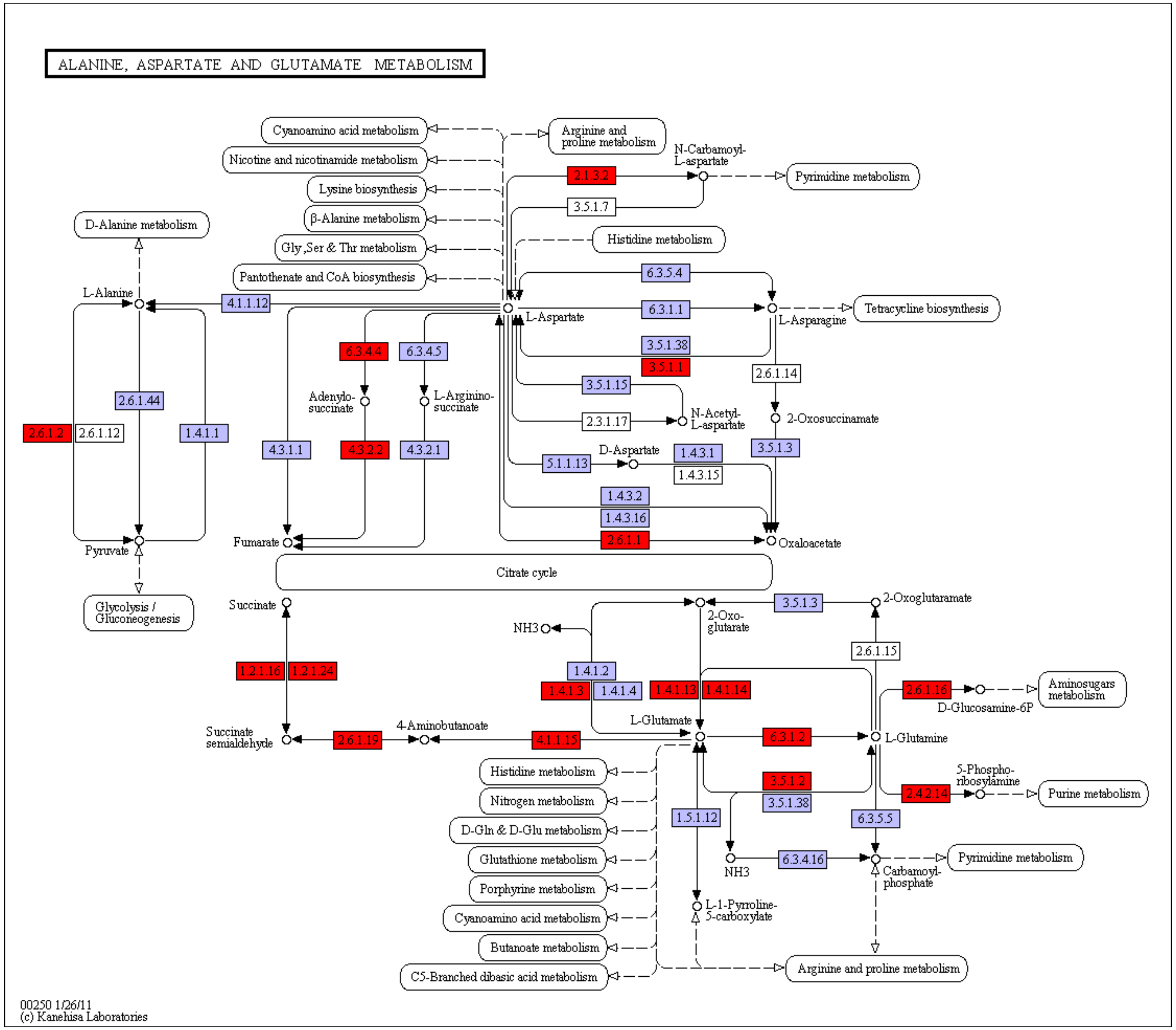

Figure 92: ko00250 Alanine, aspartate and glutamate metabolism

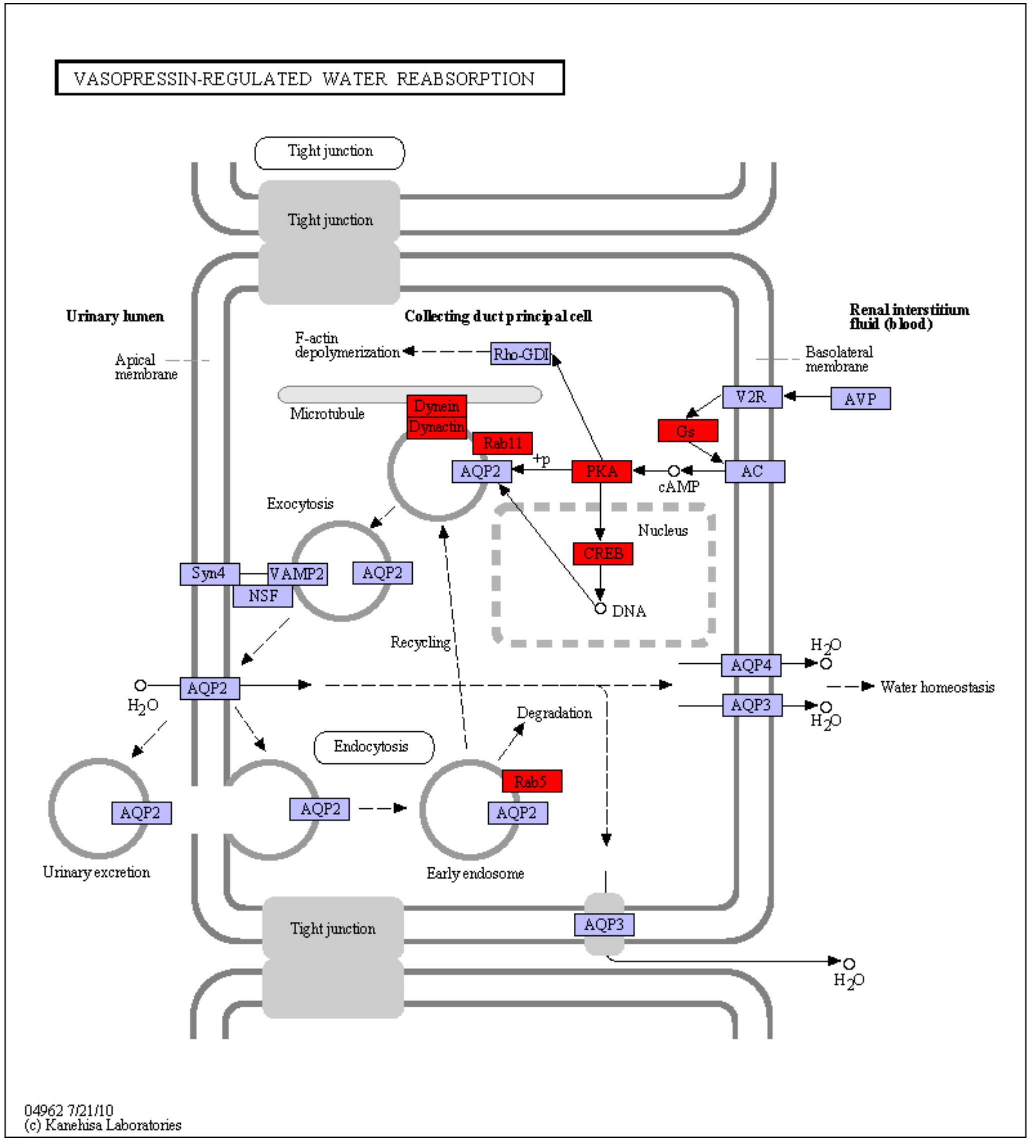

Figure 93: ko04962 Vasopressin-regulated water reabsorption

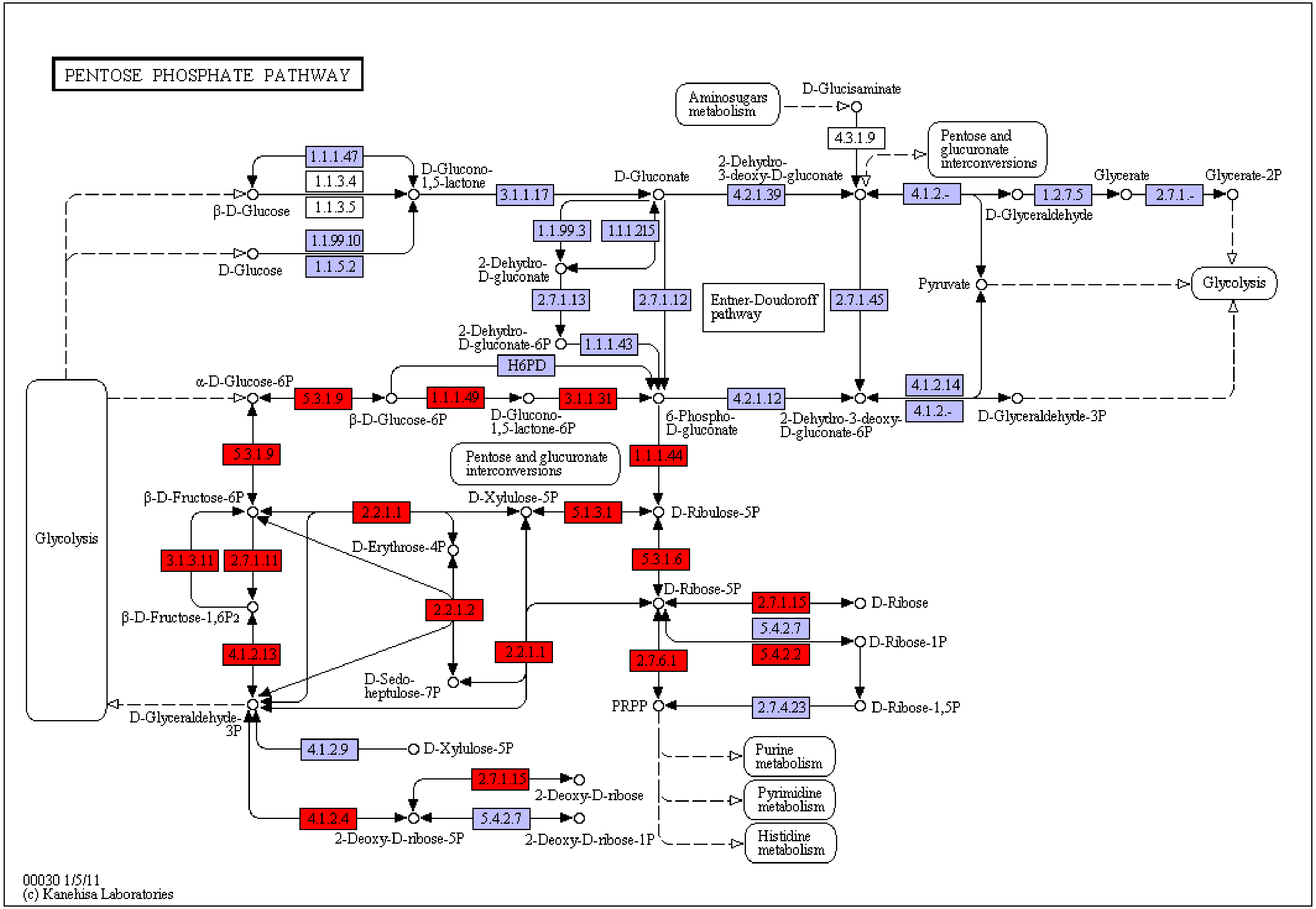

Figure 94: ko00030 Pentose phosphate pathway

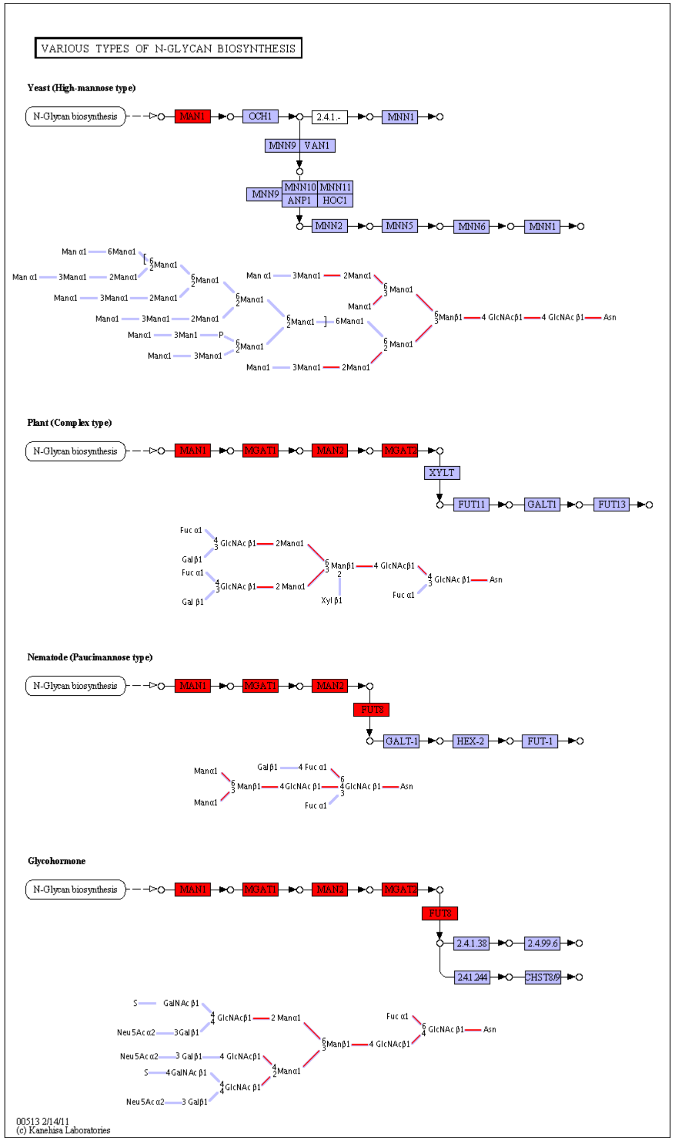

Figure 95: ko00513 Various types of N-glycan biosynthesis

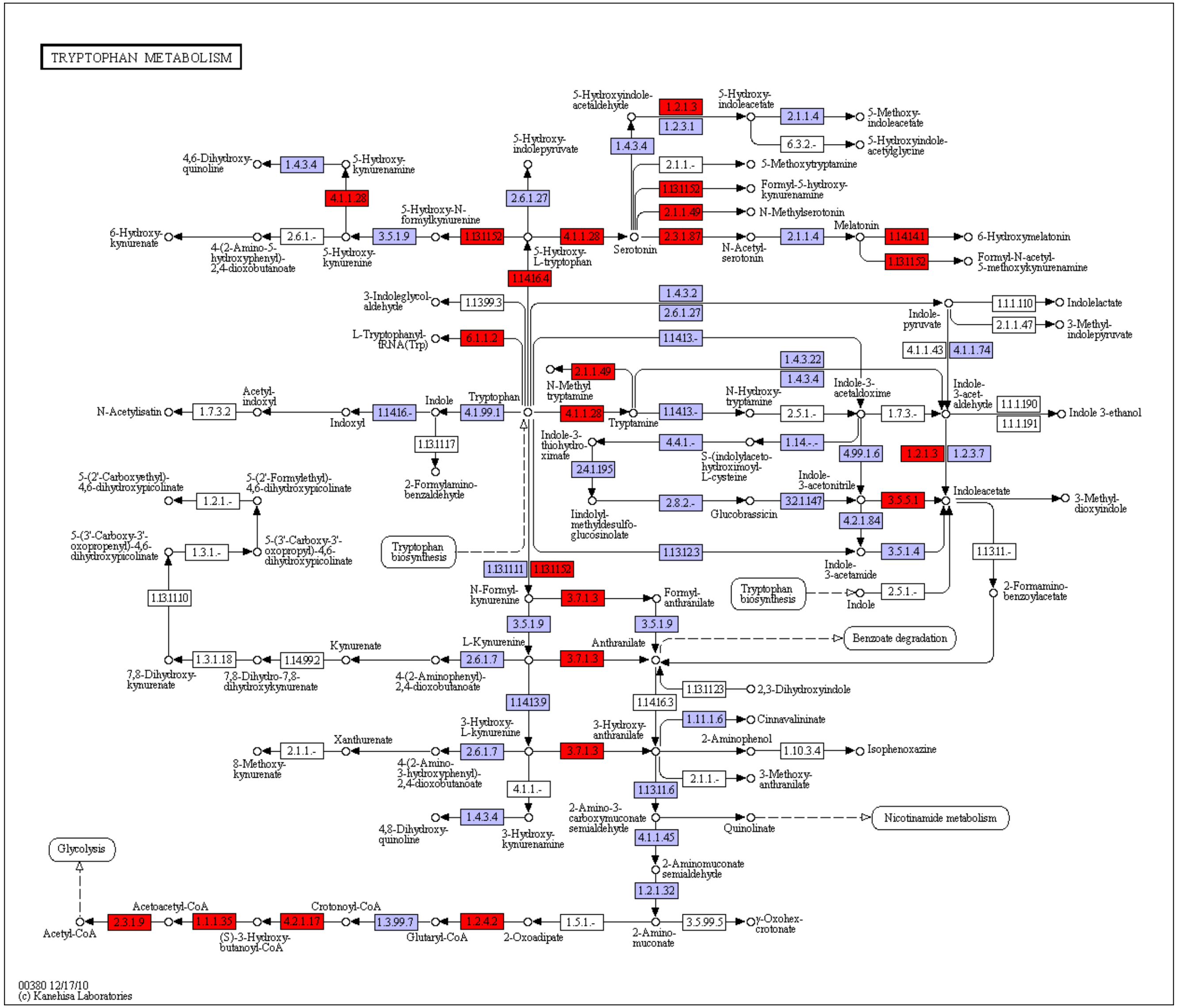

Figure 96: ko00380 Tryptophan metabolism

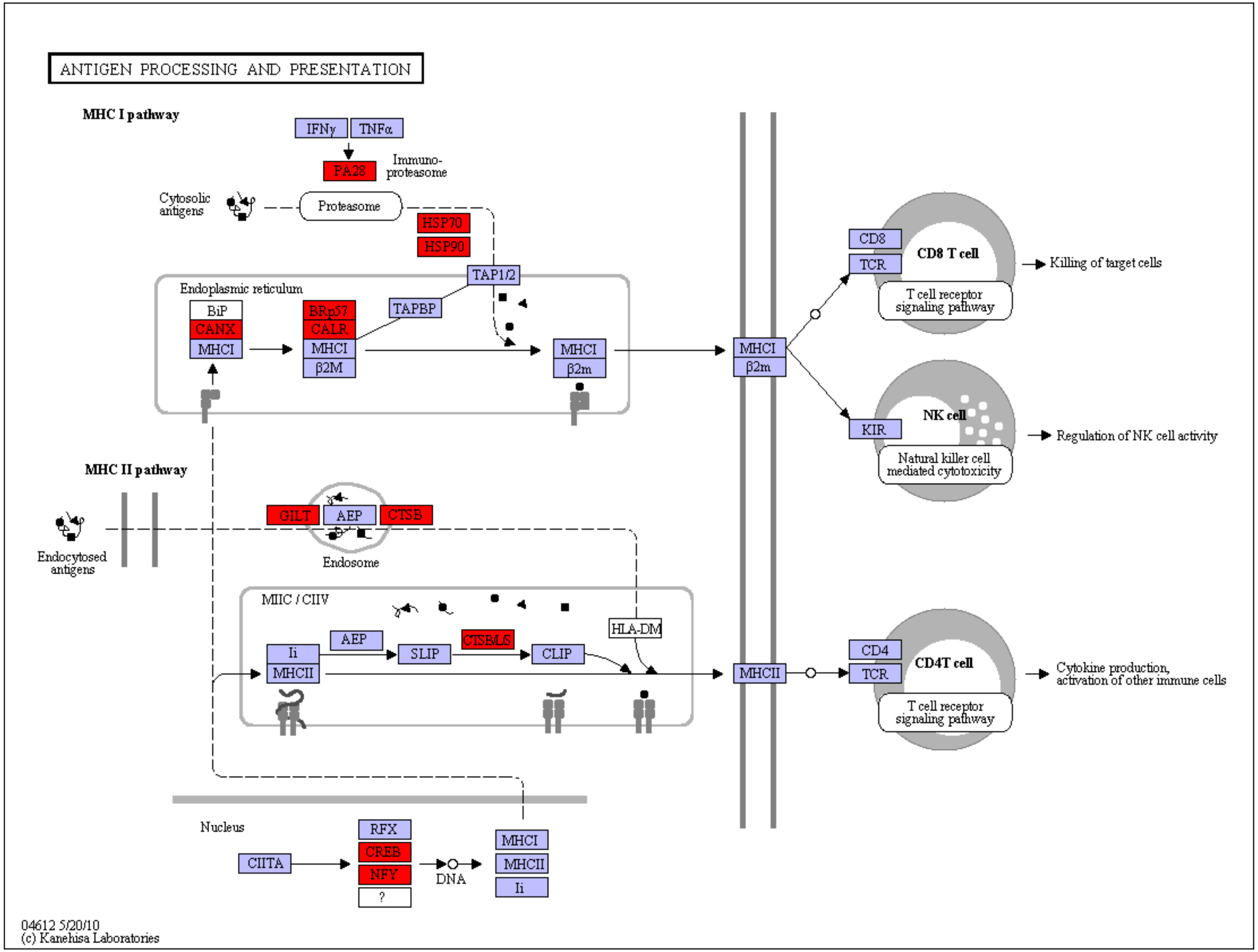

Figure 97: ko04612 Antigen processing and presentation

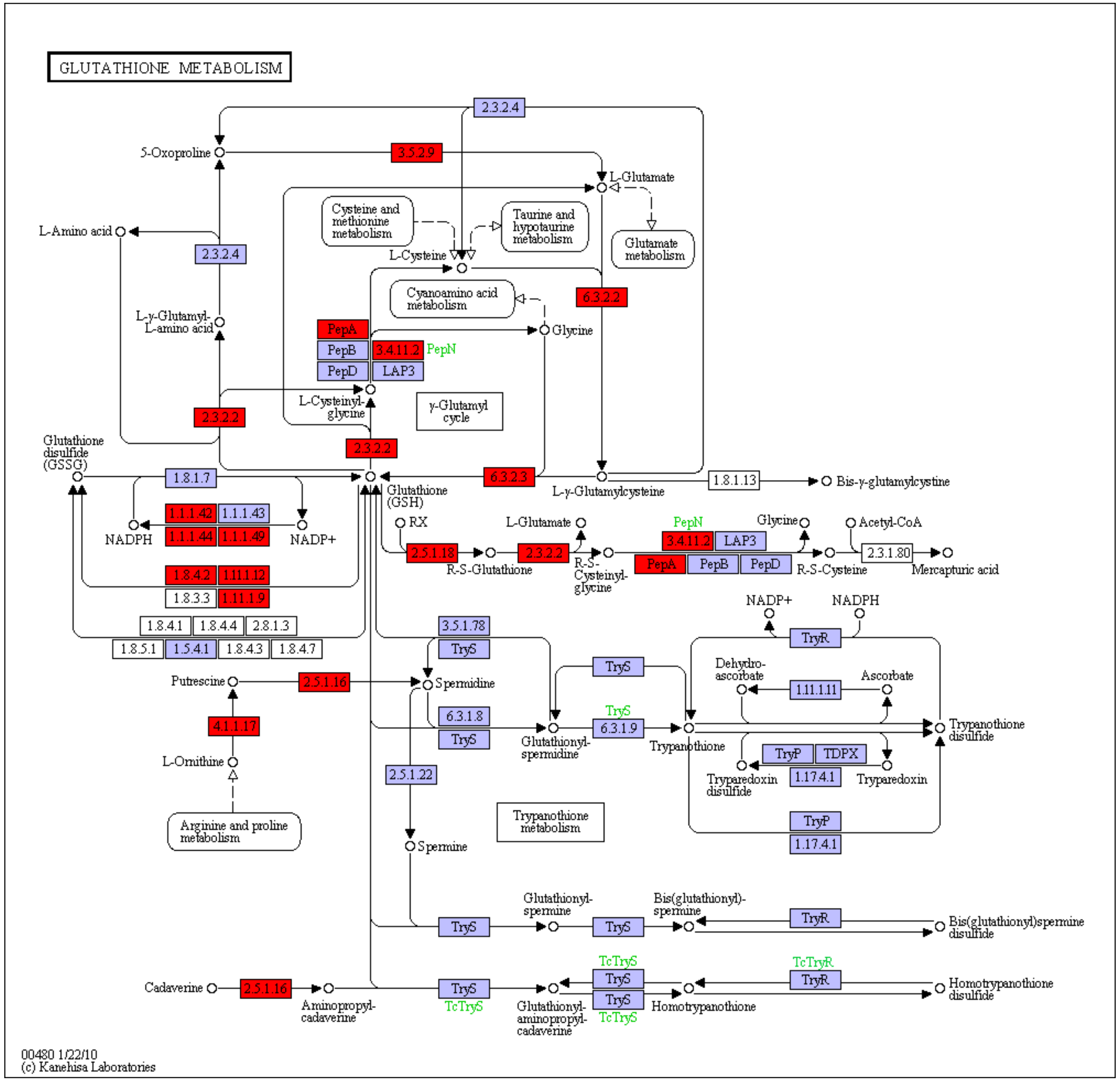

Figure 98: ko00480 Glutathione metabolism

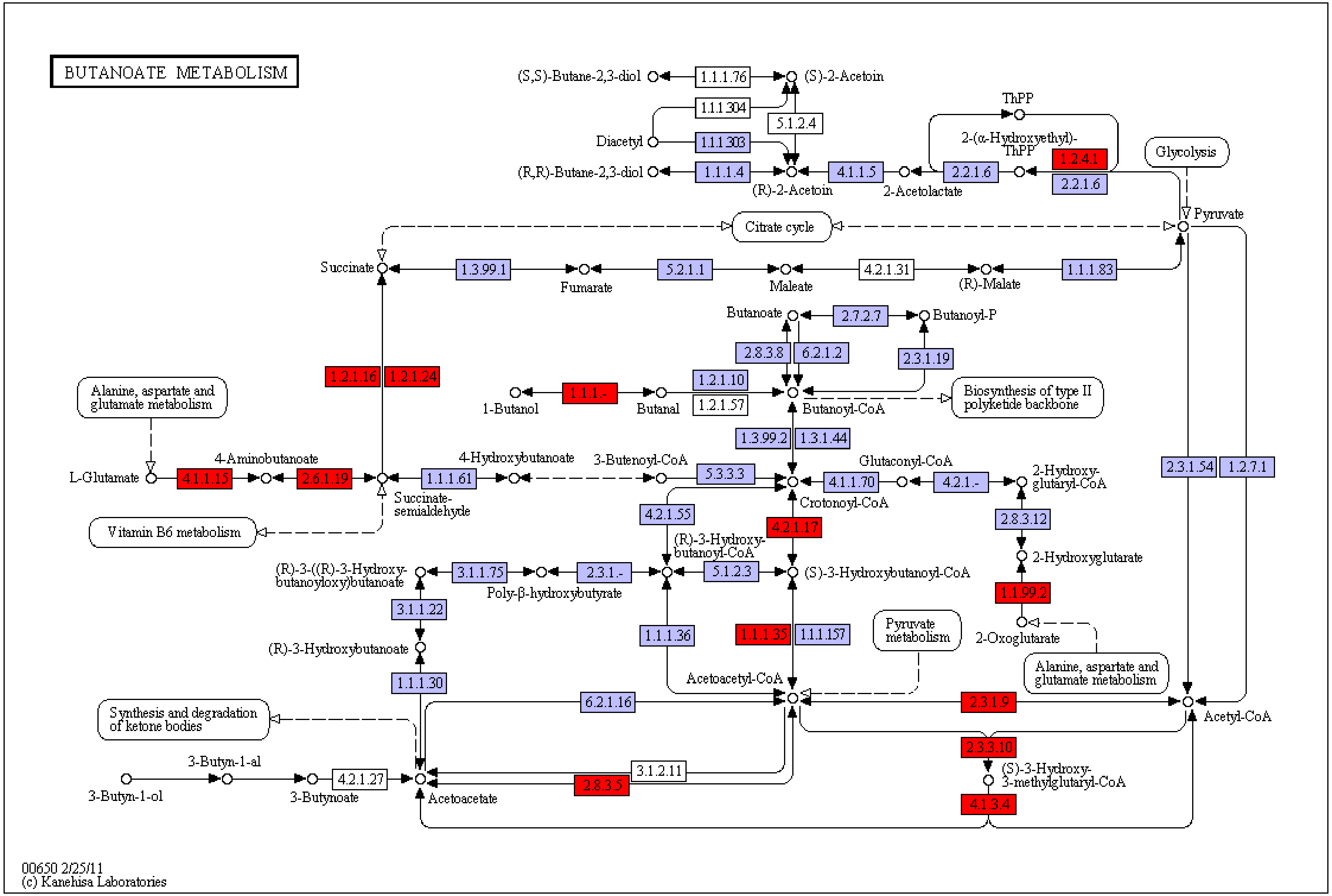

Figure 99: ko00650 Butanoate metabolism

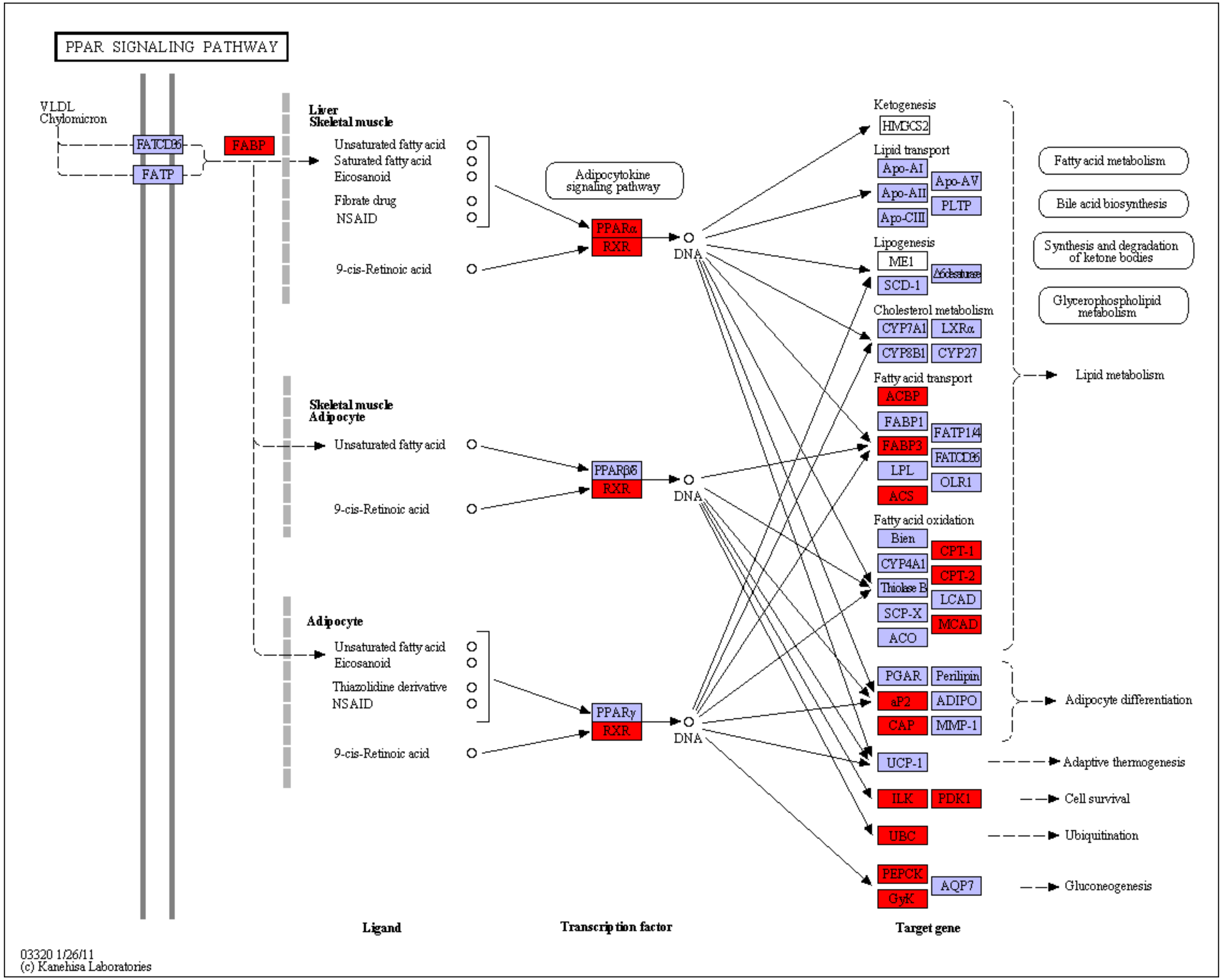

Figure 100: ko03320 PPAR signaling pathway

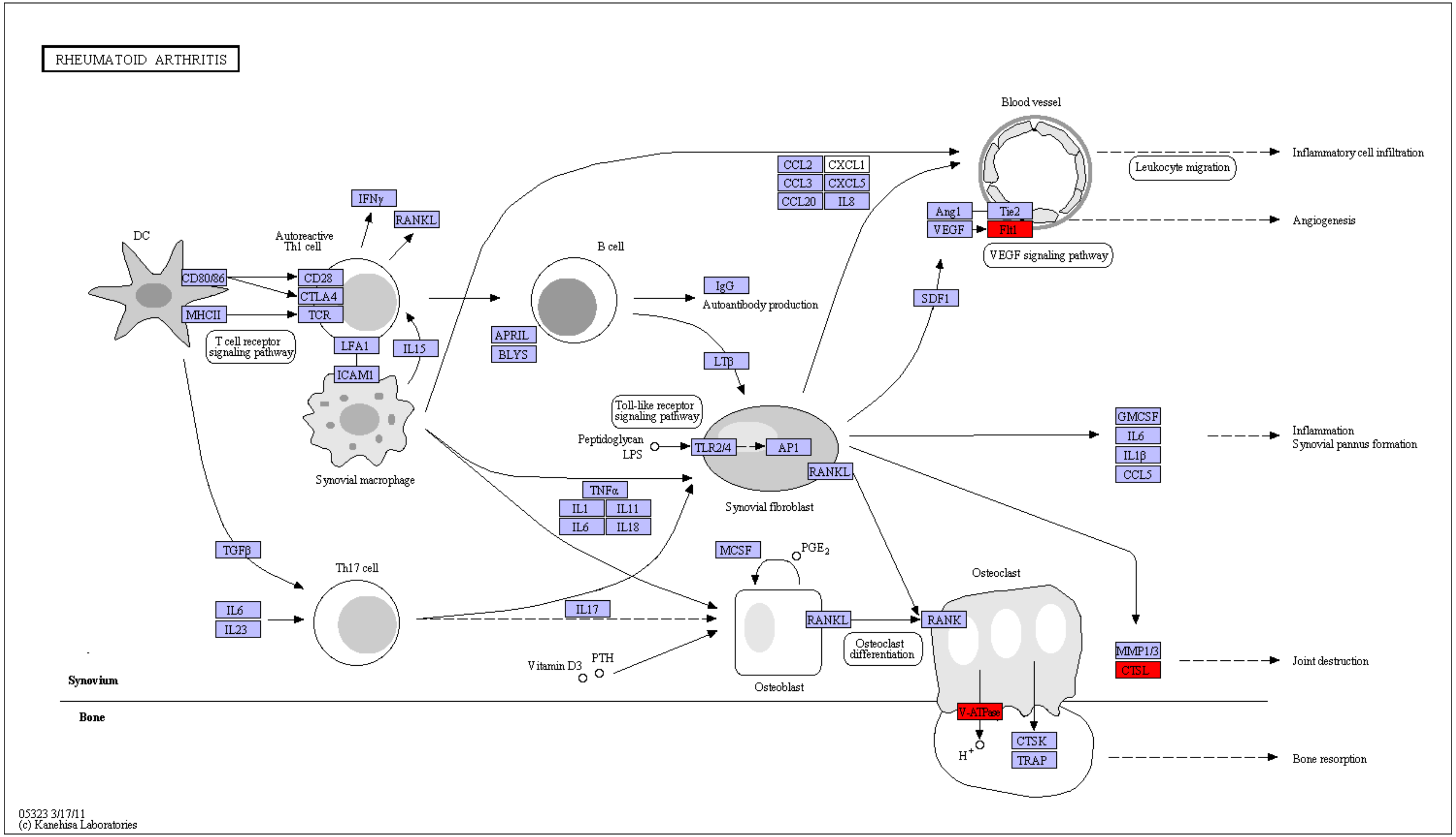

Figure 101: ko05323 Rheumatoid arthritis

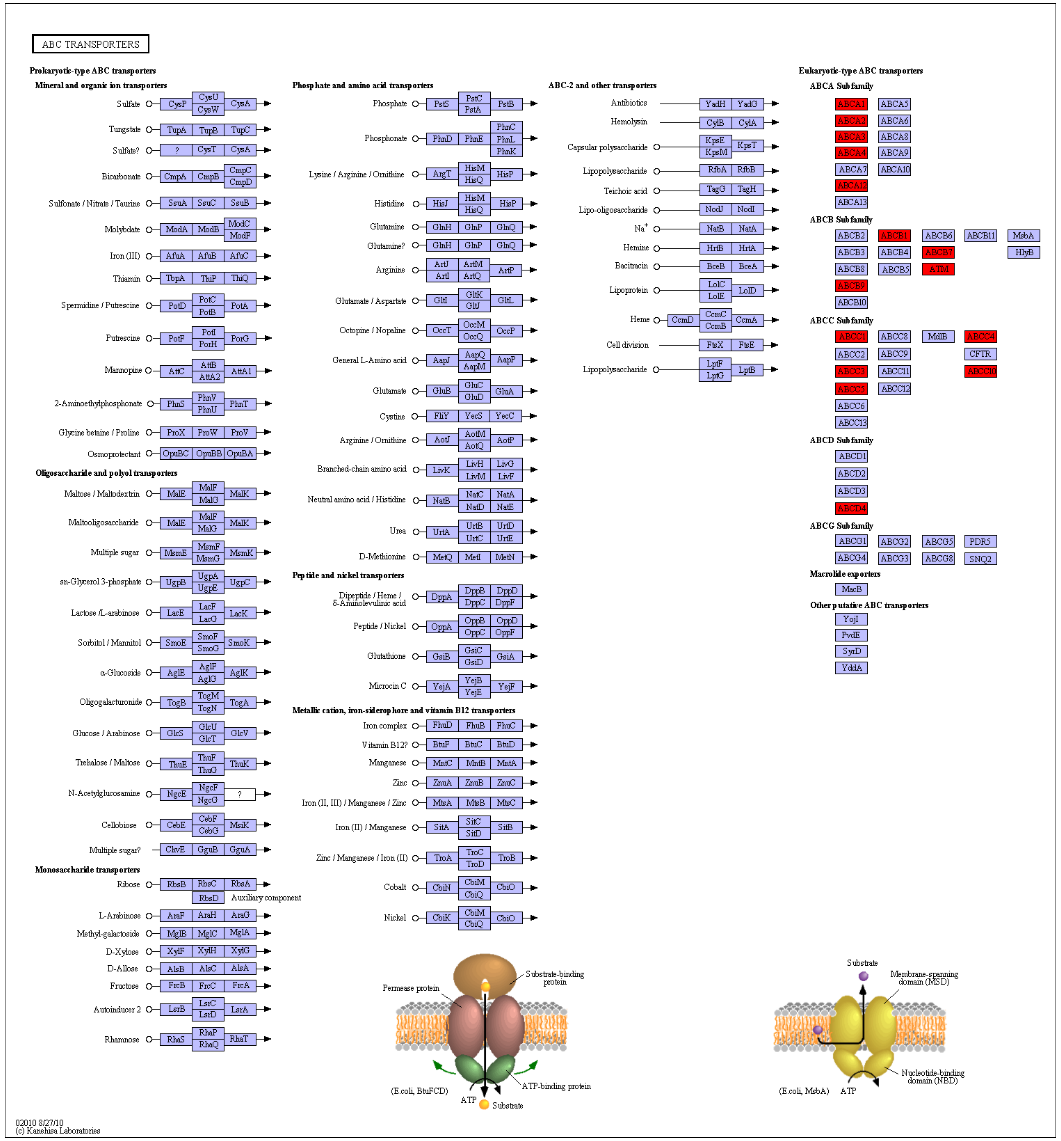

Figure 102: ko02010 ABC transporters

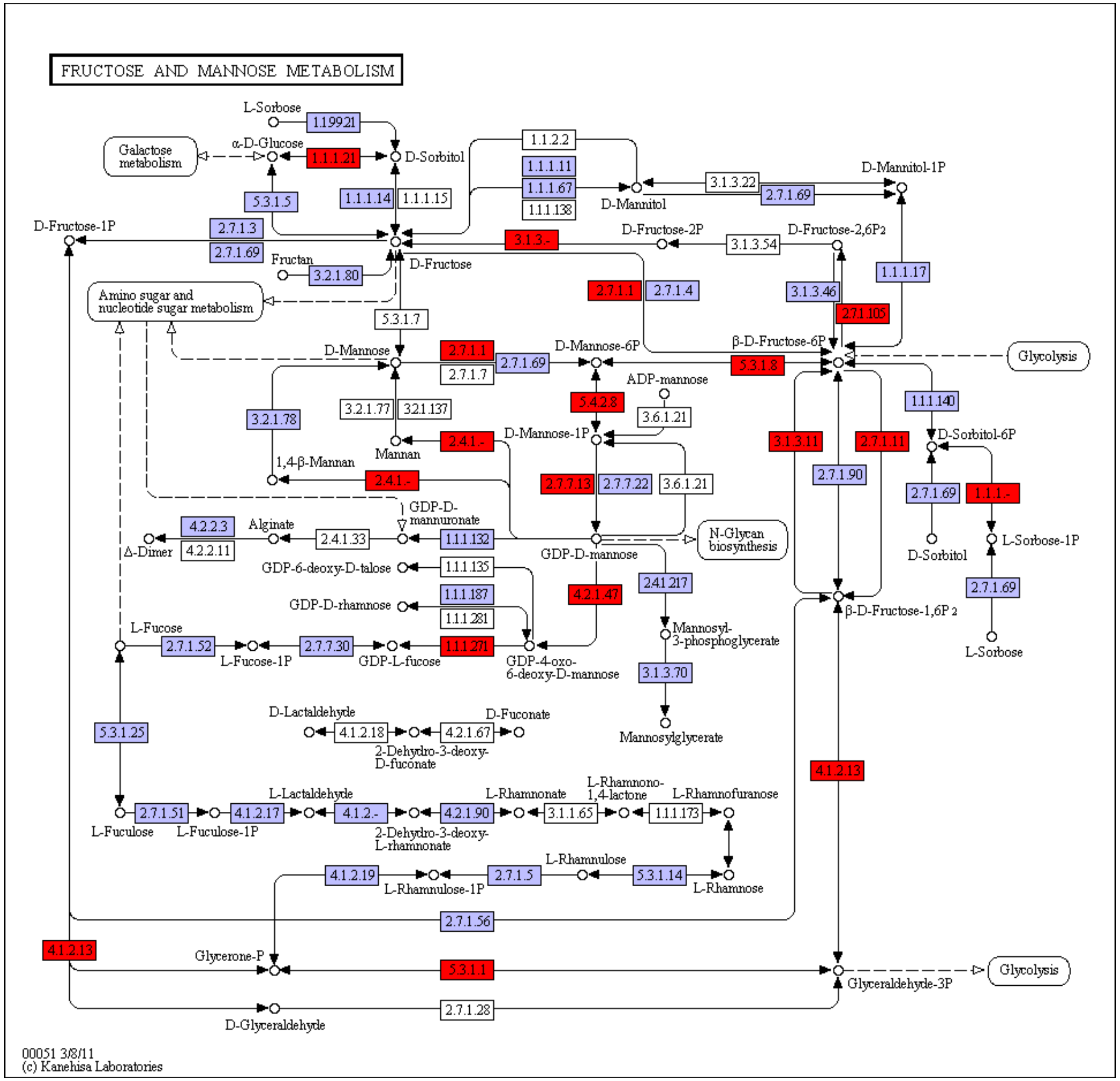

Figure 103: ko00051 Fructose and mannose metabolism

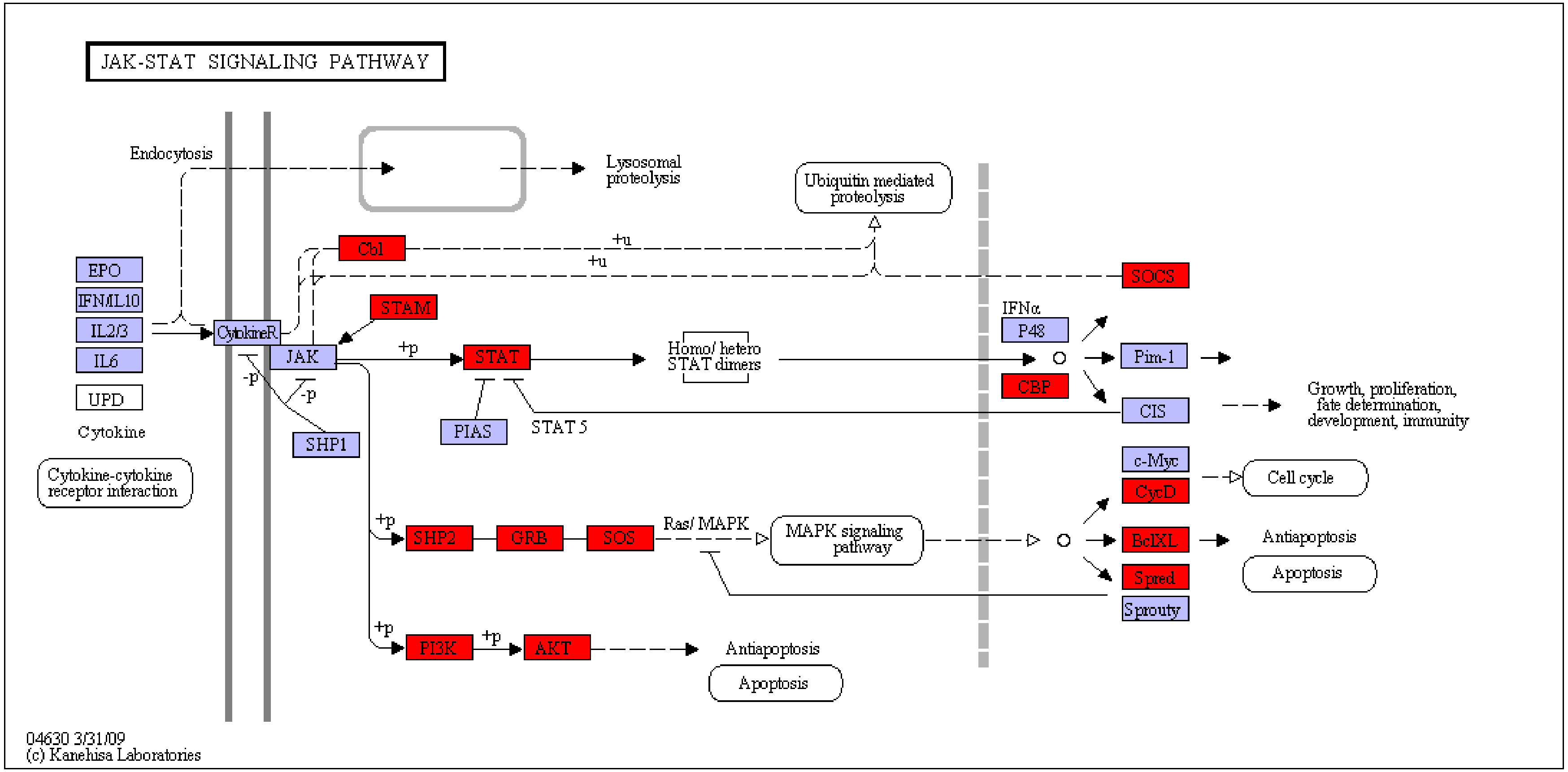

Figure 104: ko04630 Jak-STAT signaling pathway

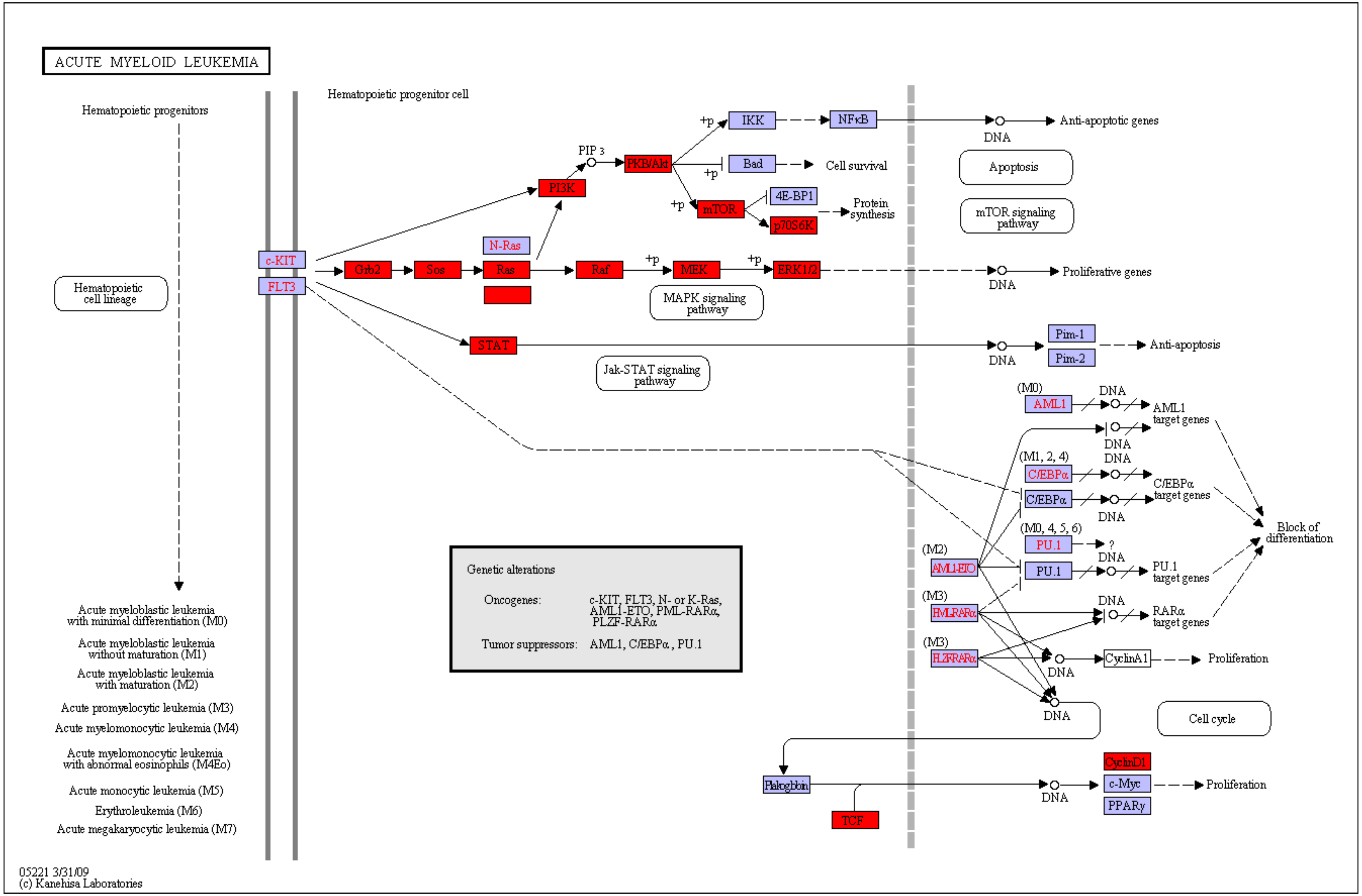

Figure 105: ko05221 Acute myeloid leukemia

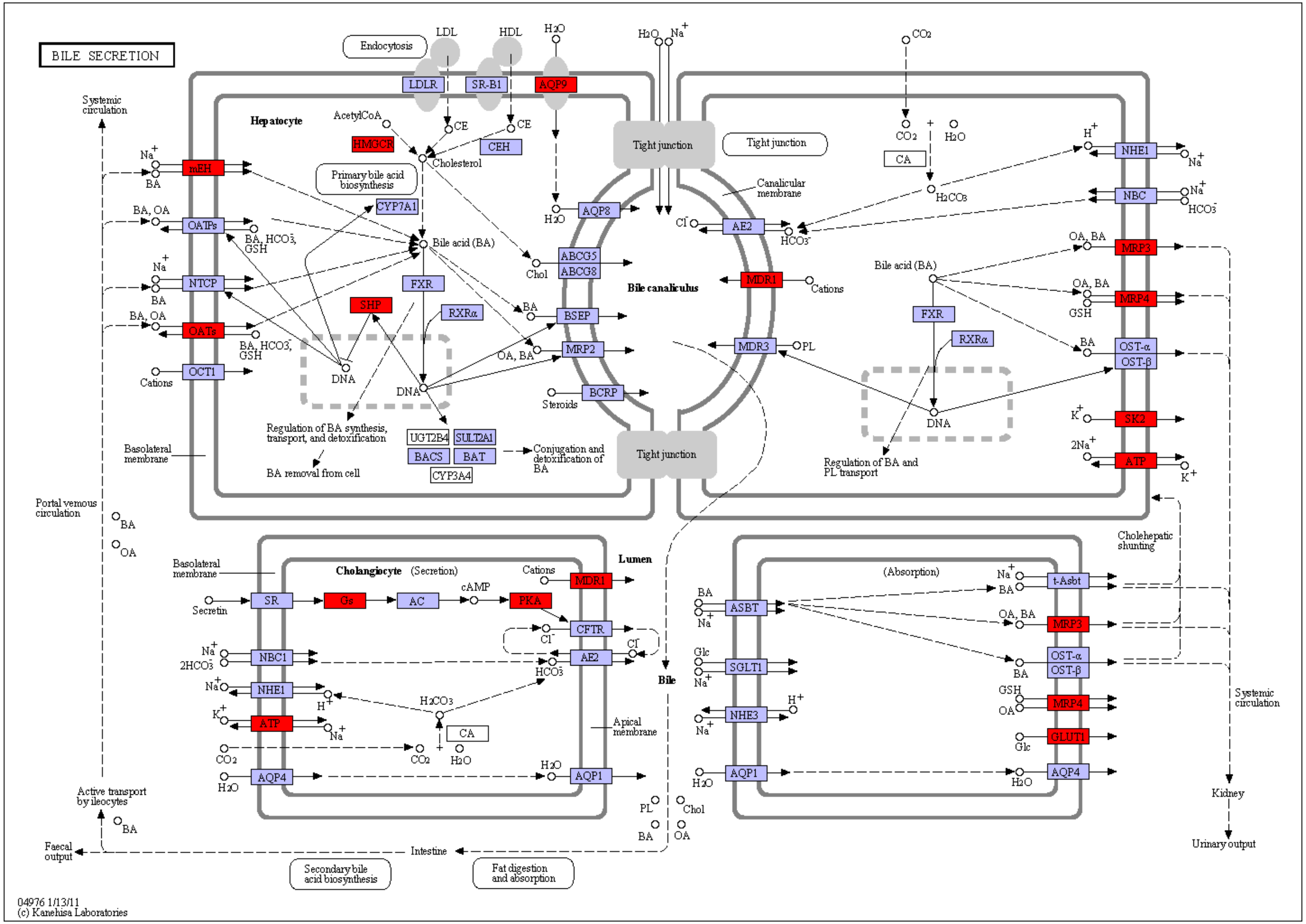

Figure 106: ko04976 Bile secretion

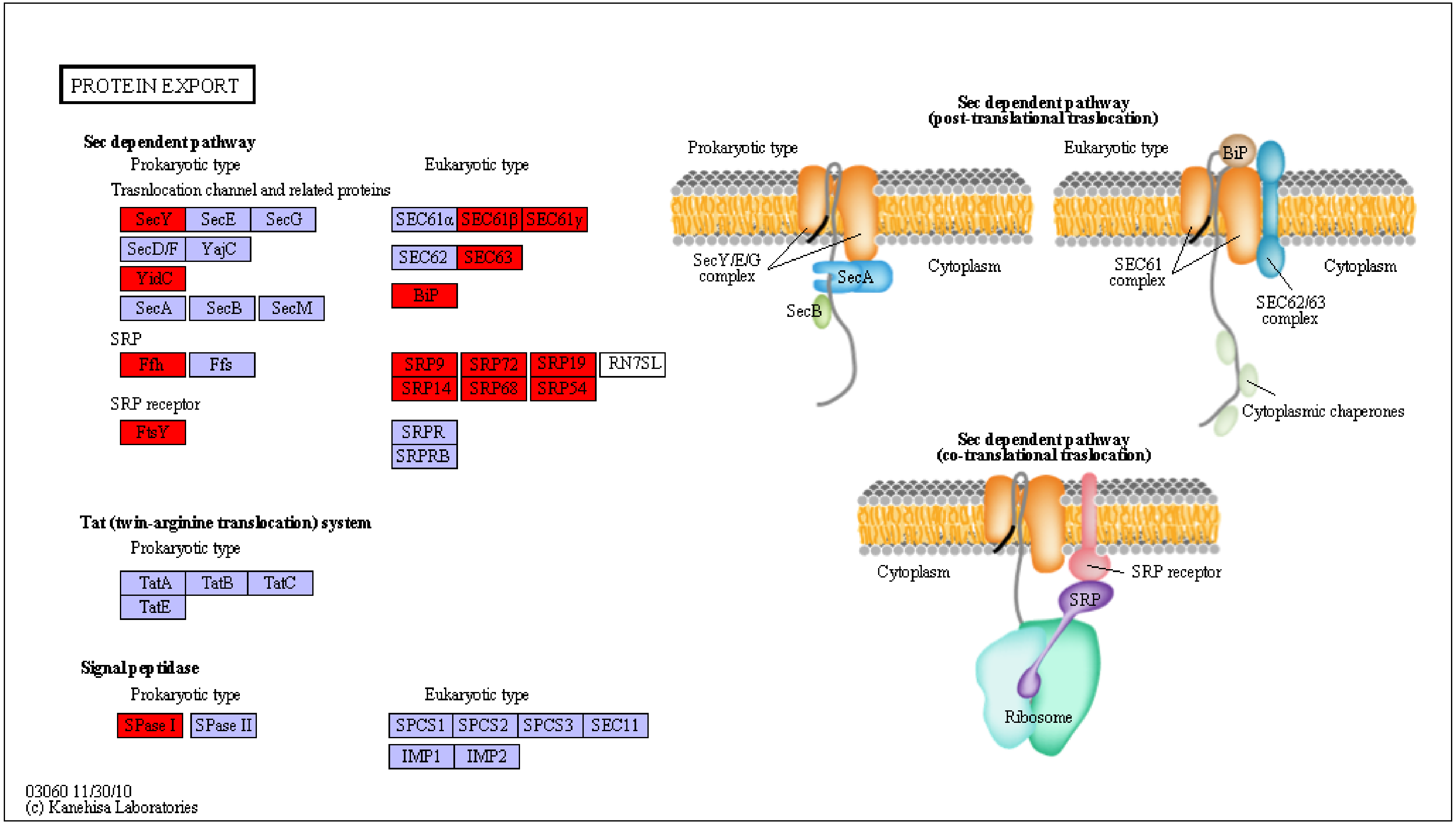

Figure 107: ko03060 Protein export

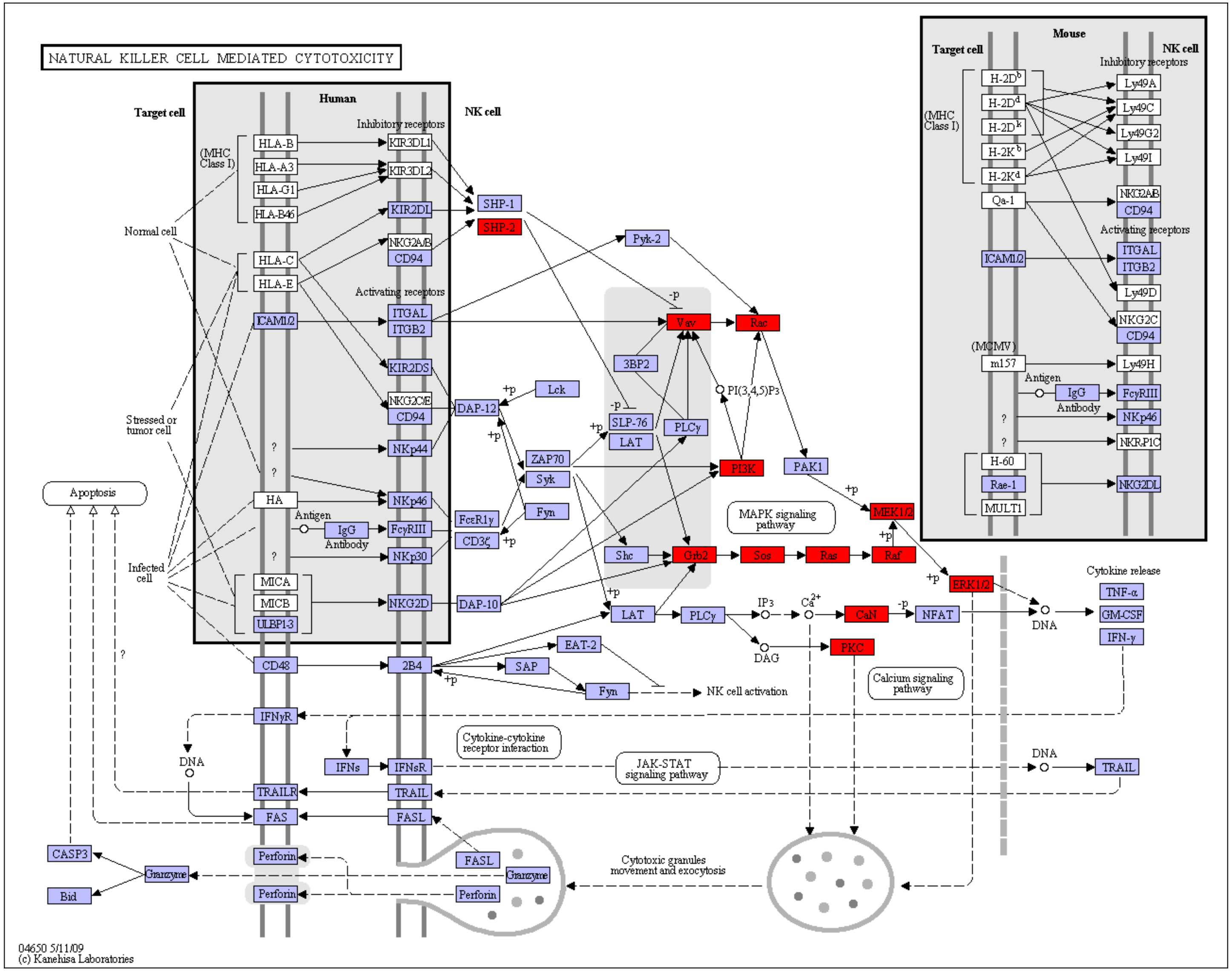

Figure 108: ko04650 Natural killer cell mediated cytotoxicity

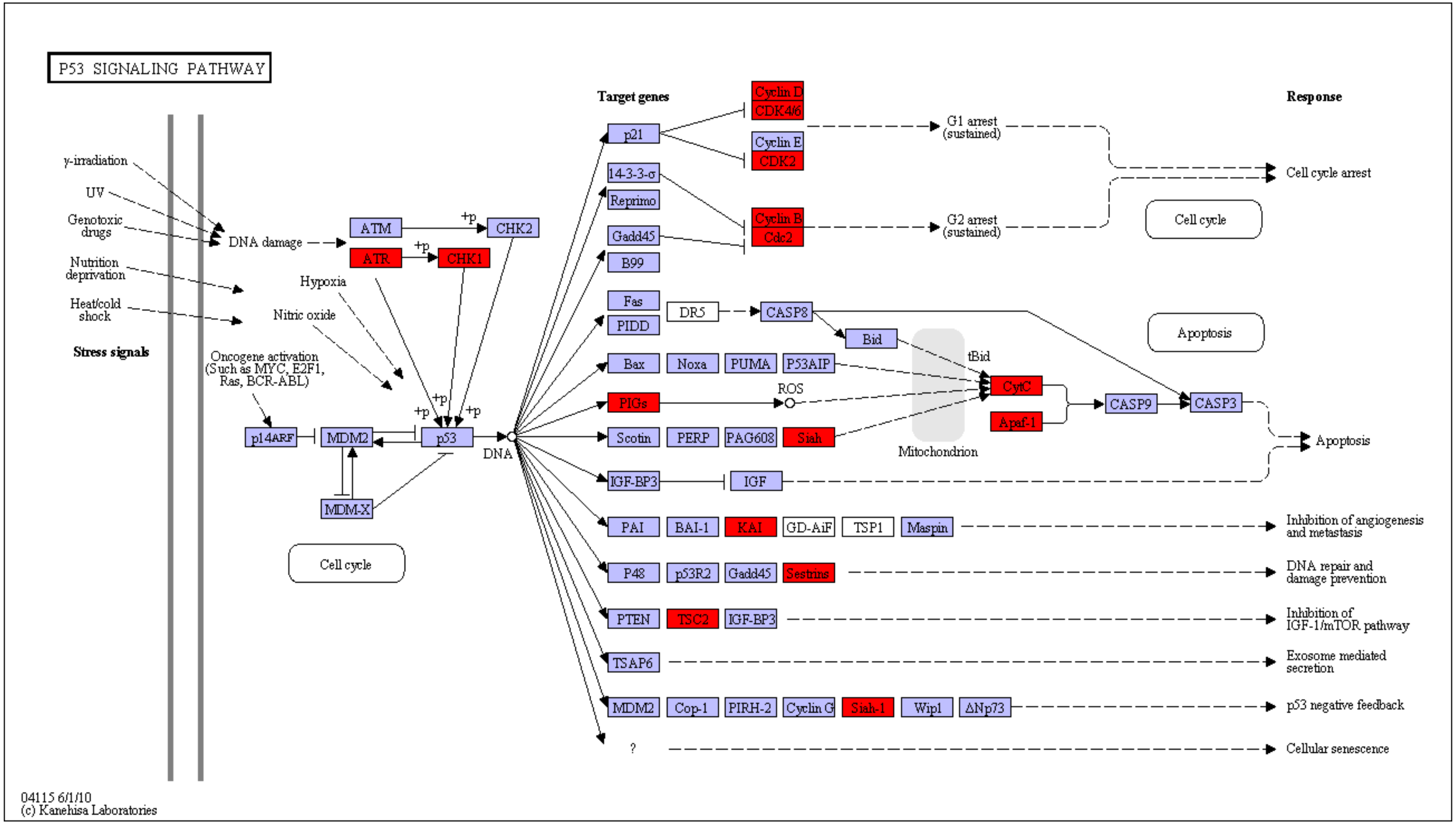

Figure 109: ko04115 p53 signaling pathway

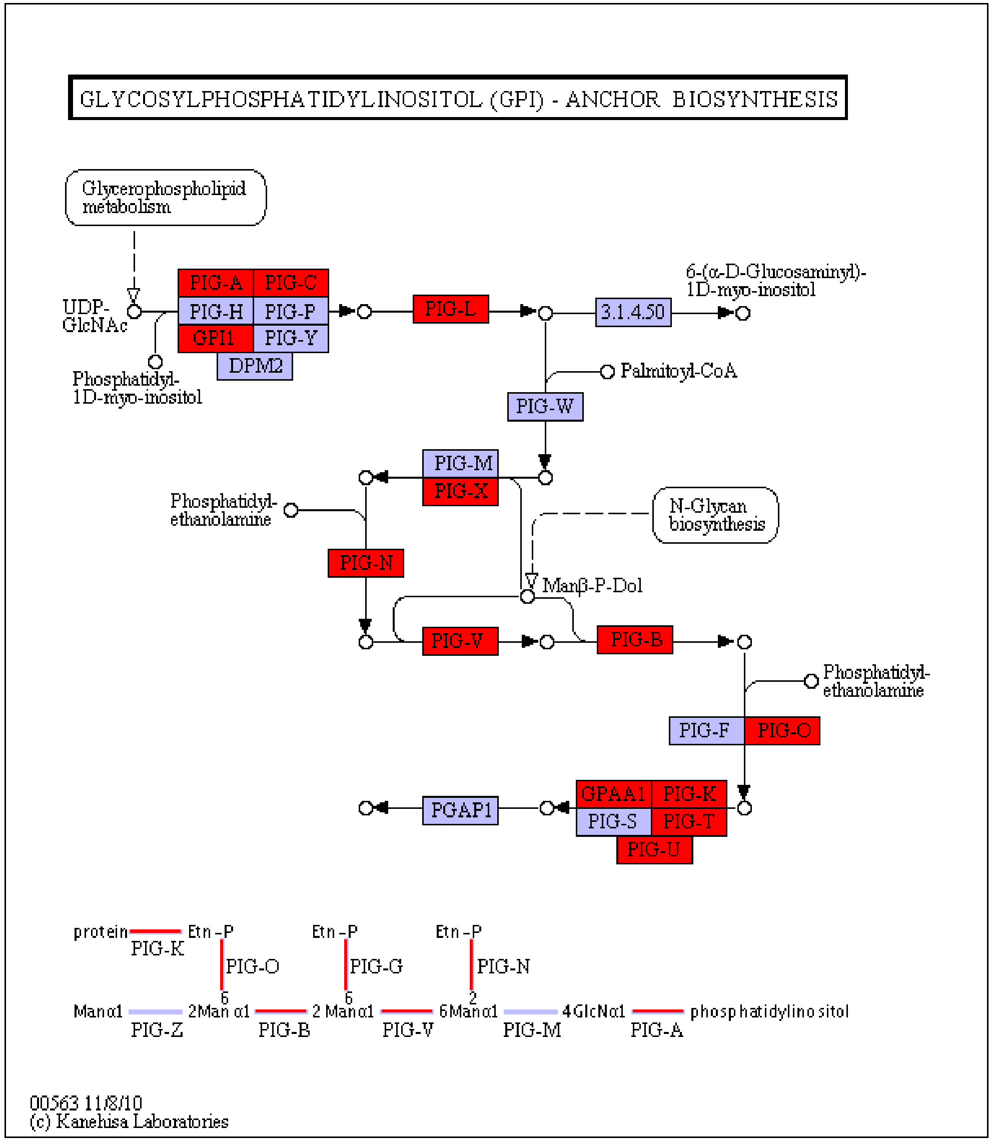

Figure 110: ko00563 Glycosylphosphatidylinositol(GPI)-anchor biosynthesis

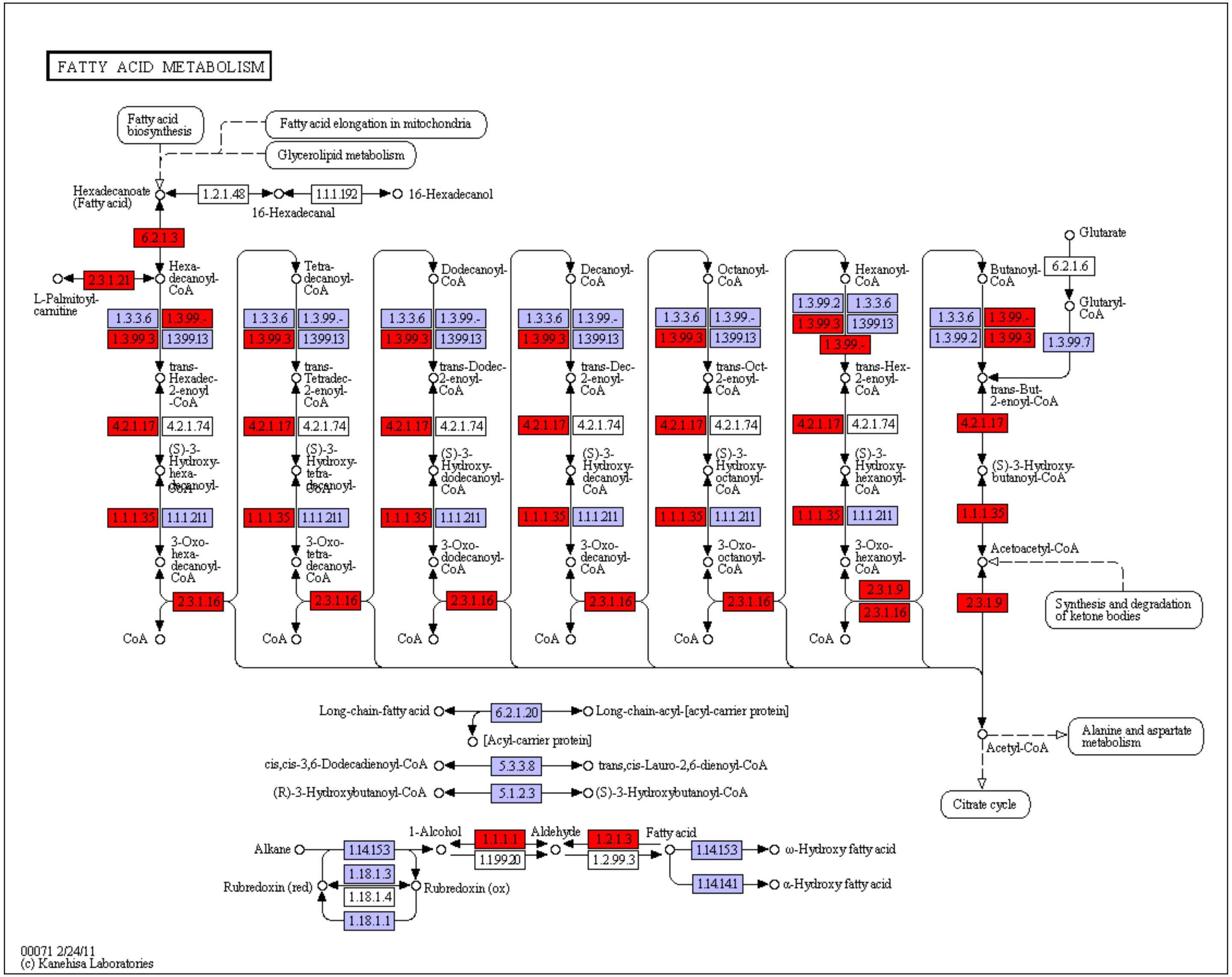

Figure 111: ko00071 Fatty acid metabolism

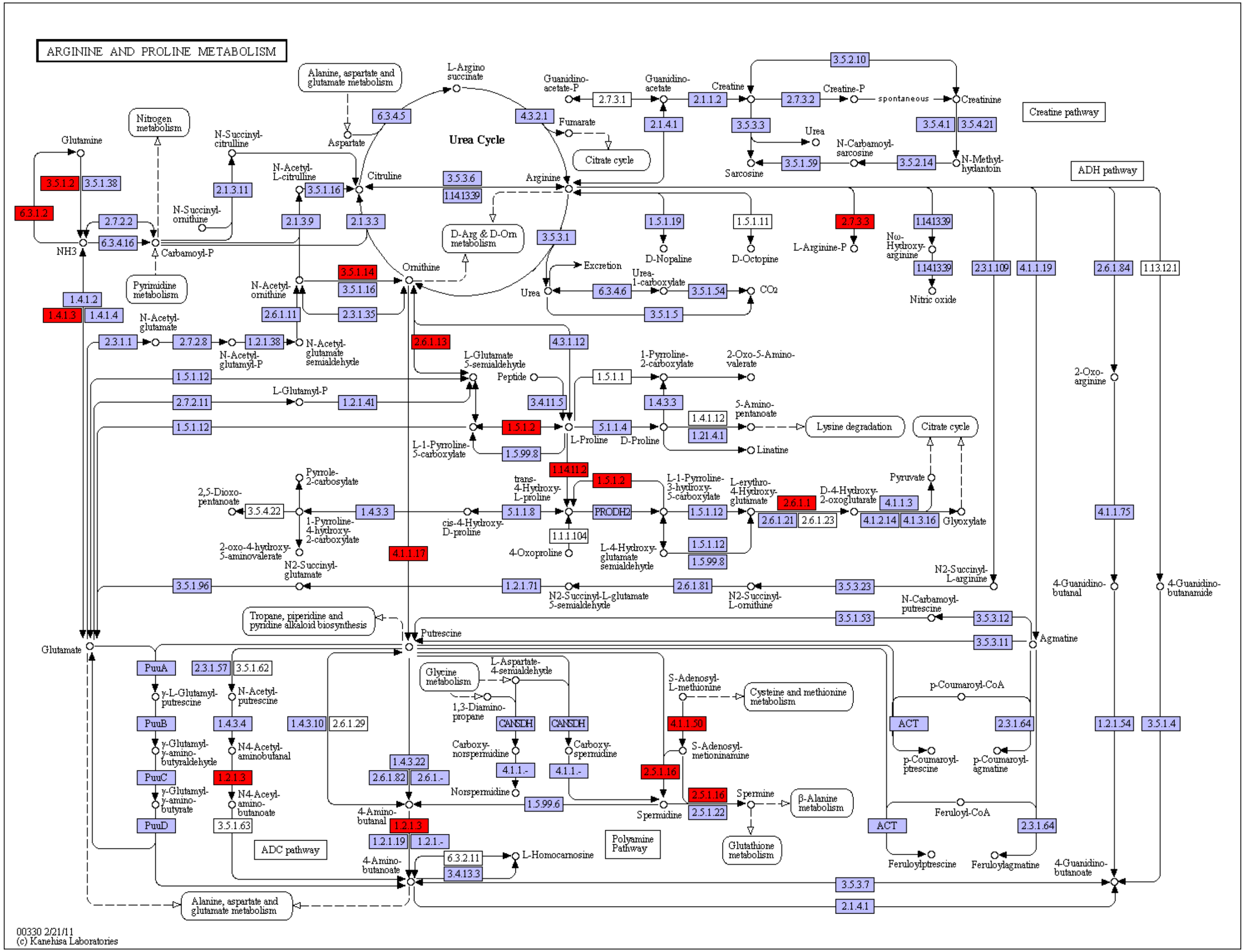

Figure 112: ko00330 Arginine and proline metabolism

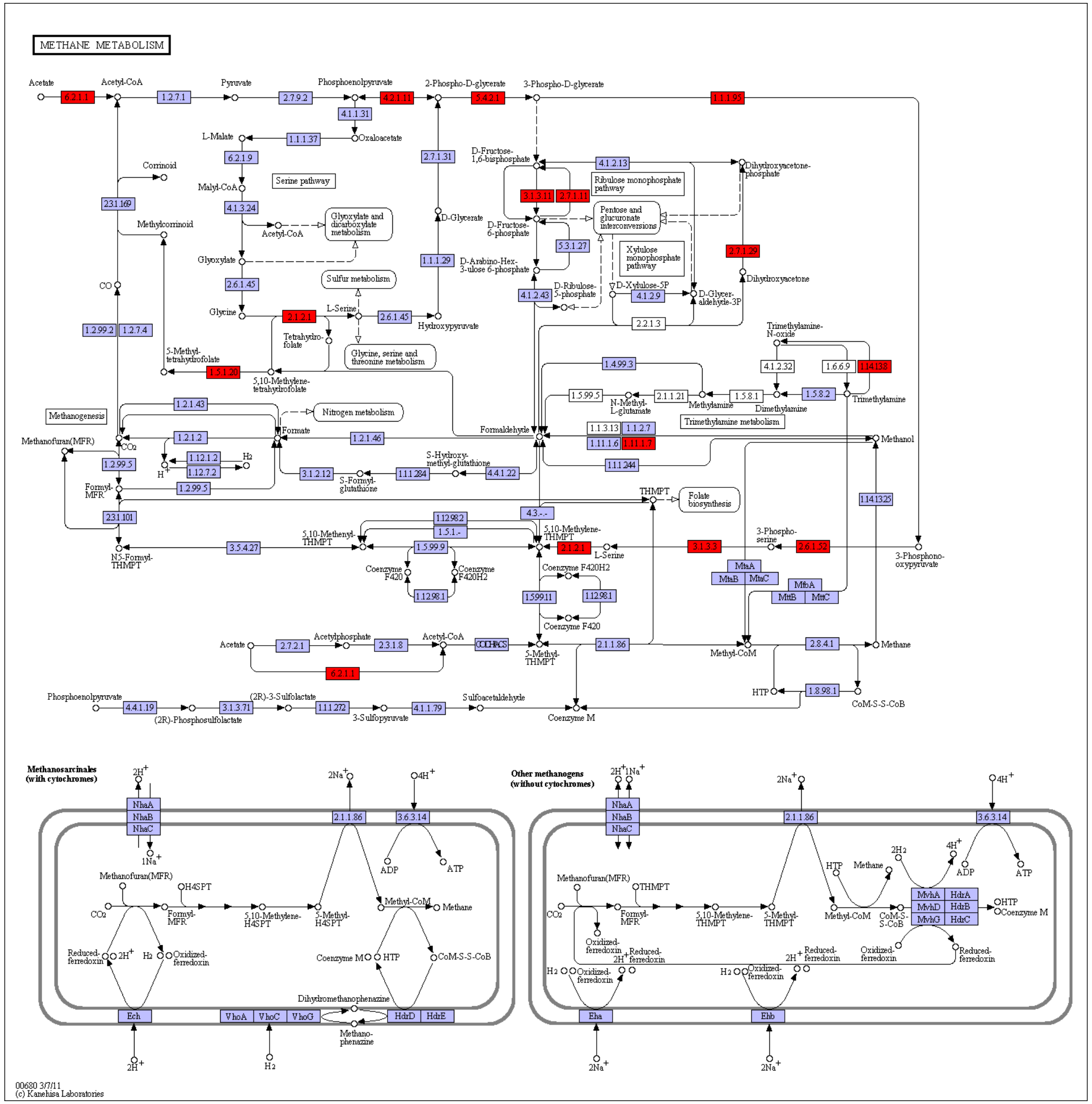

Figure 113: ko00680 Methane metabolism

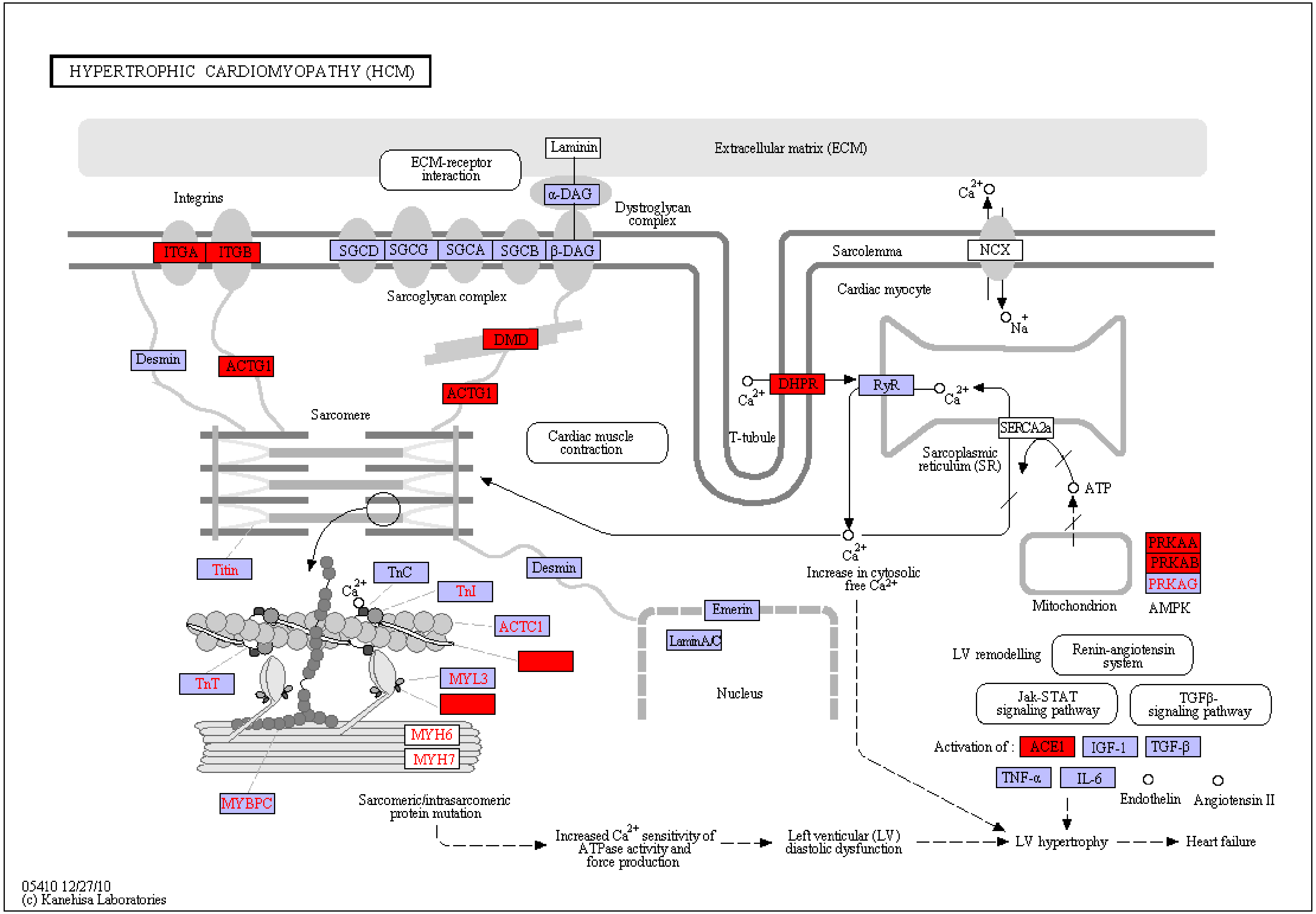

Figure 114: ko05410 Hypertrophic cardiomyopathy (HCM)

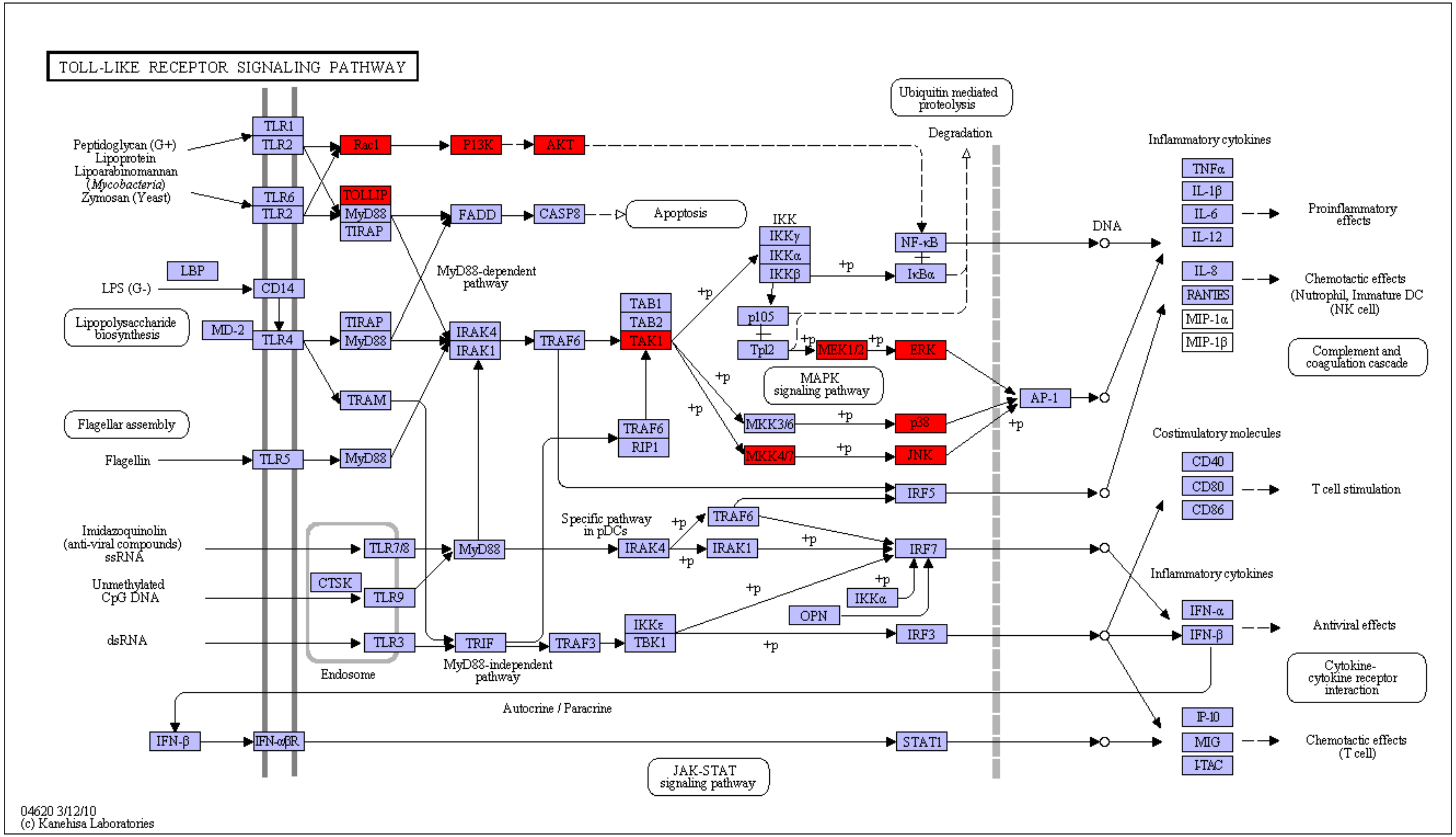

Figure 115: ko04620 Toll-like receptor signaling pathway

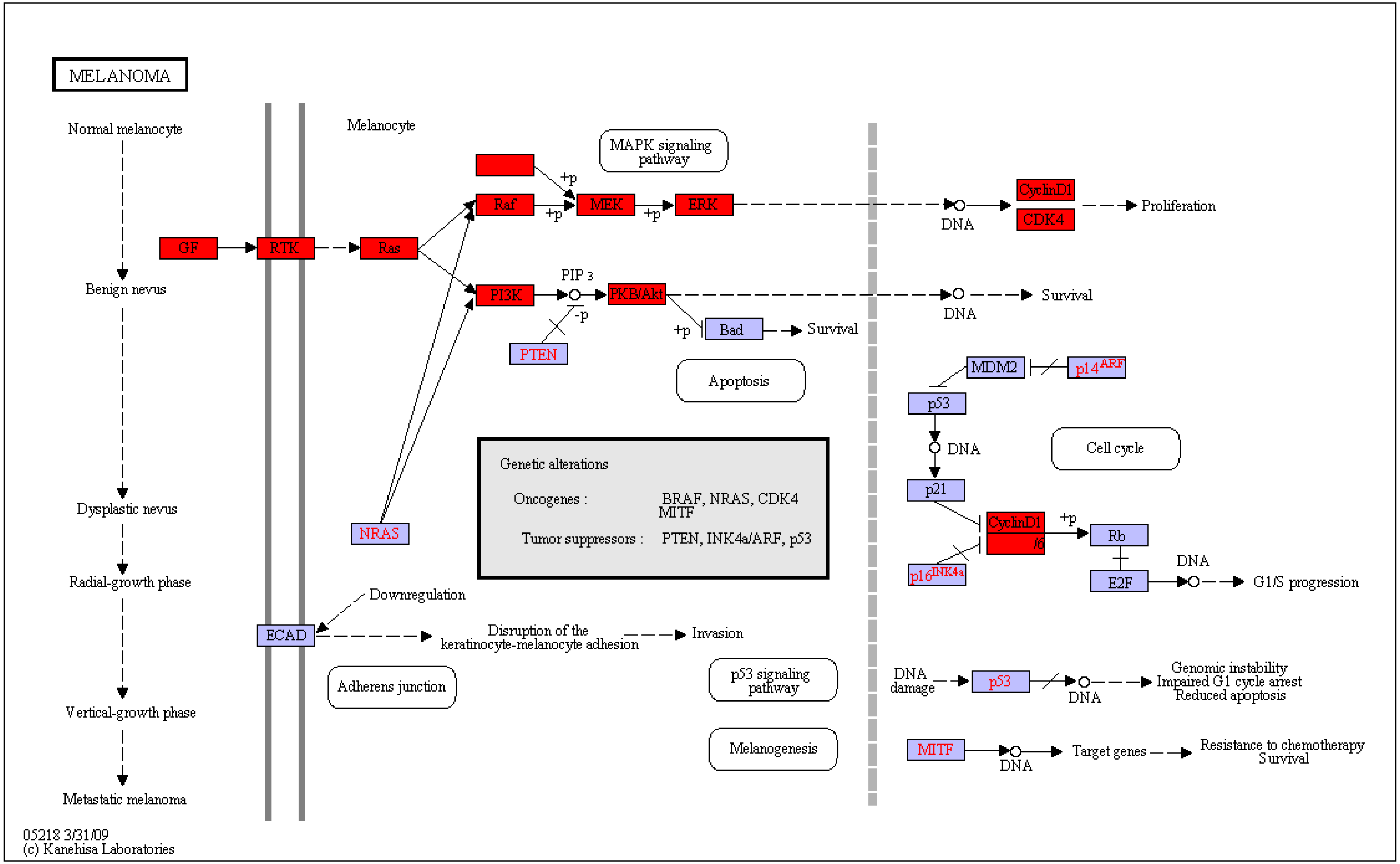

Figure 116: ko05218 Melanoma

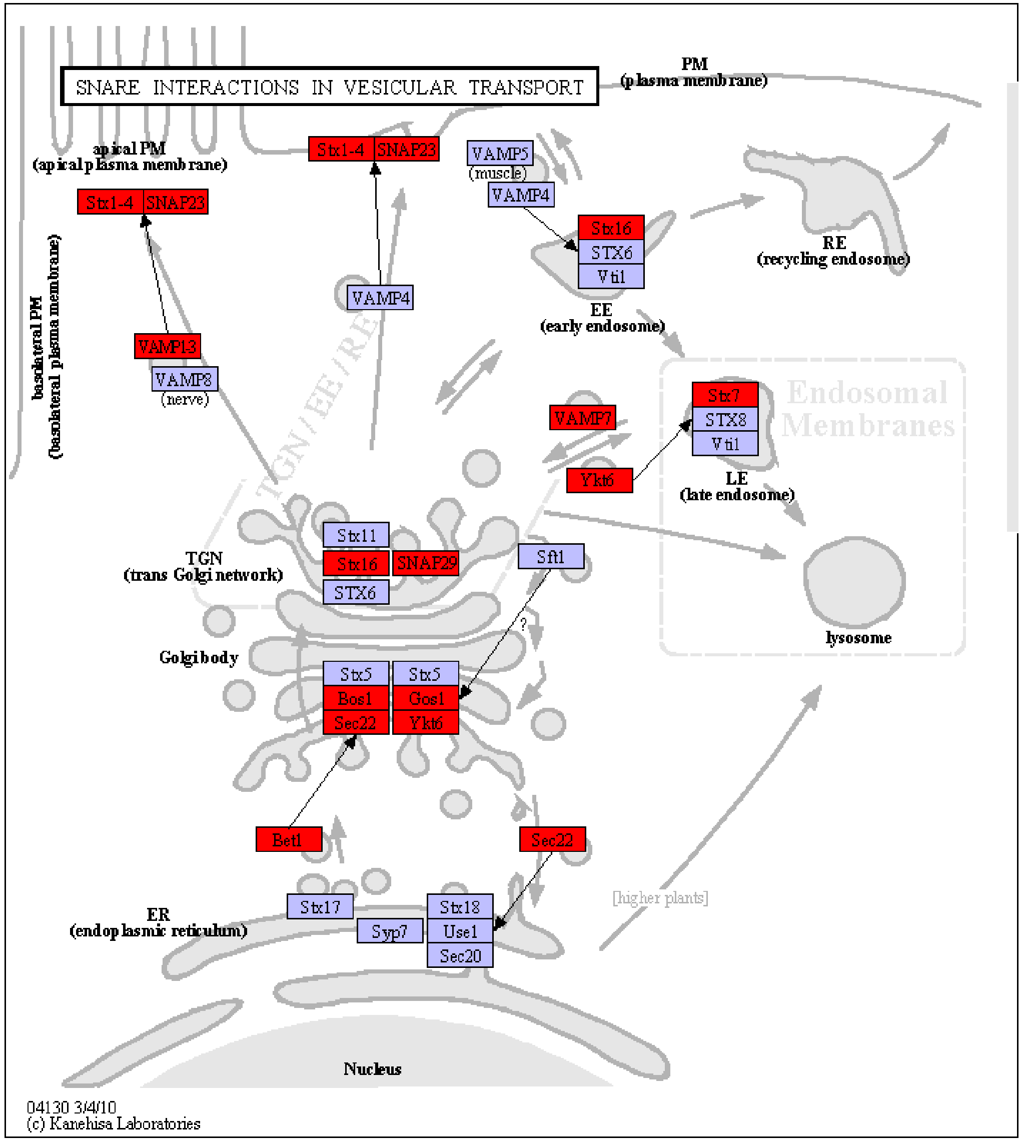

Figure 117: ko04130 SNARE interactions in vesicular transport

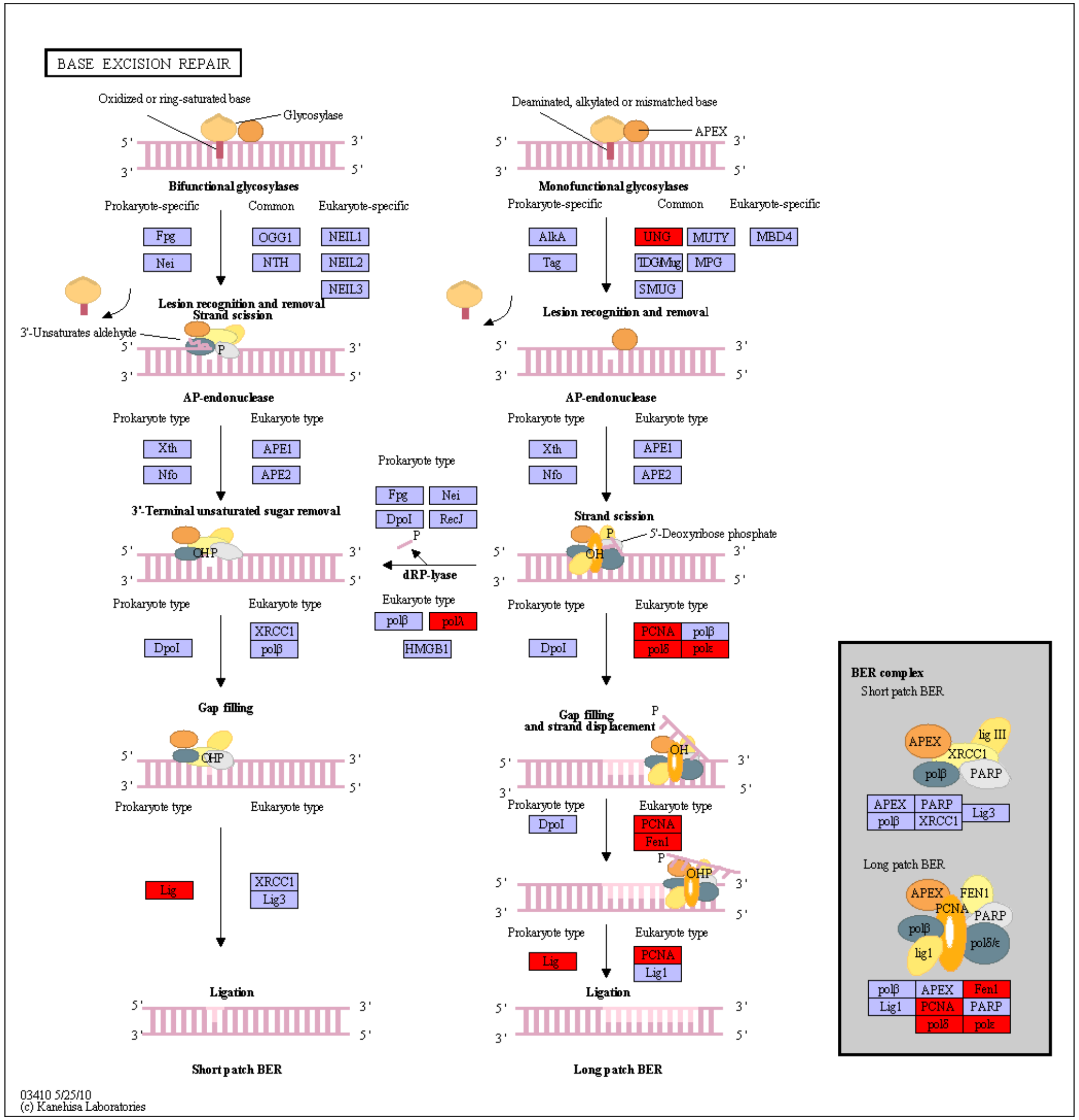

Figure 118: ko03410 Base excision repair

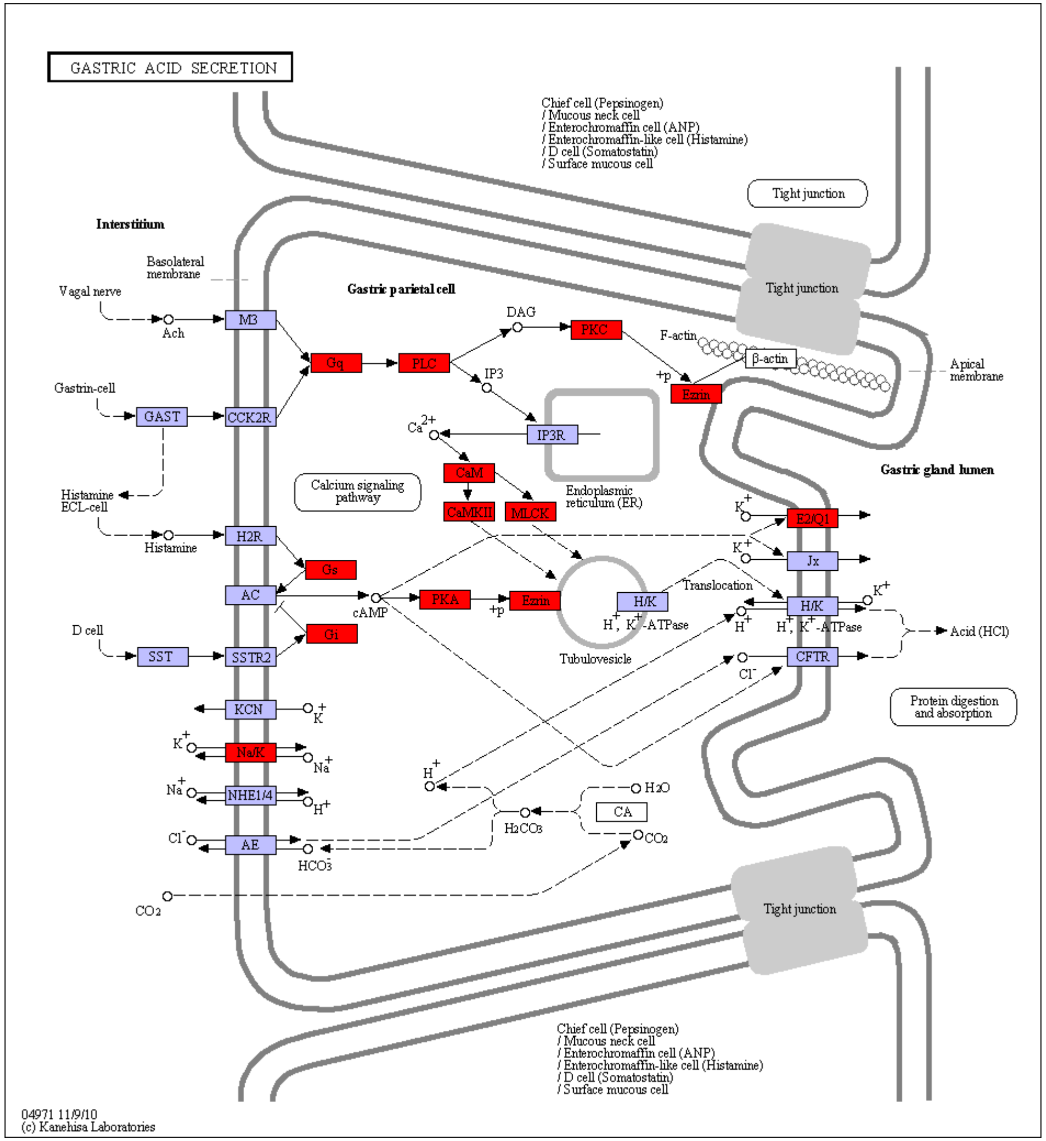

Figure 119: ko04971 Gastric acid secretion

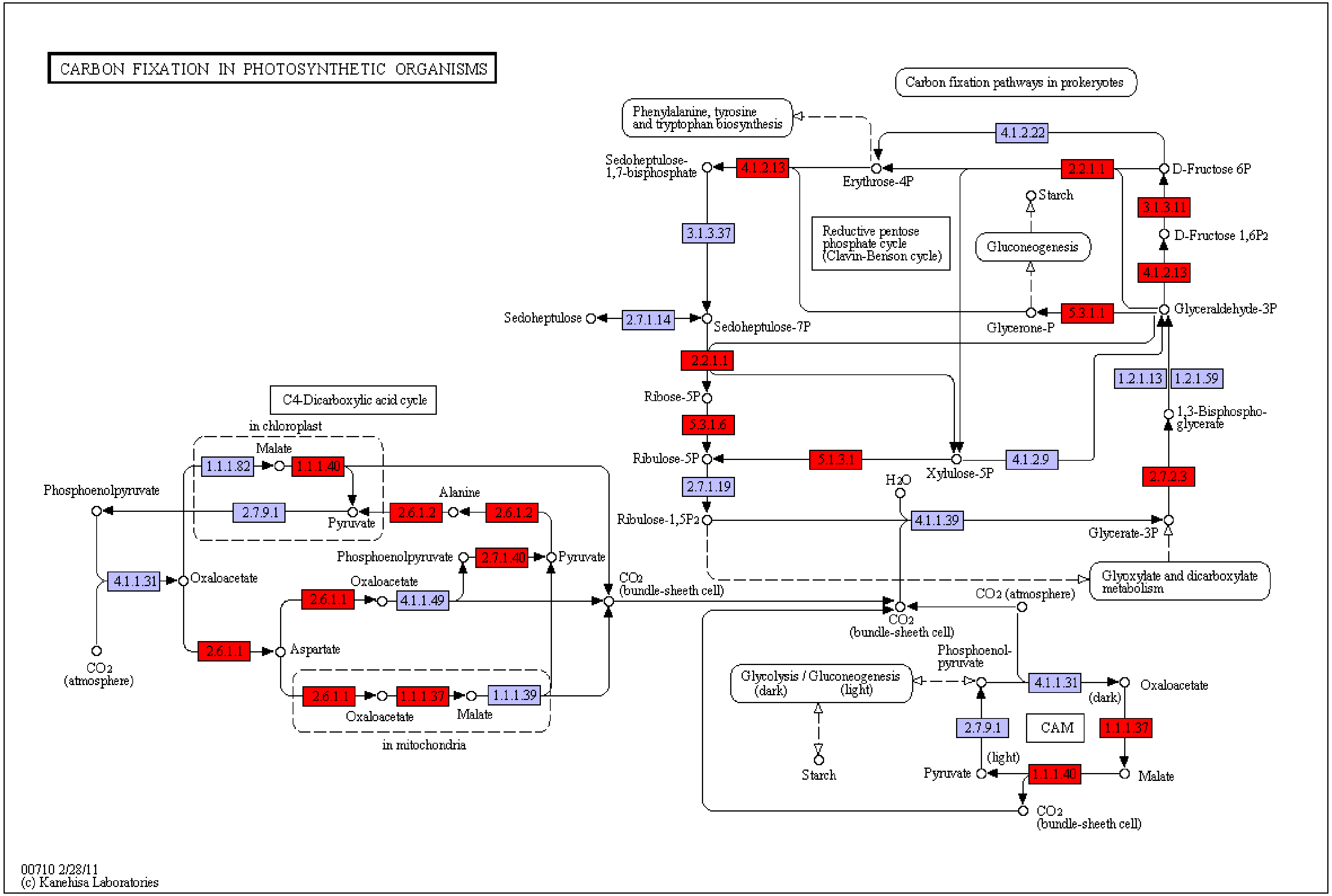

Figure 120: ko00710 Carbon fixation in photosynthetic organisms

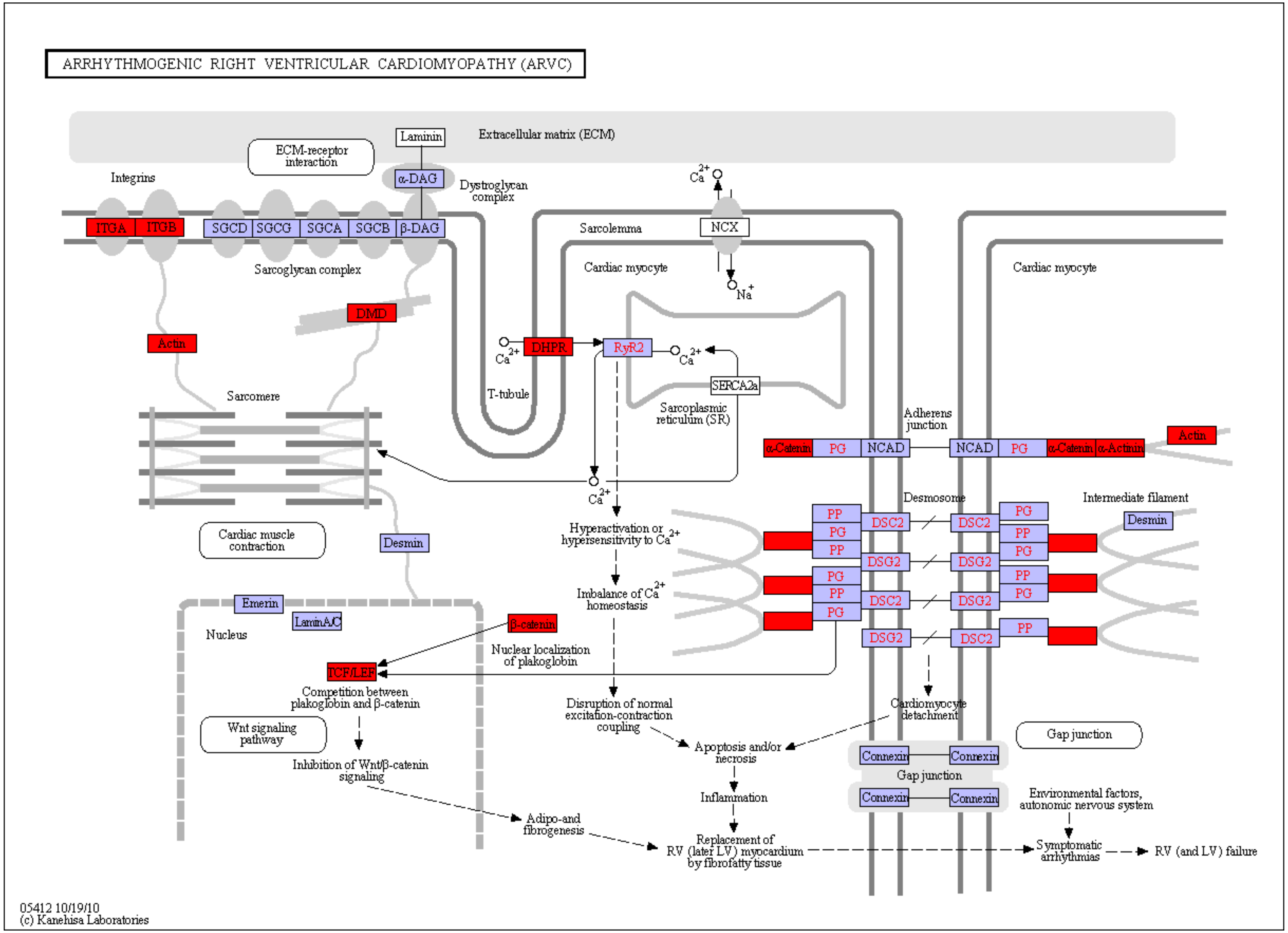

Figure 121: ko05412 Arrhythmogenic right ventricular cardiomyopathy (ARVC)

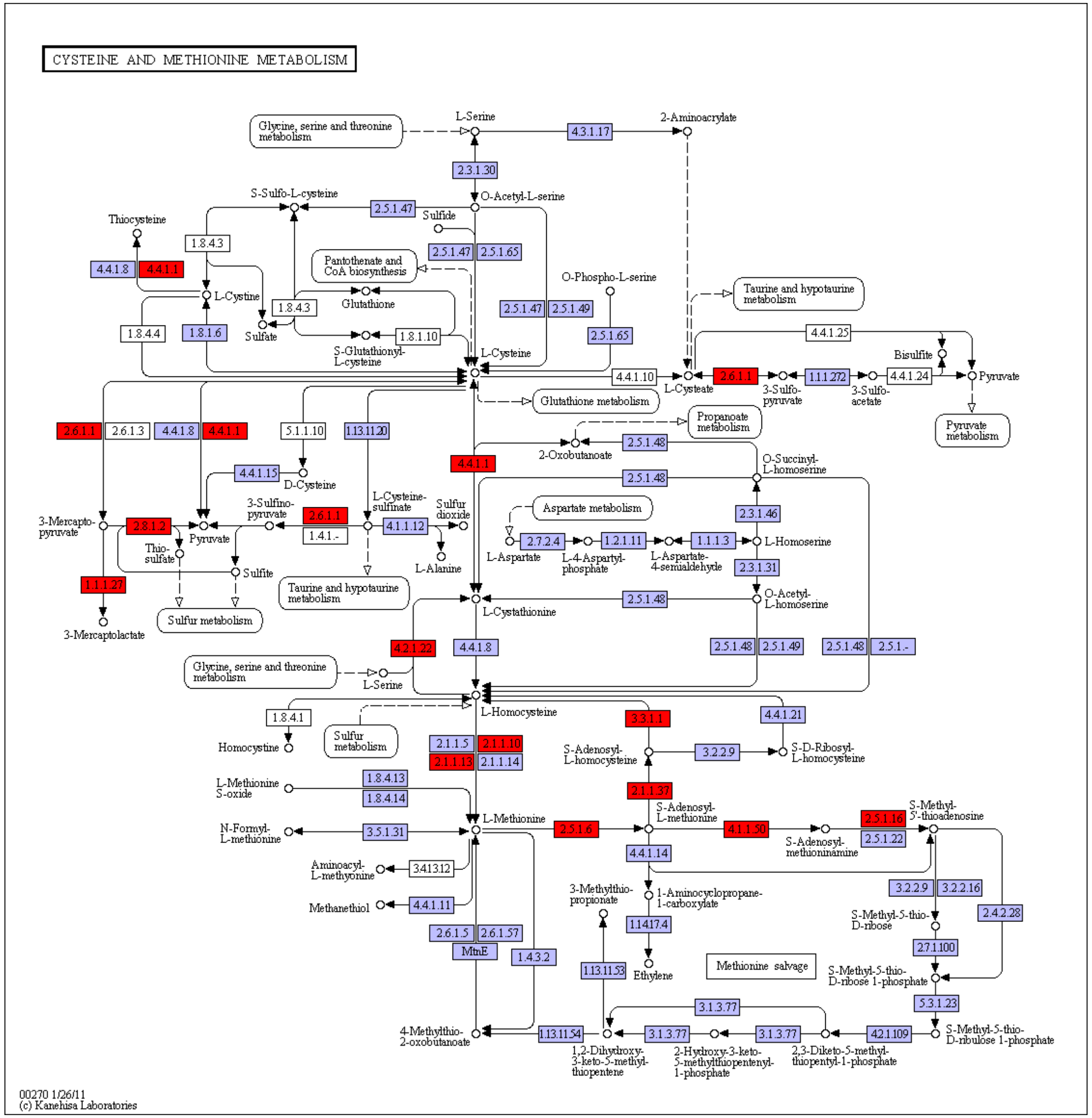

Figure 122: ko00270 Cysteine and methionine metabolism

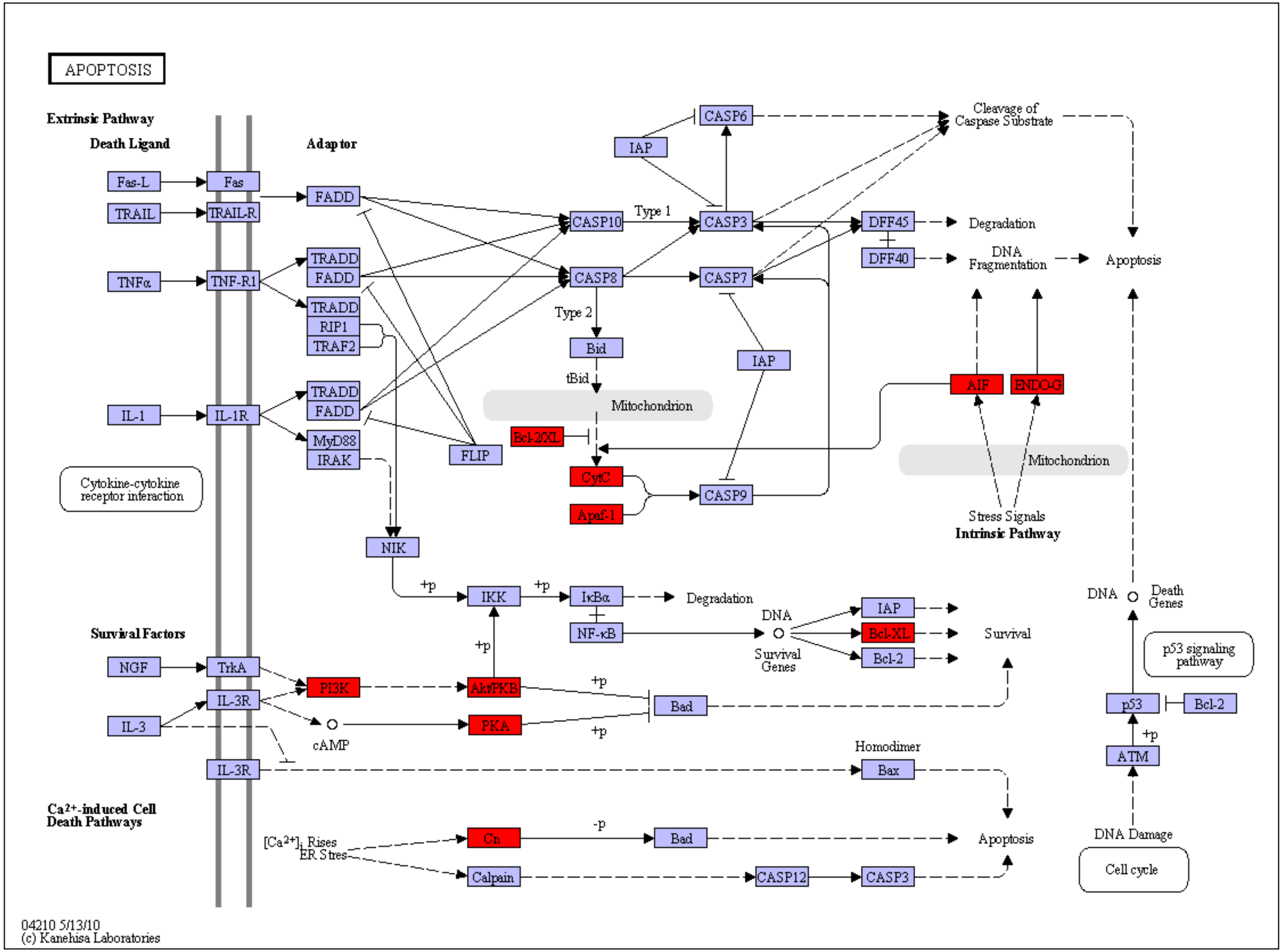

Figure 123: ko04210 Apoptosis

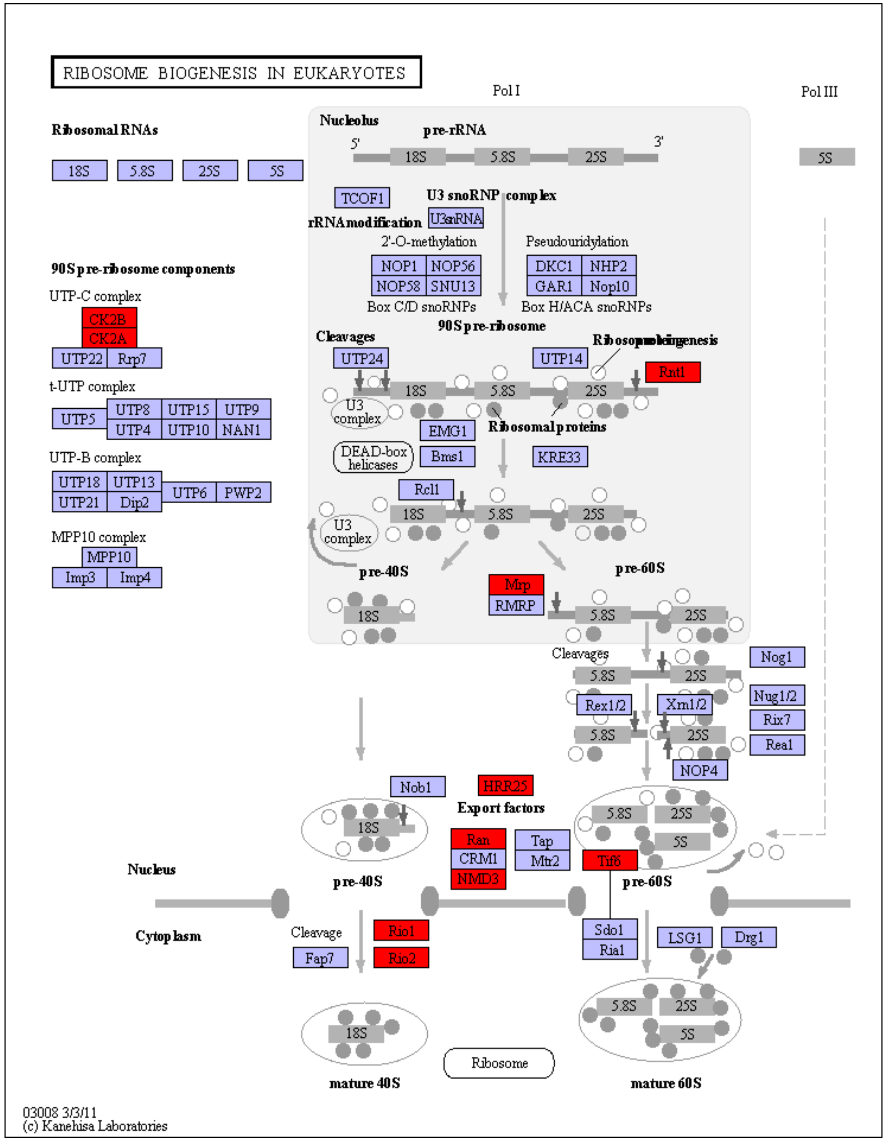

Figure 124: ko03008 Ribosome biogenesis in eukaryotes

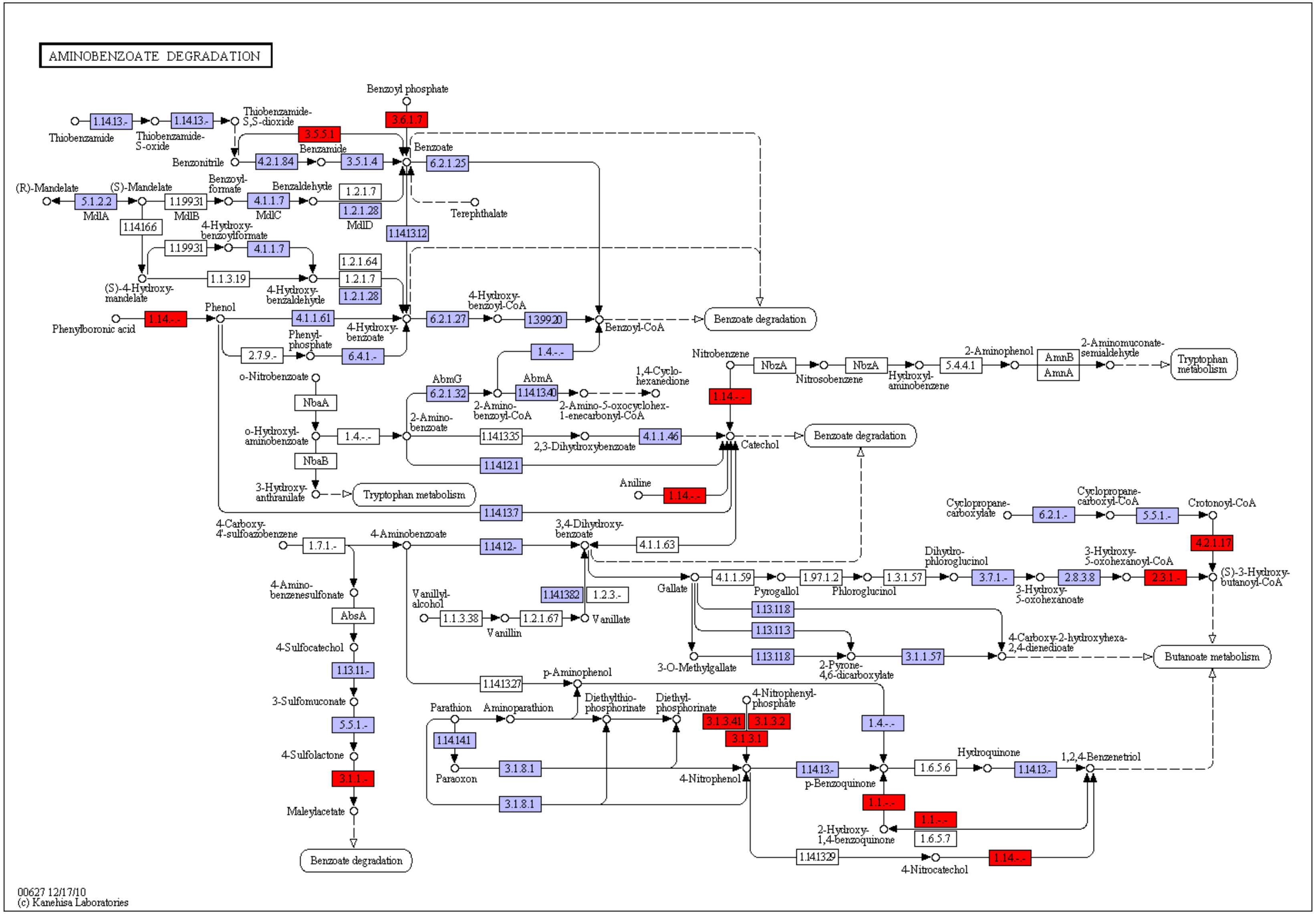

Figure 125: ko00627 Aminobenzoate degradation

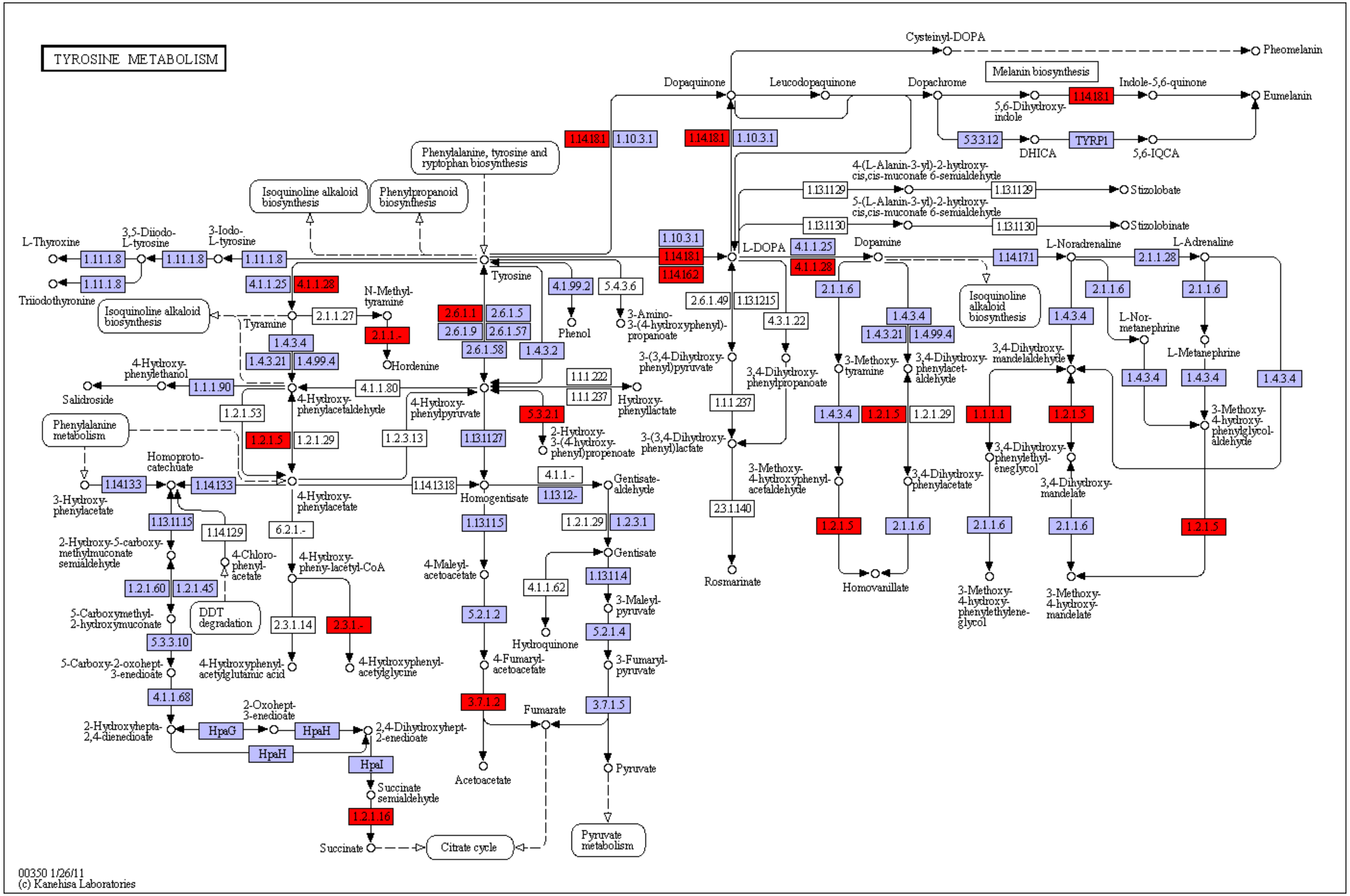

Figure 126: ko00350 Tyrosine metabolism

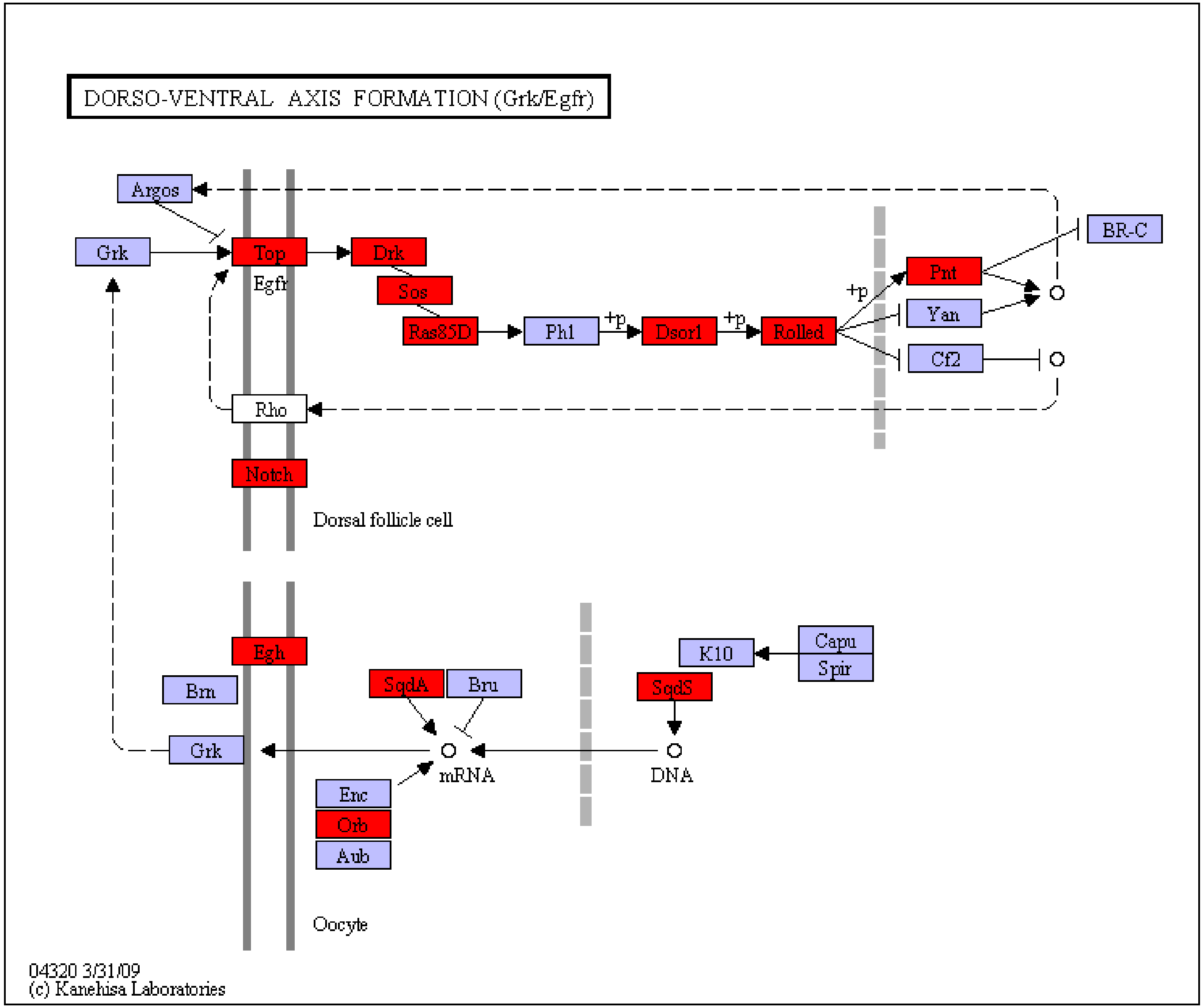

Figure 127: ko04320 Dorso-ventral axis formation

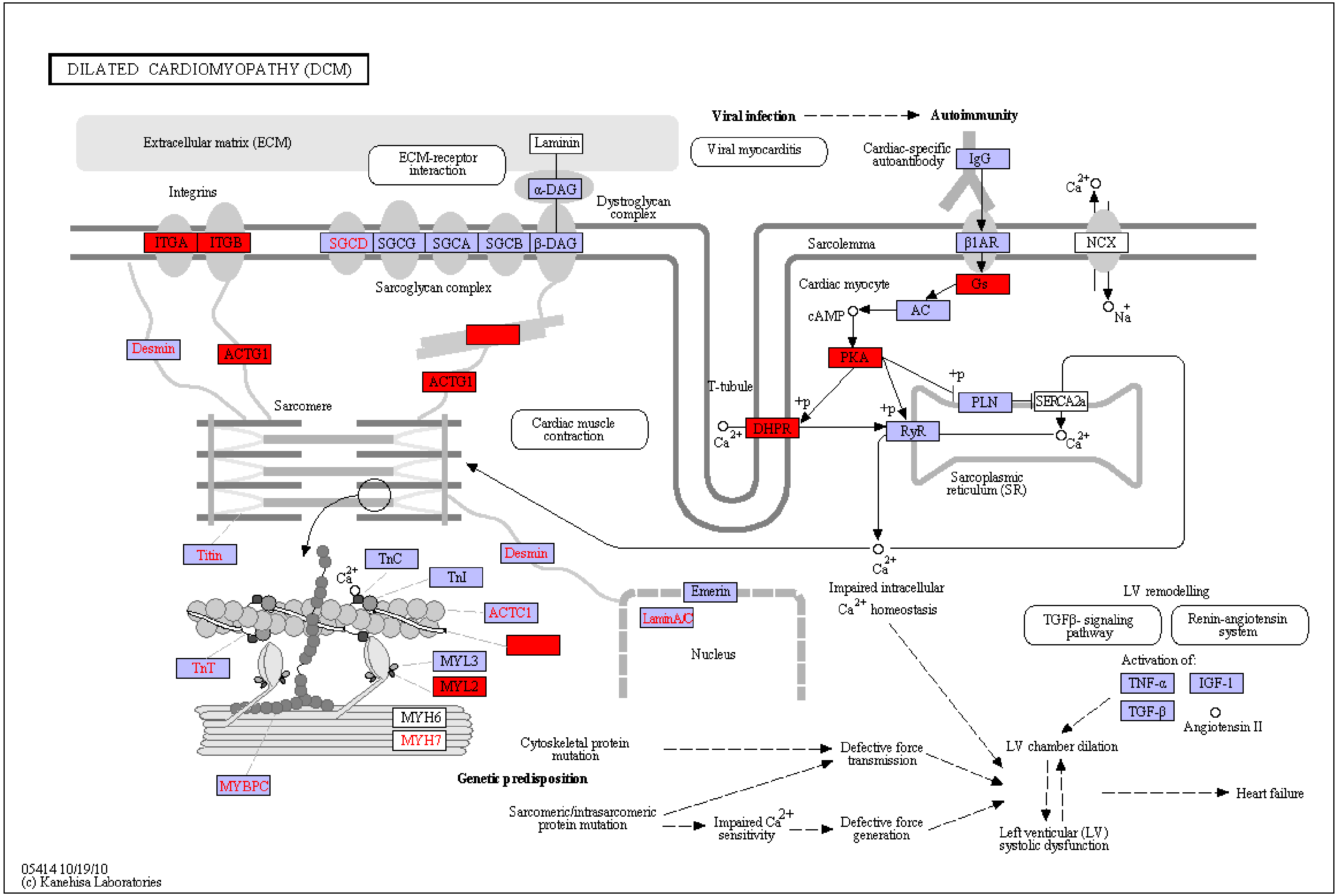

Figure 128: ko05414 Dilated cardiomyopathy

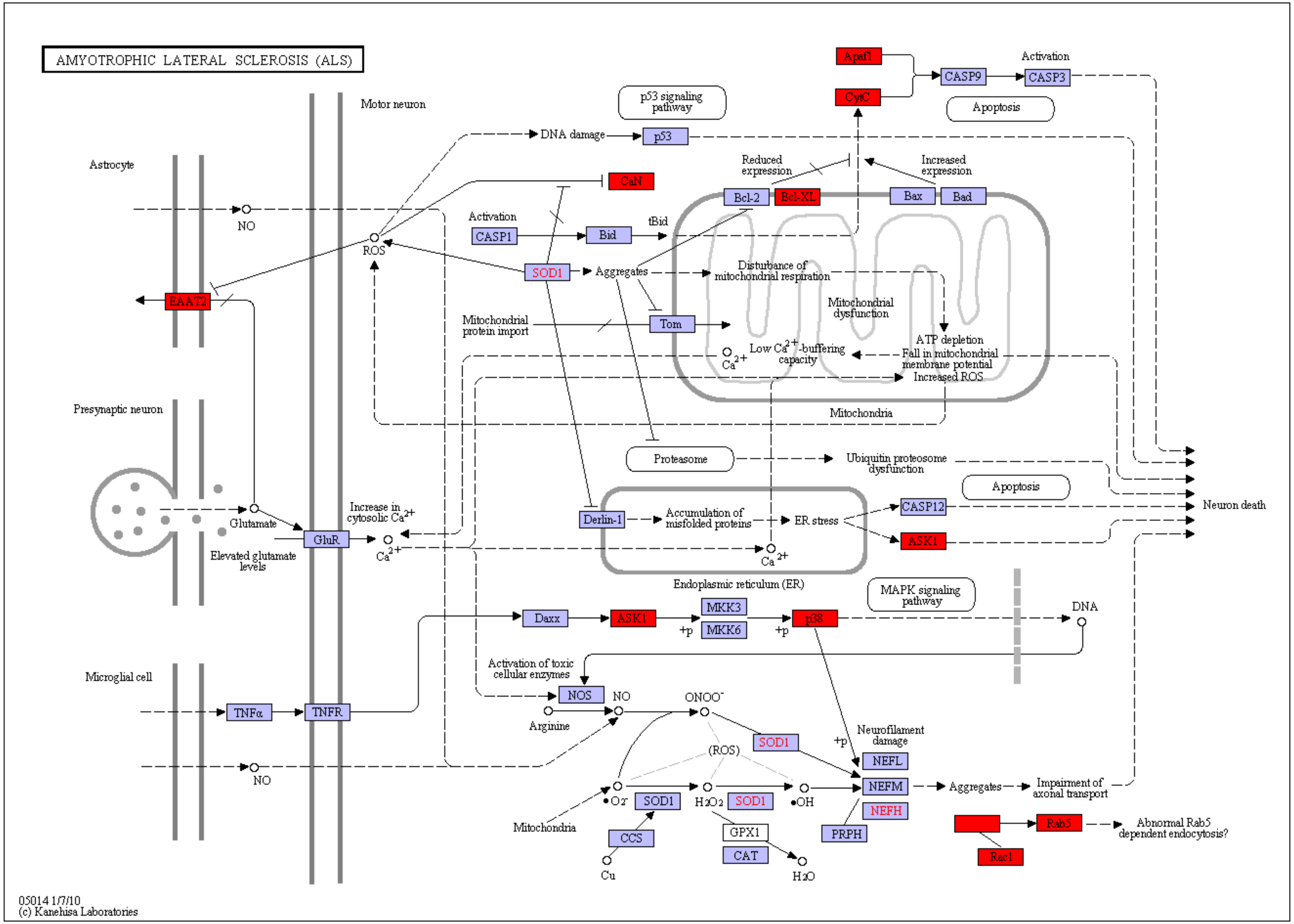

Figure 129: ko05014 Amyotrophic lateral sclerosis (ALS)

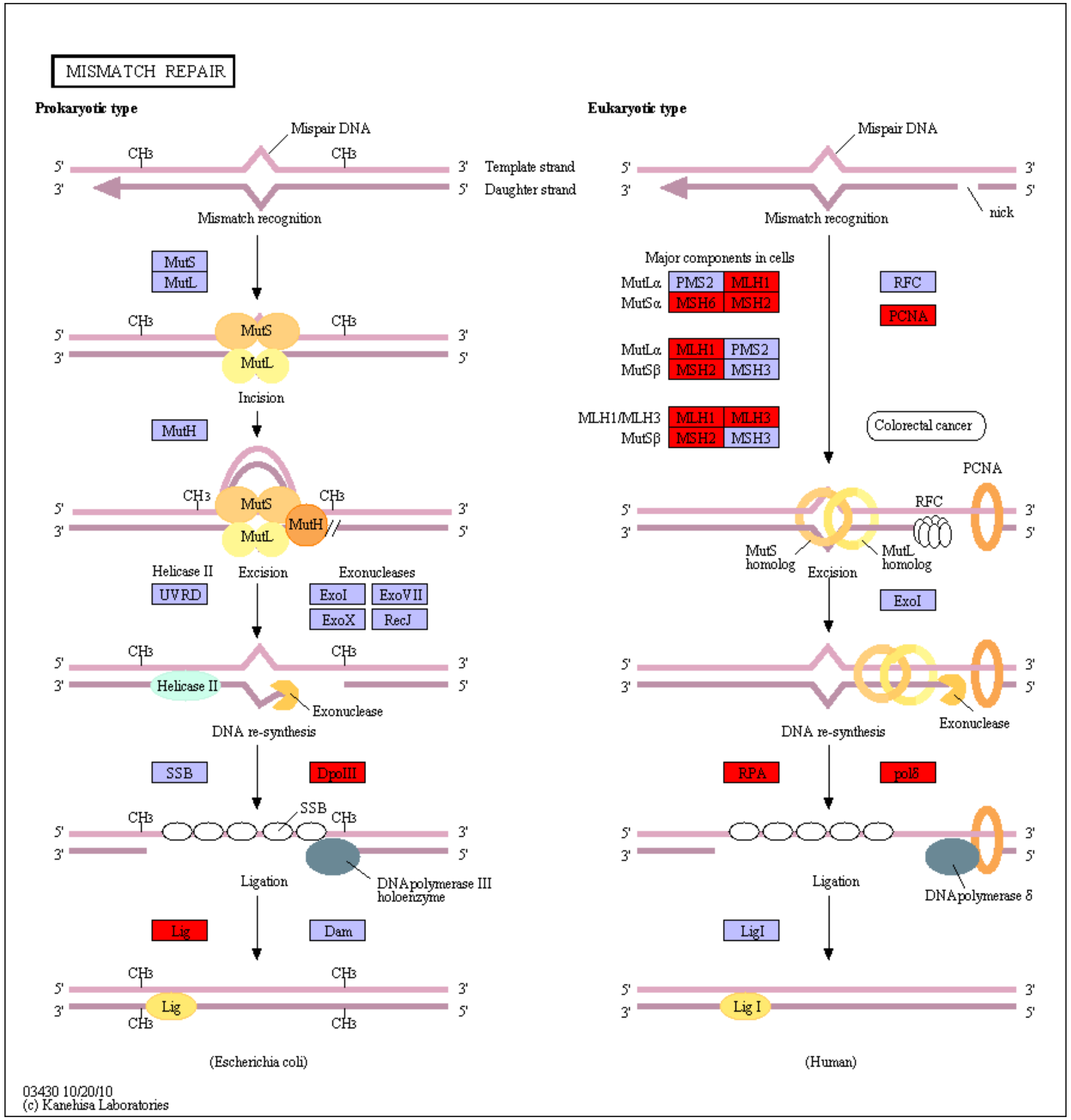

Figure 130: ko03430 Mismatch repair

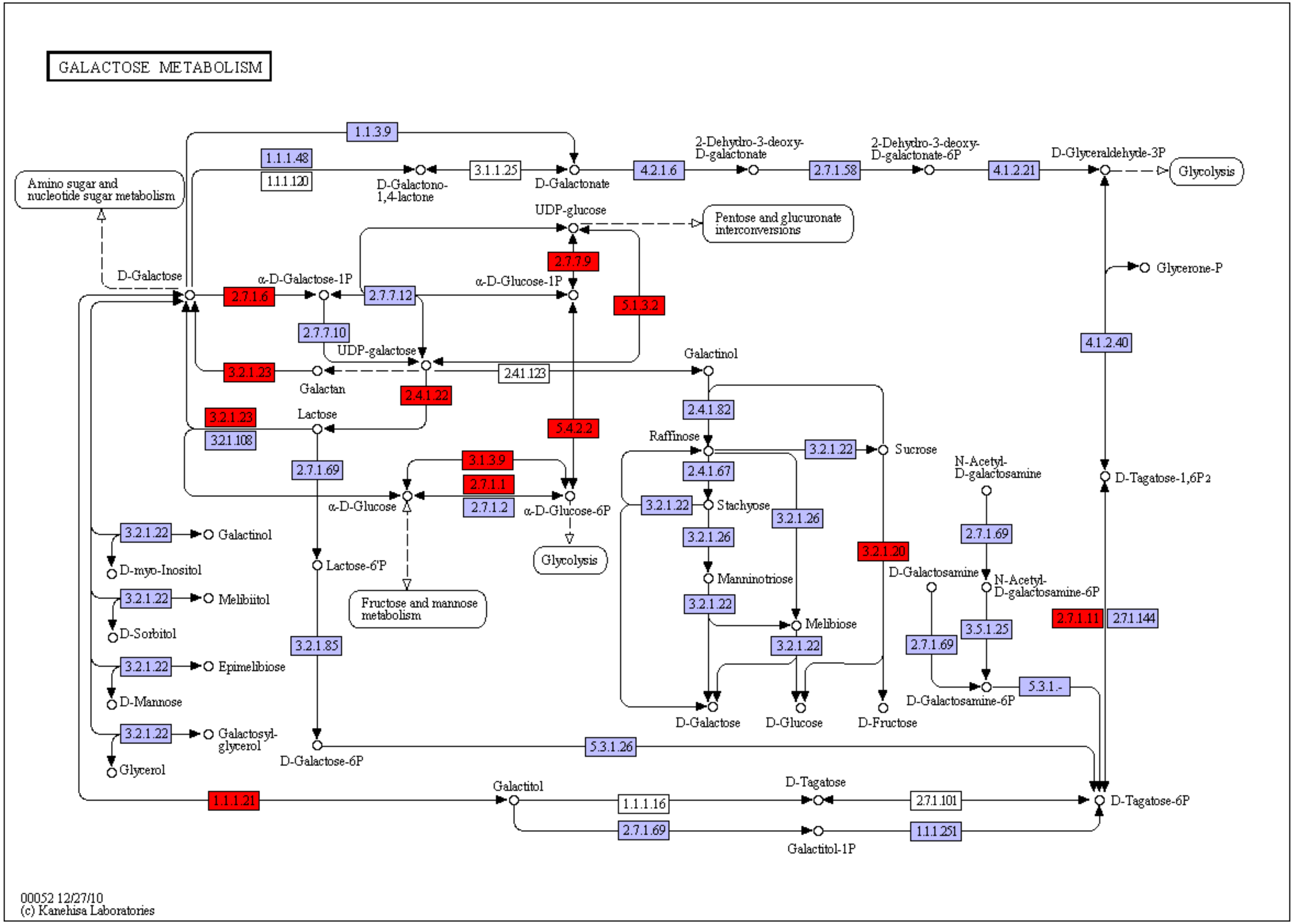

Figure 131: ko00052 Galactose metabolism

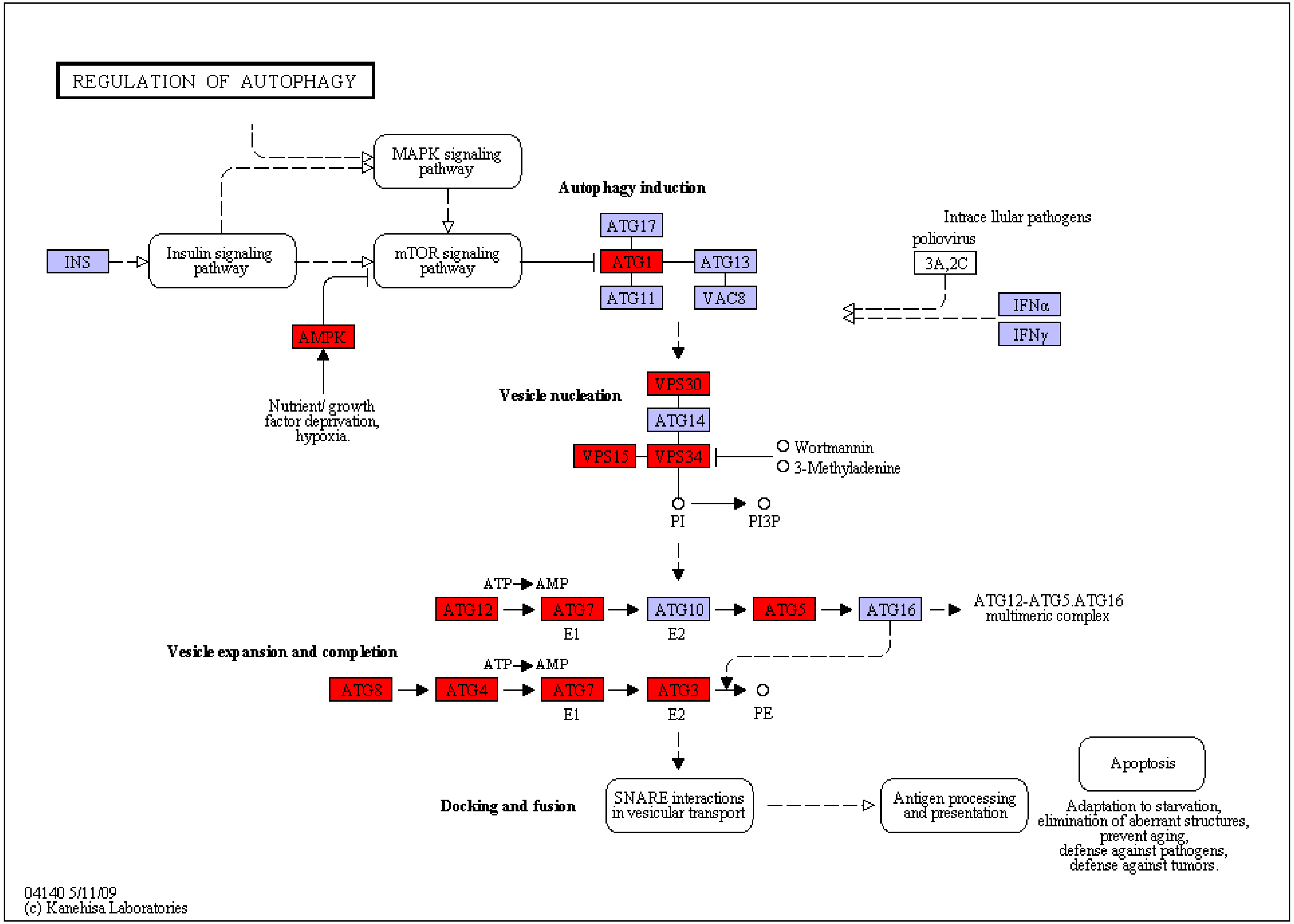

Figure 132: ko04140 Regulation of autophagy

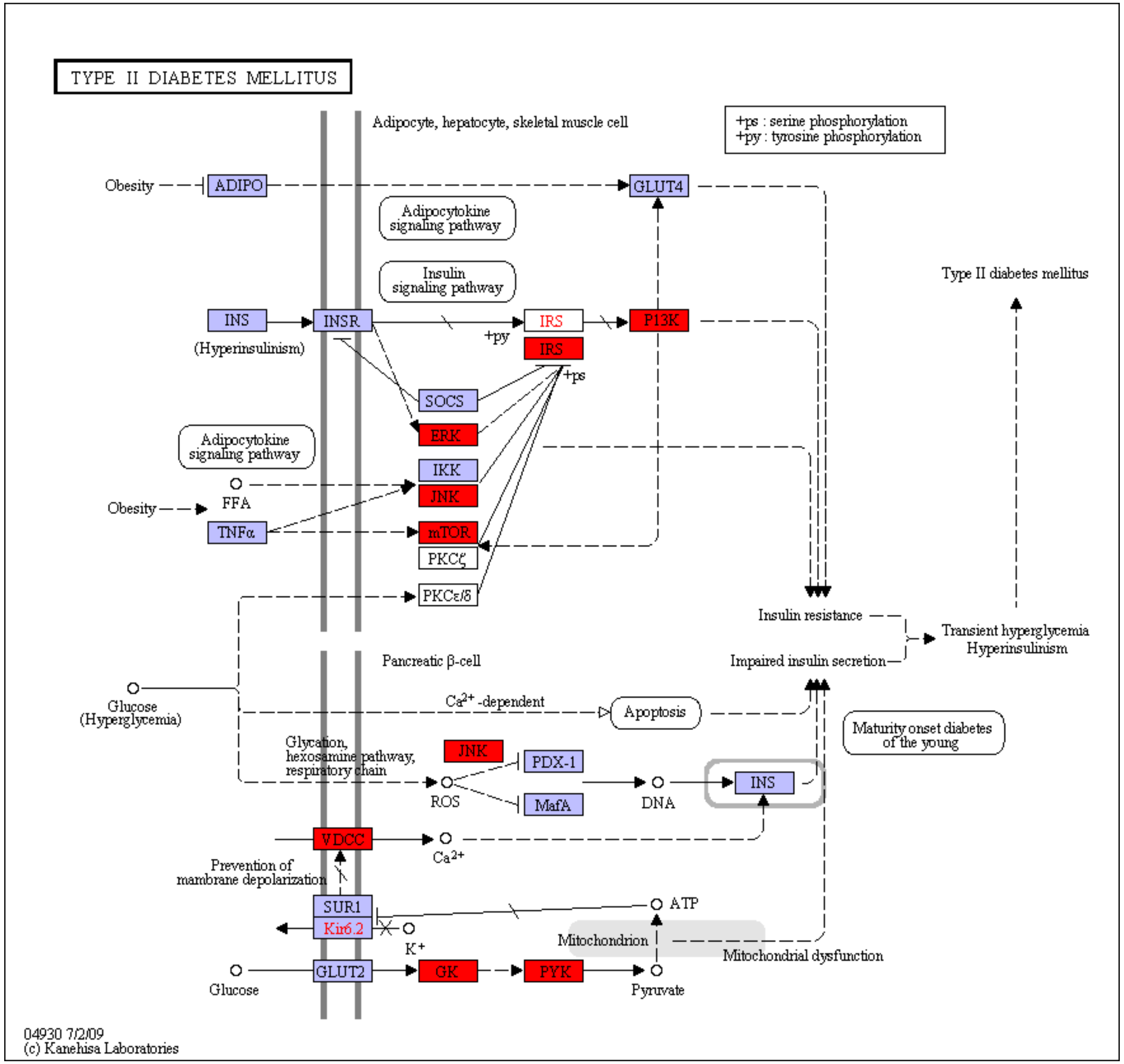

Figure 133: ko04930 Type II diabetes mellitus

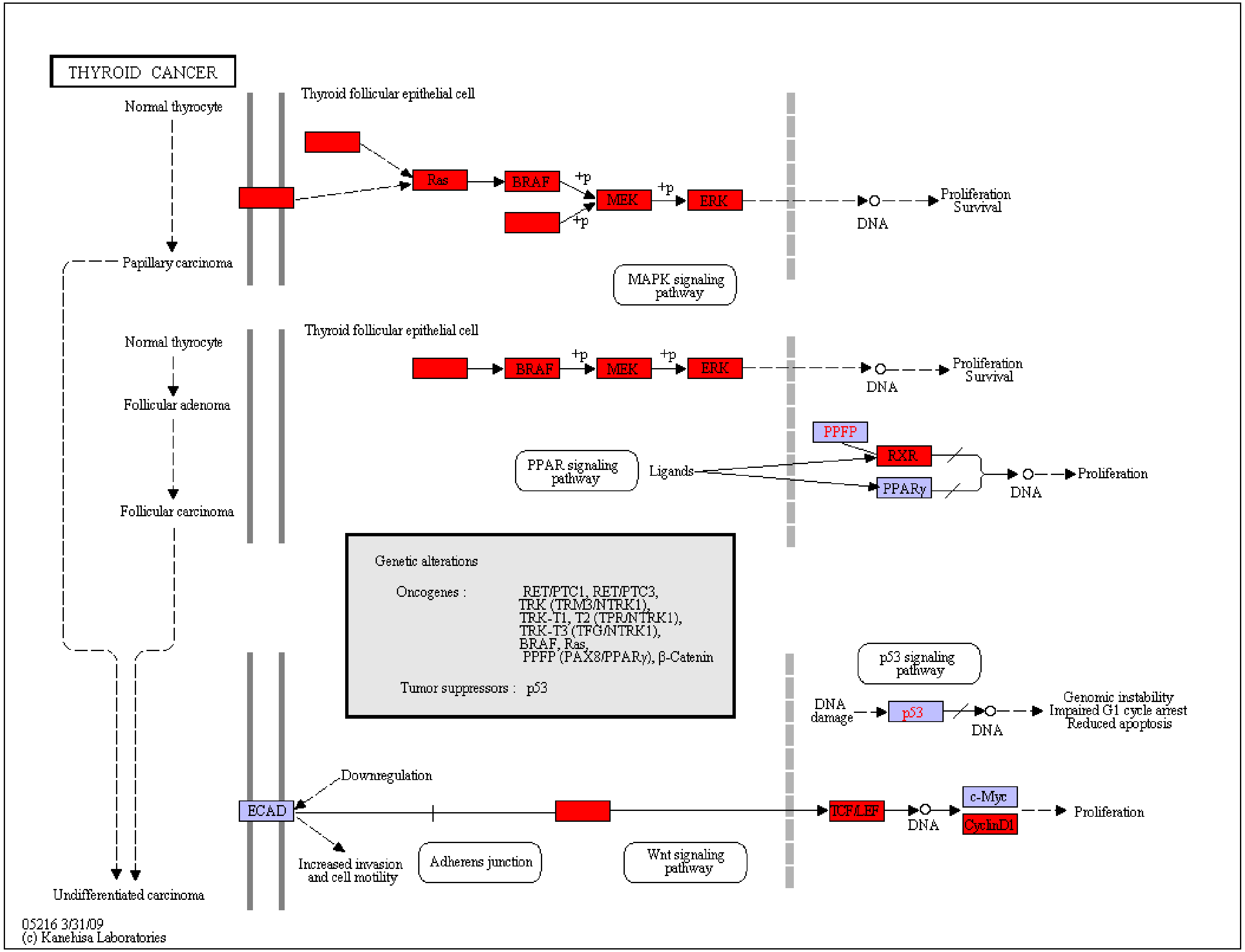

Figure 134: ko05216 Thyroid cancer

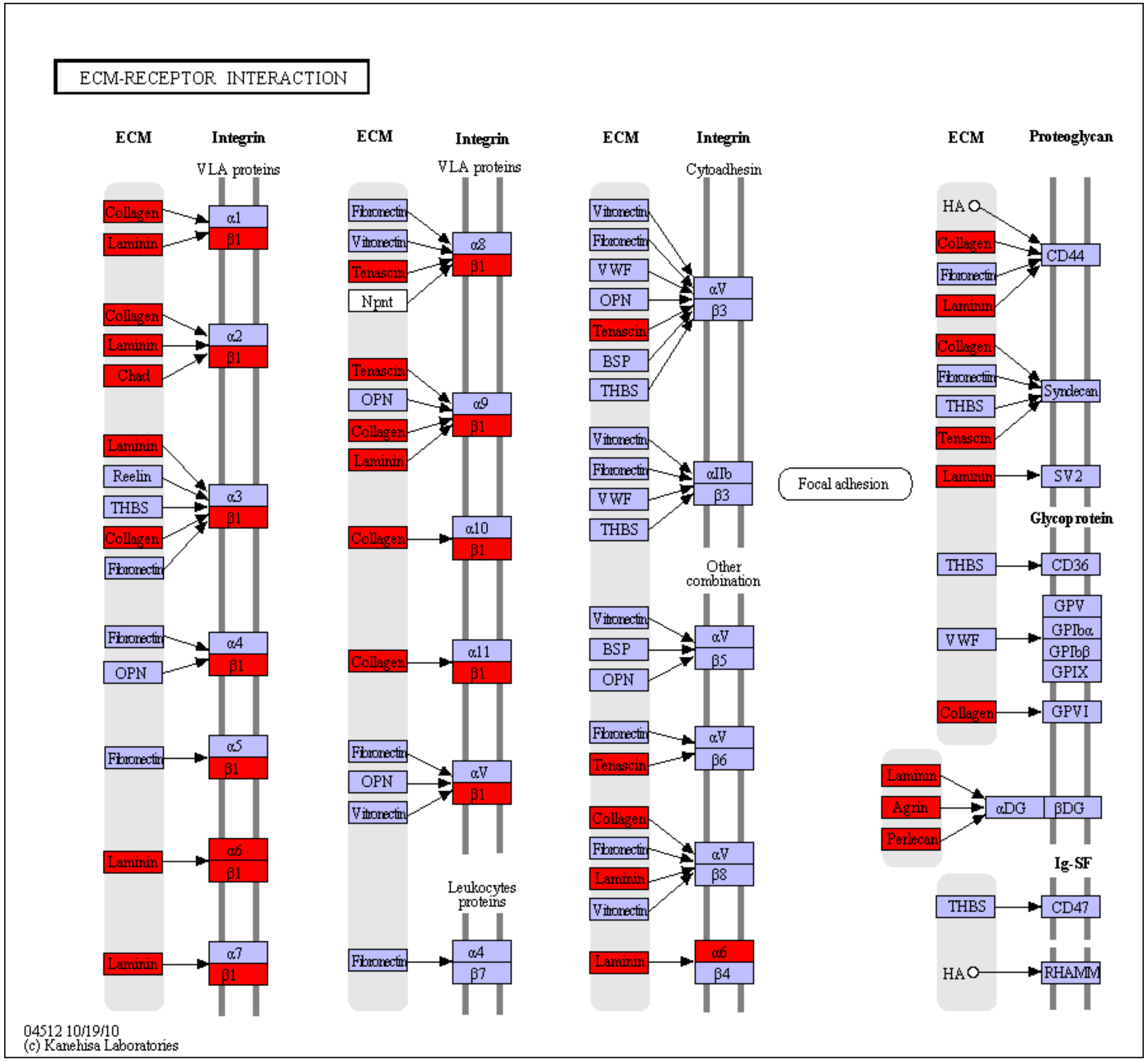

Figure 135: ko04512 ECM-receptor interaction

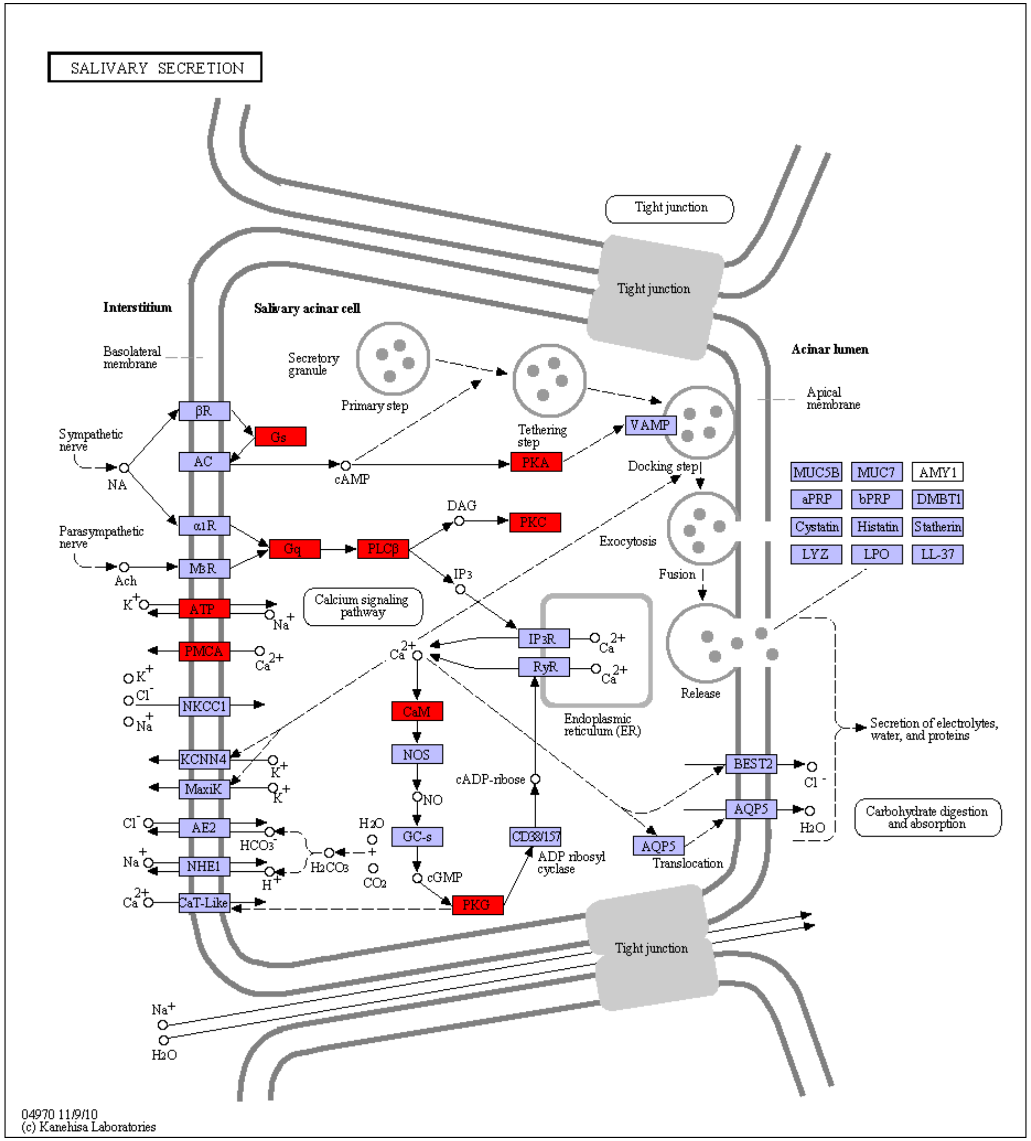

Figure 136: ko04970 Salivary secretion

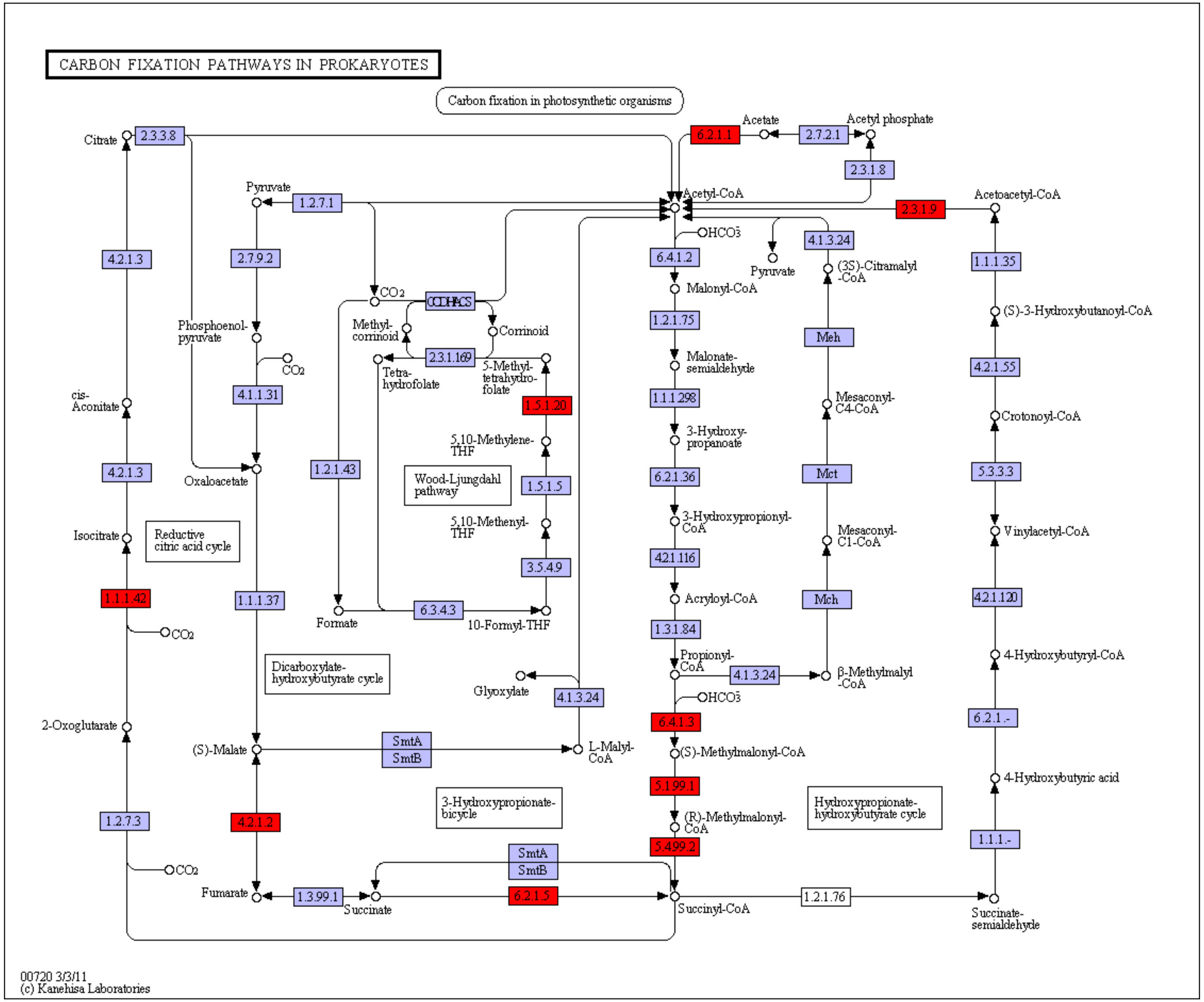

Figure 137: ko00720 Carbon fixation pathways in prokaryotes

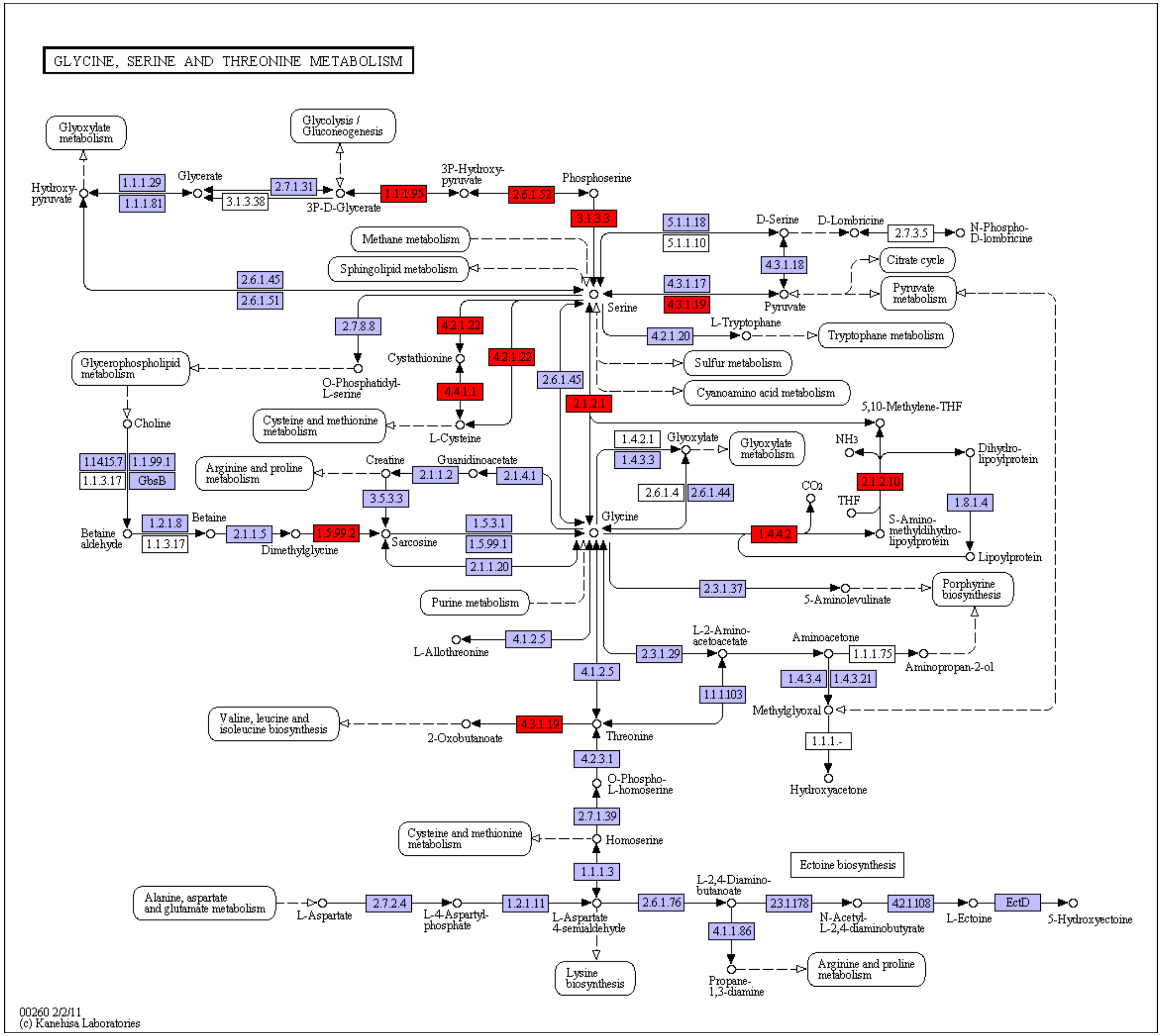

Figure 138: ko00260 Glycine, serine and threonine metabolism

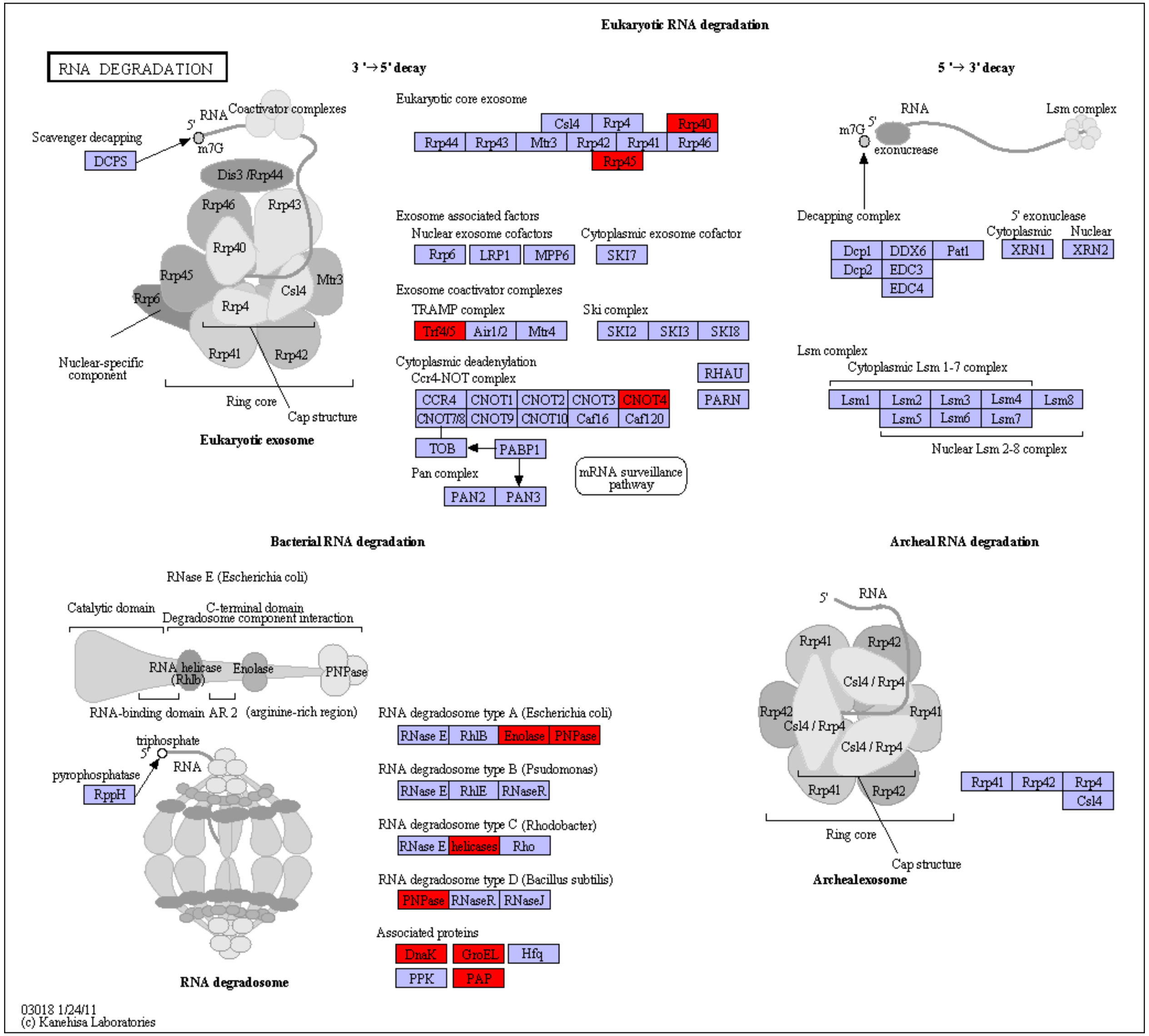

Figure 139: ko03018 RNA degradation

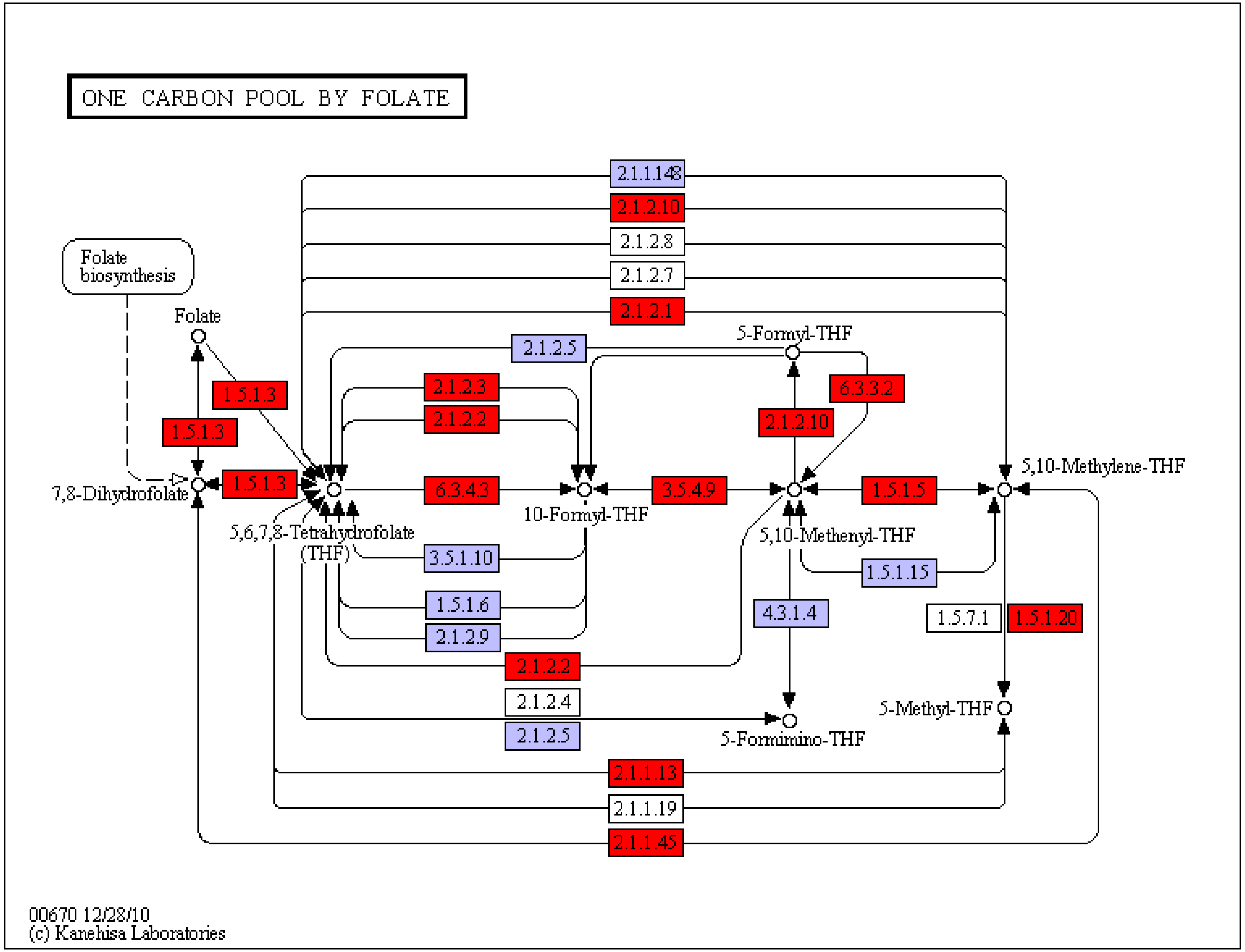

Figure 140: ko00670 One carbon pool by folate

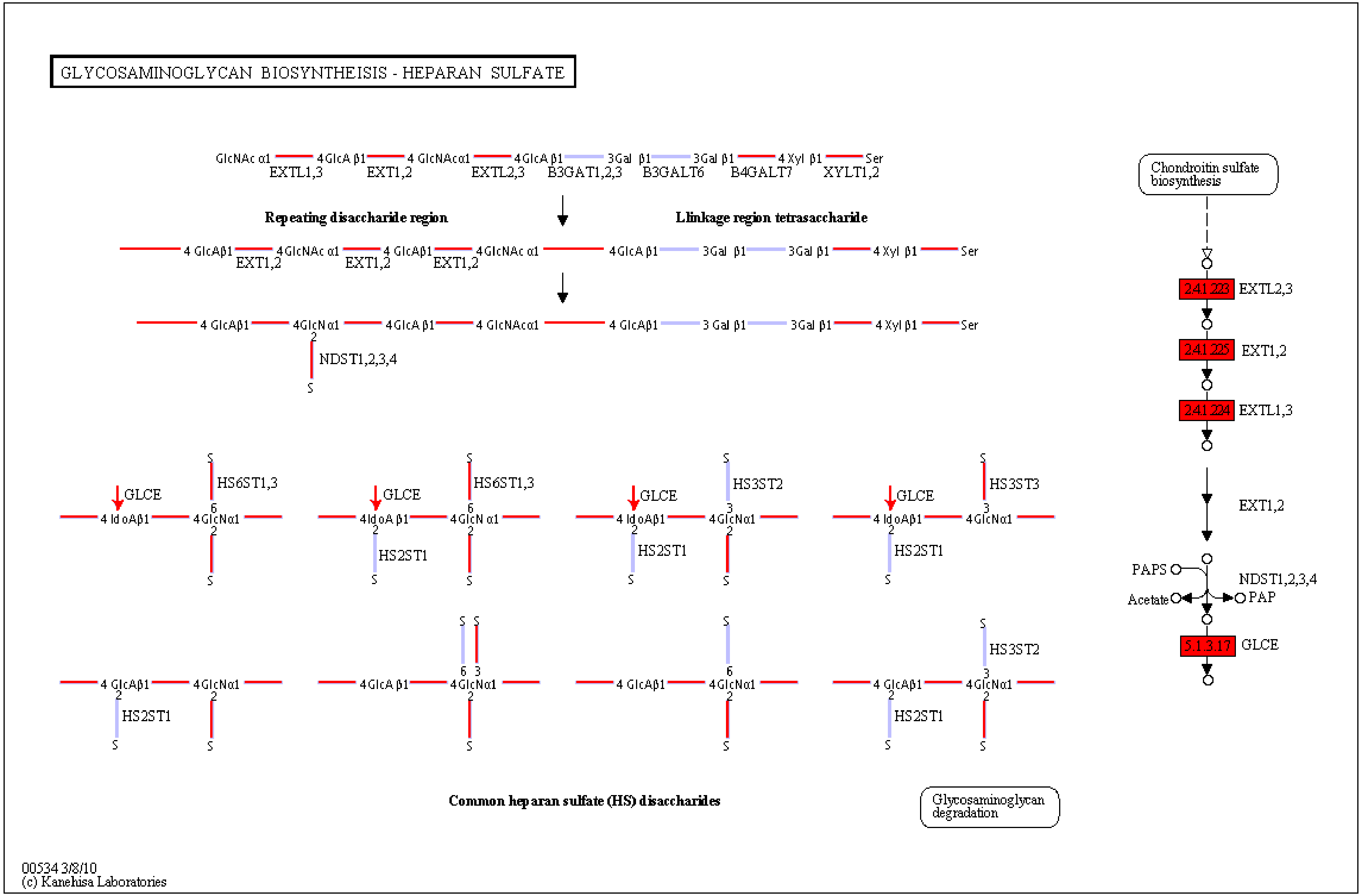

Figure 141: ko00534 Glycosaminoglycan biosynthesis - heparan sulfate

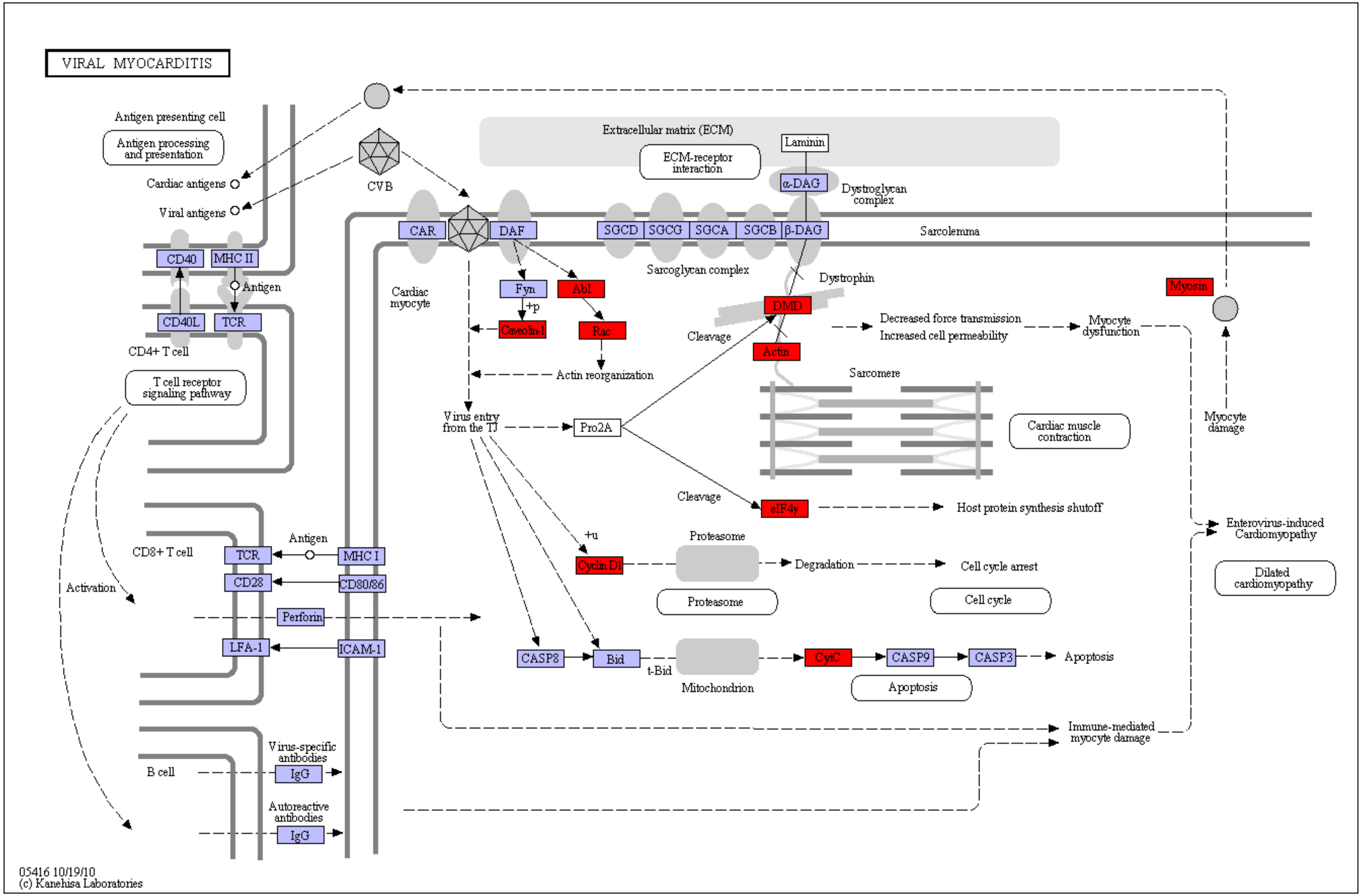

Figure 142: ko05416 Viral myocarditis

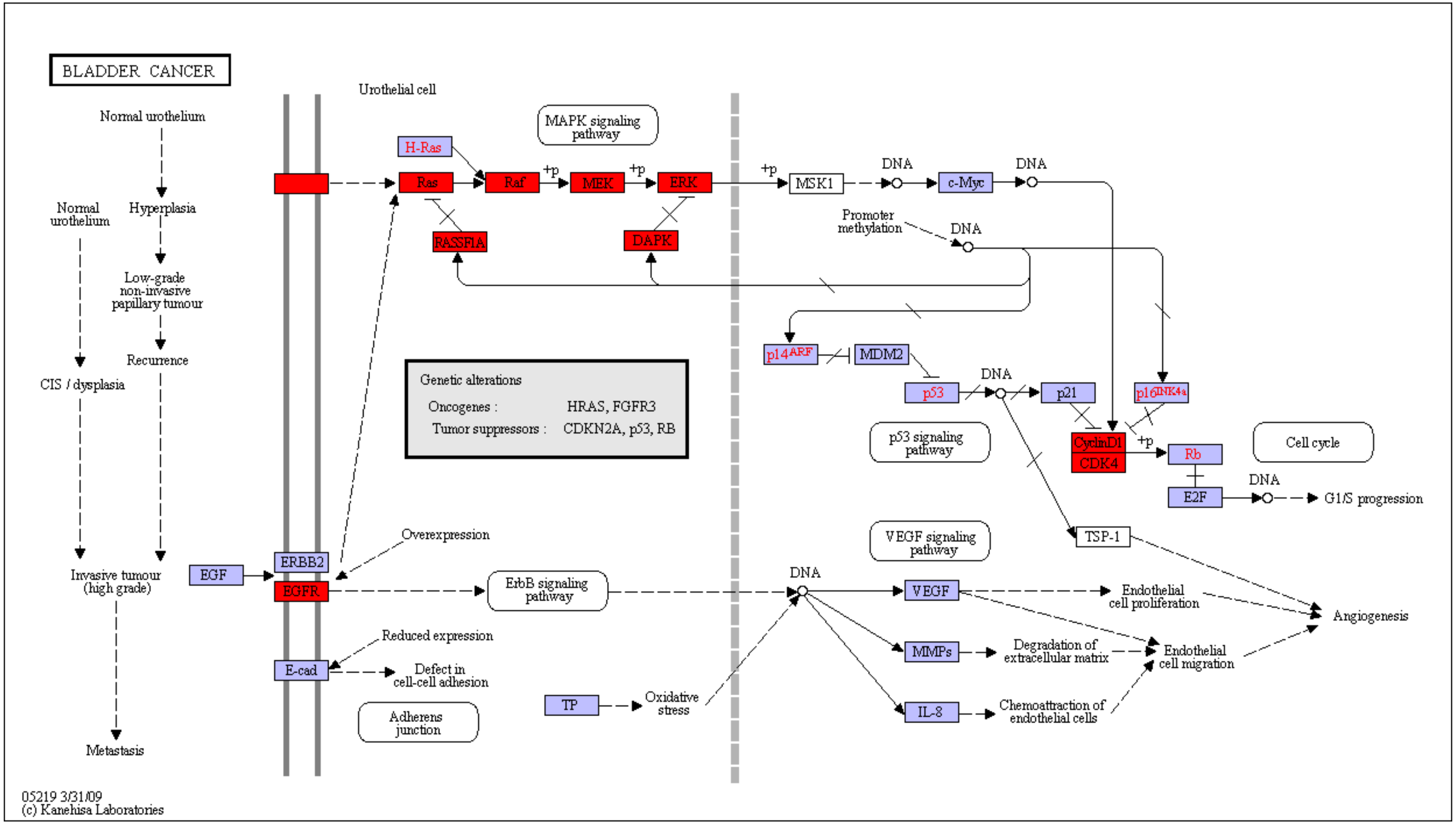

Figure 143: ko05219 Bladder cancer

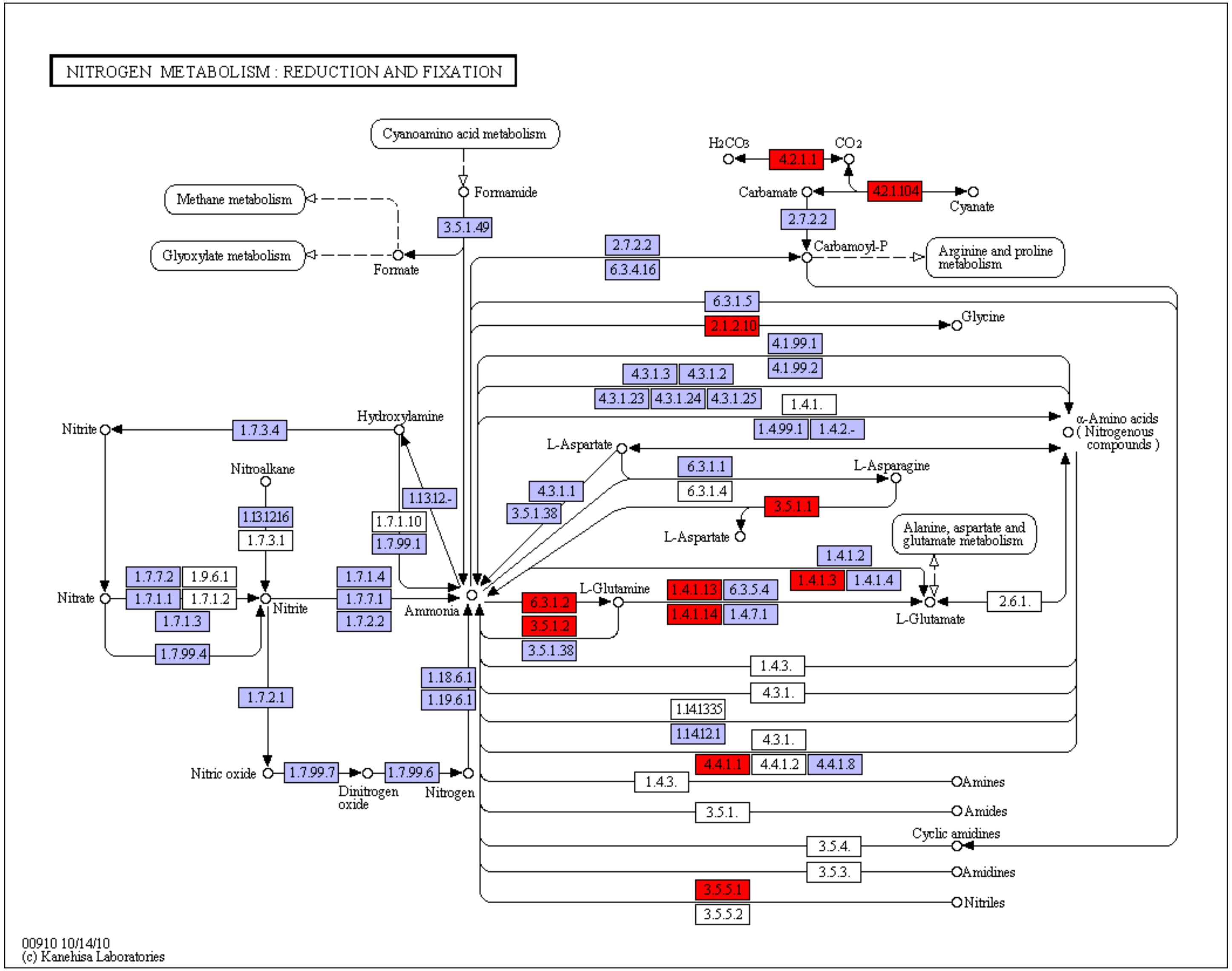

Figure 144: ko00910 Nitrogen metabolism

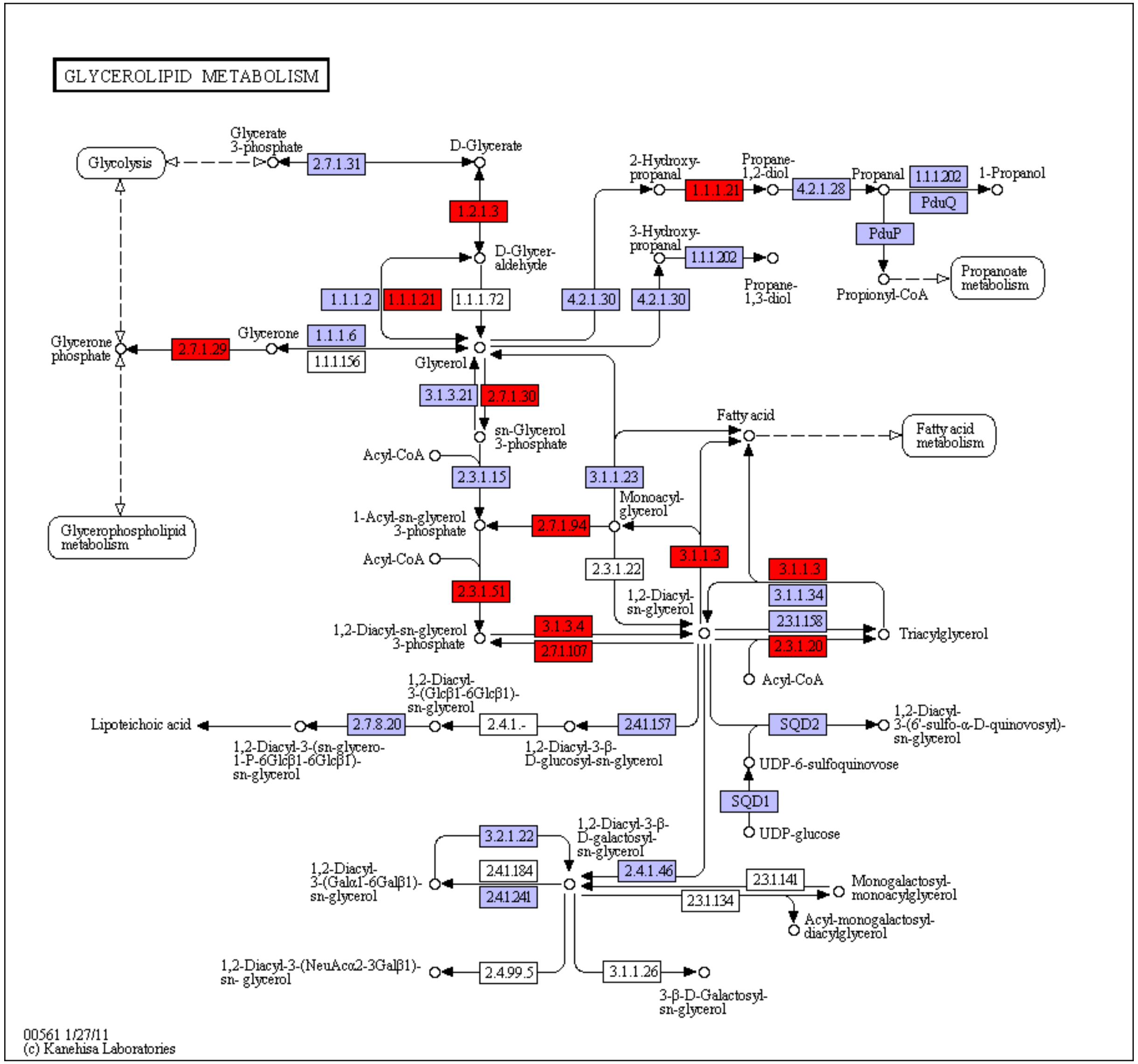

Figure 145: ko00561 Glycerolipid metabolism

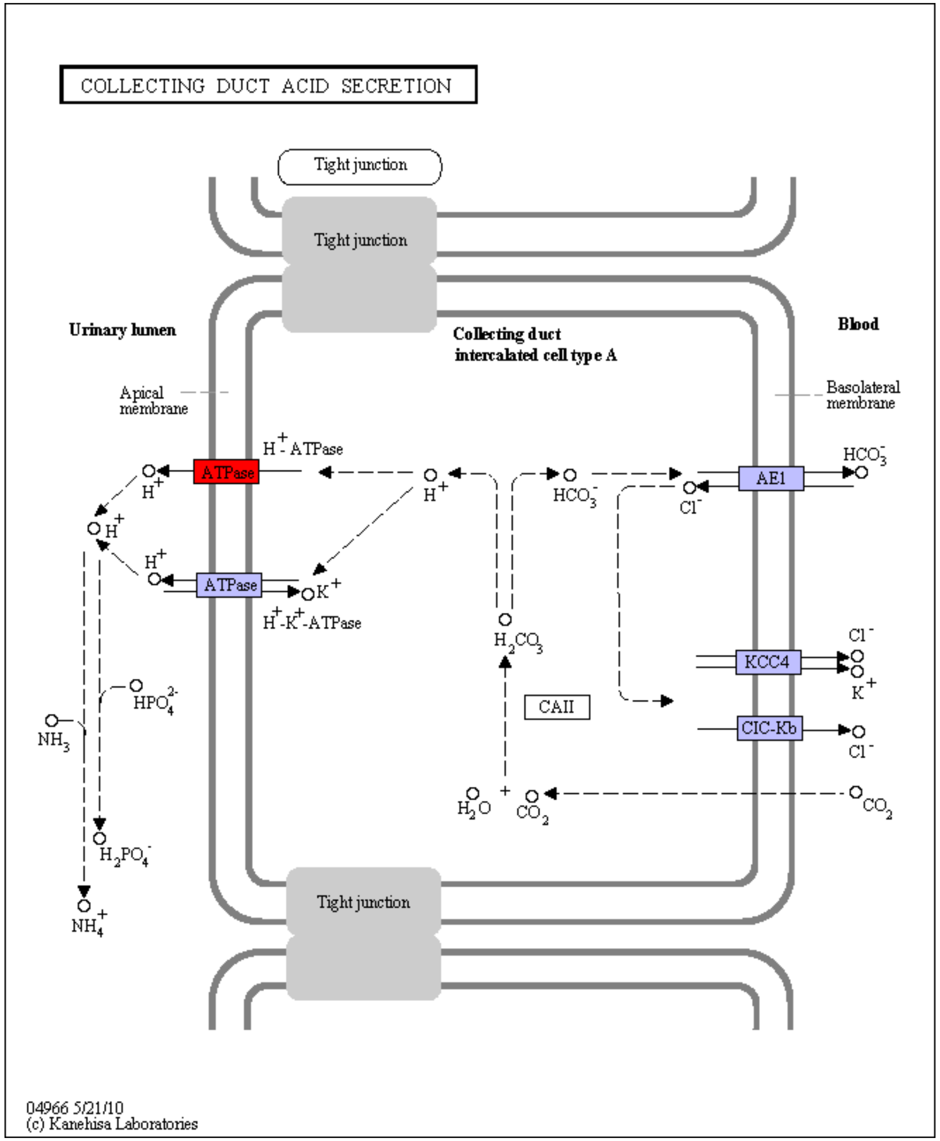

Figure 146: ko04966 Collecting duct acid secretion

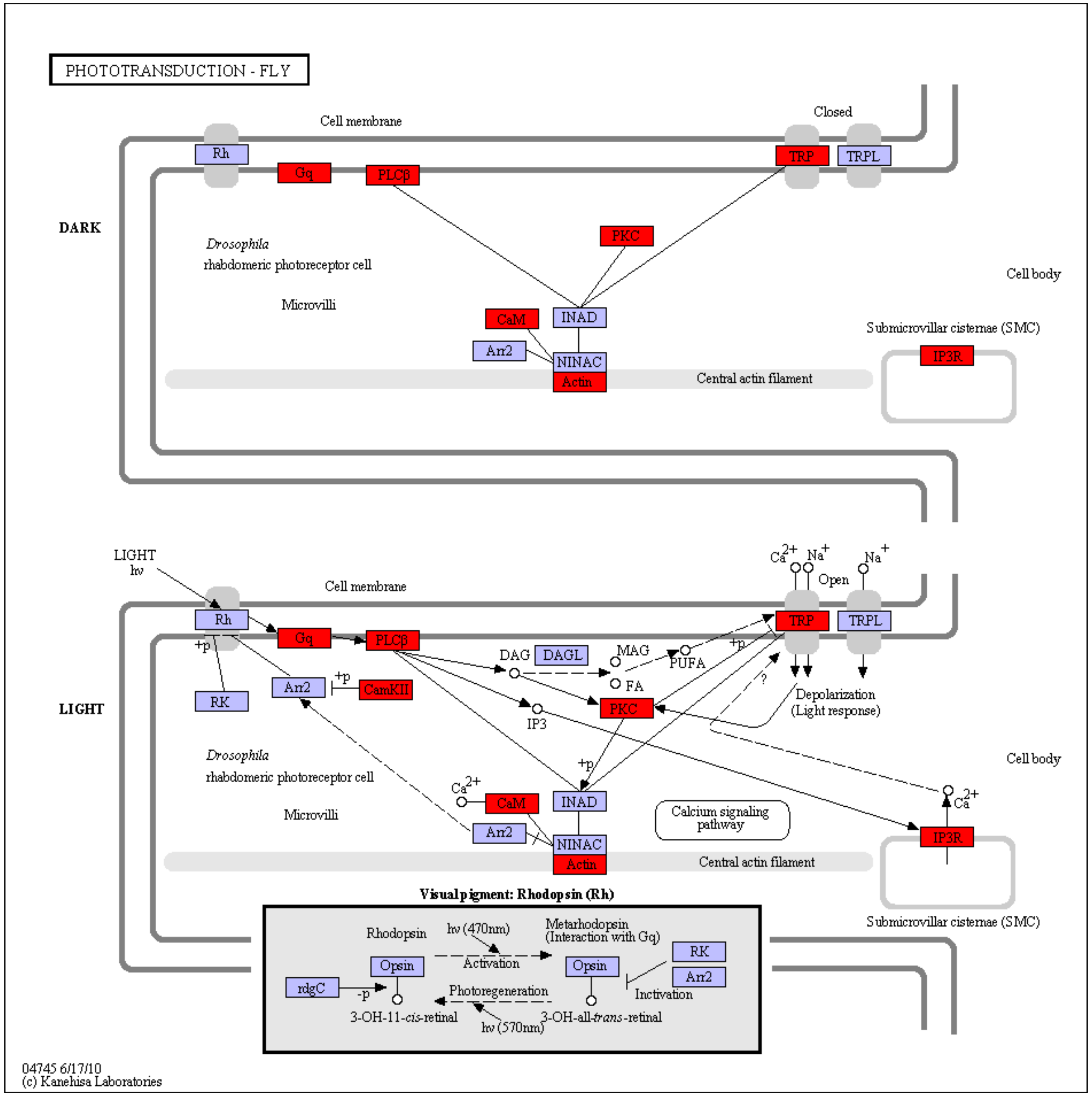

Figure 147: ko04745 Phototransduction - fly

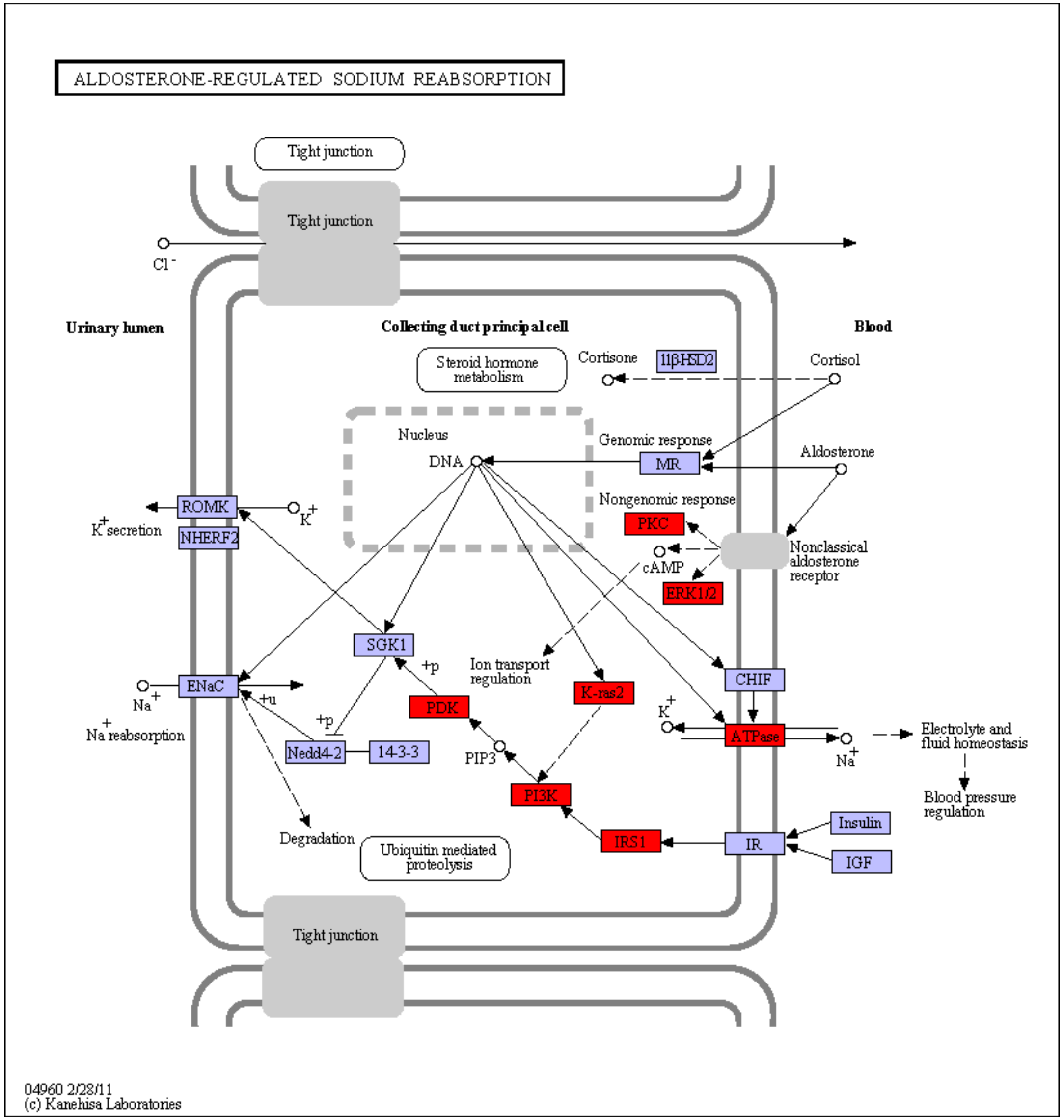

Figure 148: ko04960 Aldosterone-regulated sodium reabsorption

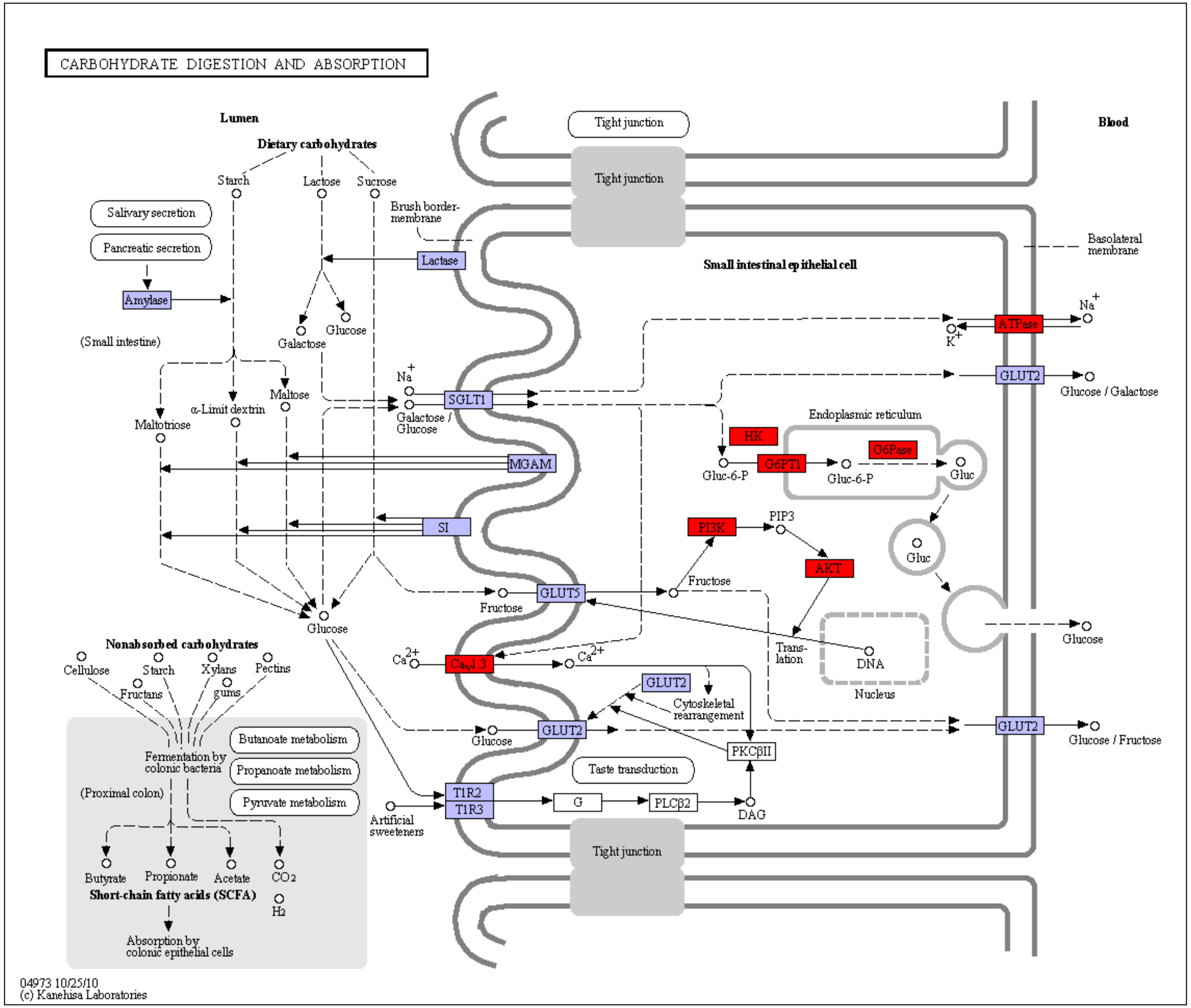

Figure 149: ko04973 Carbohydrate digestion and absorption

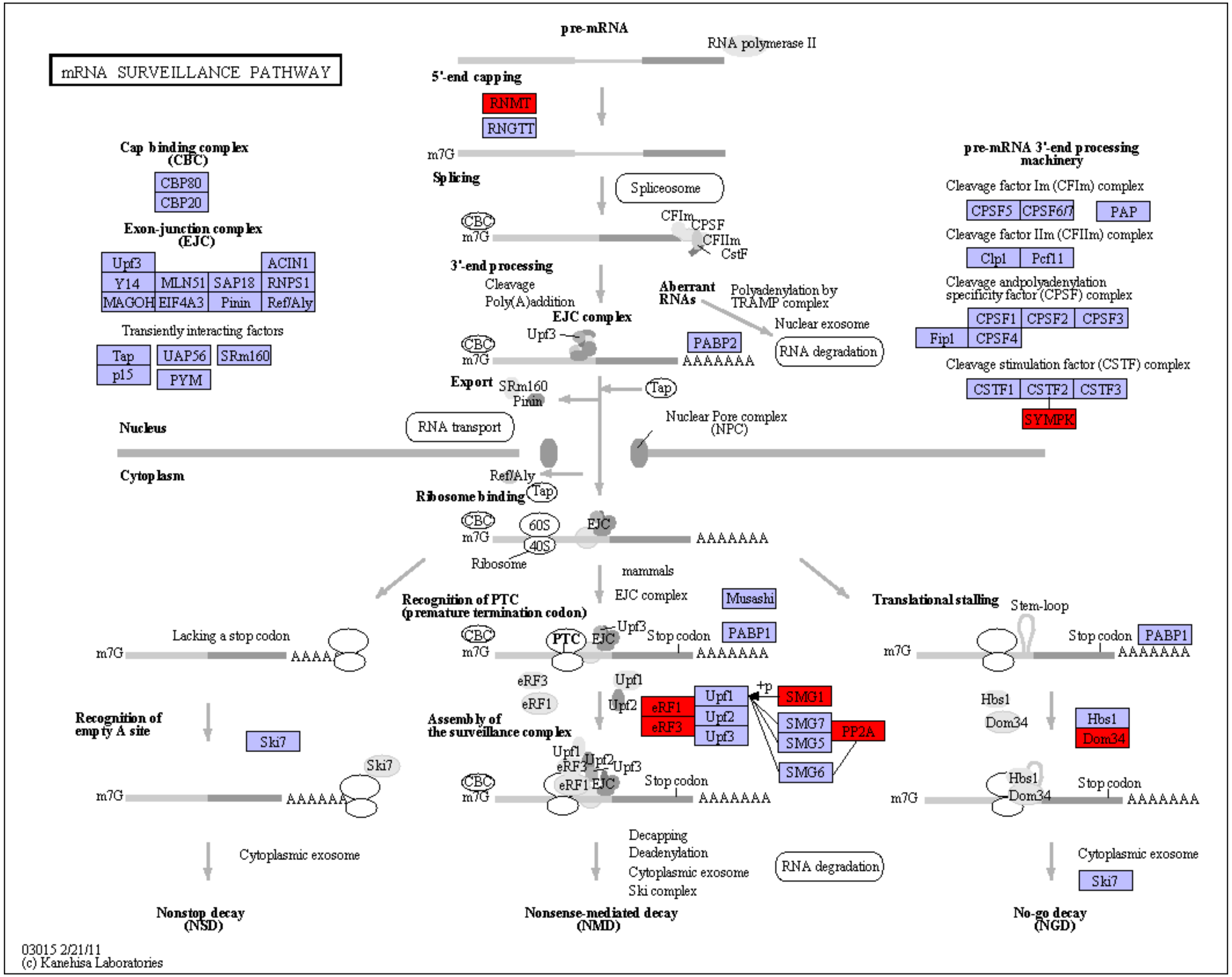

Figure 150: ko03015 mRNA surveillance pathway

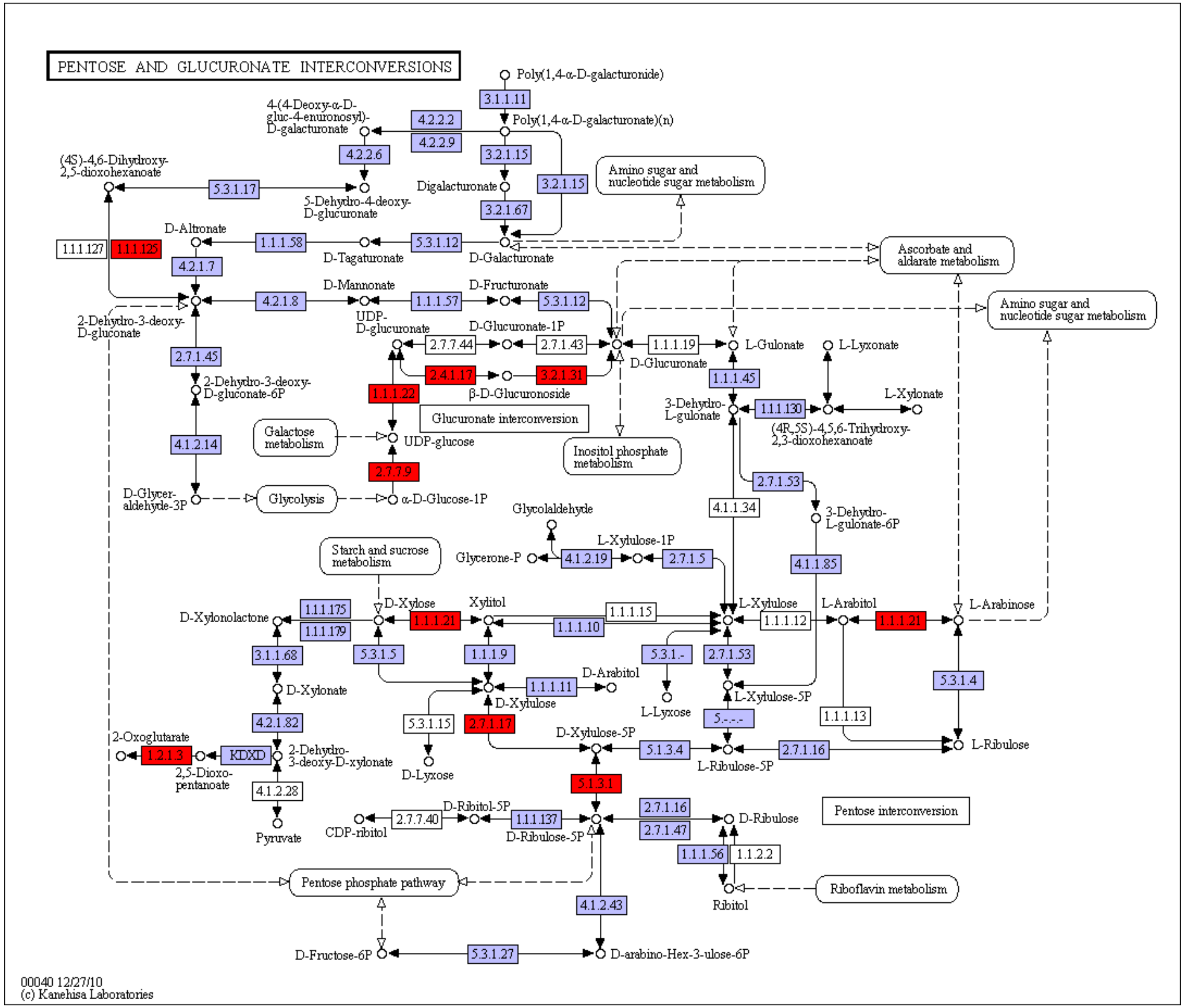

Figure 151: ko00040 Pentose and glucuronate interconversions

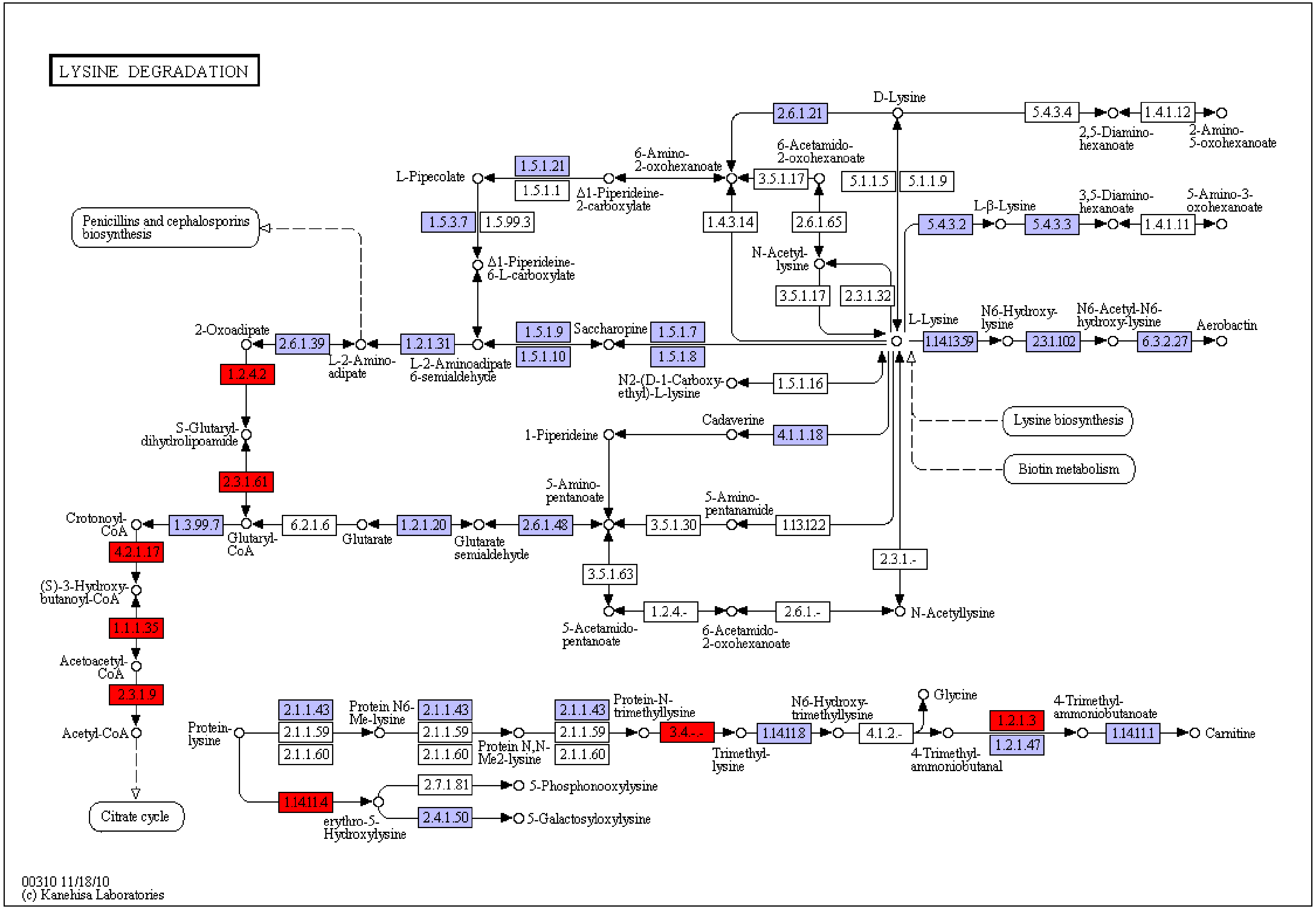

Figure 152: ko00310 Lysine degradation

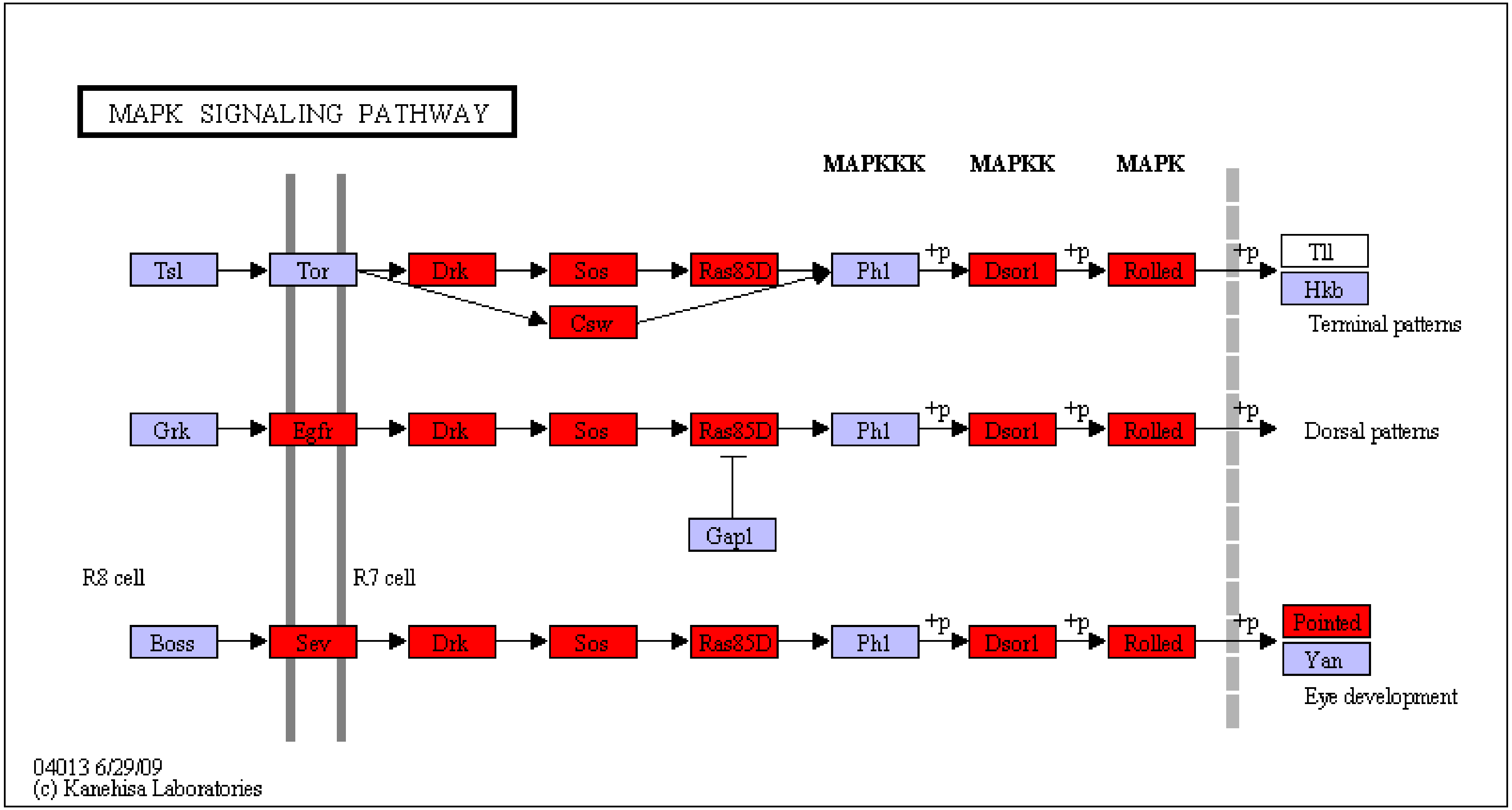

Figure 153: ko04013 MAPK signaling pathway - fly

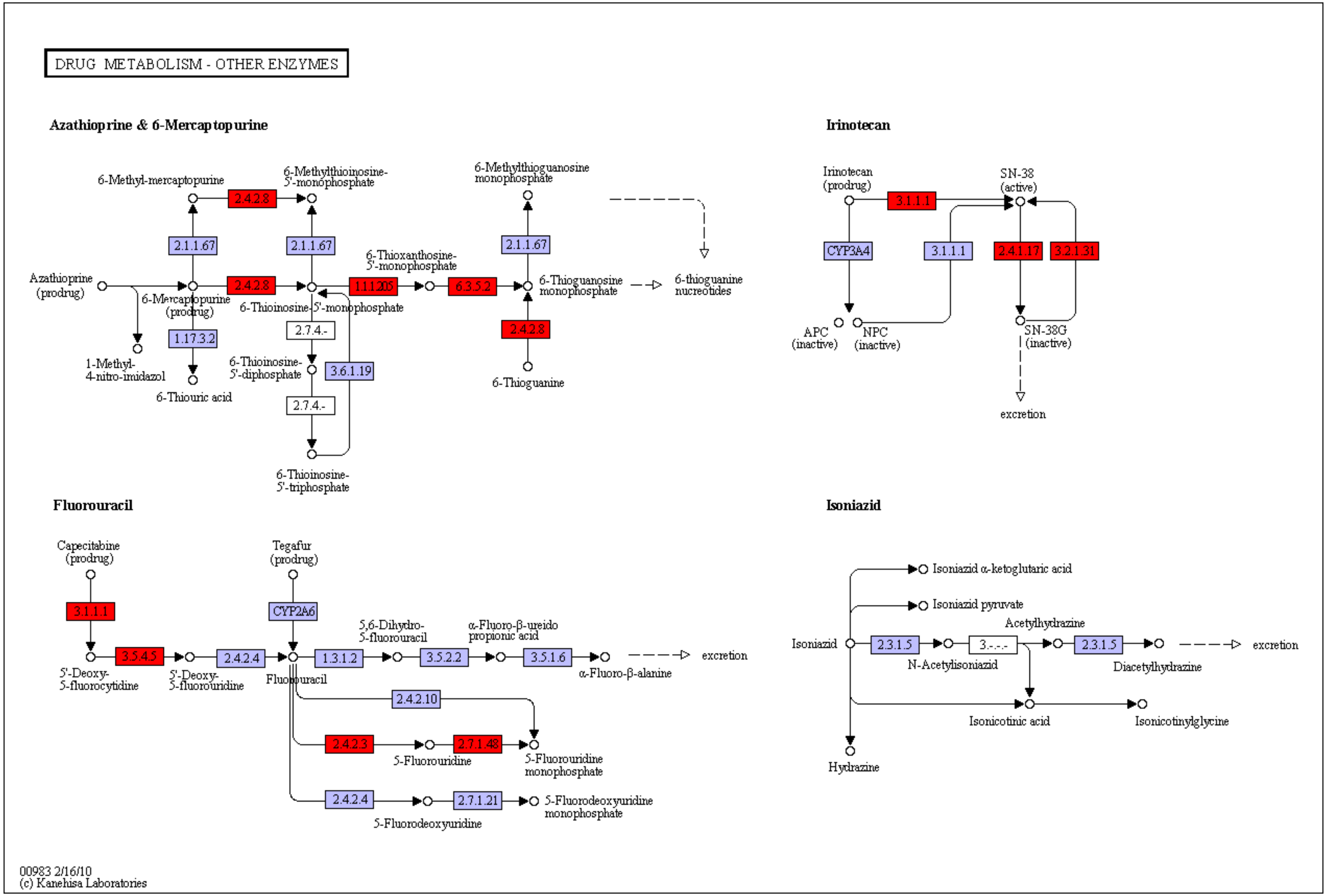

Figure 154: ko00983 Drug metabolism - other enzymes

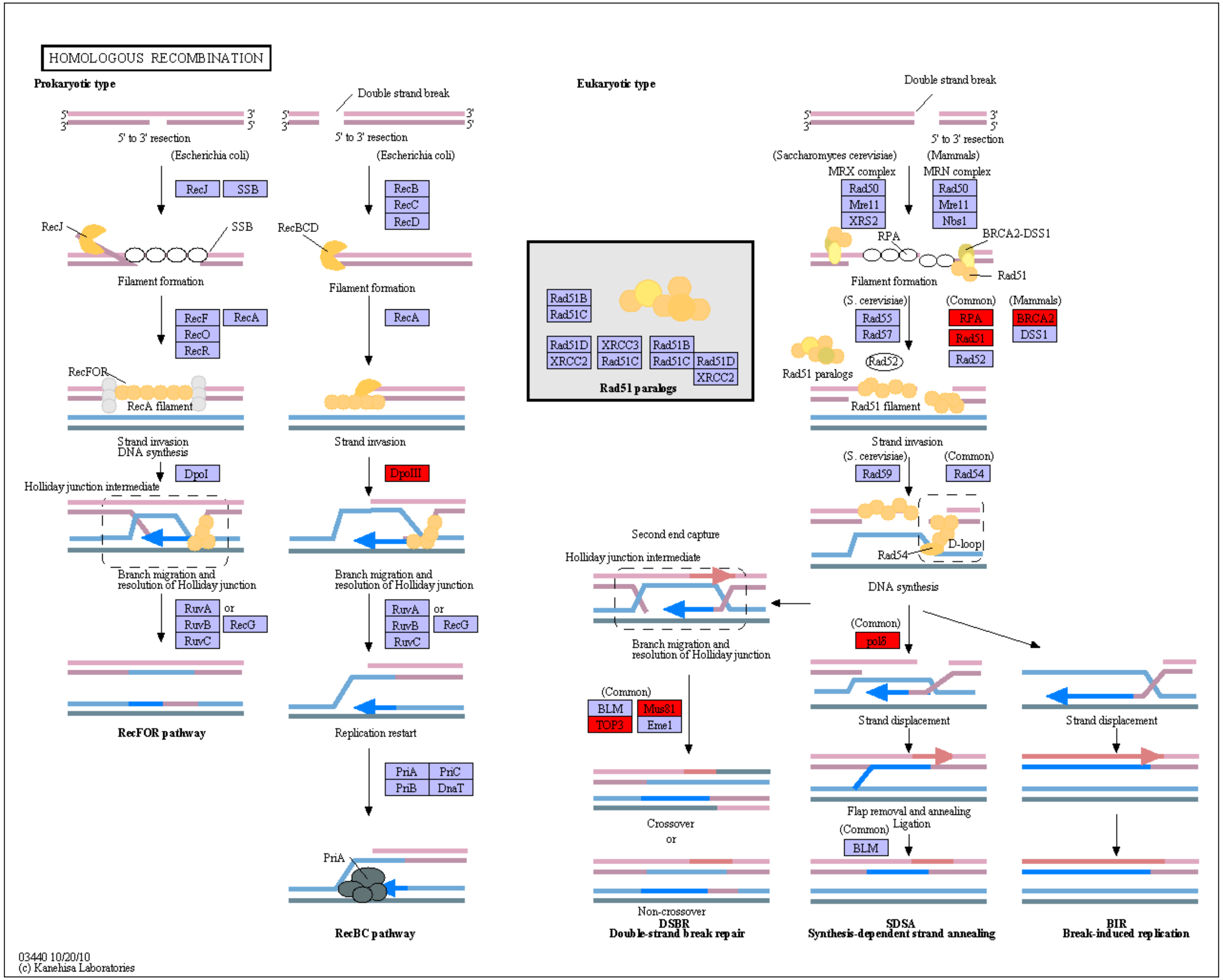

Figure 155: ko03440 Homologous recombination

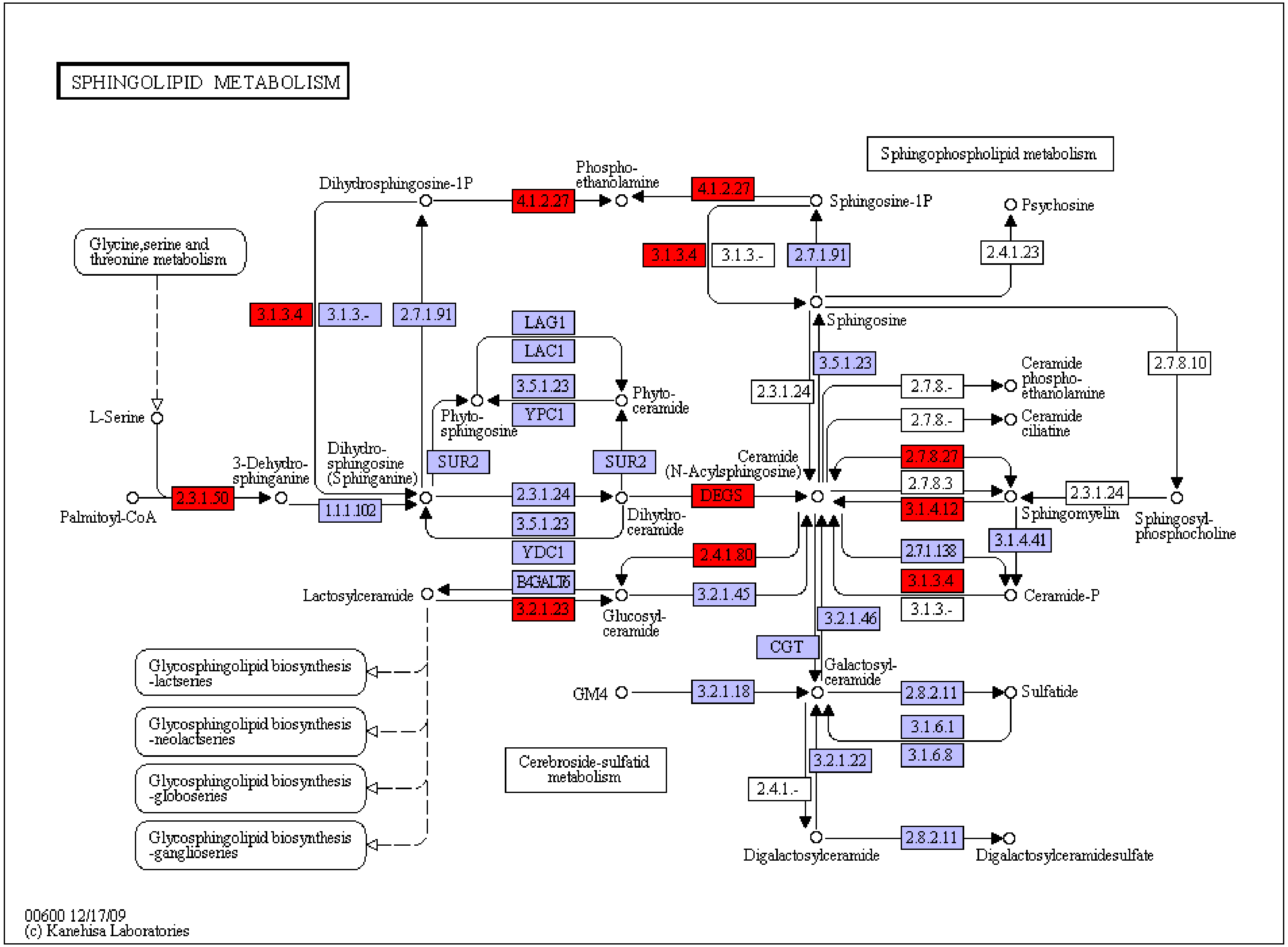

Figure 156: ko00600 Sphingolipid metabolism

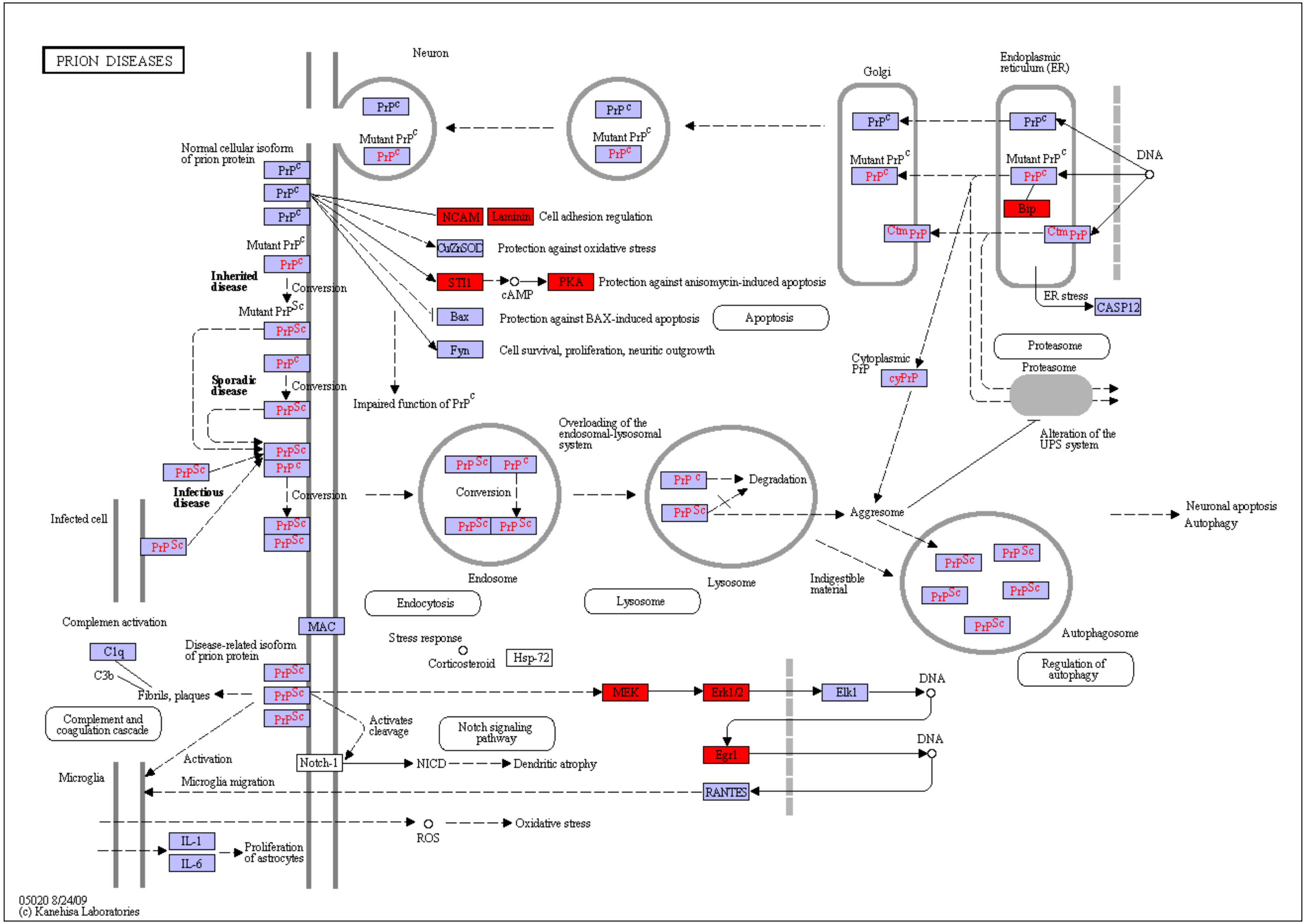

Figure 157: ko05020 Prion diseases

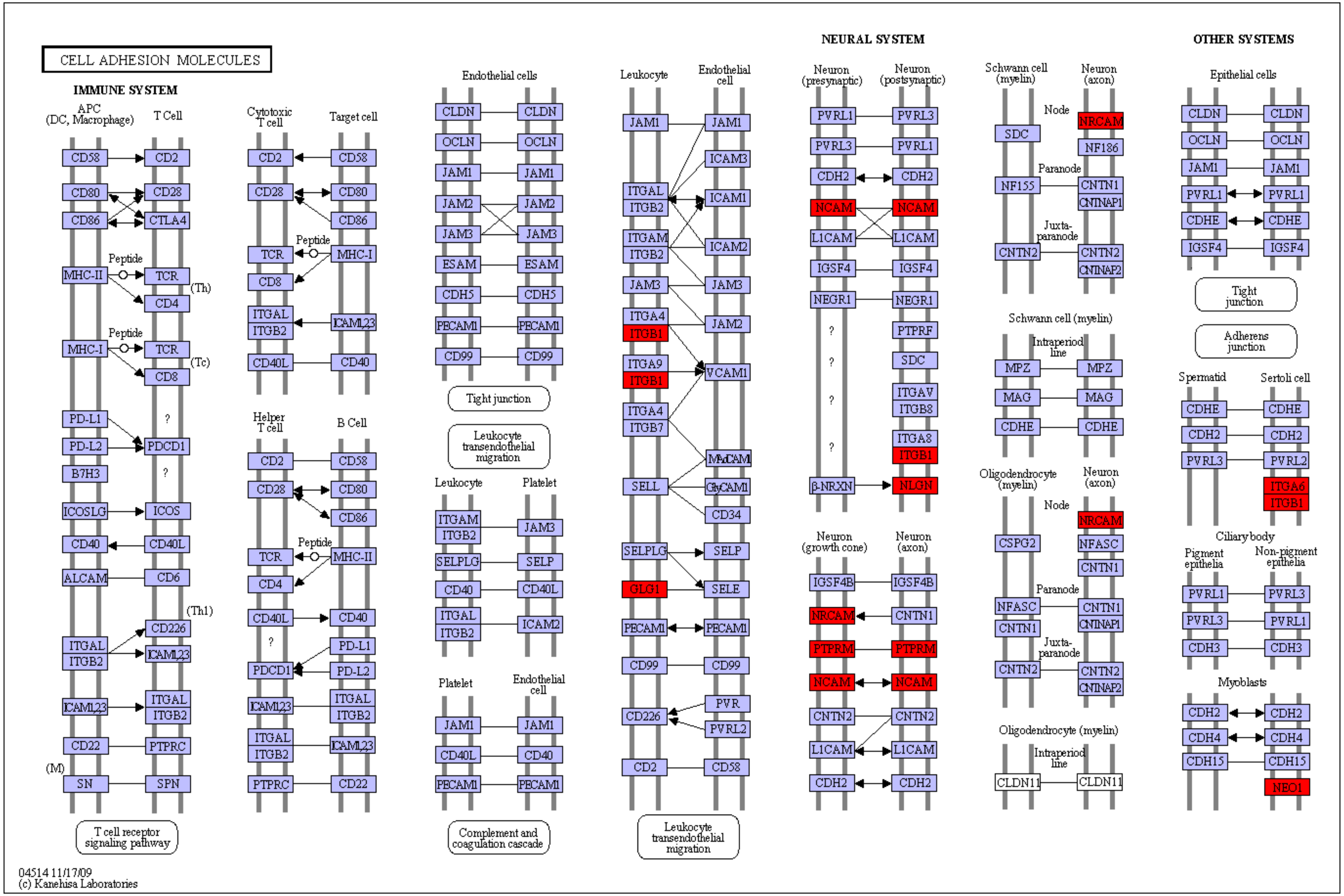

Figure 158: ko04514 Cell adhesion molecules (CAMs)

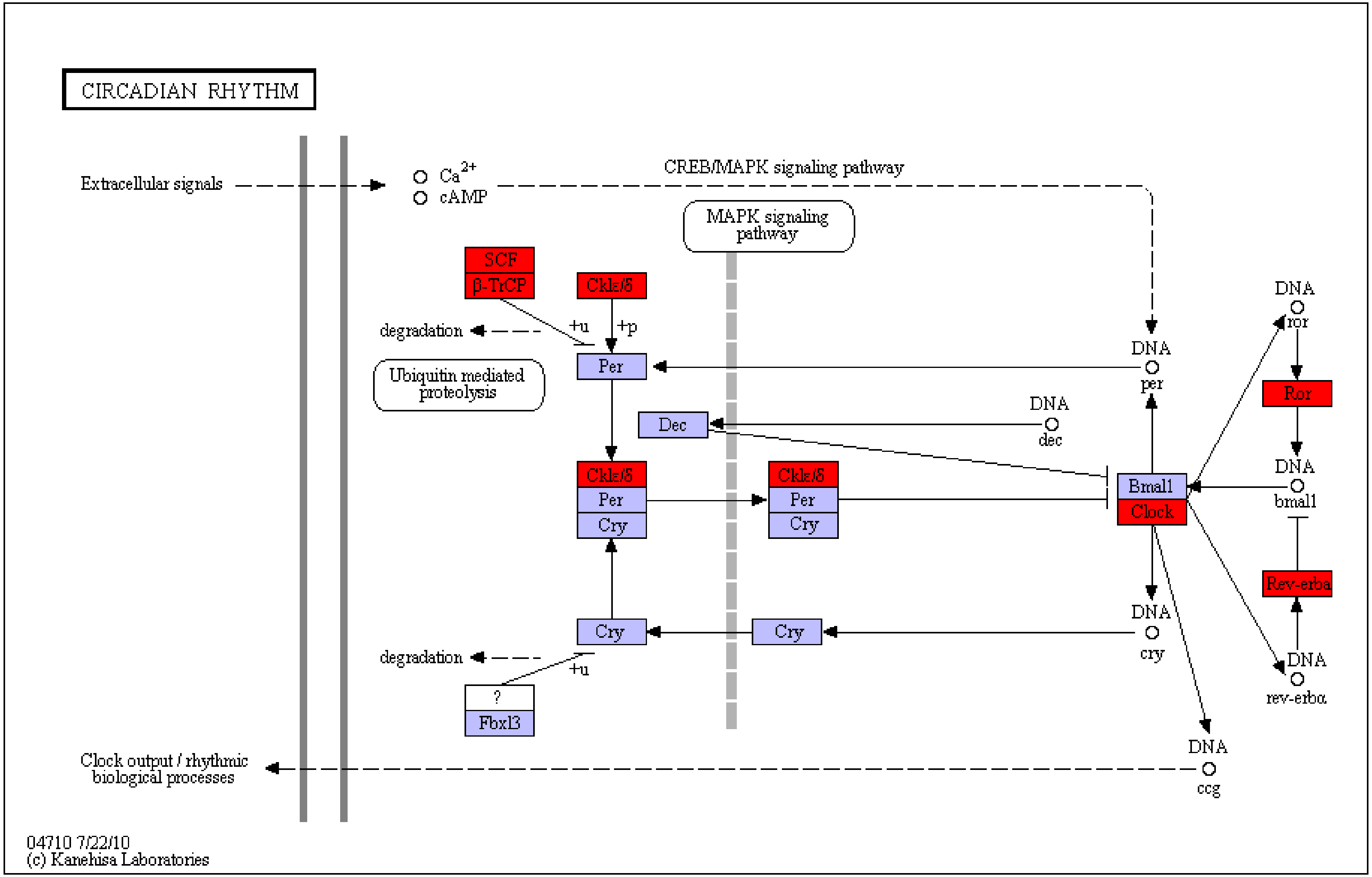

Figure 159: ko04710 Circadian rhythm - mammal

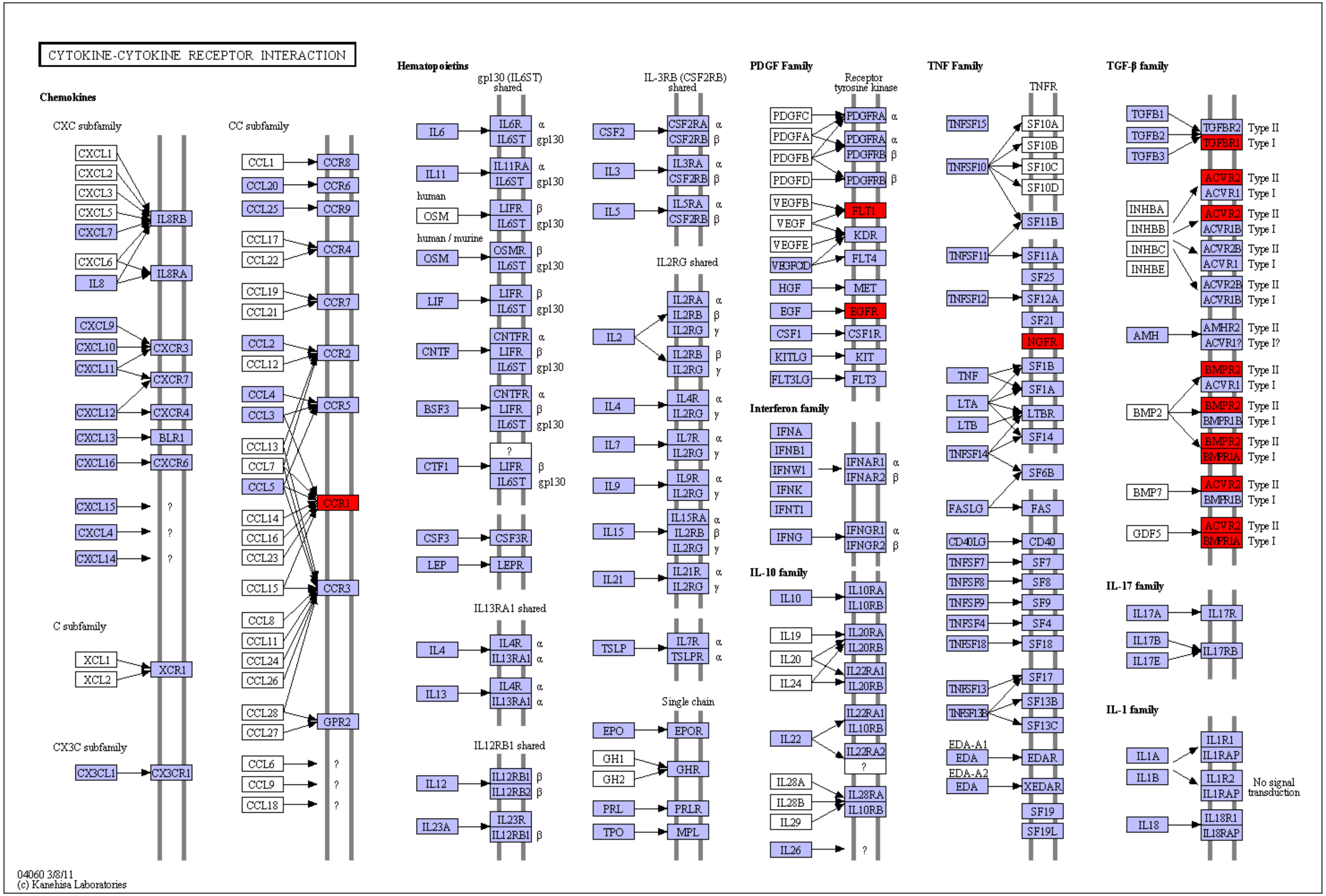

Figure 160: ko04060 Cytokine-cytokine receptor interaction

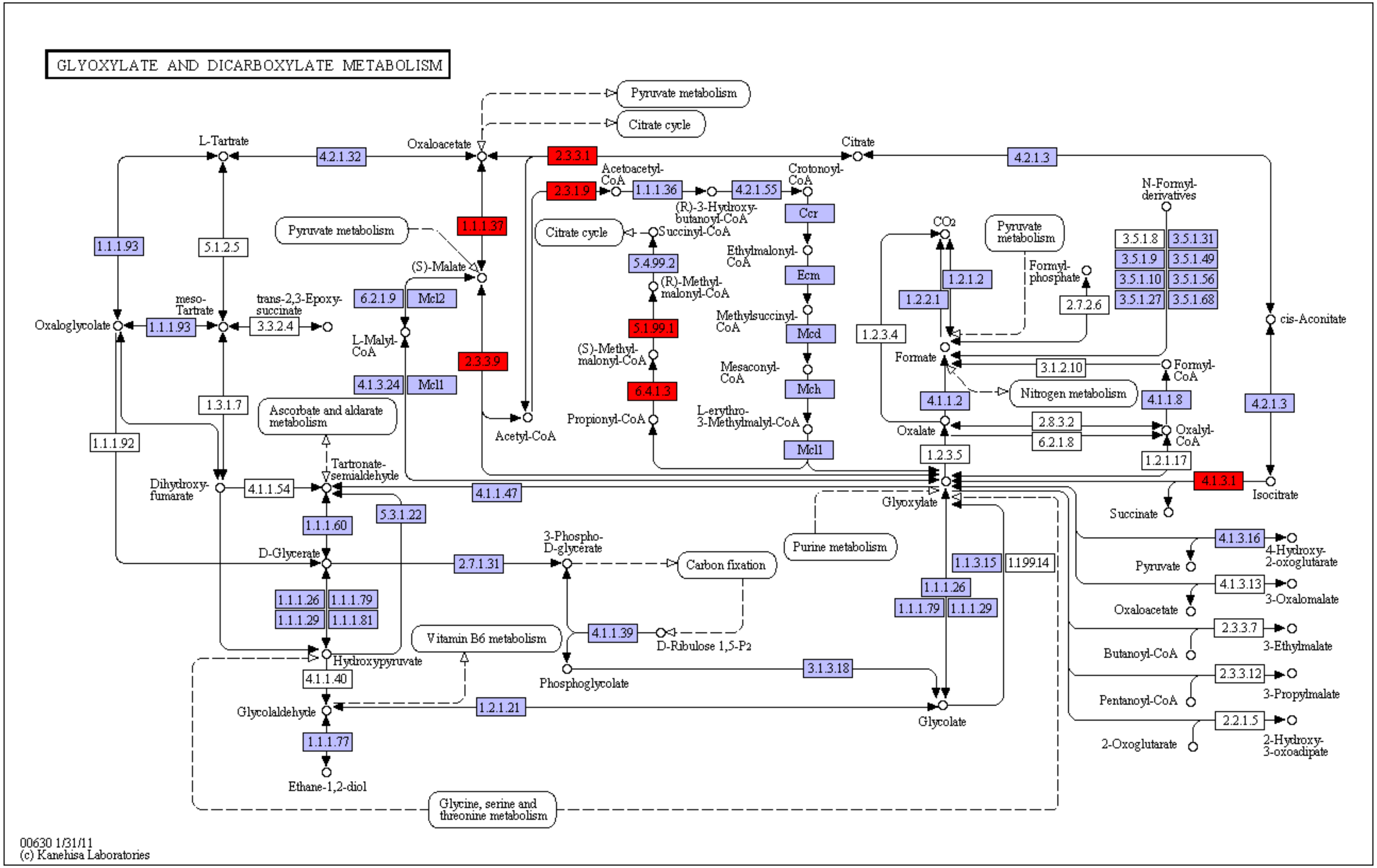

Figure 161: ko00630 Glyoxylate and dicarboxylate metabolism

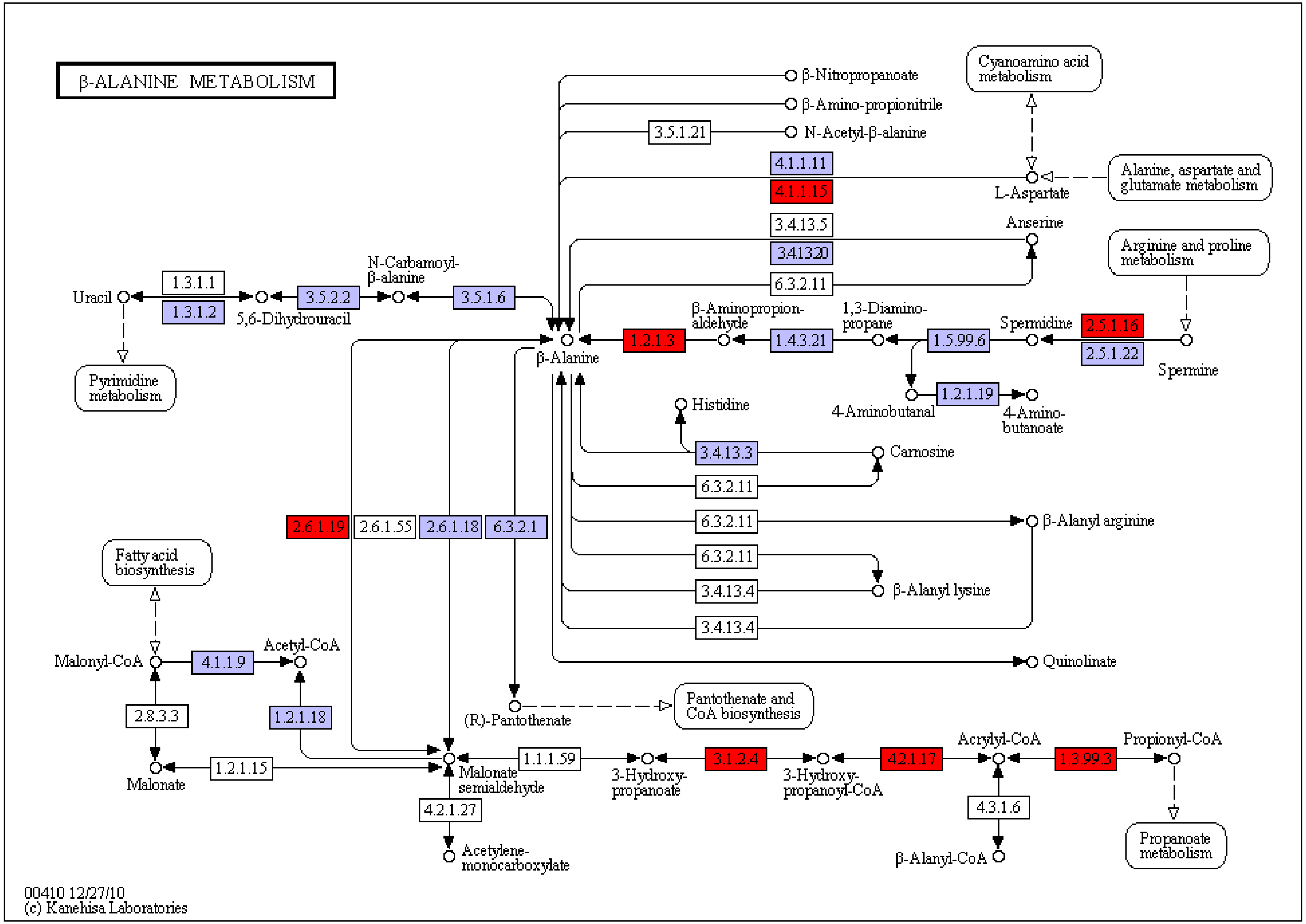

Figure 162: ko00410 beta-Alanine metabolism

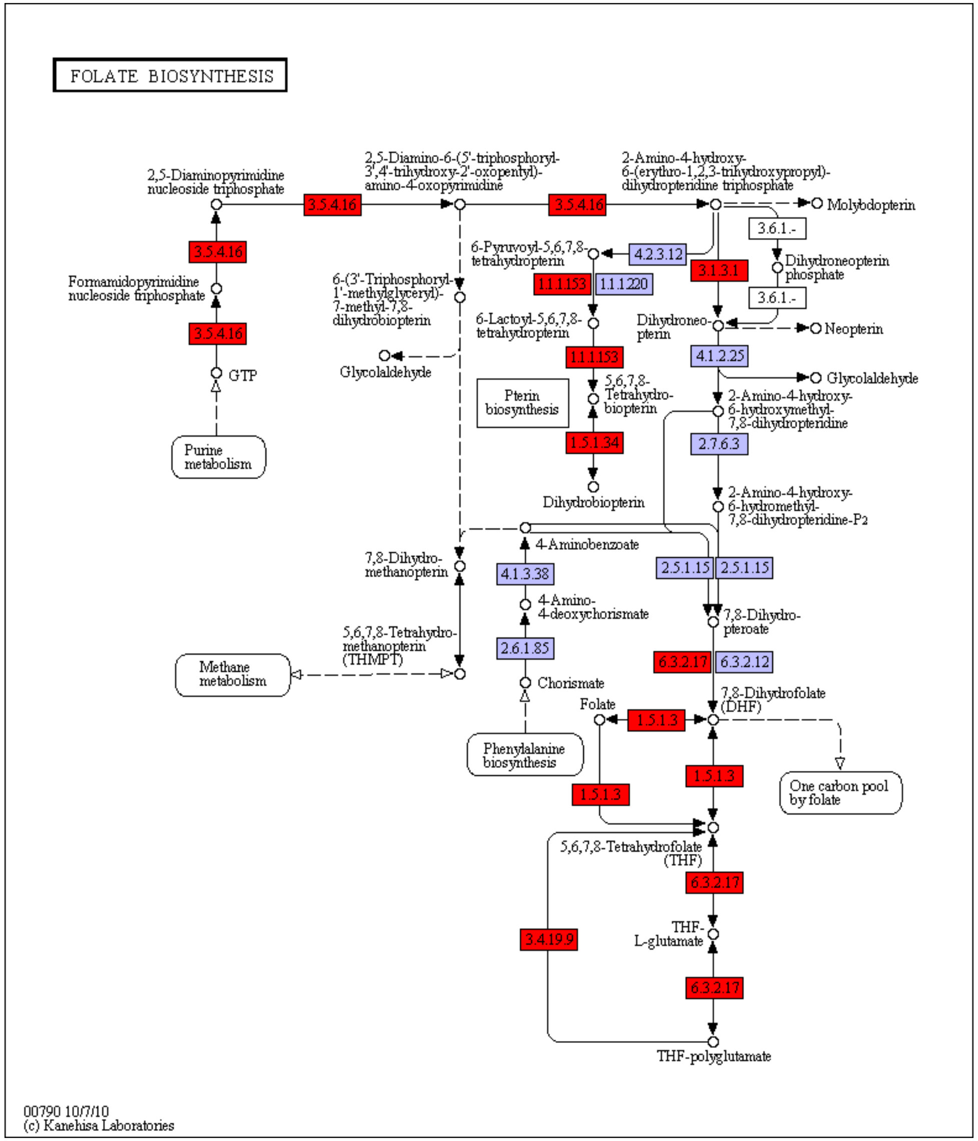

Figure 163: ko00790 Folate biosynthesis

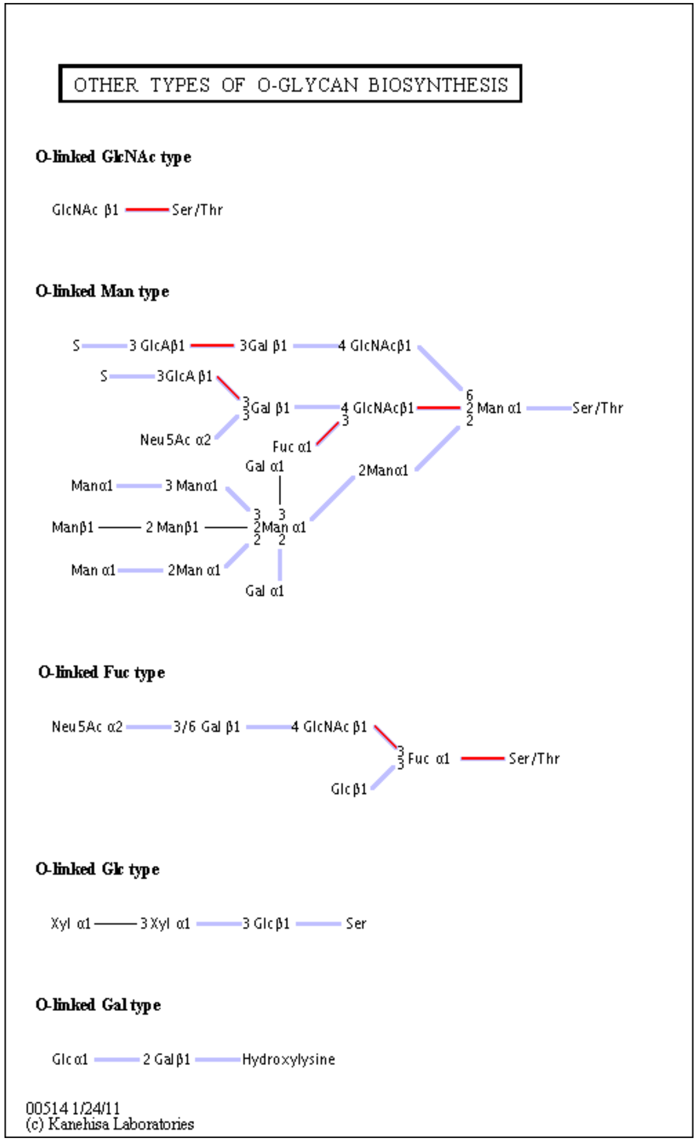

Figure 164: ko00514 Other types of O-glycan biosynthesis

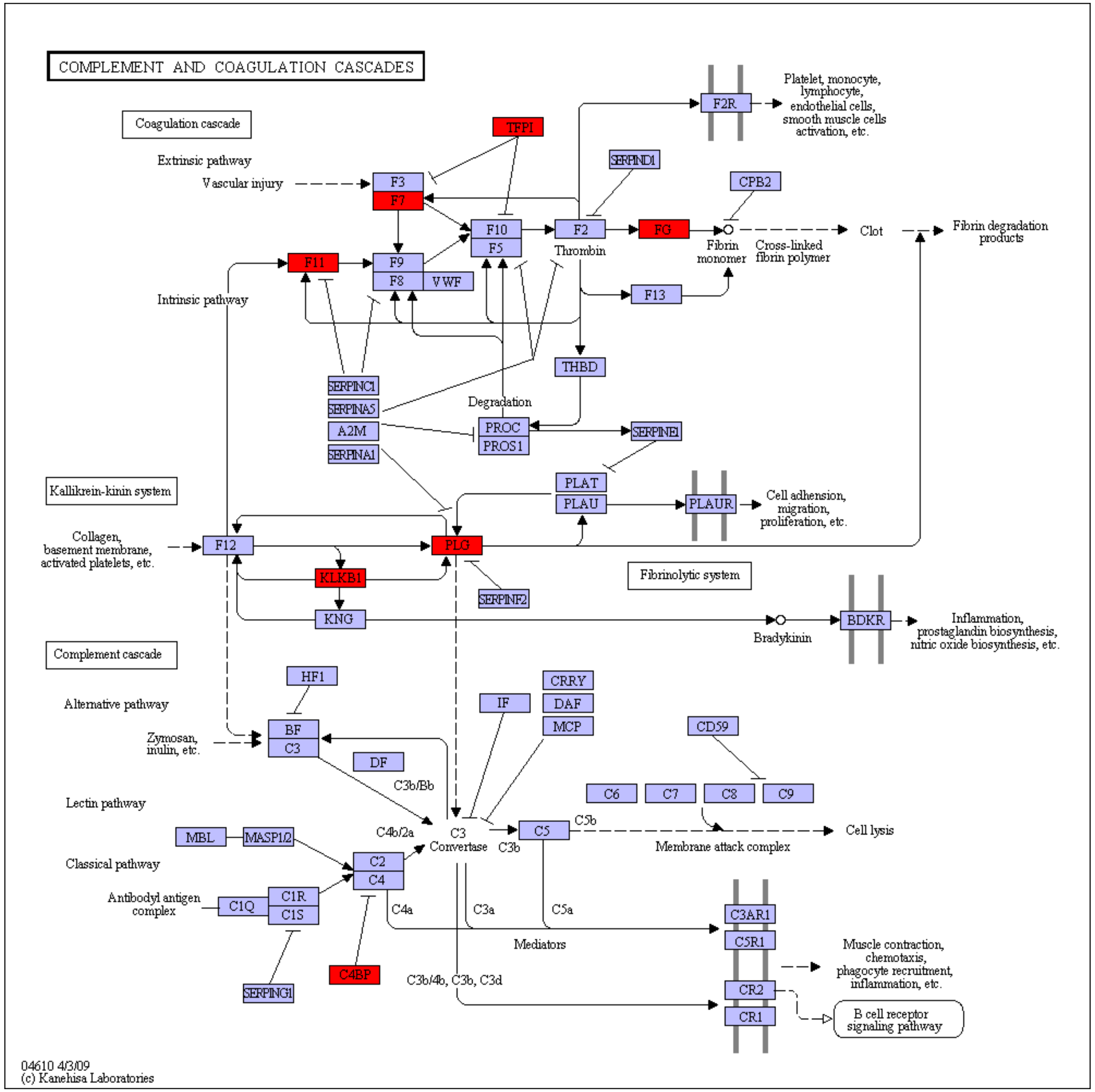

Figure 165: ko04610 Complement and coagulation cascades

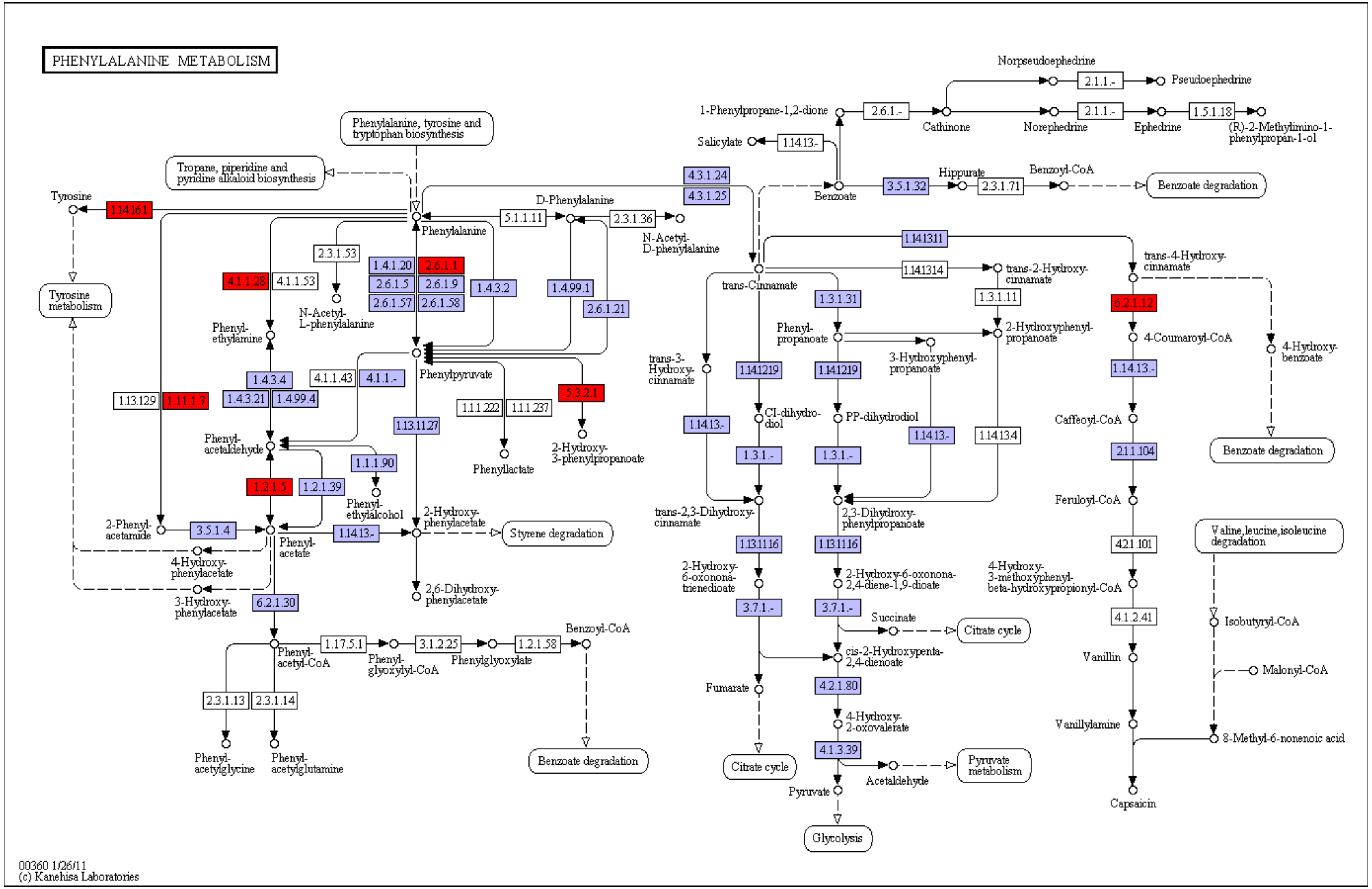

Figure 166: ko00360 Phenylalanine metabolism

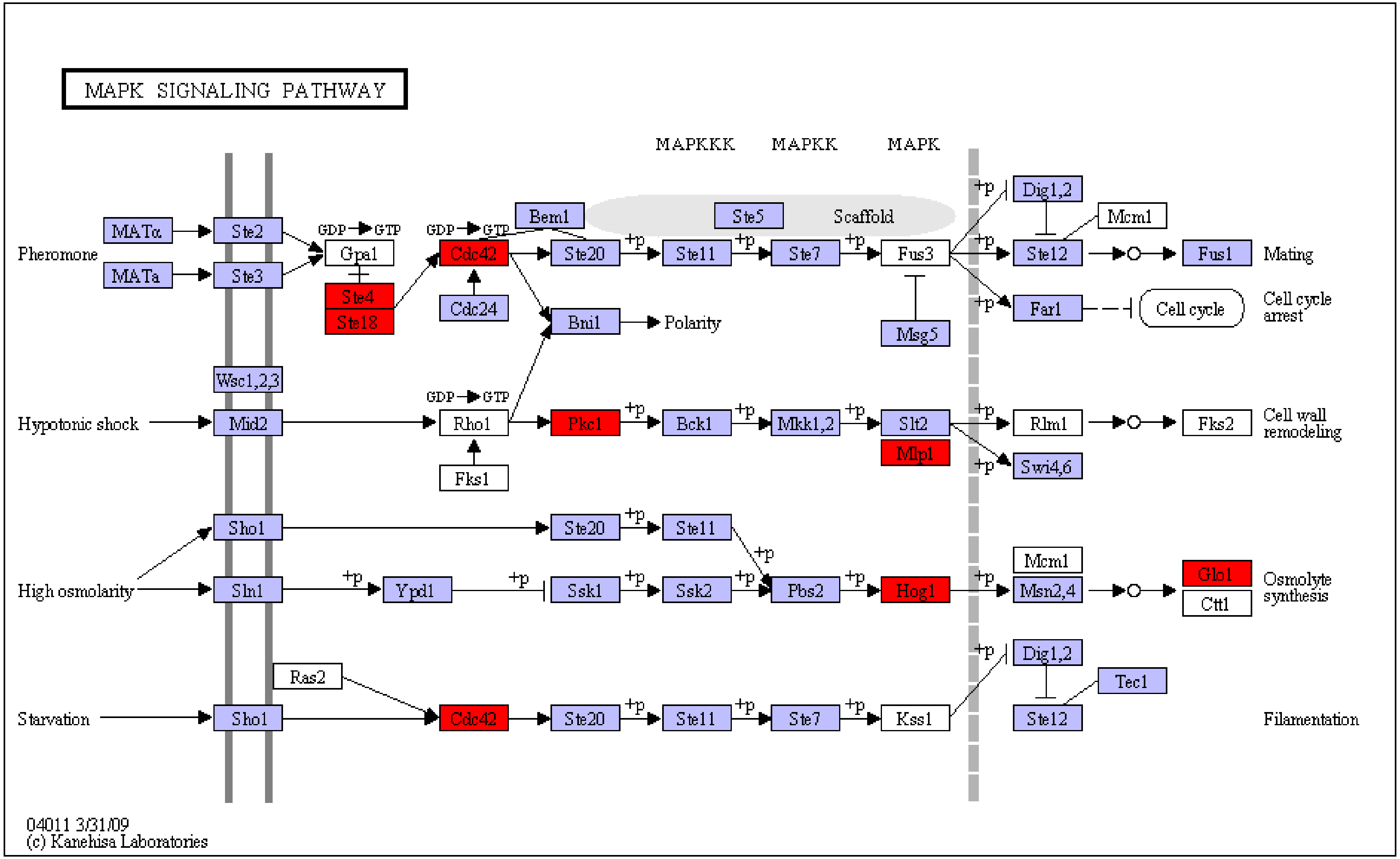

Figure 167: ko04011 MAPK signaling pathway - yeast

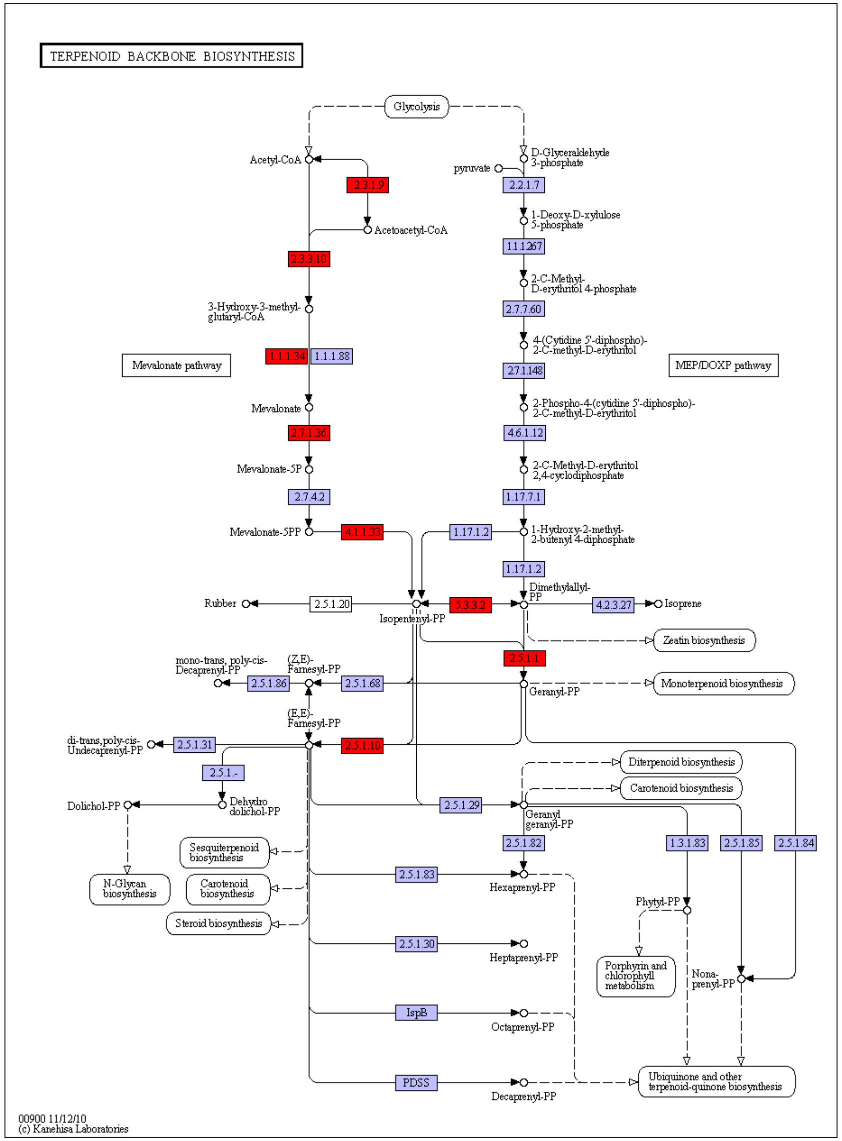

Figure 168: ko00900 Terpenoid backbone biosynthesis

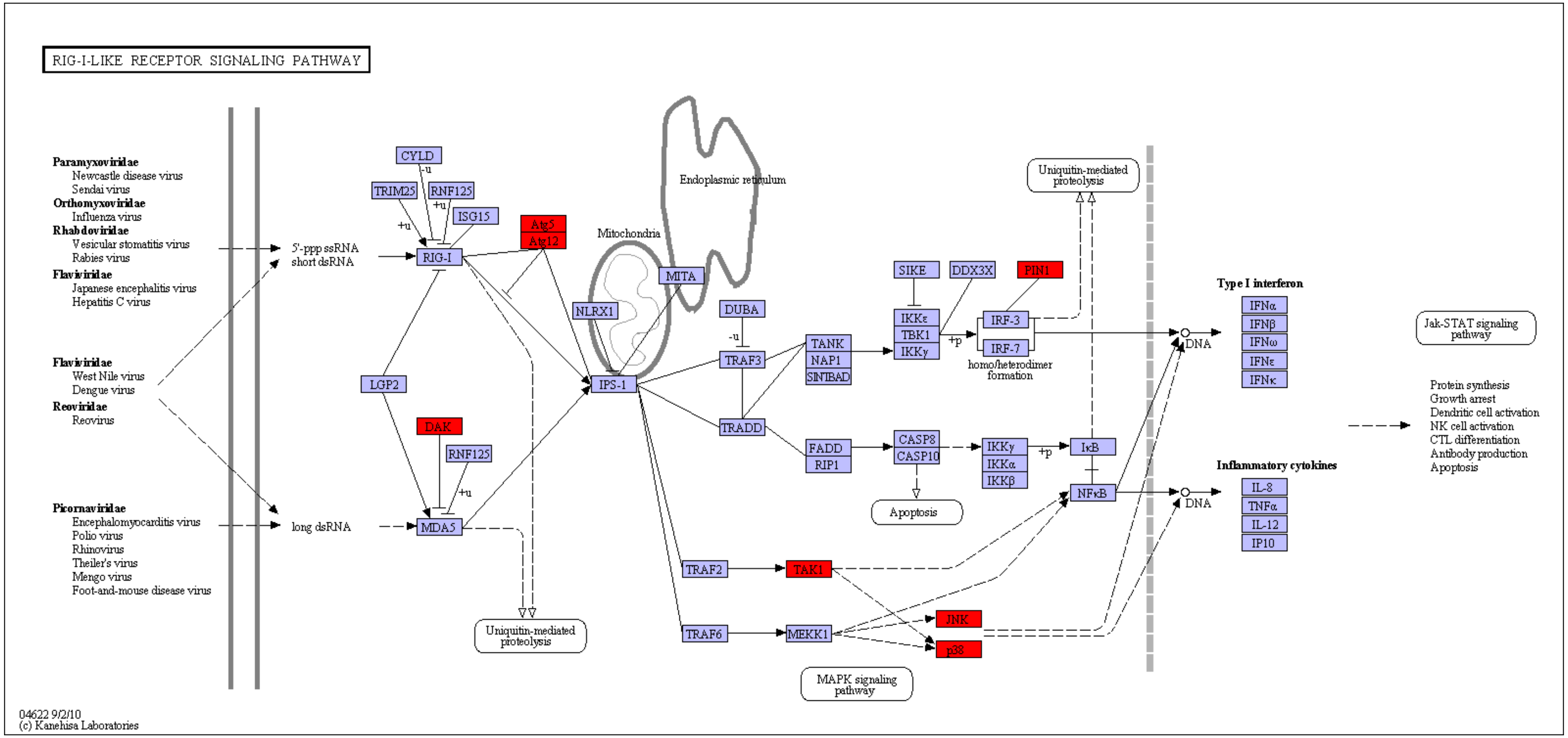

Figure 169: ko04622 RIG-I-like receptor signaling pathway

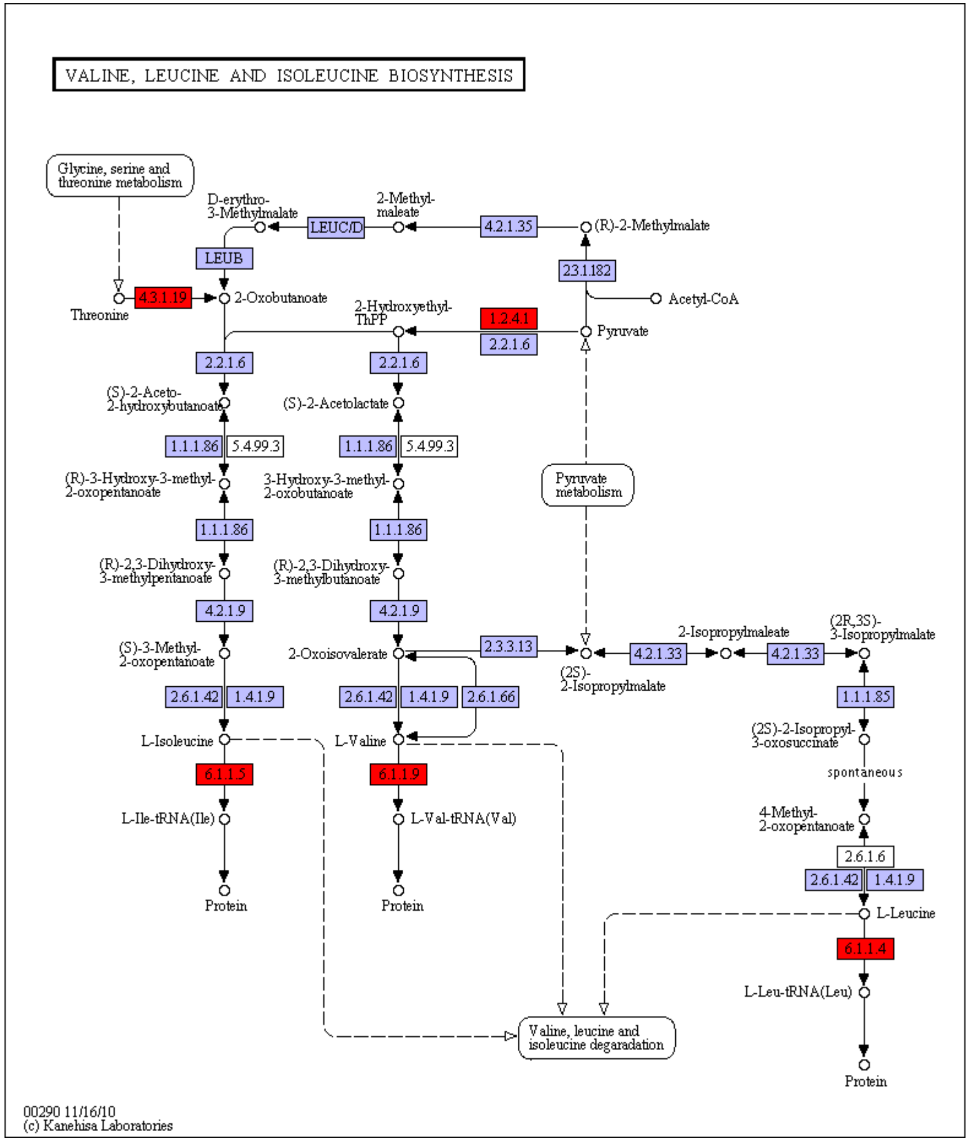

Figure 170: ko00290 Valine, leucine and isoleucine biosynthesis

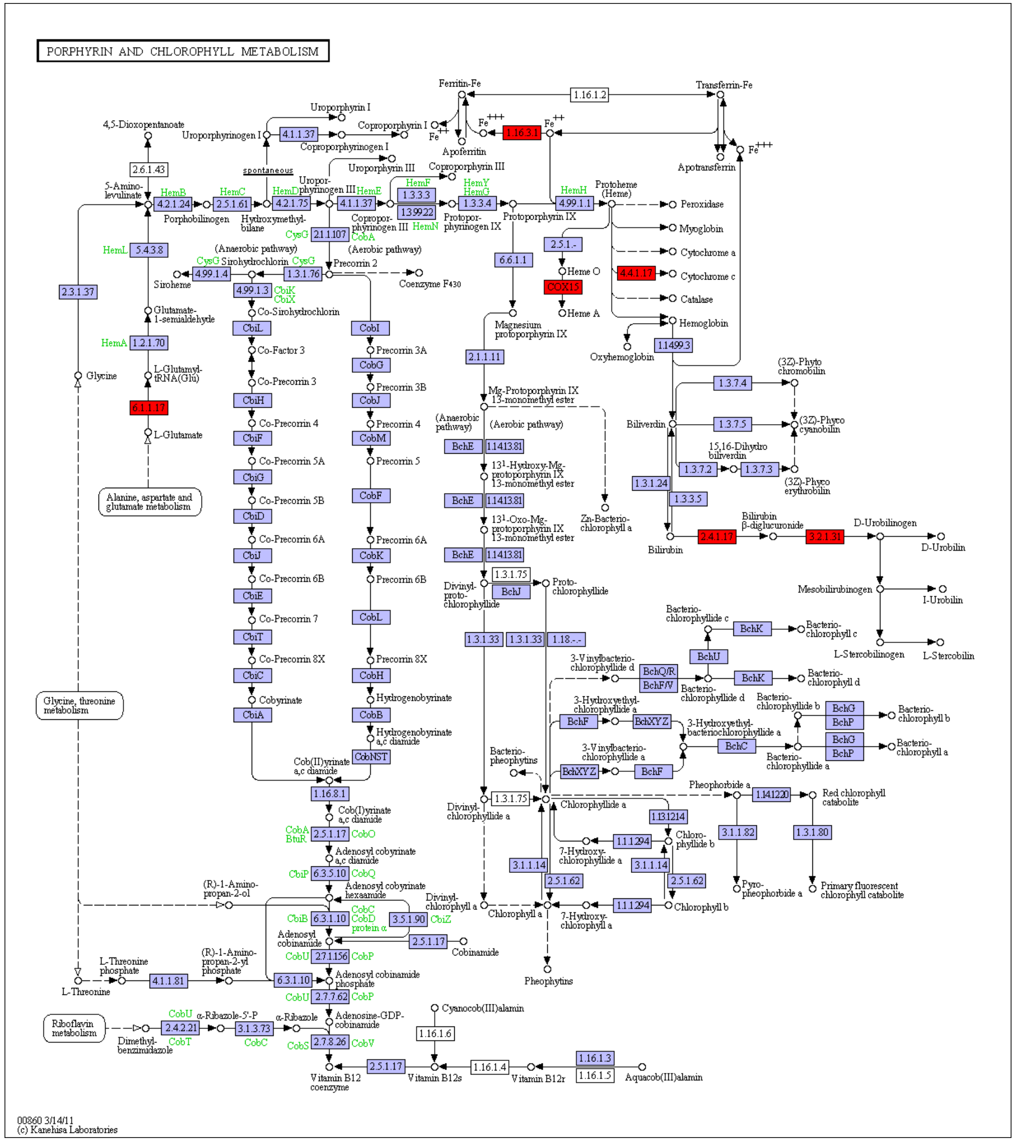

Figure 171: ko00860 Porphyrin and chlorophyll metabolism

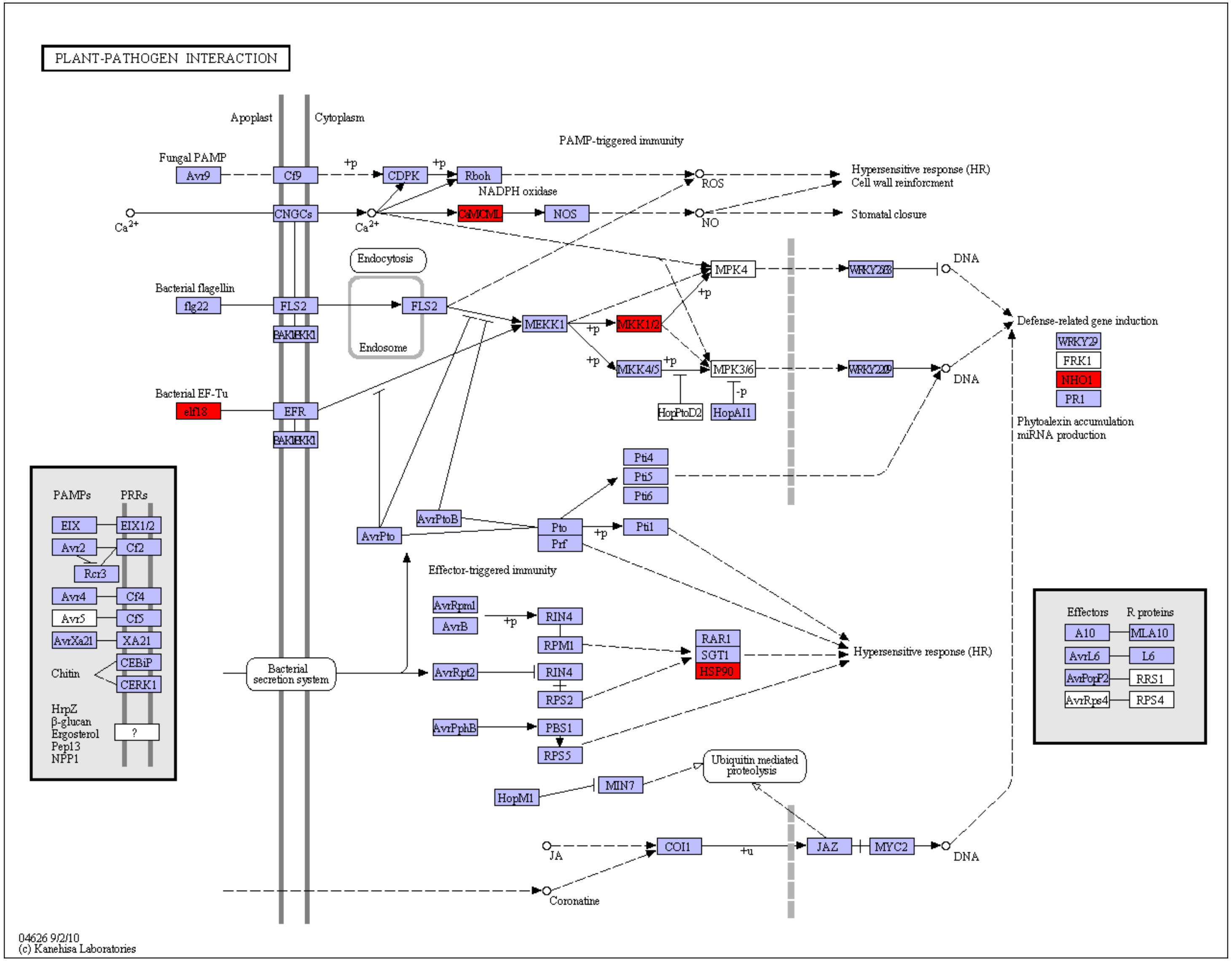

Figure 172: ko04626 Plant-pathogen interaction

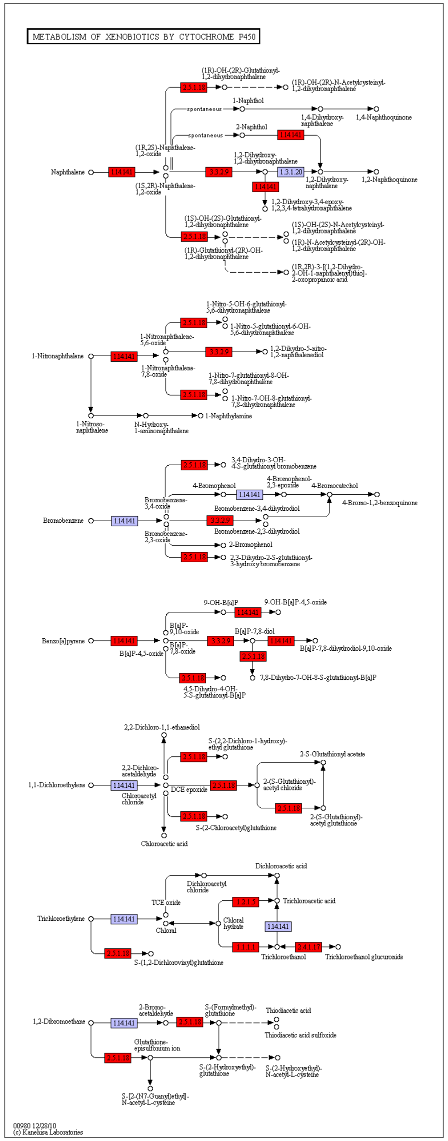

Figure 173: ko00980 Metabolism of xenobiotics by cytochrome P450

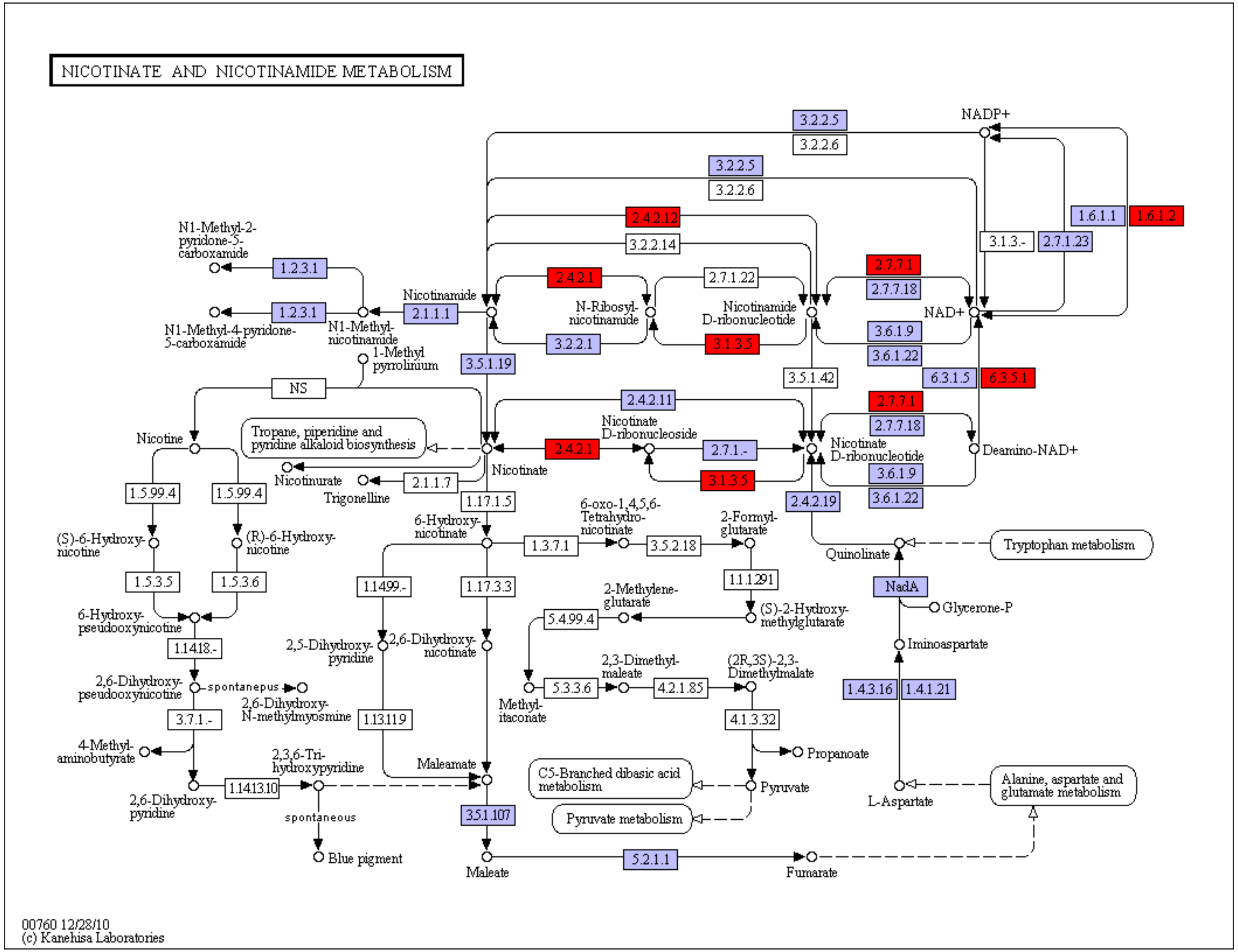

Figure 174: ko00760 Nicotinate and nicotinamide metabolism

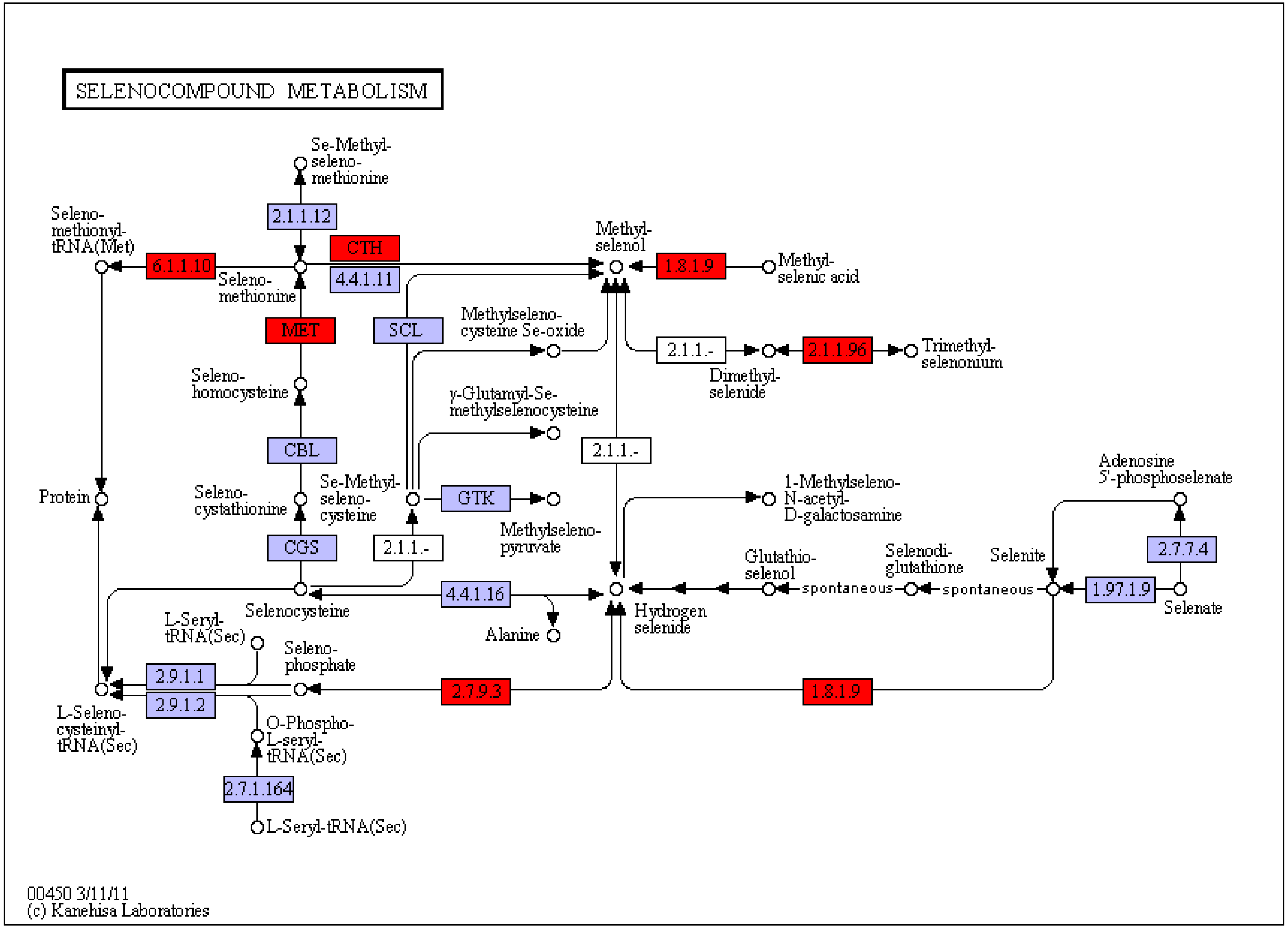

Figure 175: ko00450 Selenocompound metabolism

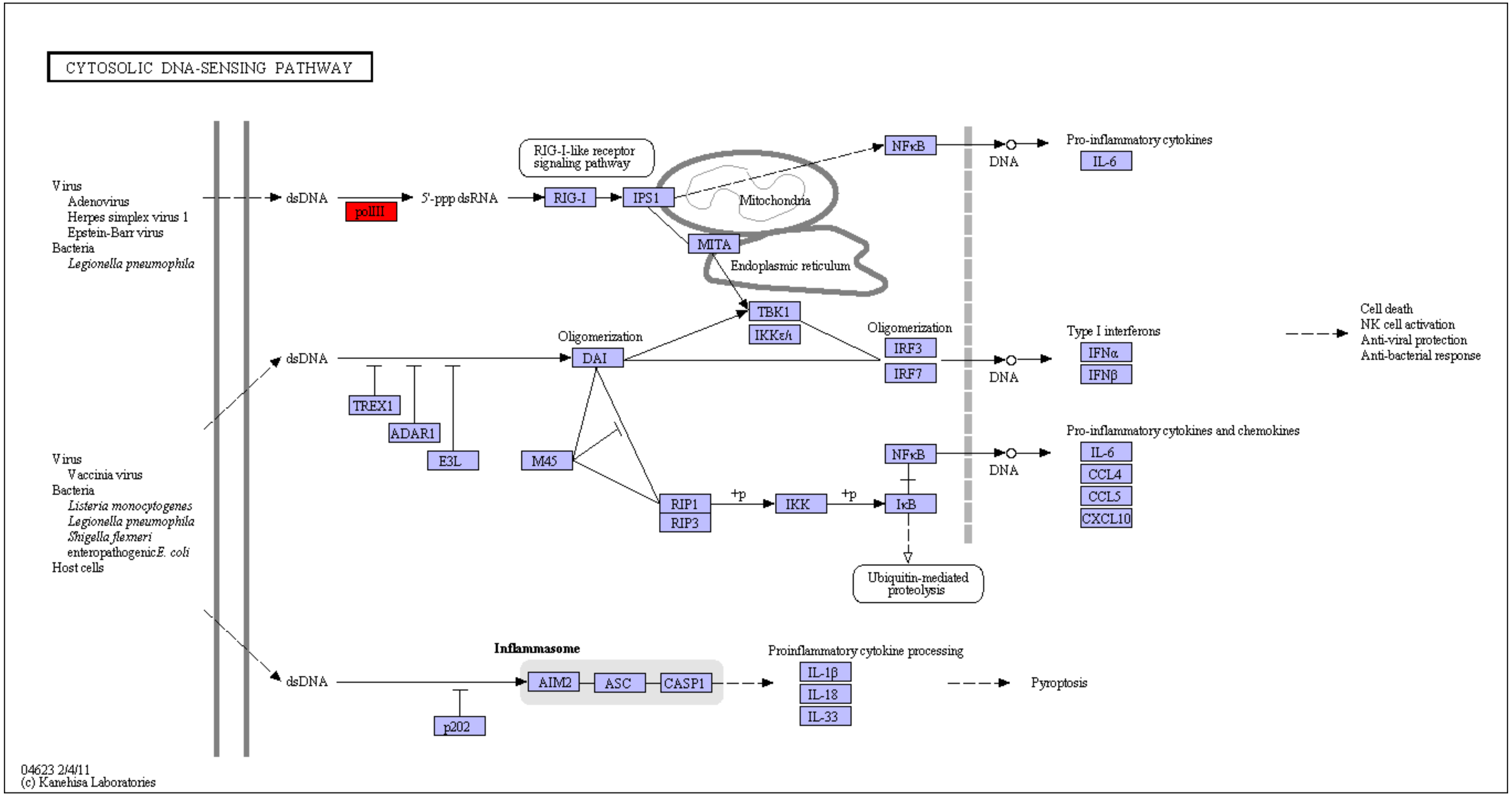

Figure 176: ko04623 Cytosolic DNA-sensing pathway

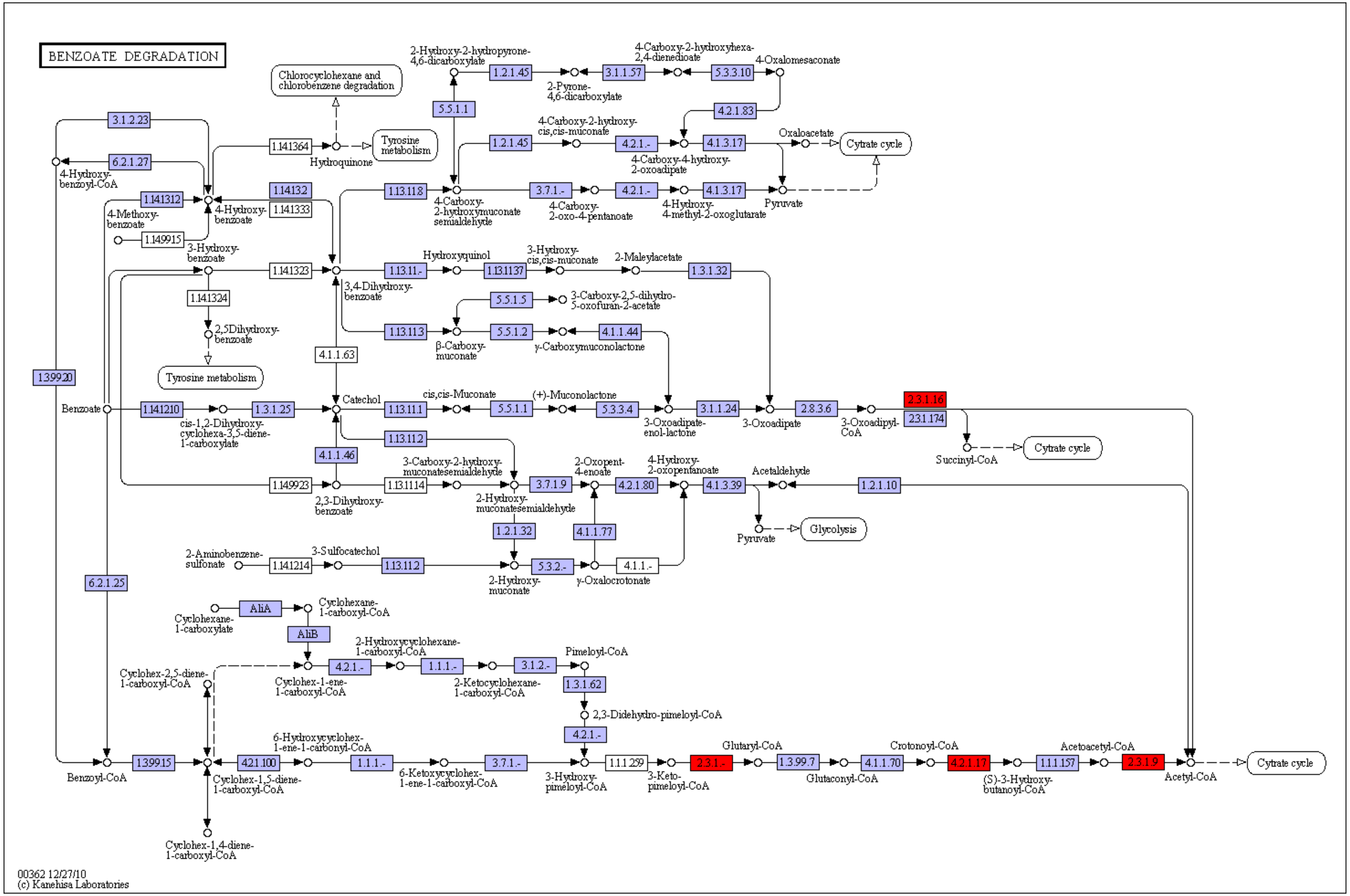

Figure 177: ko00362 Benzoate degradation

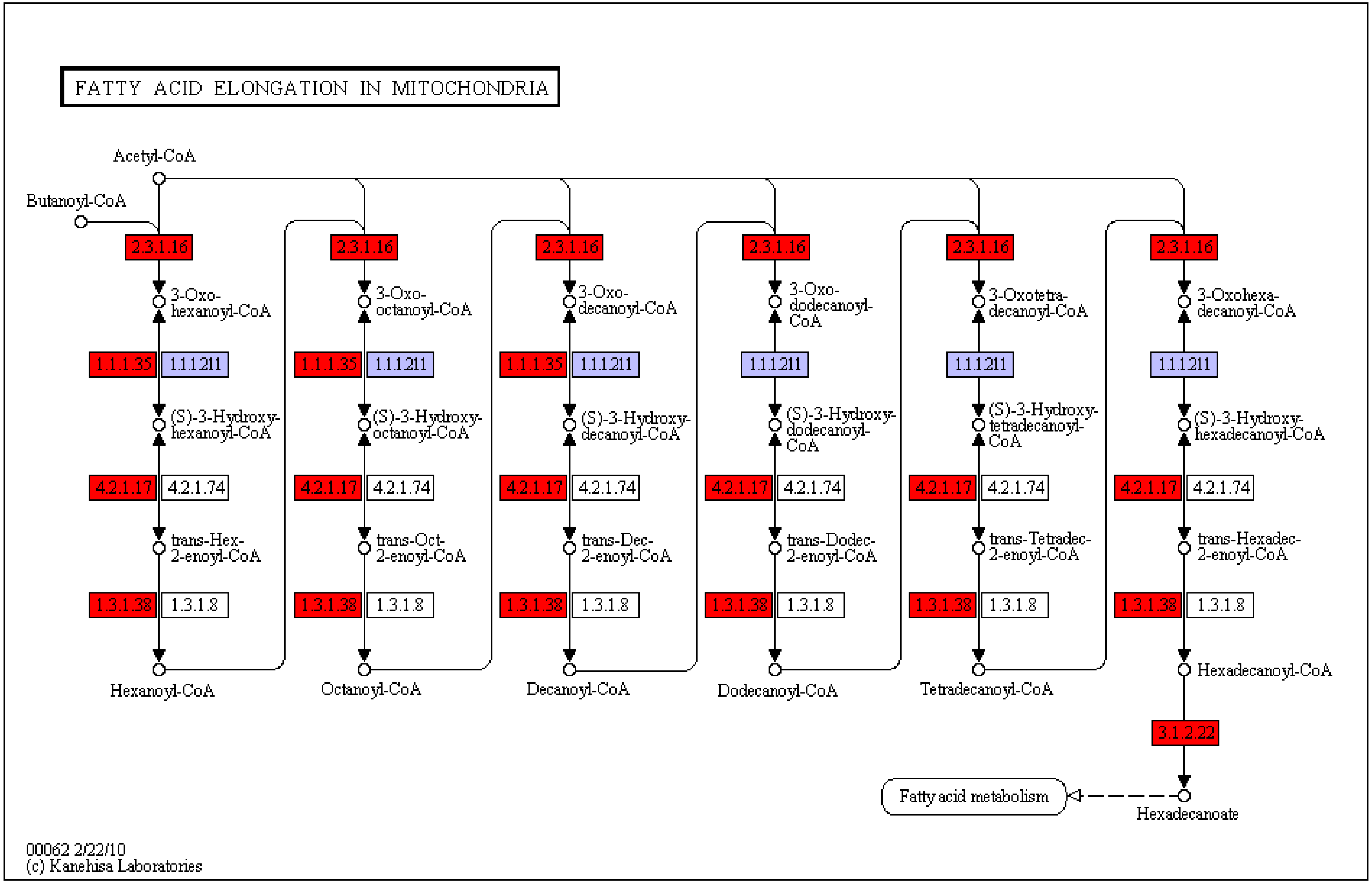

Figure 178: ko00062 Fatty acid elongation in mitochondria

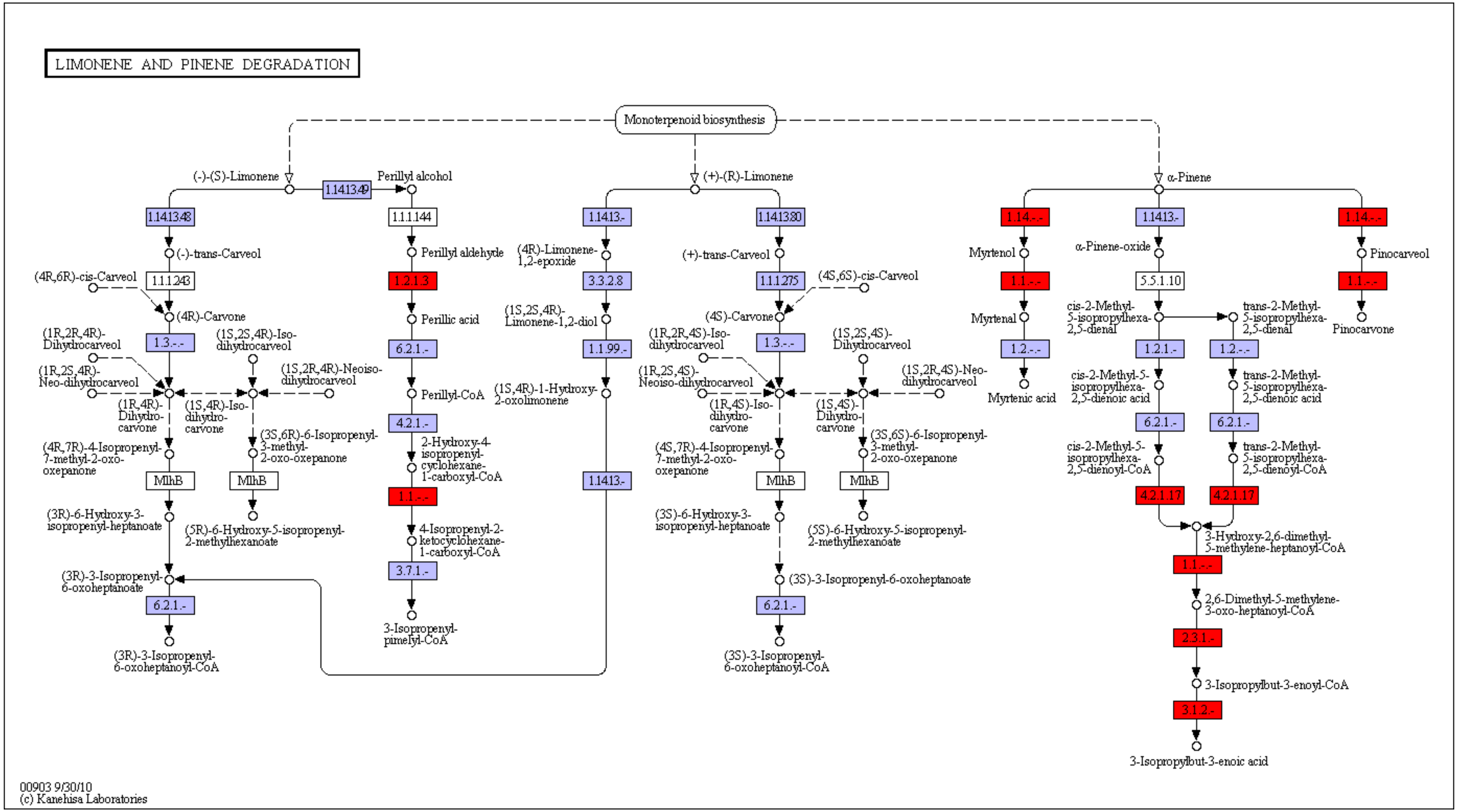

Figure 179: ko00903 Limonene and pinene degradation

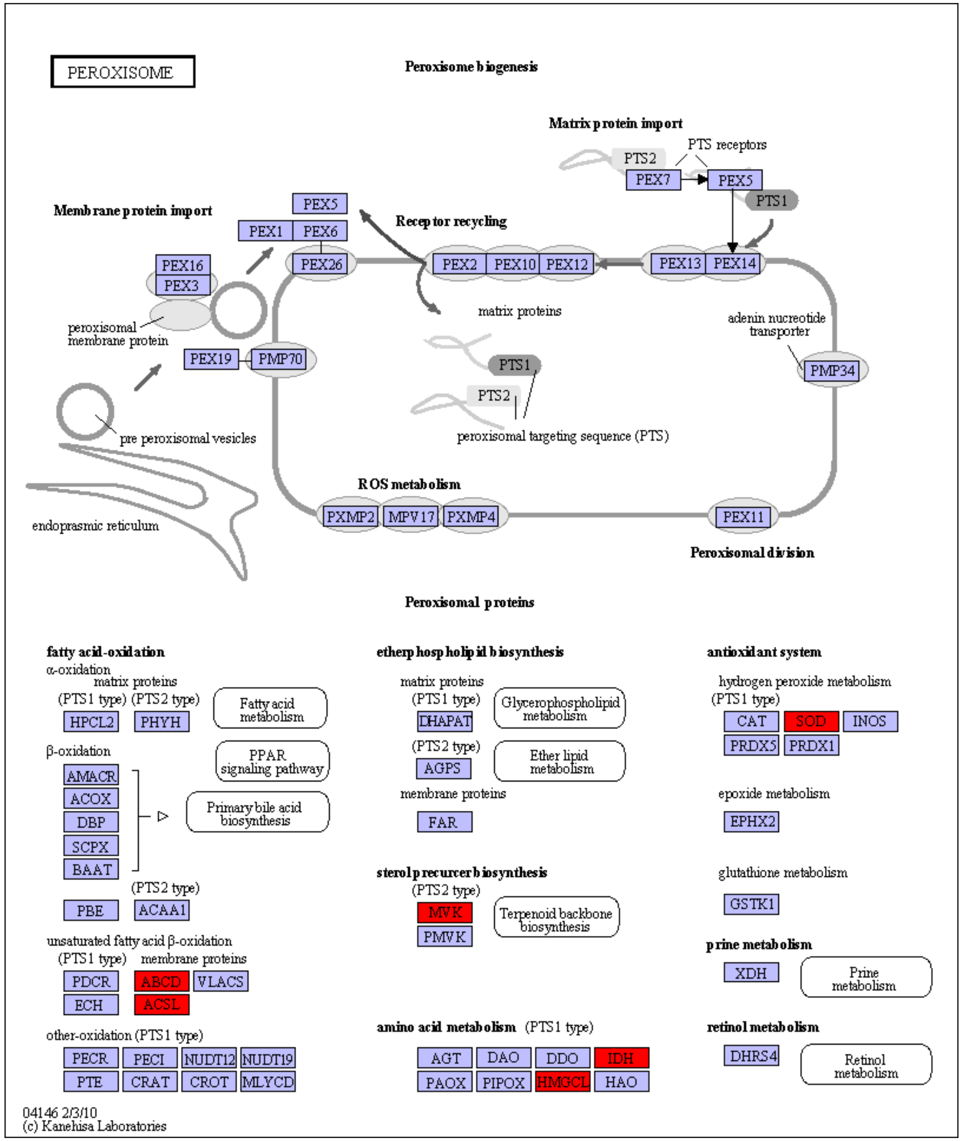

Figure 180: ko04146 Peroxisome

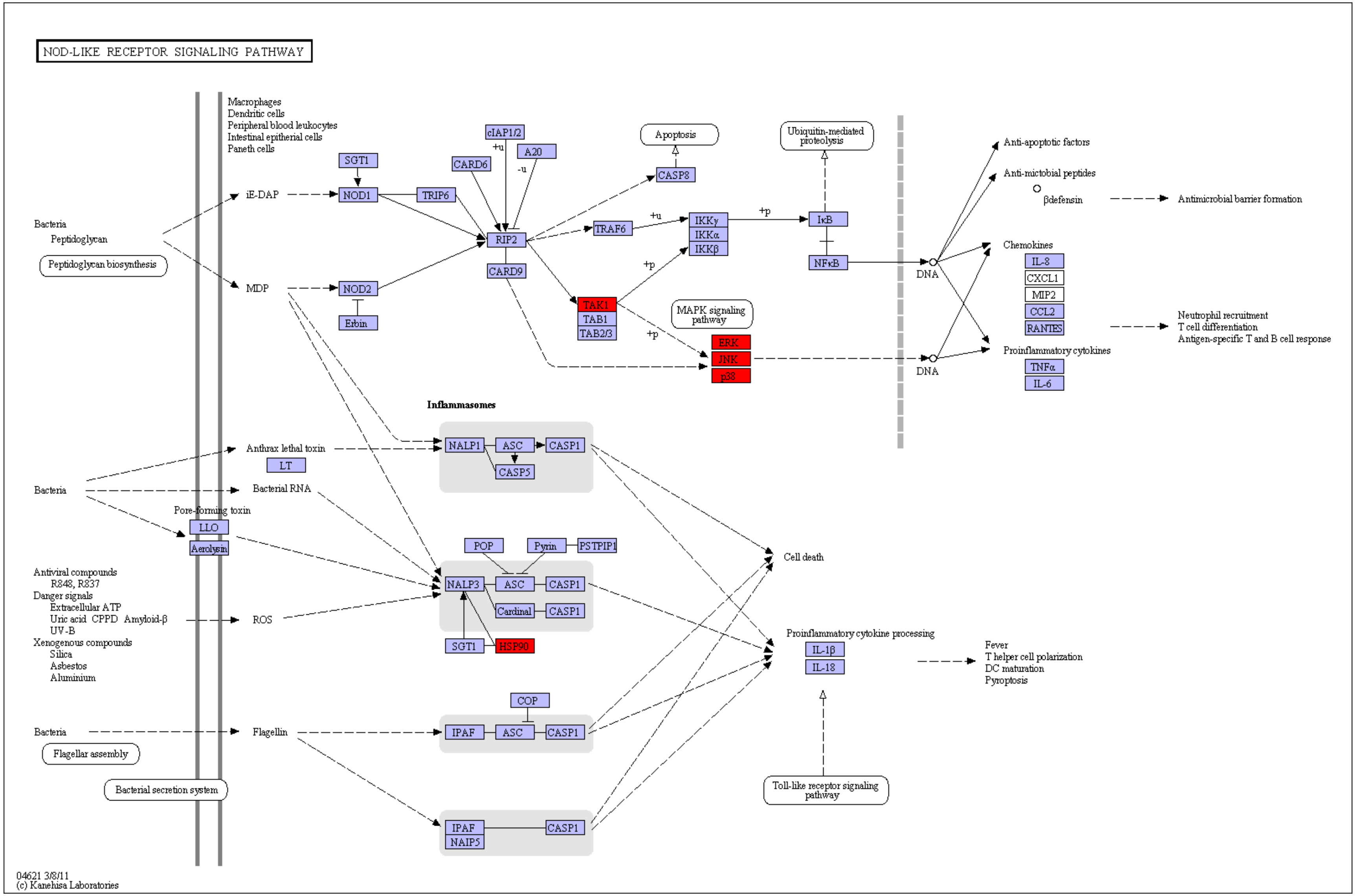

Figure 181: ko04621 NOD-like receptor signaling pathway

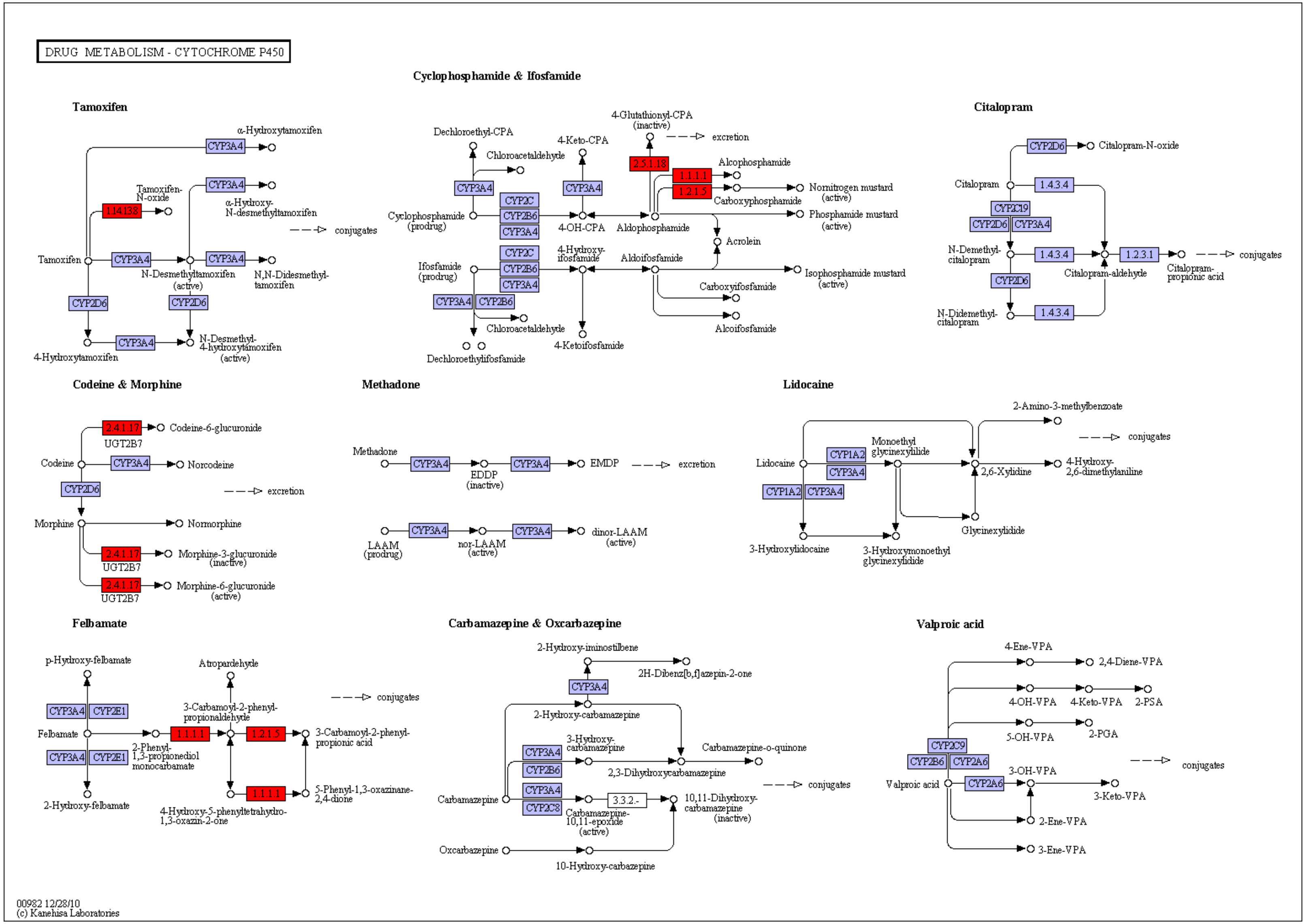

Figure 182: ko00982 Drug metabolism - cytochrome P450

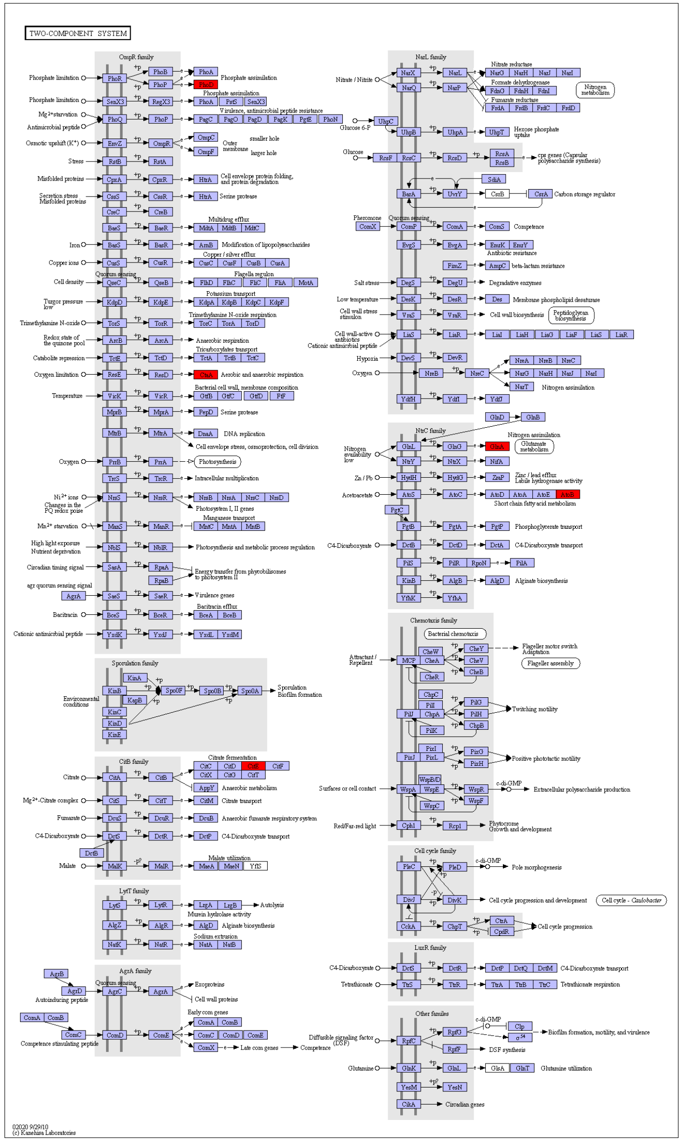

Figure 183: ko02020 Two-component system

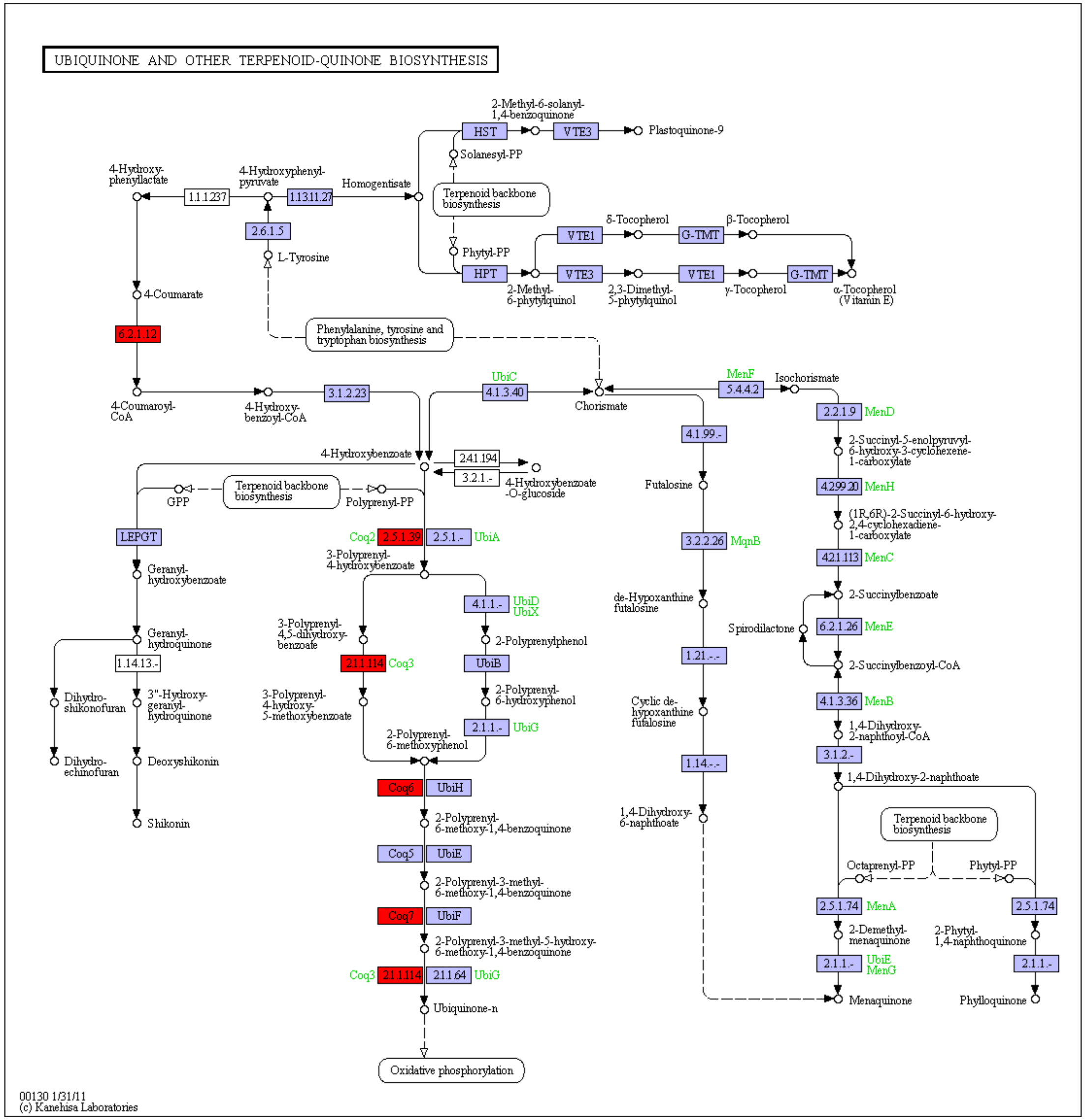

Figure 184: ko00130 Ubiquinone and other terpenoid-quinone biosynthesis

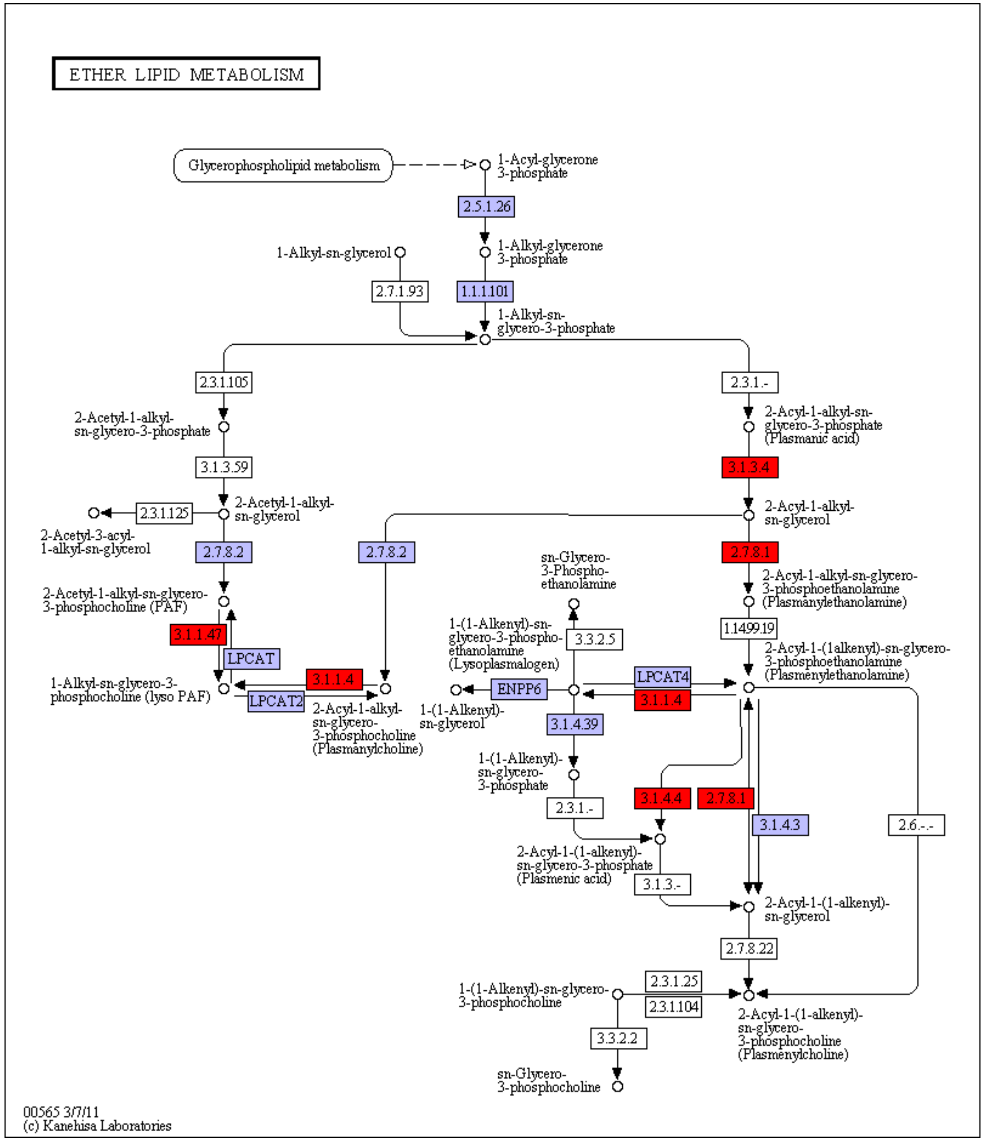

Figure 185: ko00565 Ether lipid metabolism

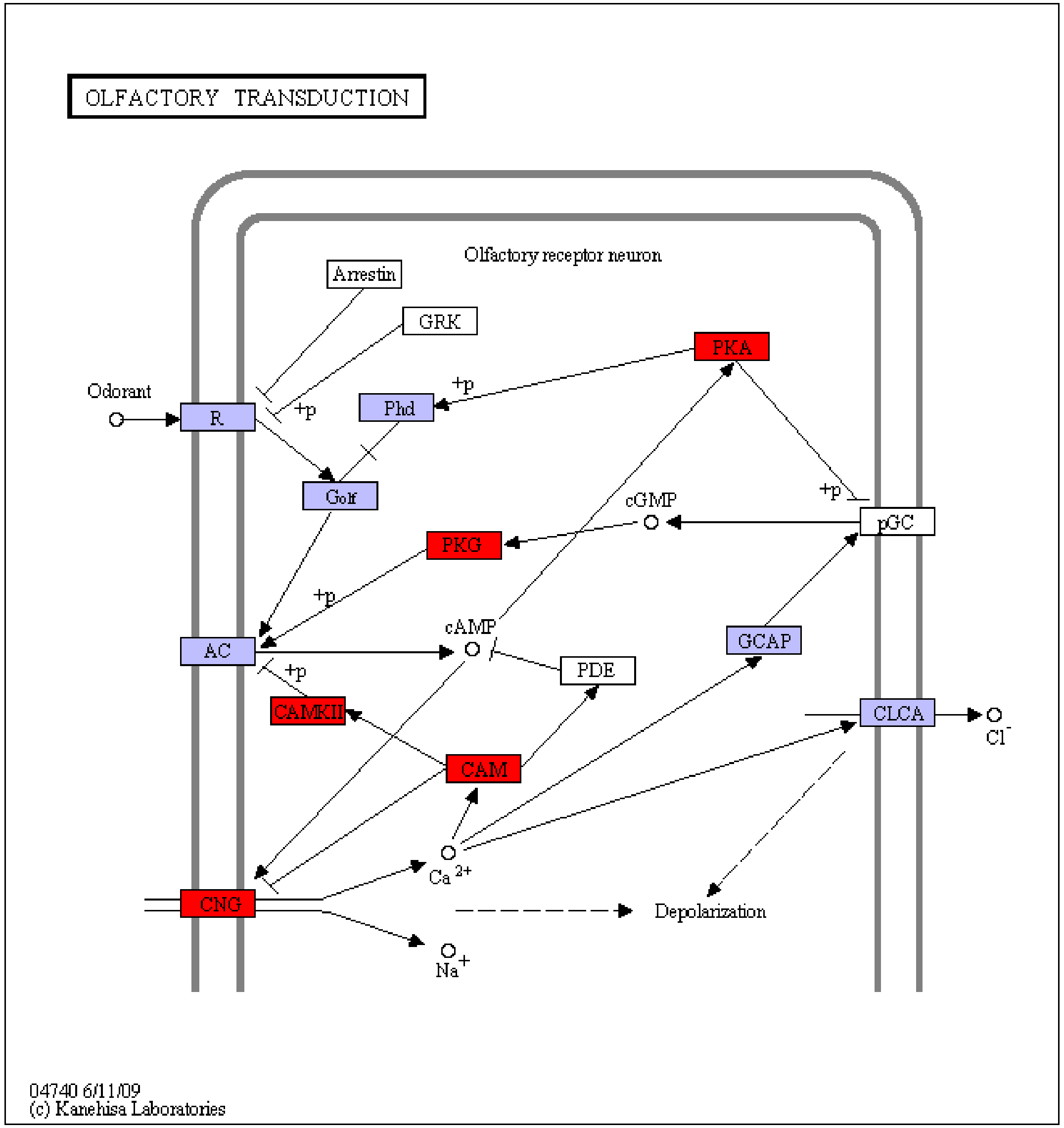

Figure 186: ko04740 Olfactory transduction

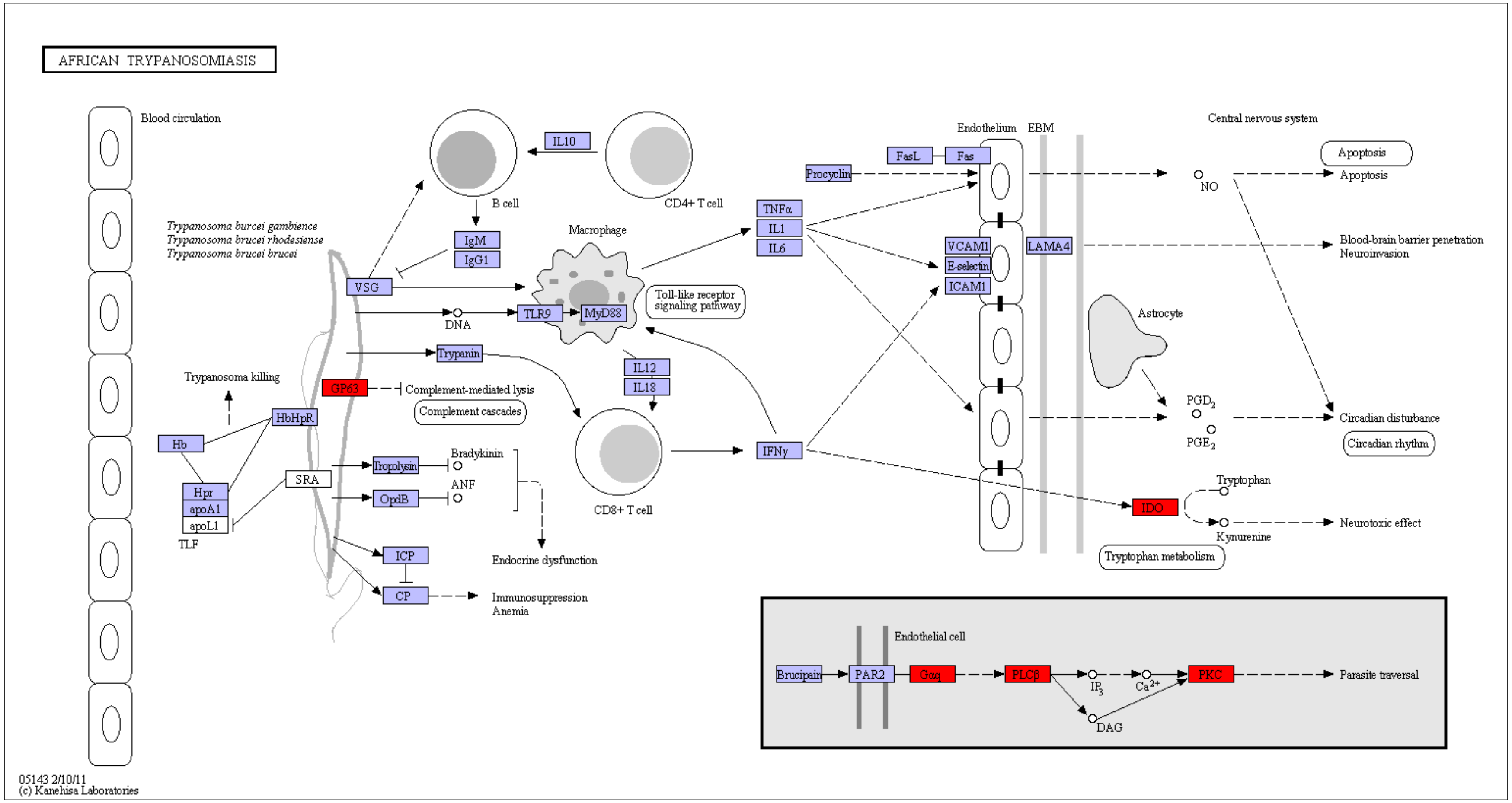

Figure 187: ko05143 African trypanosomiasis

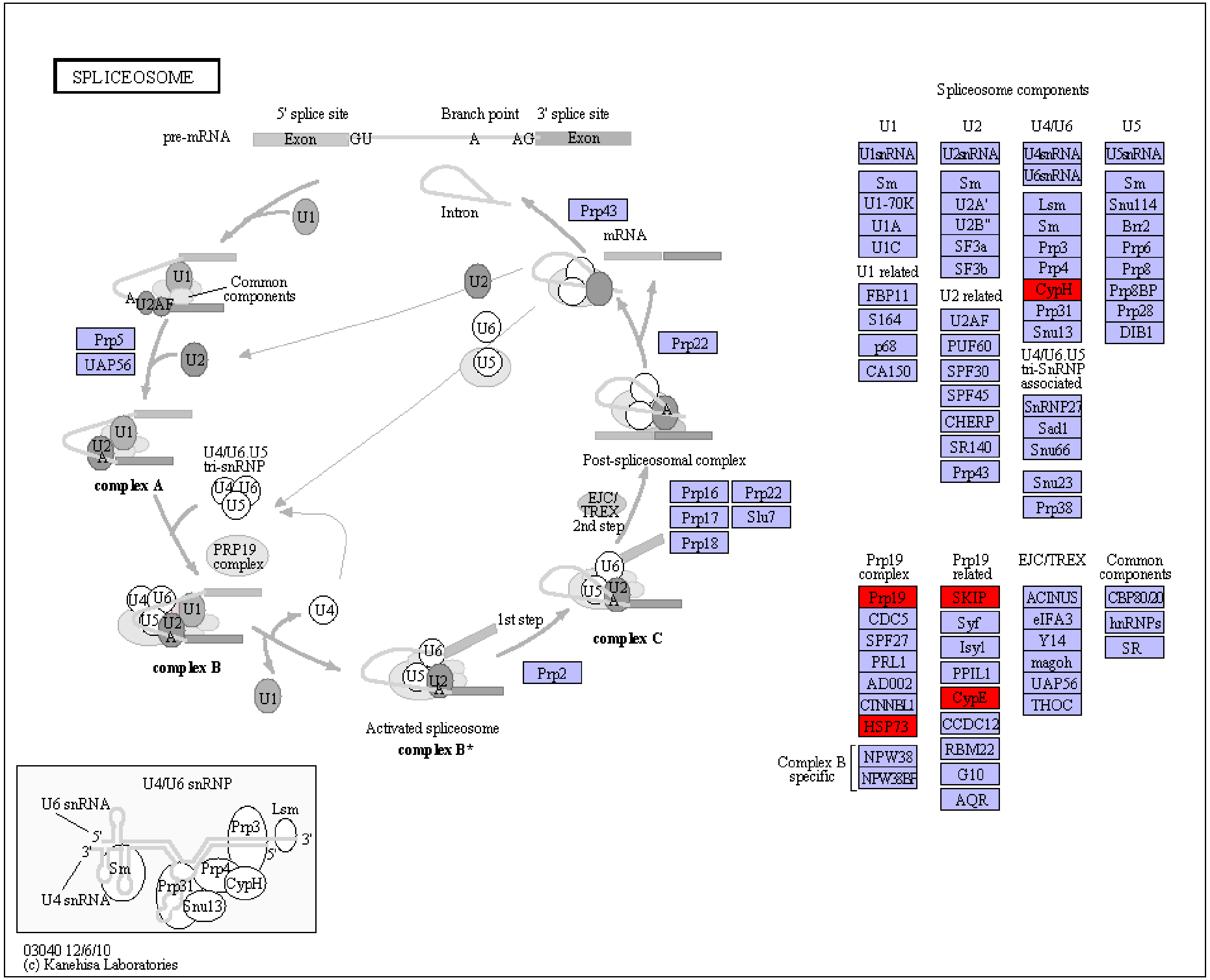

Figure 188: ko03040 Spliceosome

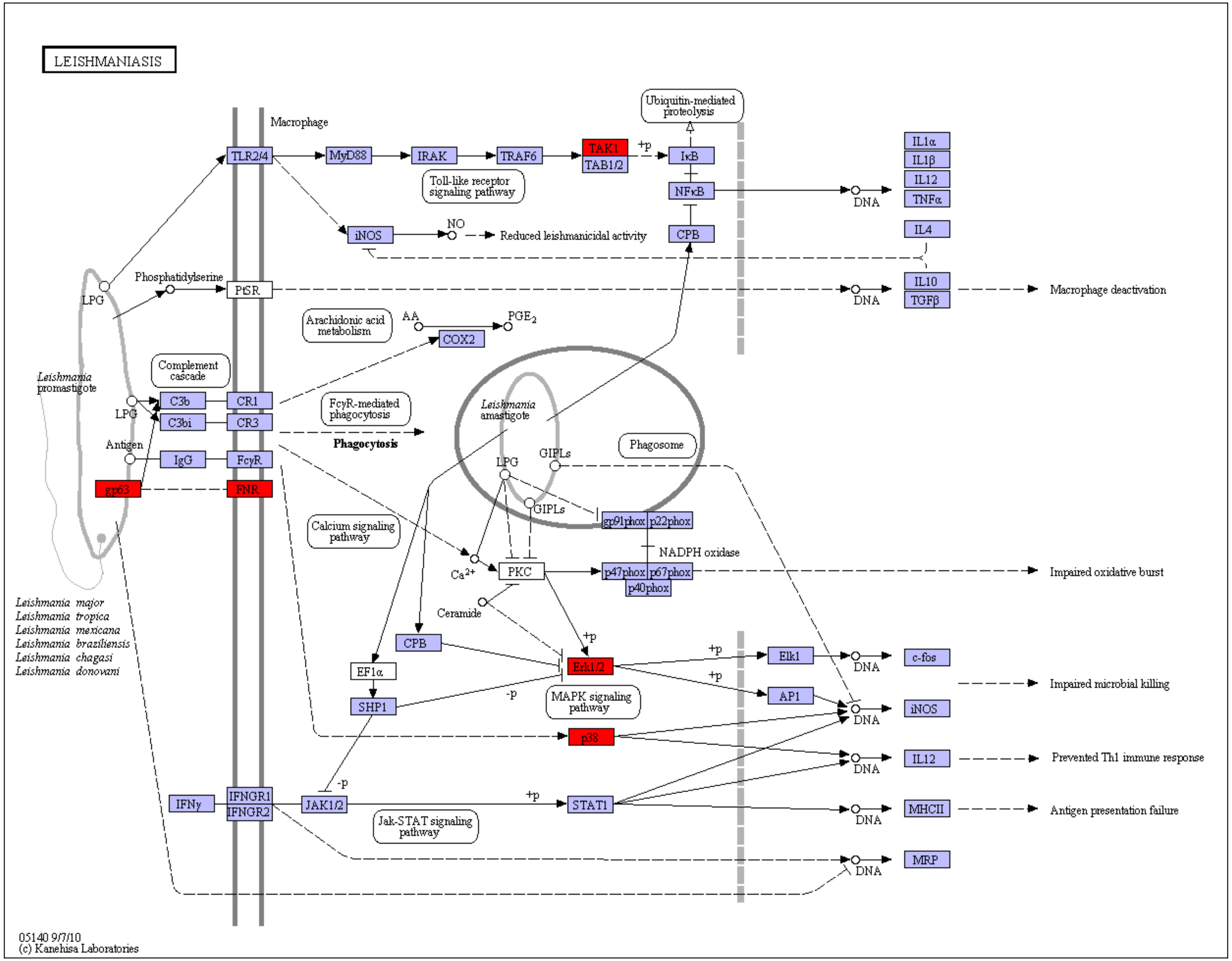

Figure 189: ko05140 Leishmaniasis

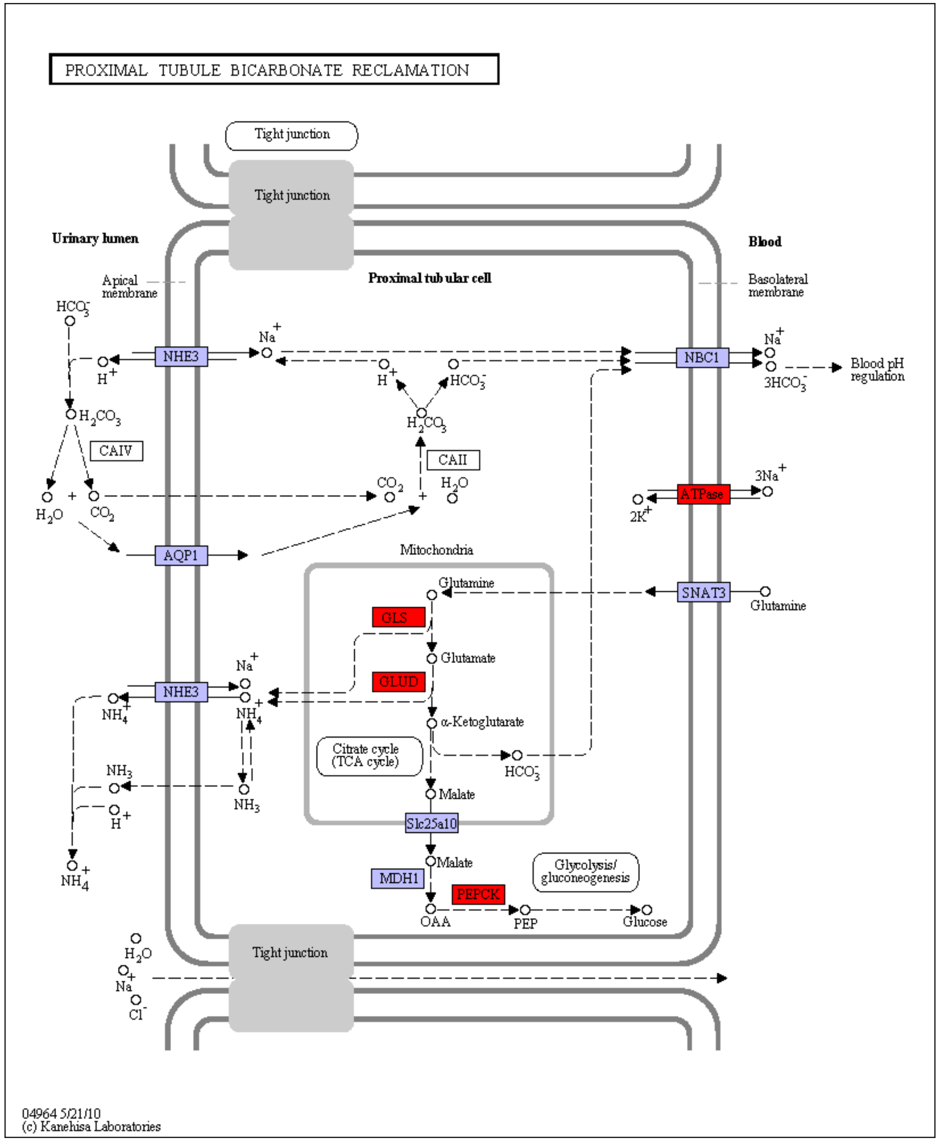

Figure 190: ko04964 Proximal tubule bicarbonate reclamation

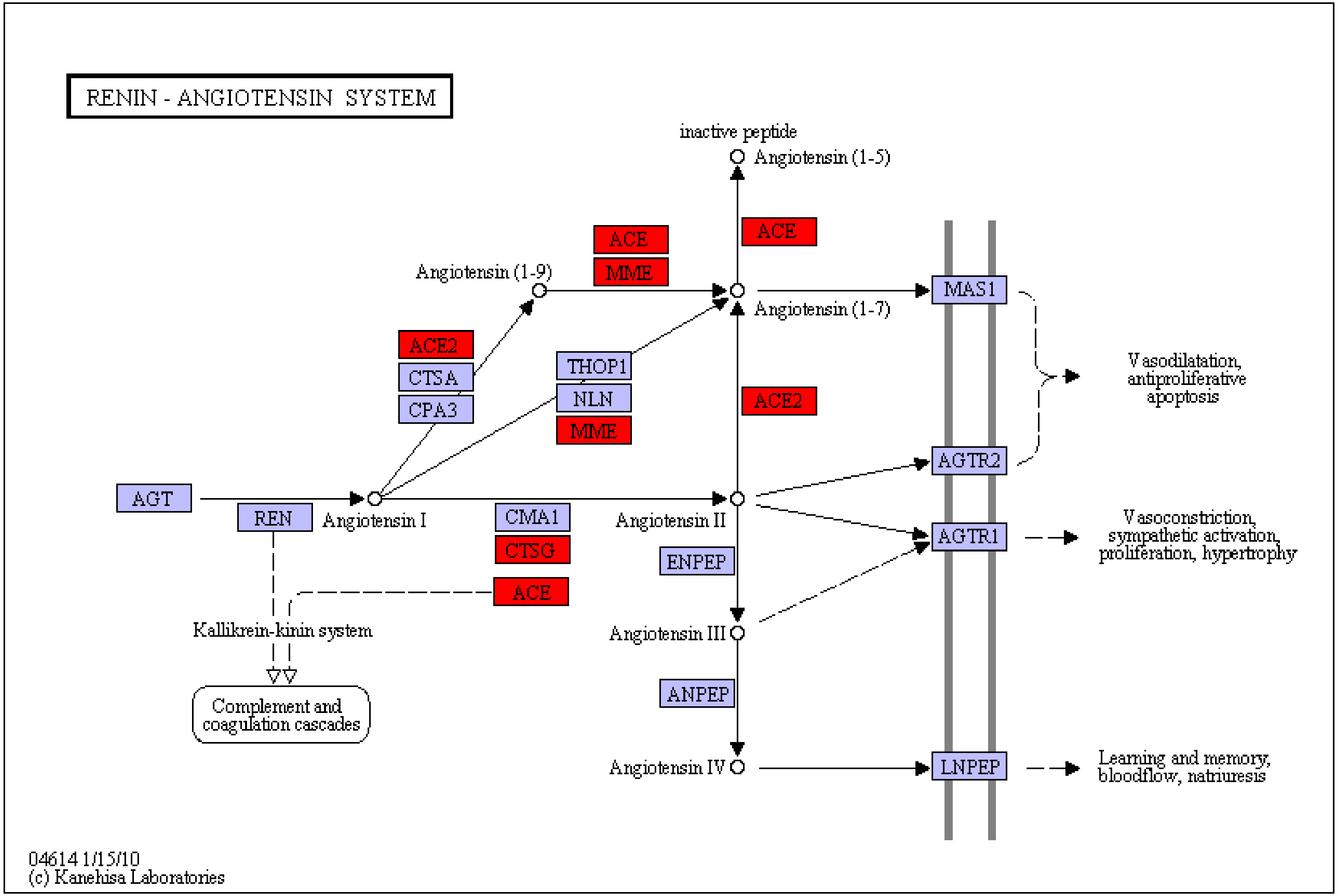

Figure 191: ko04614 Renin-angiotensin system

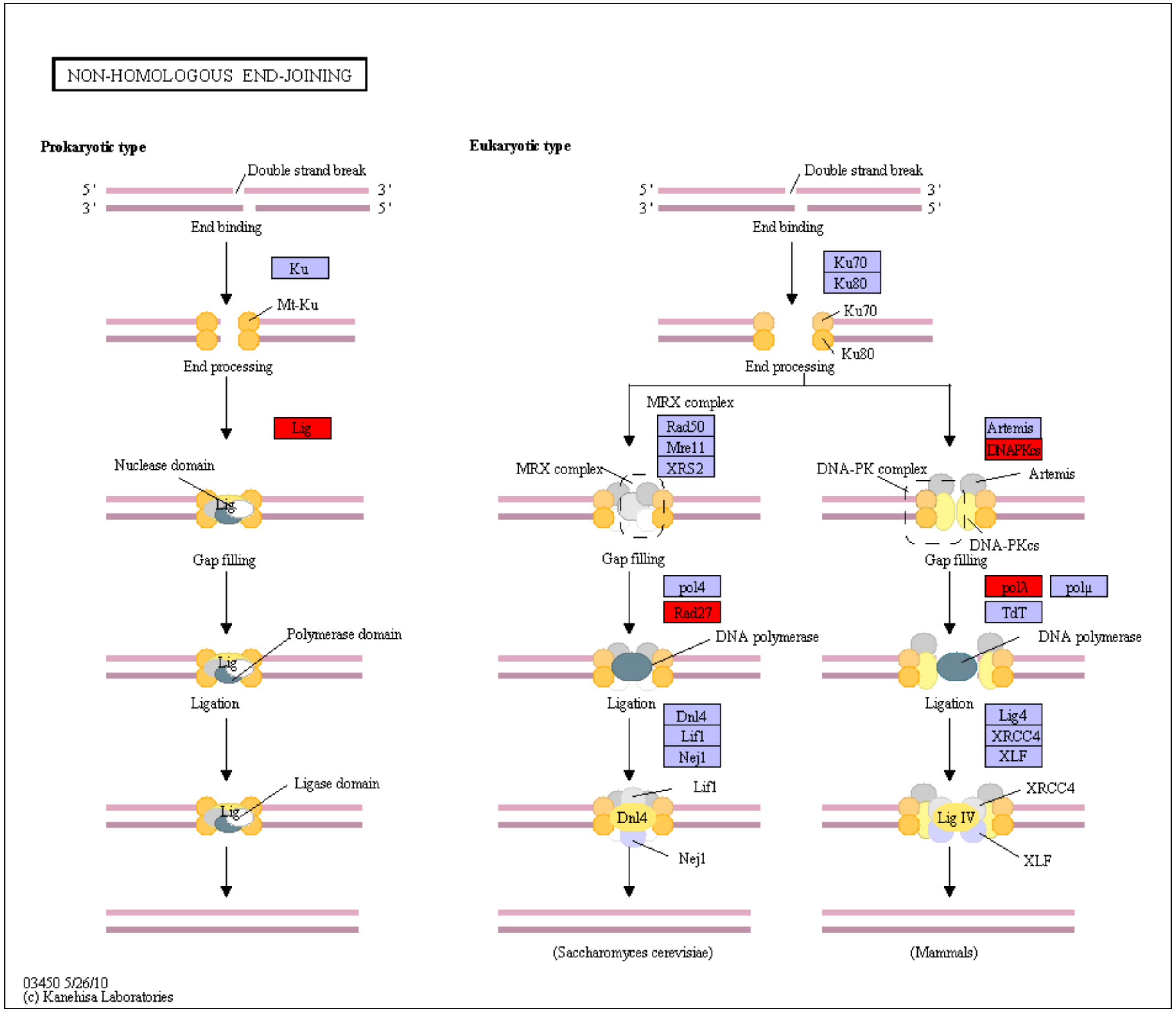

Figure 192: ko03450 Non-homologous end-joining

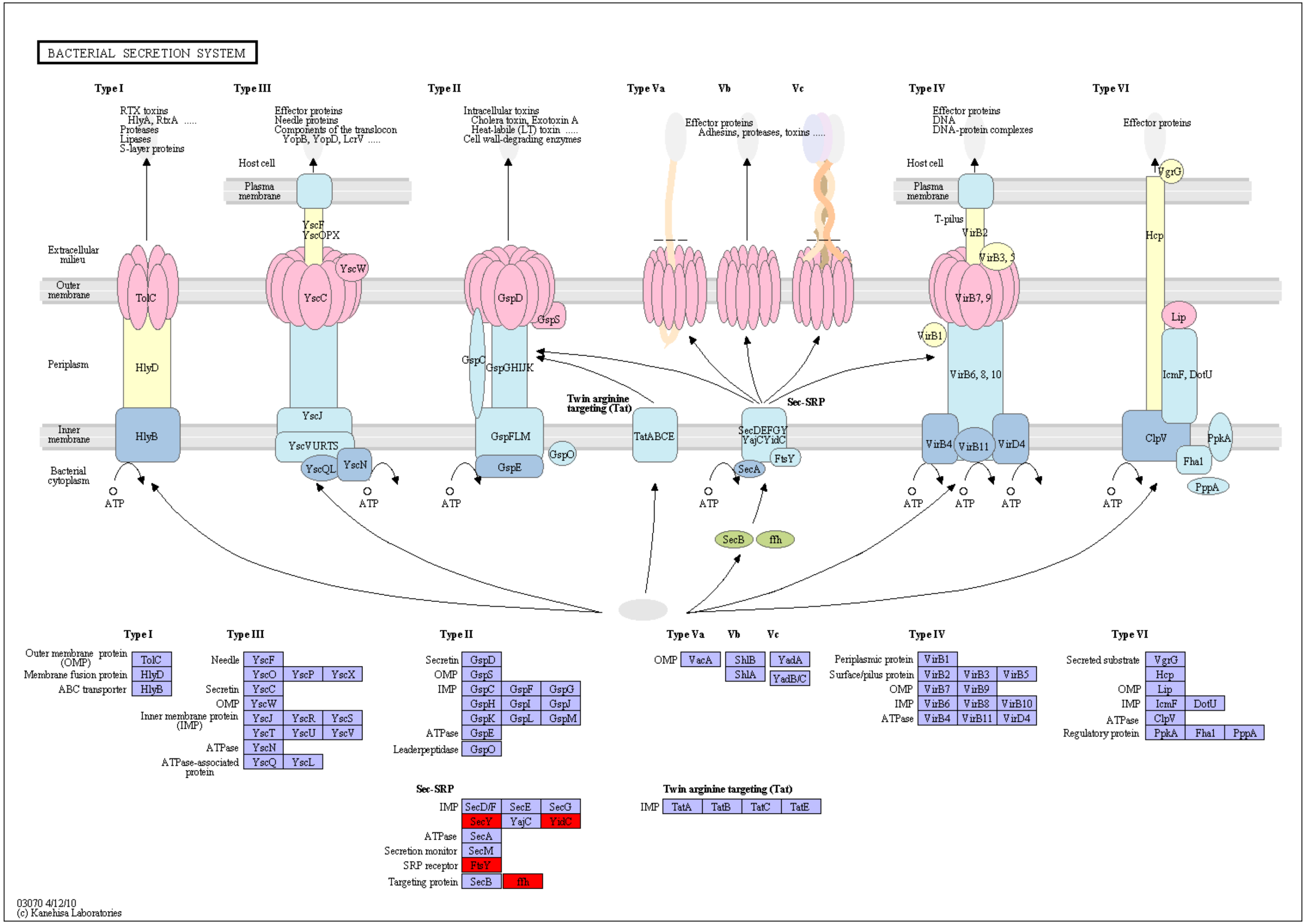

Figure 193: ko03070 Bacterial secretion system

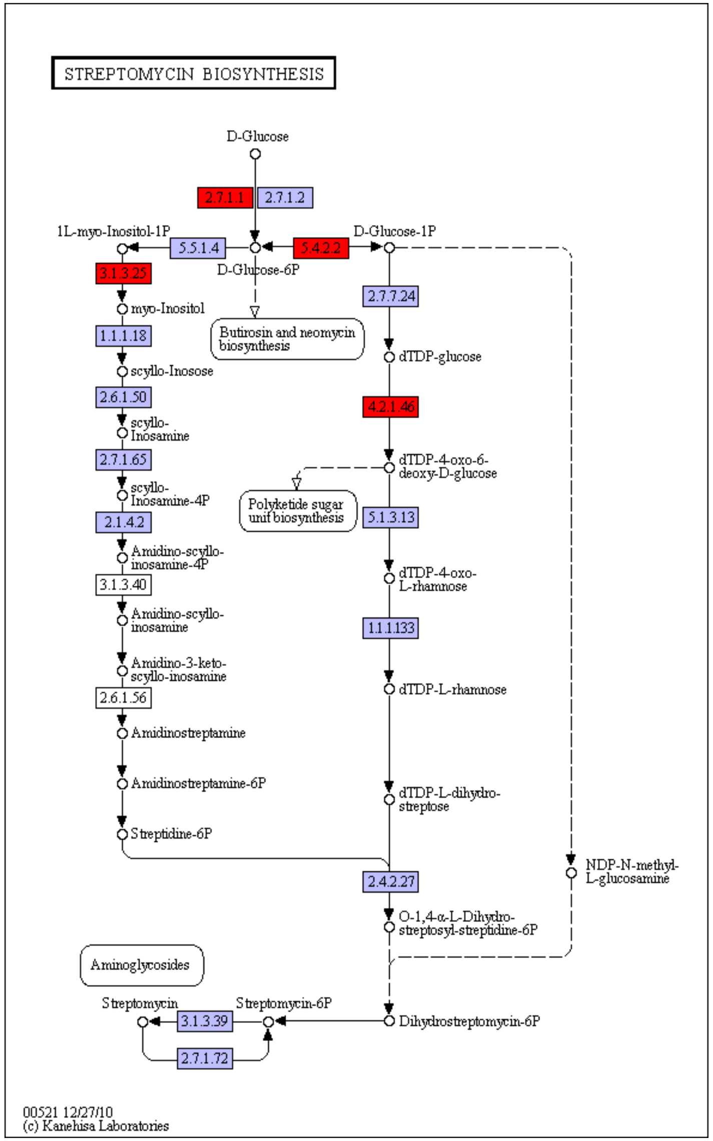

Figure 194: ko00521 Streptomycin biosynthesis

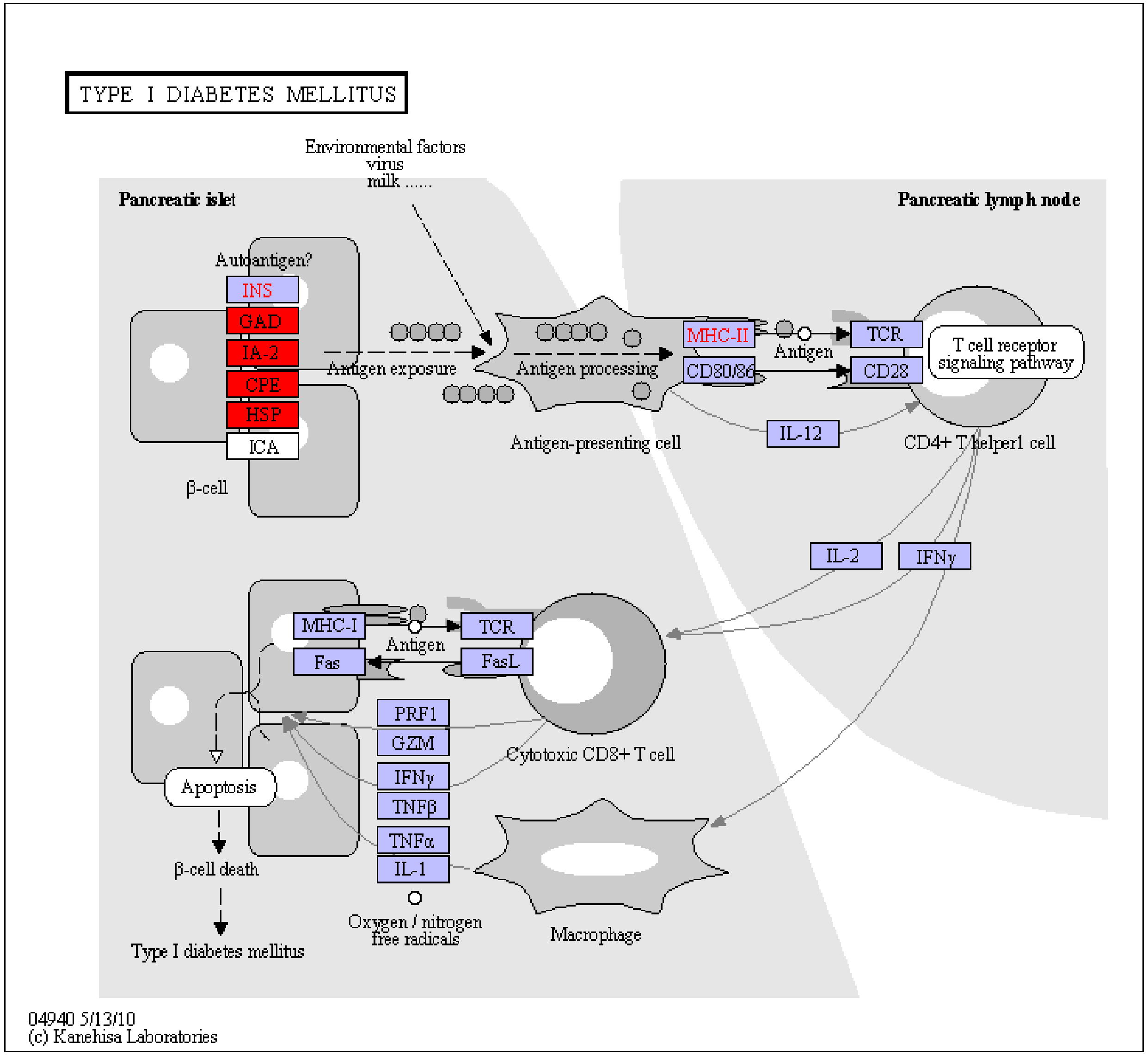

Figure 195: ko04940 Type I diabetes mellitus

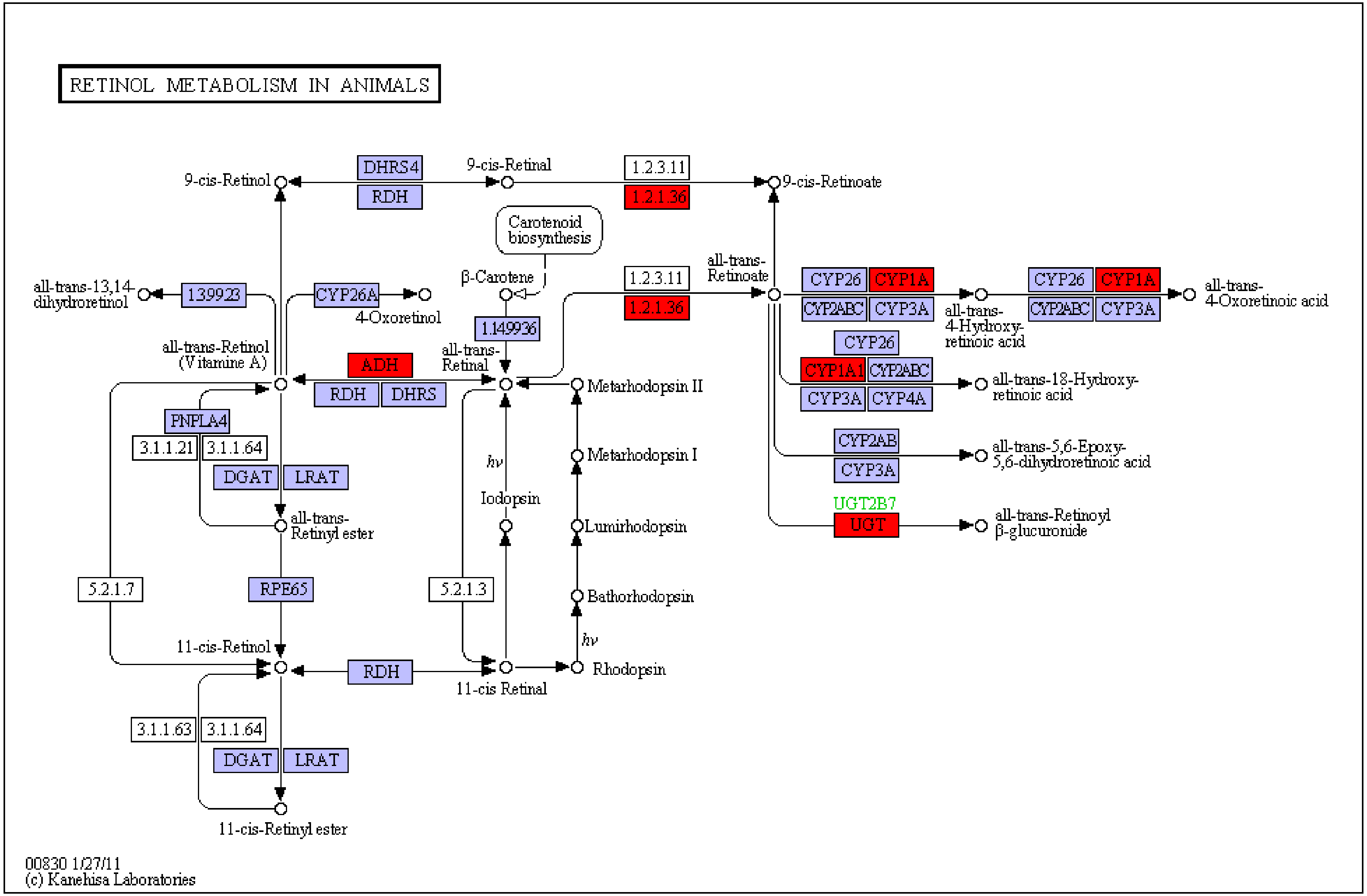

Figure 196: ko00830 Retinol metabolism

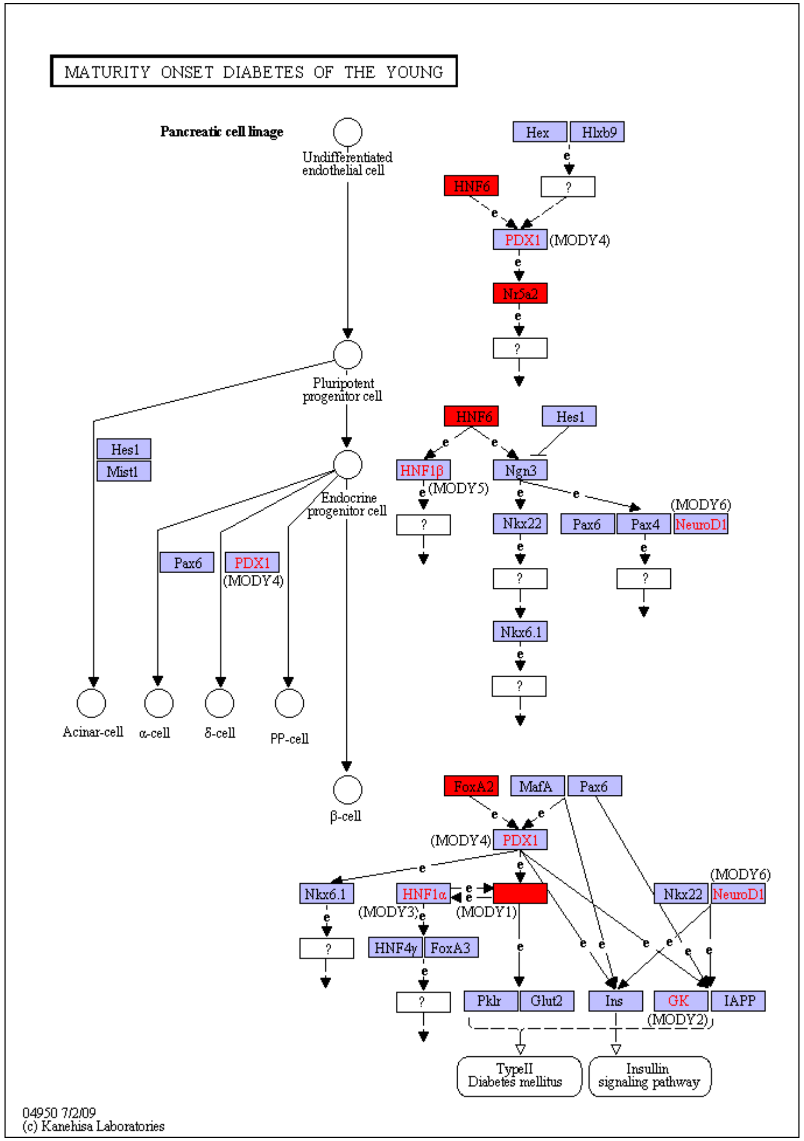

Figure 197: ko04950 Maturity onset diabetes of the young

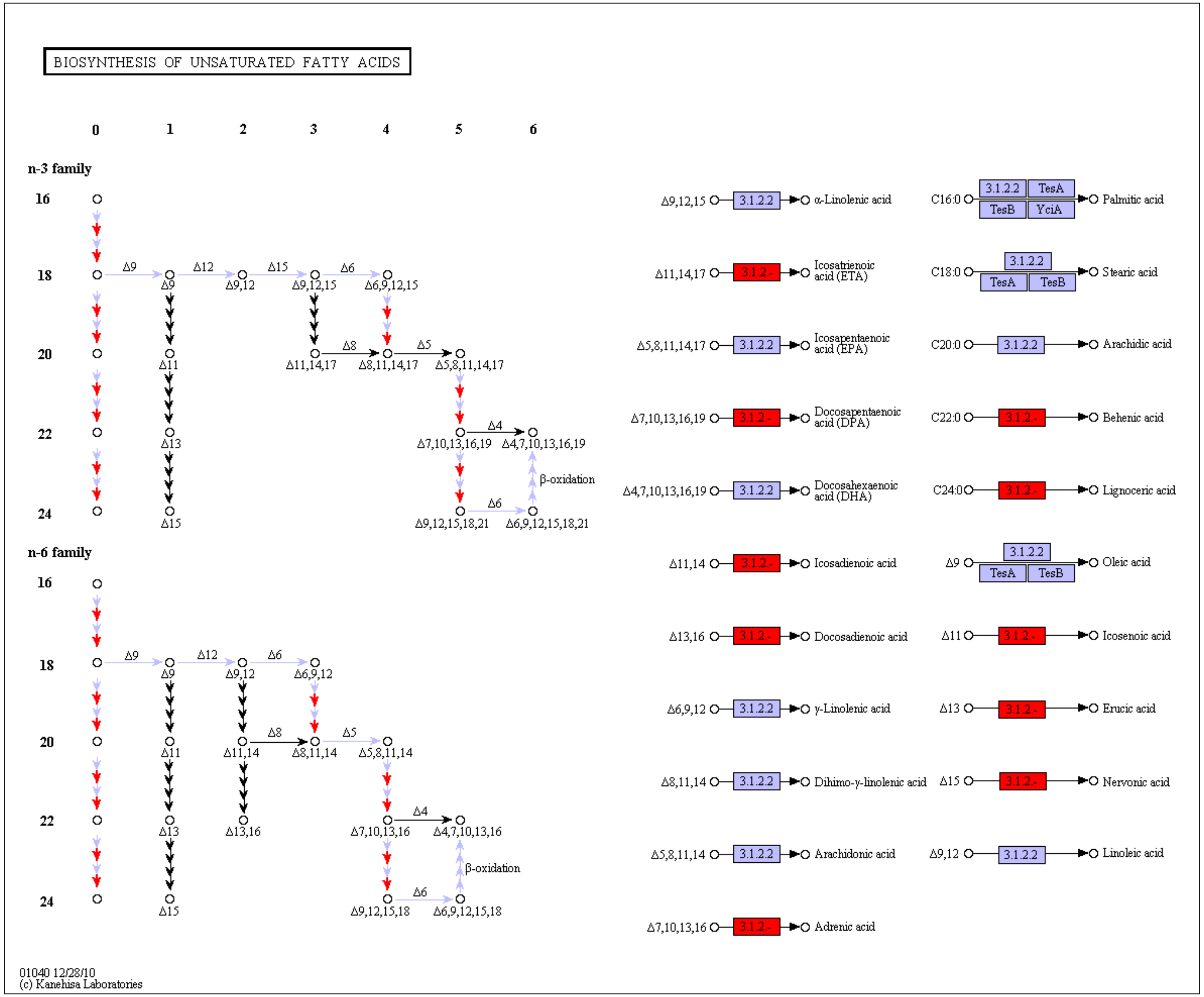

Figure 198: ko01040 Biosynthesis of unsaturated fatty acids

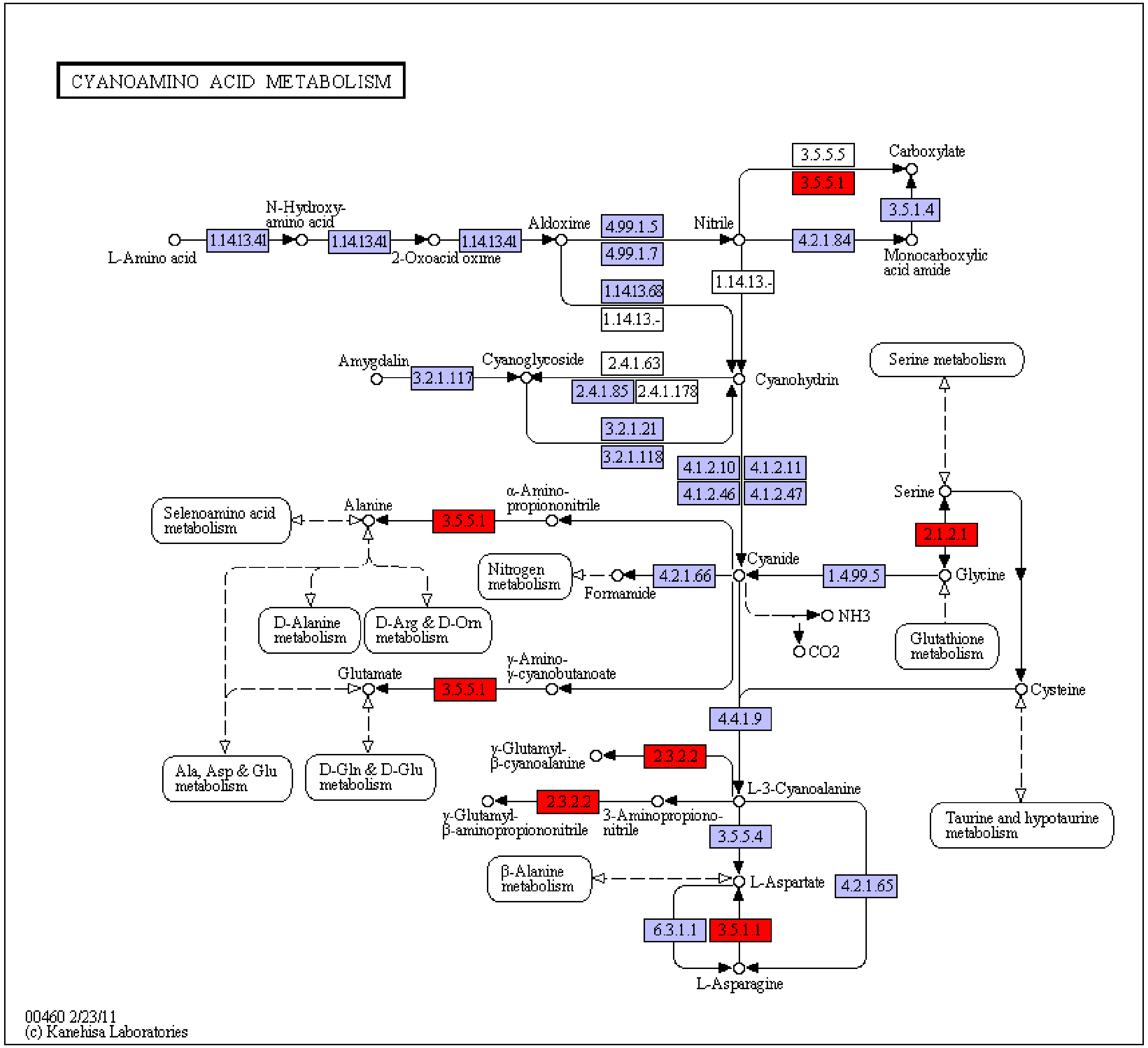

Figure 199: ko00460 Cyanoamino acid metabolism

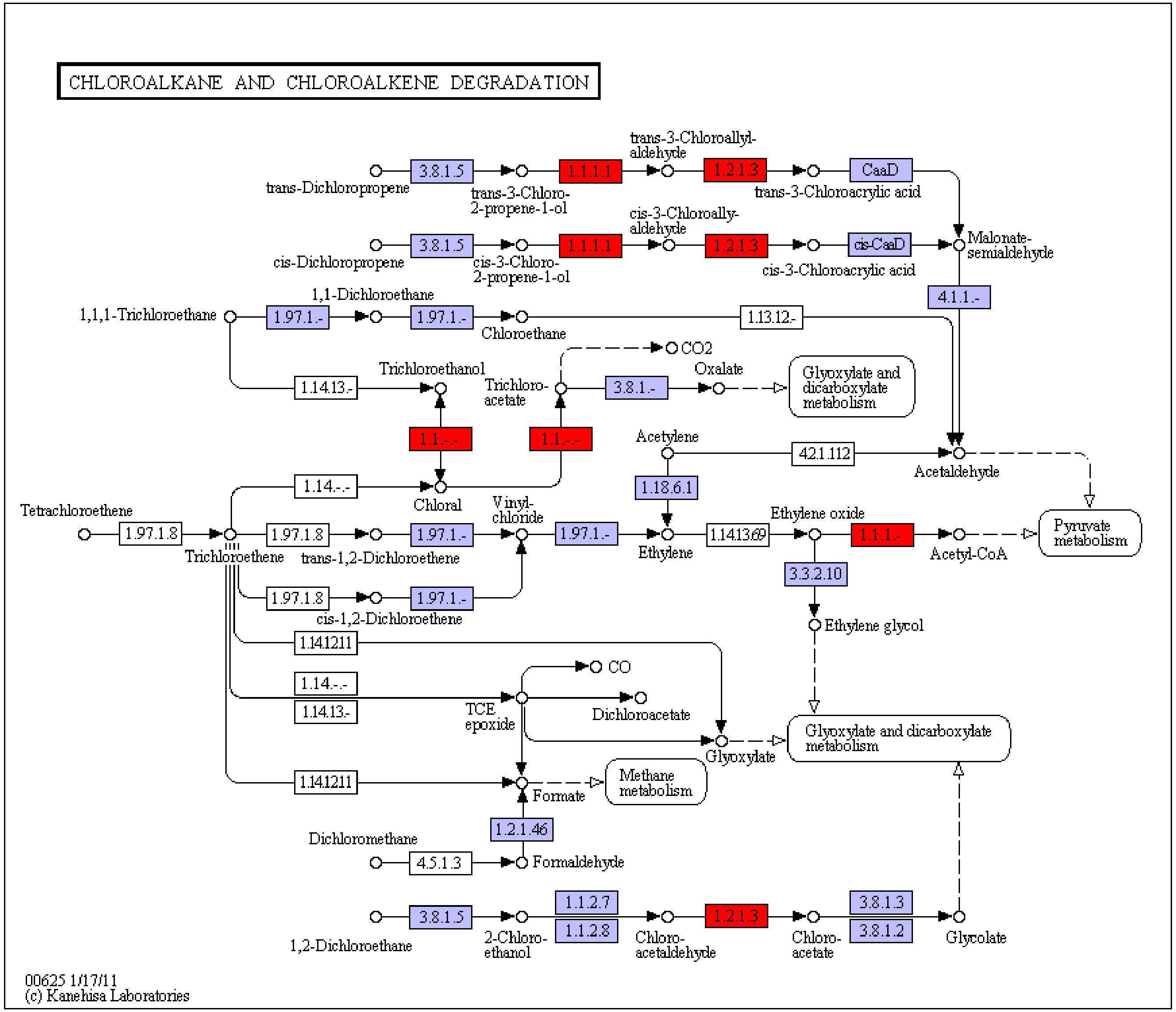

Figure 200: ko00625 Chloroalkane and chloroalkene degradation

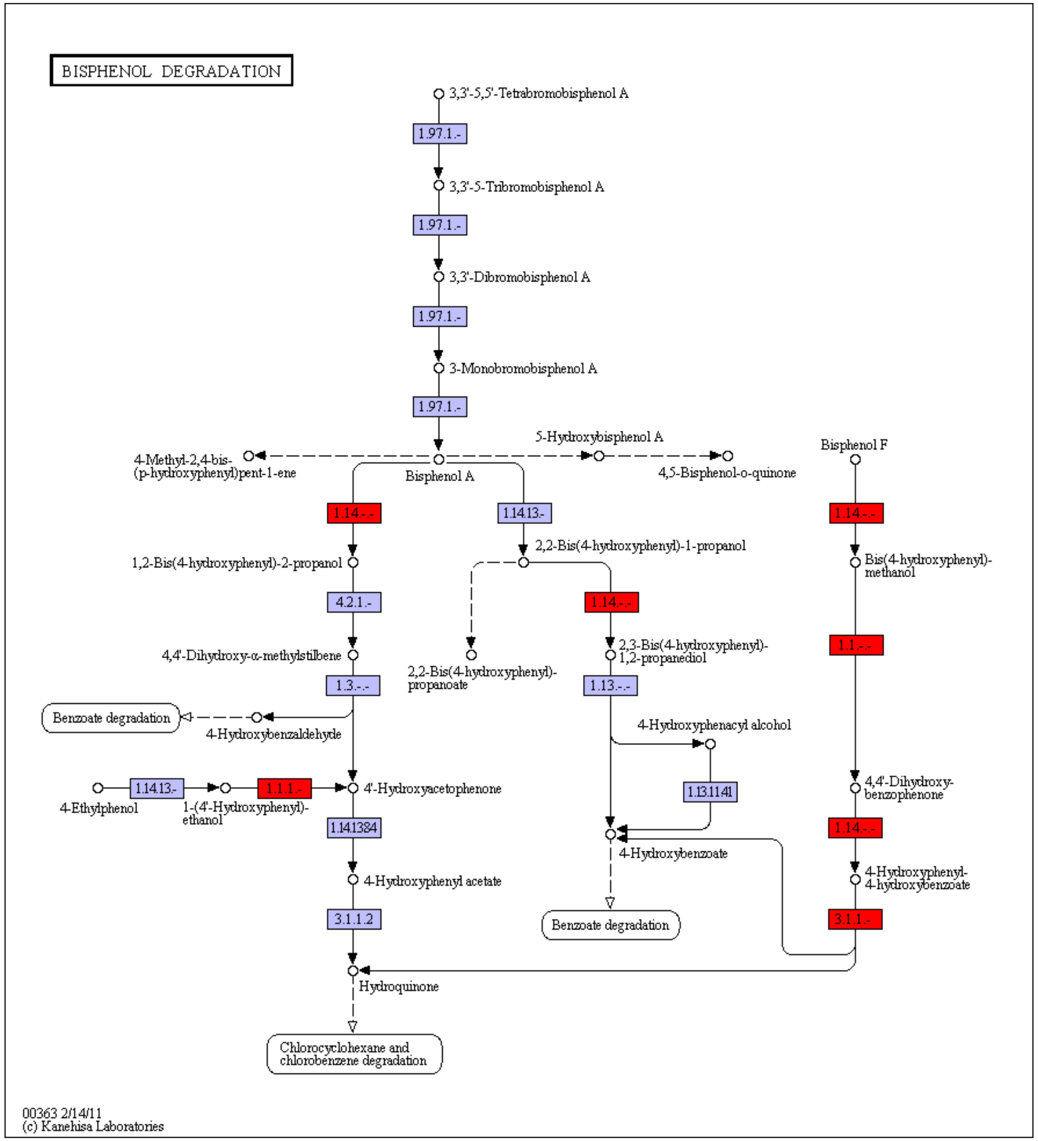

Figure 201: ko00363 Bisphenol degradation

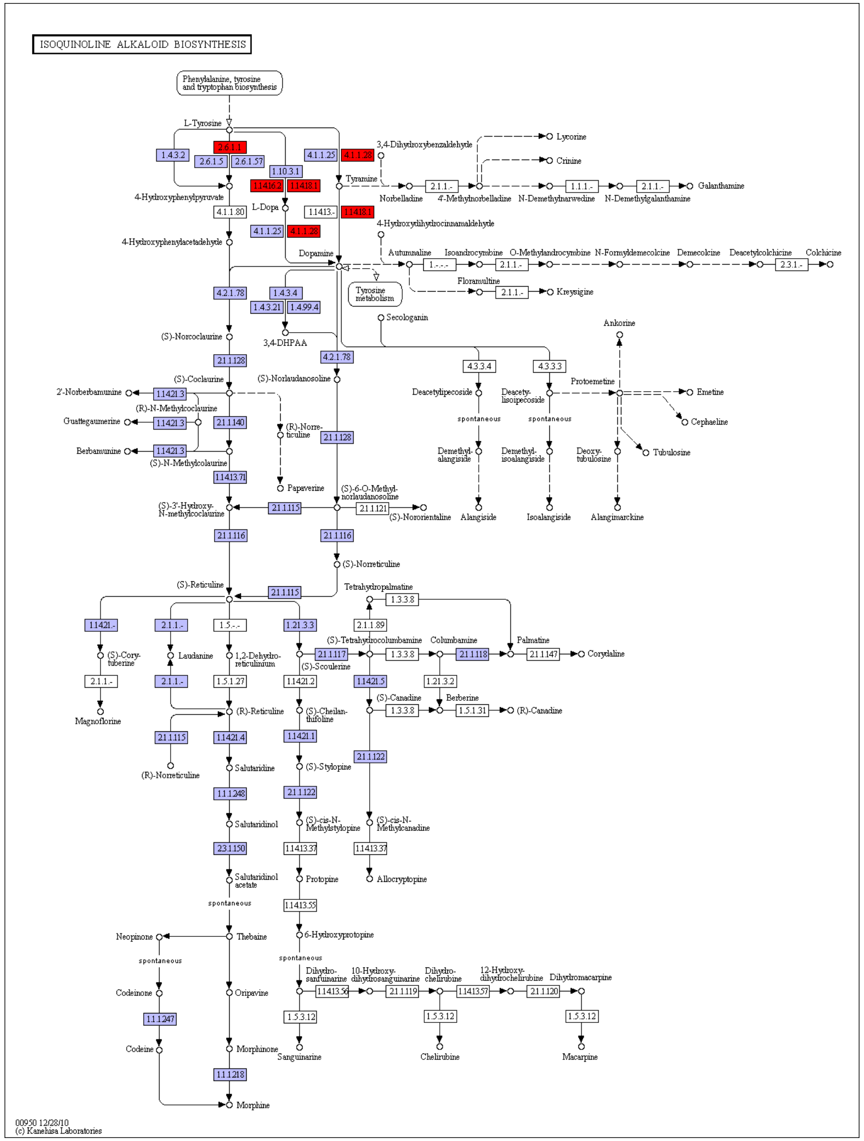

Figure 202: ko00950 Isoquinoline alkaloid biosynthesis

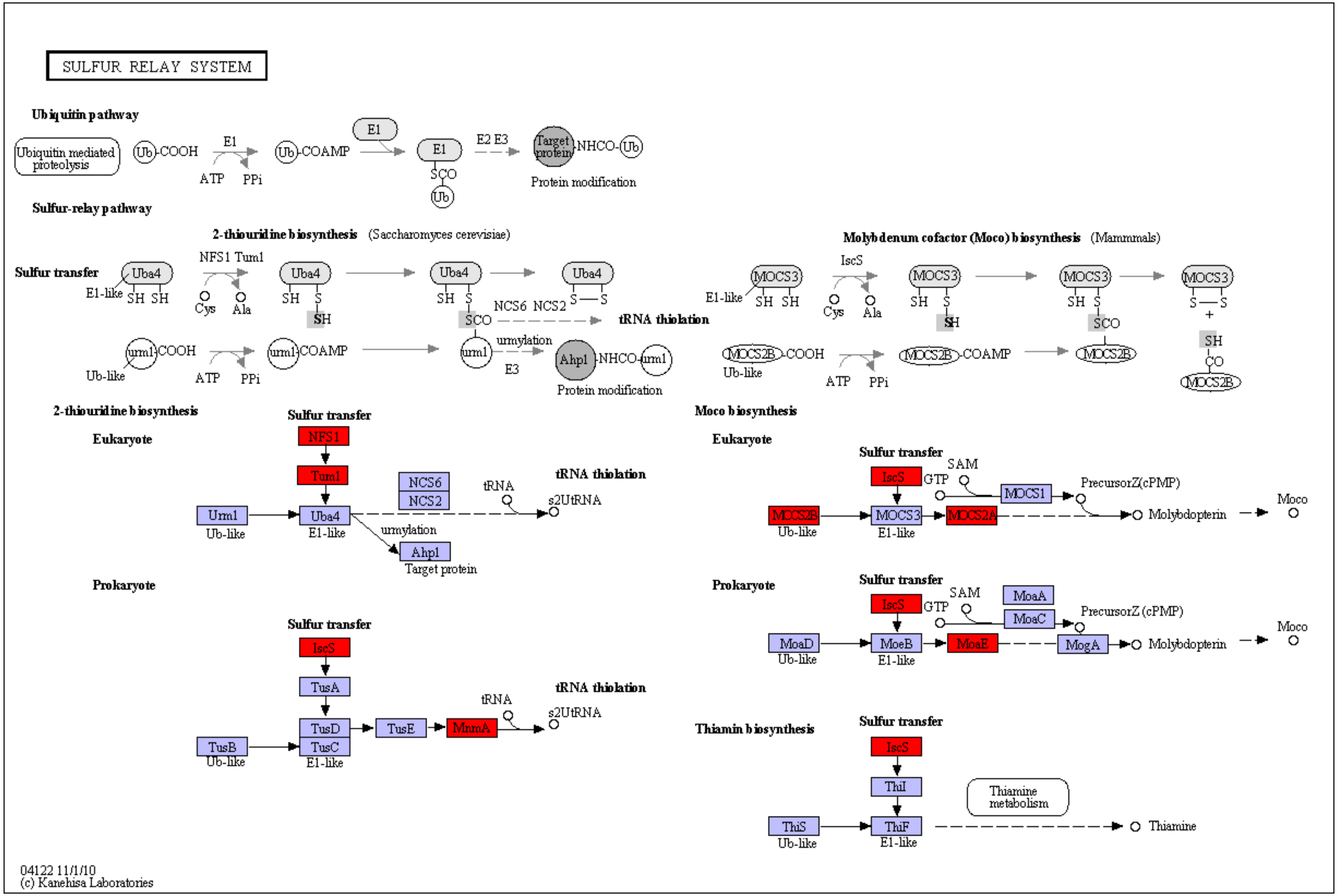

Figure 203: ko04122 Sulfur relay system

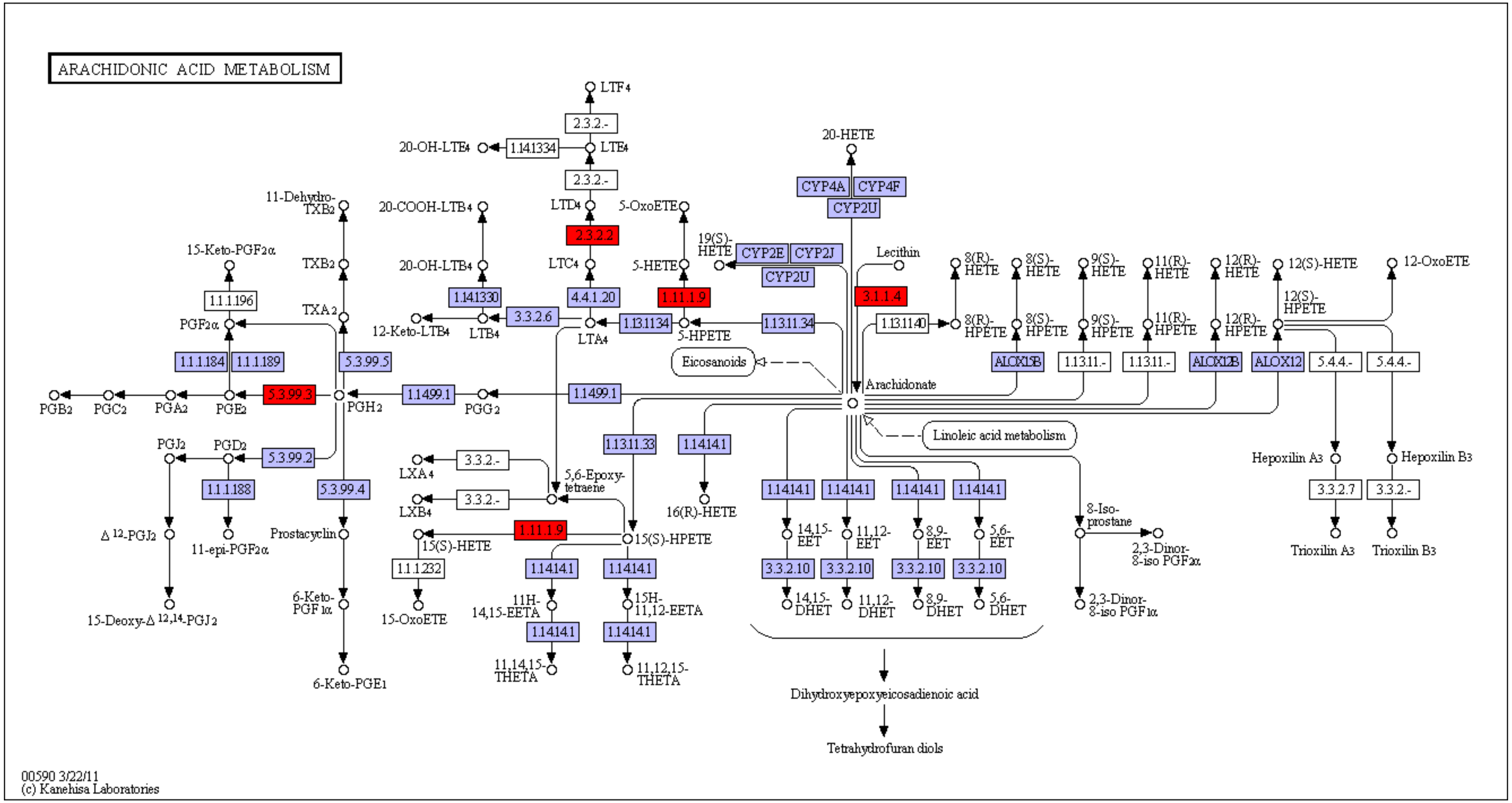

Figure 204: ko00590 Arachidonic acid metabolism

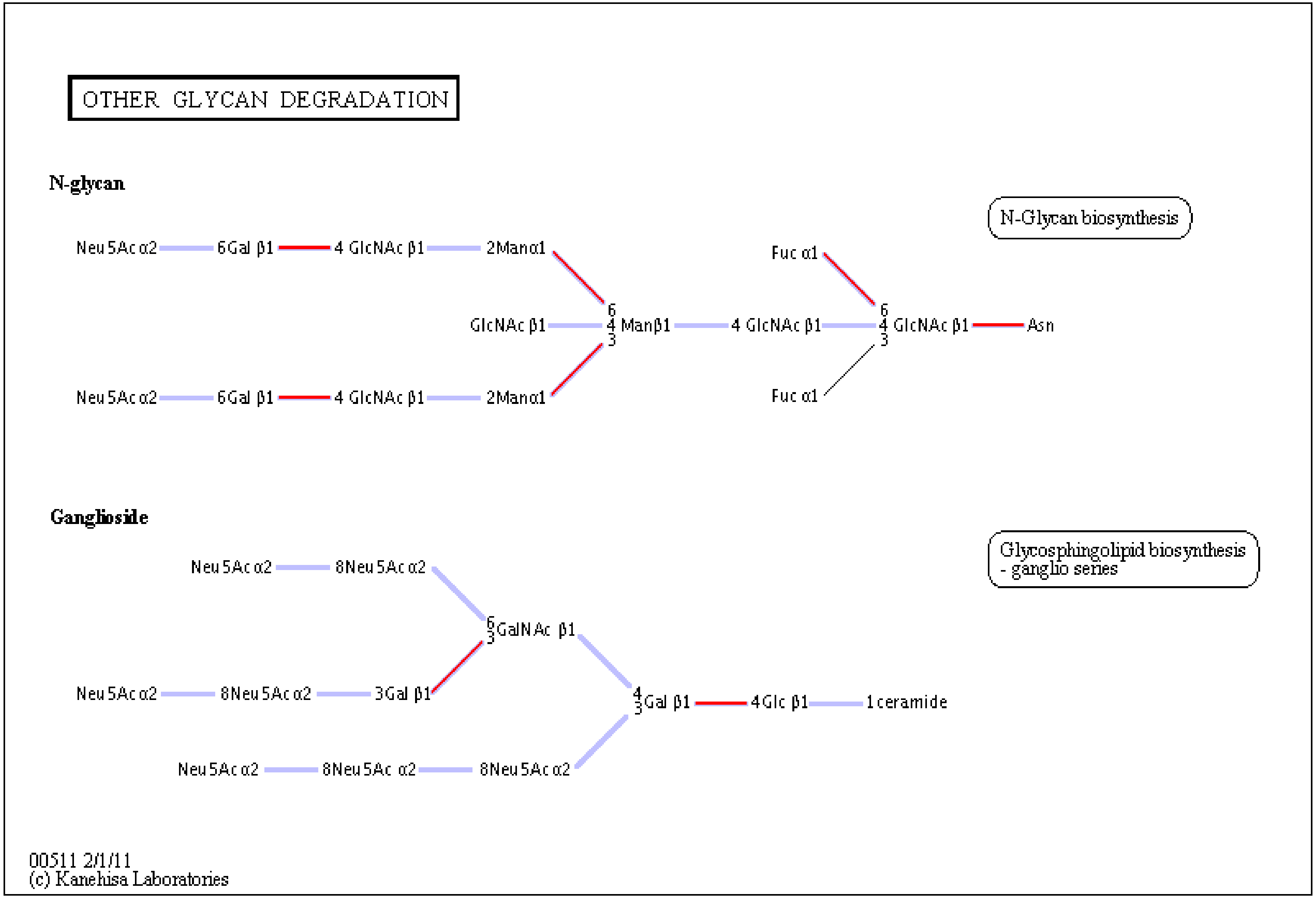

Figure 205: ko00511 Other glycan degradation

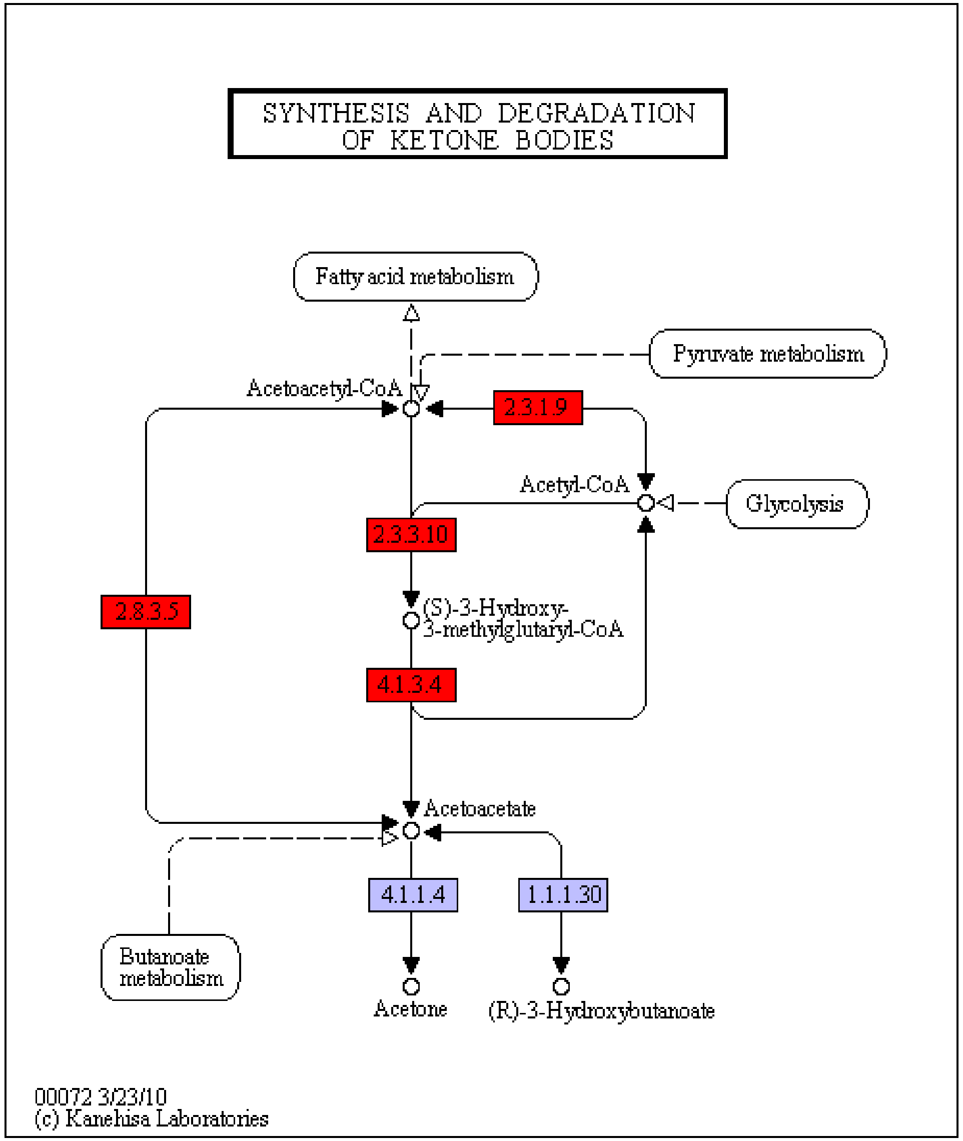

Figure 206: ko000072 Synthesis and degradation of ketone bodies

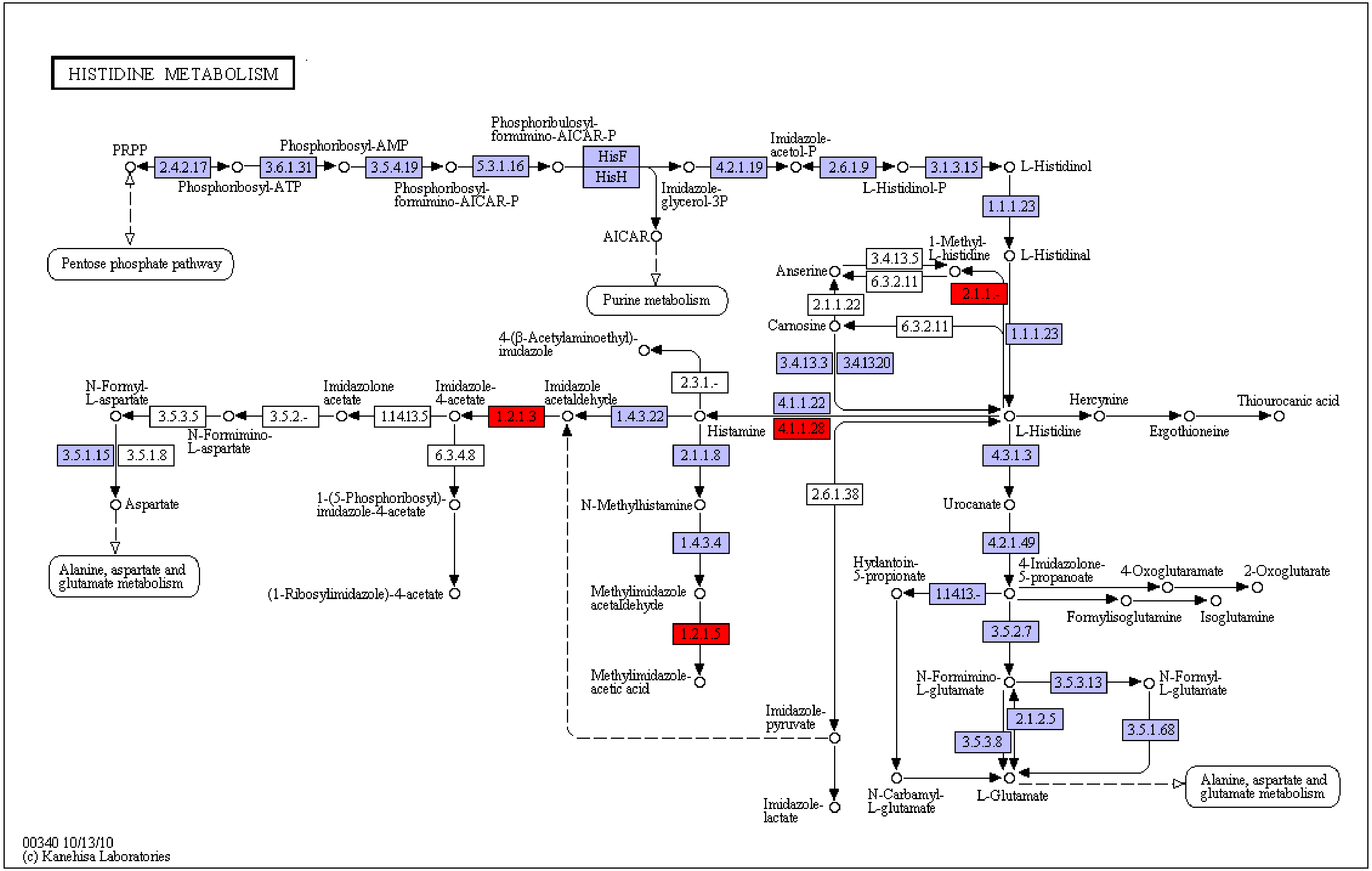

Figure 207: ko00340 Histidine metabolism

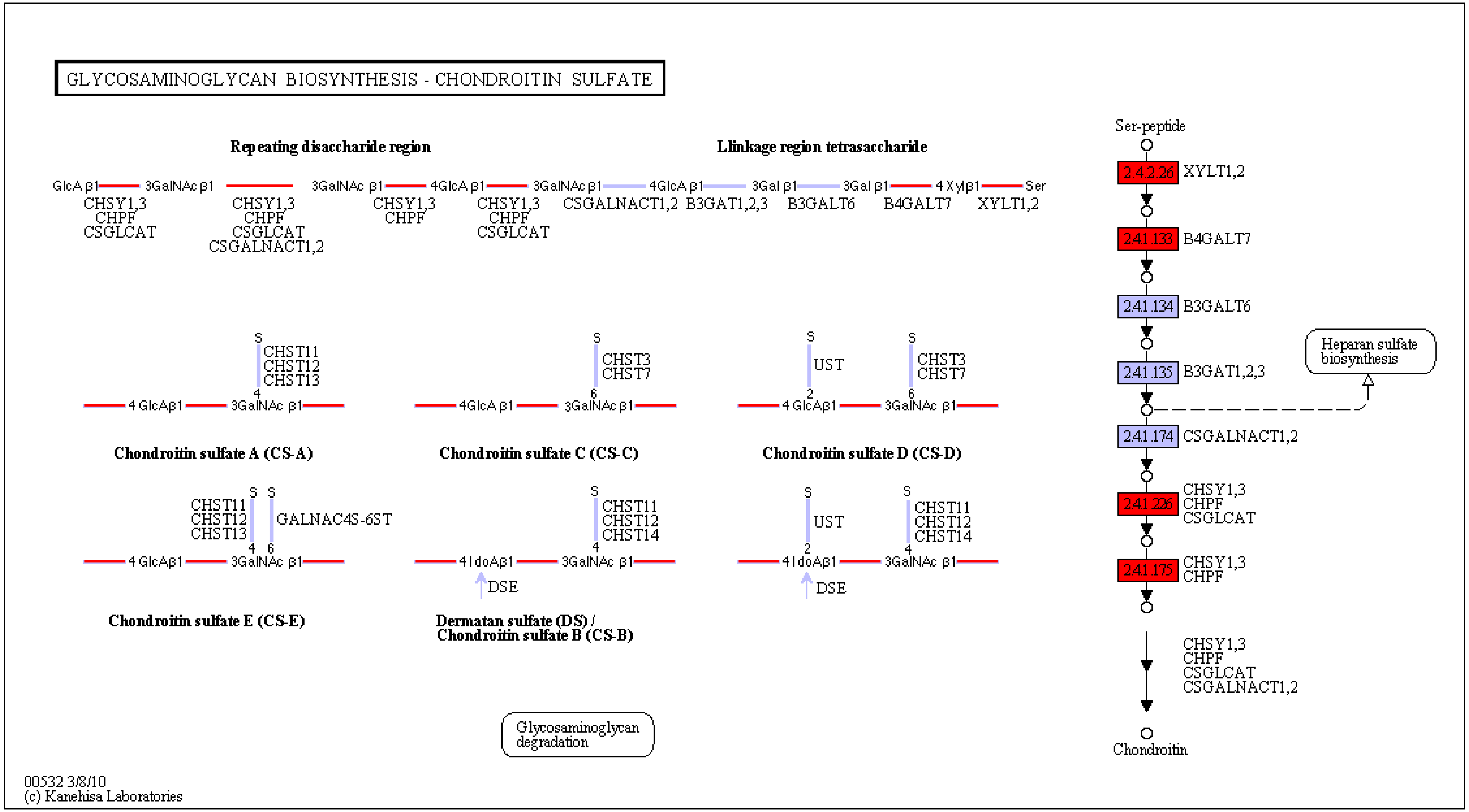

Figure 208: ko00532 Glycosaminoglycan biosynthesis - chondroitin sulfate

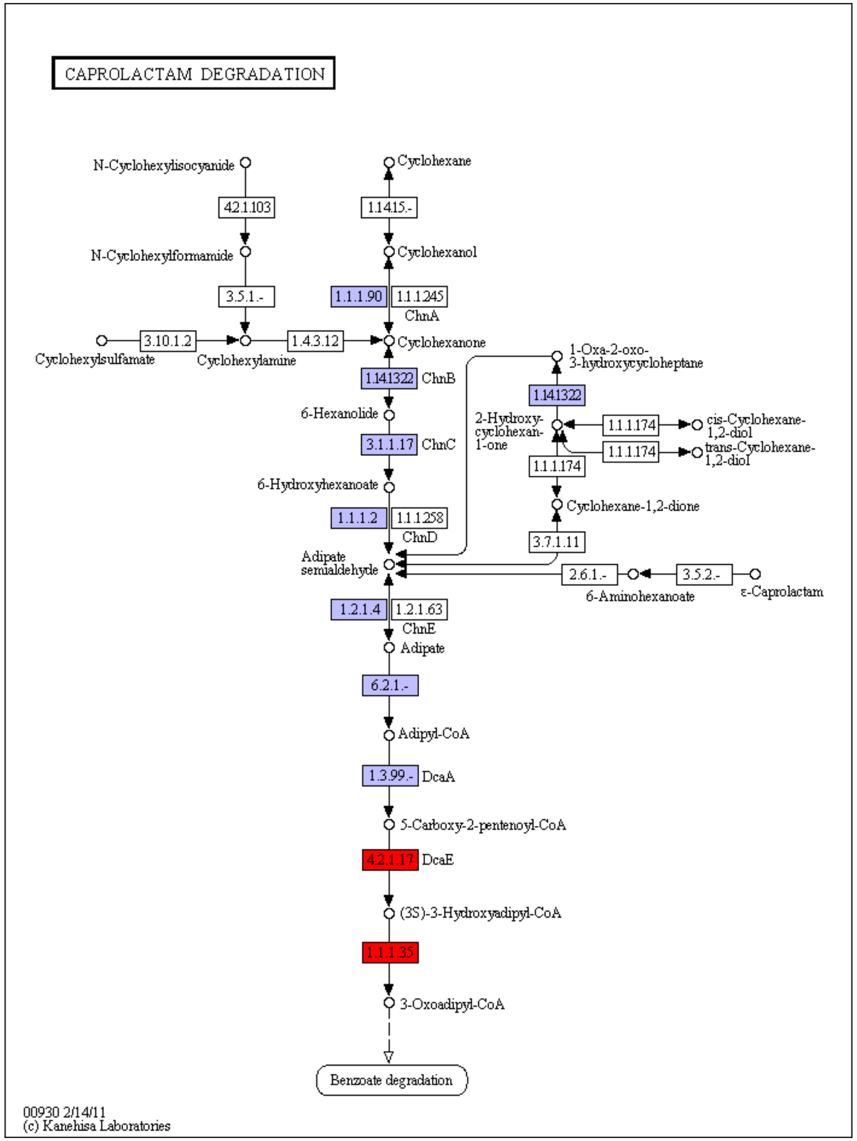

Figure 209: ko00930 Caprolactam degradation

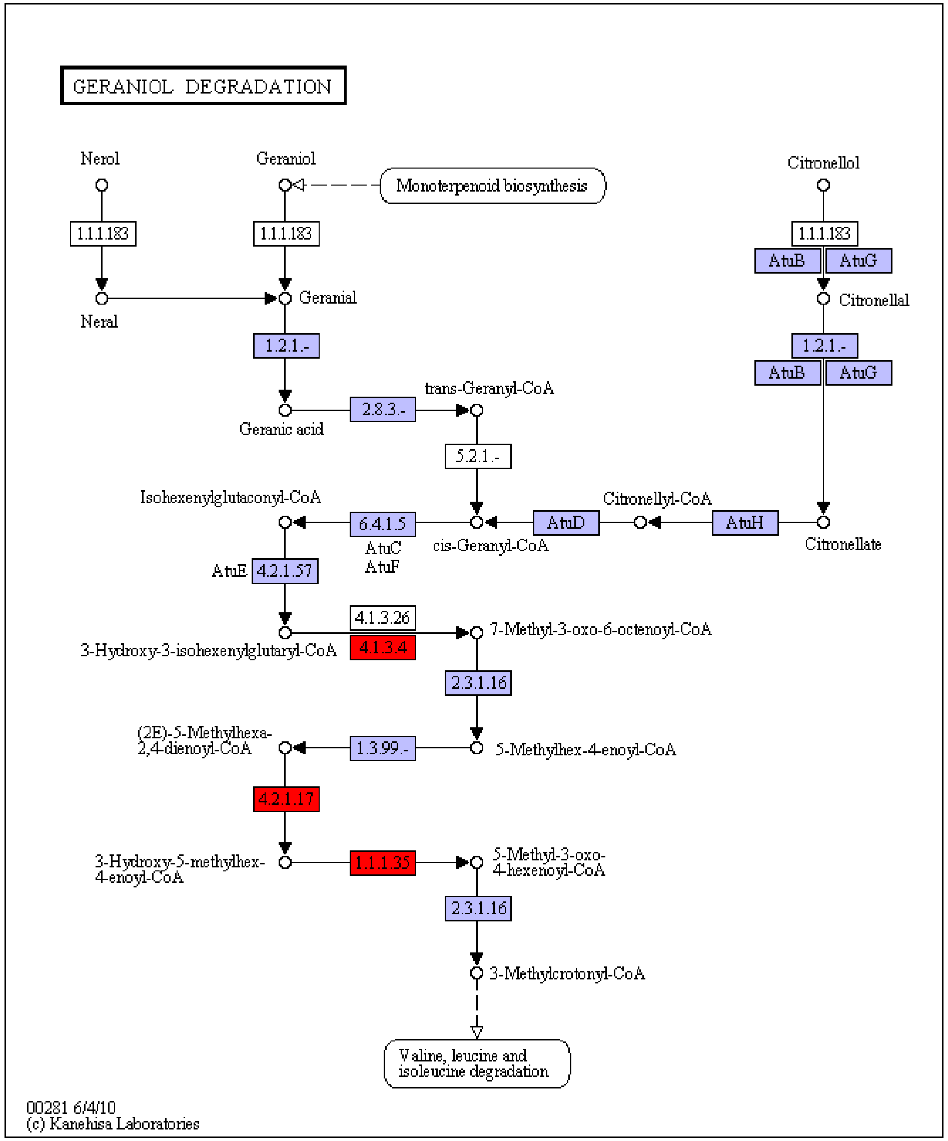

Figure 210: ko00281 Geraniol degradation

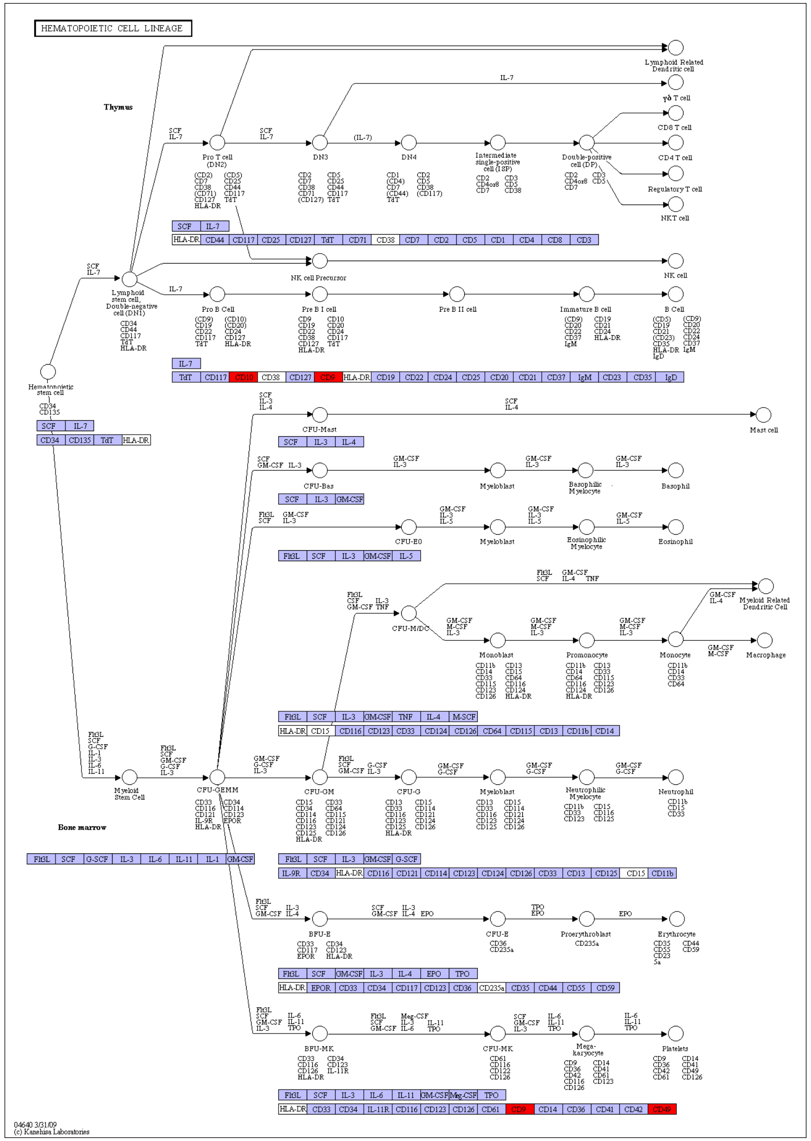

Figure 211: ko04640 Hematopoietic cell lineage

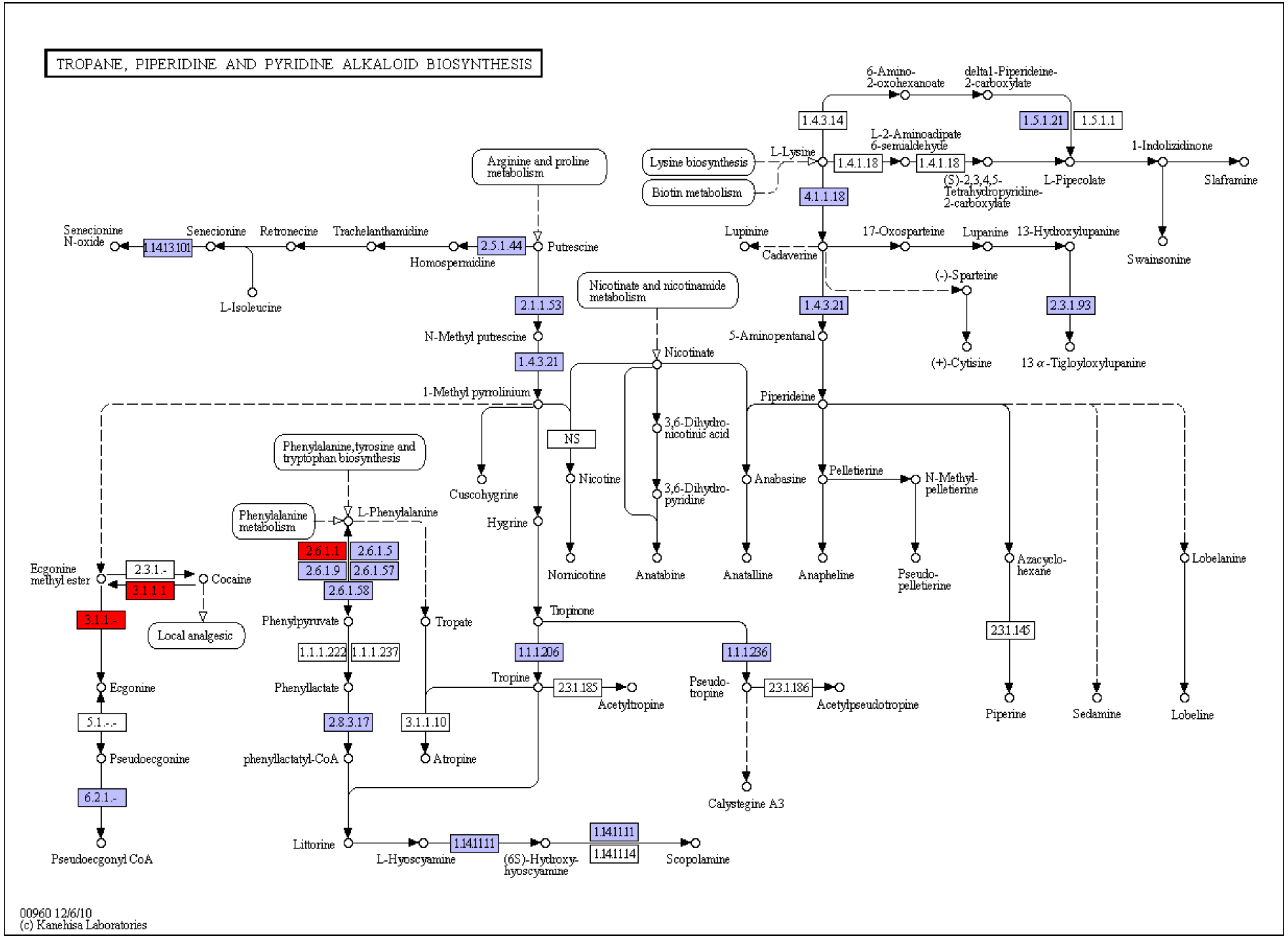

Figure 212: ko00960 Tropane, piperidine and pyridine alkaloid biosynthesis

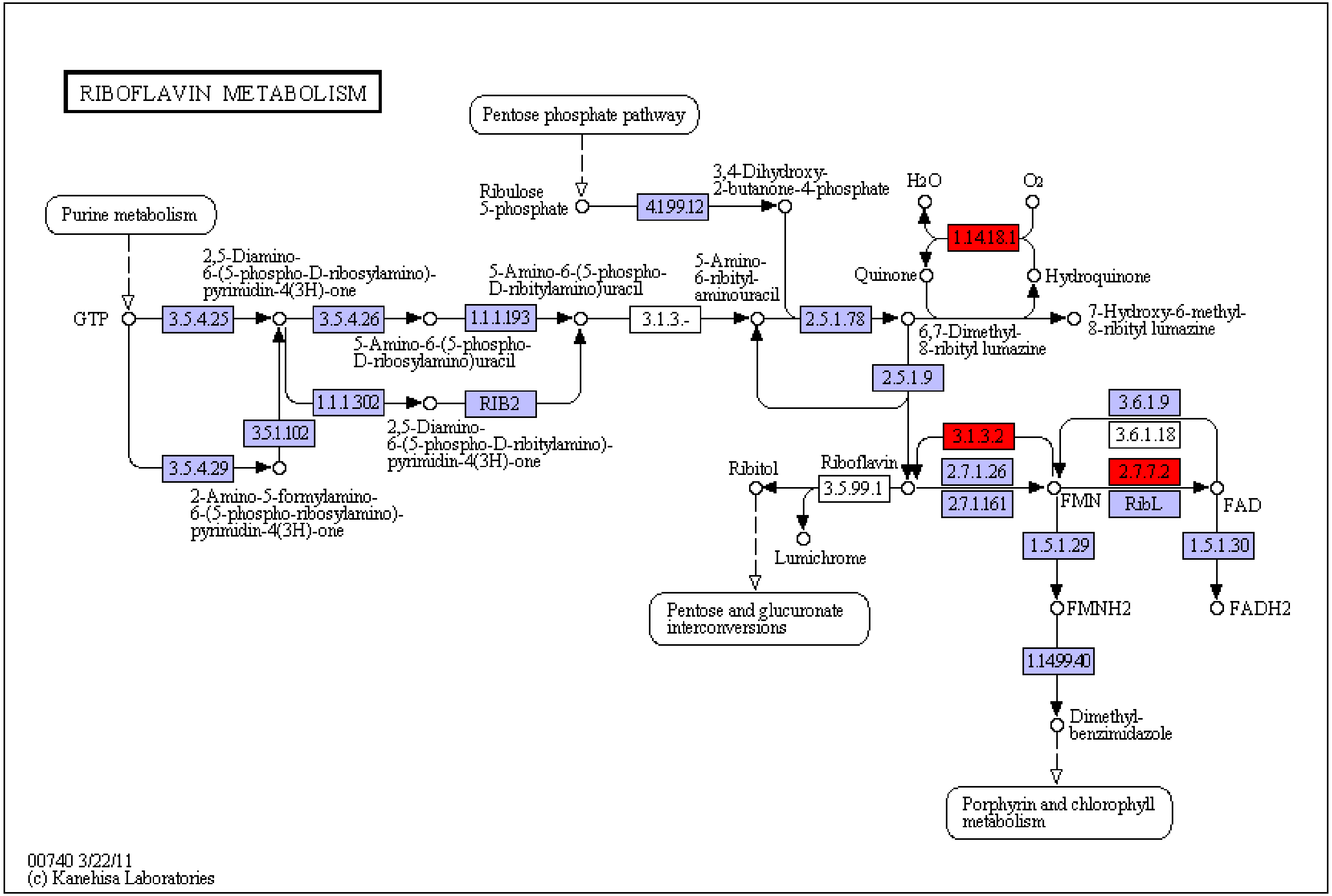

Figure 213: ko00740 Riboflavin metabolism

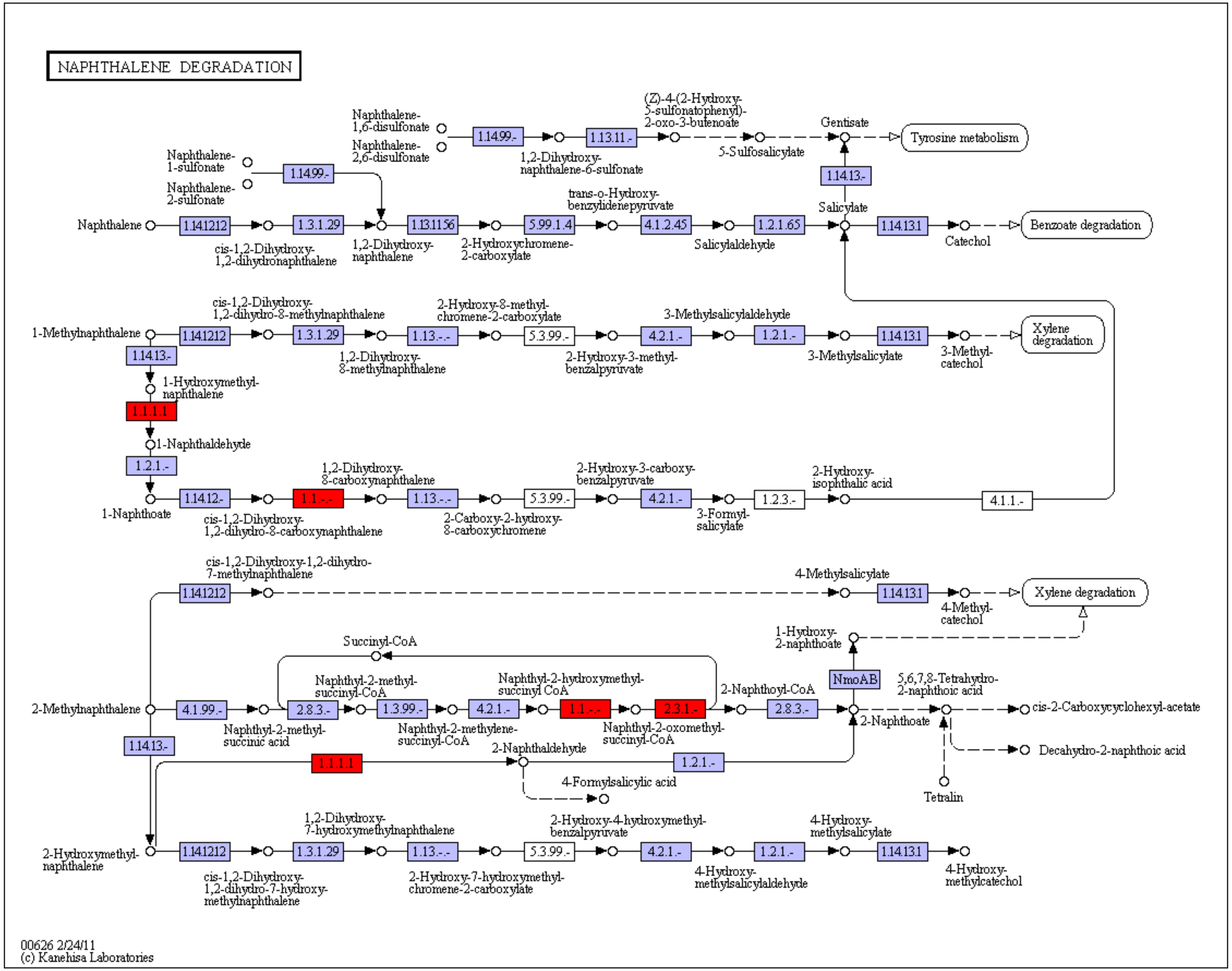

Figure 214: ko00626 Naphthalene degradation

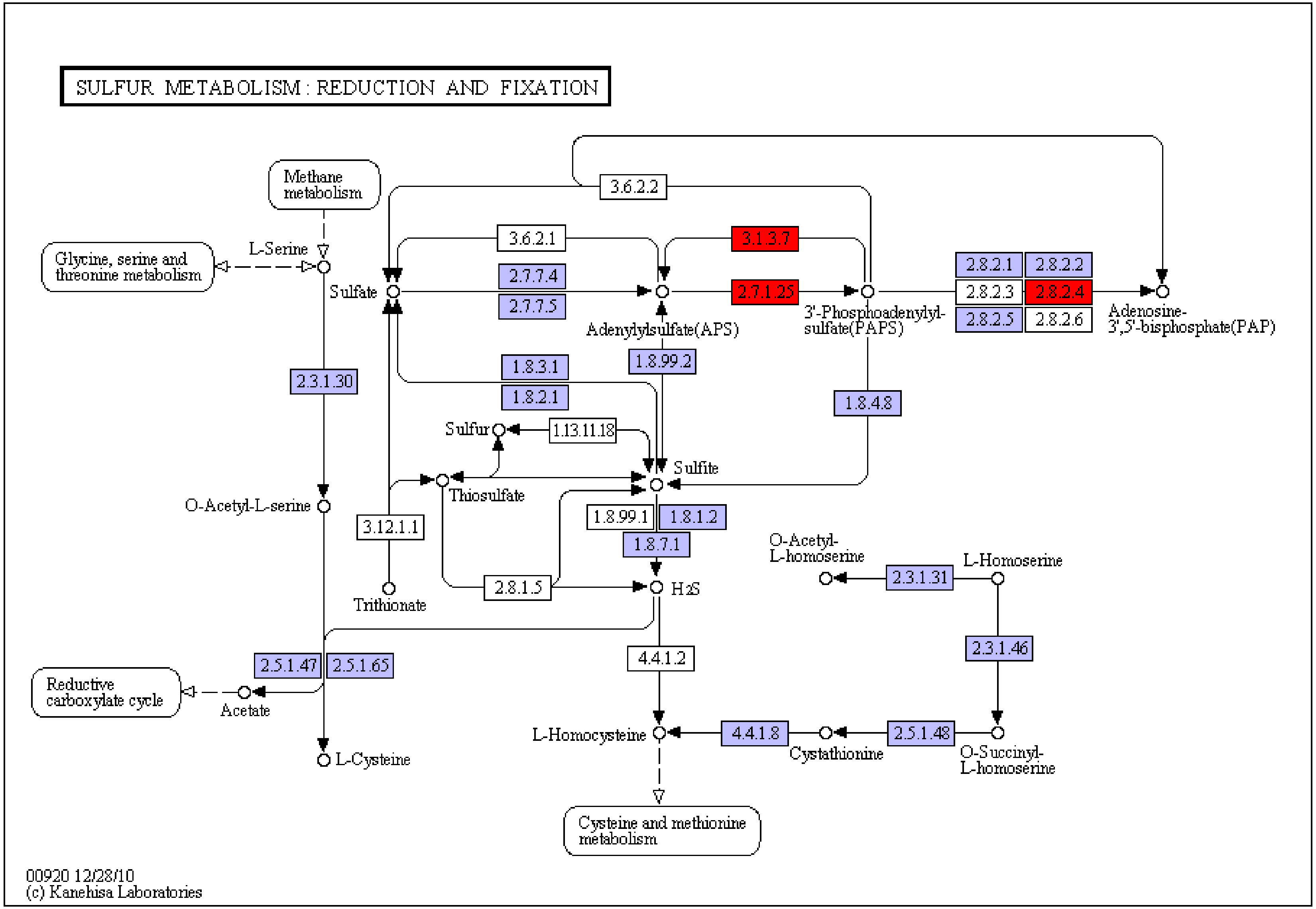

Figure 215: ko00920 Sulfur metabolism

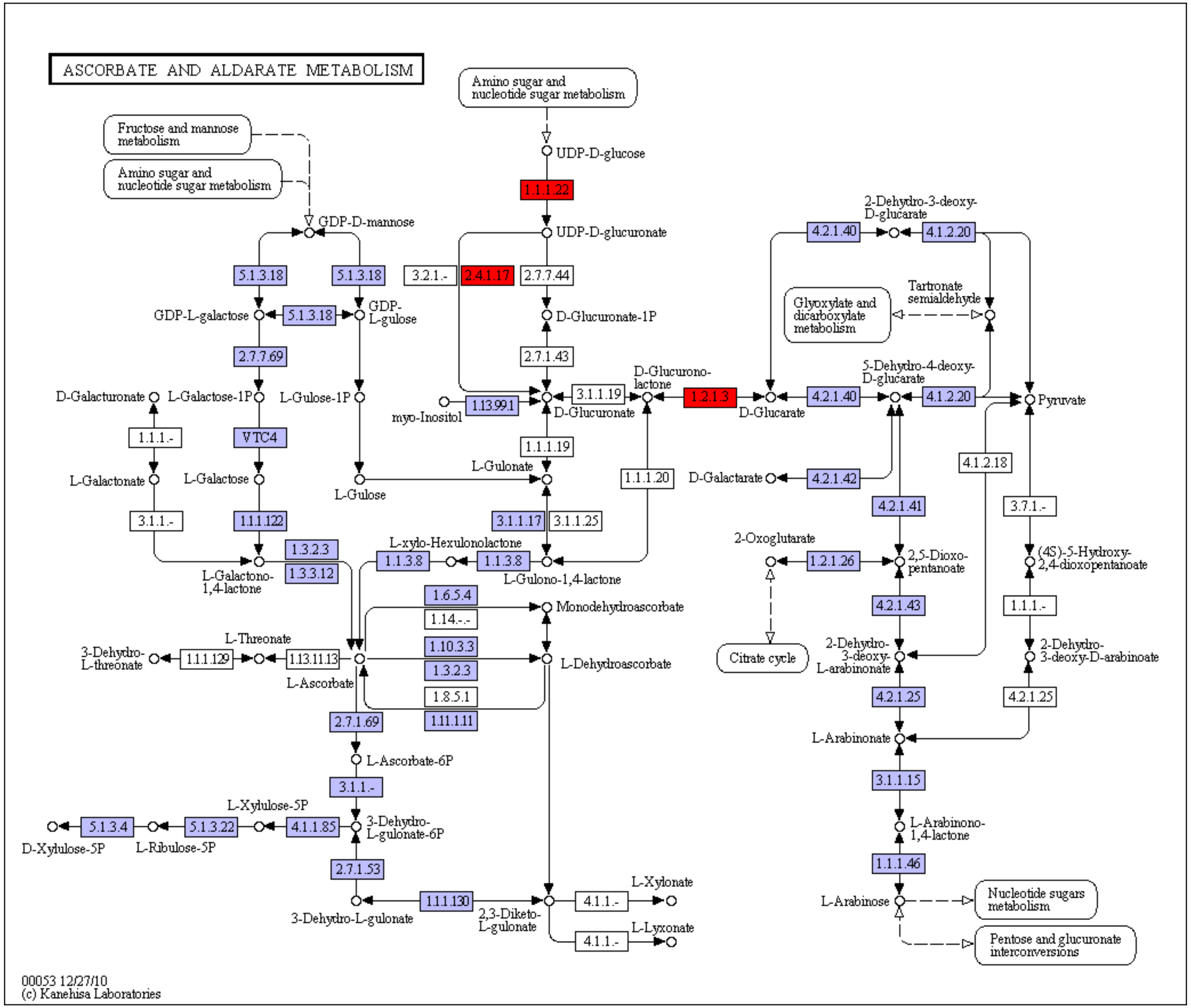

Figure 216: ko00053 Ascorbate and aldarate metabolism

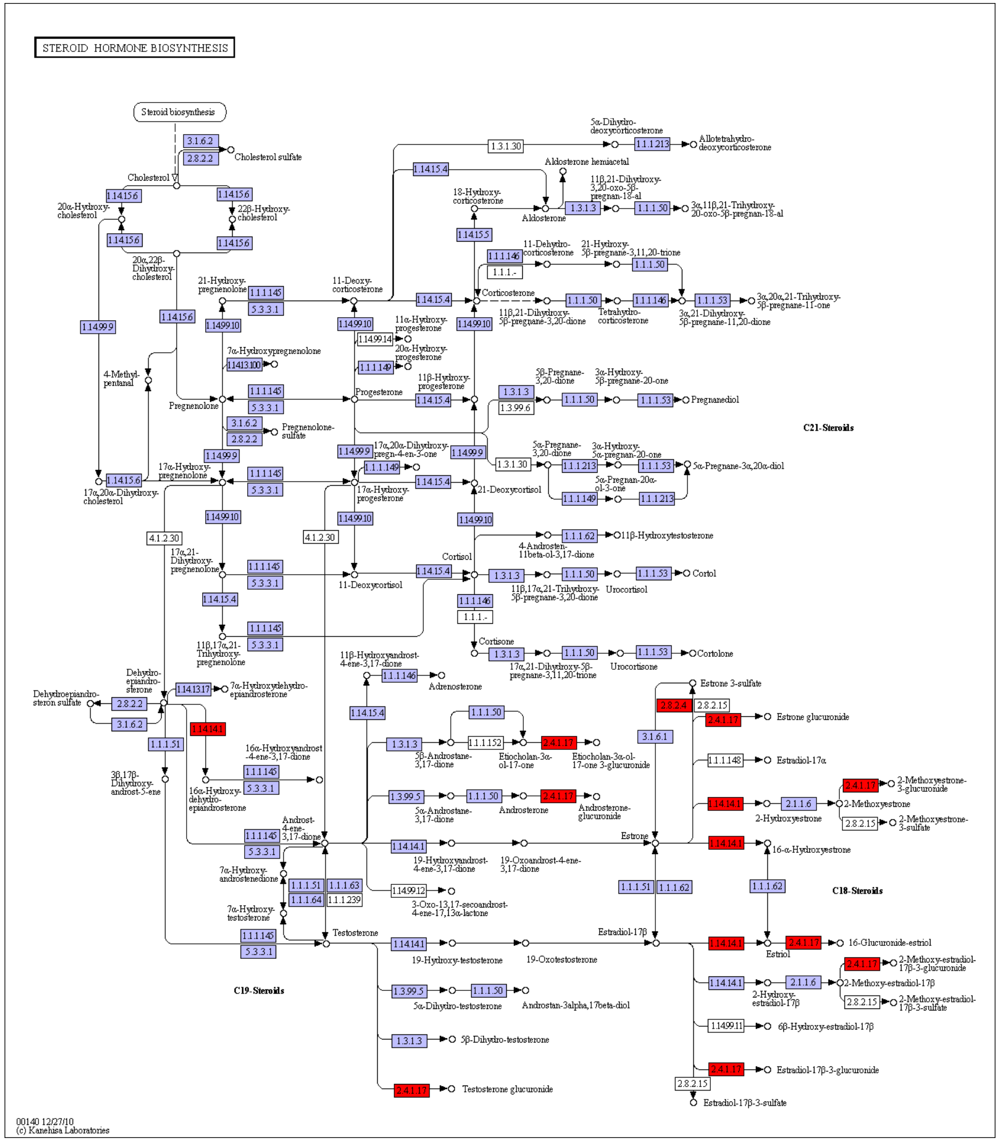

Figure 217: ko00140 Steroid hormone biosynthesis

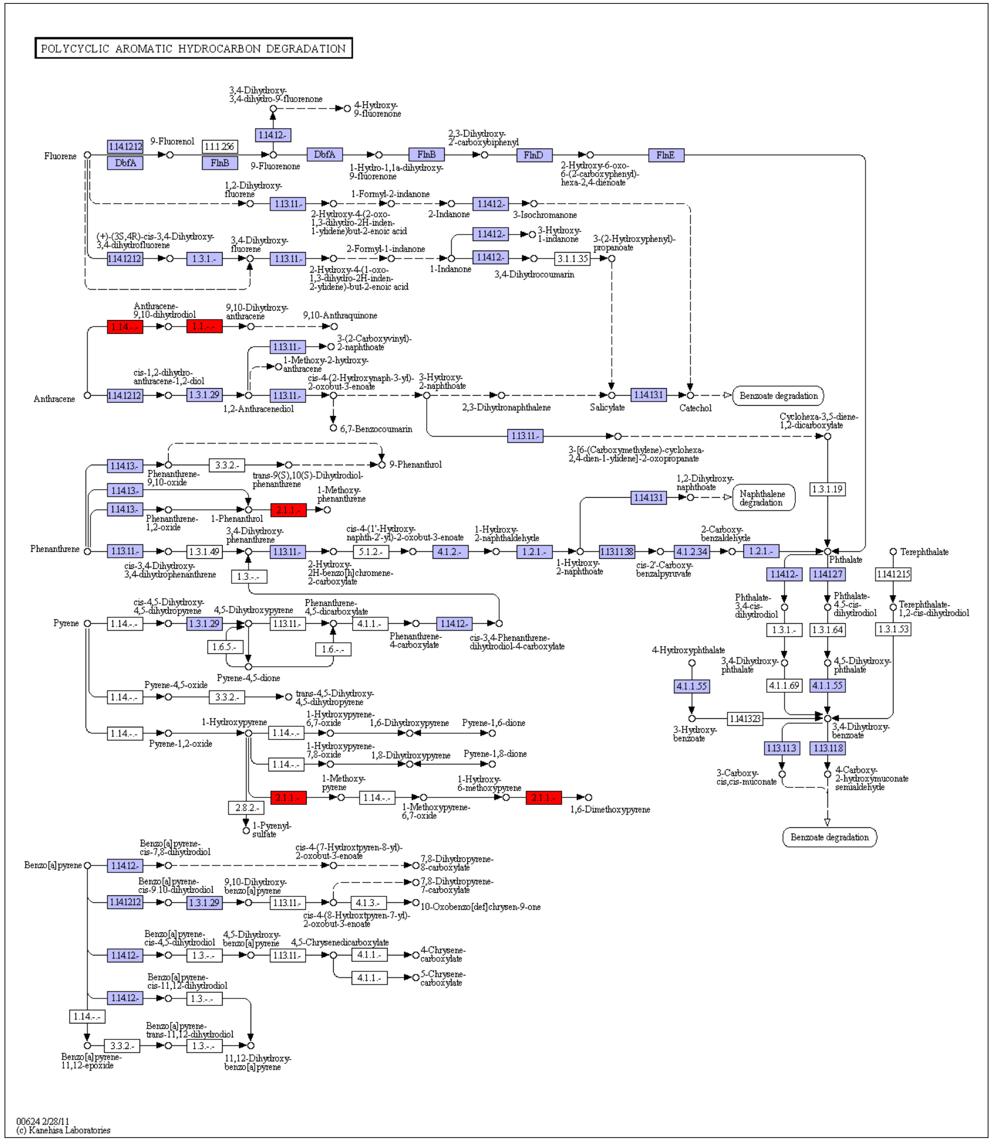

Figure 218: ko00624 Polycyclic aromatic hydrocarbon degradation

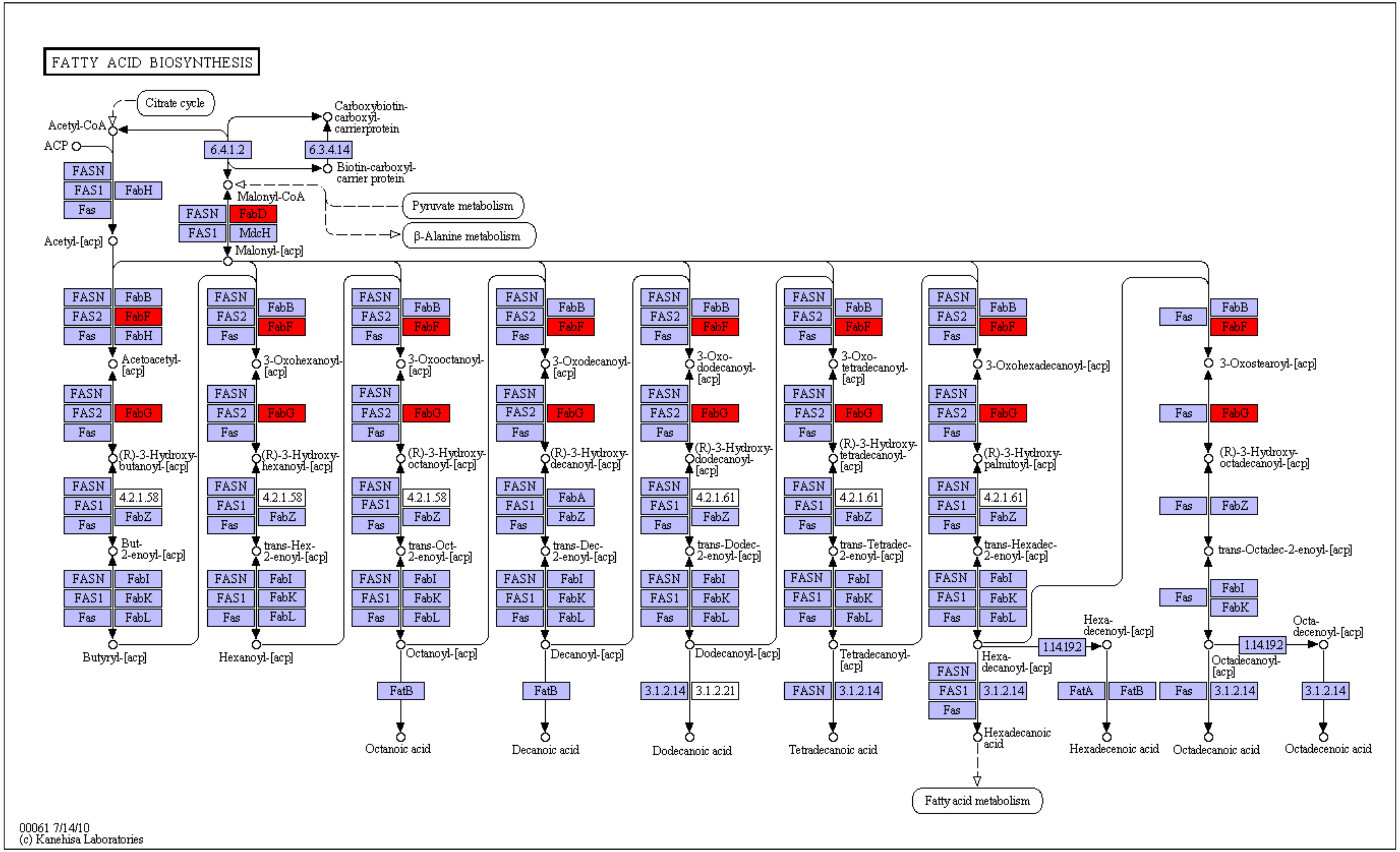

Figure 219: ko00061 Fatty acid biosynthesis

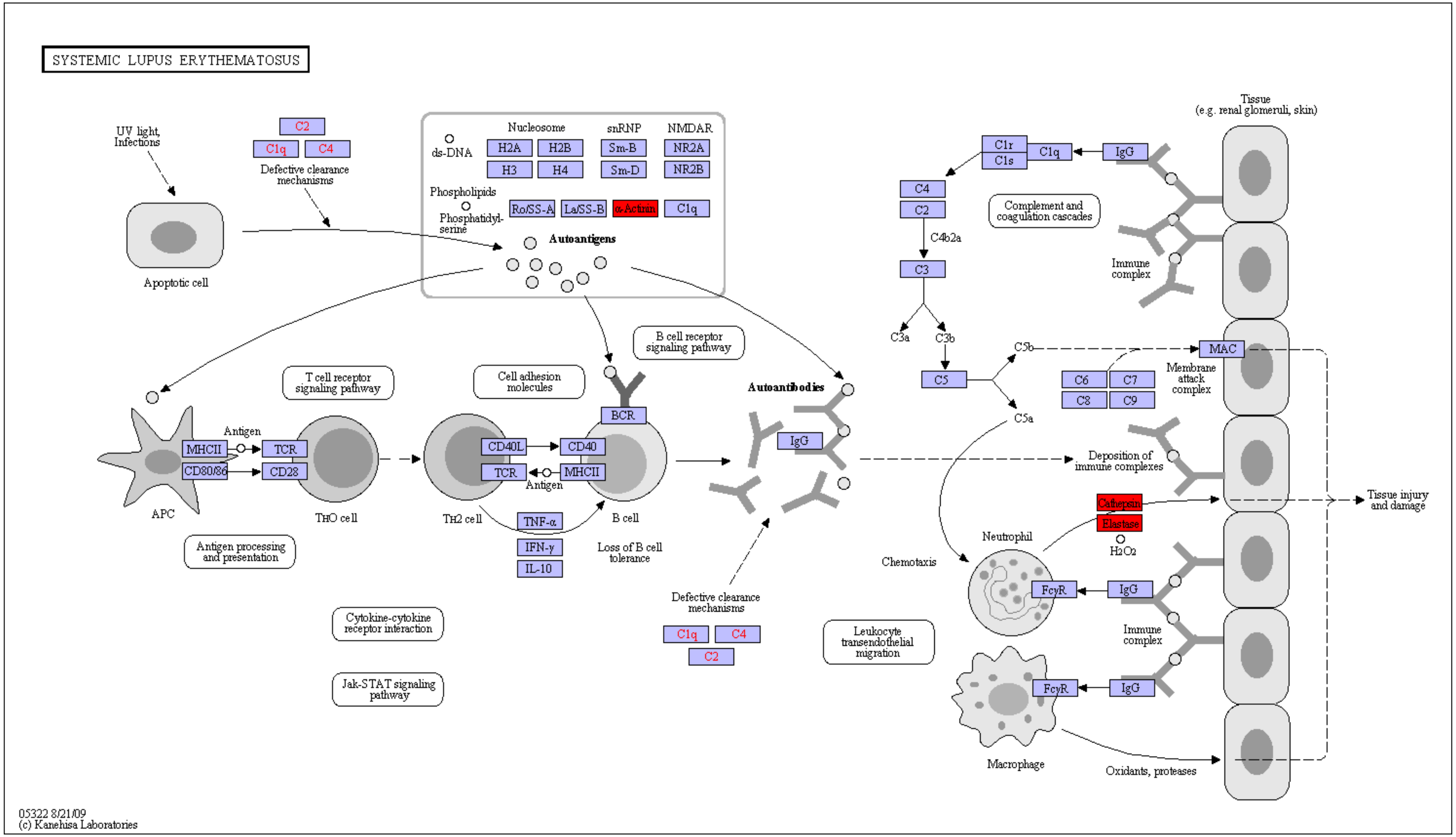

Figure 220: ko05322 Systemic lupus erythematosus

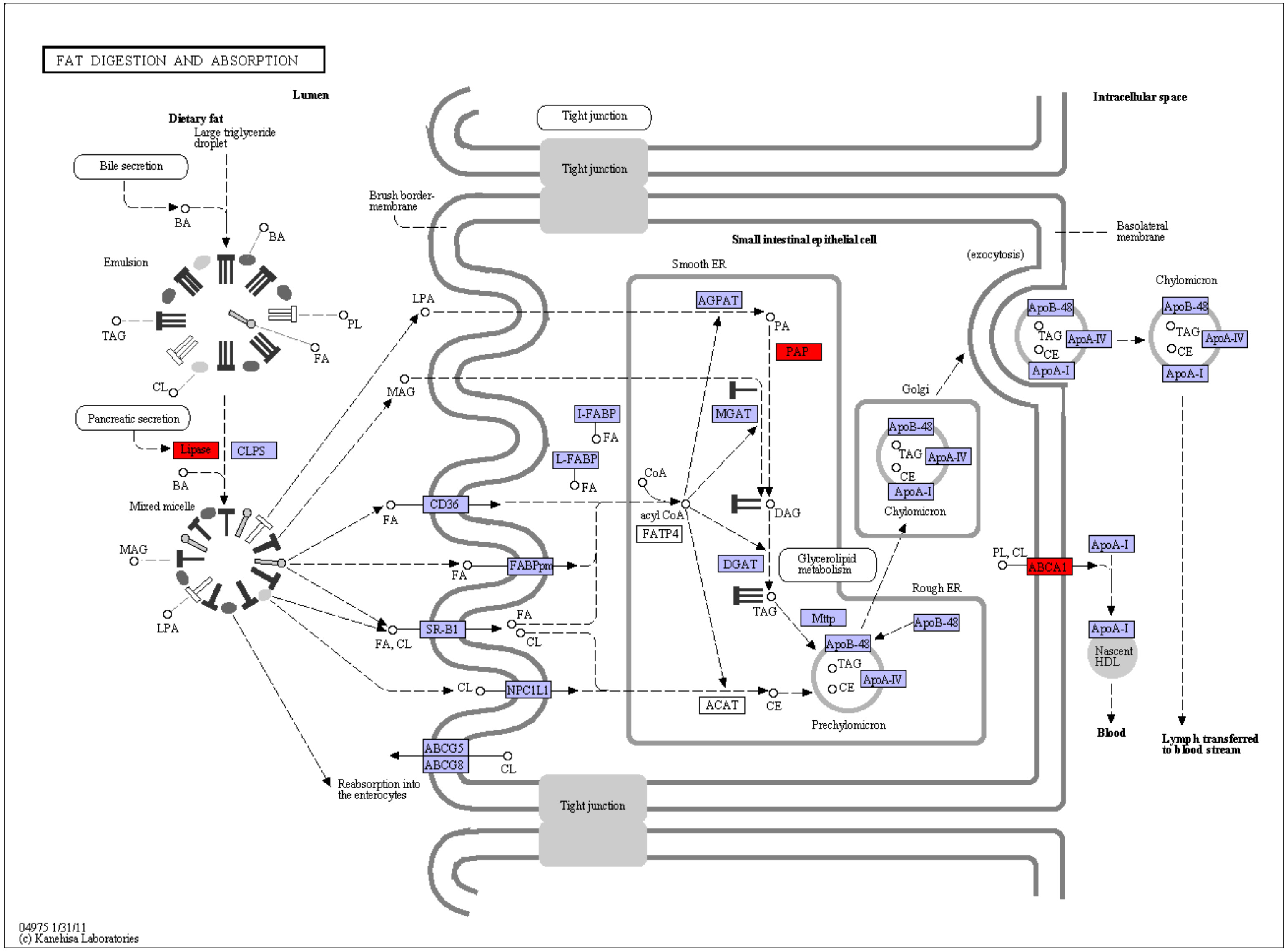

Figure 221: ko04975 Fat digestion and absorption

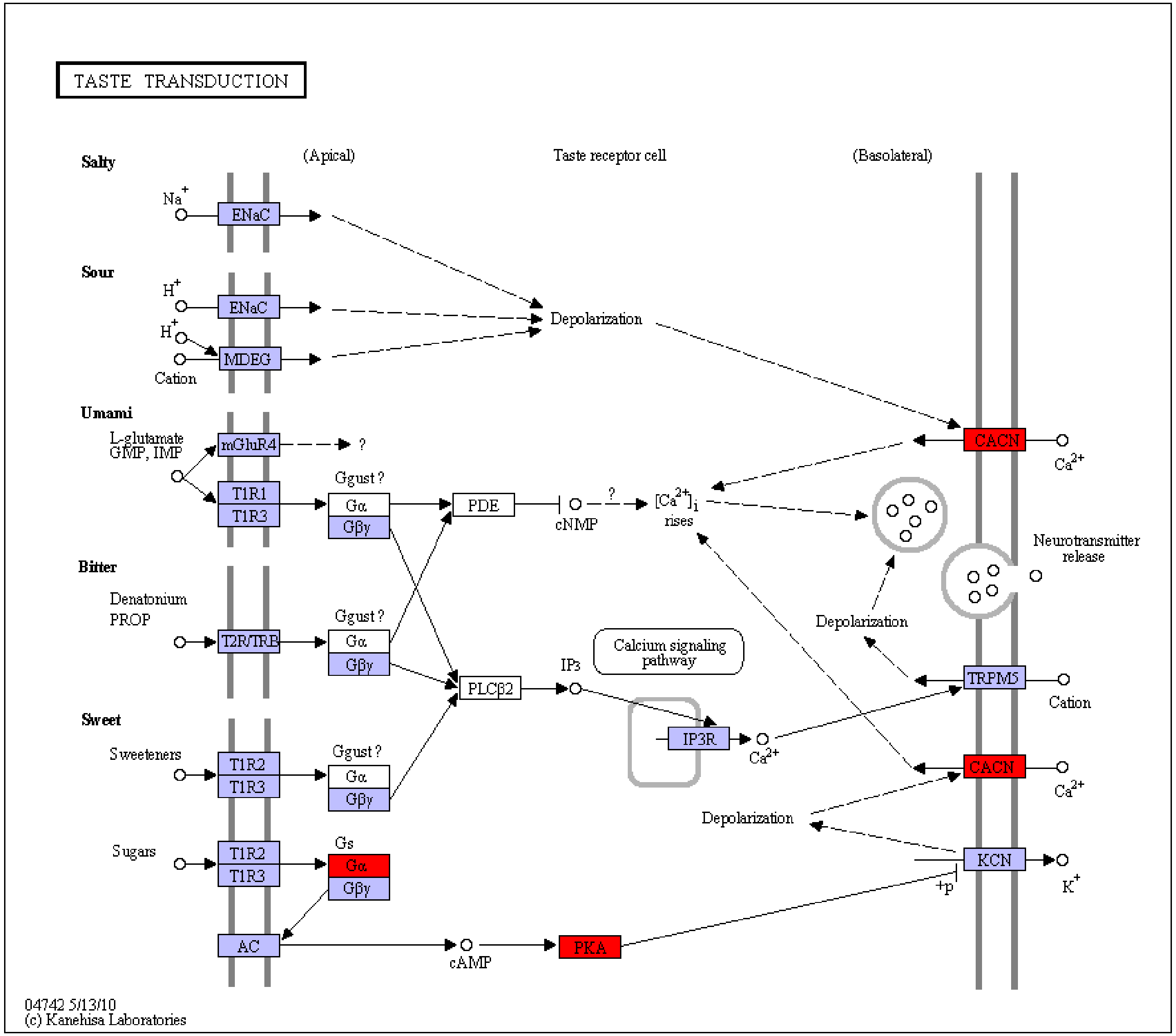

Figure 222: ko04742 Taste transduction

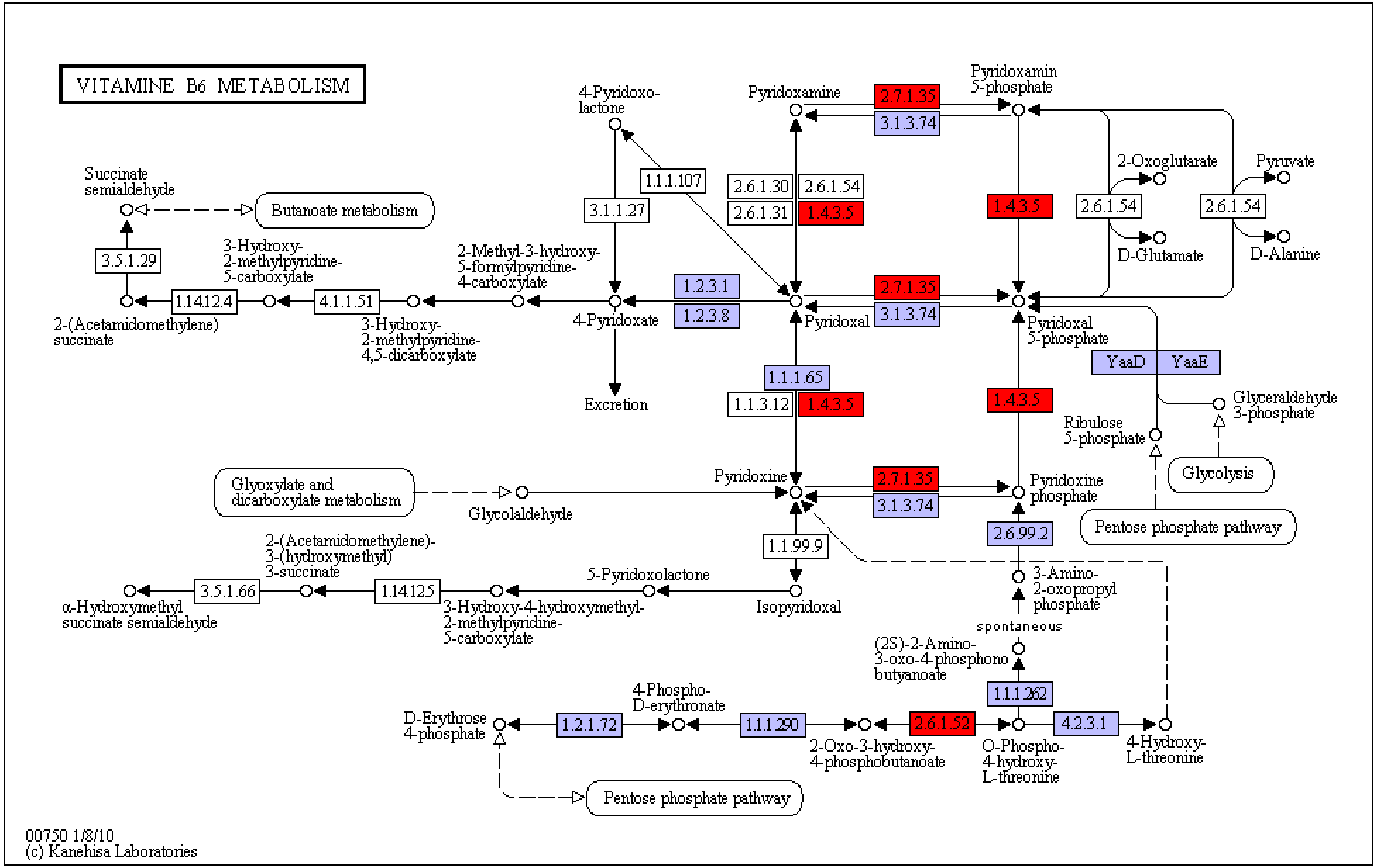

Figure 223: ko00750 Vitamin B6 metabolism

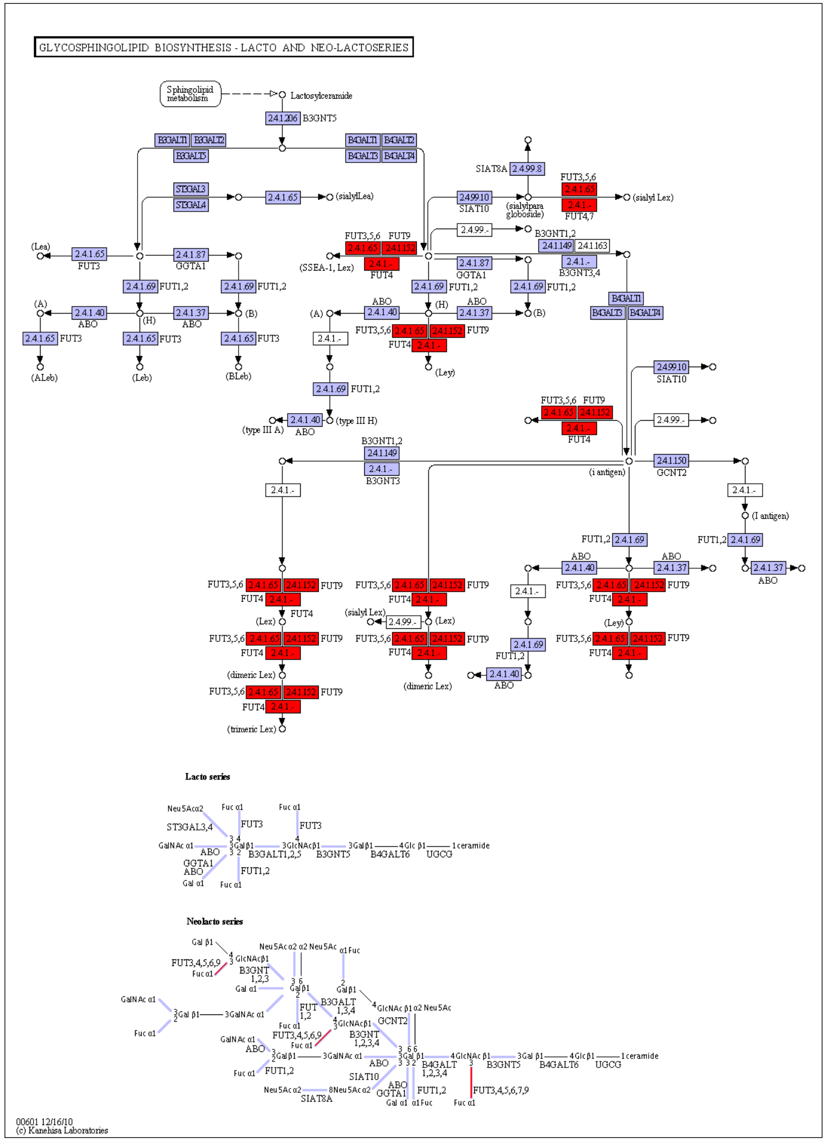

Figure 224: ko00601 Glycosphingolipid biosynthesis - lacto and neolacto series

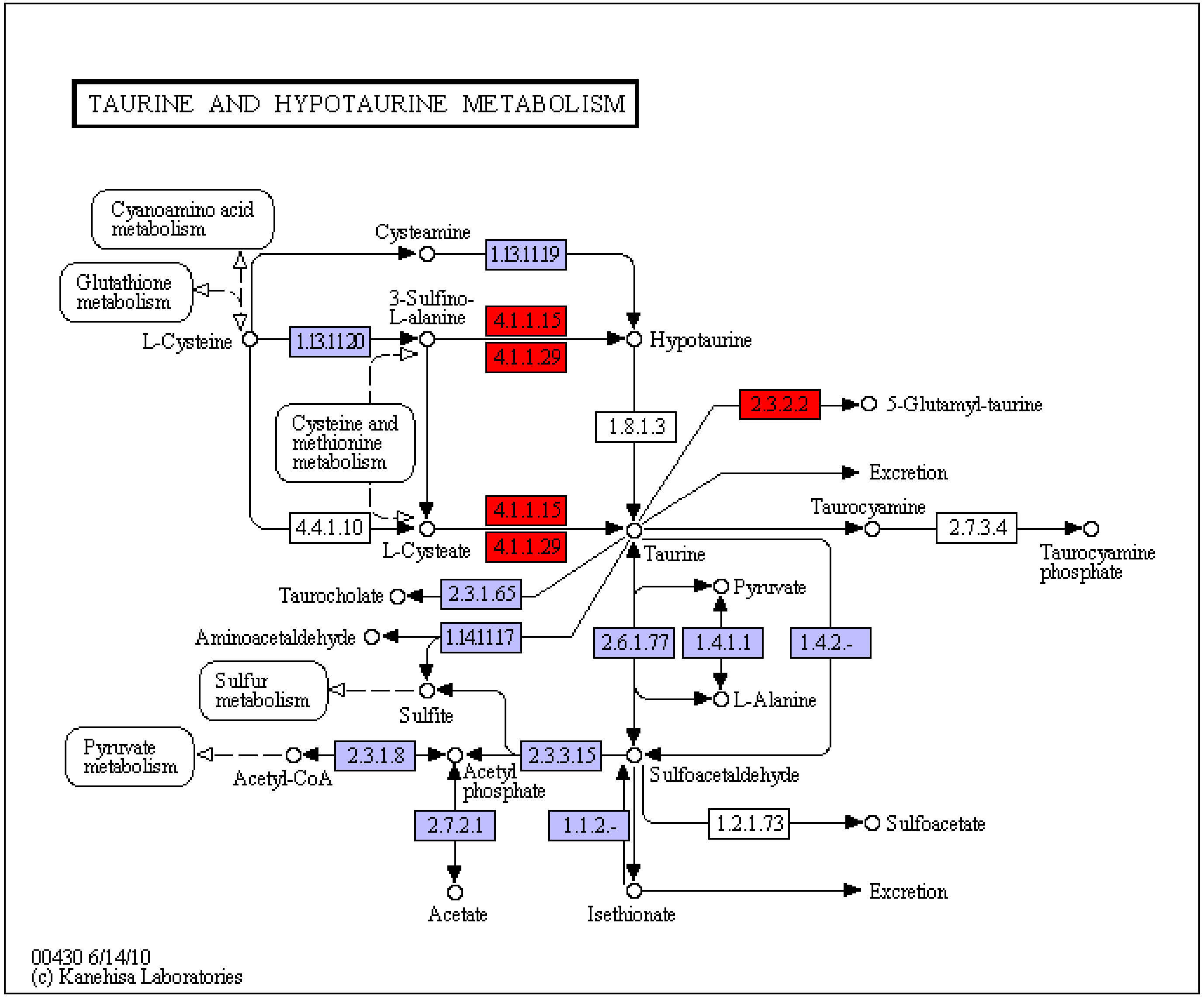

Figure 225: ko00430 Taurine and hypotaurine metabolism

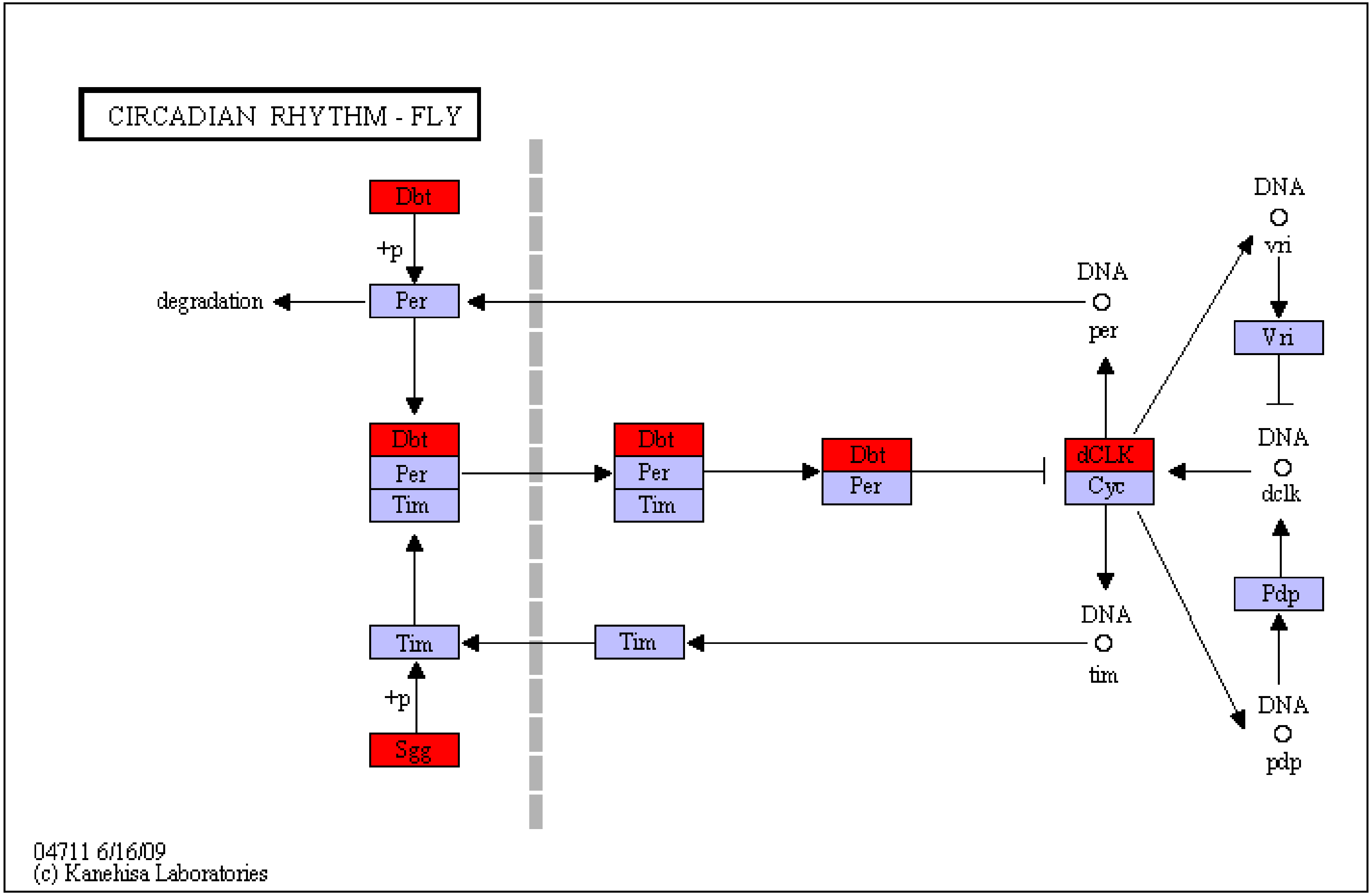

Figure 226: ko04711 Circadian rhythm - fly

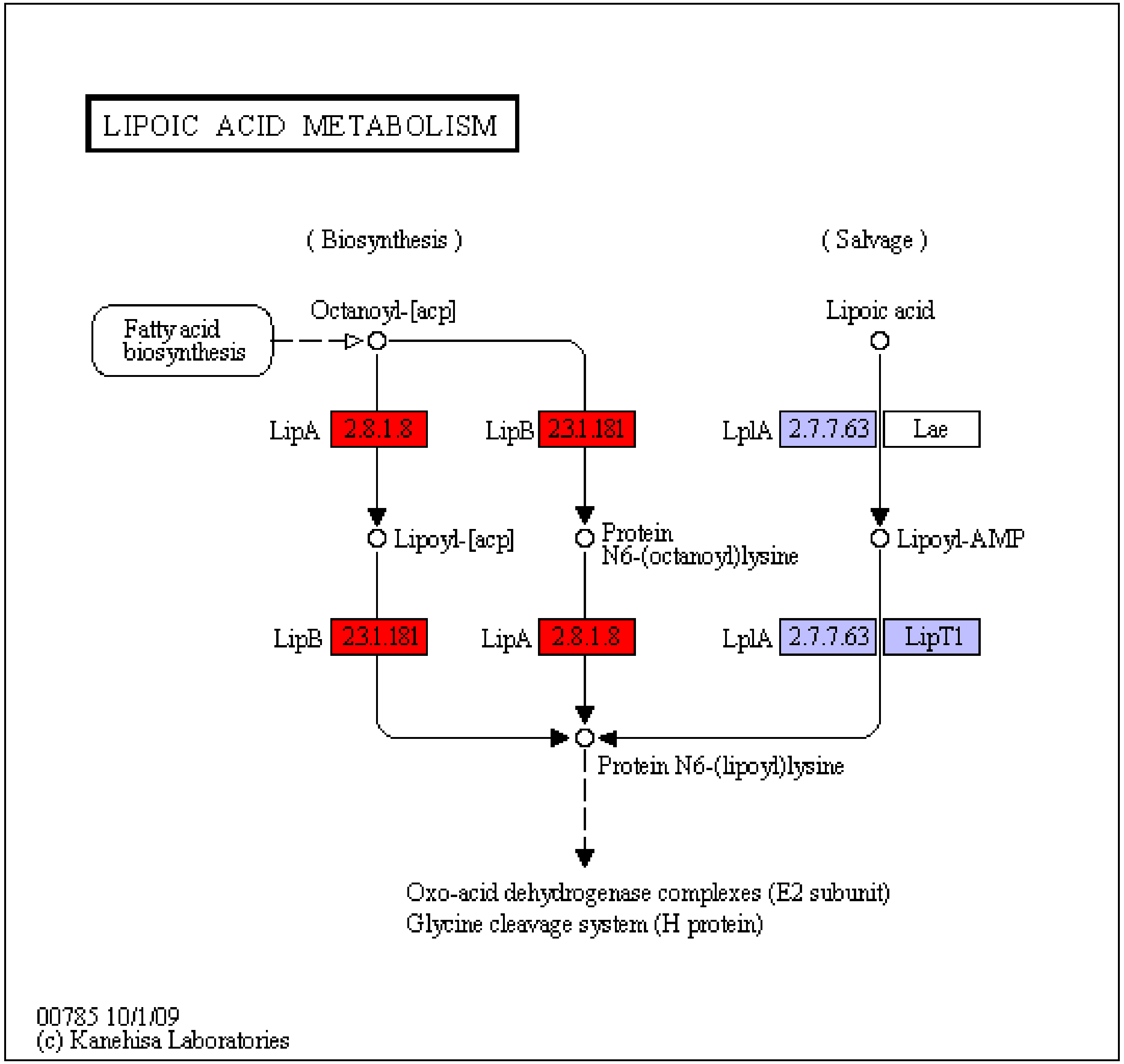

Figure 227: ko00785 Lipoic acid metabolism

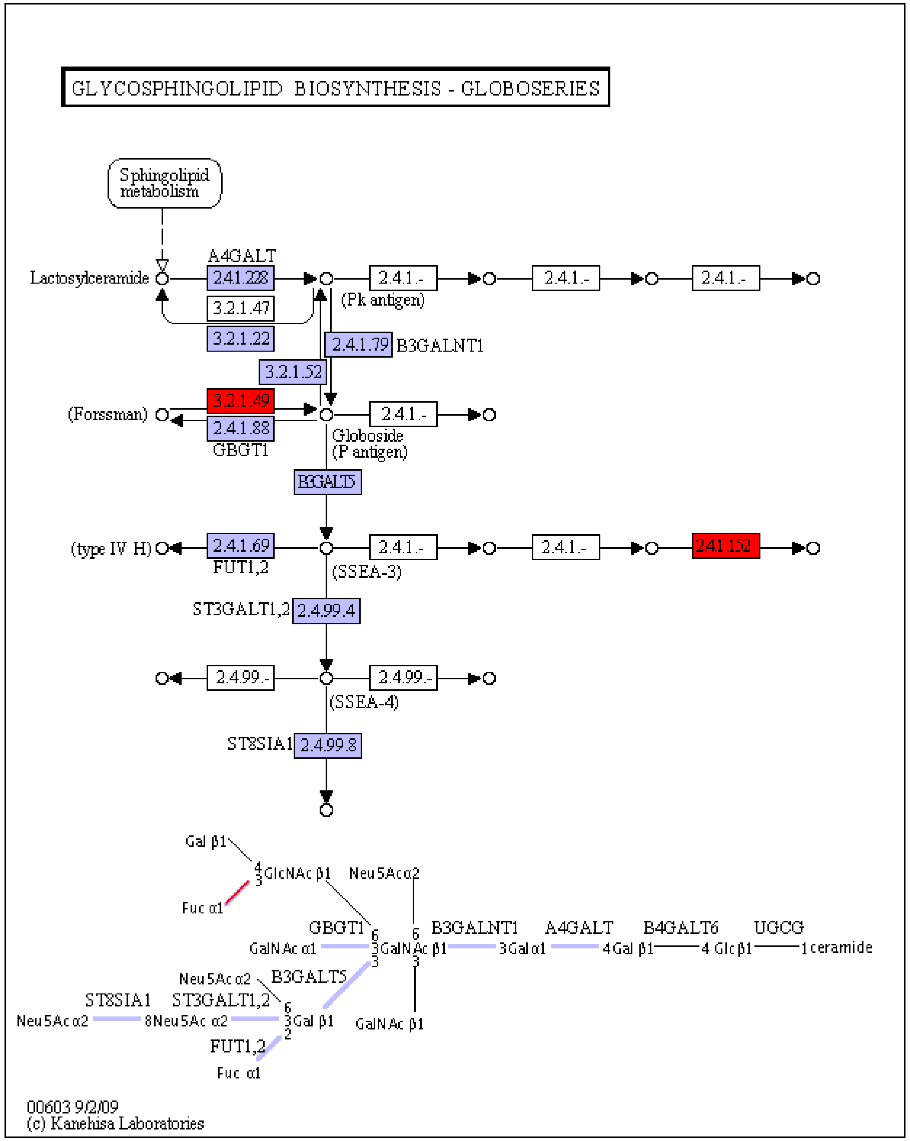

Figure 228: ko00603 Glycosphingolipid biosynthesis - globo series

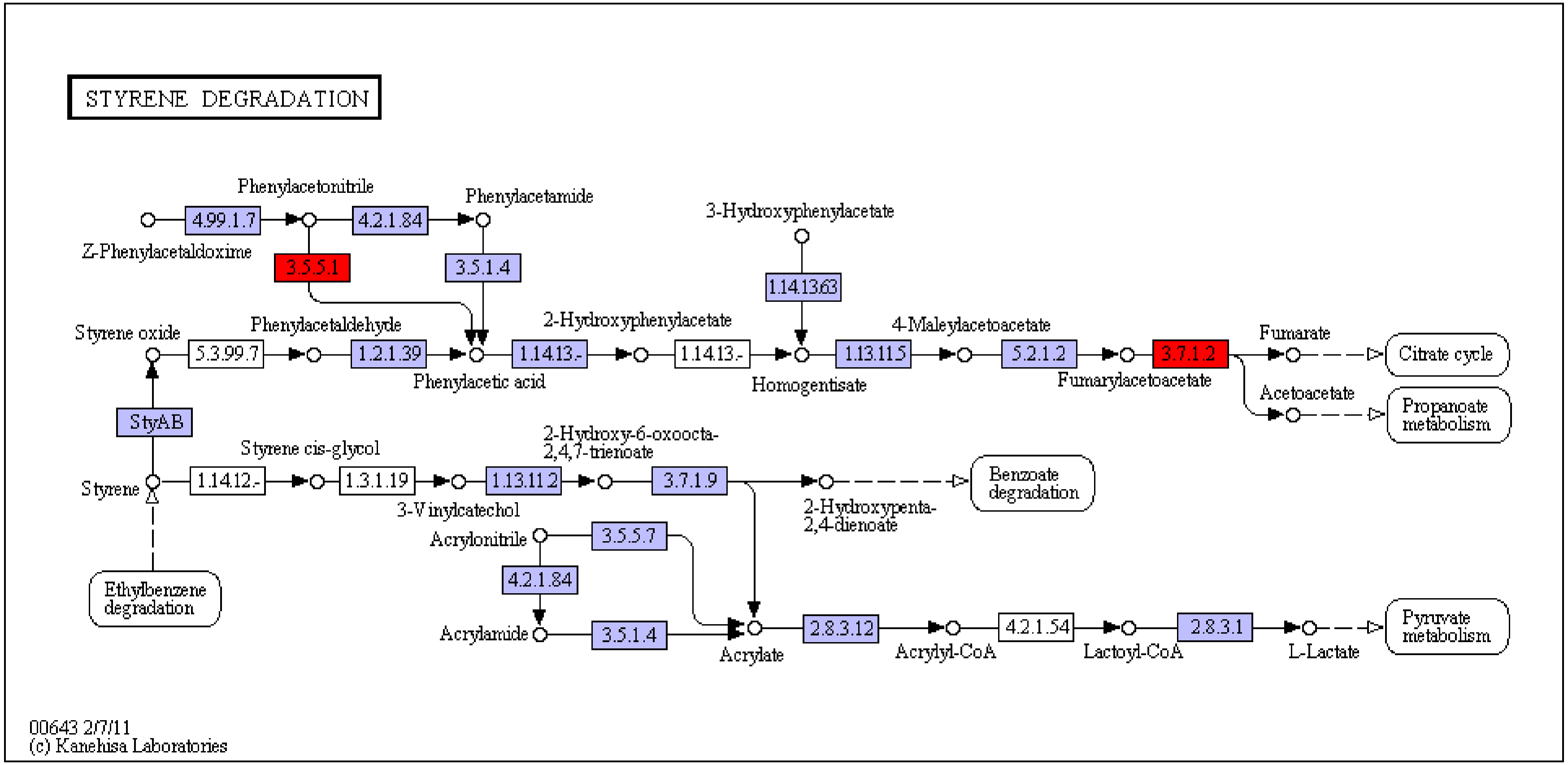

Figure 229: ko00643 Styrene degradation

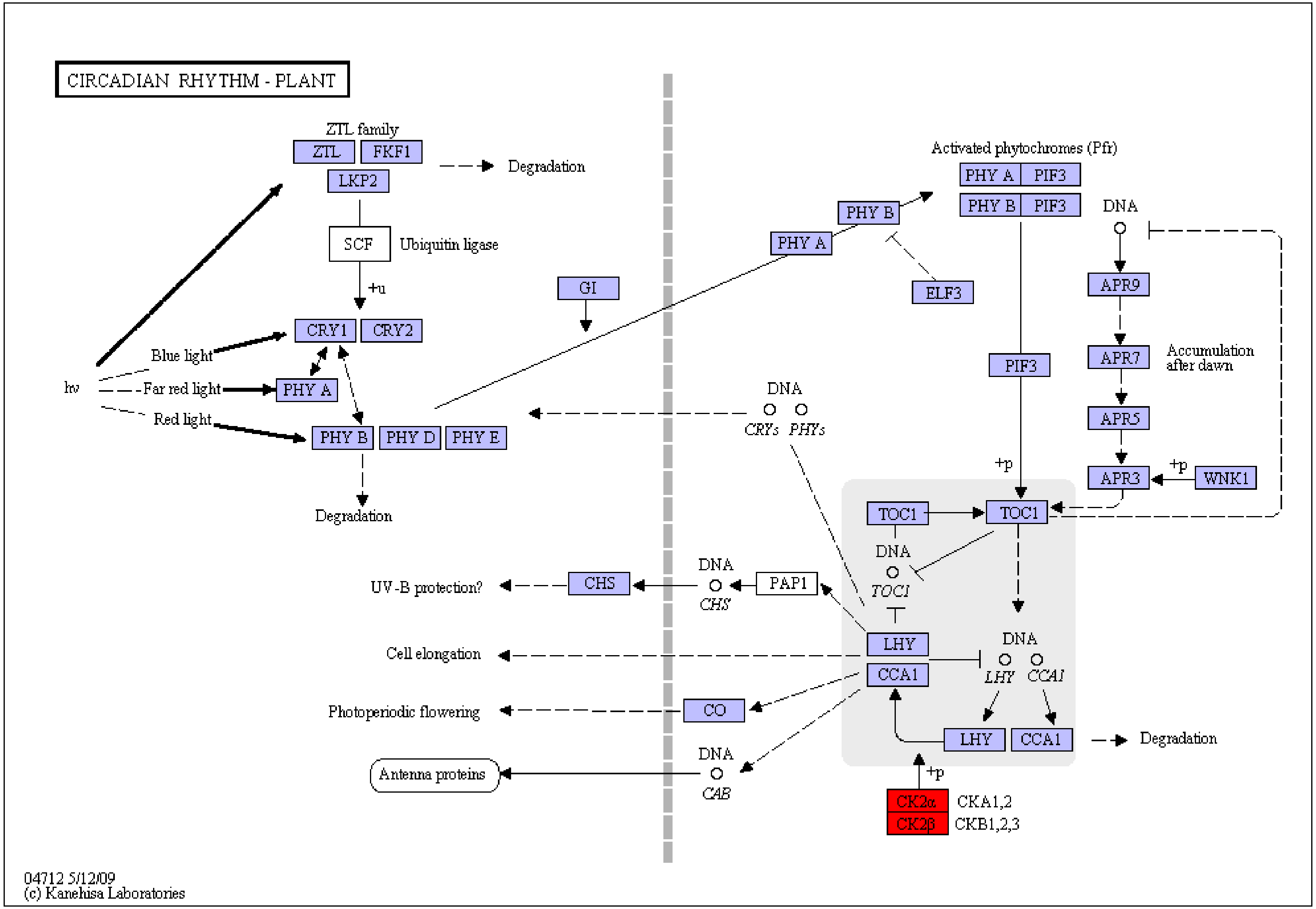

Figure 230: ko04712 Circadian rhythm - plant

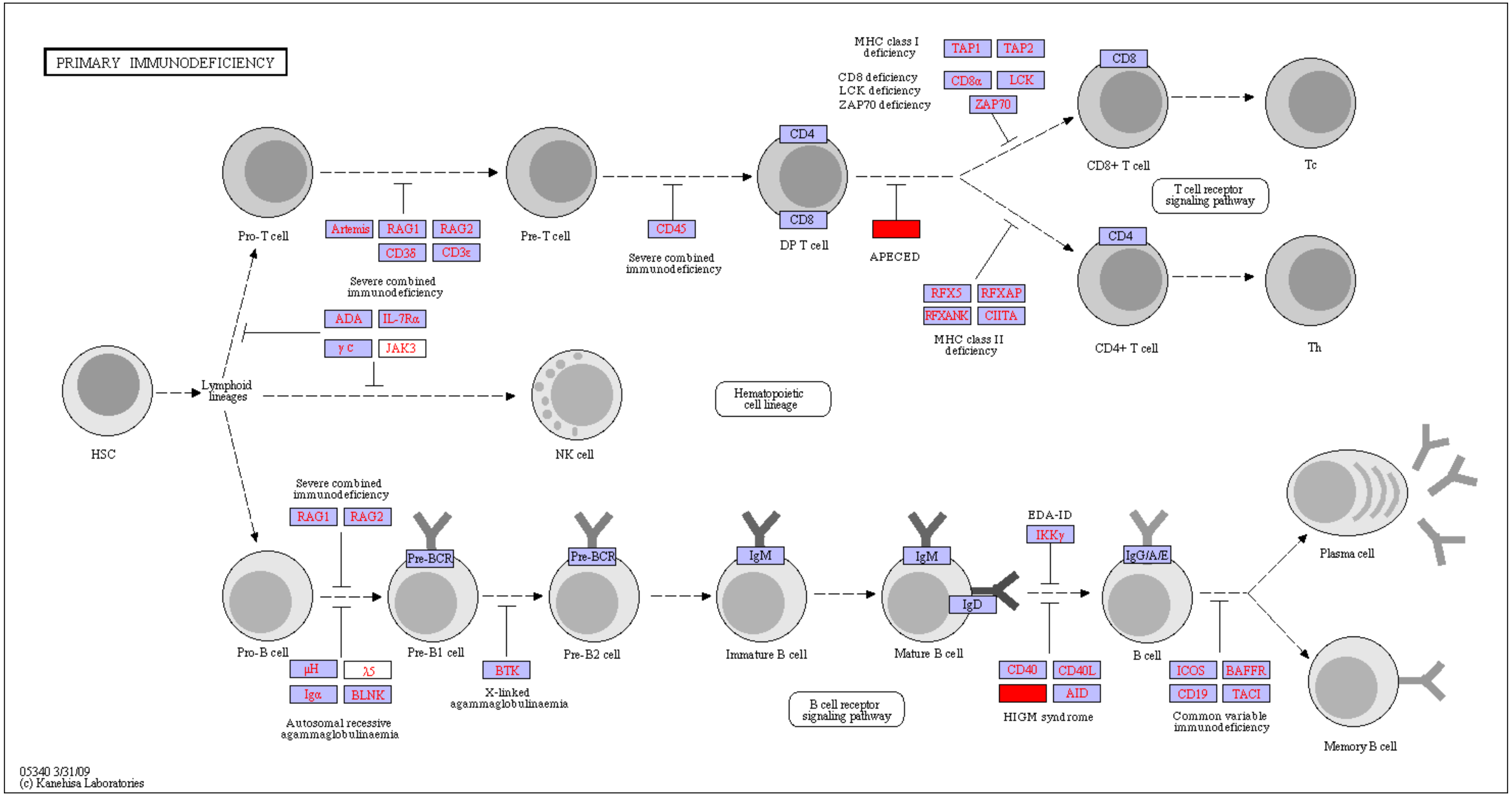

Figure 231: ko05340 Primary immunodeficiency

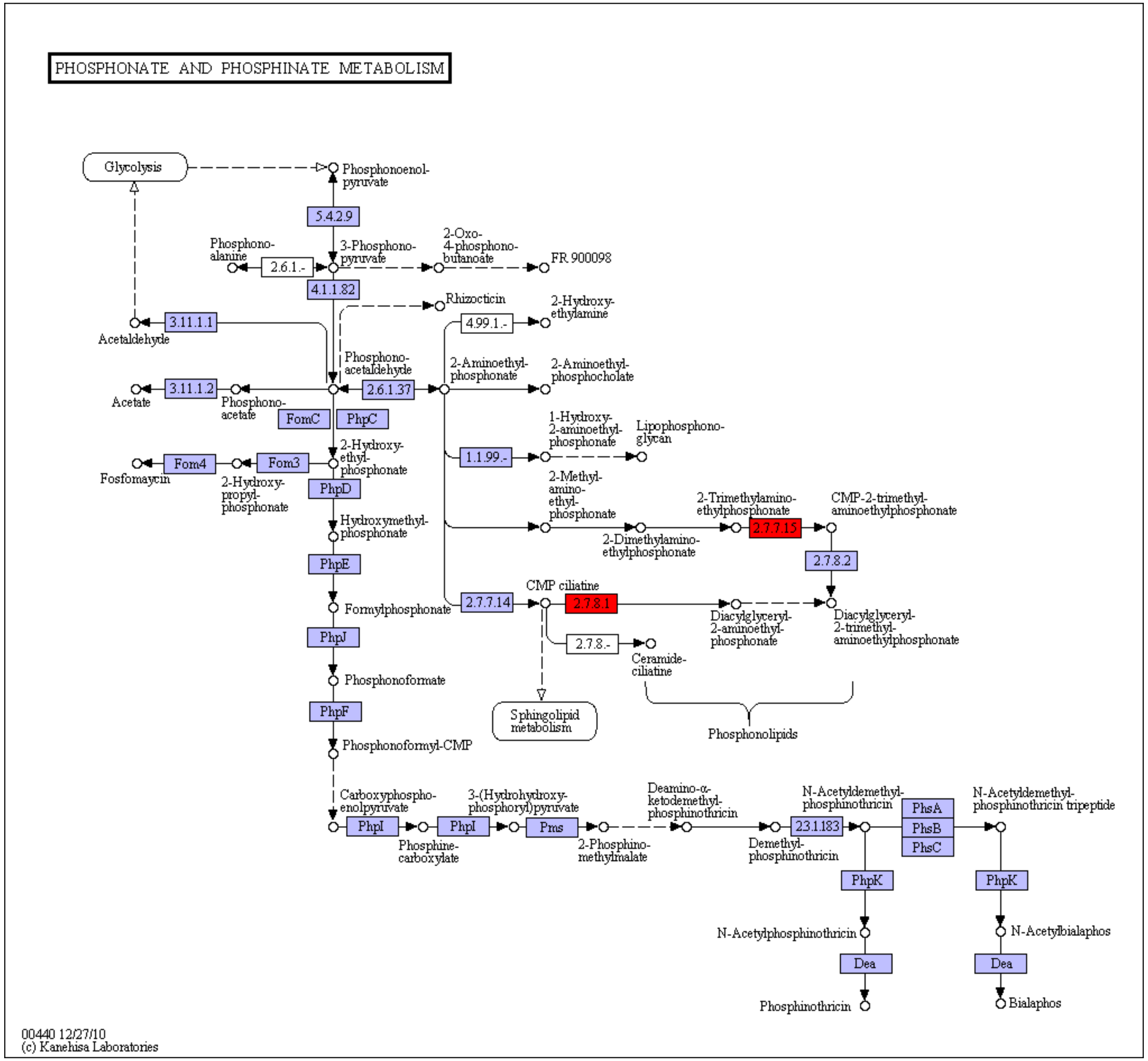

Figure 232: ko00440 Phosphonate and phosphinate metabolism

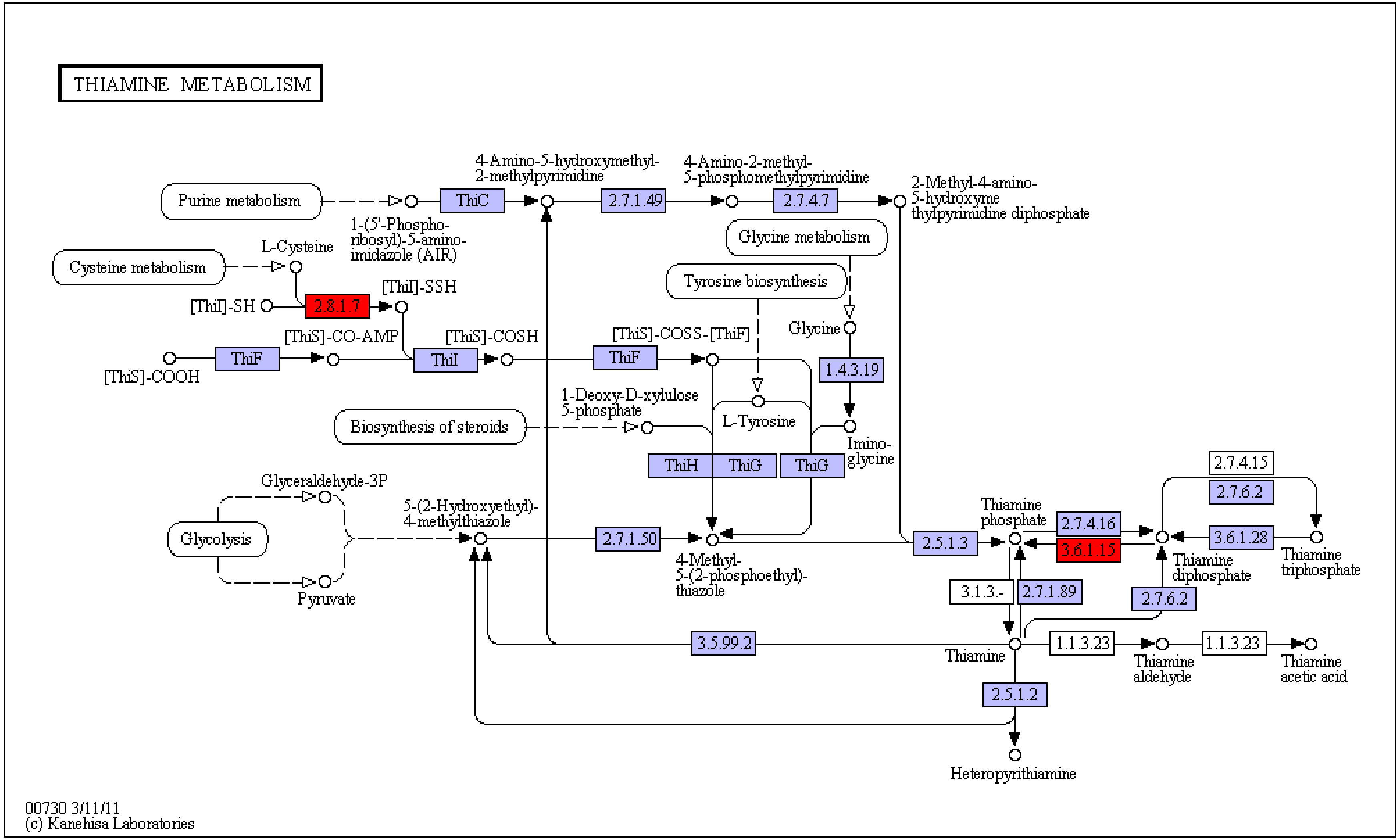

Figure 233: ko00730 Thiamine metabolism

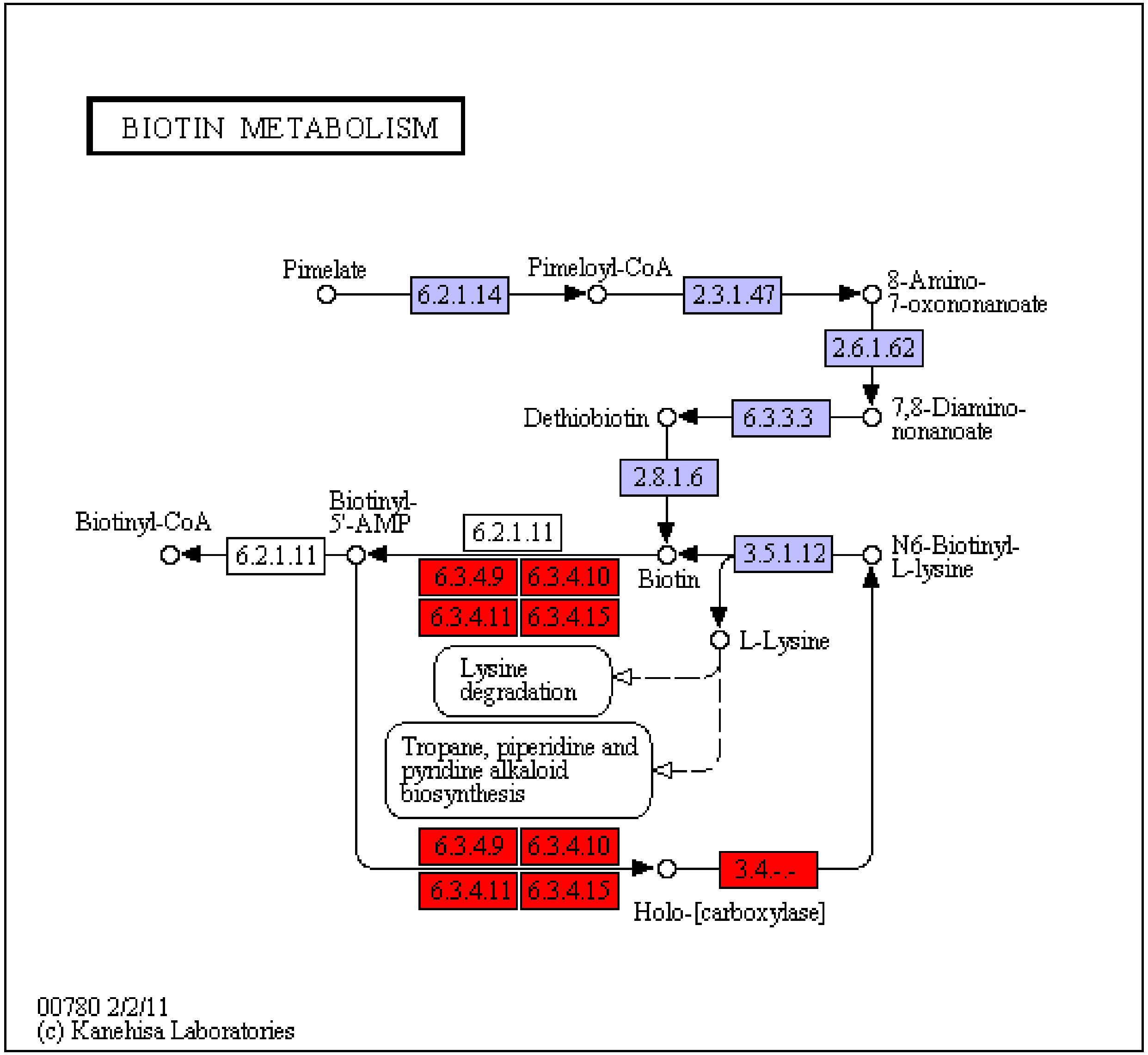

Figure 234: ko00780 Biotin metabolism

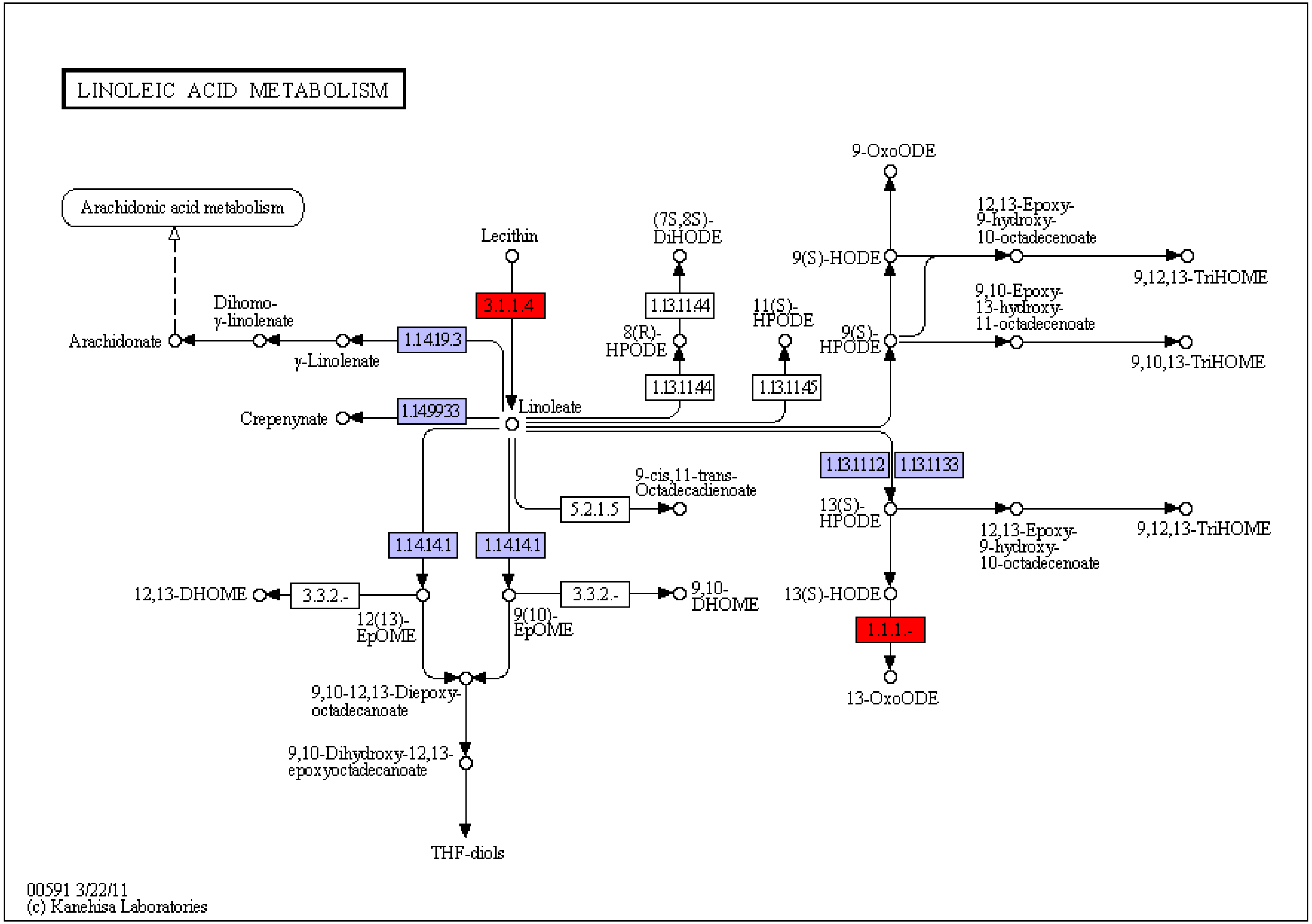

Figure 235: ko00591 Linoleic acid metabolism

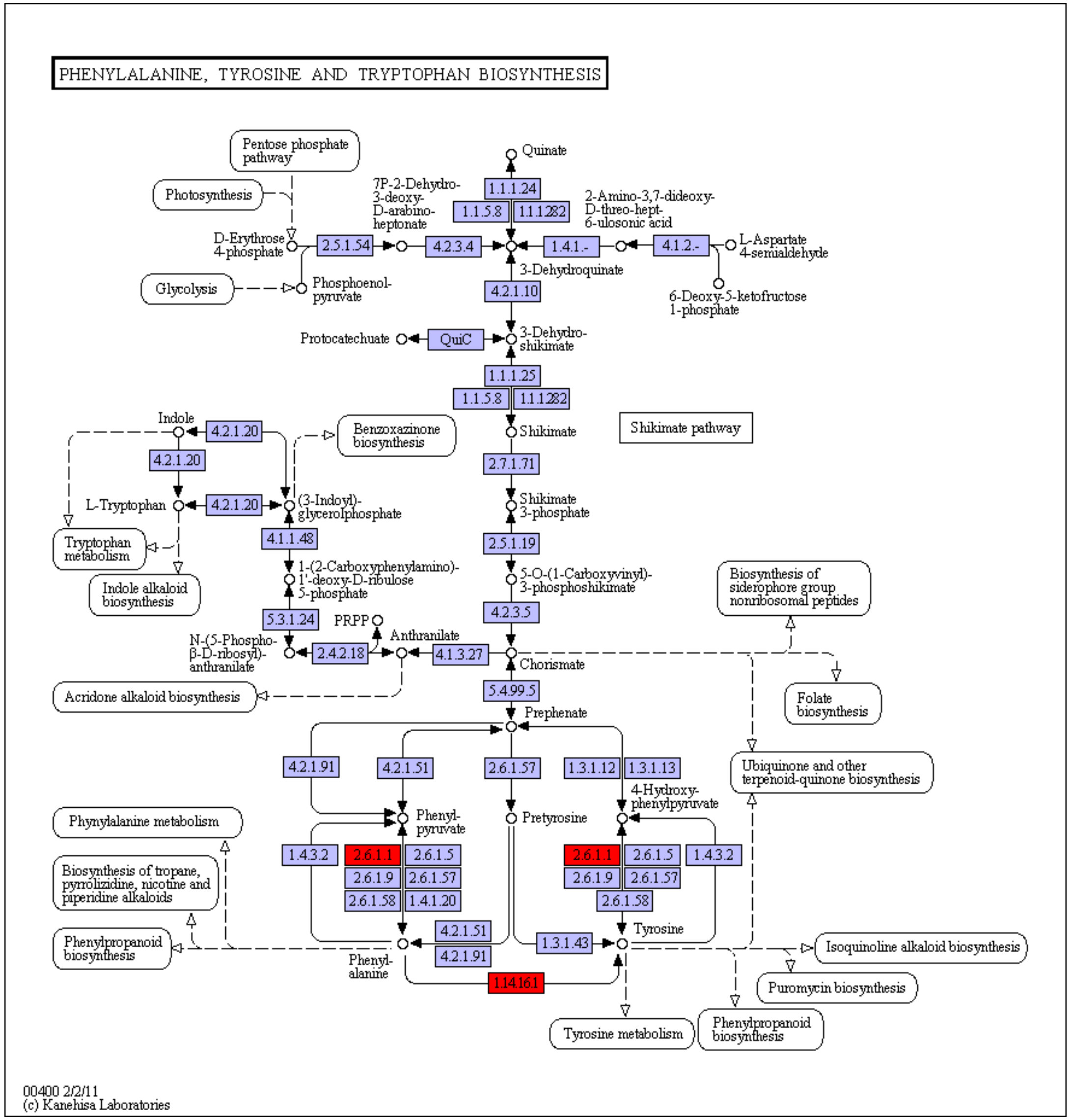

Figure 236: ko00400 Phenylalanine, tyrosine and tryptophan biosynthesis

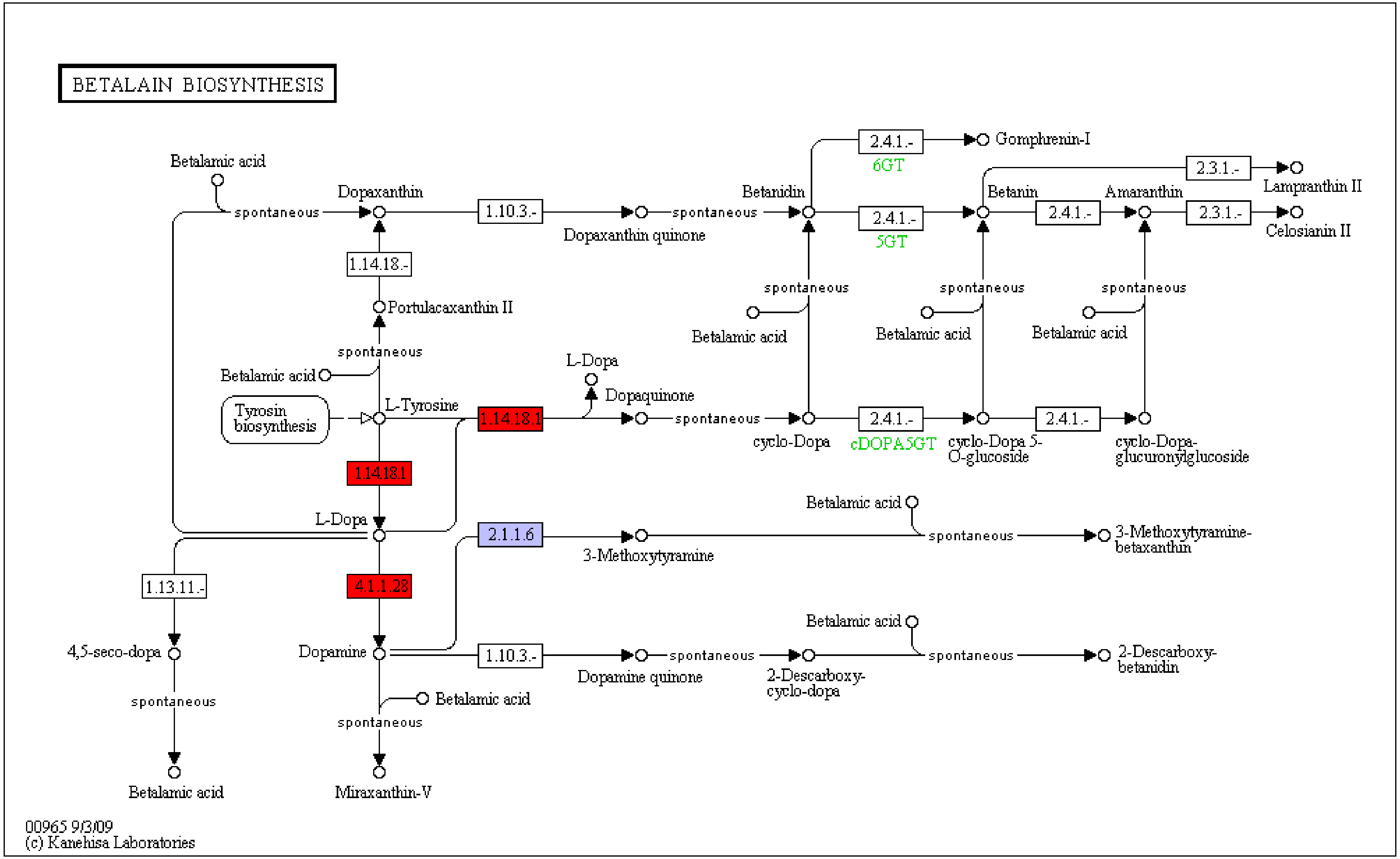

Figure 237: ko00965 Betalain biosynthesis

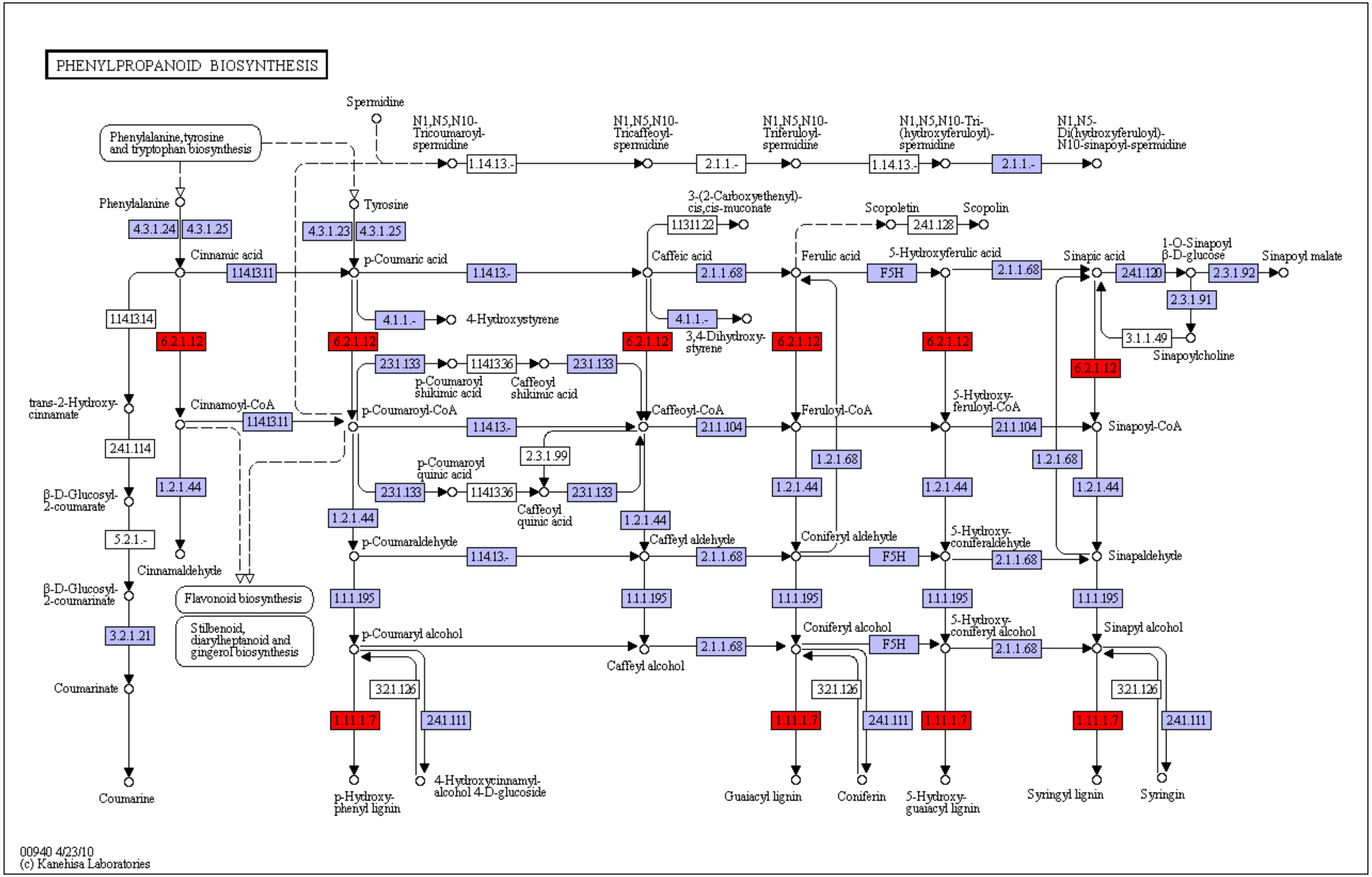

Figure 238: ko00940 Phenylpropanoid biosynthesis

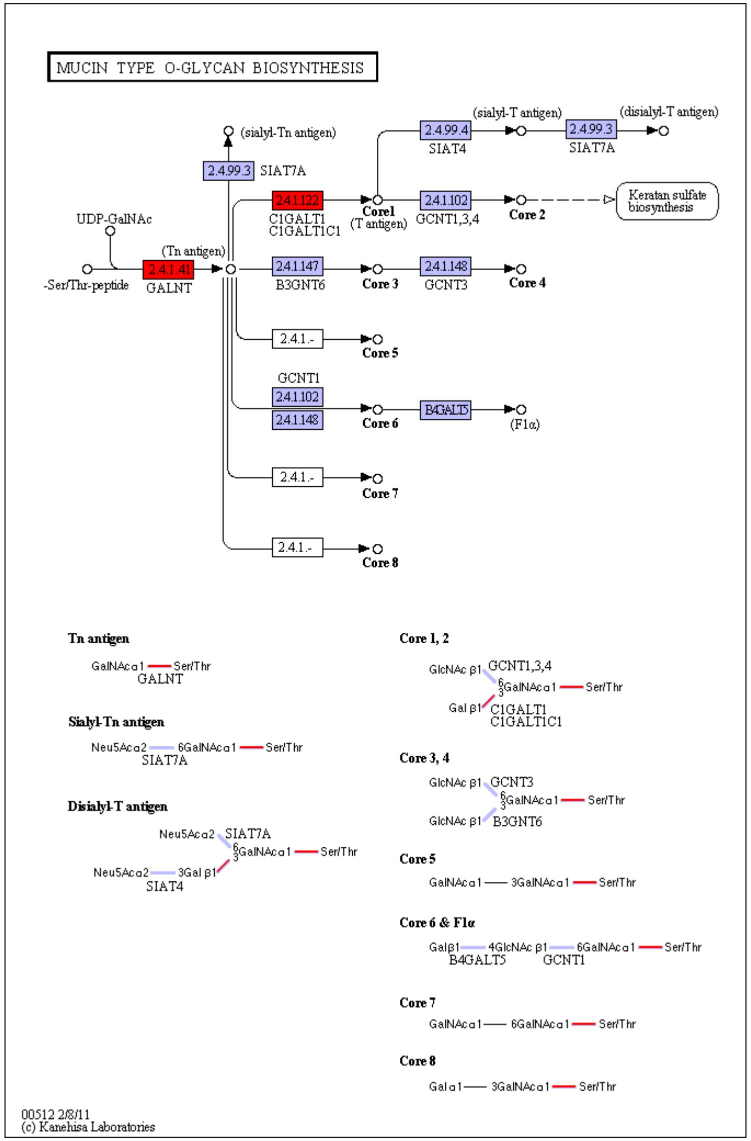

Figure 239: ko00512 Mucin type O-Glycan biosynthesis

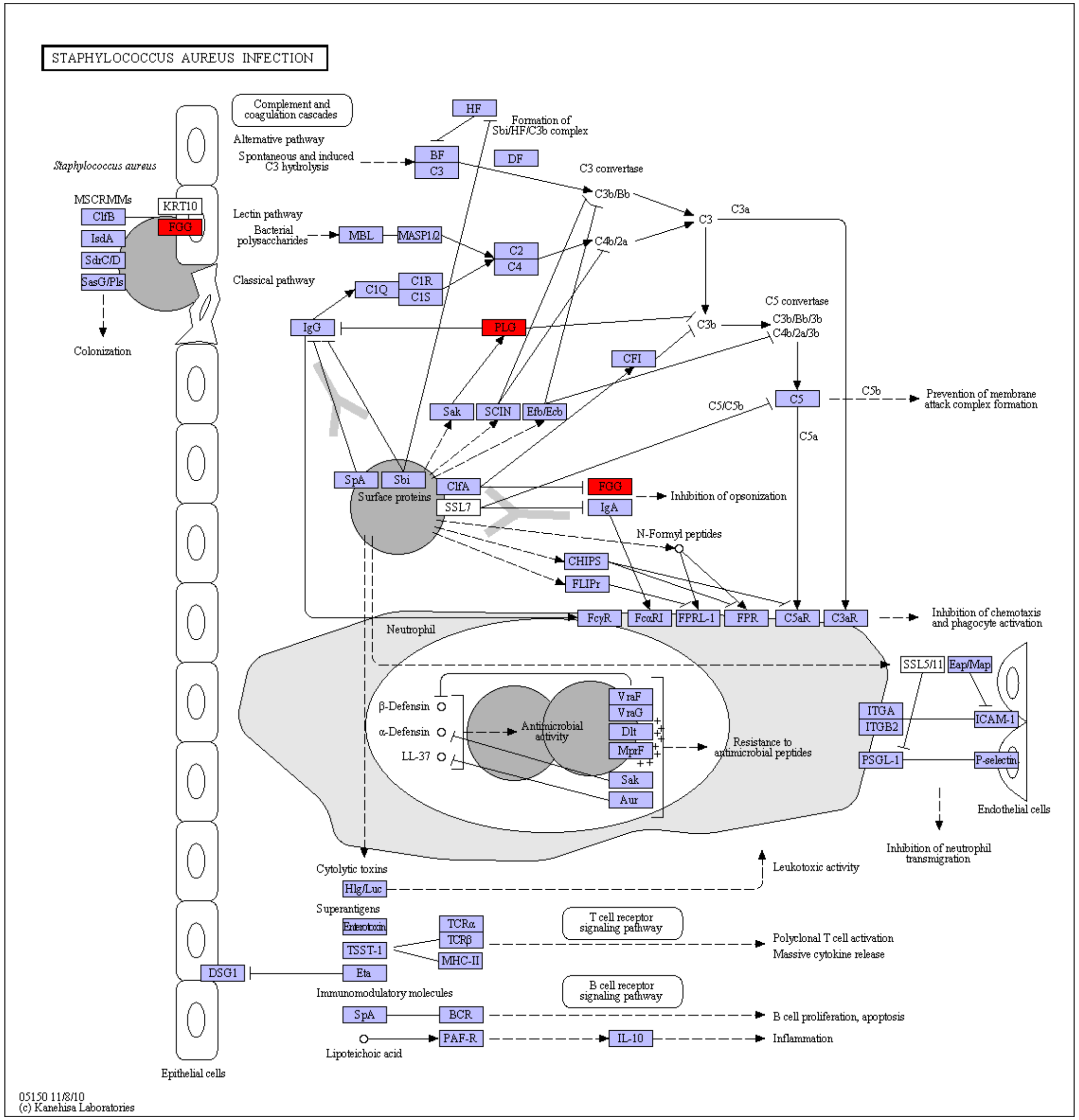

Figure 240: ko05150 Staphylococcus aureus infection

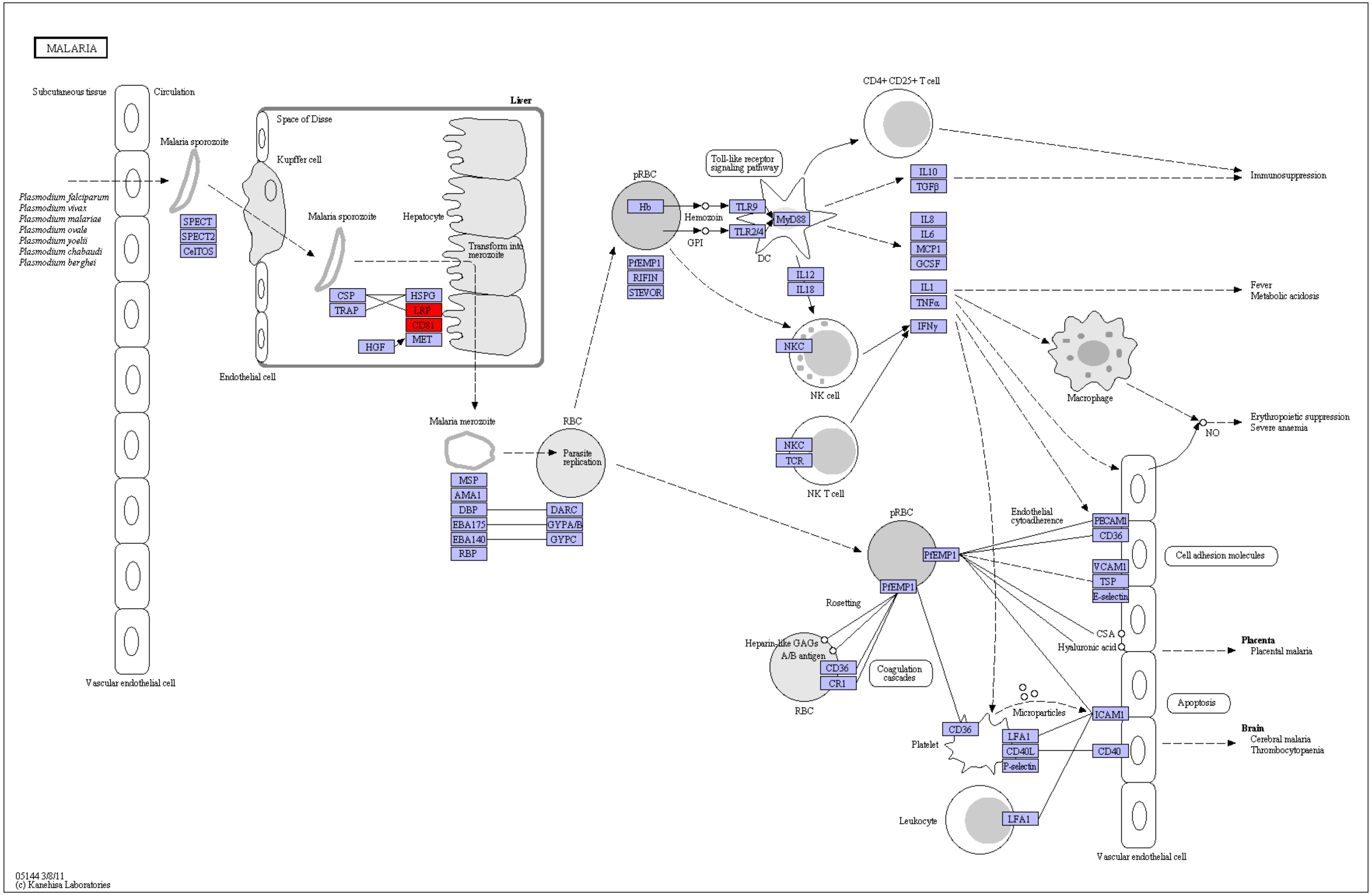

Figure 241: ko05144 Malaria

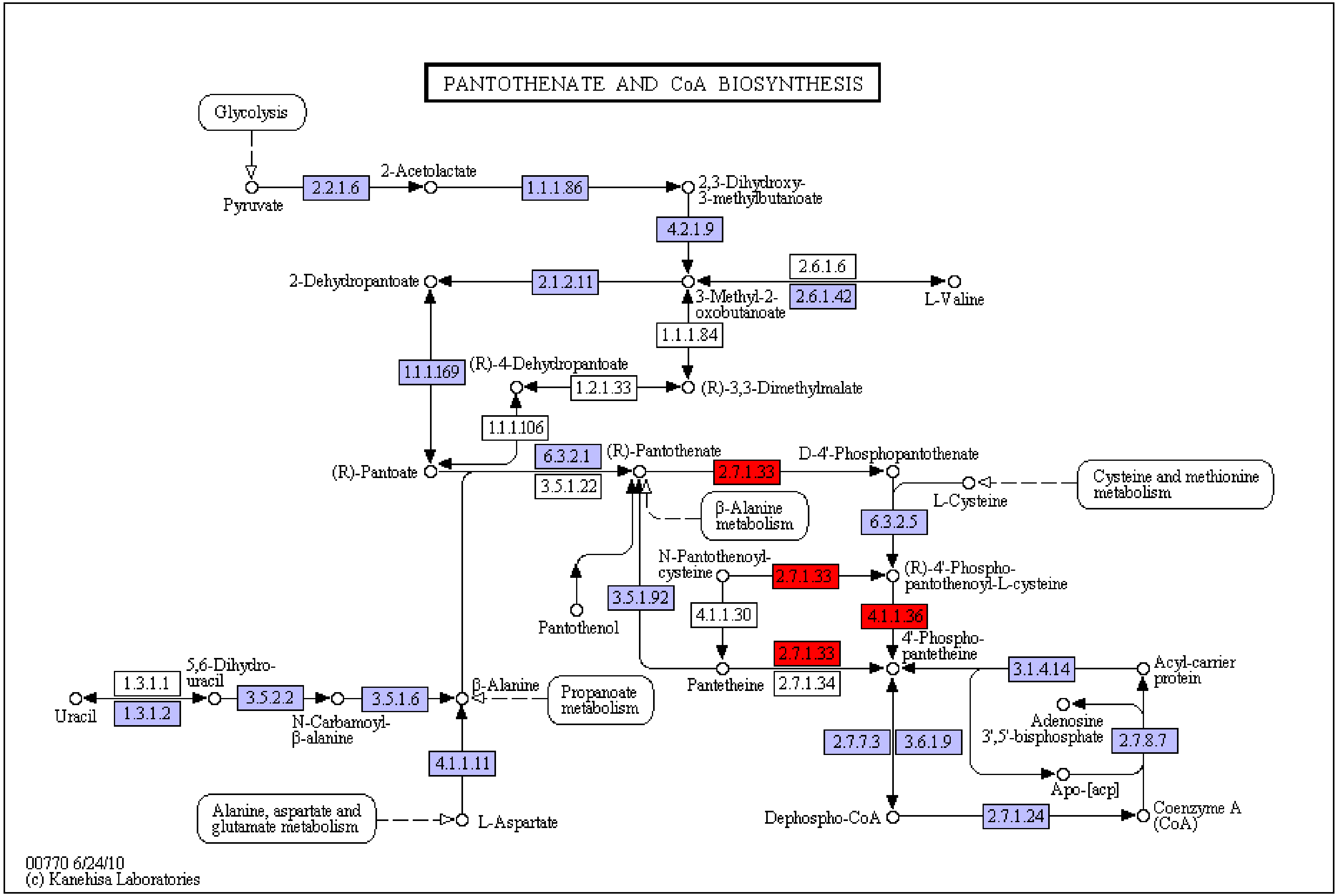

Figure 242: ko00770 Pantothenate and CoA biosynthesis

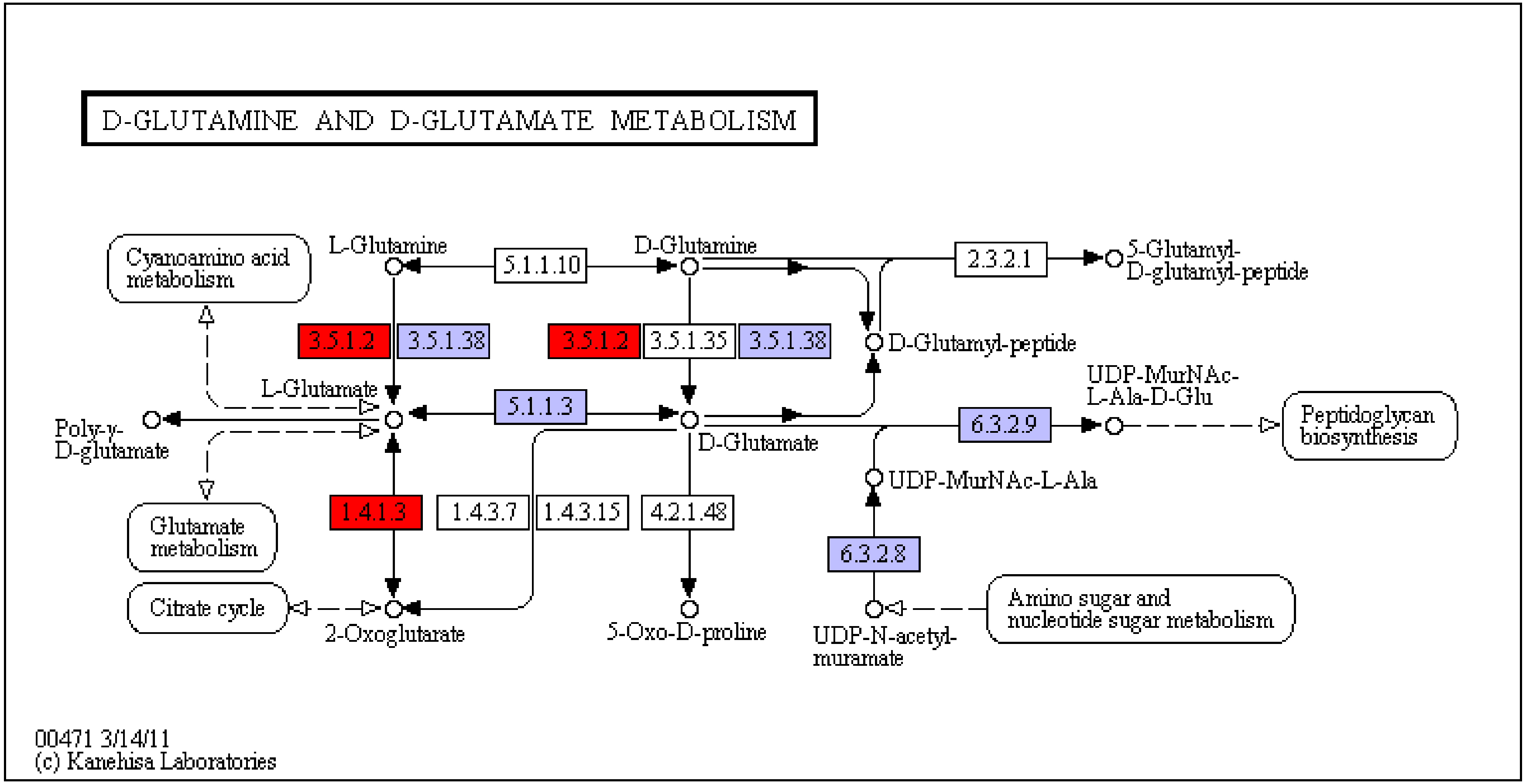

Figure 243: ko00471 D-Glutamine and D-glutamate metabolism

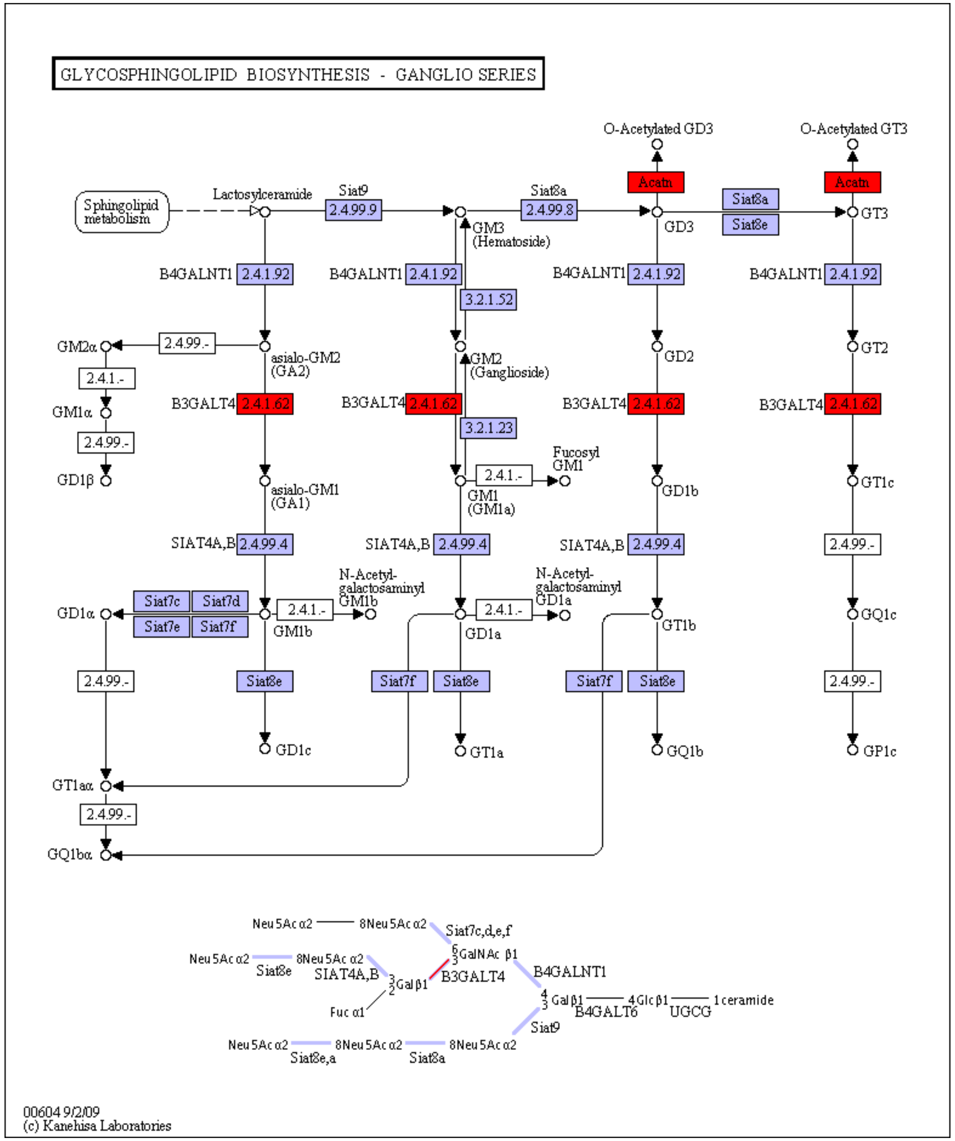

Figure 244: ko00604 Glycosphingolipid biosynthesis - ganglio series

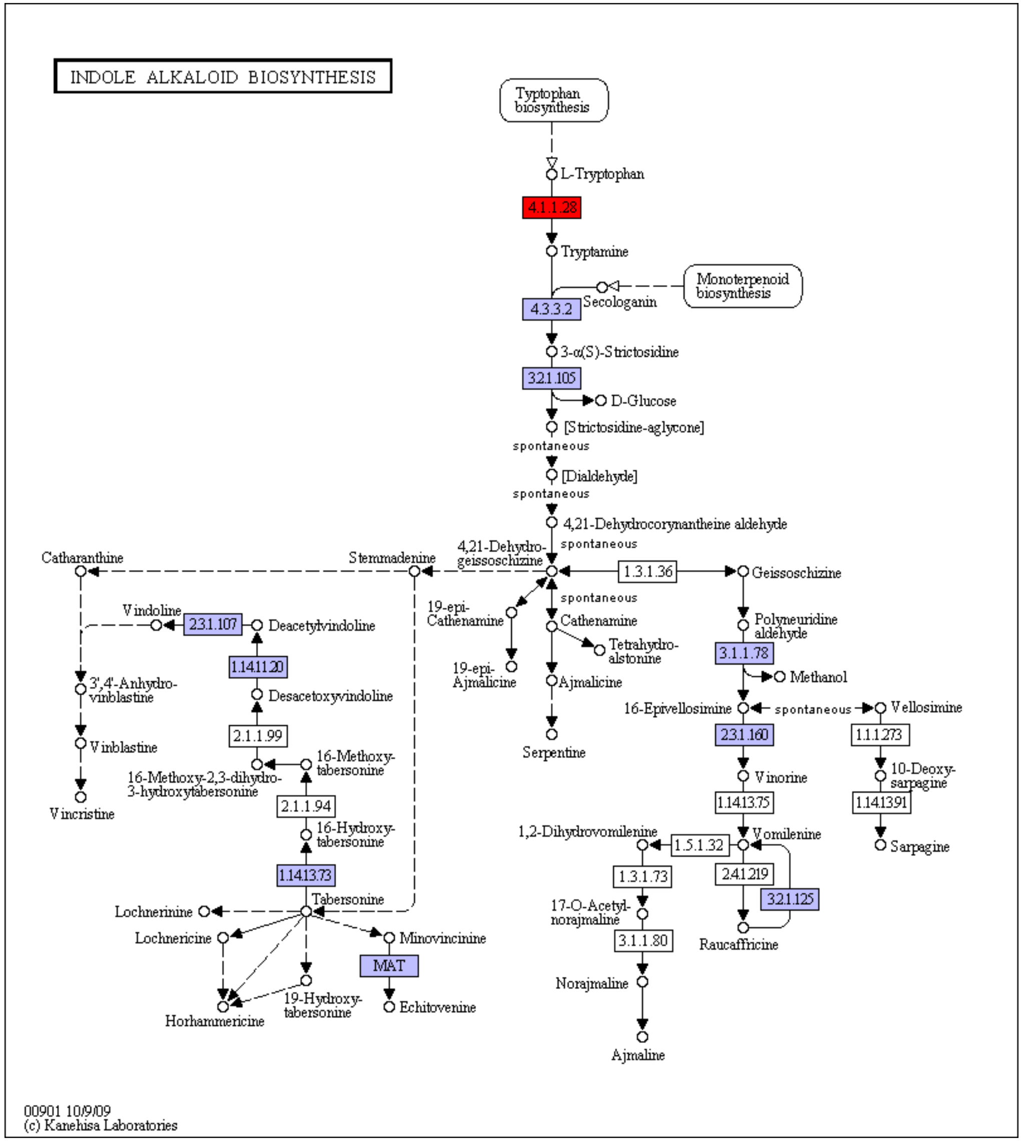

Figure 245: ko00901 Indole alkaloid biosynthesis

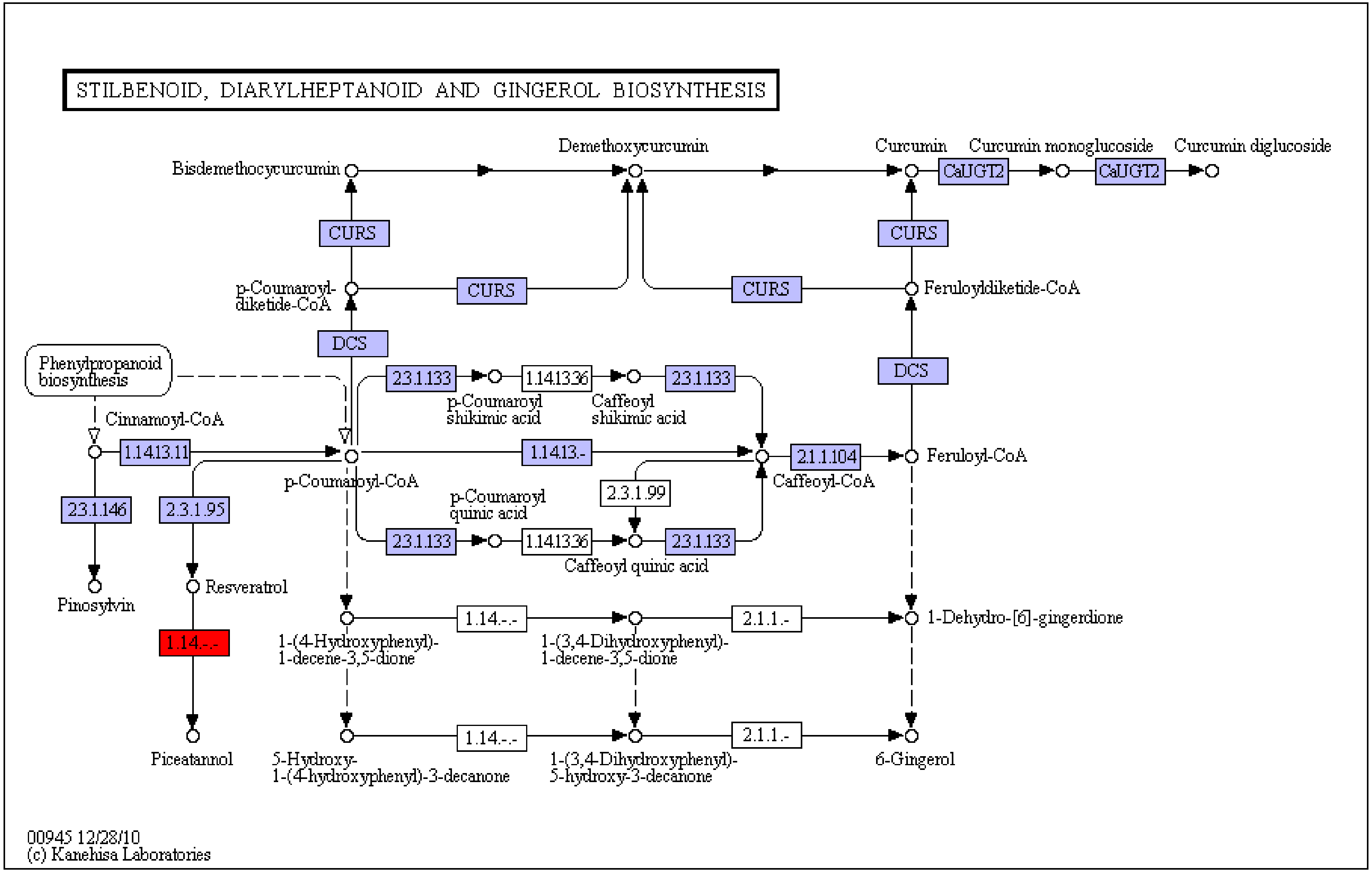

Figure 246: ko00945 Stilbenoid, diarylheptanoid and gingerol biosynthesis

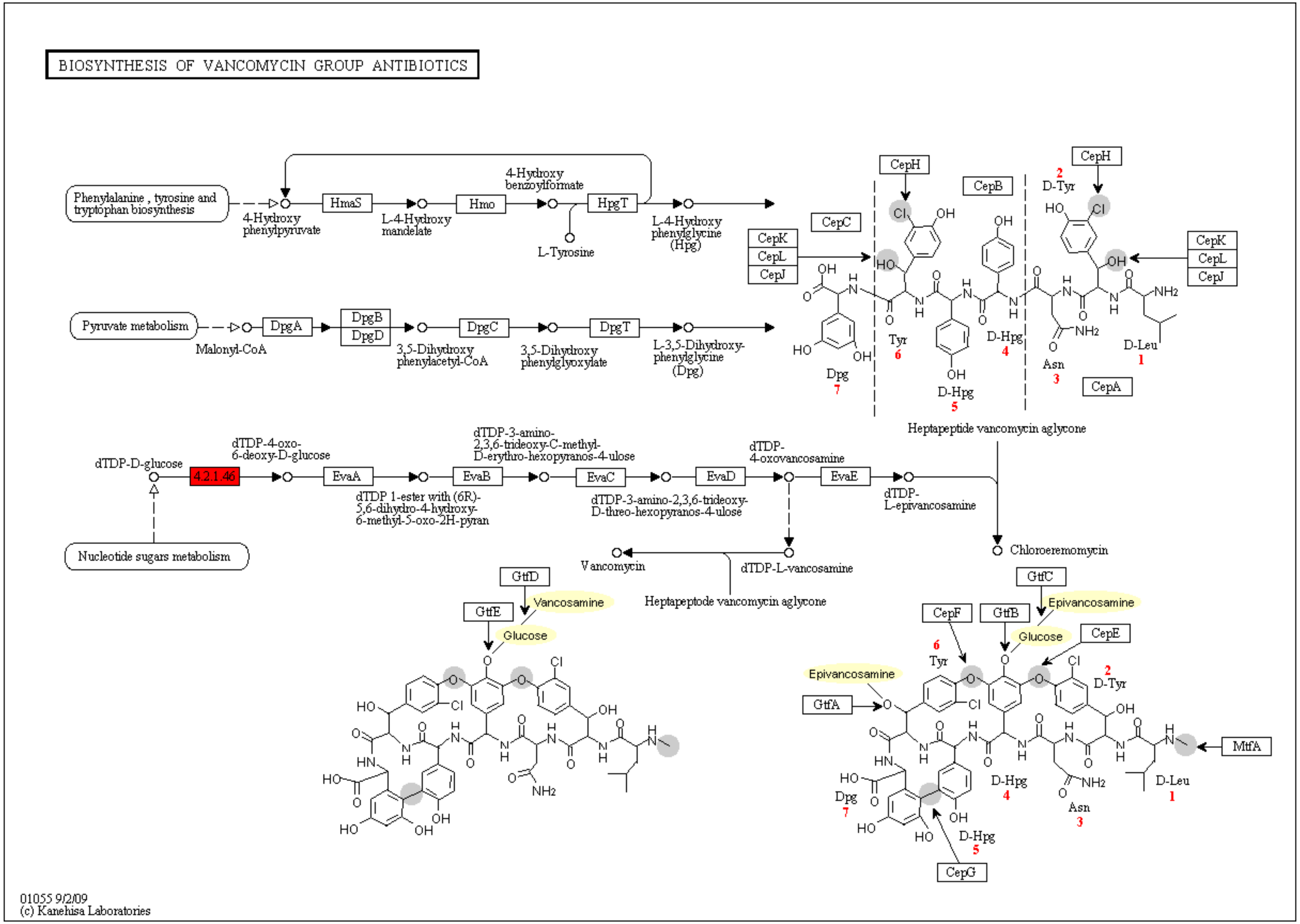

Figure 247: ko01055 Biosynthesis of vancomycin group antibiotics

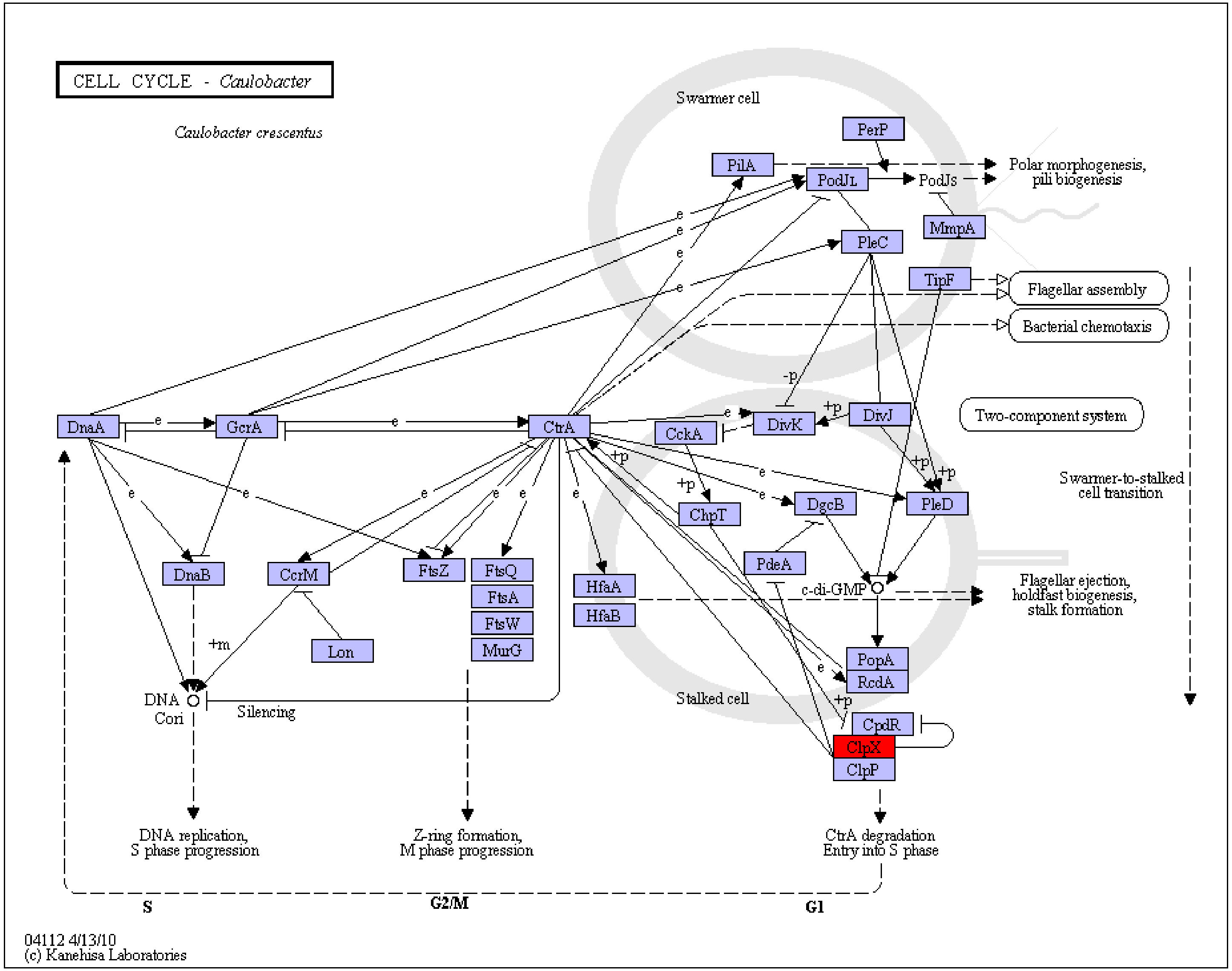

Figure 248: ko04112 Cell cycle - Caulobacter

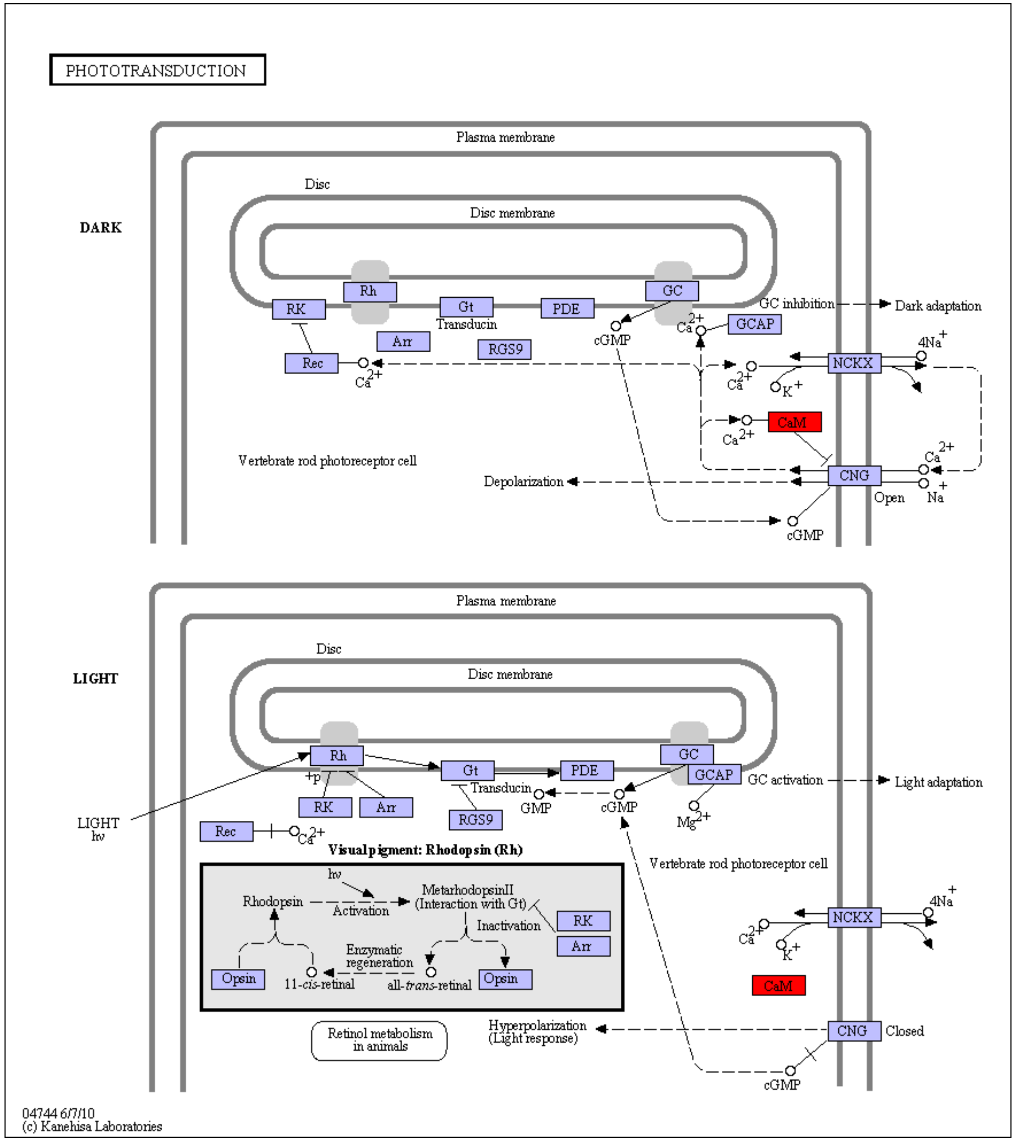

Figure 249: ko04744 Phototransduction

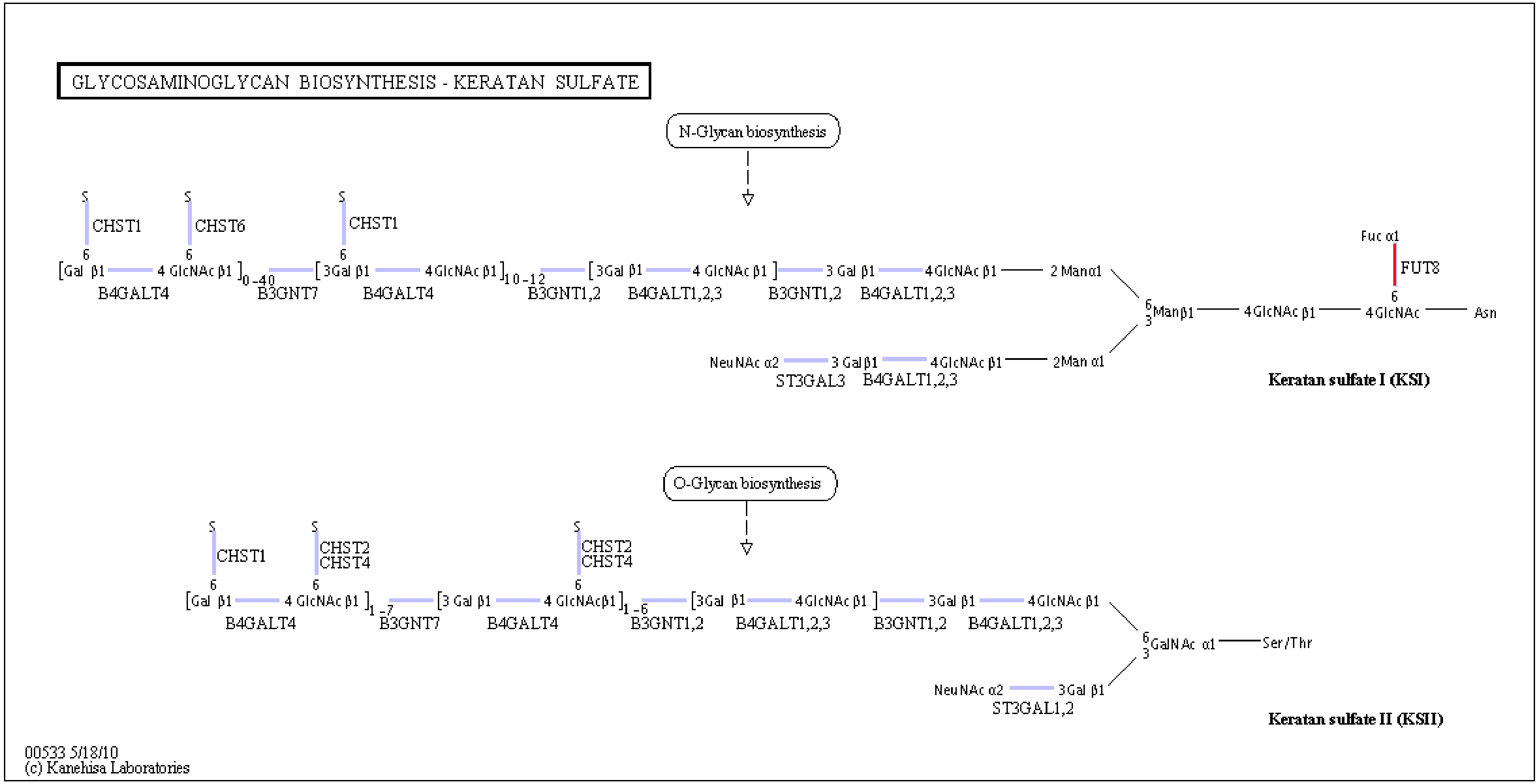

Figure 250: ko00533 Glycosaminoglycan biosynthesis - keratan sulfate

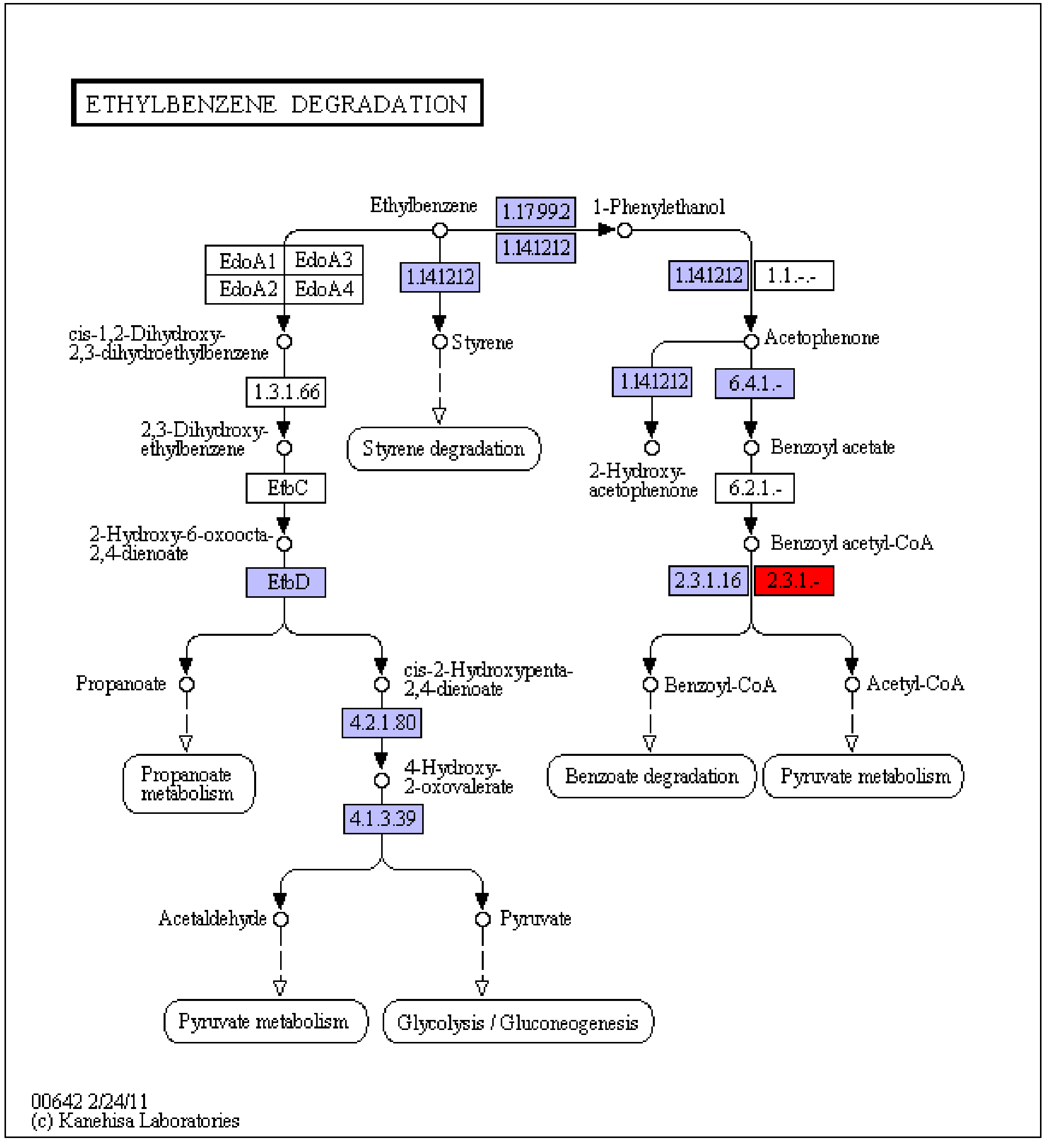

Figure 251: ko00642 Ethylbenzene degradation

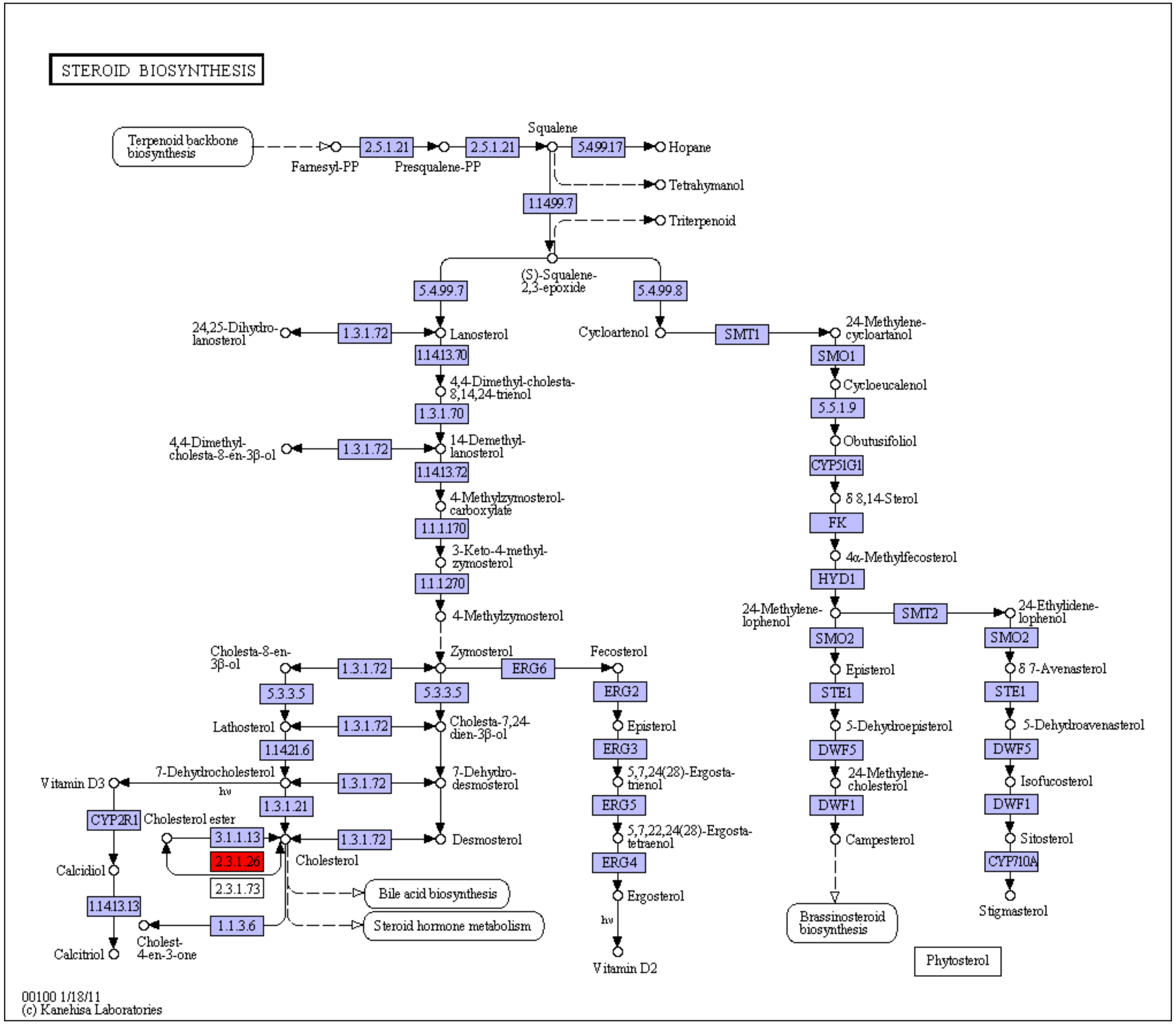

Figure 252: ko00100 Steroid biosynthesis

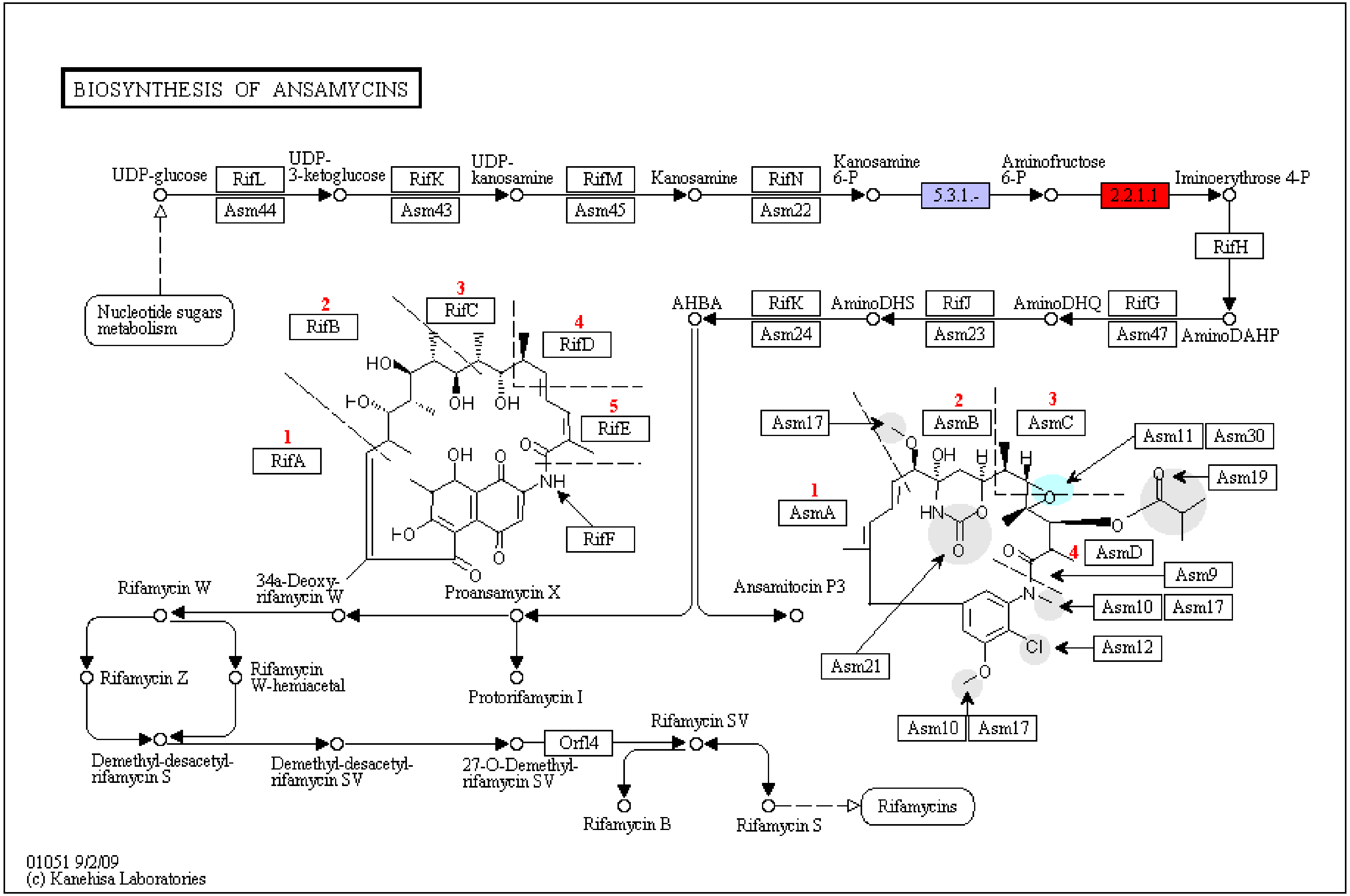

Figure 253: ko01051 Biosynthesis of ansamycins

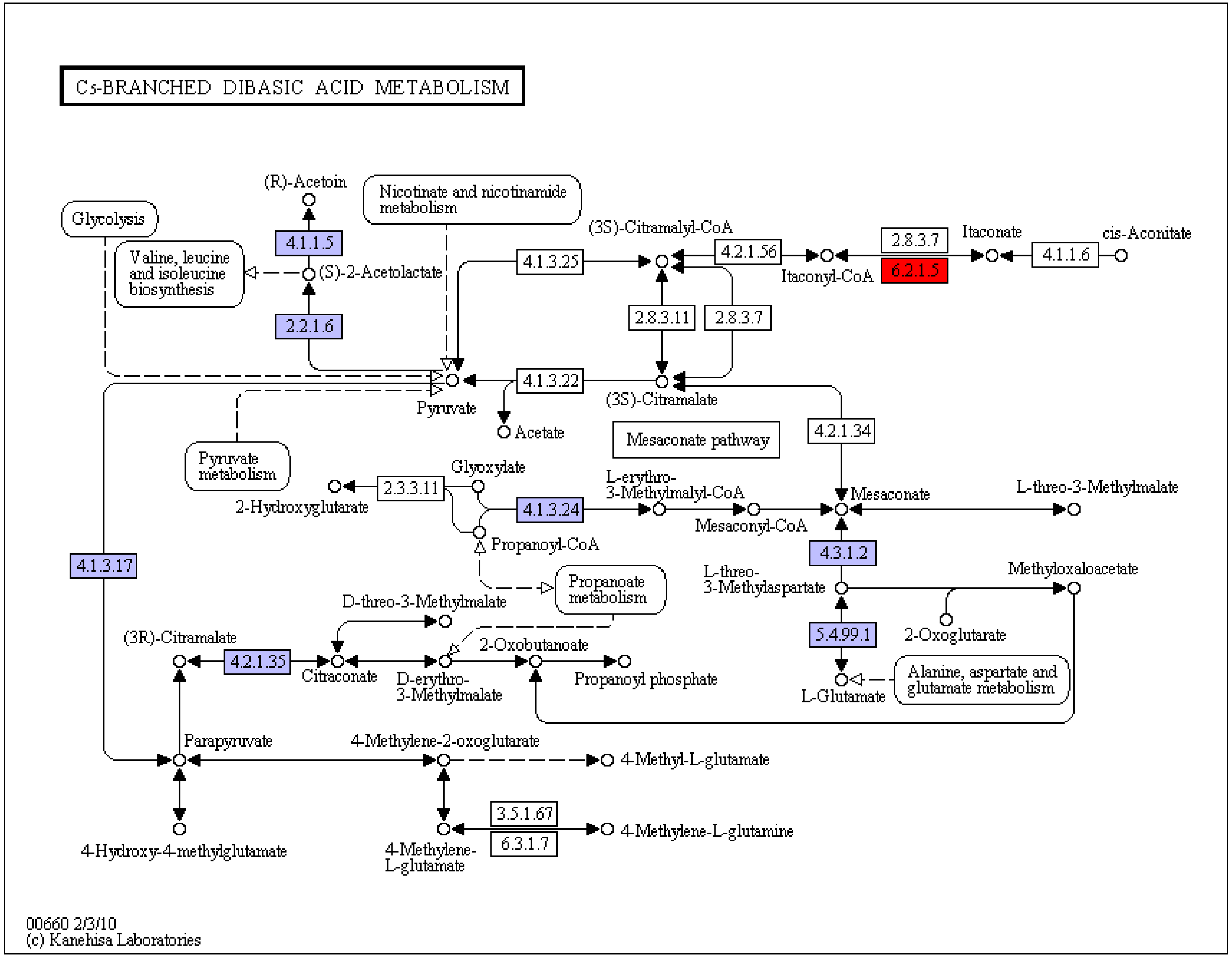

Figure 254: ko00660 C5-Branched dibasic acid metabolism

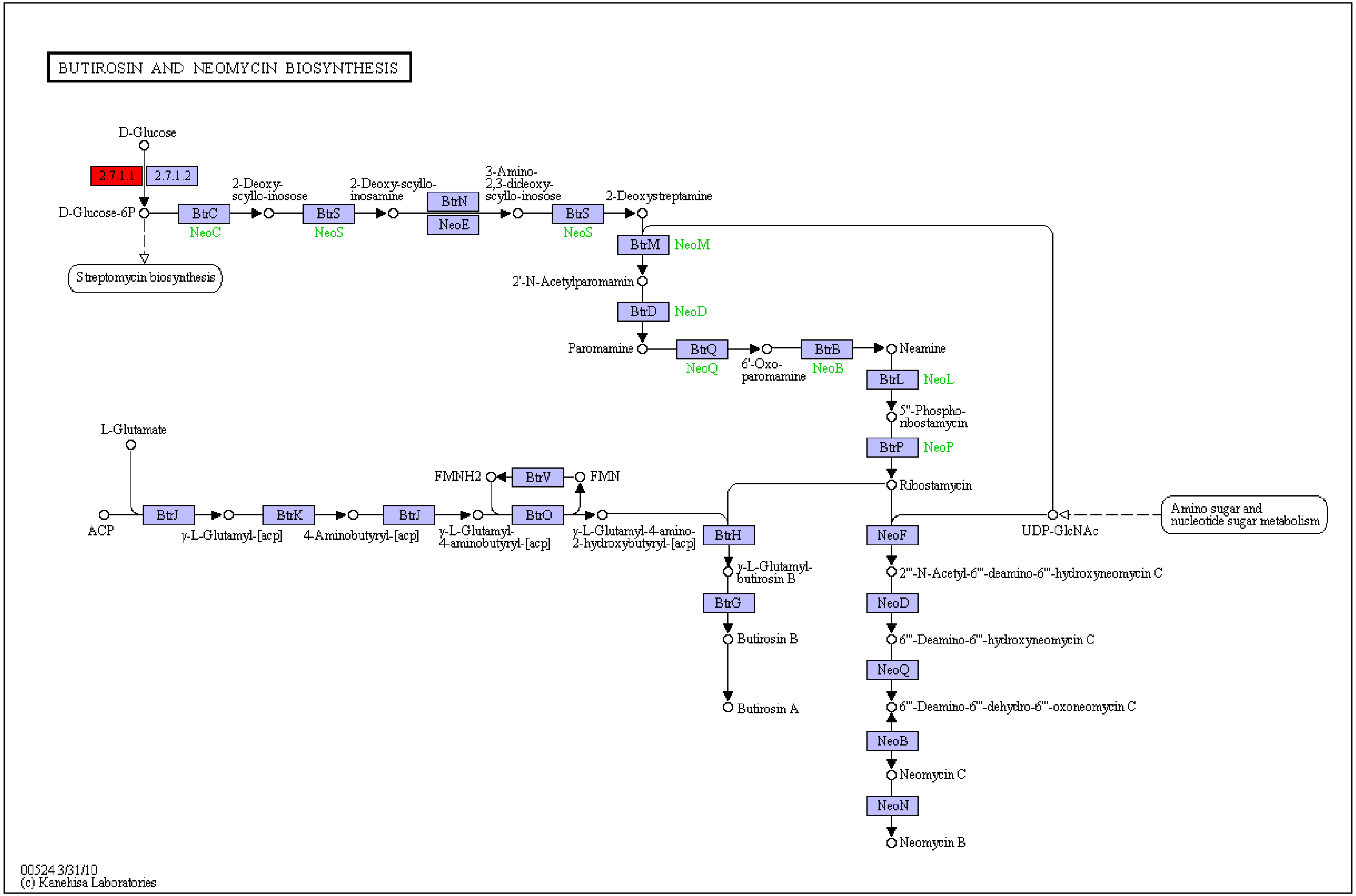

Figure 255: ko00524 Butirosin and neomycin biosynthesis

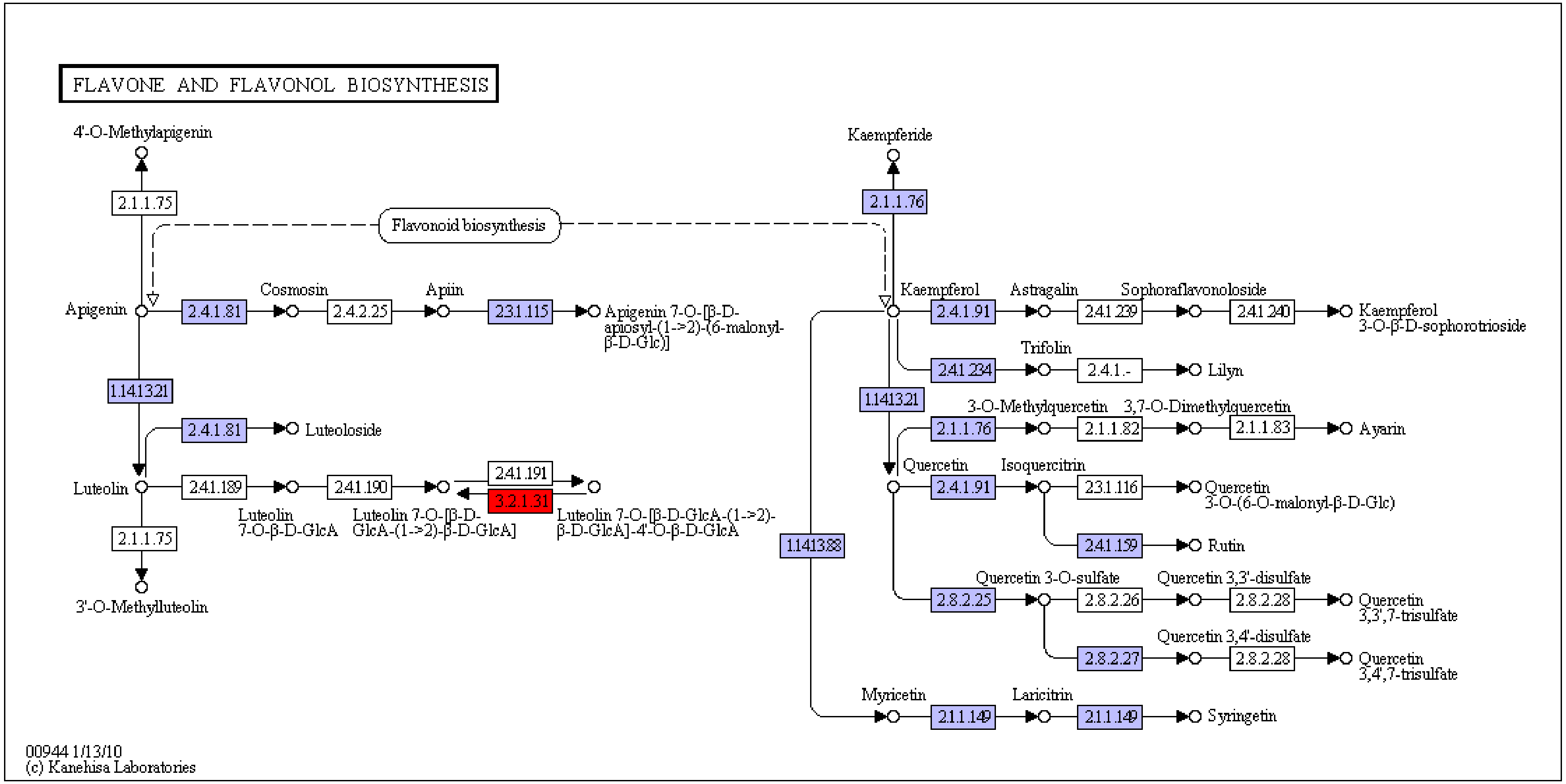

Figure 256: ko00944 Flavone and flavonol biosynthesis

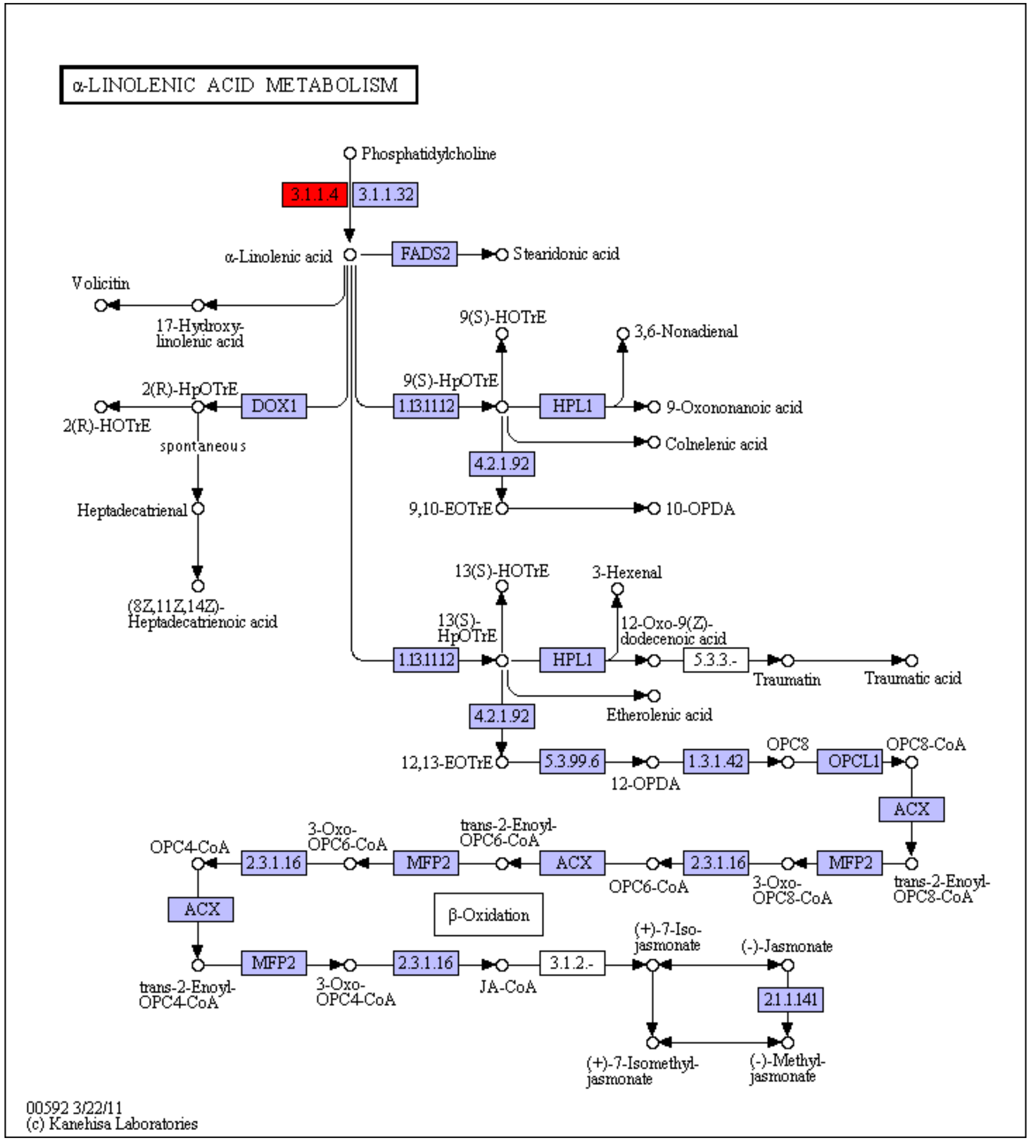

Figure 257: ko00592 alpha-Linolenic acid metabolism

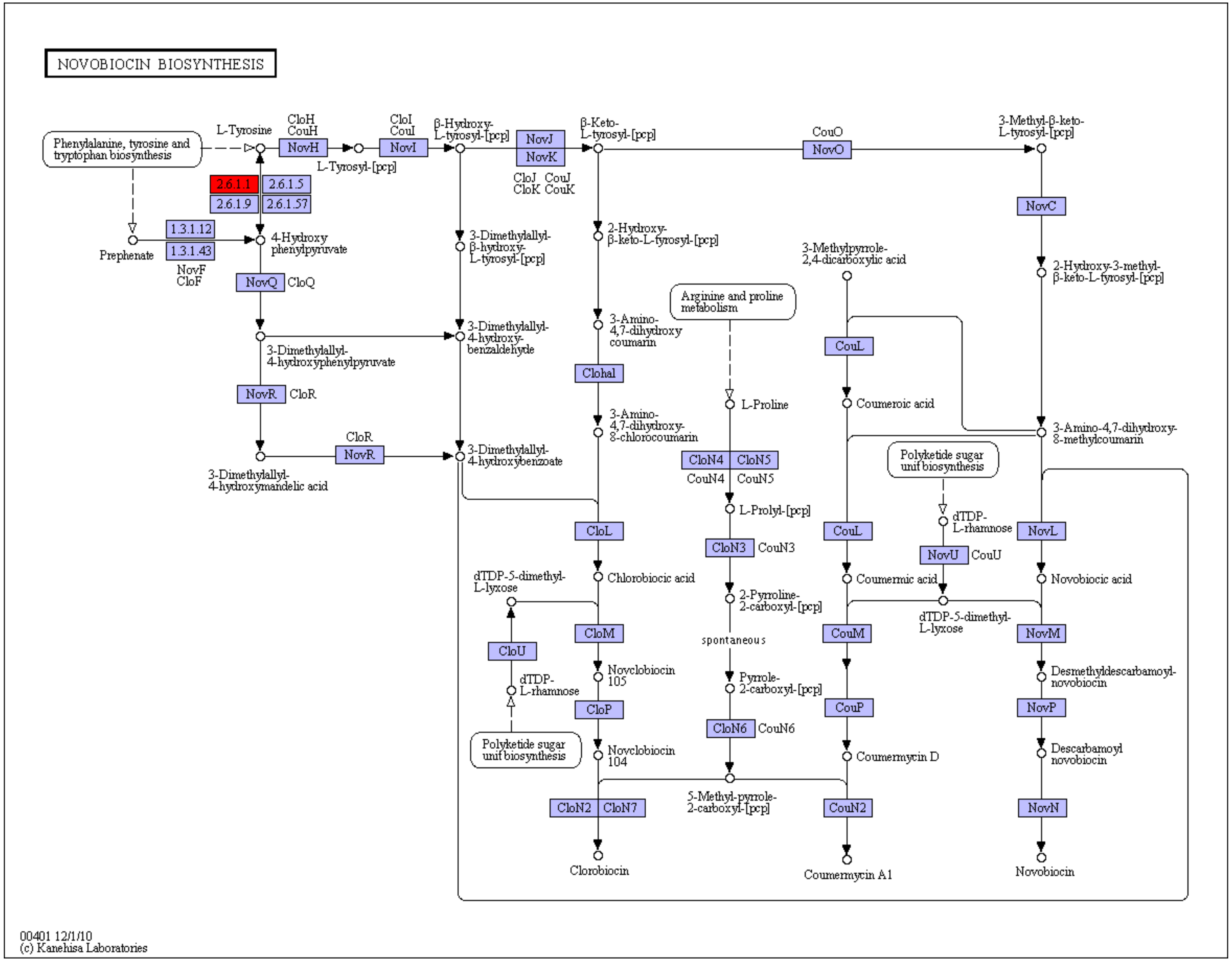

Figure 258: ko00401 Novobiocin biosynthesis

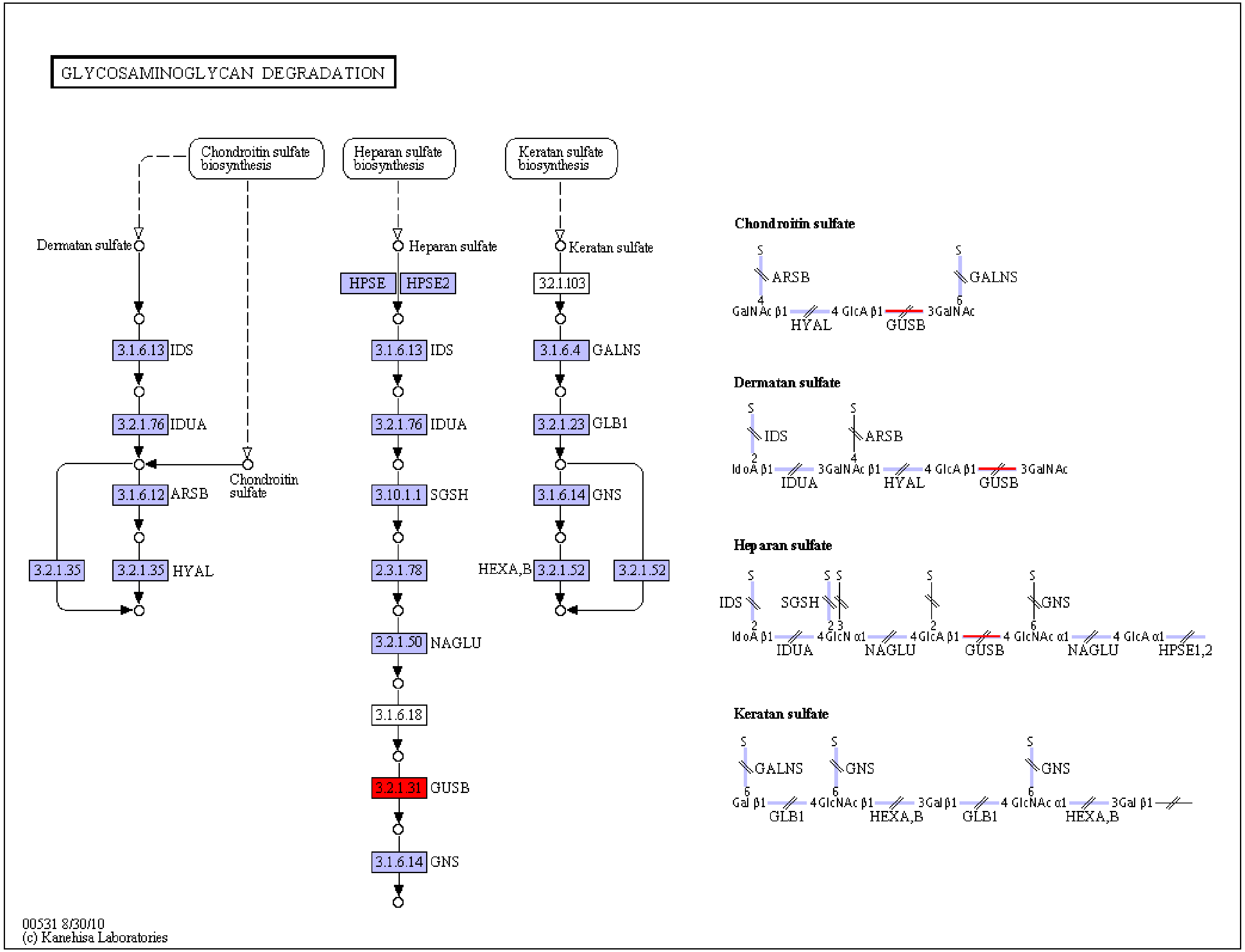

Figure 259: ko00531 Glycosaminoglycan degradation

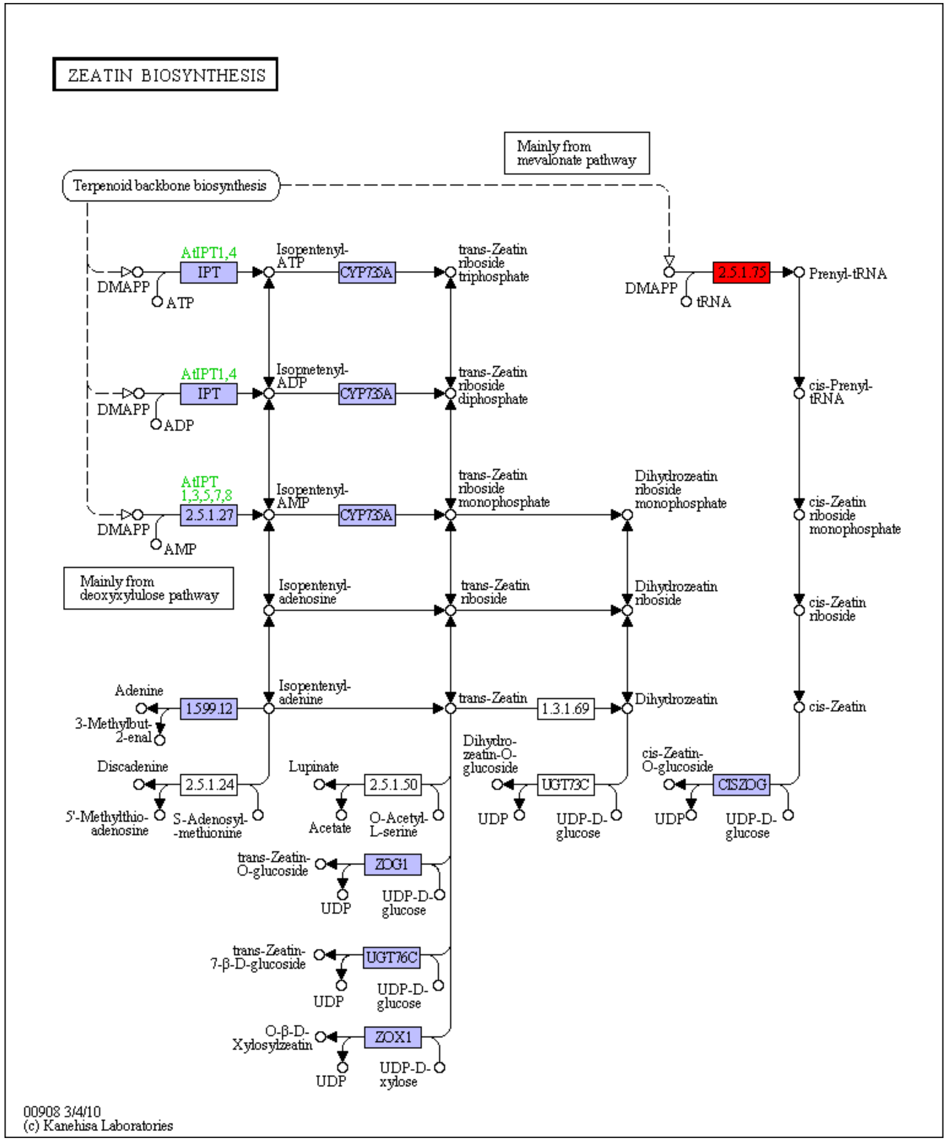

Figure 260: ko00908 Zeatin biosynthesis

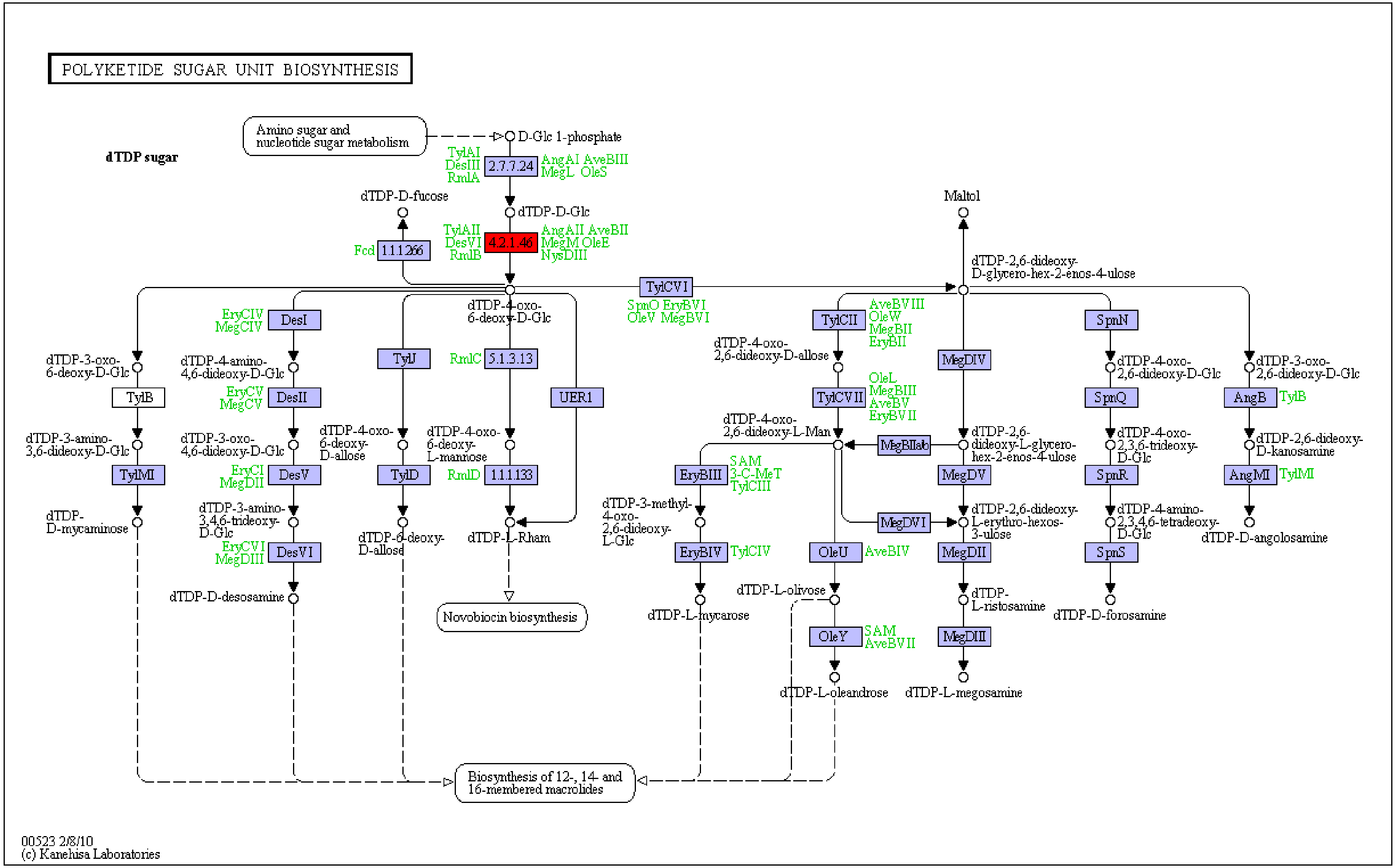

Figure 261: ko00523 Polyketide sugar unit biosynthesis

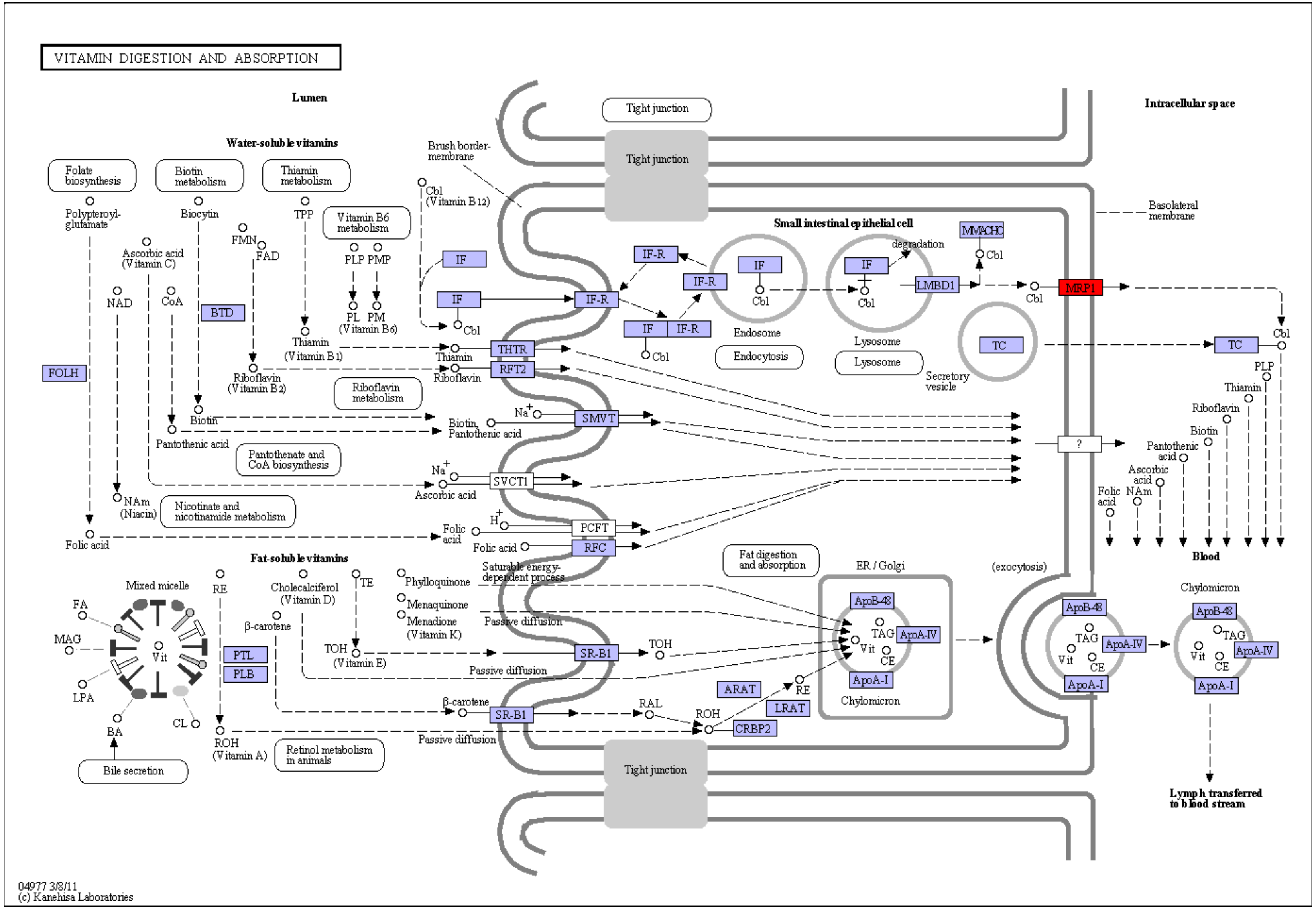

Figure 262: ko04977 Vitamin digestion and absorption
