# Supplementary material for: The Transcriptome of Trichuris suis – First Molecular Insights into a Parasite with Curative Properties for Key Immune Diseases of Humans
Source: PLoS One. 2011 Aug 24;6(8):e23590. doi: 10.1371/journal.pone.0023590 (PMC3160910; doi:10.1371/journal.pone.0023590)
Supplement: Table S1 — The twenty most abundant proteins encoded in the transcriptome of Trichuris suis, following conceptual translation of individual contigs. (DOC) [file pone.0023590.s002.doc]

**Table S1**. The twenty most abundant proteins encoded in the transcriptome of *Trichuris suis*, following conceptual translation of individual contigs.

| Contig | Description | RPKM log_2_ ratio | InterPro term(s) (code) | Gene Ontology term(s) |  |  | KEGG pathway term(s) (code) |
| --- | --- | --- | --- | --- | --- | --- | --- |
|  |  |  |  | Biological process | Cellular component | Molecular function |  |
| Contig146 | UniProt: VIT5_CAEEL Protein ID: AAA835587.1 (*Caenorhabditis elegans* vitellogenin) | 5.86 | Lipid transport protein (IPR001747); | Lipid transport |  | Lipid transporter activity |  |
|  |  |  | Vitellinogen (IPR011030) |  |  |  |  |
| Contig288 | UniProt:Q21650_CAEEL Protein ID: AAA50720.2 (*Caenorhabditis elegans* unnamed protein) | 5.81 | Chitin-binding domain (IPR002557) | Chitin metabolic process | Extracellular region | Chitin binding |  |
| Contig289 | UniProt: Q18529_CAEEL Protein ID: AAK39209.2 (*Caenorhabditis elegans* unnamed protein) | 5.65 | Chitin-binding domain (IPR002557) | Chitin metabolic process | Extracellular region | Chitin binding |  |
| Contig292 | UniProt: 045599_CAEEL Protein ID: CAB07215.2 (*Caenorhabditis elegans* unnamed protein) | 5.65 | Chitin-binding domain (IPR002557) | Embryonic development; | Extracellular region | Chitin binding |  |
|  |  |  |  | Chitin metabolic process |  |  |  |
| Contig1322 | UniProt:P10986 Protein ID: AAB045 (*Caenorhabditis elegans* actin) | 5.69 | Actin-like (IPR004000); | Cytokinesis; | Cytoskeleton | Structural constituent of cytoskeleton; | Cytoskeleton (ko04812) |
|  |  |  | Actin, conserved site (IPR004001) | Embryonic development; |  | ATP binding |  |
|  |  |  |  | Growth; |  |  |  |
|  |  |  |  | Genitalia development; |  |  |  |
|  |  |  |  | Inductive cell migration; |  |  |  |
|  |  |  |  | Locomotion; |  |  |  |
|  |  |  |  | Larval development; |  |  |  |
|  |  |  |  | Reproduction |  |  |  |
| Contig143 | UniProt: VIT6_CAEEL Protein ID: AAQ91901.1 (*Caenorhabditis elegans* vitellogenin) | 5.58 | Lipid transport protein (IPR001747); | Lipid transport |  | Lipid transporter activity |  |
|  |  |  | Vitellinogen (IPR015816) |  |  |  |  |
| Contig217 | UniProt:Q18581 Protein ID: AAA98719.2 (*Caenorhabditis elegans* peptidase) | 5.54 | Peptidase M2 (IPR001558) | Growth; | Membrane | Metallopeptidase activity; | Peptidases (ko01002) |
|  |  |  |  | Genitalia development; |  | Peptidyl-dipeptidase activity |  |
|  |  |  |  | Locomotion; |  |  |  |
|  |  |  |  | Moulting cycle; |  |  |  |
|  |  |  |  | Larval development; |  |  |  |
|  |  |  |  | Oviposition; |  |  |  |
|  |  |  |  | Reproduction; |  |  |  |
|  |  |  |  | Proteolysis |  |  |  |
| Contig300 | UniProt: E5T6I6_TRISP (*Trichinella spiralis* uncharacterised protein) | 5.53 |  |  |  |  |  |
| Contig310 | UniProt: Q97J38_CLOAB (*Clostridium acetobutylicum* tetracycline resistance protein) | 5.44 | Galectin (IPR001079); |  |  |  |  |
|  |  |  | Concanavalin-like lectin (IPR008985) |  |  |  |  |
| Contig324 | UniProt: VIT1_CAEEL Protein ID: AAF59557 (*Caenorhabditis elegans* vitellogenin) | 5.43 | Lipid transport protein (IPR001747); | Lipid transport |  | Lipid transporter activity |  |
| Contig705 | UniProt:P17140 Protein ID: AAA96215.1 (*Caenorhabditis elegans* alpha-2 type IV collagen) | 5.39 | Collagen IV (IPR001442); | Embryonic development; | Basement membrane | Structural molecule activity | Cell adhesion molecules (CAMs) (ko04515) |
|  |  |  | Collagen triple-helix repeat (IPR008160); | Larval development; |  |  |  |
|  |  |  | C-type lectin (IPR016187) | Growth; |  |  |  |
|  |  |  |  | Inductive cell migration; |  |  |  |
|  |  |  |  | Locomotion; |  |  |  |
|  |  |  |  | Reproduction |  |  |  |
| Contig181 | UniProt: E5SHY6_TRISP (*Trichinella spiralis* uncharacterised protein) | 5.32 | Unintegrated (no IPR) |  |  |  |  |
| Contig21 | UniProt: Q4FX63_LEIMA (*Leishmania major* proteophosphoglycan) | 5.28 | Unintegrated (no IPR) |  |  |  |  |
| Contig28 | UniProt: Q4FX64_LEIMA (*Leishmania major* proteophosphoglycan) | 5.28 | Unintegrated (no IPR) |  |  |  |  |
| Contig22 | UniProt: Q4FX61_LEIMA (*Leishmania major* proteophosphoglycan) | 5.28 | Unintegrated (no IPR) |  |  |  |  |
| Contig27 | UniProt: Q4FX62_LEIMA (*Leishmania major* proteophosphoglycan) | 5.26 |  |  |  |  |  |
| Contig188 | UniProt: PCHTP_TRISP (*Trichinella spiralis* poly-cysteine and histidine-tailed protein) | 5.25 | Unintegrated (no IPR) |  |  |  |  |
| Contig952 | UniProt: P17139 Protein ID: CAA81584.4 (Collagen) | 5.23 | Collagen IV (IPR001442); | Embryonic development; | Basement membrane | Extracellular matrix structural constituemt | ECM-receptor interaction (ko04512) |
|  |  |  | Collagen triple-helix repeat (IPR008160); | Extracellular matrix organization; |  |  |  |
|  |  |  | C-type lectin (IPR016187) | Hatching; |  |  |  |
|  |  |  |  | Inductive cell migration; |  |  |  |
|  |  |  |  | Muscle development; |  |  |  |
|  |  |  |  | Determination of adult life span; |  |  |  |
|  |  |  |  | Larval development; |  |  |  |
|  |  |  |  | Growth; |  |  |  |
|  |  |  |  | Locomotion; |  |  |  |
|  |  |  |  | Reproduction |  |  |  |
| Contig72 | UniProt: Q7K797_CAEEL Protein ID: CAE54916.1 (Polyadenylate-binding protein) | 5.21 | RNA recognition motif (IPR000504); | Embryonic development |  | RNA binding; | RNA transport (ko03013) |
|  |  |  | Polyadenylate-binding protein (IPR002004); |  |  | Nucleic acid binding |  |
|  |  |  | Nucleotide-binding (IPR012677) |  |  |  |  |
| Contig147 | UniProt: VIT4_CAEEL Protein ID: AAK09074.1 (Vitellogenin) | 5.2 | Lipid transport protein (IPR001747); | Lipid transport |  | Lipid transporter activity |  |
|  |  |  | Vitellinogen (IPR011030) |  |  |  |  |
